# Organocatalytic synthesis of chiral tetrasubstituted allenes from racemic propargylic alcohols

**DOI:** 10.1038/s41467-017-00251-x

**Published:** 2017-09-18

**Authors:** Deyun Qian, LinLin Wu, Zhenyang Lin, Jianwei Sun

**Affiliations:** 1 Department of Chemistry, the Hong Kong University of Science and Technology (HKUST), Clear Water Bay, Kowloon, Hong Kong SAR, China; 2 The Hong Kong Branch of Chinese National Engineering Research Center for Tissue Restoration and Reconstruction, the Hong Kong University of Science and Technology (HKUST), Clear Water Bay, Kowloon, Hong Kong SAR, China; 3 HKUST Shenzhen Research Institute, Shenzhen, 518057 China

## Abstract

Although chiral allene preparation via formal S_N_2’ nucleophilic substitutions of enantioenriched propargylic derivatives or metal-catalyzed reactions of racemic propargylic derivatives has attracted considerable attention and found applications in many areas of research, direct use of propargylic alcohols instead of propargylic derivatives for catalytic asymmetric allene synthesis is unknown. Here, we show that a highly enantioselective synthesis of tetrasubstituted allenes from racemic propargylic alcohols has been realized by organocatalysis with good efficiency (up to 96% yield and 97% ee). The intermolecular C–C and C–S bond formation was achieved efficiently with simultaneous stereocontrol over the axial chirality. Furthermore, an adjacent quaternary stereocenter could also be constructed. Mechanistically, the reaction may involve efficient stereocontrol on the propargylic cation by its chiral counter anion or 1,8-conjugate addition of *para*-quinone methides. In sharp contrast to previous central chirality construction, this process employs quinone methides for axial chirality construction.

## Abstract (web summary)

Axially chiral allenes that are normally present in natural products, bioactive molecules, organocatalysts, and functional materials are usually produced from propargylic derivatives. Here, the authors show direct use of propargylic alcohols for catalytic asymmetric allene synthesis.

## Introduction

**Fig. 1:**
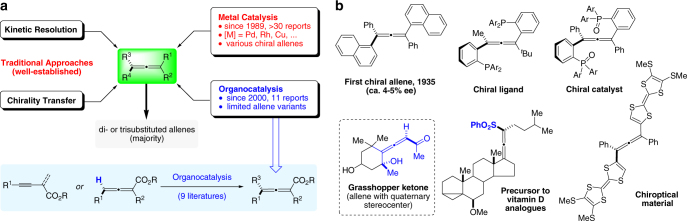
Strategies for the asymmetric synthesis of allenes and representative axially chiral allenes. **a** Previous approaches to the enantioselective synthesis of allenes, which are largely limited to the formation of di- or trisubstituted allenes. Compared with other methods, organocatalytic approaches still remain underexplored and are largely limited to allenaoates. **b** Useful chiral tetrasubstituted allenes and an allene bearing adjacent quaternary stereocenter

Axially chiral allenes are well-known subunits widely present in natural products, bioactive molecules, ligands, organocatalysts, and functional materials as well as versatile chiral building blocks in organic synthesis^1–5^. Their orthogonal cumulative π-systems often lead to complementary and unique reactivity compared with olefins and alkynes. However, the development of efficient methods to access chiral allenes from readily available starting materials has not been able to fulfill the need for their utilization in organic synthesis. Since Maitland and Mills reported the first chiral allene in 1935^6^, substantial endeavors have been made toward the stereoselective synthesis of axially chiral allenes. The well-established approaches mainly rely on either resolution of racemic allenes or chirality transfer from enantioenriched propargylic derivatives (Fig. 1a). In contrast, direct construction of the axial chirality of allenes from achiral or racemic substrates via asymmetric induction by a chiral catalyst remains substantially challenging, though highly attractive.

Recently, some progress has been made in catalyst-controlled asymmetric synthesis of chiral allenes based on metal catalysis^7–11^ and organocatalysis^12–19^. However, unfortunately these methods are largely limited to the formation of di- or trisubstituted allenes^1–19^. In particular, organocatalytic approaches have been mainly based on asymmetric protonation or deprotonation (Fig. 1a).

The lack of efficient catalytic strategies^20–23^ for the synthesis of tetrasubstituted chiral allenes is by no means an indication that these molecules are less important. Indeed, as shown in Fig. 1b, tetrasubstituted chiral allenes have been known as key units in useful chiral catalysts^24^, ligands^25^, important intermediates toward natural products^4, 26^, and molecular materials^3^. In fact, the challenges lie in simultaneous construction of a non-hydrogen- involved bond and establishment of the axial chirality, in which the catalyst must be effective in controlling both events^8, 16–18^.

## Results

### Design plan

**Fig. 2:**
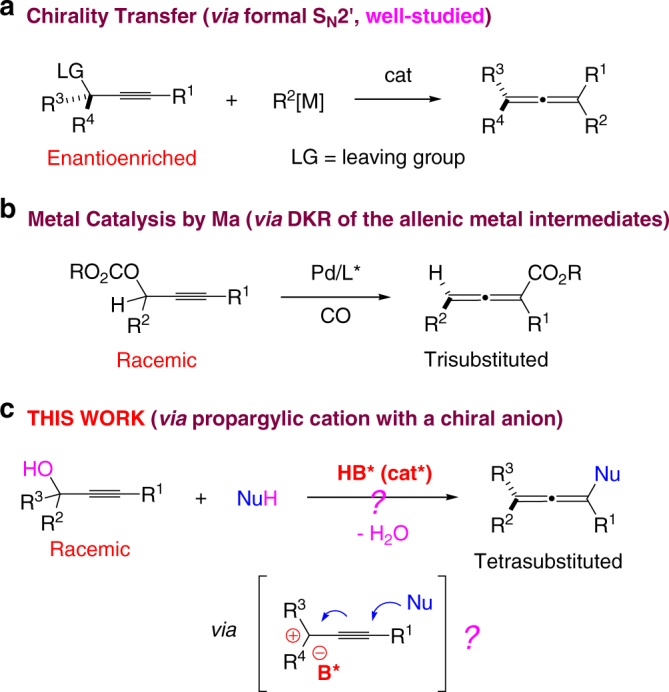
Chiral allene synthesis from propargylic compounds. **a** Well-established formal SN2′ reactions from enantioenriched propargylic derivatives. **b** A rare metalyzed-catalyzed example from racemic propargylic derivatives. **c** Our design of an organocatalytic approach from racemic propargylic alcohols

The direct transformation of propargylic alcohols or their derivatives to chiral allenes is one of the most straightforward and efficient strategies^1–5^. Specifically, chirality transfer from enantioenriched propargylic derivatives bearing a suitable leaving group by means of formal S_N_2′ reaction represents the most studied and reliable approach (Fig. 2a)^5^. However, the required pre-establishment of the central chirality in the starting material is a major limitation preventing its large scale applications. Racemization is another issue that could occur in many cases. Recently, Ma and co-worker reported a catalytic asymmetric synthesis of chiral 2,3-allenoates from racemic propargylic carbonates^9^. Mechanistically, dynamic kinetic resolution (DKR) of the crucial allenic metal intermediates plays a key role in achieving both high efficiency and enantioselectivity by catalyst control, thereby representing a pioneering study of this type (Fig. 2b). However, this elegant approach is limited to the synthesis of disubstituted allenoates. Indeed, catalytic asymmetric synthesis of fully substituted allenes from similar racemic propargylic derivatives, in particular propargylic alcohol itself, remains unknown. Inspired by this pioneering study and prompted by our continued interest in allene synthesis^14, 15^, we envisioned that, in the presence of a chiral Brønsted acid^27–29^, propargylic alcohols may generate a propargylic cation paired with a chiral counter anion (Fig. 2c)^30–32^. Subsequent nucleophilic attack may generate an allene in a stereocontrolled trajectory governed by the chiral anion. If successful, this formidably challenging process would provide an attractive approach for efficient synthesis for fully substituted chiral allenes.

### Diastereo- and enantioselective C‒C bond formation

We began to test our hypothesis using the racemic alcohol **1a** as the propargylic cation precursor and 1,3-diketone **2a** as the nucleophile. Chiral phosphoric acids and their analogues were employed as potential catalysts in view of their demonstrated versatility in asymmetric induction in chiral anion catalysis^27–32^. In the presence of the highly acidic *N*-triflylphosphoramide (*R*)**-A1**, the reaction proceeded smoothly at room temperature to provide chiral allene **3a** in high yield with good enantioselectivity within a few minutes (Table 1, entry 1). When using (*R*)**-A2** as catalyst, however, the reaction could not take place, indicating the acidity of chiral Brønsted acids plays an important role in our system. These results encouraged us to further evaluate different Brønsted acids for the transformation. As shown in Table 1, the electronic properties and the steric environment as well as the chiral backbone of the catalysts have very strong influence on the diastereoselectivity and enantioselectivity (Table 1, entries 1−6). Catalyst (*R*)-**A1** provided the best results in terms of conversion and enantioselectivity (80% ee) (Table 1, entry 1). After optimizing other reaction parameters, including solvent (Table 1, entries 7–15), temperature (Table 1, entries 16–17), etc. (more details in Supplementary Tables 1–3 of the Supplementary Information), we identified that the axially chiral allene **3a** could be obtained in 92% isolated yield with 90% ee and 6.1:1 dr (Table 1, entry 17) when the reaction was run at ‒20 °C with catalyst (*R*)-**A1** (5 mol%) and solvent CCl_4_ (0.05 M). It is noteworthy that the reaction not only furnished the axially chiral allene unit, but also simultaneously established an adjacent all-carbon quaternary stereocenter, which is a significant challenge in asymmetric synthesis^33, 34^. Notably, quaternary stereocenters are widely found in biologically important allenic natural products (e.g., Grasshopper ketone, Fig. 1b)^1^.

**Table 1:** Optimization of the reaction conditions with 1,3-diketone **2a** as nucleophile


Entry	B*H	Solvent	*t* (h)	dr^a^	ee (%)^b^
1	**A1**	toluene	0.1	2.5:1	80
2	**A2**	toluene	12	–^c^	–^c^
3	**A3**	toluene	0.2	1.4:1	52
4	**A4**	toluene	0.2	1.8:1	71
5	**A5**	toluene	0.3	1.7:1	12
6	**B1**	toluene	0.3	1.7:1	15
7	**A1**	Et_2_O	12	2.8:1	84
8	**A1**	CH_3_CN	0.5	1:1	7
9	**A1**	CH_2_Cl_2_	0.1	1.6:1	73
10	**A1**	DCE	0.1	1.4:1	72
11	**A1**	CHCl_3_	0.5	1.6:1	76
12	**A1**	CCl_4_	0.1	4.6:1	85
13	**A1**	Hexane	3	2.1:1	65
14	**A1**	CyH	1	3.5:1	80
15	**A1**	CyH/CCl_4_^d^	0.3	4.3:1	83
16^e^	**A1**	CCl_4_	1.5	5.3:1	88
17^f,g^	**A1**	CCl_4_	3	6.1:1	90

*PMP p*-MeOC_6_H_4.,_ *CyH* cyclohexane

Reaction conditions: **1a** (0.05 mmol), **2a** (0.075 mmol), catalyst (2.5 μmol), solvent (1.0 mL); unless otherwise noted, all the reactions gave >95% conversion

^a^Determined by ^1^H NMR analysis

^b^Determined by HPLC with a chiral stationary phase

^c^No reaction

^d^Cyclohexane/CCl_4_ = 1:1

^e^Run at 0 °C

^f^Run at −20 °C

^g^ **3a** was obtained in 92% isolated yield

**Fig. 3:**
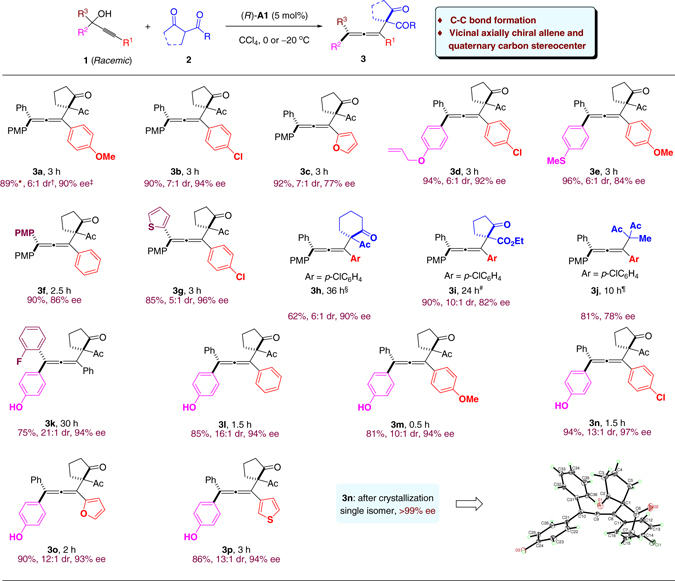
Allene formation with 1,3-dicarbonyl nucleophiles. Reaction conditions: **1** (0.2 mmol), **2** (0.3 mmol for **3a**–**3j**; 0.6 mmol for **3k**–**3p**), (*R*)-**A1** (0.01 mmol), CCl_4_ (4.0 mL), −20 °C (**3a**–**3g**) or 0 °C (**3k**–**3p**), 0.5–30 h. *Isolated yield. ^†^Determined by ^1^H NMR analysis. ^‡^Determined by HPLC with a chiral stationary phase. ^§^Run at room temperature with anhydrous MgSO_4_ (200 mg) as additive. ^#^Run at −15 °C. ^¶^Run at 0 °C. *PMP p*-MeOC_6_H_4_

With the optimized conditions, next we examined the scope of this process. As shown in Fig. 3, a variety of racemic propargylic alcohols bearing electron-rich, electron-neutral, and electron-deficient aryl moieties all smoothly participated in the efficient intermolecular C‒C bond formation process. The corresponding tetrasubstituted chiral allenes were all formed in high yield with good diastereoselectivity and good to excellent enantioselectivity (62‒96% yield, up to 21:1 dr, 77‒97% ee). Heterocycles could also be incorporated into the chiral allene products (**3c**, **3o, p**). Cyclic and acyclic 1,3-diketones as well as *β*-ketoesters were also suitable nucleophiles (**3h–j**). It is believed that the 4-methoxyphenyl group (*R* ^2^ = PMP) assists the generation and stabilization of the important cation intermediate. It also provides sufficient differentiation from the *gem*-substituent (*R* ^3^) in the subsequent enantioselective C‒C bond formation. In addition to PMP, other substituents, such as *p*-allyloxyphenyl (**3d**), *p*-sulfenylphenyl (**3e**), and *p*-hydroxyphenyl (**3k–p**) groups, can also serve the same role. The absolute configuration of product **3n** was unambiguously confirmed by X-ray crystallography.

### Enantioselective C‒S bond formation

**Fig. 4:**
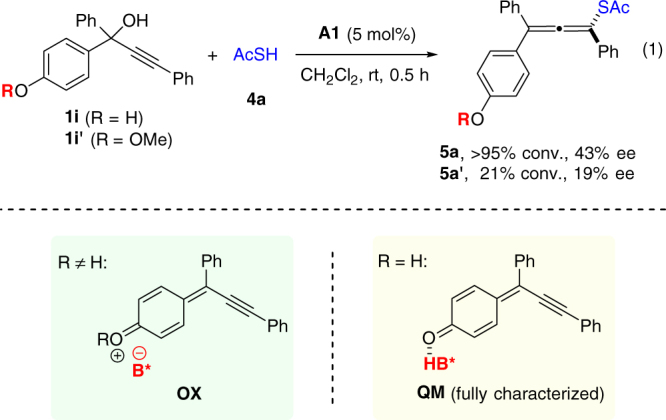
The rate difference between **1i** and **1i**’ with thioacetic acid nucleophile and the proposed intermediates. When R≠H, the reaction may proceed via ion pair OX, while when R=H, the para-quinone methide **QM** activated by hydrogen﻿ bonding might be involved

It is worth noting that in the above scope study allenes bearing a phenol unit **3k**–**3p** were not only smoothly obtained, but also formed with higher reaction rate and generally better enantiocontrol. The difference in reactivity and enantioselectivity was more pronounced with thioacetic acid as nucleophile (Fig. 4, Eq. 1). We reasoned that the mechanisms might be different for these two types of substrates (with or without free hydroxy groups). In the absence of a free hydroxy group, the key intermediate should be a cation bearing a chiral counter anion (e.g., **OX**), as initially proposed. In contrast, in the presence of a free hydroxy group, the initially formed cation could induce proton loss to form a neutral *para*-quinone methide intermediate (**QM**)^35–39^, which was indeed isolated and fully characterized when the substrate was treated with the catalyst in the absence of nucleophile (for details, see the Supporting Information). The *p*-QM can be activated by the chiral Brønsted acid catalyst for subsequent nucleophilic attack. Notably, both cases require remarkable remote stereocontrol and regiocontrol, and the latter situation is indeed an asymmetric 1,8-conjugate addition. It is worth noting that although catalytic asymmetric 1,4- and 1,6-conjugate additions have received tremendous attention, efficient catalytic asymmetric 1,8-addition has remained almost unknown and this field is still in its infancy^40–43^. Furthermore, to the best of our knowledge, the focus of previous efforts on asymmetric reactions of quinone methides all centered on the establishment of central chirality^35–39^, while this reaction establishes axial chirality with quinone methides.

Encouraged by these results, we next focused on the asymmetric synthesis of hetero-substituted allenes. The presence of a heteroatom can not only bring new synthetic and biological values, but also lead to potential useful interactions by coordination or hydrogen-bonding when needed. Therefore, the design of new enantioselective synthetic strategies to access hetero-substituted chiral allenes is highly desirable. To the best of our knowledge, examples of catalytic asymmetric synthesis of tetrasubstituted allenes bearing a hetero-substituent are essentially unknown^1–25, 44^.

While the previous standard conditions with catalyst **A1** could not lead to good enantioselectivity for the intermolecular C‒S bond formation with thioacid nucleophiles, fortunately, we were able to improve the reaction selectivity after considerable optimization efforts. As shown in Table 2, other additional acid catalysts were evaluated. Among them, the spirocyclic acid **B1** provided the best combination of catalytic activity and asymmetric induction (73% yield, 81% ee, entry 4). Further solvent screening indicated that toluene can slightly improve the yield and enantioselectivity. However, the reactivity was shut down in Et_2_O, presumably due to competing binding of ether with the catalyst (entry 6). Next, we found that the use of 3 Å molecular sieves could further improve the enantioselectivity (entry 8), while in drastic contrast, 4 Å molecular sieves resulted in almost no product formation (entry 9). Finally, evaluation of other parameters, such as concentration and temperature, identified the optimal conditions that gave both excellent enantioselectivity and good yield (entry 11).

**Table 2:** Optimization of the reaction conditions using thioacetic acid **4a** as nucleophile


Entry	B*H	Solvent	*t* (h)	Yield (%)^a^	ee (%)^b^
1	**A1**	CH_2_Cl_2_	0.5	90	43
2	**A2**	CH_2_Cl_2_	18	62	57
3	**A6**	CH_2_Cl_2_	12	40	7
4	**B1**	CH_2_Cl_2_	0.3	73	81
5	**B2**	CH_2_Cl_2_	24	51	75
6	**B1**	Et_2_O	12	<5	–
7	**B1**	Toluene	1	93	85
8^c^	**B1**	Toluene	5	87	89
9^d^	**B1**	Toluene	12	<5	–
10^c,e^	**B1**	Toluene	6	85	90
11^c,e,f^	**B1**	Toluene	12	84	94

^a^Yield was determined by ^1^H NMR analysis of the crude mixture

^b^Ee was determined by HPLC with a chiral stationary phase

^c^Run with 3 Å molecular sieves (MS, 15 mg)

^d^Run 4 Å MS (15 mg)

^e^c = 0.05 M

^f^Run at −5 °C

**Fig. 5:**
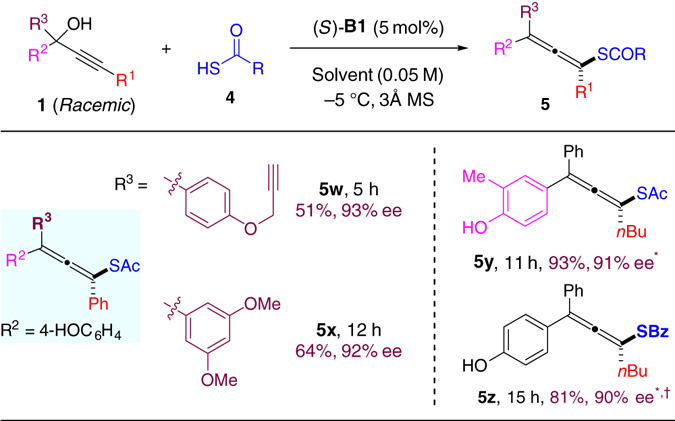
Formation of allenes **5w**–**z**. Reaction was carried out with **1** (0.3 mmol), **4** (0.6 mmol), catalyst **B1** (5 mol%), and 3 Å MS (90 mg) in solvent (6.0 mL, CCl_4_ for **5w**, **x** and CH_2_Cl_2_ for **5y**, **z**) at −5 °C. *Run without 3 Å MS. ^†^Run with **A7** at −15 °C

With the optimized conditions, we next examined the scope of the C‒S bond formation process. As shown in Table 3 and Fig. 5, a wide range of chiral tetrasubstituted allenes bearing a sulfur substituent can be prepared with good efficiency and excellent enantioselectivity. Various substituents, including aryl, alkenyl, alkyl, and hetero substituents, at different positions of the substrates did not affect the good reactivity and stereocontrol (51–96% yield, 88–94% ee). Notably, steric hindrance on the alkyne, where the nucleophile approaches, did not affect the reaction (**5u**, **v**)^8^. Substitution with heterocycles proved suitable (**5f**, **g**). Furthermore, the mild conditions can tolerate a range of functional groups, such as ethers (**5b**, **5w–x**), aromatic and aliphatic halides (**5c**, **d**, **5r**), silanes and silyl-protected alcohols (**5k**, **l**, **5q**), olefins (**5o**), alkynes (**5p**, **5w**), nitriles (**5 s**), etc. Furthermore, this protocol can also be applied to late-stage installation of a chiral allene unit to an estrone-derived complex molecule (**5 h**), in which high diastereomeric excess was observed as a result of stereocontrol by the chiral catalyst. In addition to thioacetic acid, thiobenzoic acid (BzSH) can also serve as a suitable nucleophile with slight modification of the conditions. For example, with catalyst (*R*)-**A7**, the reaction of **1 u** with BzSH smoothly afforded the corresponding chiral allene **5z** with high efficiency and enantioselectivity (90% ee). It is remarkable that the stereogenecity was efficiently relayed over eight chemical bonds. It is worth noting that the excellent generality of the process also benefits largely from the compatible in situ generation of the unstable *p*-QMs.

**Table 3:** Allenes **5a–v** formation with thioacetic acid

	

Reaction was carried out with **1** (0.3 mmol), thioacetic acid **4a** (0.6 mmol), catalyst **B1** (5 mol%), and 3 Å MS (90 mg) in solvent (6.0 mL, toluene for **5a–h** and CH_2_Cl_2_ for others, or otherwise noted) at −5 °C

^a^Run in CCl_4_ solvent

^b^ **B1** (7.5 mol%) and thioacetic acid (0.75 mmol)

^c^Run without 3 Å MS

### Product transformations

**Fig. 6:**
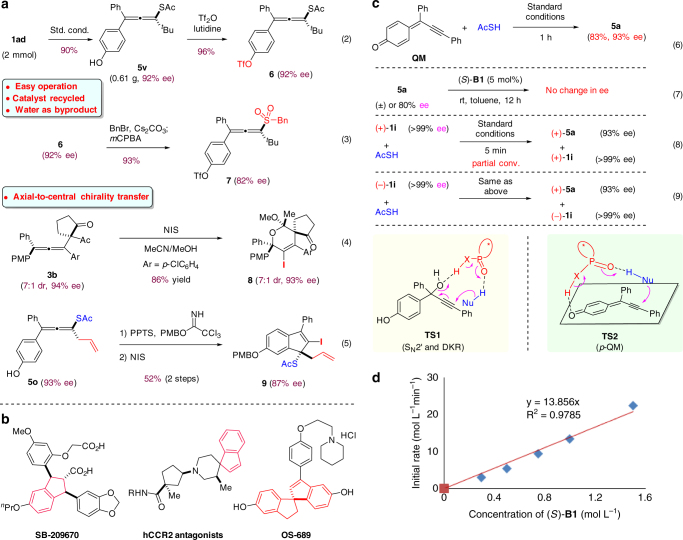
Representative product transformations and mechanistic studies. **a** Triflation of the free hydroxy group (Eq. 2), transformation of the thioacetate moiety to a sulfone unit (Eq. 3), and NIS-mediated cyclizations of the chiral tetrasubstituted allenes proceed via the axial-to-central chirality transfer (Eqs. 4–5). **b** Important molecules bearing chiral indene or indane units. **c** Control experiments and possible mechanism. The reactions suggest that the 1,8-addition to *p*-QM is rate-determining (Eq. 6) and the conjugate addition step is irreversible (Eq. 7). The product stereochemistry was purely determined by the chiral catalyst, regardless of the absolute configuration of substrate (Eqs. 8–9), which rules out the possible S_N_2’ pathway (via **TS1**). **d** Kinetic study indicated that the reaction is first-order in catalyst, suggesting that one catalyst molecule is involved in the rate-determining transition state

To further demonstrate the utility of the chiral allene products from our protocol, we carried out a preparative-scale synthesis of allene **5v** and some transformations. To our delight, comparably high efficiency and enantioselectivity (0.6 g, 90% yield, 92% ee) were observed (Fig. 6a, Eq. 2). Moreover, catalyst **B1** could be recycled without loss of catalytic activity. These results suggest that our reaction is amenable to large-scale application. Triflation of the free hydroxy group in **5v** efficiently provided the corresponding triflate compound, which is poised for further functionalizations, such as cross-coupling^45^. The thioacetate moiety can also be smoothly converted to a sulfone unit (**7**, Eq. 3). Thus, another family of useful activated allenes, allenyl sulfones, can be made in their enantioenriched form^46^. Furthermore, the tetrasubstituted allene products can be transformed to other useful molecules, demonstrating the ability of axial-to-central chirality transfer^1–5^. For example, iodocyclization of allene **3b** in the presence of NIS and MeOH furnished chiral spirocyclic molecule **8** (Eq. 4). In addition, chiral indene derivative **9** can be obtained from allene **5o**. Notably, chiral indene/indane is an important subunit in medicinally important molecules (Fig. 6b)^47–49^.

### Mechanistic studies

To gain further insight into the reaction mechanism, we carried out some control experiments. Firstly, subjecting the fully characterized pure **QM** to the standard conditions led to successful formation of chiral allene **5a** in essentially the same yield and enantiomeric excess as the standard protocol from **1i** (Fig. 6c, Eq. 6). Indeed, in the standard reaction, we could observe rapid consumption of **1i** followed by accumulation and slow disappearance of **QM**. These observations suggest that the 1,8-addition to *p*-QM is rate-determining. Moreover, when the allene product **5a** (either racemic or enantioenriched) was treated with catalyst **B1**, no change in enantiopurity was observed, indicating that the conjugate addition step is irreversible (Eq. 7). Furthermore, the enantiopure tertiary alcohol substrate **1i** was subjected to the standard conditions (Eqs. 8–9). At partial conversion, the recovered substrate **1i** remained enantiopure, indicating the first step (dehydration) is irreversible, presumably due to the elimination of water by molecular sieves. Without molecular sieves, essentially the same results were obtained (**1i**: **>**99% ee) along with some enone byproducts generated through the classical Meyer−Schuster rearrangement^50^. Moreover, it was found that the product stereochemistry was purely determined by the chiral catalyst, regardless of the absolute configuration of substrate **1i** (Eqs. 8–9). This observation is consistent with the intermediacy of *p*-QM. It also rules out the possible S_N_2’ pathway (via **TS1**), in which DKR might be operative^1–5^. Finally, kinetic study indicated that the reaction is first-order in catalyst, suggesting that one catalyst molecule is involved in the rate-determining transition state (Fig. 6d). Thus, we proposed a possible transition state (**TS2**, Fig. 6c), in which the catalyst plays a bifunctional role for activation of both partners and remote stereocontrol (see the Supplementary Note 4 for preliminary DFT calculations).

## Discussion

In summary, we have developed an efficient and unified method for catalytic asymmetric synthesis of chiral tetrasubstituted allenes from racemic propargylic alcohols. It is not only an early demonstration of highly enantioselective chiral ion-pair catalysis involving a formal propargylic carbocation as well as catalytic asymmetric 1,8-conjugate addition, but also a new addition to the small family of catalytic syntheses of chiral hetero- and tetrasubstituted allenes. The chiral Brønsted acid catalysts of proper choice not only allow in situ generation of the reactive intermediates, thereby simplifying substrate preparation and expanding functional group compatibility, but also ensure excellent remote stereocontrol. Control experiments provided important insights into the reaction mechanism. The enantioenriched allene products are also precursors to other useful chiral molecules.

## Methods

### General information

Reagents were purchased at the highest commercial quality and used without further purification, unless otherwise stated. Anhydrous dichloromethane, toluene, diethyl ether, and tetrahydrofuran were purified by the Innovative^®^ solvent purification system. ^1^H, ^13^C, and ^19^F nuclear magnetic resonance (NMR) spectra were collected on a Bruker AV 400 MHz NMR spectrometer using residue solvent peaks as an internal standard (^1^H NMR: CDCl_3_ at 7.26 ppm, CD_2_Cl_2_ at 5.32 ppm, acetone-*d*_*6*_ at 2.05 ppm; ^13^C NMR: CDCl_3_ at 77.0 ppm, CD_2_Cl_2_ at 54.0 ppm, acetone-*d*_*6*_ at 29.8 ppm). Mass spectra were collected on an Agilent GC/MS 5975 C system, a MALDI Micro MX mass spectrometer, or an API QSTAR XL System. Infrared spectra were recorded on Bruker TENSOR 27 spectrometer and reported in terms of frequency of absorption (cm^−1^). Optical rotations were measured on JASCO P-2000 polarimeter with [α]_D_ values reported in degrees; concentration (*c*) is in 10 mg/mL. The enantiomeric excess values were determined by chiral high-performance liquid chromatography (HPLC) using an Agilent 1200 LC instrument. Flash column chromatography was performed on silica gel. NMR and high performance liquid chromatography (HPLC) spectra are supplied for all compounds: see Supplementary Figs 1–218. See Supplementary Method and Notes 1–2 for the characterization data of compounds **1**–**9**.

All the racemic products were prepared using the same substrates with the racemic phosphoric acid derived from BINOL as catalyst.

### General procedure for the synthesis of axially chiral allenes **3**

At room temperature, an oven-dried 10-mL vial was charged with a mixture of the alcohol substrate **1** (0.2 mmol), the carbon nucleophile **2** (0.3 mmol, 1.5 equiv), and CCl_4_ (3.8 mL). The reaction mixture was cooled to ‒20 °C (for **3a–j**) or 0 °C (for **3k–p**) unless otherwise specified, and a solution of the catalyst (*R*)-**A1** (10 μmol, 5 mol%) in CCl_4_ (0.2 mL) was added in one portion. The reaction mixture was stirred at the same temperature. The progress was monitored by thin layer chromatography. Upon completion, the crude mixture was directly subjected to silica gel flash chromatography (5–10% EtOAc in hexanes) to afford the pure product **3**.

### General procedure for the synthesis of axially chiral allenes **5**

At room temperature, an oven-dried 10-mL vial was charged with a mixture of the alcohol substrate **1** (0.3 mmol), 3 Å molecular sieves (90 mg), and the solvent (6.0 mL, toluene for **5a–g** and DCM for others, or noted otherwise). The reaction mixture was cooled to −5 °C, and a solution of the catalyst (*S*)-**B1** (0.015 mmol, 5 mol%) in the same solvent (0.2 mL) and thioacetic acid **4a** (0.6 mmol, 42.9 uL) were added in one portion. The reaction progress was monitored by thin layer chromatography. Upon completion, the reaction mixture was directly subjected to silica gel column flash chromatography (10% EtOAc and 1% AcOH in hexanes, the column was pretreated with 10% AcOH in hexanes) to afford the desired product **5**.

### Data availability

The X-ray crystallographic coordinates for structures reported in this article have been deposited at the Cambridge Crystallographic Data Centre (CCDC), under deposition number of CCDC 1498348. These data can be obtained free of charge from The Cambridge Crystallographic Data Centre via http://www.ccdc.cam.ac.uk/data_request/cif. All other data is available from the authors upon reasonable request.

## Electronic supplementary material

Supplementary Information
