# Supplementary material for: Organocatalytic synthesis of chiral tetrasubstituted allenes from racemic propargylic alcohols
Source: Nat Commun. 2017 Sep 18;8:567. doi: 10.1038/s41467-017-00251-x (PMC5603569; doi:10.1038/s41467-017-00251-x)
Supplement: Supplementary file 1 — Supplementary Information [file 41467_2017_251_MOESM1_ESM.pdf]

File Name: Supplementary Information
Description: Supplementary Figures, Supplementary Tables, Supplementary Notes, Supplementary Methods and Supplementary References

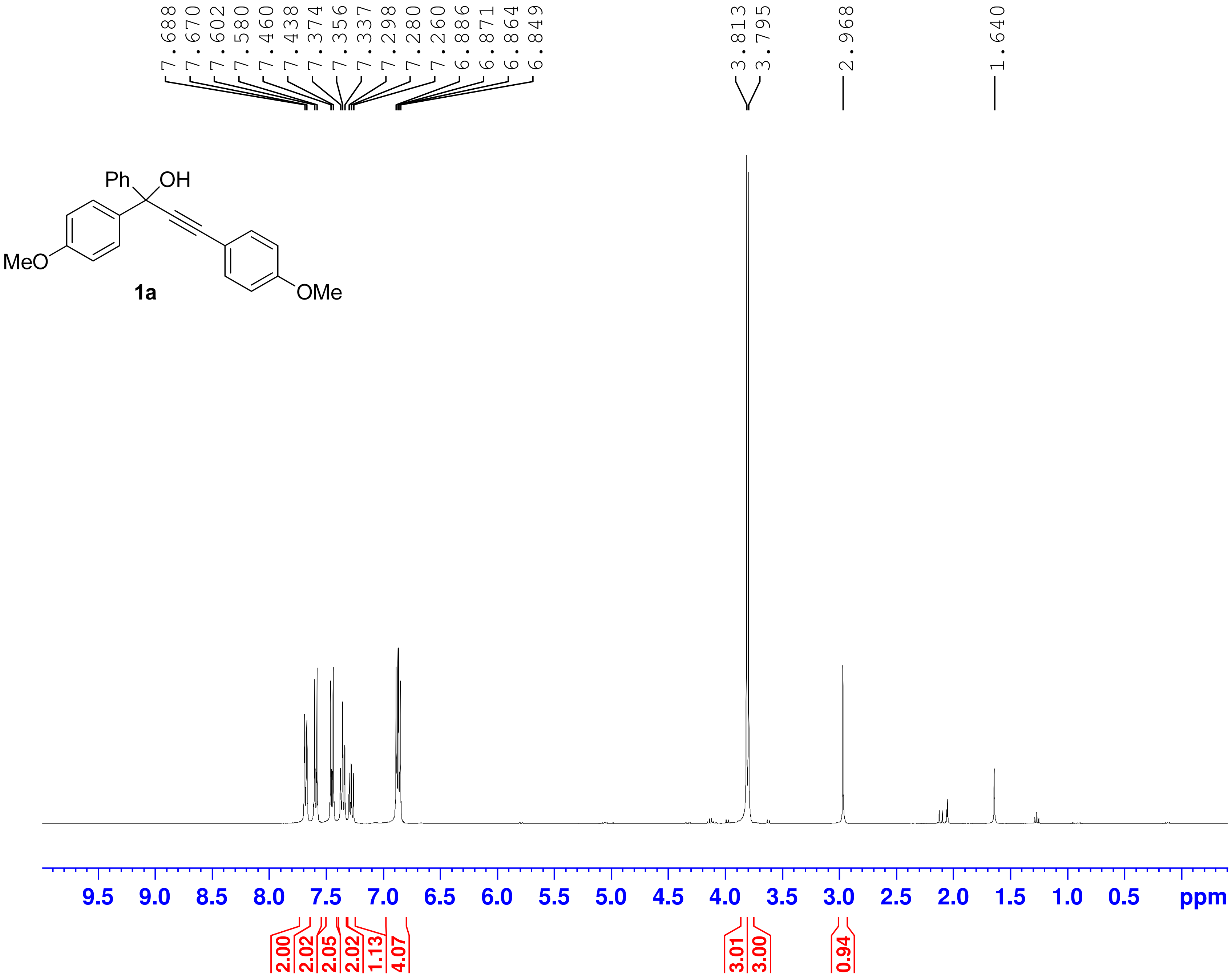


```
Current Data Parameters
NAME        qdy-40004-1 H
EXPNO                   1
PROCNO                  1

F2 - Acquisition Parameters
Date_            20161214
Time                22.02
INSTRUM             spect
PROBHD    5 mm DUL 13C-1
PULPROG              zg30
TD                  65536
SOLVENT             CDCl3
NS                      4
DS                      0
SWH              8012.820 Hz
FIDRES           0.122266 Hz
AQ              4.0894465 sec
RG                  62.93
DW                 62.400 usec
DE                   6.50 usec
TE                  296.6 K
D1             1.00000000 sec
TD0                     1

======== CHANNEL f1 ========
SFO1          400.1324710 MHz
NUC1                   1H
P1                  14.30 usec
PLW1           9.10000038 W

F2 - Processing parameters
SI                  65536
SF            400.1300092 MHz
WDW                    EM
SSB      0
LB                   0.30 Hz
GB       0
PC                   1.00
```

Supplementary Figure 1. ^{1}H NMR spectrum for 1a

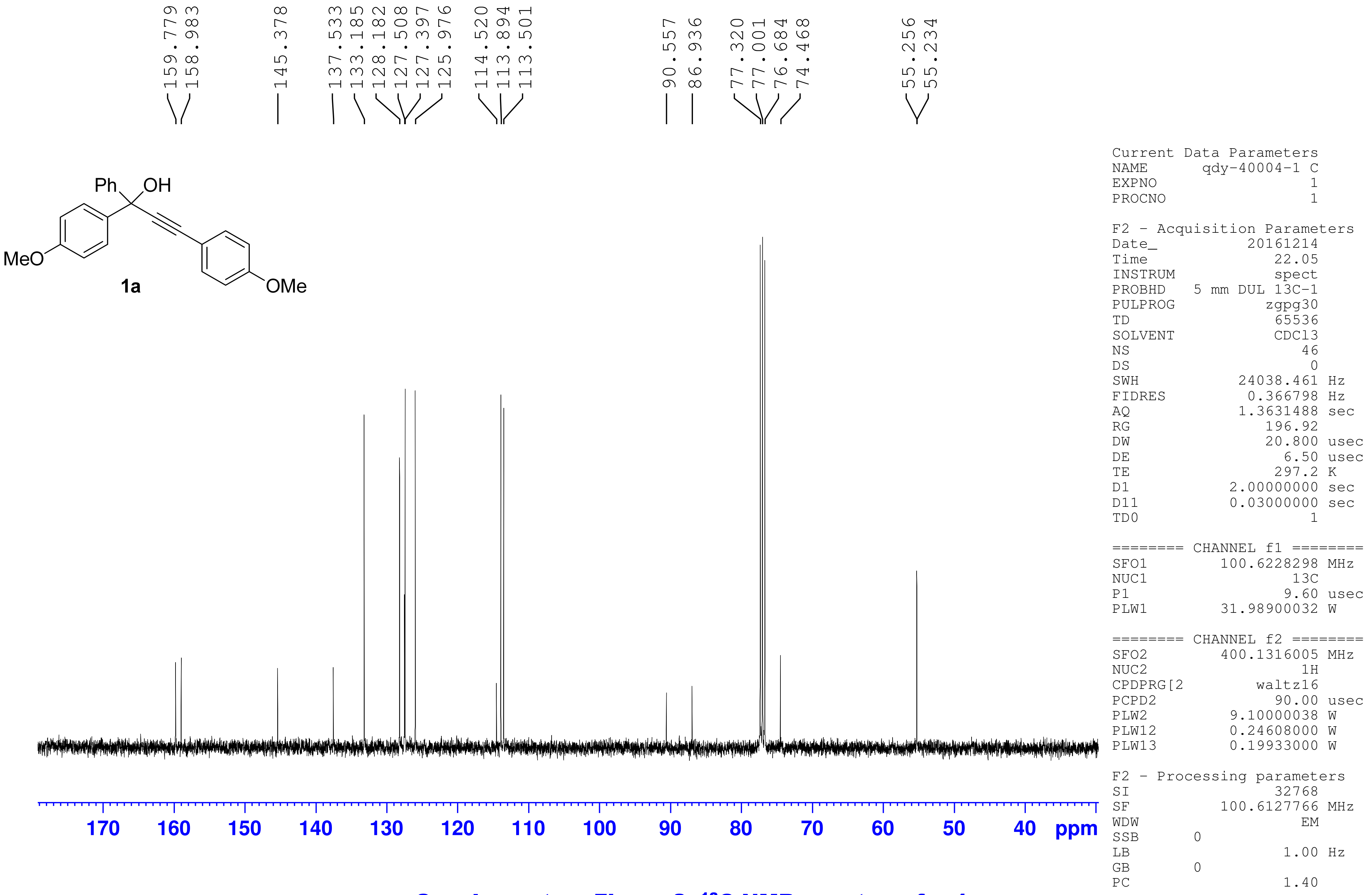

Supplementary Figure 2. ^{13}C NMR spectrum for 1a

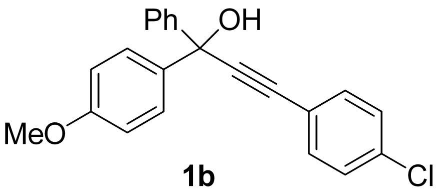

1b

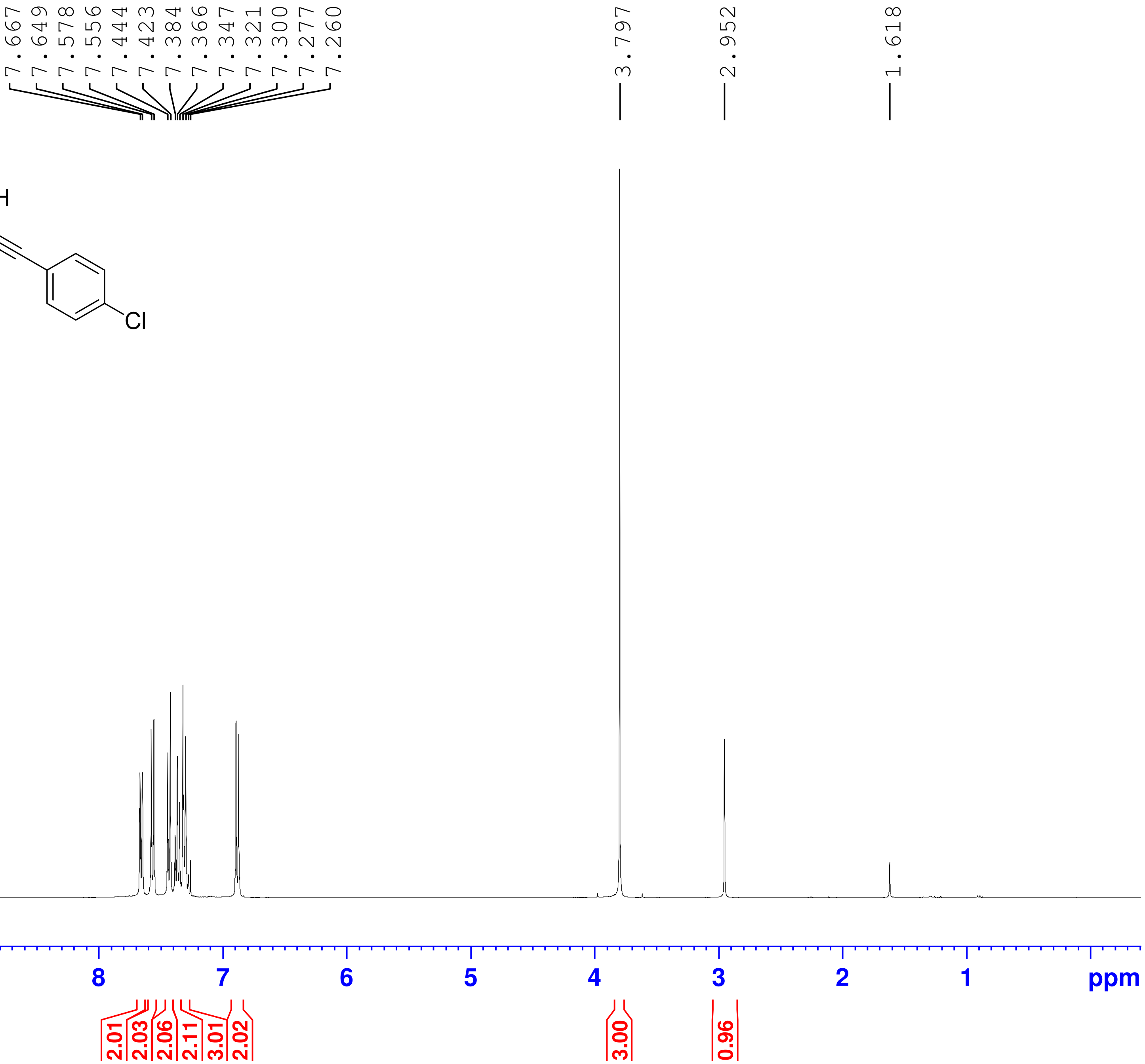


```
Current Data Parameters
NAME       qdy-40006-1 H
EXPNO                  1
PROCNO                 1

F2 - Acquisition Parameters
Date_           20161219
Time               19.36
INSTRUM            spect
PROBHD   5 mm PABBO BB/
PULPROG             zg30
TD                 65536
SOLVENT            CDCl3
NS                     4
DS                     0
SWH             8012.820 Hz
FIDRES          0.122266 Hz
AQ             4.0894465 sec
RG                 62.93
DW                62.400 usec
DE                  6.50 usec
TE                 295.8 K
D1            1.00000000 sec
TD0                    1

======== CHANNEL f1 ========
SFO1         400.1324710 MHz
NUC1                  1H
P1                 14.50 usec
PLW1         11.99499989 W

F2 - Processing parameters
SI                 65536
SF           400.1300092 MHz
WDW                   EM
SSB       0
LB                  0.30 Hz
GB        0
PC                  1.00
```

Supplementary Figure 3. ^{1}H NMR spectrum for 1b

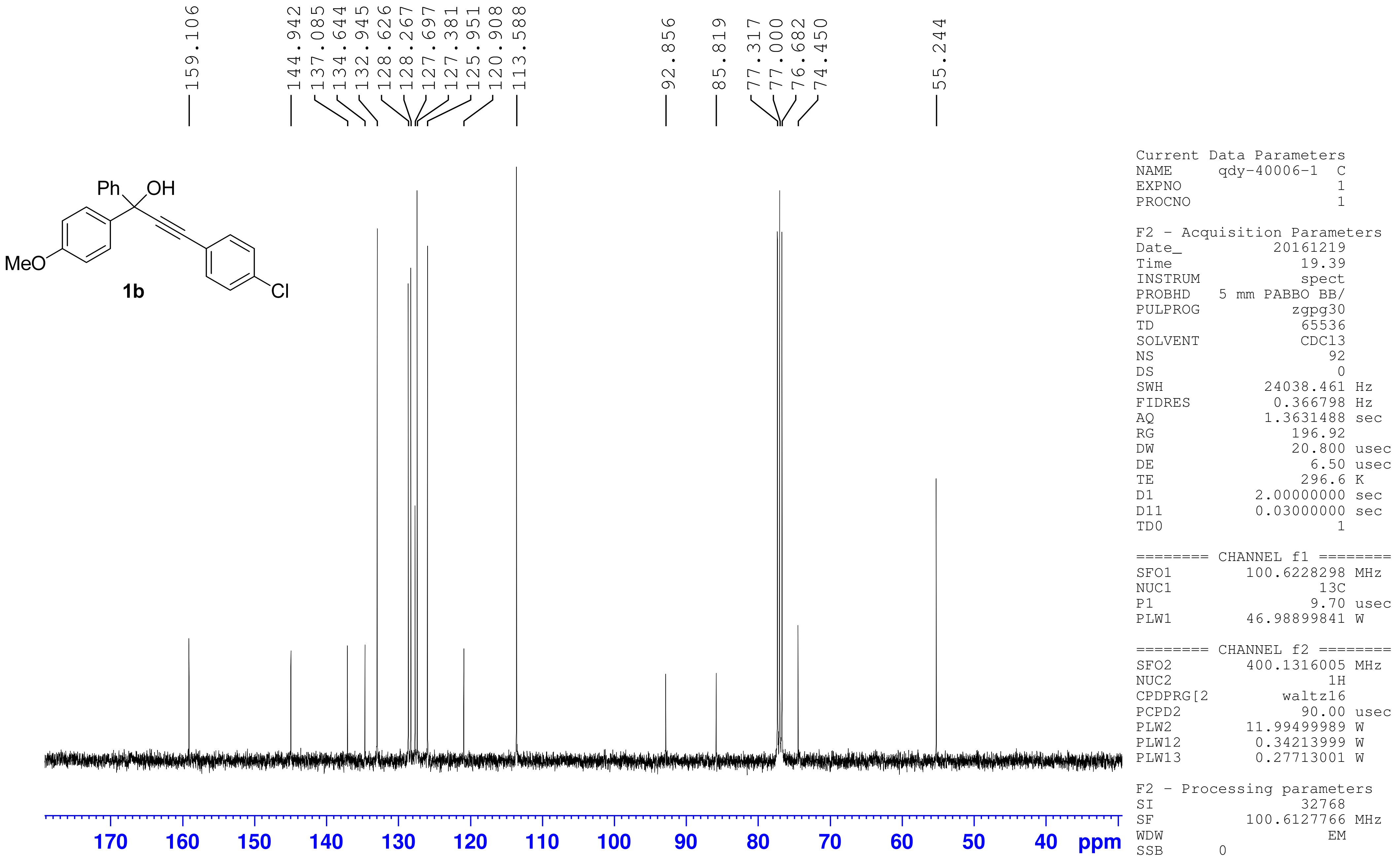

Supplementary Figure 4. ^{13}C NMR spectrum for 1b

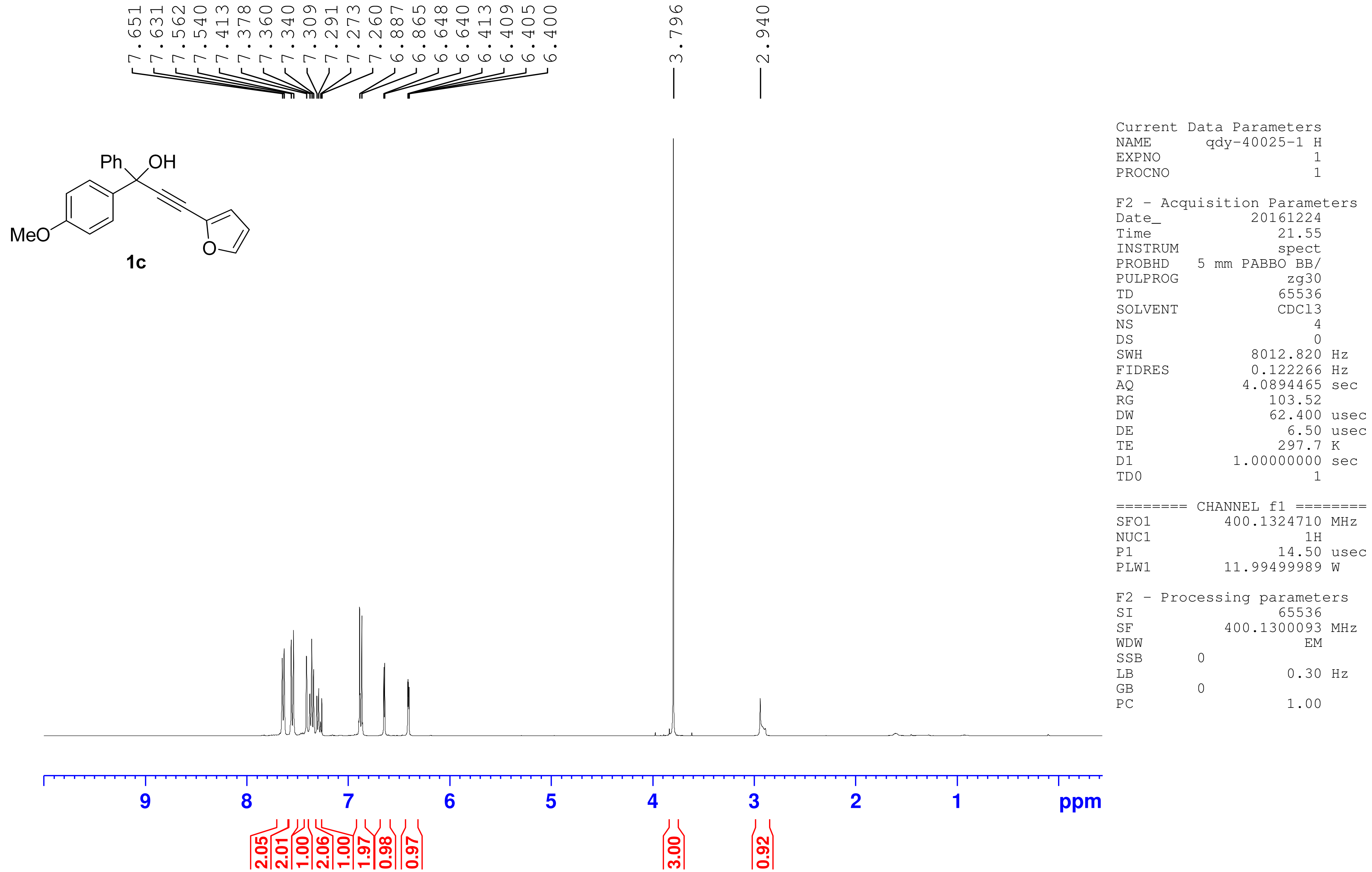

Supplementary Figure 5. ¹H NMR spectrum for 1c

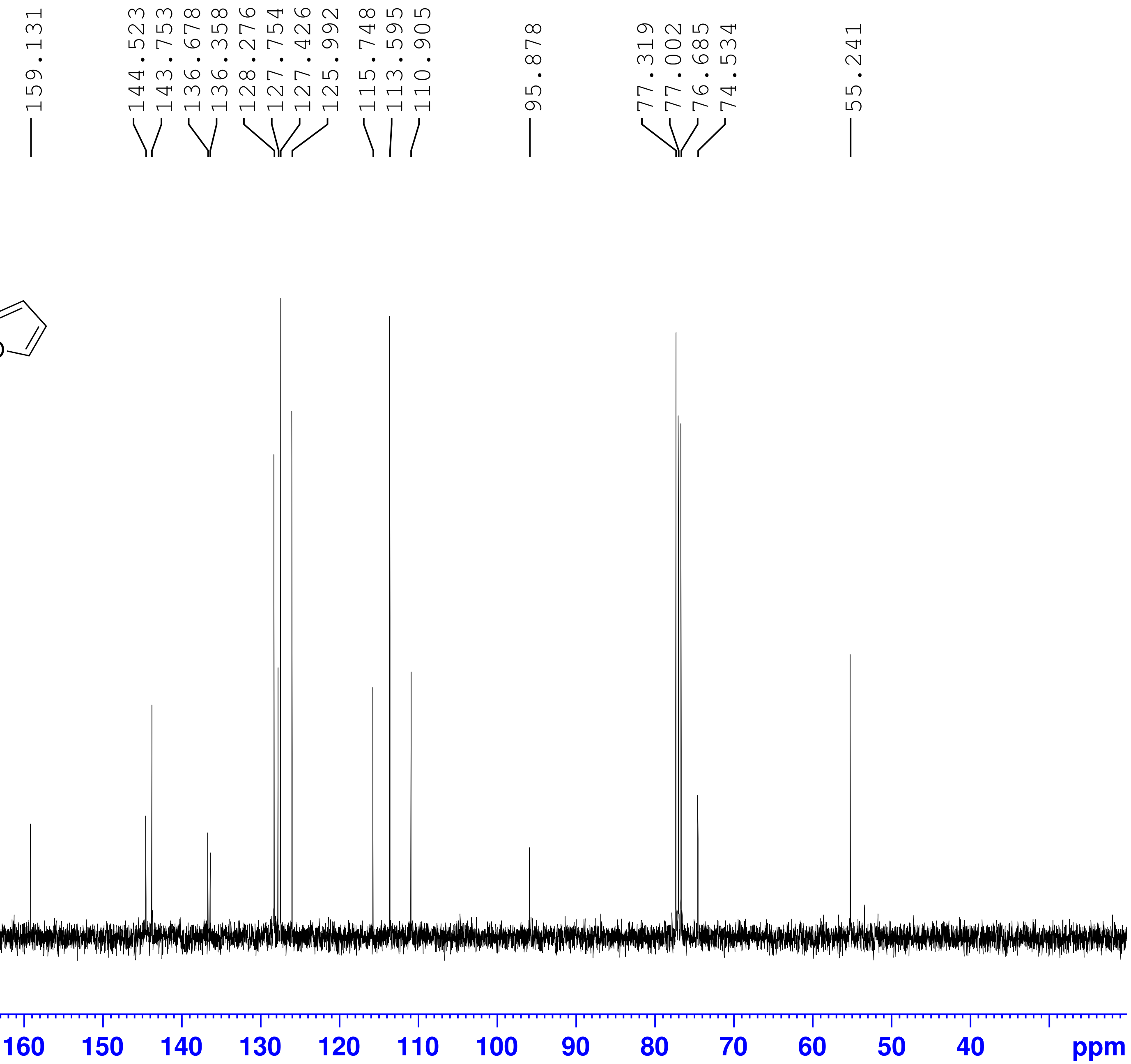

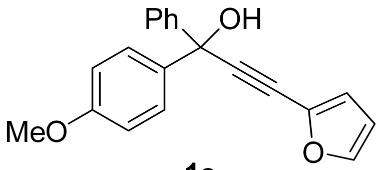

1c

Current Data Parameters
NAME qdy-40025-1 C
EXPNO 1
PROCNO 1

F2 - Acquisition Parameters
Date_ 20161224
Time 16.32
INSTRUM spect
PROBHD 5 mm PABBO BB/
PULPROG zgpg30
TD 65536
SOLVENT CDCl3
NS 29
DS 0
SWH 24038.461 Hz
FIDRES 0.366798 Hz
AQ 1.3631488 sec
RG 196.92
DW 20.800 usec
DE 6.50 usec
TE 295.4 K
D1 2.00000000 sec
D11 0.03000000 sec
TD0 1

======== CHANNEL f1 ========
SFO1 100.6228298 MHz
NUC1 13C
P1 9.70 usec
PLW1 46.98899841 W

======== CHANNEL f2 ========
SFO2 400.1316005 MHz
NUC2 1H
CPDPRG[2 waltz16
PCPD2 90.00 usec
PLW2 11.99499989 W
PLW12 0.34213999 W
PLW13 0.27713001 W

F2 - Processing parameters
SI 32768
SF 100.6127773 MHz
WDW EM
SSB 0
LB 1.00 Hz
GB 0
PC 1.40

Supplementary Figure 6. ^{13}C NMR spectrum for 1c

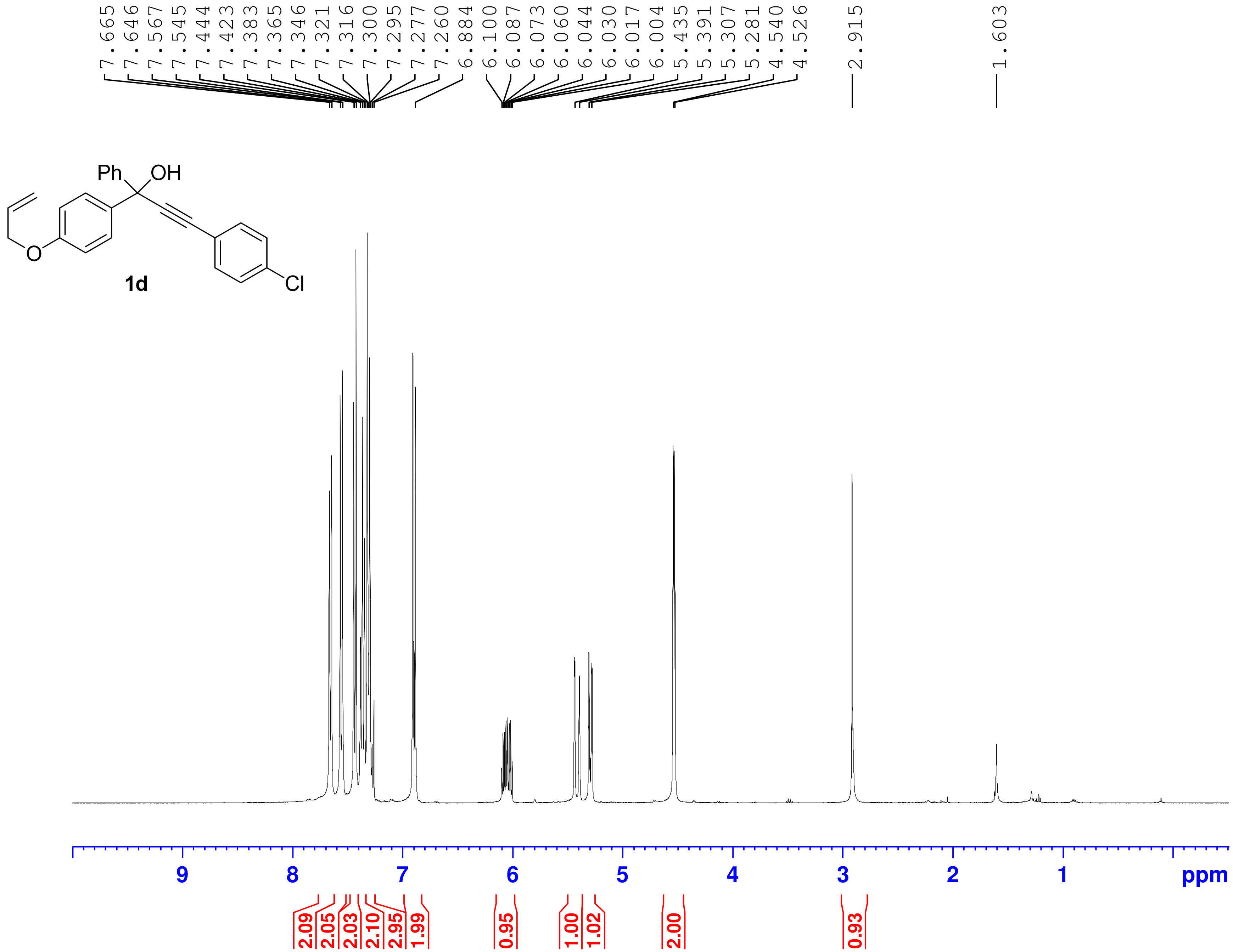

Current Data Parameters
NAME qdy-40021-2 H
EXPNO 1
PROCNO 1

F2 - Acquisition Parameters
Date_ 20161221
Time 16.54
INSTRUM spect
PROBHD 5 mm PABBO BB/
PULPROG zg30
TD 65536
SOLVENT CDCl3
NS 4
DS 0
SWH 8012.820 Hz
FIDRES 0.122266 Hz
AQ 4.0894465 sec
RG 54.81
DW 62.400 usec
DE 6.50 usec
TE 297.4 K
D1 1.00000000 sec
TD0 1

======== CHANNEL f1 ========
SFO1 400.1324710 MHz
NUC1 1H
P1 14.50 usec
PLW1 11.99499989 W

F2 - Processing parameters
SI 65536
SF 400.1300093 MHz
WDW EM
SSB 0
LB 0.30 Hz
GB 0
PC 1.00

Supplementary Figure 7. ^{1}H NMR spectrum for 1d

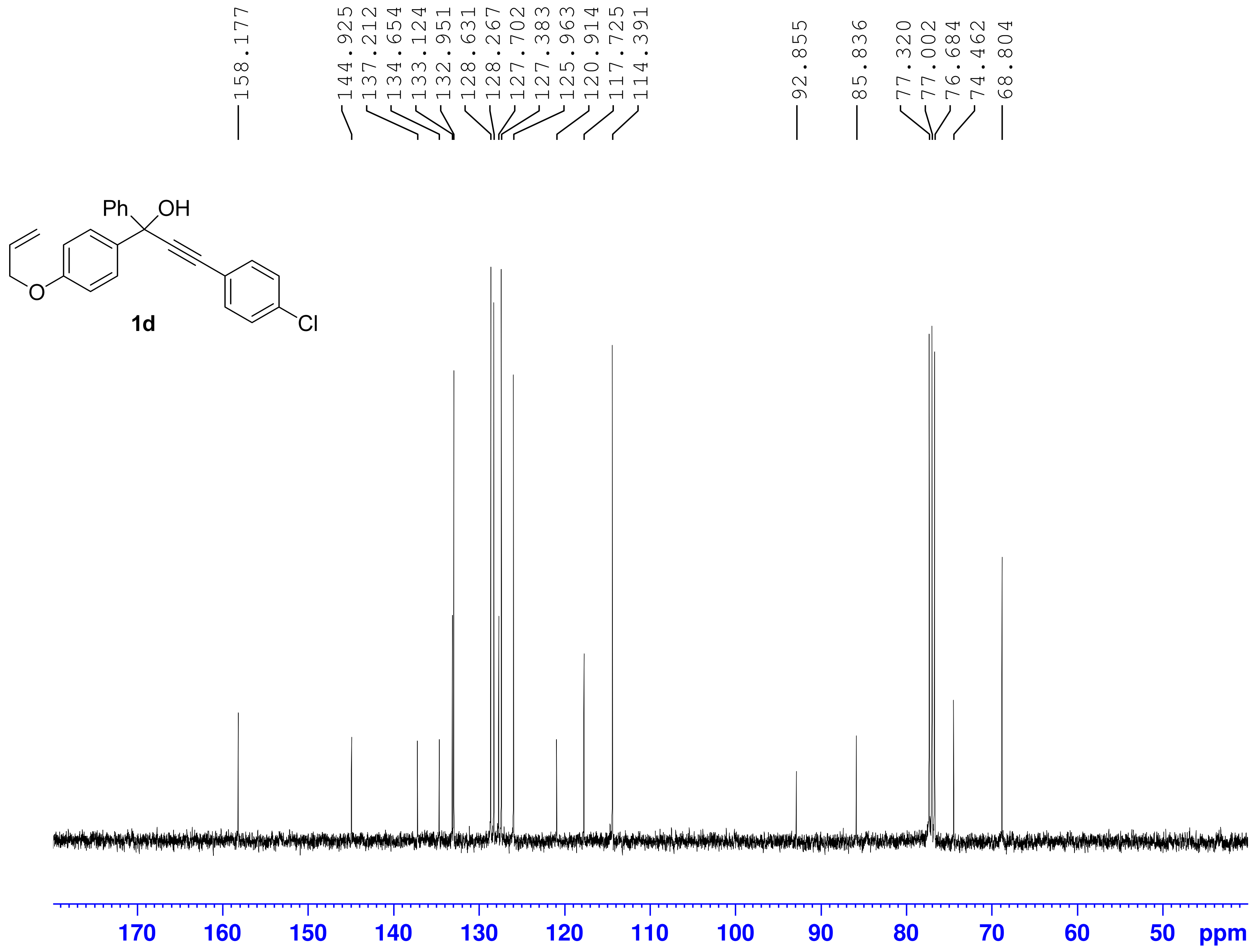

Current Data Parameters
NAME qdy-40021-2 C
EXPNO 1
PROCNO 1

F2 - Acquisition Parameters
Date_ 20161221
Time 16.56
INSTRUM spect
PROBHD 5 mm PABBO BB/
PULPROG zgpg30
TD 65536
SOLVENT CDCl3
NS 71
DS 0
SWH 24038.461 Hz
FIDRES 0.366798 Hz
AQ 1.3631488 sec
RG 196.92
DW 20.800 usec
DE 6.50 usec
TE 298.2 K
D1 2.00000000 sec
D11 0.03000000 sec
TD0 1

======== CHANNEL f1 ========
SFO1 100.6228298 MHz
NUC1 13C
P1 9.70 usec
PLW1 46.98899841 W

======== CHANNEL f2 ========
SFO2 400.1316005 MHz
NUC2 1H
CPDPRG[2 waltz16
PCPD2 90.00 usec
PLW2 11.99499989 W
PLW12 0.34213999 W
PLW13 0.27713001 W

F2 - Processing parameters
SI 32768
SF 100.6127759 MHz
WDW EM
SSB 0
LB 1.00 Hz
GB 0
PC 1.40

Supplementary Figure 8. ^{13}C NMR spectrum for 1d

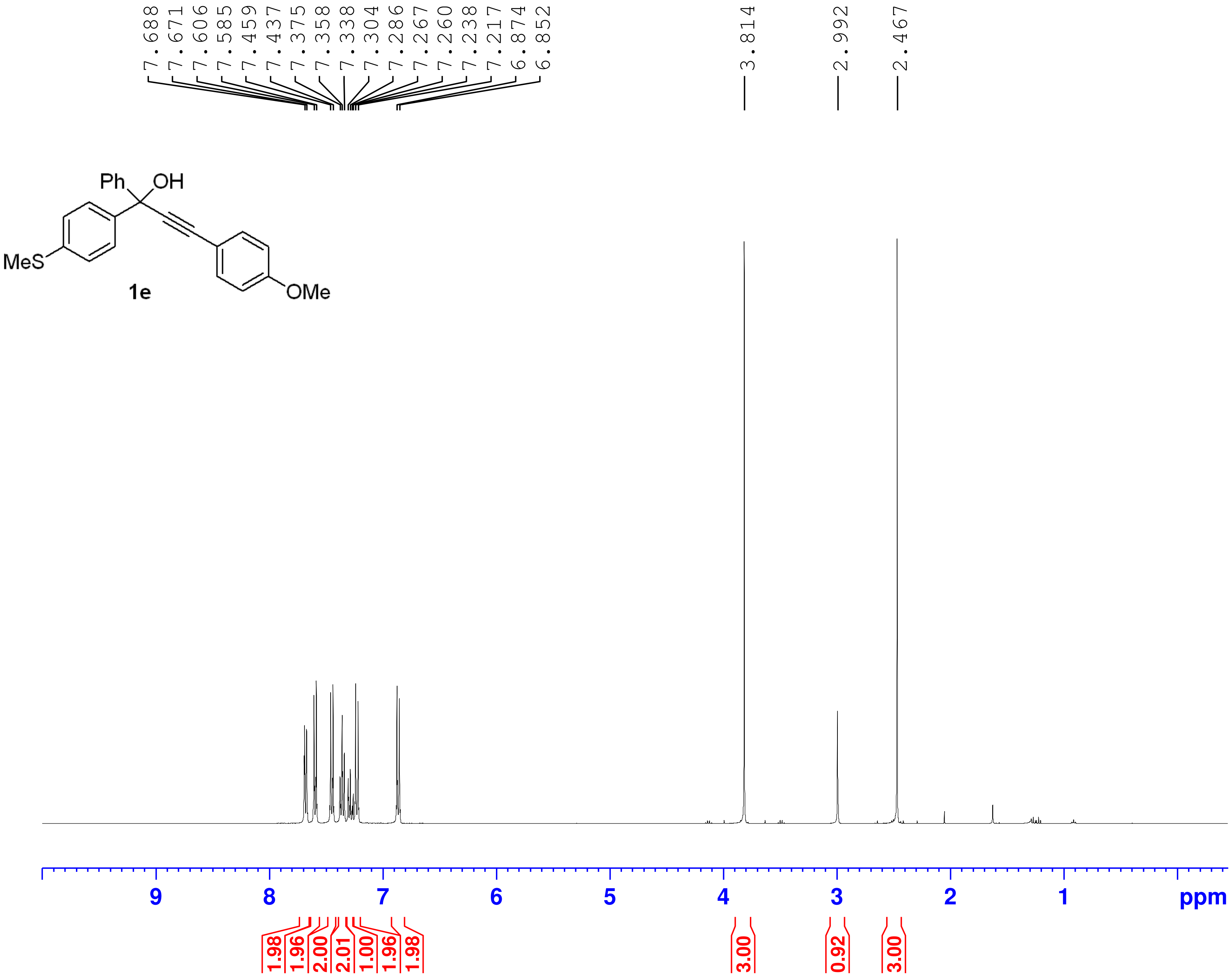

Current Data Parameters
NAME qdy-40017-1 H
EXPNO 2
PROCNO 1

F2 - Acquisition Parameters
Date_ 20161217
Time 19.36
INSTRUM spect
PROBHD 5 mm PABBO BB/
PULPROG zg30
TD 65536
SOLVENT CDCl3
NS 4
DS 0
SWH 8012.820 Hz
FIDRES 0.122266 Hz
AQ 4.0894465 sec
RG 54.81
DW 62.400 usec
DE 6.50 usec
TE 296.9 K
D1 1.00000000 sec
TD0 1

======== CHANNEL f1 ========
SFO1 400.1324710 MHz
NUC1 1H
P1 14.50 usec
PLW1 11.99499989 W

F2 - Processing parameters
SI 65536
SF 400.1300093 MHz
WDW EM
SSB 0
LB 0.30 Hz
GB 0
PC 1.00

Supplementary Figure 9. ^{1}H NMR spectrum for 1e

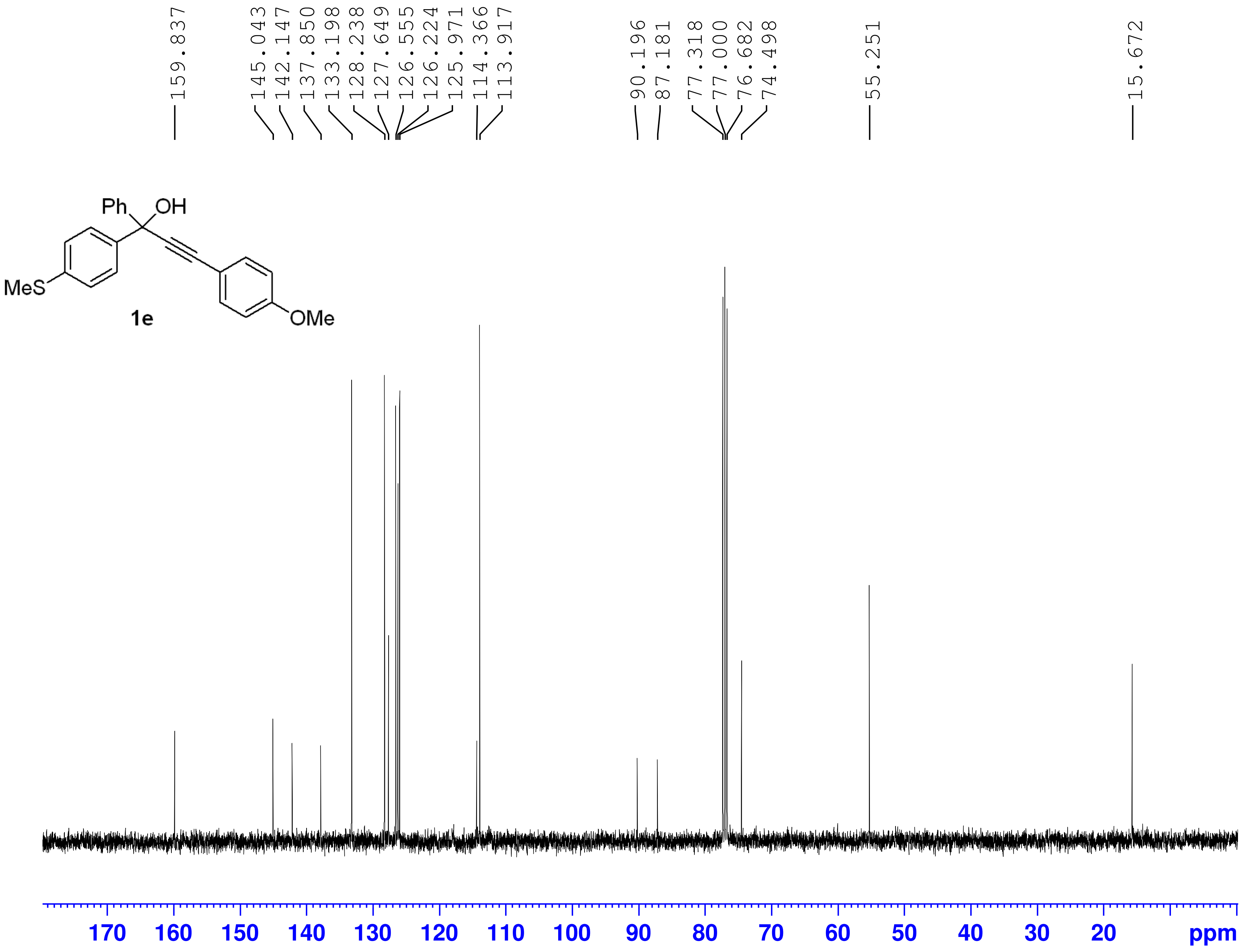


```
Current Data Parameters
NAME       qdy-40017-1 C
EXPNO                  2
PROCNO                 1

F2 - Acquisition Parameters
Date_           20161217
Time               19.40
INSTRUM            spect
PROBHD    5 mm PABBO BB/
PULPROG          zgpg30
TD                65536
SOLVENT           CDCl3
NS                  113
DS                    0
SWH           24038.461 Hz
FIDRES         0.366798 Hz
AQ            1.3631488 sec
RG               196.92
DW               20.800 usec
DE                 6.50 usec
TE                297.8 K
D1           2.00000000 sec
D11          0.03000000 sec
TD0                   1

======== CHANNEL f1 ========
SFO1        100.6228298 MHz
NUC1                13C
P1                 9.70 usec
PLW1        46.98899841 W

======== CHANNEL f2 ========
SFO2        400.1316005 MHz
NUC2                 1H
CPDPRG[2        waltz16
PCPD2             90.00 usec
PLW2        11.99499989 W
PLW12        0.34213999 W
PLW13        0.27713001 W

F2 - Processing parameters
SI                32768
SF          100.6127780 MHz
WDW                  EM
SSB      0
LB                 1.00 Hz
GB       0
PC                 1.40
```

Supplementary Figure 10. ^{13}C NMR spectrum for 1e

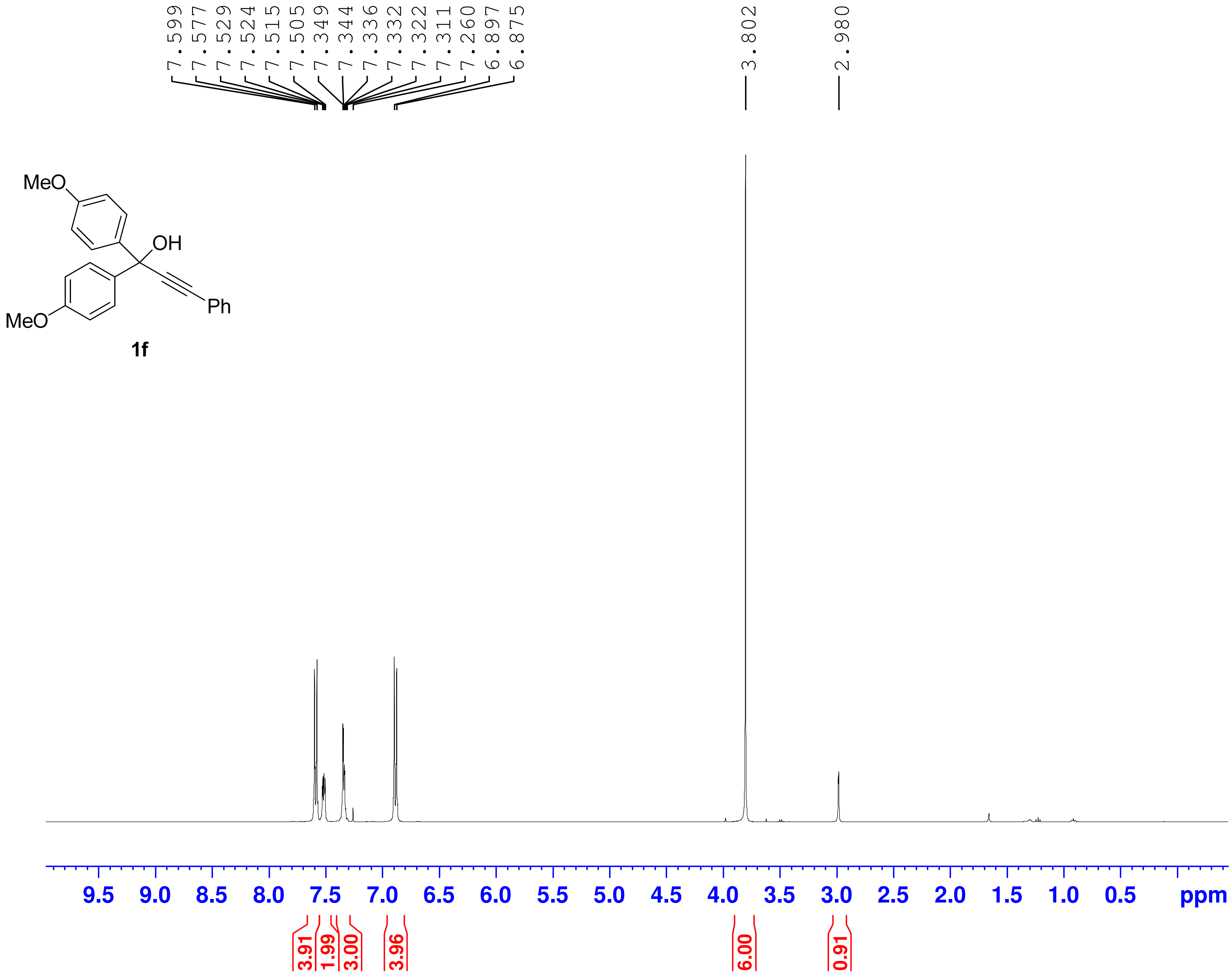

Current Data Parameters
NAME qdy-30184 H
EXPNO 2
PROCNO 1

F2 - Acquisition Parameters
Date_ 20170116
Time 14.10
INSTRUM spect
PROBHD 5 mm PABBO BB/
PULPROG zg30
TD 65536
SOLVENT CDCl3
NS 4
DS 0
SWH 8012.820 Hz
FIDRES 0.122266 Hz
AQ 4.0894465 sec
RG 54.81
DW 62.400 usec
DE 6.50 usec
TE 295.5 K
D1 1.00000000 sec
TD0 1

======== CHANNEL f1 ========
SFO1 400.1324710 MHz
NUC1 1H
P1 14.50 usec
PLW1 11.99499989 W

F2 - Processing parameters
SI 65536
SF 400.1300100 MHz
WDW EM
SSB 0
LB 0.30 Hz
GB 0
PC 1.00

Supplementary Figure 11. ^{1}H NMR spectrum for 1f

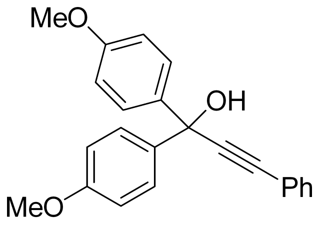

1f

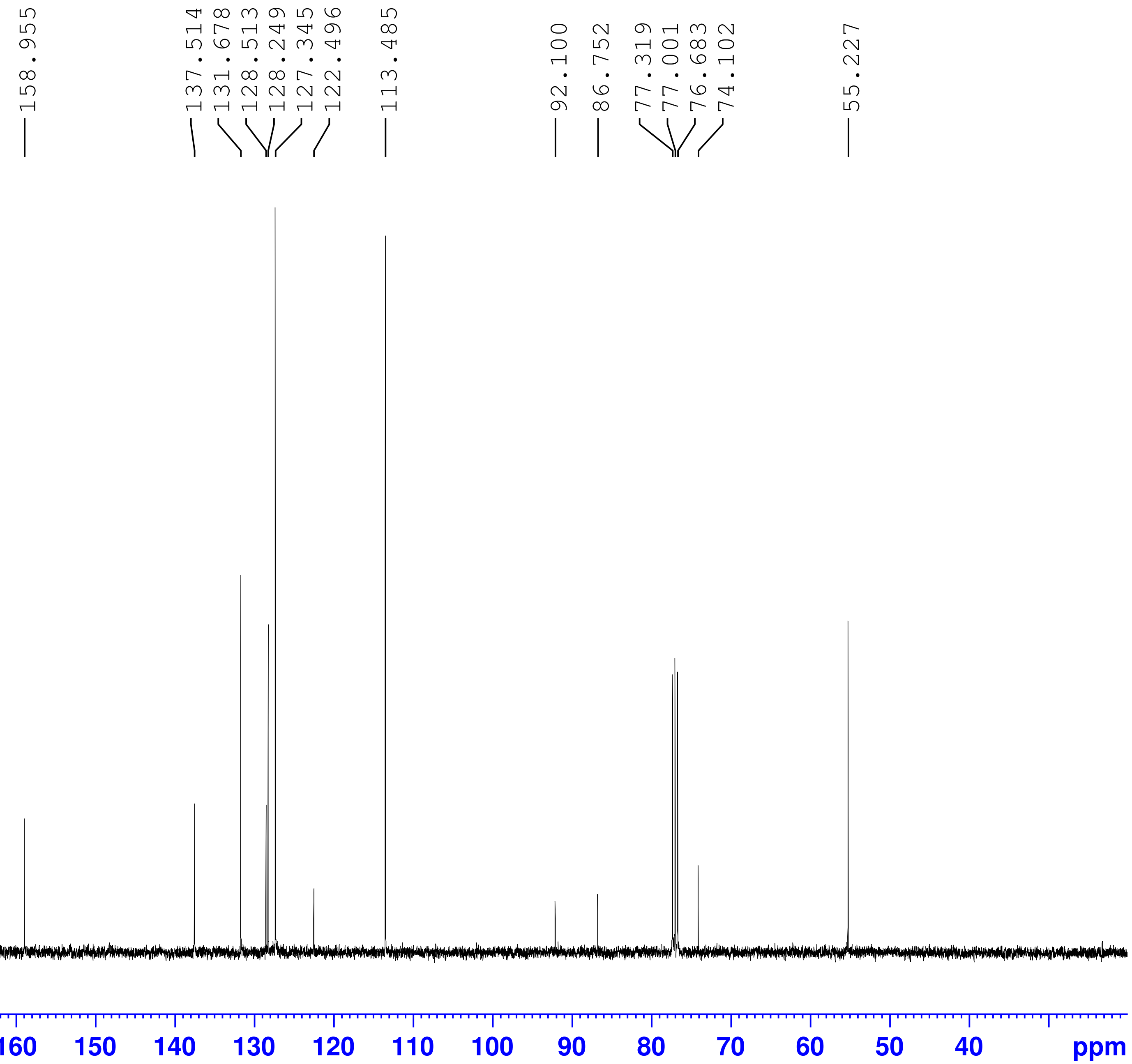


```
Current Data Parameters
NAME          qdy-30184 C
EXPNO                   2
PROCNO                  1

F2 - Acquisition Parameters
Date_            20170116
Time                14.12
INSTRUM             spect
PROBHD   5 mm PABBO BB/
PULPROG           zgpg30
TD                 65536
SOLVENT            CDCl3
NS                    60
DS                     0
SWH            24038.461 Hz
FIDRES          0.366798 Hz
AQ             1.3631488 sec
RG                196.92
DW                20.800 usec
DE                  6.50 usec
TE                 296.3 K
D1            2.00000000 sec
D11           0.03000000 sec
TD0                    1

======== CHANNEL f1 ========
SFO1         100.6228298 MHz
NUC1                 13C
P1                  9.70 usec
PLW1         46.98899841 W

======== CHANNEL f2 ========
SFO2         400.1316005 MHz
NUC2                  1H
CPDPRG[2         waltz16
PCPD2              90.00 usec
PLW2         11.99499989 W
PLW12         0.34213999 W
PLW13         0.27713001 W

F2 - Processing parameters
SI                 32768
SF           100.6127788 MHz
WDW                   EM
SSB      0
LB                  1.00 Hz
GB       0
PC                  1.40
```

Supplementary Figure 12. ^{13}C NMR spectrum for 1f

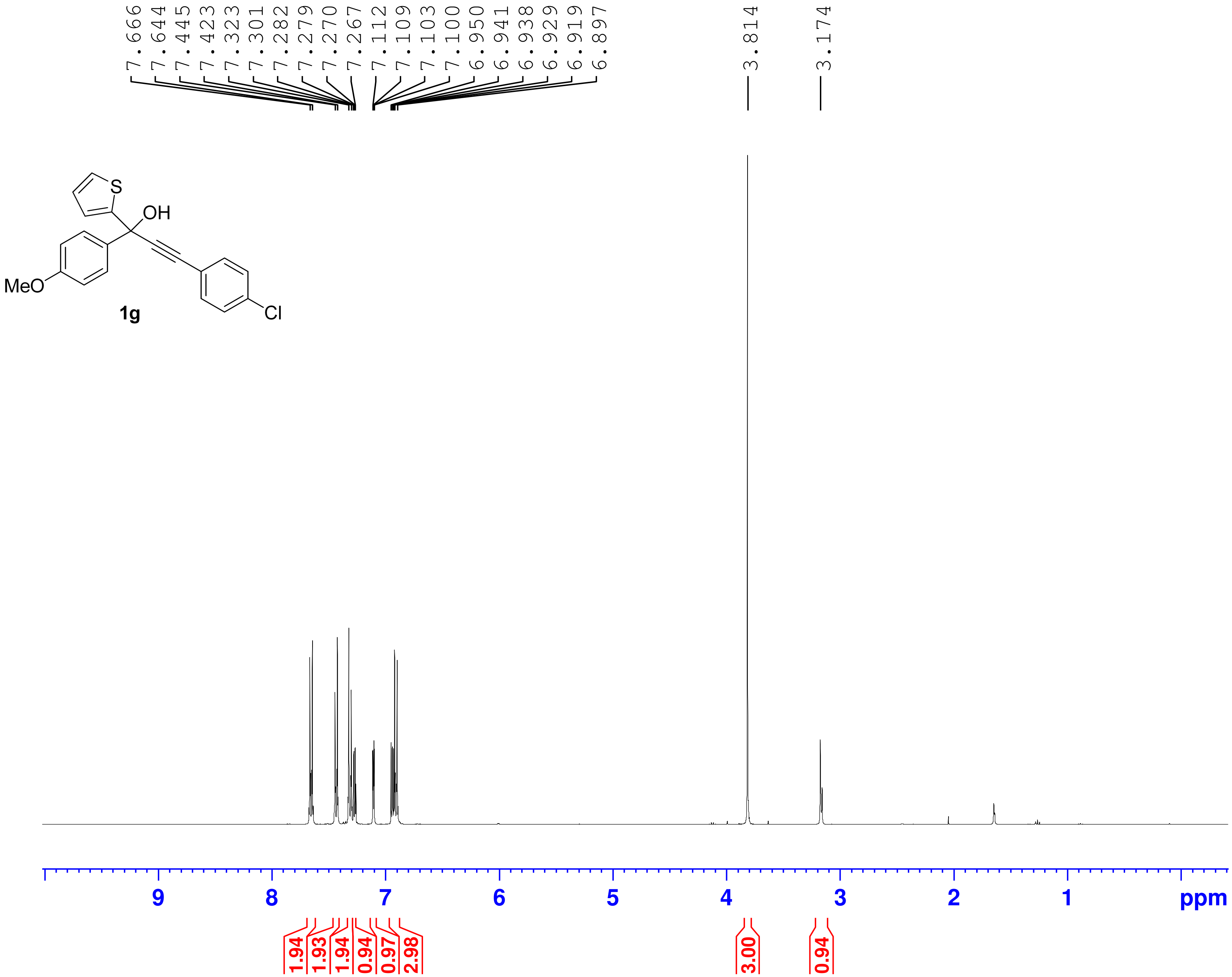

Current Data Parameters
NAME qdy-40012-1 H
EXPNO 2
PROCNO 1

F2 - Acquisition Parameters
Date_ 20161216
Time 17.20
INSTRUM spect
PROBHD 5 mm PABBO BB/
PULPROG zg30
TD 65536
SOLVENT CDCl3
NS 4
DS 0
SWH 8012.820 Hz
FIDRES 0.122266 Hz
AQ 4.0894465 sec
RG 103.52
DW 62.400 usec
DE 6.50 usec
TE 294.6 K
D1 1.00000000 sec
TD0 1

======== CHANNEL f1 ========
SFO1 400.1324710 MHz
NUC1 1H
P1 14.50 usec
PLW1 11.99499989 W

F2 - Processing parameters
SI 65536
SF 400.1300092 MHz
WDW EM
SSB 0
LB 0.30 Hz
GB 0
PC 1.00

Supplementary Figure 13. ^{1}H NMR spectrum for 1g

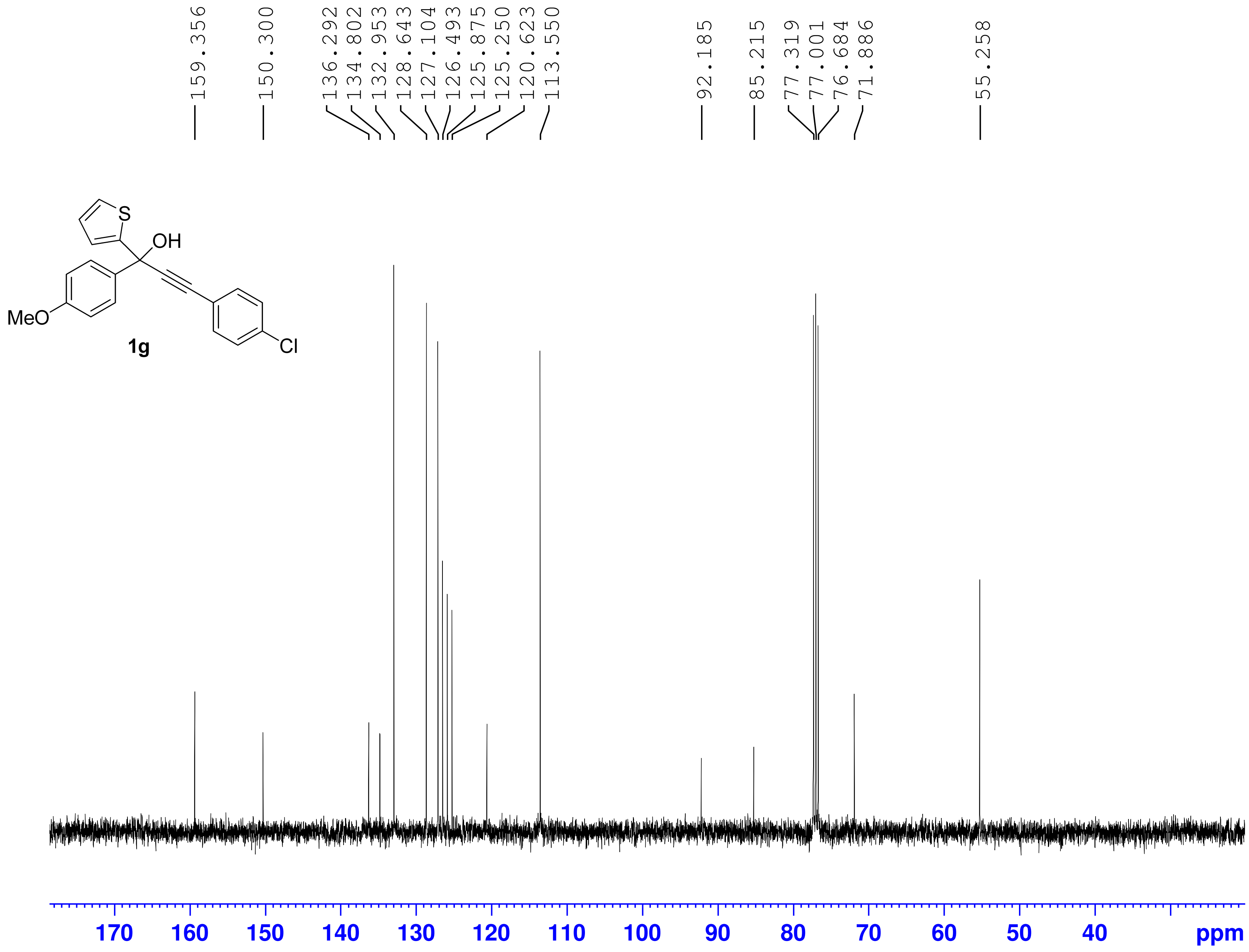

Current Data Parameters
NAME qdy-40012-1 C
EXPNO 1
PROCNO 1

F2 - Acquisition Parameters
Date_ 20161214
Time 21.57
INSTRUM spect
PROBHD 5 mm DUL 13C-1
PULPROG zgpg30
TD 65536
SOLVENT CDCl3
NS 46
DS 0
SWH 24038.461 Hz
FIDRES 0.366798 Hz
AQ 1.3631488 sec
RG 196.92
DW 20.800 usec
DE 6.50 usec
TE 297.1 K
D1 2.00000000 sec
D11 0.03000000 sec
TD0 1

======== CHANNEL f1 ========
SFO1 100.6228298 MHz
NUC1 13C
P1 9.60 usec
PLW1 31.98900032 W

======== CHANNEL f2 ========
SFO2 400.1316005 MHz
NUC2 1H
CPDPRG[2 waltz16
PCPD2 90.00 usec
PLW2 9.10000038 W
PLW12 0.24608000 W
PLW13 0.19933000 W

F2 - Processing parameters
SI 32768
SF 100.6127780 MHz
WDW EM
SSB 0
LB 1.00 Hz
GB 0
PC 1.40

Supplementary Figure 14. ^{13}C NMR spectrum for 1g

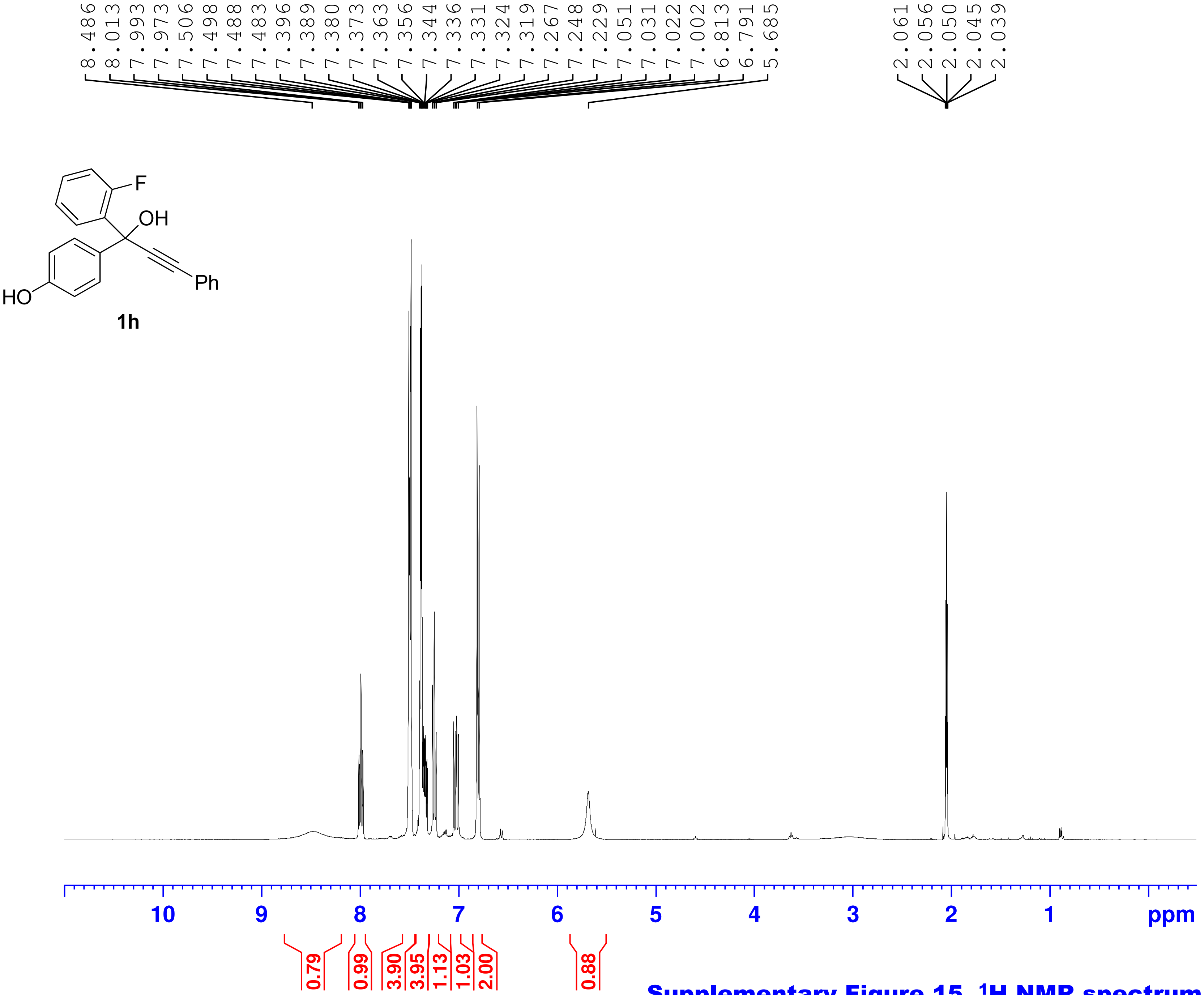


```
Current Data Parameters
NAME          qdy-20089 H
EXPNO                   1
PROCNO                  1

F2 - Acquisition Parameters
Date_            20160324
Time                19.47
INSTRUM             spect
PROBHD    5 mm PABBO BB/
PULPROG              zg30
TD                  65536
SOLVENT           Acetone
NS                     16
DS                      0
SWH              8012.820 Hz
FIDRES           0.122266 Hz
AQ              4.0894465 sec
RG                  70.97
DW                 62.400 usec
DE                   6.50 usec
TE                  296.6 K
D1             1.00000000 sec
TD0                     1

======== CHANNEL f1 ========
SFO1          400.1324710 MHz
NUC1                   1H
P1                  14.50 usec
PLW1          11.99499989 W

F2 - Processing parameters
SI                  65536
SF            400.1300067 MHz
WDW                    EM
SSB       0
LB                   0.30 Hz
GB        0
PC                   1.00
```

Supplementary Figure 15. ^{1}H NMR spectrum for 1h

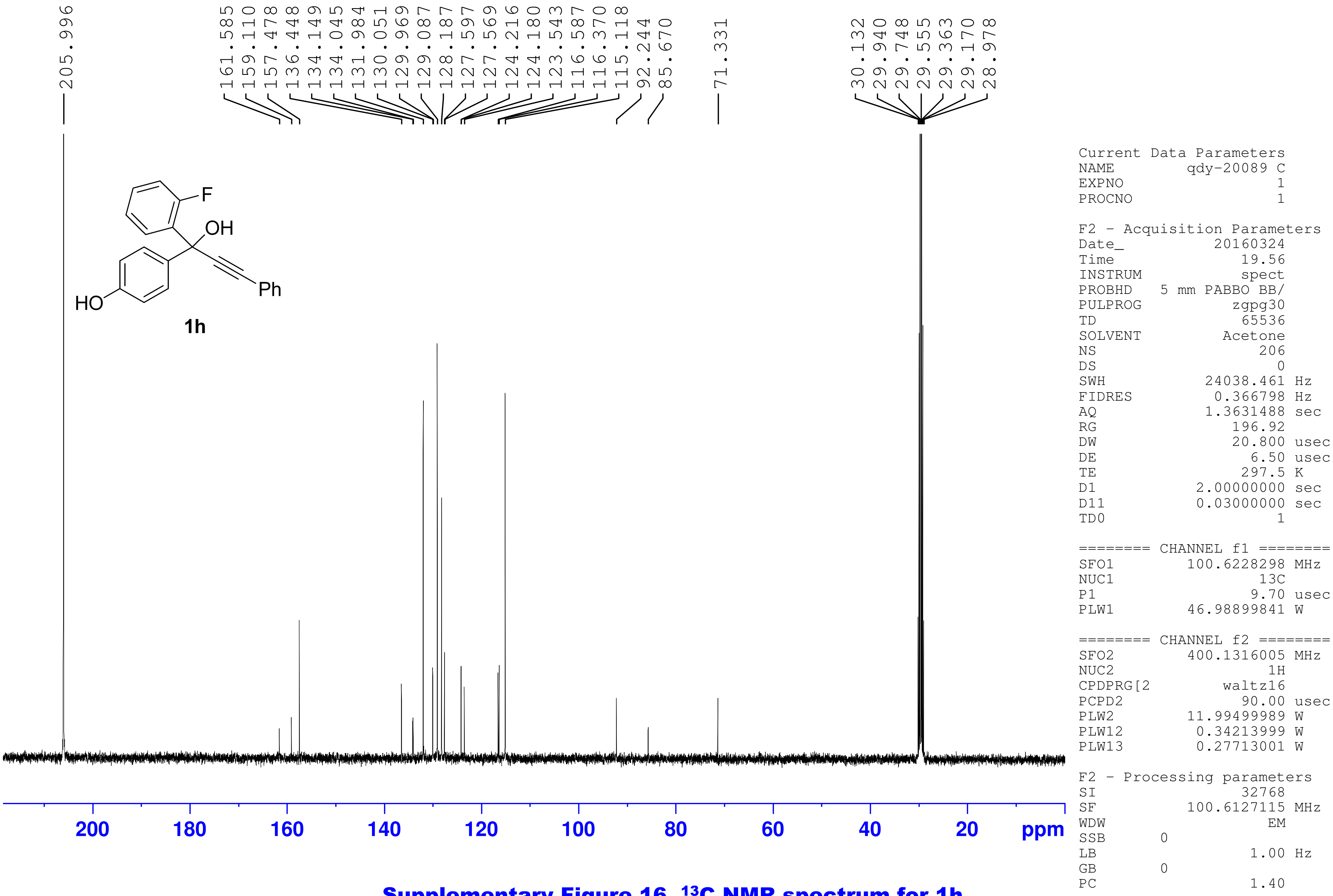

Supplementary Figure 16. ^{13}C NMR spectrum for 1h

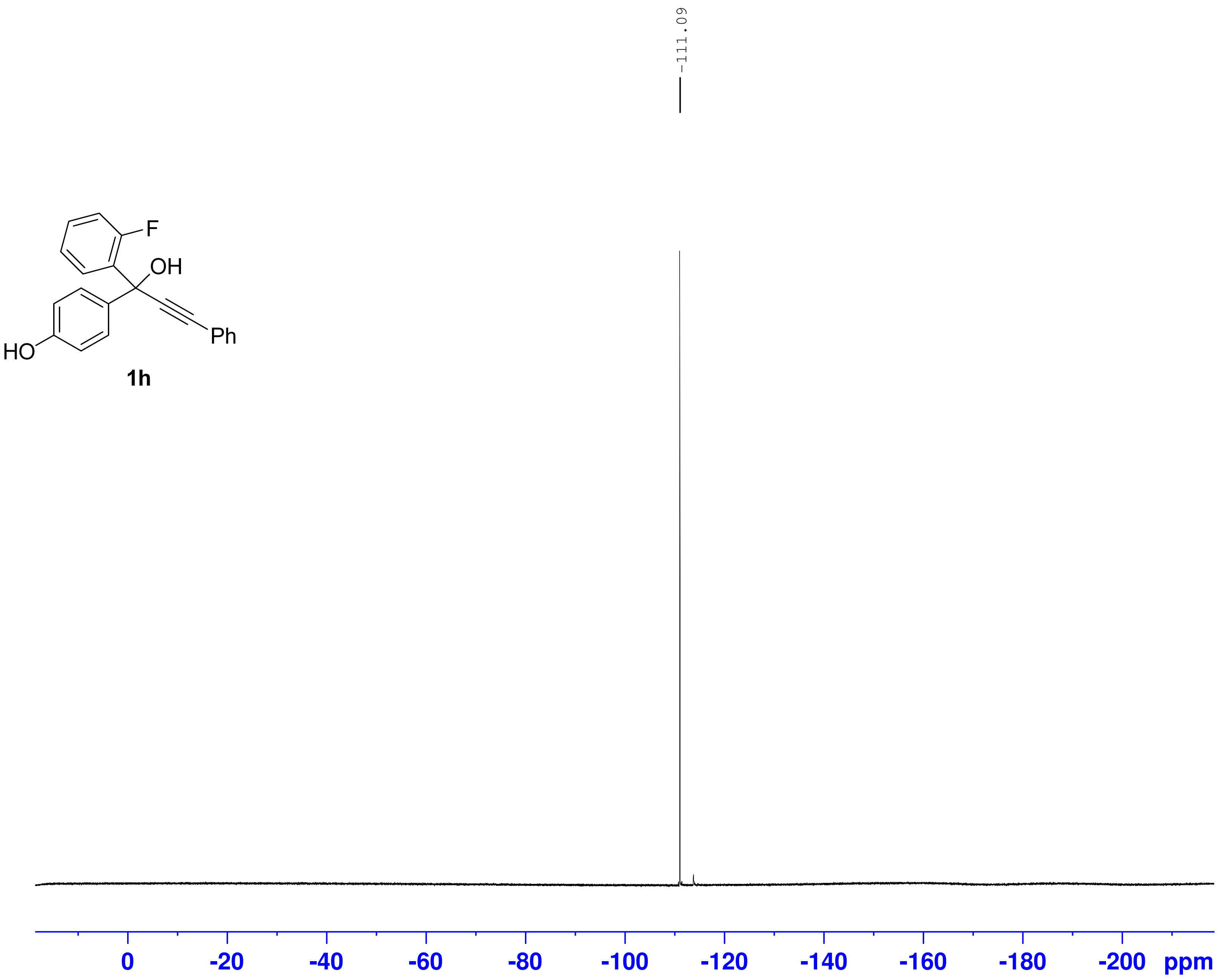


```
Current Data Parameters
NAME          qdy-20089 F
EXPNO                   1
PROCNO                  1

F2 - Acquisition Parameters
Date_            20160324
Time                19.49
INSTRUM             spect
PROBHD   5 mm PABBO BB/
PULPROG            zgflqn
TD                 131072
SOLVENT           Acetone
NS                     16
DS                      0
SWH            89285.711 Hz
FIDRES          0.681196 Hz
AQ             0.7340032 sec
RG                 196.92
DW                  5.600 usec
DE                   6.50 usec
TE                  296.5 K
D1             1.00000000 sec
TD0                     1

======== CHANNEL f1 ========
SFO1         376.4607164 MHz
NUC1                  19F
P1                  14.70 usec
PLW1         15.99600029 W

F2 - Processing parameters
SI                  65536
SF           376.4983660 MHz
WDW                    EM
SSB      0
LB                   0.30 Hz
GB       0
PC                   1.00
```

Supplementary Figure 17. ^{19}F NMR spectrum for 1h

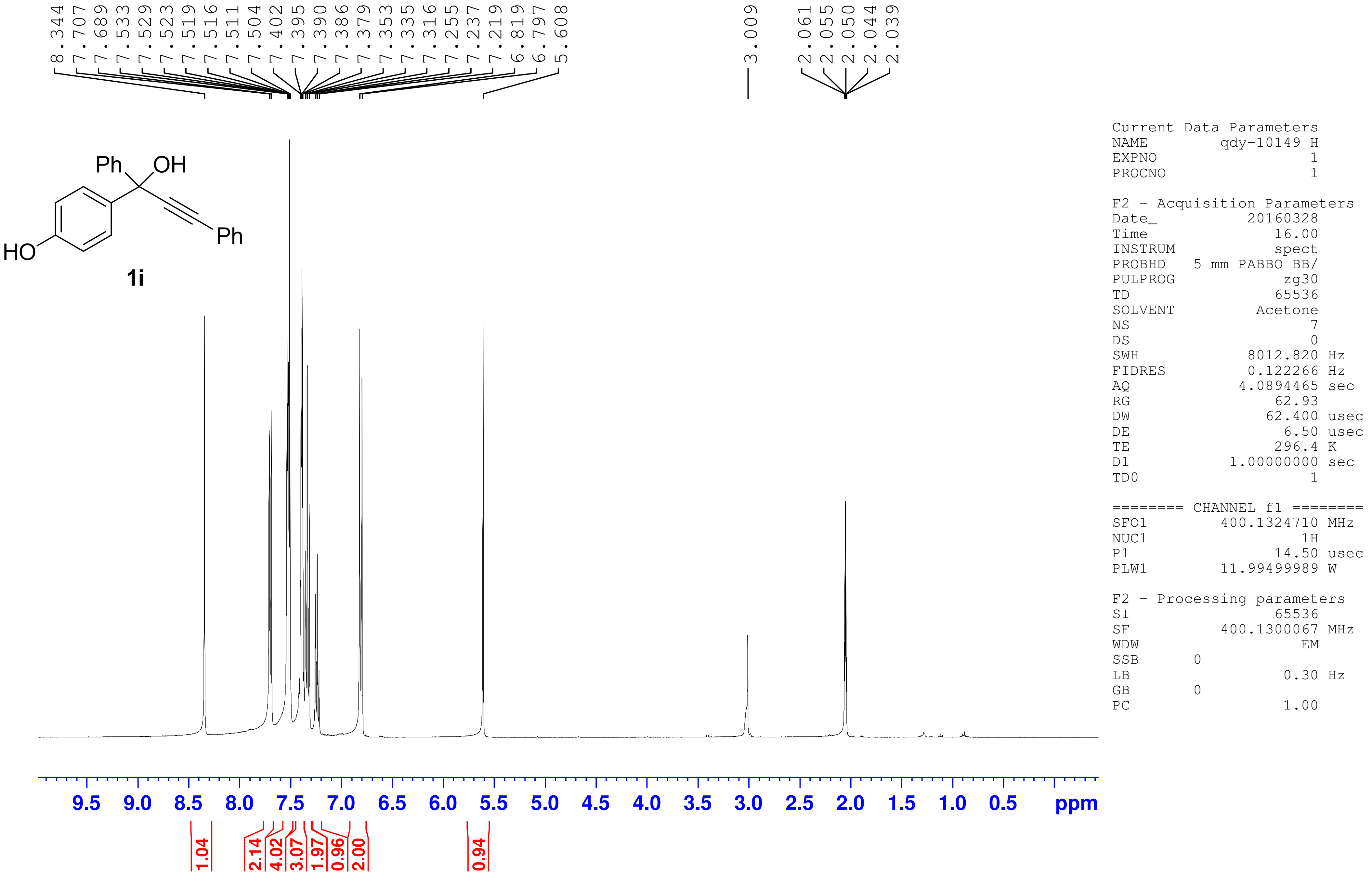

Supplementary Figure 18. ^{1}H NMR spectrum for 1i

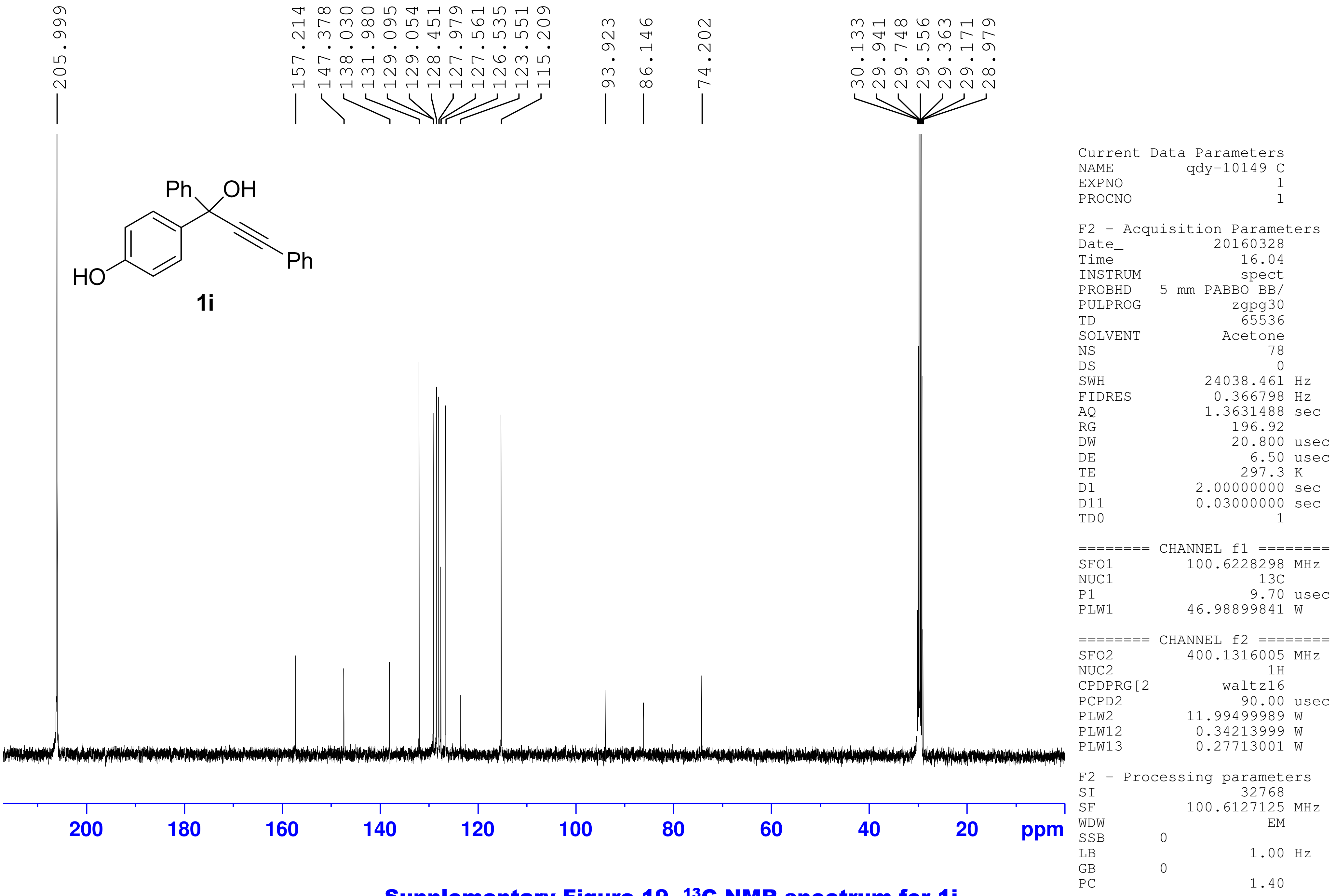

Supplementary Figure 19. ^{13}C NMR spectrum for 1i

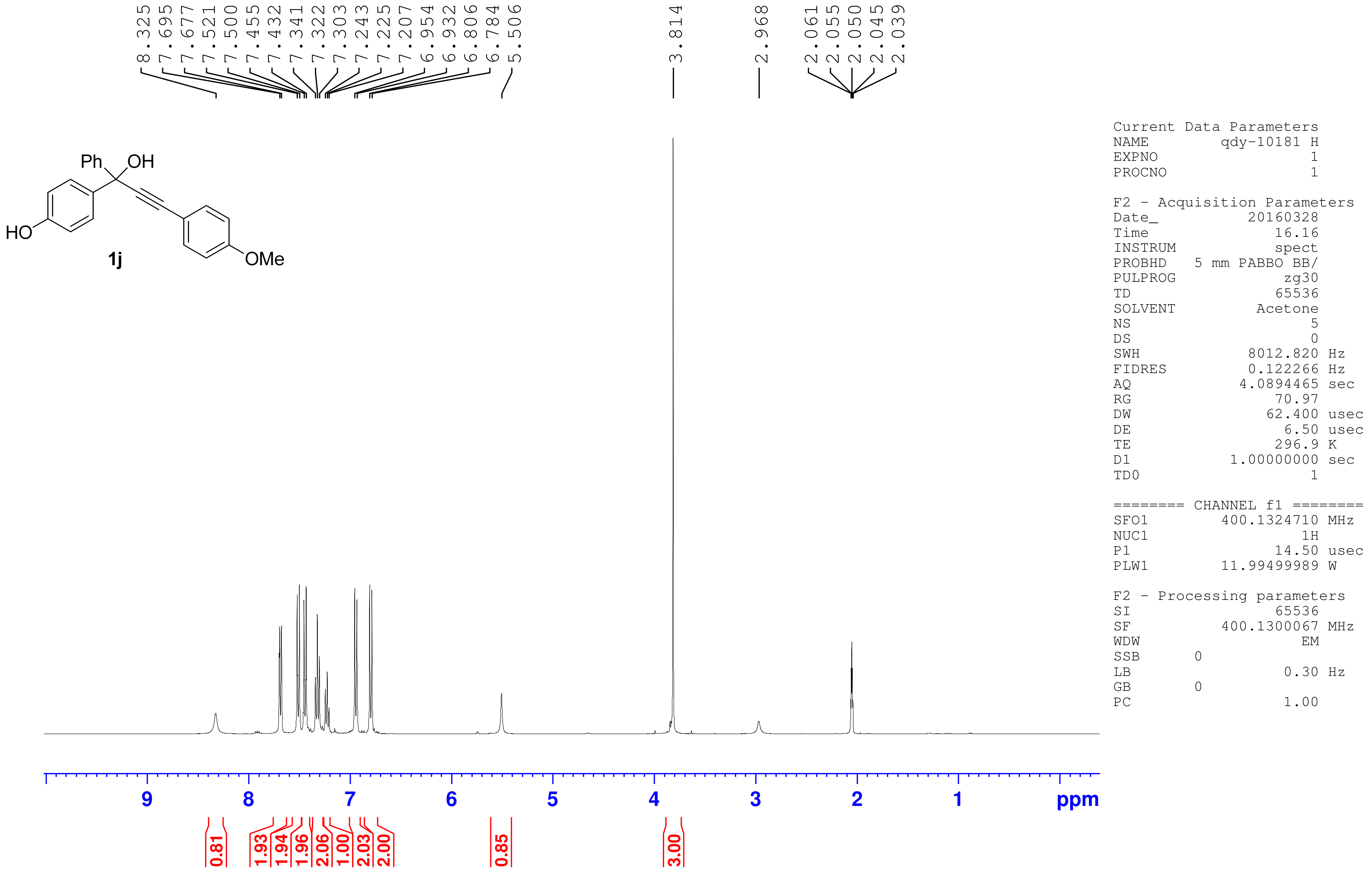

Supplementary Figure 20. ^{1}H NMR spectrum for 1j

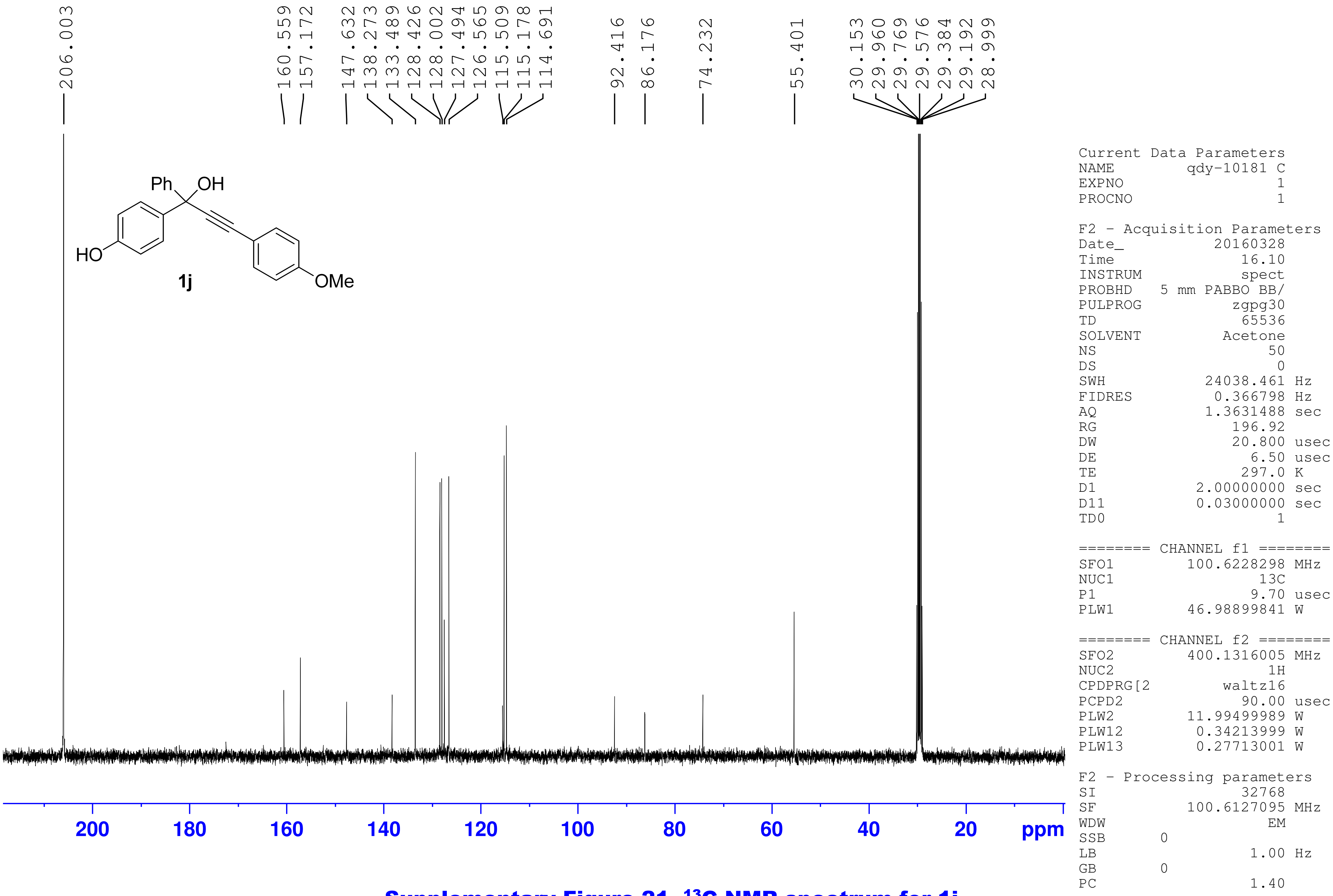

Supplementary Figure 21. ^{13}C NMR spectrum for 1j

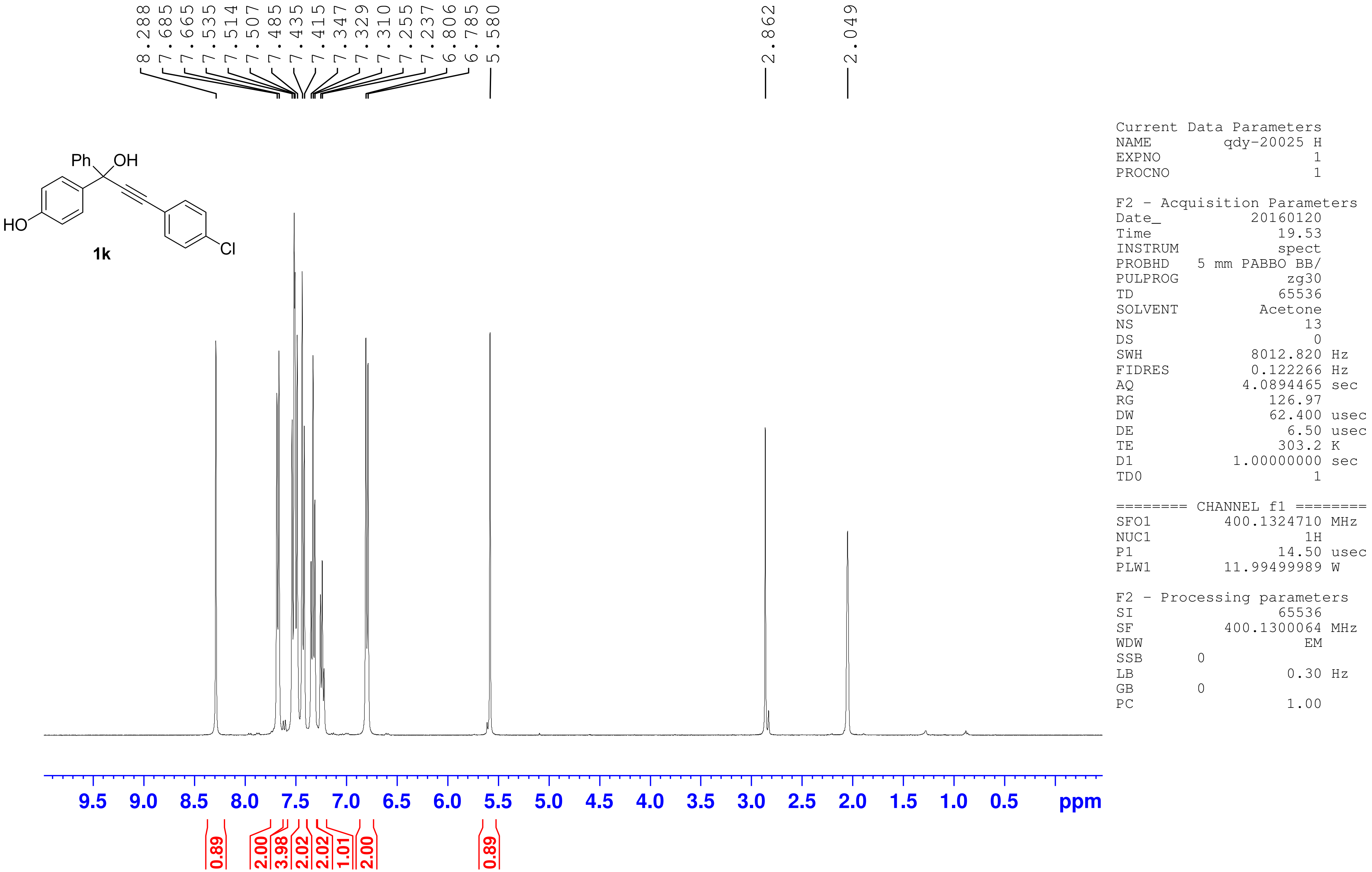

Supplementary Figure 22. ^{1}H NMR spectrum for 1k

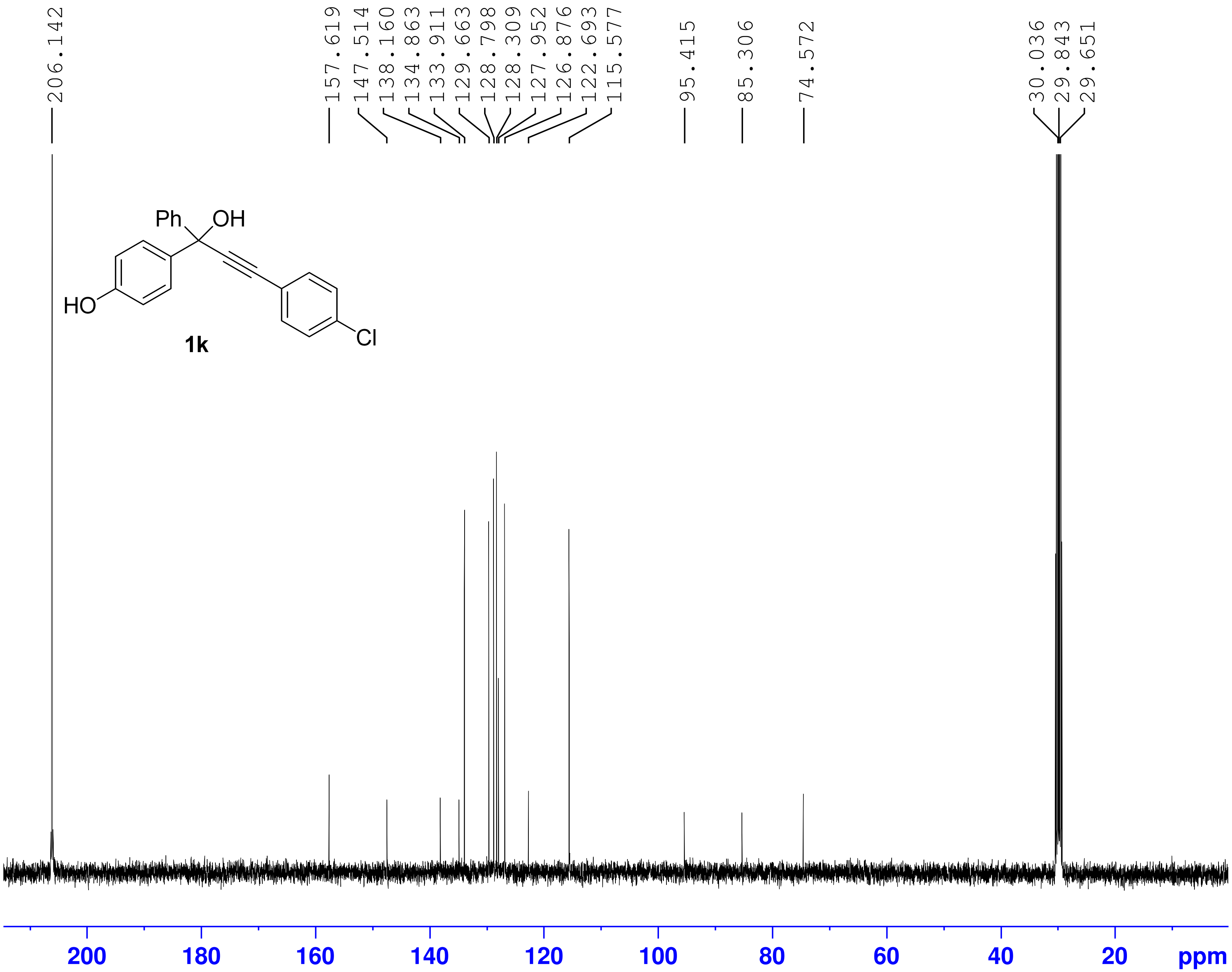

Current Data Parameters
NAME qdy-20025 C
EXPNO 1
PROCNO 1

F2 - Acquisition Parameters
Date_ 20160120
Time 19.58
INSTRUM spect
PROBHD 5 mm PABBO BB/
PULPROG zgpg30
TD 65536
SOLVENT Acetone
NS 101
DS 2
SWH 24038.461 Hz
FIDRES 0.366798 Hz
AQ 1.3631488 sec
RG 196.92
DW 20.800 usec
DE 6.50 usec
TE 303.5 K
D1 2.00000000 sec
D11 0.03000000 sec
TD0 1

======== CHANNEL f1 ========
SFO1 100.6228298 MHz
NUC1 13C
P1 9.70 usec
PLW1 46.98899841 W

======== CHANNEL f2 ========
SFO2 400.1316005 MHz
NUC2 1H
CPDPRG[2 waltz16
PCPD2 90.00 usec
PLW2 11.99499989 W
PLW12 0.34213999 W
PLW13 0.27713001 W

F2 - Processing parameters
SI 32768
SF 100.6126768 MHz
WDW EM
SSB 0
LB 1.00 Hz
GB 0
PC 1.40

Supplementary Figure 23. ^{13}C NMR spectrum for 1k

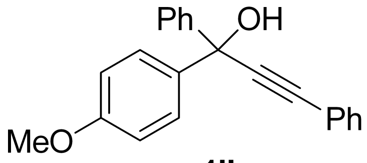

1i'

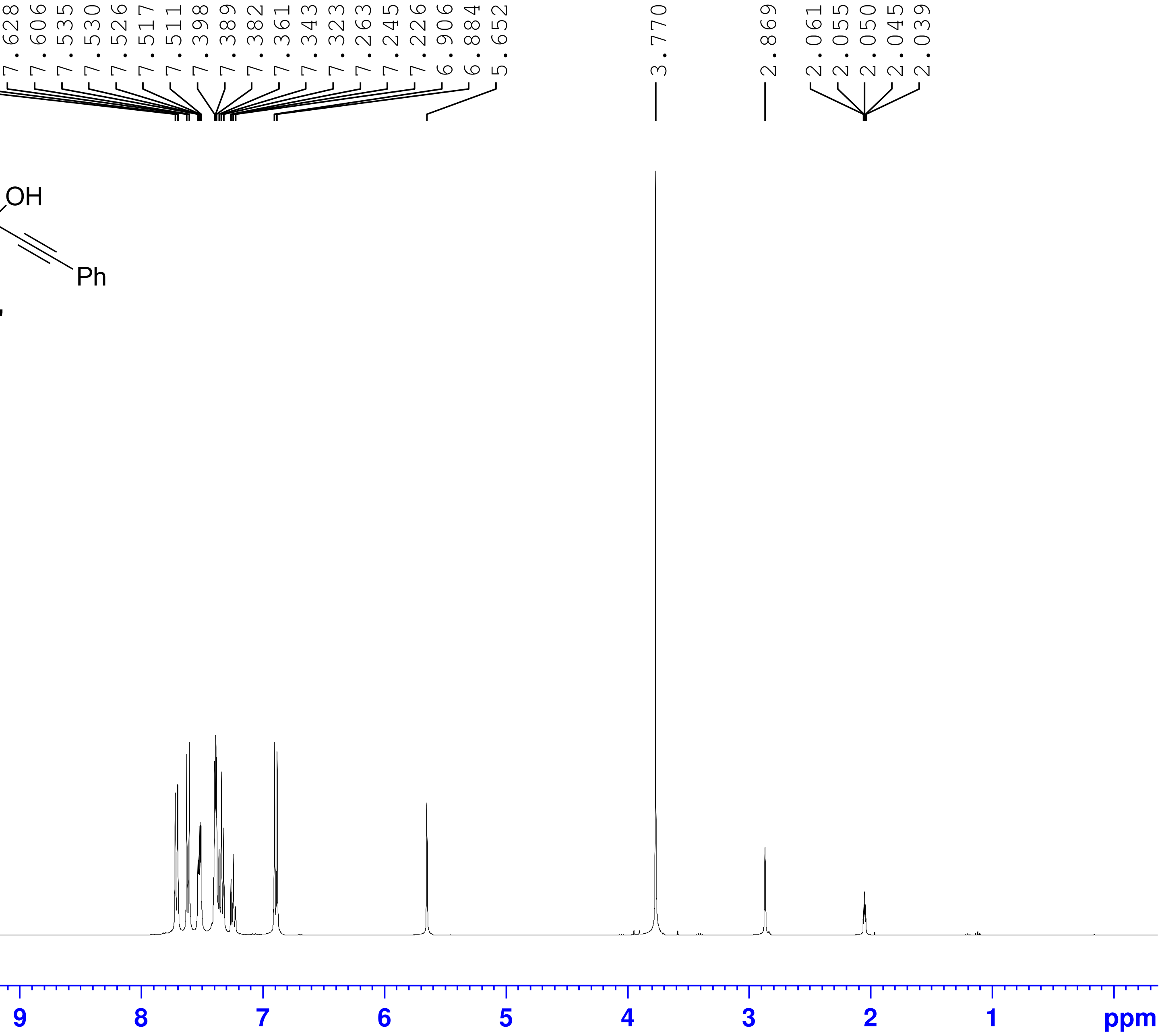


```
Current Data Parameters
NAME          qdy-10173 H
EXPNO                   1
PROCNO                  1

F2 - Acquisition Parameters
Date_            20160529
Time                21.08
INSTRUM             spect
PROBHD    5 mm PABBO BB/
PULPROG              zg30
TD                  65536
SOLVENT           Acetone
NS                      8
DS                      0
SWH              8012.820 Hz
FIDRES           0.122266 Hz
AQ              4.0894465 sec
RG                  31.55
DW                 62.400 usec
DE                   6.50 usec
TE                  300.4 K
D1             1.00000000 sec
TD0                     1

======== CHANNEL f1 ========
SFO1          400.1324710 MHz
NUC1                   1H
P1                  14.50 usec
PLW1          11.99499989 W

F2 - Processing parameters
SI                  65536
SF            400.1300066 MHz
WDW                    EM
SSB       0
LB                   0.30 Hz
GB        0
PC                   1.00
```

Supplementary Figure 24. ^{1}H NMR spectrum for 1i'

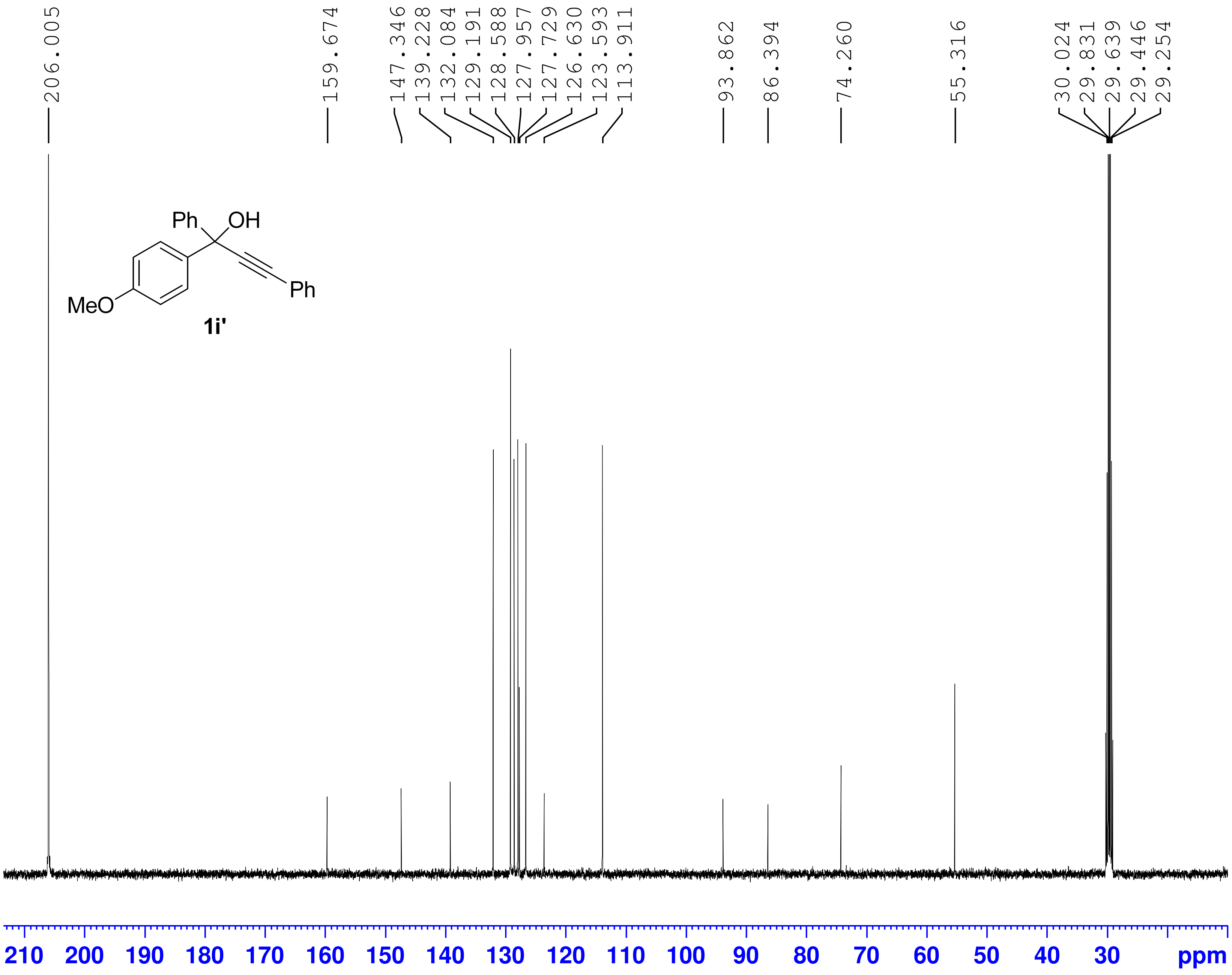

Current Data Parameters
NAME qdy-10173 C
EXPNO 1
PROCNO 1

F2 - Acquisition Parameters
Date_ 20160529
Time 21.02
INSTRUM spect
PROBHD 5 mm PABBO BB/
PULPROG zgpg30
TD 65536
SOLVENT Acetone
NS 106
DS 0
SWH 24038.461 Hz
FIDRES 0.366798 Hz
AQ 1.3631488 sec
RG 196.92
DW 20.800 usec
DE 6.50 usec
TE 300.6 K
D1 2.00000000 sec
D11 0.03000000 sec
TD0 1

======== CHANNEL f1 ========
SFO1 100.6228298 MHz
NUC1 13C
P1 9.70 usec
PLW1 46.98899841 W

======== CHANNEL f2 ========
SFO2 400.1316005 MHz
NUC2 1H
CPDPRG[2 waltz16
PCPD2 90.00 usec
PLW2 11.99499989 W
PLW12 0.34213999 W
PLW13 0.27713001 W

F2 - Processing parameters
SI 32768
SF 100.6127034 MHz
WDW EM
SSB 0
LB 1.00 Hz
GB 0
PC 1.40

Supplementary Figure 25. ^{13}C NMR spectrum for 1i'

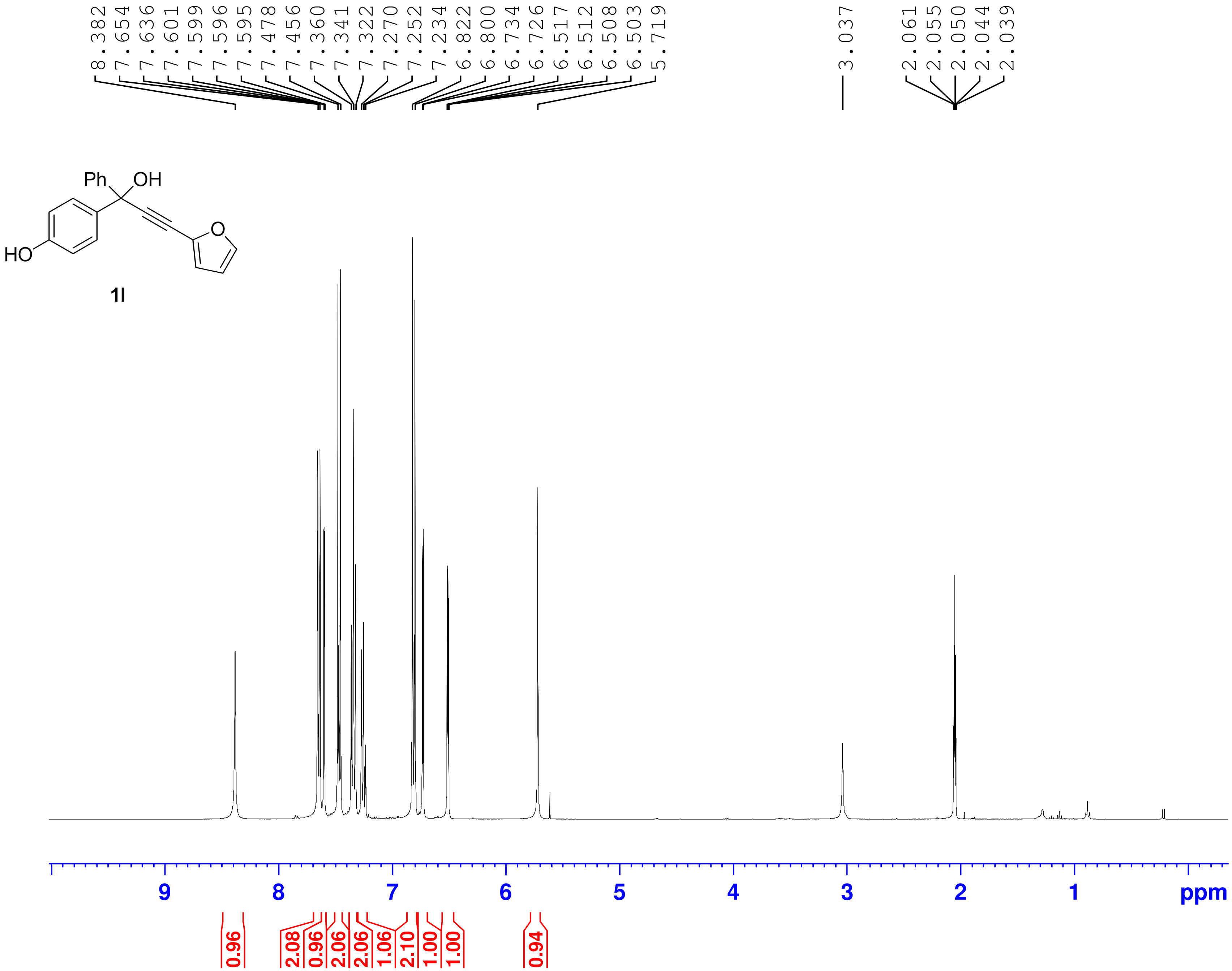

1l

Current Data Parameters
NAME qdy-wcw-1019 H
EXPNO 3
PROCNO 1

F2 - Acquisition Parameters
Date_ 20160326
Time 14.03
INSTRUM spect
PROBHD 5 mm PABBO BB/
PULPROG zg30
TD 65536
SOLVENT Acetone
NS 16
DS 0
SWH 8012.820 Hz
FIDRES 0.122266 Hz
AQ 4.0894465 sec
RG 54.81
DW 62.400 usec
DE 6.50 usec
TE 296.8 K
D1 1.00000000 sec
TD0 1

======== CHANNEL f1 ========
SFO1 400.1324710 MHz
NUC1 1H
P1 14.50 usec
PLW1 11.99499989 W

F2 - Processing parameters
SI 65536
SF 400.1300066 MHz
WDW EM
SSB 0
LB 0.30 Hz
GB 0
PC 1.00

Supplementary Figure 26. ^{1}H NMR spectrum for 1l

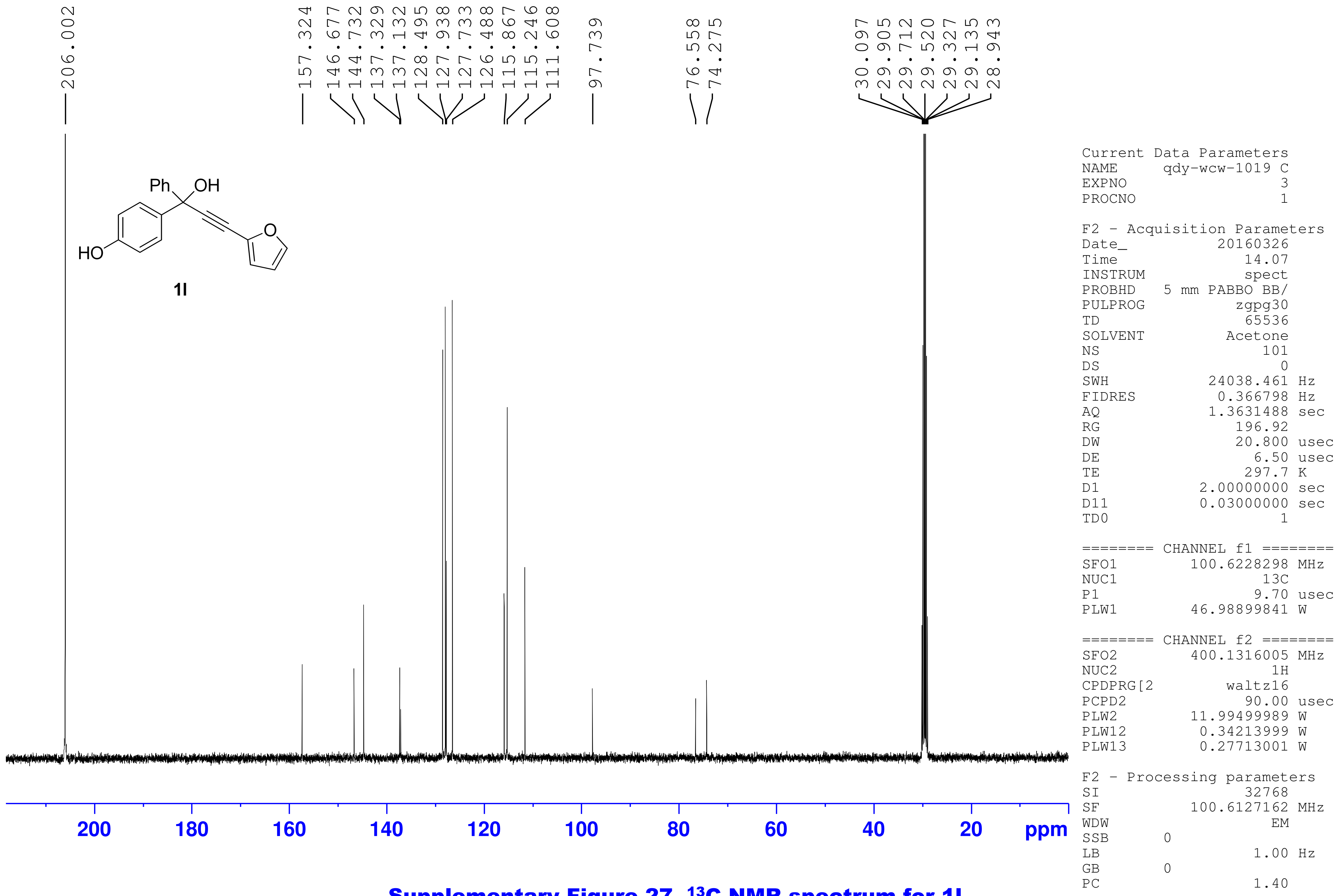

Supplementary Figure 27. ^{13}C NMR spectrum for 1l

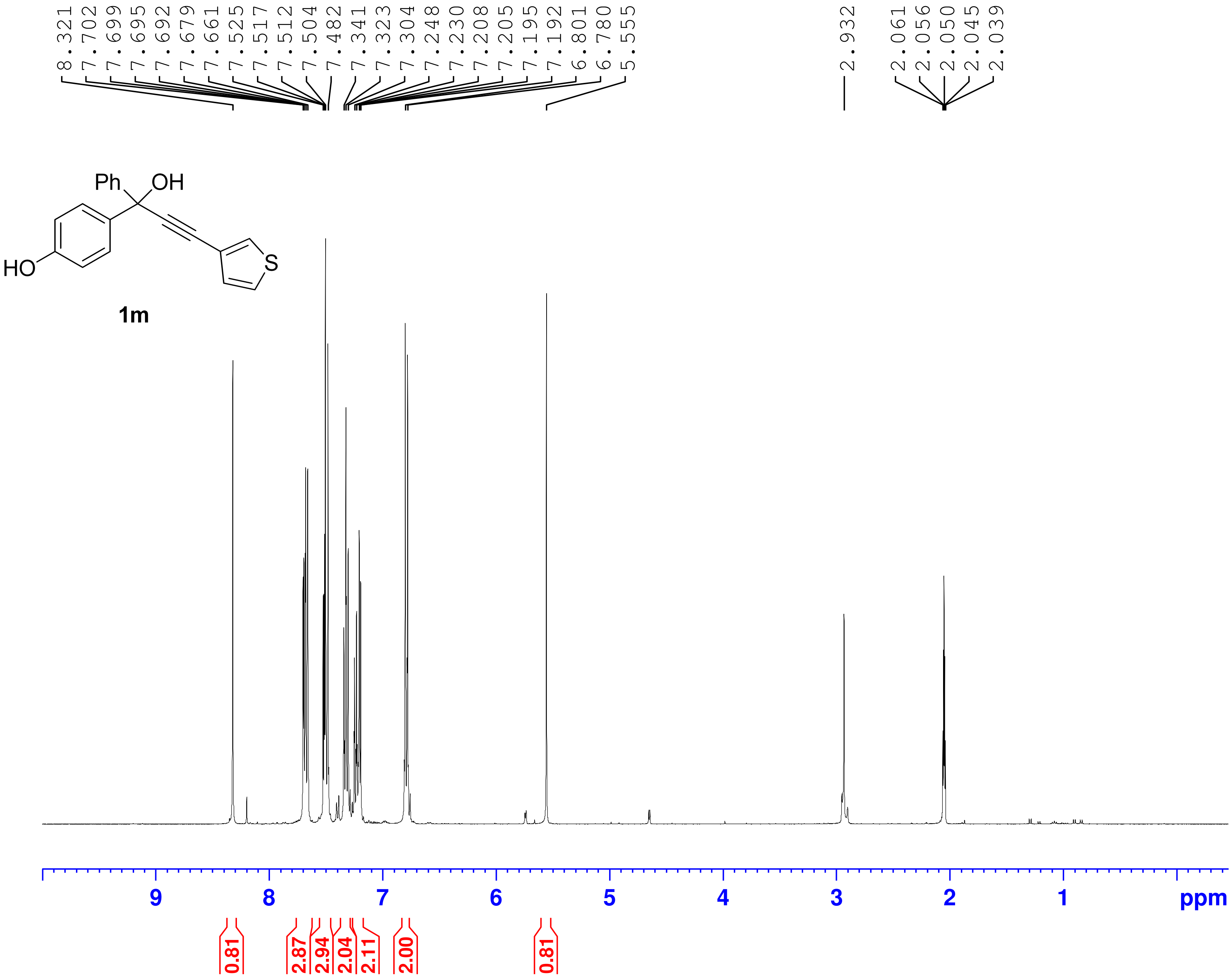

1m


```
Current Data Parameters
NAME          qdy-20058 H
EXPNO                   2
PROCNO                  1

F2 - Acquisition Parameters
Date_            20160227
Time                21.45
INSTRUM             spect
PROBHD   5 mm PABBO BB/
PULPROG              zg30
TD                  65536
SOLVENT           Acetone
NS                      6
DS                      0
SWH              8012.820 Hz
FIDRES           0.122266 Hz
AQ              4.0894465 sec
RG                 112.31
DW                 62.400 usec
DE                   6.50 usec
TE                  297.3 K
D1             1.00000000 sec
TD0                     1

======== CHANNEL f1 ========
SFO1         400.1324710 MHz
NUC1                   1H
P1                  14.50 usec
PLW1          11.99499989 W

F2 - Processing parameters
SI                  65536
SF            400.1300067 MHz
WDW                    EM
SSB      0
LB                   0.30 Hz
GB       0
PC                   1.00
```

Supplementary Figure 28. ^{1}H NMR spectrum for 1m

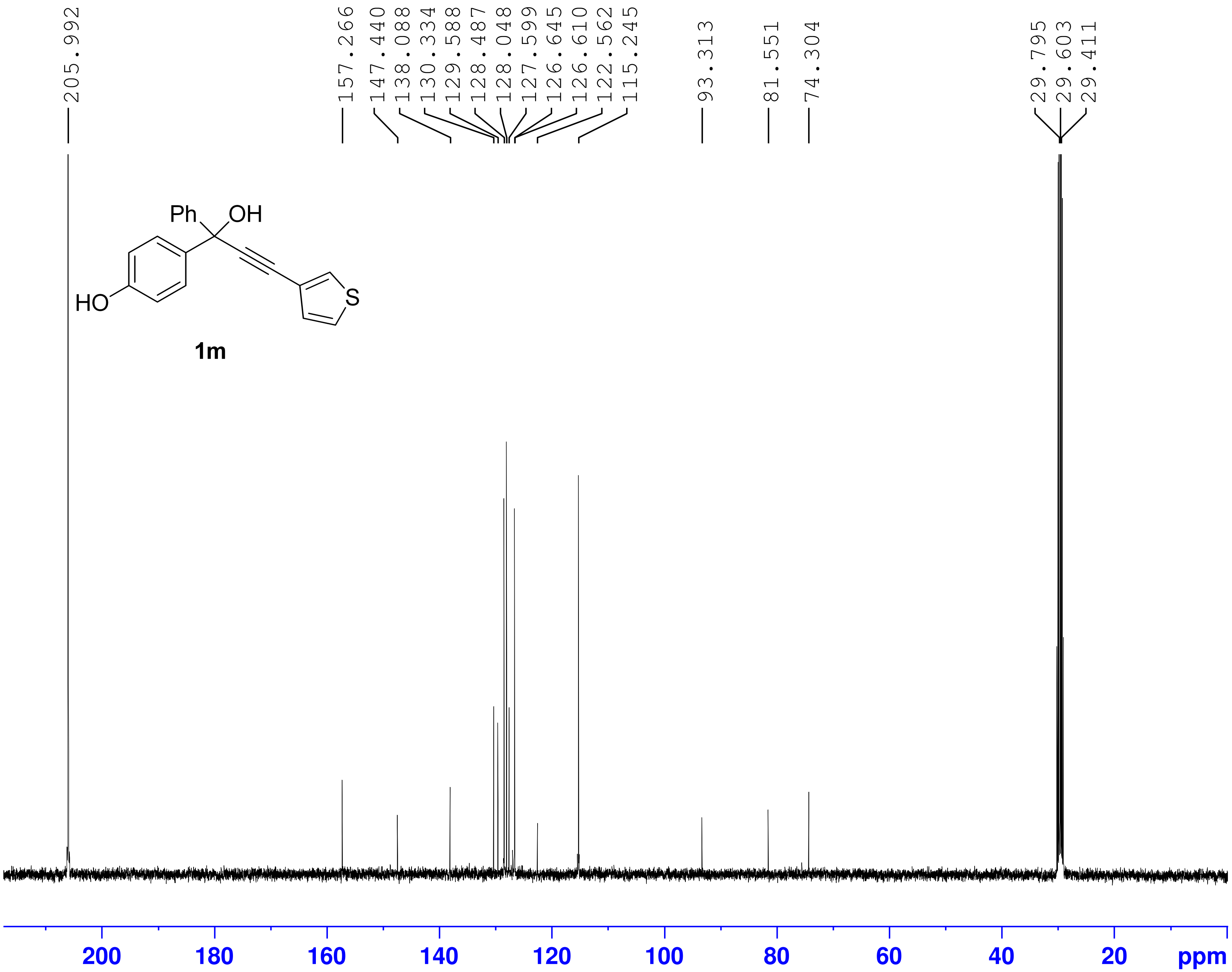

Current Data Parameters
NAME qdy-20058 C
EXPNO 1
PROCNO 1

F2 - Acquisition Parameters
Date_ 20160227
Time 16.55
INSTRUM spect
PROBHD 5 mm PABBO BB/
PULPROG zgpg30
TD 65536
SOLVENT Acetone
NS 159
DS 0
SWH 24038.461 Hz
FIDRES 0.366798 Hz
AQ 1.3631488 sec
RG 196.92
DW 20.800 usec
DE 6.50 usec
TE 297.5 K
D1 2.00000000 sec
D11 0.03000000 sec
TD0 1

======== CHANNEL f1 ========
SFO1 100.6228298 MHz
NUC1 13C
P1 9.70 usec
PLW1 46.98899841 W

======== CHANNEL f2 ========
SFO2 400.1316005 MHz
NUC2 1H
CPDPRG[2 waltz16
PCPD2 90.00 usec
PLW2 11.99499989 W
PLW12 0.34213999 W
PLW13 0.27713001 W

F2 - Processing parameters
SI 32768
SF 100.6127052 MHz
WDW EM
SSB 0
LB 1.00 Hz
GB 0
PC 1.40

Supplementary Figure 29. ^{13}C NMR spectrum for 1m

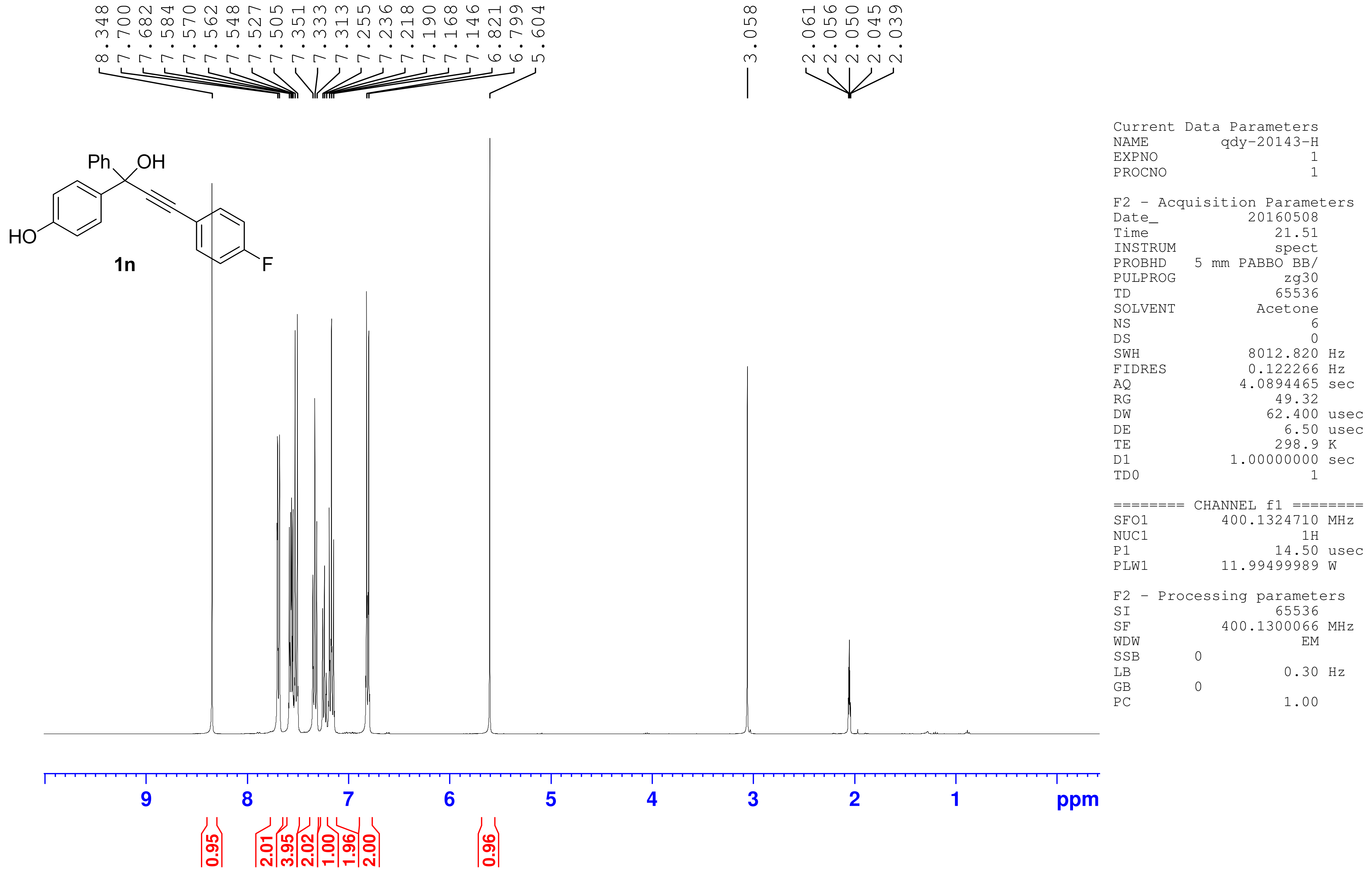

Supplementary Figure 30. ^{1}H NMR spectrum for 1n

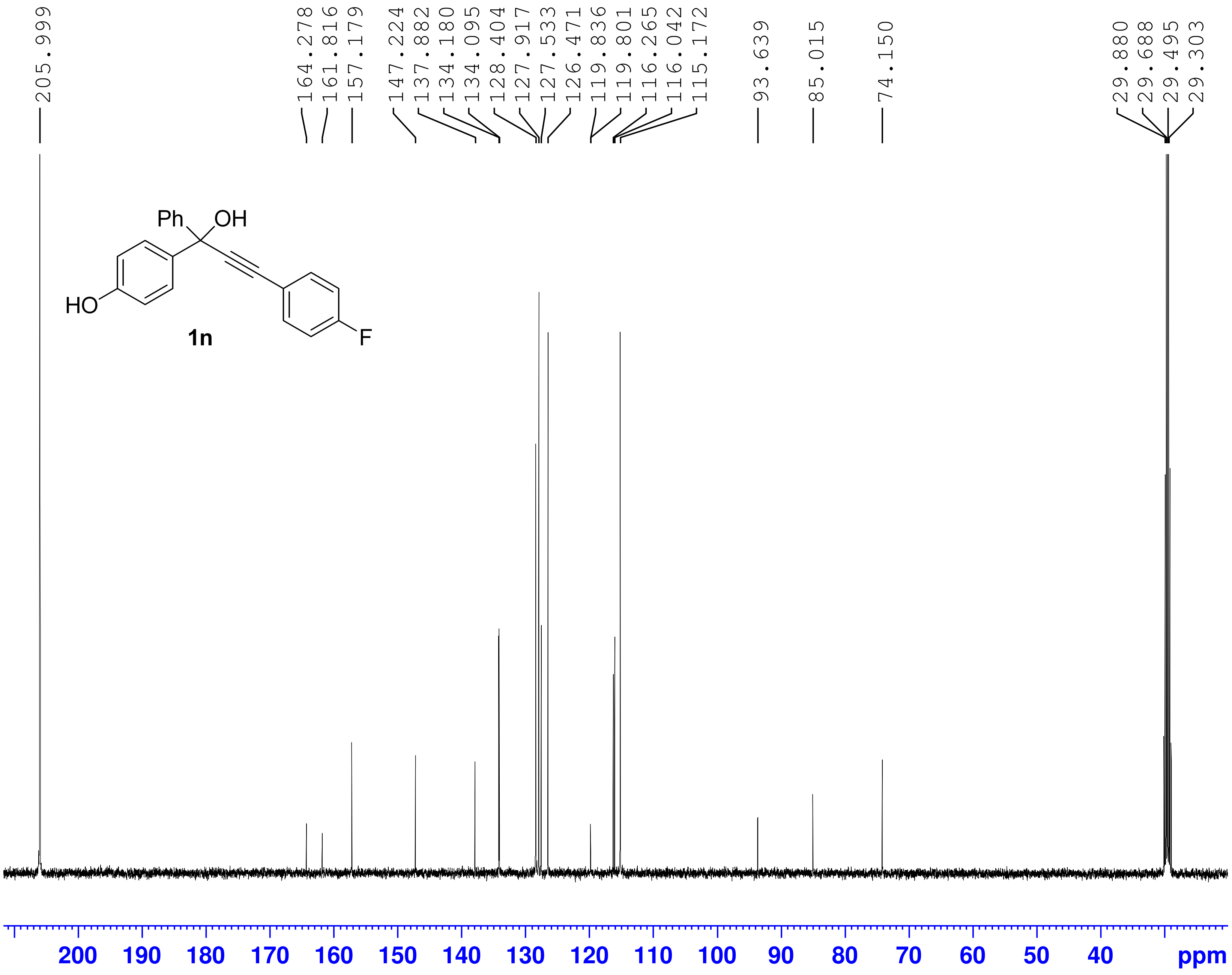

Current Data Parameters
NAME qdy-20143-C
EXPNO 1
PROCNO 1

F2 - Acquisition Parameters
Date_ 20160508
Time 21.54
INSTRUM spect
PROBHD 5 mm PABBO BB/
PULPROG zgpg30
TD 65536
SOLVENT Acetone
NS 80
DS 0
SWH 24038.461 Hz
FIDRES 0.366798 Hz
AQ 1.3631488 sec
RG 196.92
DW 20.800 usec
DE 6.50 usec
TE 299.7 K
D1 2.00000000 sec
D11 0.03000000 sec
TD0 1

======== CHANNEL f1 ========
SFO1 100.6228298 MHz
NUC1 13C
P1 9.70 usec
PLW1 46.98899841 W

======== CHANNEL f2 ========
SFO2 400.1316005 MHz
NUC2 1H
CPDPRG[2 waltz16
PCPD2 90.00 usec
PLW2 11.99499989 W
PLW12 0.34213999 W
PLW13 0.27713001 W

F2 - Processing parameters
SI 32768
SF 100.6127184 MHz
WDW EM
SSB 0
LB 1.00 Hz
GB 0
PC 1.40

Supplementary Figure 31. ^{13}C NMR spectrum for 1n

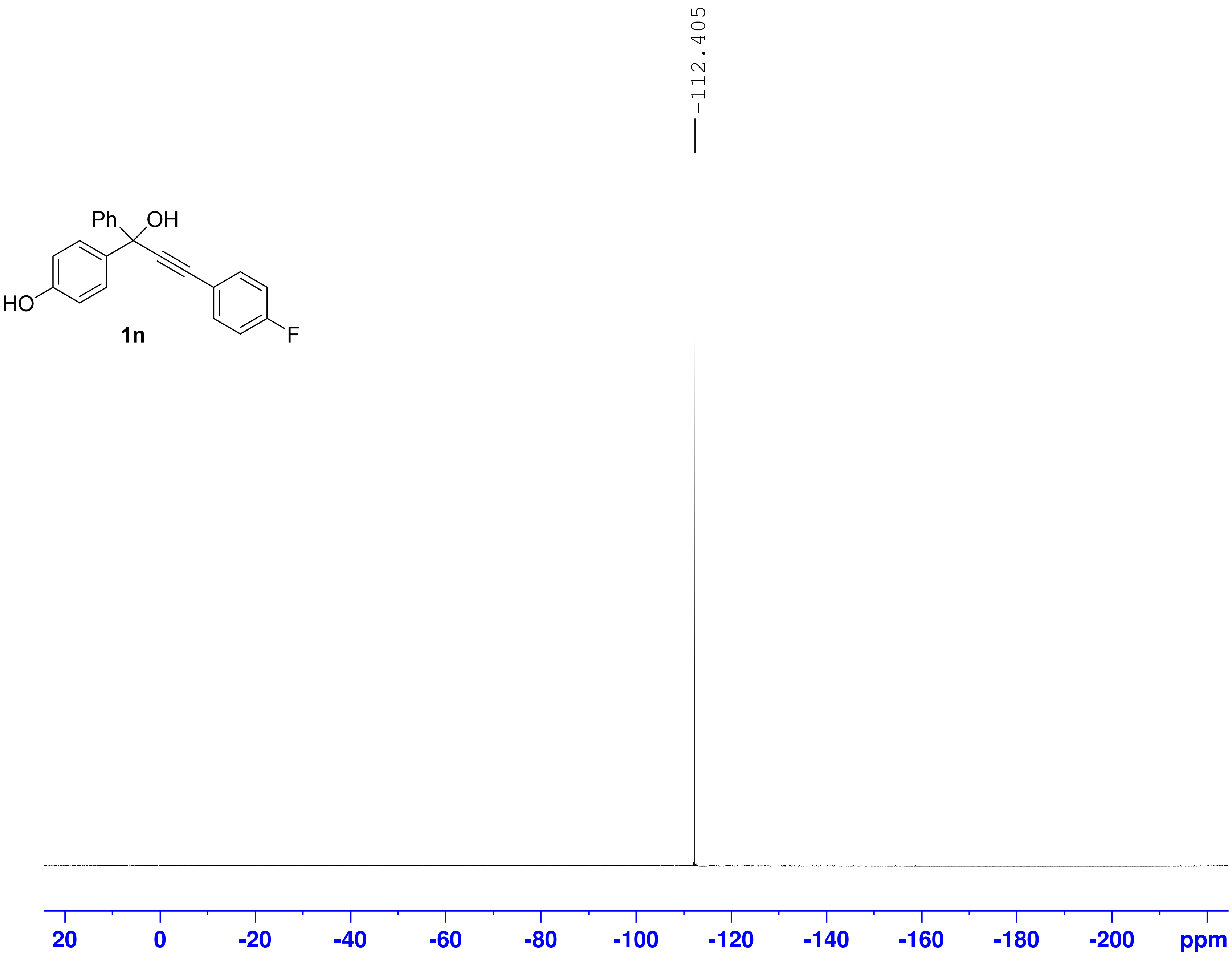


```
Current Data Parameters
NAME            qdy-20143-F
EXPNO                     1
PROCNO                    1

F2 - Acquisition Parameters
Date_              20160508
Time                  22.04
INSTRUM               spect
PROBHD     5 mm PABBO BB/
PULPROG             zgpg30
TD                    65536
SOLVENT             Acetone
NS                        4
DS                        0
SWH             93750.000 Hz
FIDRES           1.430511 Hz
AQ              0.3495253 sec
RG                  196.92
DW                   5.333 usec
DE                    6.50 usec
TE                   299.0 K
D1              2.00000000 sec
D11             0.03000000 sec
TD0                       1

======== CHANNEL f1 ========
SFO1          376.4607162 MHz
NUC1                    19F
P1                   14.70 usec
PLW1          15.99600029 W

======== CHANNEL f2 ========
SFO2          400.1316005 MHz
NUC2                     1H
CPDPRG[2            waltz16
PCPD2                90.00 usec
PLW2          11.99499989 W
PLW12          0.34213999 W
PLW13          0.27713001 W

F2 - Processing parameters
SI                    32768
SF            376.4983660 MHz
WDW                      EM
SSB        0
LB                     1.00 Hz
GB         0
PC                     1.40
```

Supplementary Figure 32. ^{19}F NMR spectrum for 1n

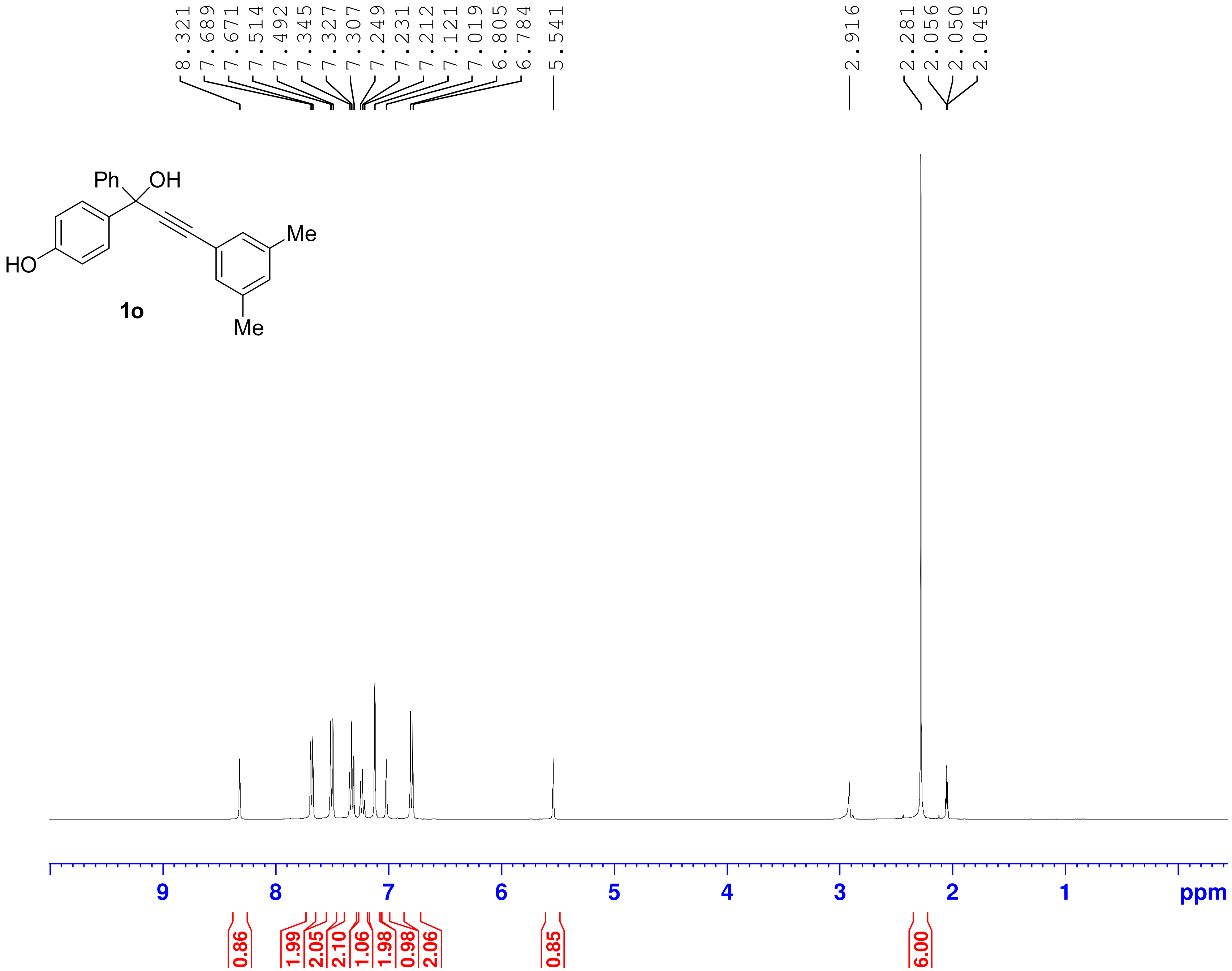

Current Data Parameters
NAME qdy-wcw-1013 H
EXPNO 2
PROCNO 1

F2 - Acquisition Parameters
Date_ 20160301
Time 21.43
INSTRUM spect
PROBHD 5 mm PABBO BB/
PULPROG zg30
TD 65536
SOLVENT Acetone
NS 16
DS 0
SWH 8012.820 Hz
FIDRES 0.122266 Hz
AQ 4.0894465 sec
RG 82.92
DW 62.400 usec
DE 6.50 usec
TE 297.3 K
D1 1.00000000 sec
TD0 1

======== CHANNEL f1 ========
SFO1 400.1324710 MHz
NUC1 1H
P1 14.50 usec
PLW1 11.99499989 W

F2 - Processing parameters
SI 65536
SF 400.1300066 MHz
WDW EM
SSB 0
LB 0.30 Hz
GB 0
PC 1.00

Supplementary Figure 33. ^{1}H NMR spectrum for 1o

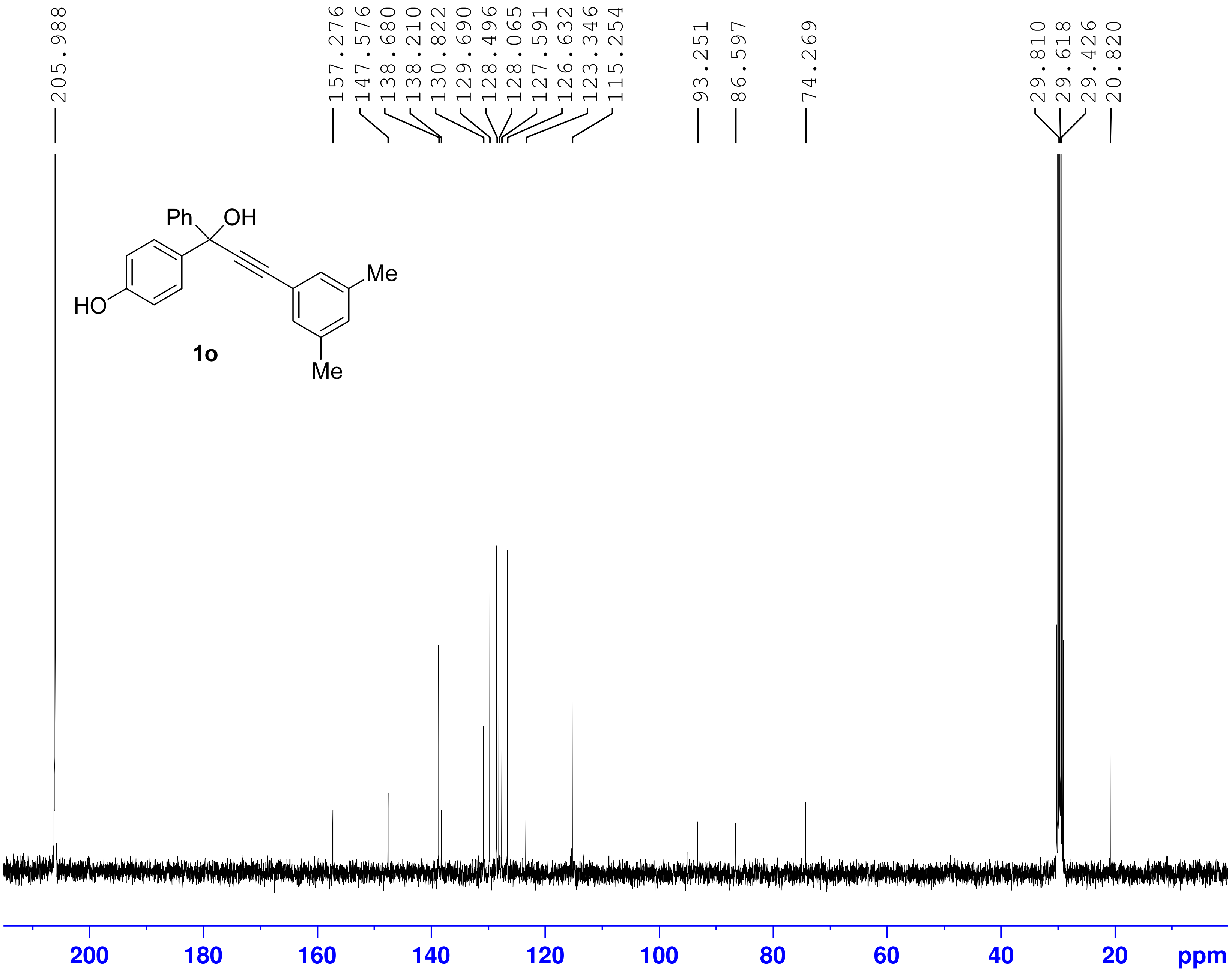

Current Data Parameters
NAME qdy-wcw-1013 C
EXPNO 2
PROCNO 1

F2 - Acquisition Parameters
Date_ 20160301
Time 21.36
INSTRUM spect
PROBHD 5 mm PABBO BB/
PULPROG zgpg30
TD 65536
SOLVENT Acetone
NS 81
DS 0
SWH 24038.461 Hz
FIDRES 0.366798 Hz
AQ 1.3631488 sec
RG 196.92
DW 20.800 usec
DE 6.50 usec
TE 297.7 K
D1 2.00000000 sec
D11 0.03000000 sec
TD0 1

======== CHANNEL f1 ========
SFO1 100.6228298 MHz
NUC1 13C
P1 9.70 usec
PLW1 46.98899841 W

======== CHANNEL f2 ========
SFO2 400.1316005 MHz
NUC2 1H
CPDPRG[2 waltz16
PCPD2 90.00 usec
PLW2 11.99499989 W
PLW12 0.34213999 W
PLW13 0.27713001 W

F2 - Processing parameters
SI 32768
SF 100.6127029 MHz
WDW EM
SSB 0
LB 1.00 Hz
GB 0
PC 1.40

Supplementary Figure 34. ^{13}C NMR spectrum for 1o

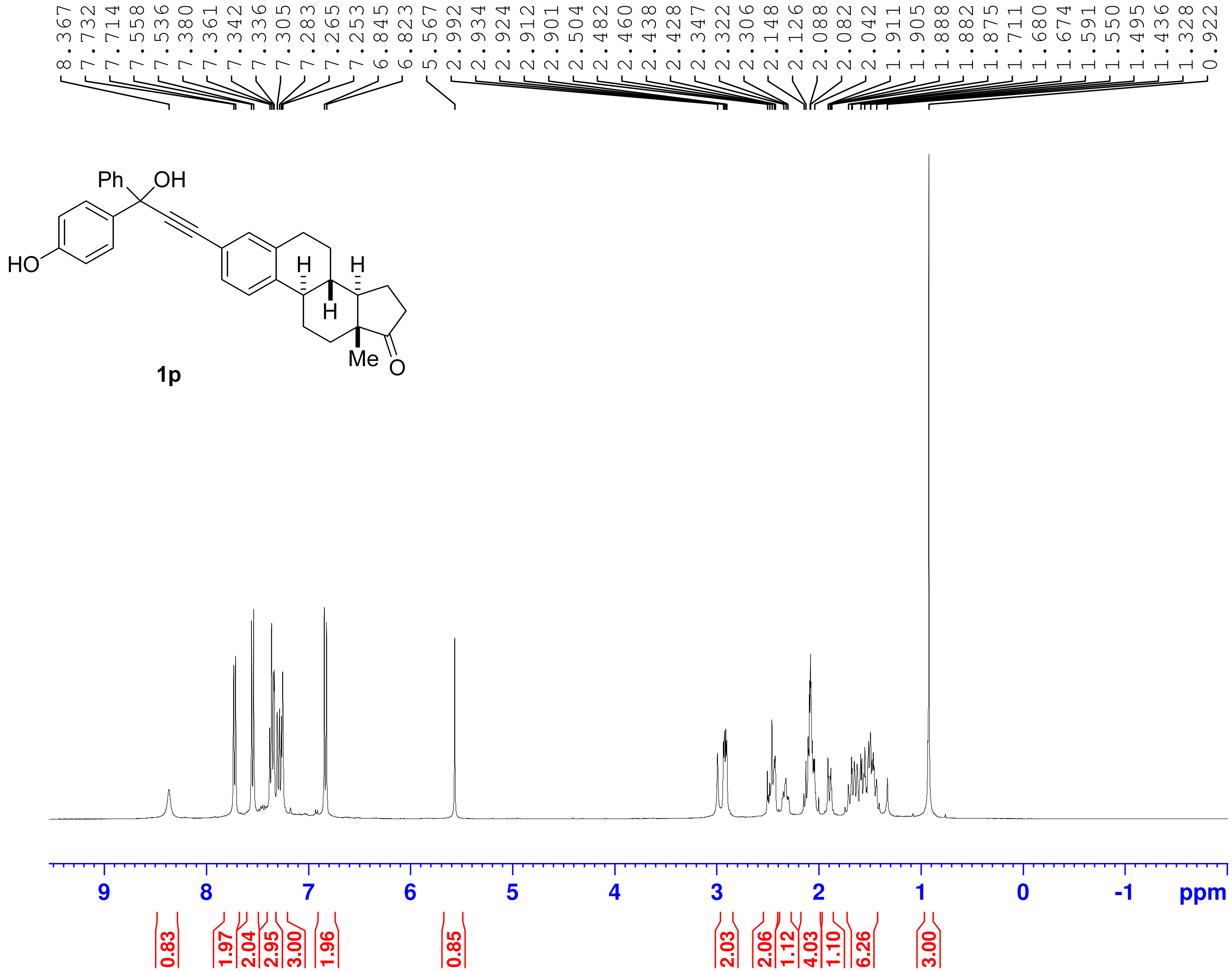

Current Data Parameters
NAME qdy-20110-2 H
EXPNO 3
PROCNO 1

F2 - Acquisition Parameters
Date_ 20160415
Time 23.03
INSTRUM spect
PROBHD 5 mm PABBO BB/
PULPROG zg30
TD 65536
SOLVENT Acetone
NS 12
DS 0
SWH 8012.820 Hz
FIDRES 0.122266 Hz
AQ 4.0894465 sec
RG 49.32
DW 62.400 usec
DE 6.50 usec
TE 297.7 K
D1 1.00000000 sec
TD0 1

======== CHANNEL f1 ========
SFO1 400.1324710 MHz
NUC1 1H
P1 14.50 usec
PLW1 11.99499989 W

F2 - Processing parameters
SI 65536
SF 400.1299916 MHz
WDW EM
SSB 0
LB 0.30 Hz
GB 0
PC 1.00

Supplementary Figure 35. ^{1}H NMR spectrum for 1p

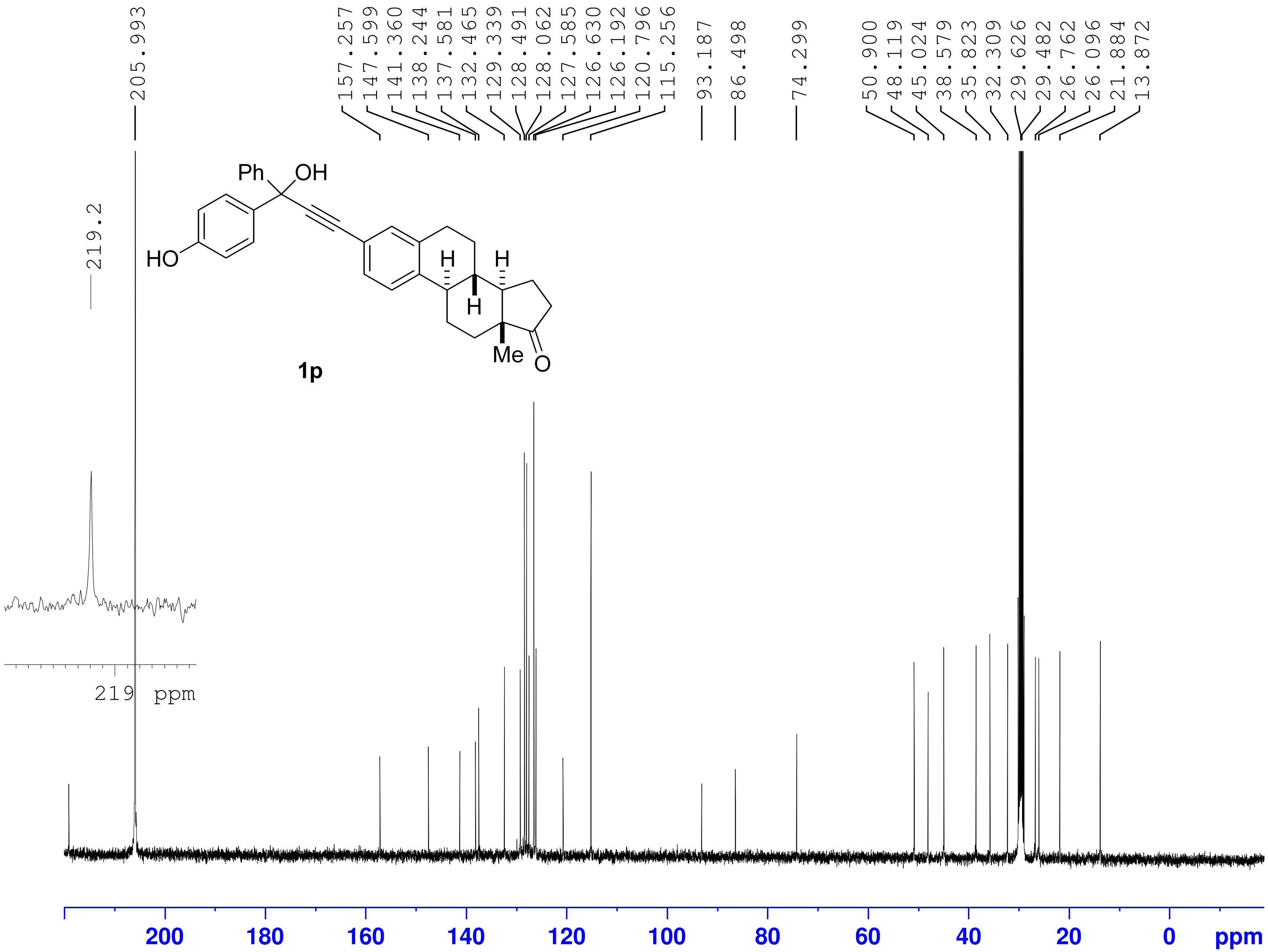


```
Current Data Parameters
NAME        qdy-20110-2 C
EXPNO                   3
PROCNO                  1

F2 - Acquisition Parameters
Date_            20160415
Time                23.10
INSTRUM             spect
PROBHD   5 mm PABBO BB/
PULPROG           zgpg30
TD                 65536
SOLVENT           Acetone
NS                   268
DS                     0
SWH            24038.461 Hz
FIDRES          0.366798 Hz
AQ             1.3631488 sec
RG                196.92
DW                20.800 usec
DE                  6.50 usec
TE                 298.8 K
D1            2.00000000 sec
D11           0.03000000 sec
TD0                    1

======== CHANNEL f1 ========
SFO1         100.6228298 MHz
NUC1                 13C
P1                  9.70 usec
PLW1         46.98899841 W

======== CHANNEL f2 ========
SFO2         400.1316005 MHz
NUC2                  1H
CPDPRG[2          waltz16
PCPD2              90.00 usec
PLW2         11.99499989 W
PLW12         0.34213999 W
PLW13         0.27713001 W

F2 - Processing parameters
SI                 32768
SF           100.6127042 MHz
WDW                   EM
SSB       0
LB                  1.00 Hz
GB        0
PC                  1.40
```

Supplementary Figure 36. ^{13}C NMR spectrum for 1p

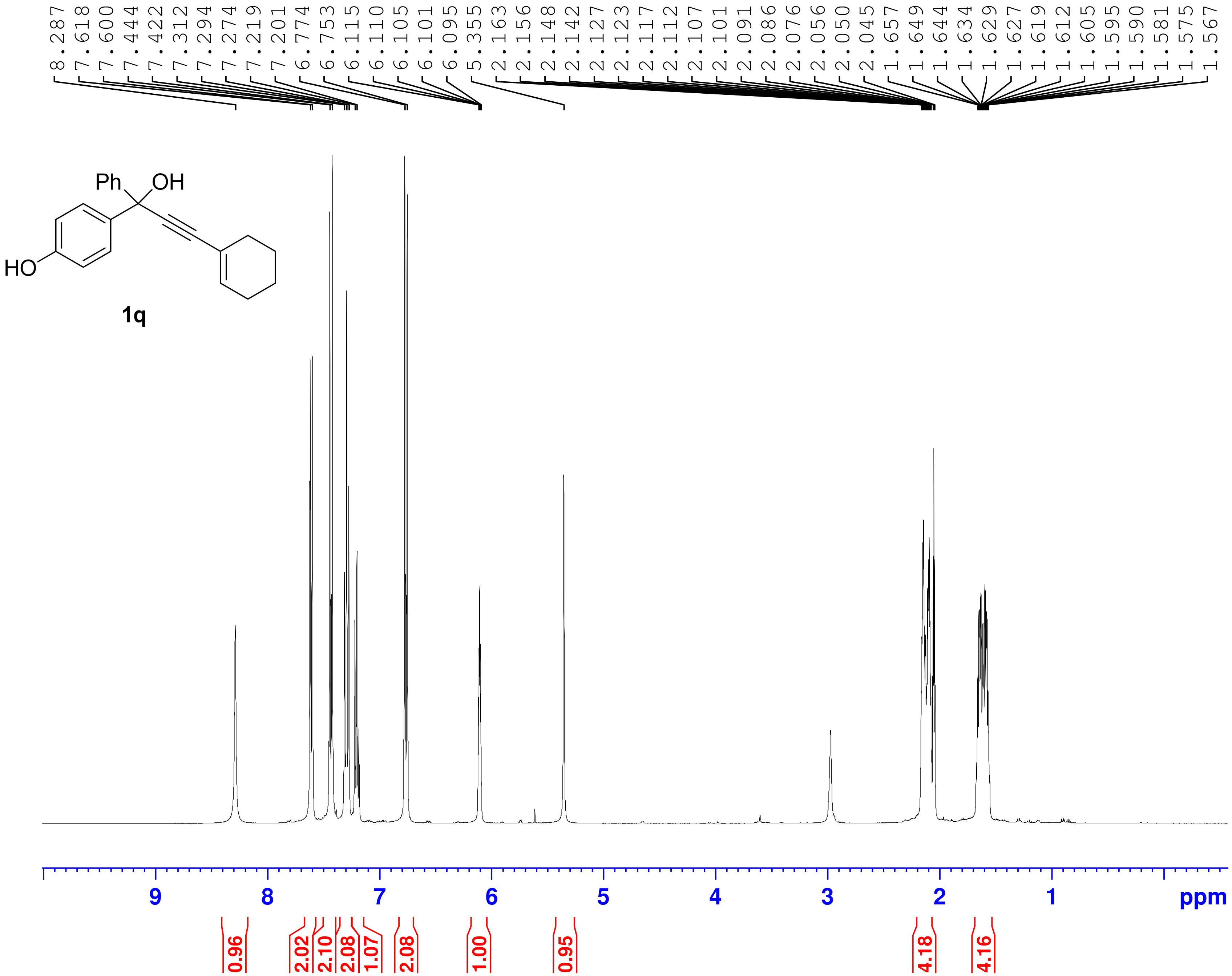

1q

Current Data Parameters
NAME qdy-20018 C
EXPNO 2
PROCNO 1

F2 - Acquisition Parameters
Date_ 20160328
Time 16.34
INSTRUM spect
PROBHD 5 mm PABBO BB/
PULPROG zg30
TD 65536
SOLVENT Acetone
NS 16
DS 0
SWH 8012.820 Hz
FIDRES 0.122266 Hz
AQ 4.0894465 sec
RG 70.97
DW 62.400 usec
DE 6.50 usec
TE 296.8 K
D1 1.00000000 sec
TD0 1

======== CHANNEL f1 ========
SFO1 400.1324710 MHz
NUC1 1H
P1 14.50 usec
PLW1 11.99499989 W

F2 - Processing parameters
SI 65536
SF 400.1300066 MHz
WDW EM
SSB 0
LB 0.30 Hz
GB 0
PC 1.00

Supplementary Figure 37. ^{1}H NMR spectrum for 1q

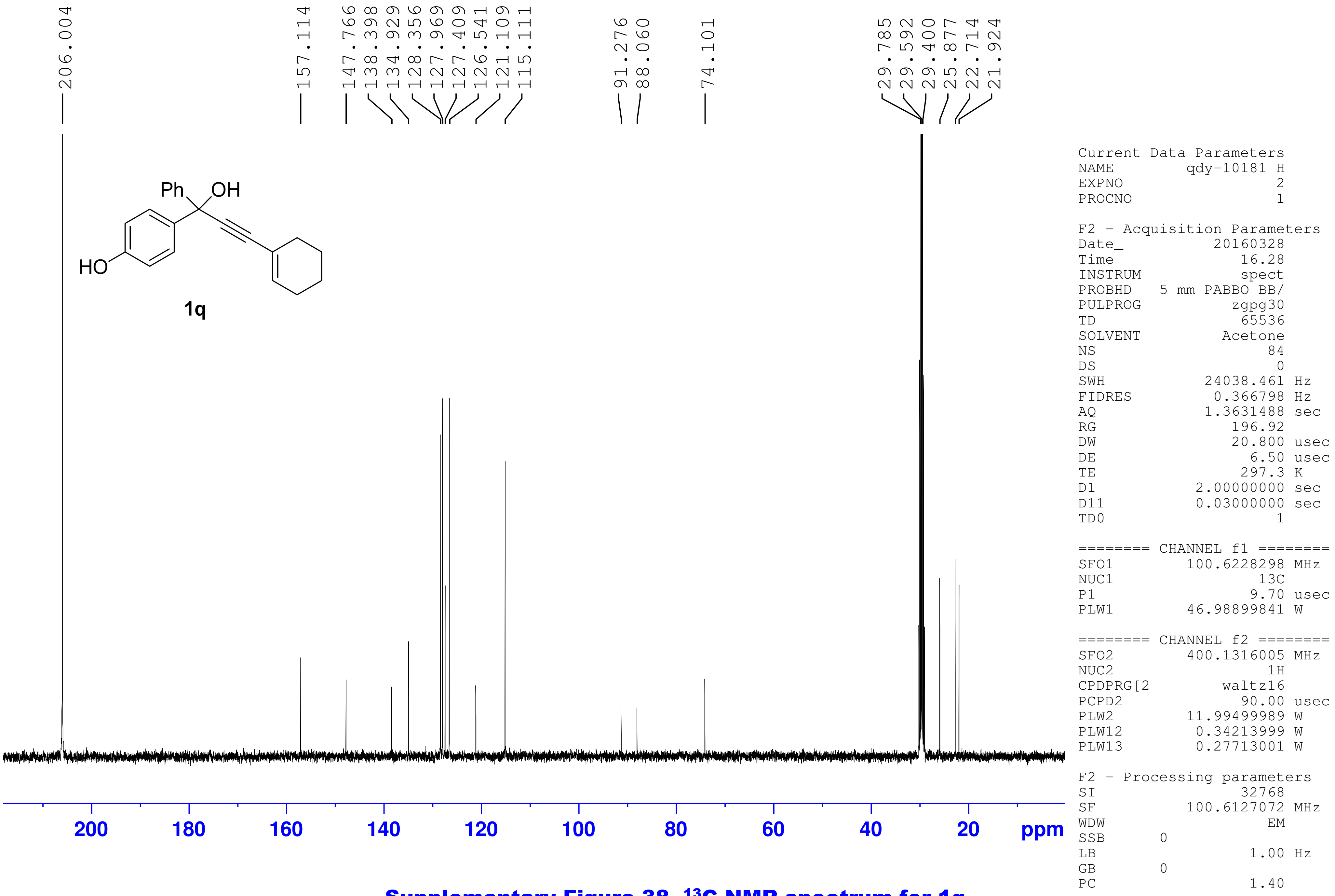

Supplementary Figure 38. ^{13}C NMR spectrum for 1q

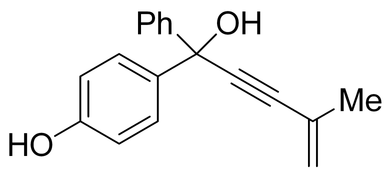

1r

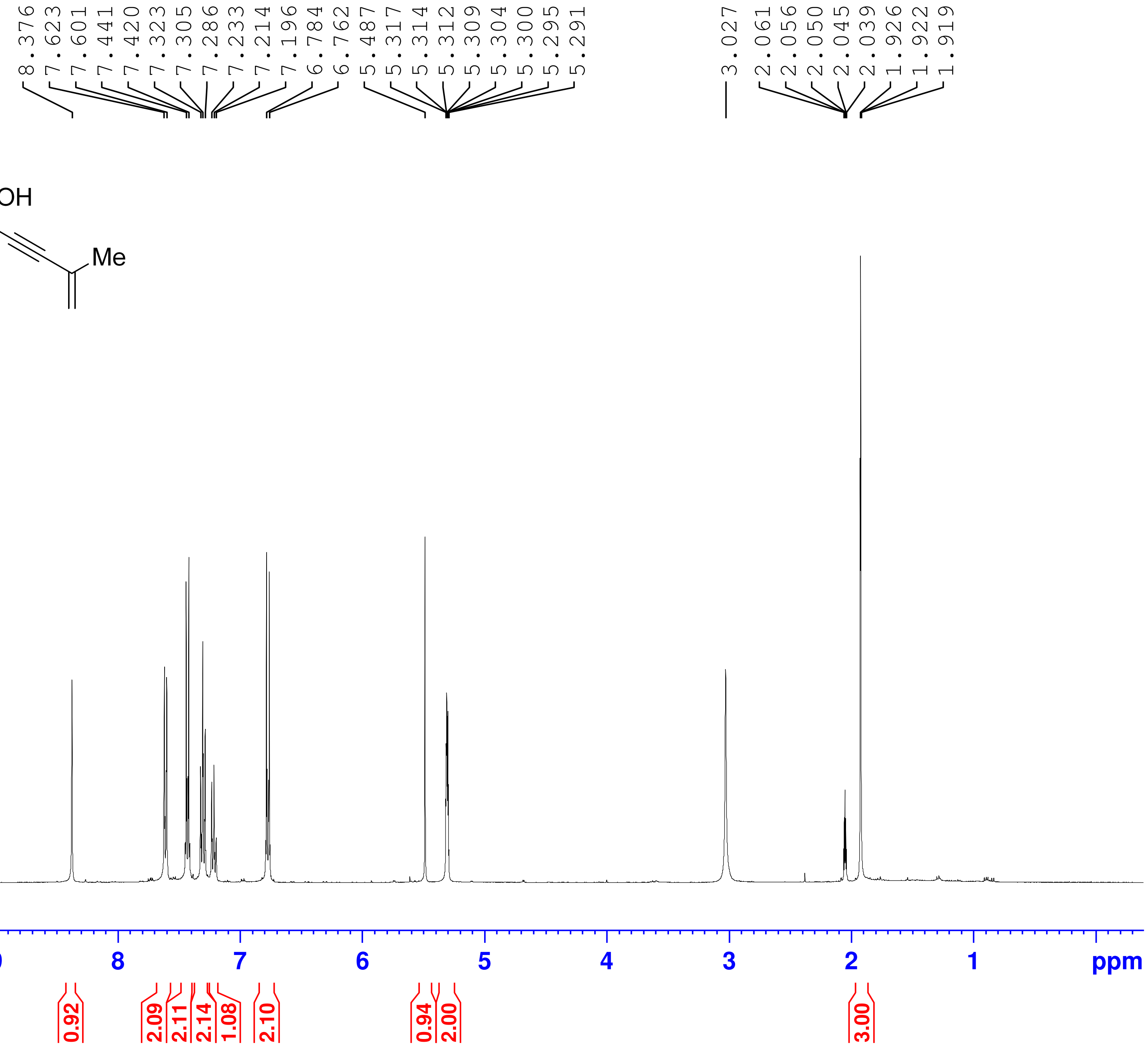

Current Data Parameters
NAME qdy-20063 H
EXPNO 2
PROCNO 1

F2 - Acquisition Parameters
Date_ 20160310
Time 20.01
INSTRUM spect
PROBHD 5 mm PABBO BB/
PULPROG zg30
TD 65536
SOLVENT Acetone
NS 5
DS 0
SWH 8012.820 Hz
FIDRES 0.122266 Hz
AQ 4.0894465 sec
RG 88.84
DW 62.400 usec
DE 6.50 usec
TE 296.9 K
D1 1.00000000 sec
TD0 1

======== CHANNEL f1 ========
SFO1 400.1324710 MHz
NUC1 1H
P1 14.50 usec
PLW1 11.99499989 W

F2 - Processing parameters
SI 65536
SF 400.1300067 MHz
WDW EM
SSB 0
LB 0.30 Hz
GB 0
PC 1.00

Supplementary Figure 39. ^{1}H NMR spectrum for 1r

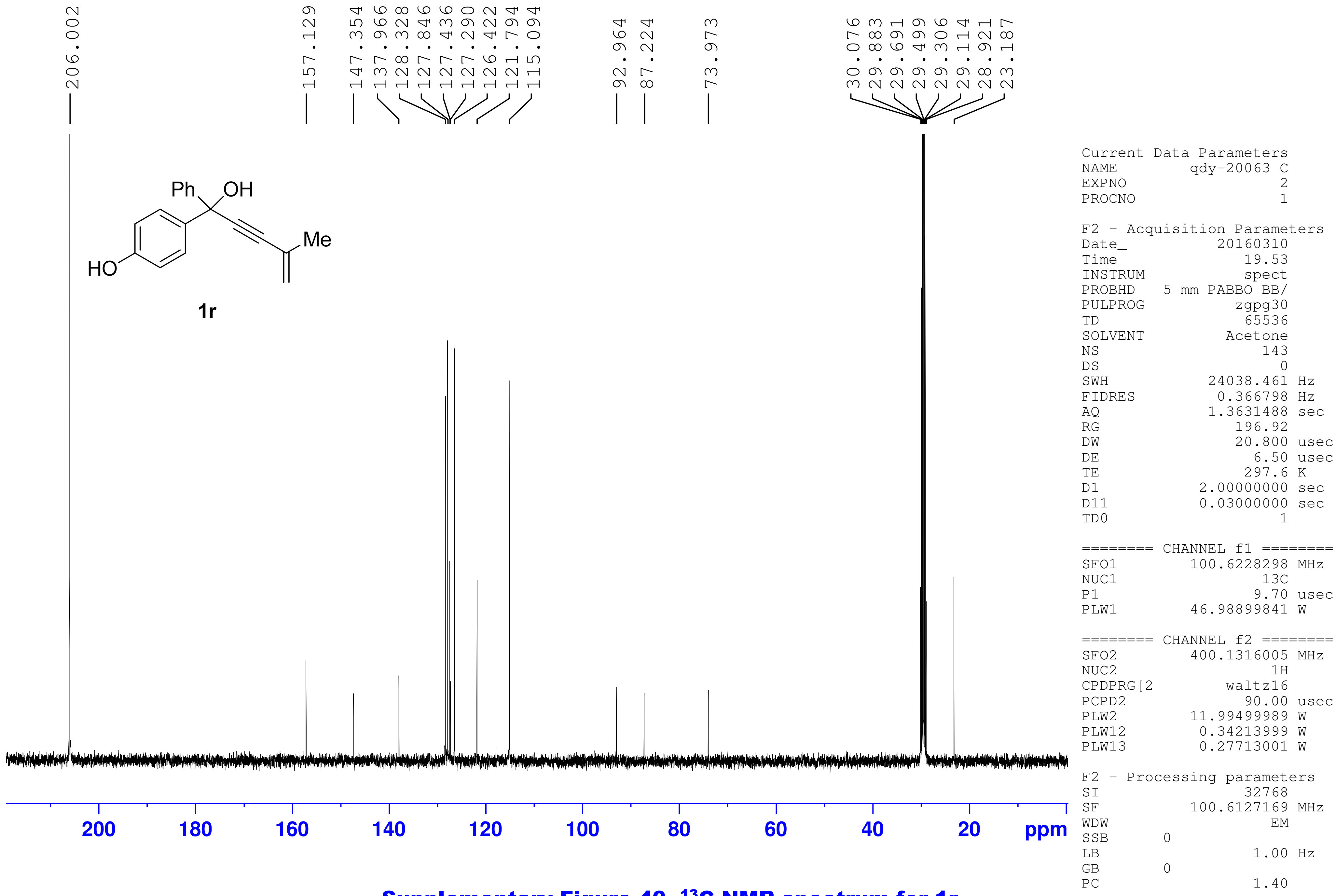

Supplementary Figure 40. ^{13}C NMR spectrum for 1r

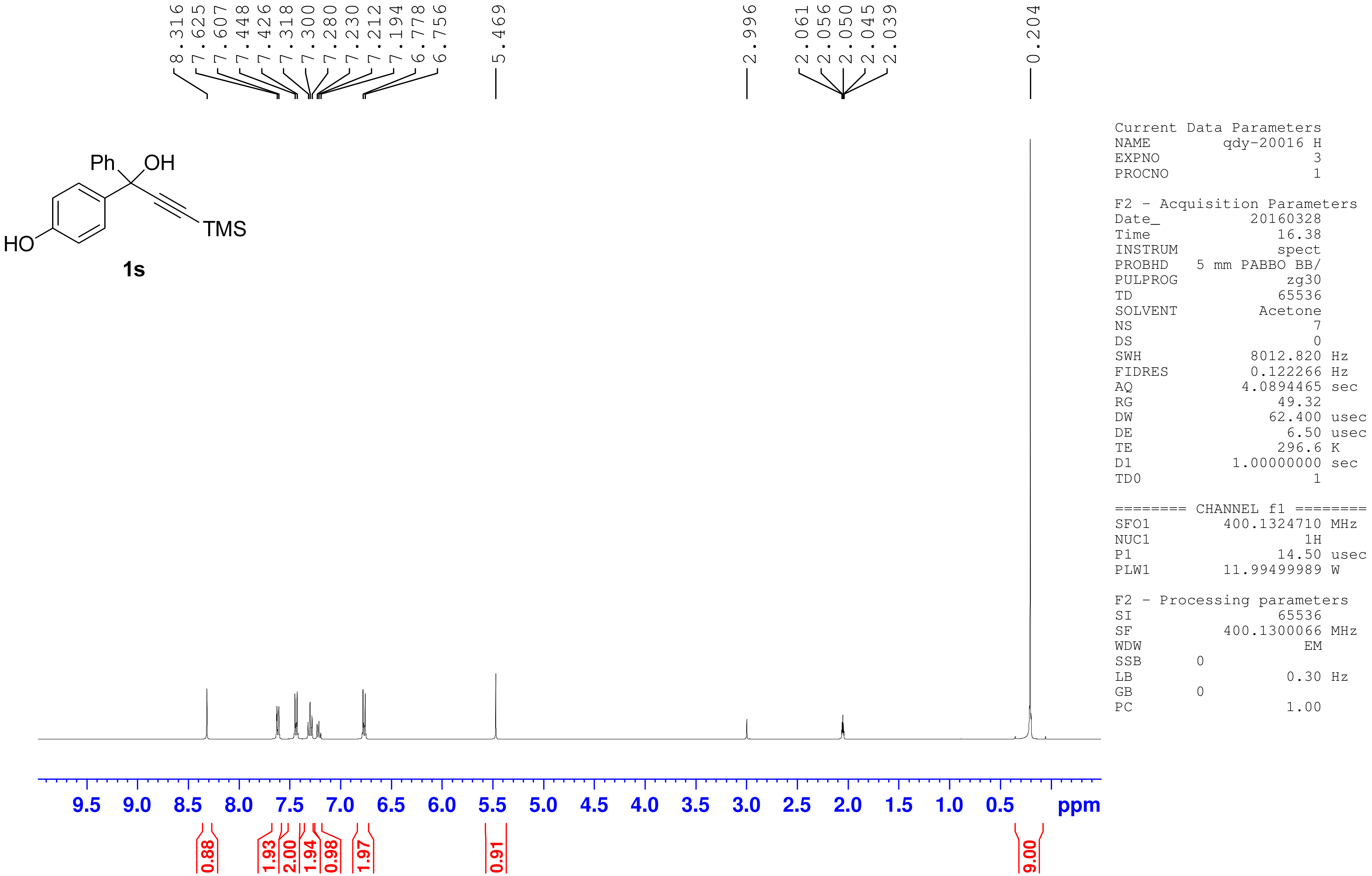

Supplementary Figure 41. ^{1}H NMR spectrum for 1s

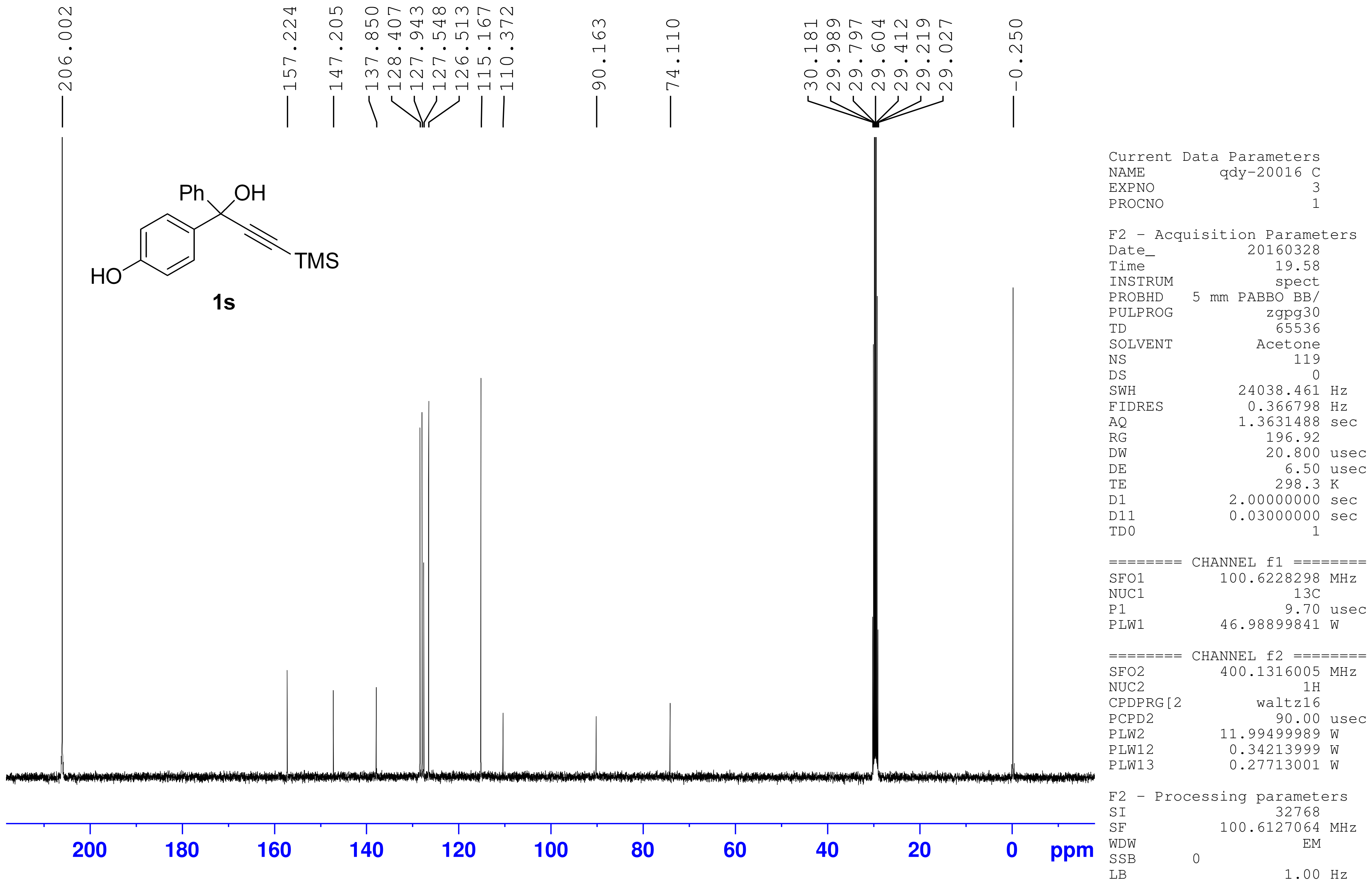

Supplementary Figure 42. ^{13}C NMR spectrum for 1s

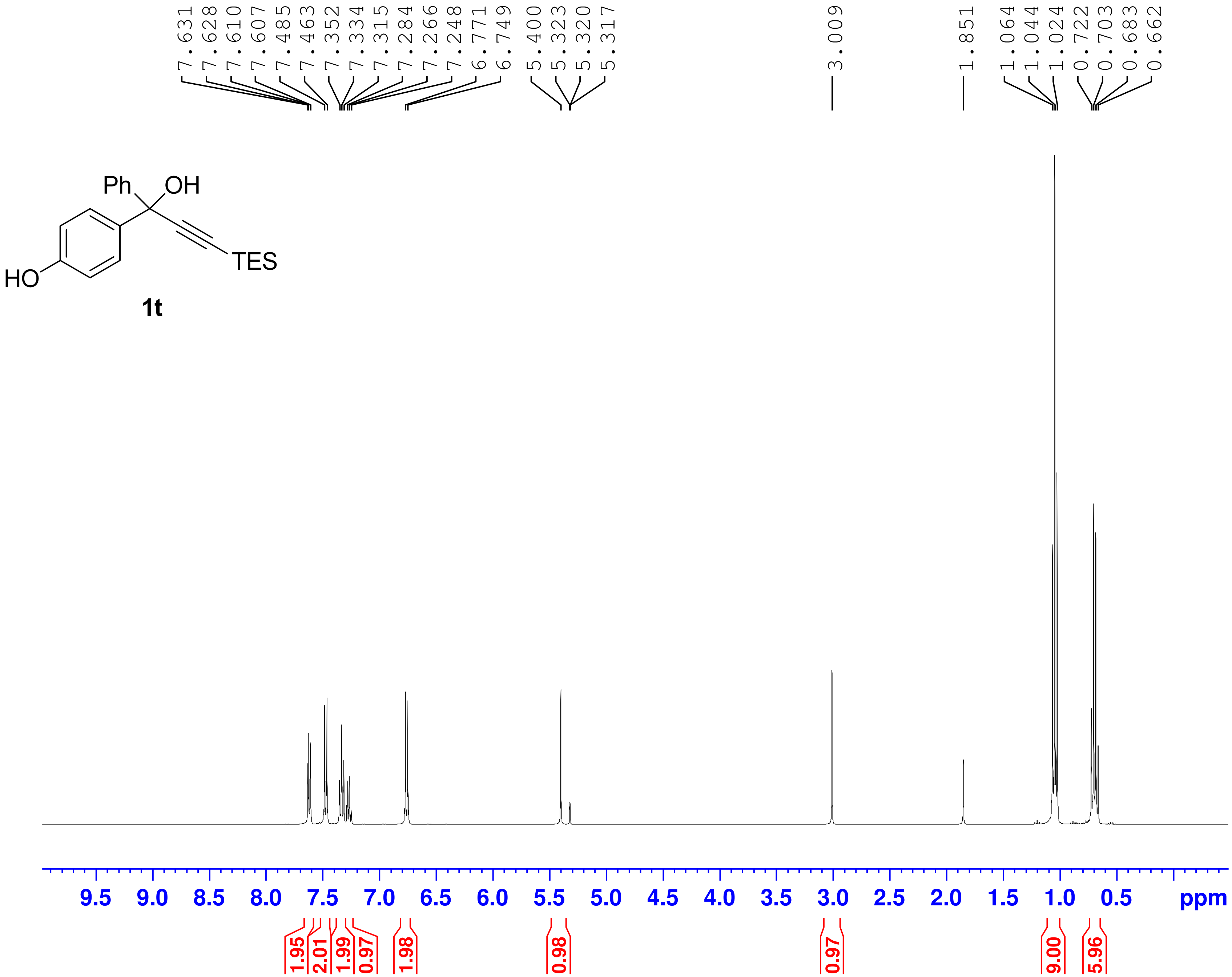

Current Data Parameters
NAME qdy-20085-2 H
EXPNO 1
PROCNO 1

F2 - Acquisition Parameters
Date_ 20160319
Time 19.48
INSTRUM spect
PROBHD 5 mm PABBO BB/
PULPROG zg30
TD 65536
SOLVENT CD2Cl2
NS 14
DS 0
SWH 8012.820 Hz
FIDRES 0.122266 Hz
AQ 4.0894465 sec
RG 39.46
DW 62.400 usec
DE 6.50 usec
TE 297.9 K
D1 1.00000000 sec
TD0 1

======== CHANNEL f1 ========
SFO1 400.1324710 MHz
NUC1 1H
P1 14.50 usec
PLW1 11.99499989 W

F2 - Processing parameters
SI 65536
SF 400.1300152 MHz
WDW EM
SSB 0
LB 0.30 Hz
GB 0
PC 1.00

Supplementary Figure 43. ¹H NMR spectrum for 1t

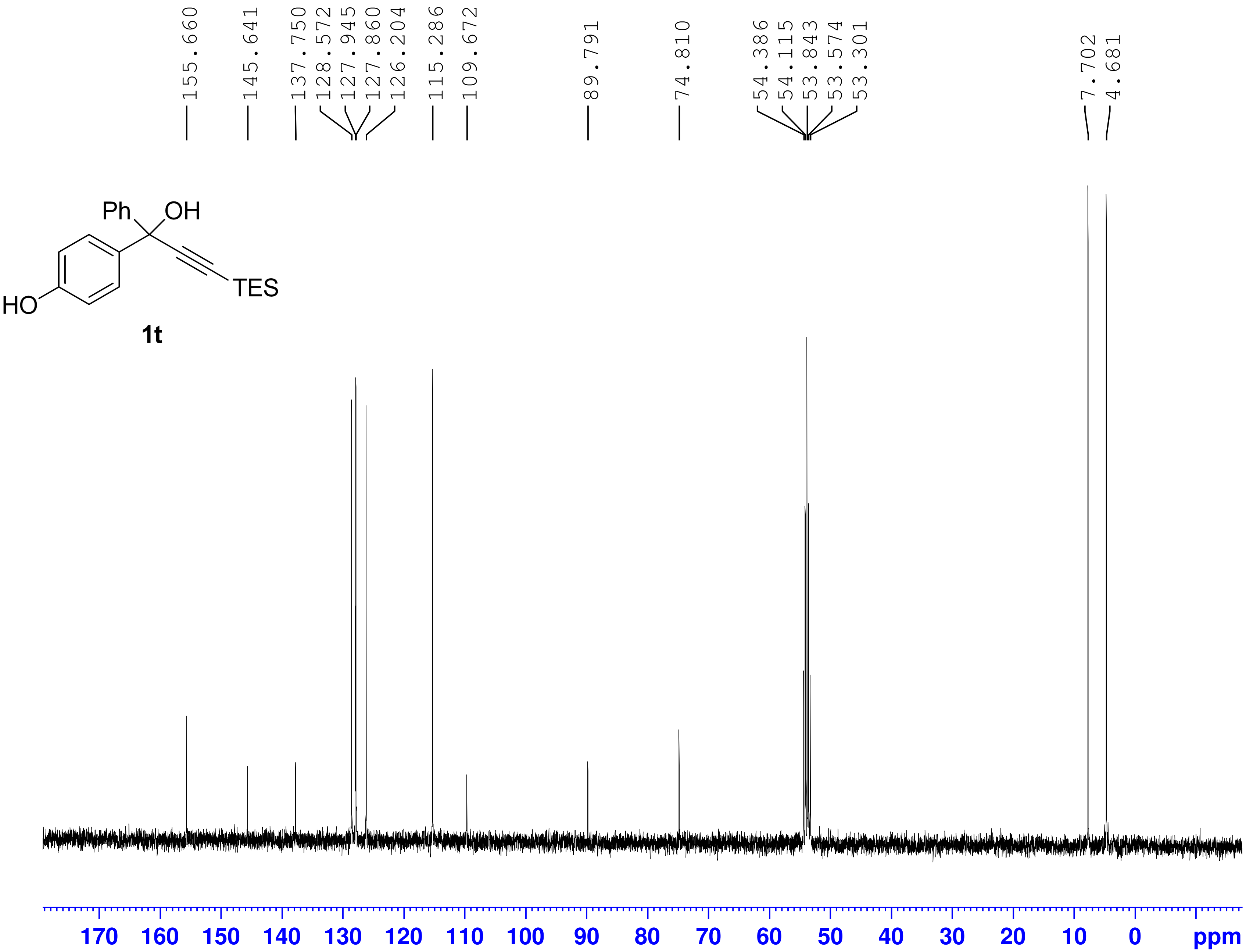


```
Current Data Parameters
NAME     qdy-200885-2 C
EXPNO                 1
PROCNO                1

F2 - Acquisition Parameters
Date_          20160318
Time              21.45
INSTRUM           spect
PROBHD   5 mm PABBO BB/
PULPROG         zgpg30
TD               65536
SOLVENT         CD2Cl2
NS                  98
DS                   0
SWH          24038.461 Hz
FIDRES        0.366798 Hz
AQ           1.3631488 sec
RG              196.92
DW              20.800 usec
DE                6.50 usec
TE               298.0 K
D1          2.00000000 sec
D11         0.03000000 sec
TD0                  1

======== CHANNEL f1 ========
SFO1       100.6228298 MHz
NUC1               13C
P1                9.70 usec
PLW1       46.98899841 W

======== CHANNEL f2 ========
SFO2       400.1316005 MHz
NUC2                1H
CPDPRG[2        waltz16
PCPD2            90.00 usec
PLW2       11.99499989 W
PLW12       0.34213999 W
PLW13       0.27713001 W

F2 - Processing parameters
SI               32768
SF         100.6127270 MHz
WDW                 EM
SSB      0
LB                1.00 Hz
GB       0
PC                1.40
```

Supplementary Figure 44. ^{13}C NMR spectrum for 1t

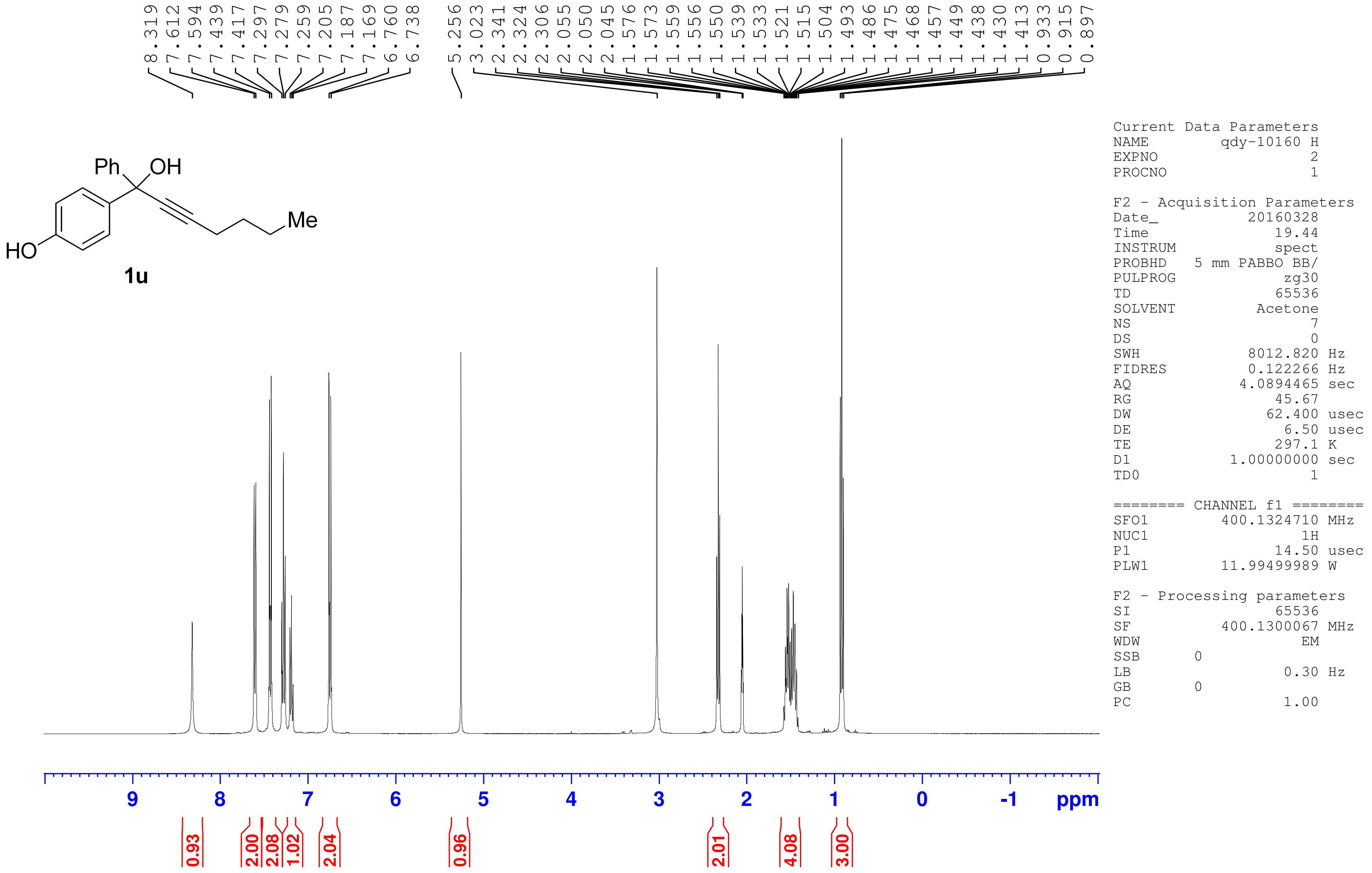

Supplementary Figure 45. ^{1}H NMR spectrum for 1u

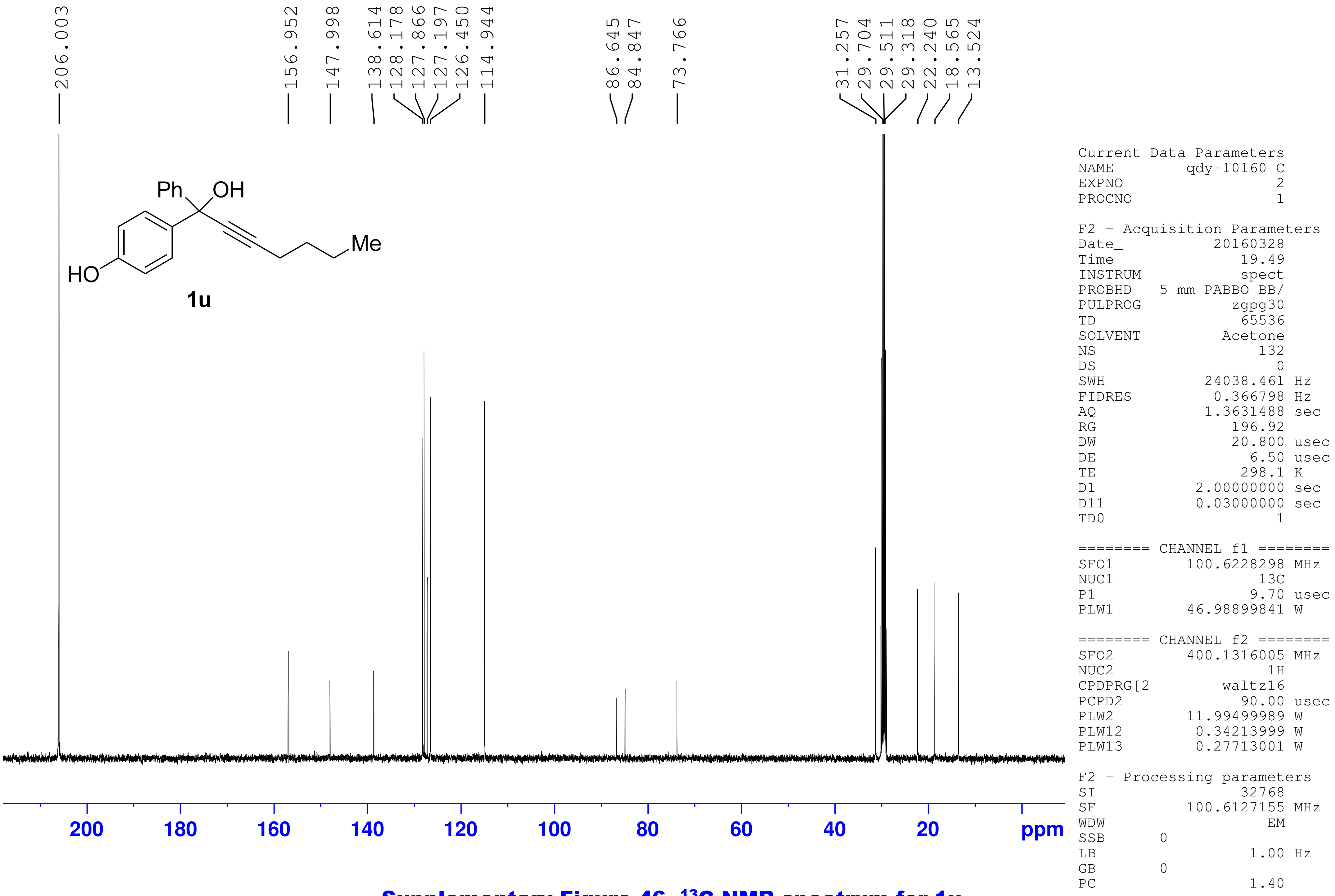

Supplementary Figure 46. ^{13}C NMR spectrum for 1u

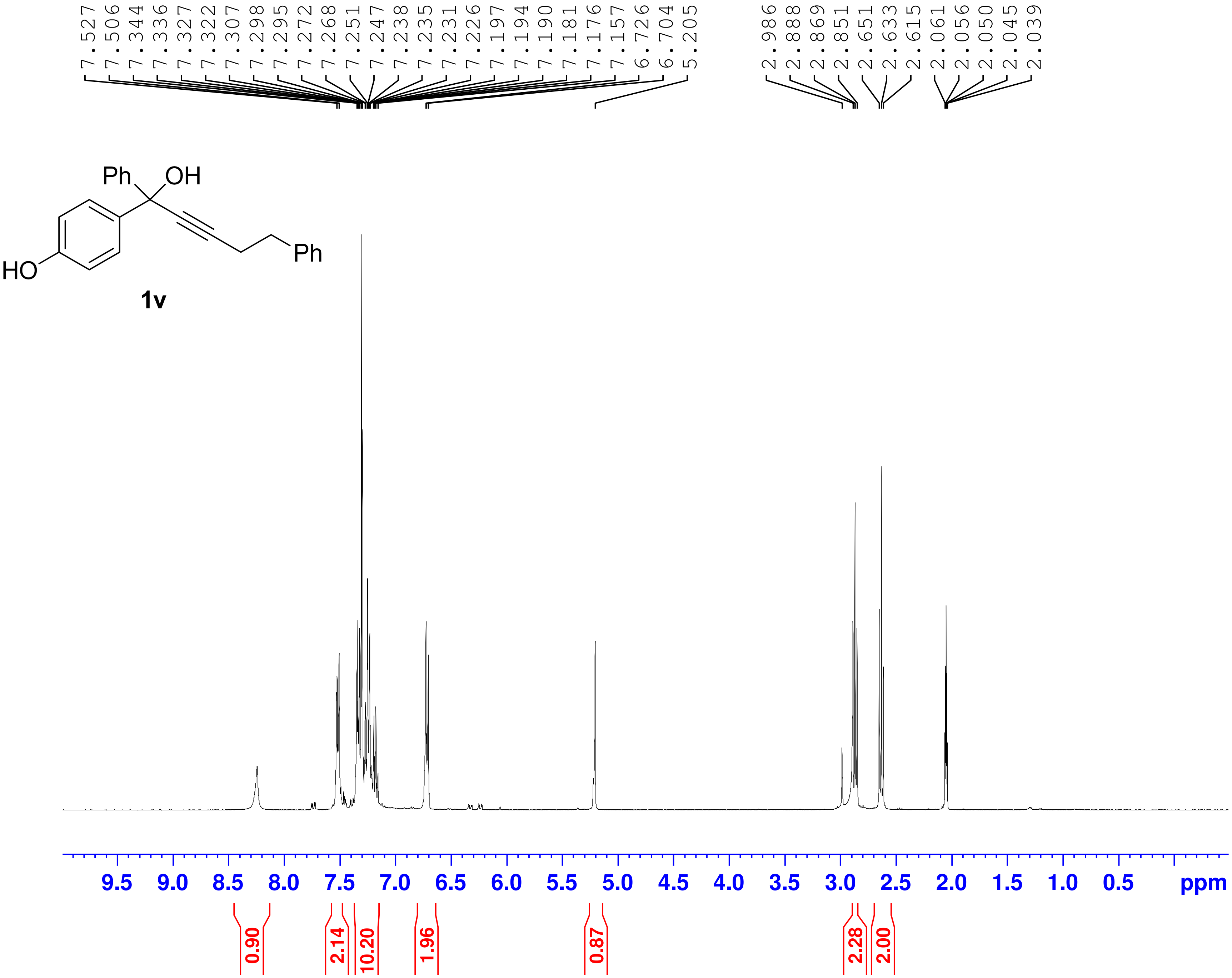

1v

Current Data Parameters
NAME qdy-20116 H
EXPNO 2
PROCNO 1

F2 - Acquisition Parameters
Date_ 20160418
Time 21.43
INSTRUM spect
PROBHD 5 mm PABBO BB/
PULPROG zg30
TD 65536
SOLVENT Acetone
NS 6
DS 0
SWH 8012.820 Hz
FIDRES 0.122266 Hz
AQ 4.0894465 sec
RG 103.52
DW 62.400 usec
DE 6.50 usec
TE 296.9 K
D1 1.00000000 sec
TD0 1

======== CHANNEL f1 ========
SFO1 400.1324710 MHz
NUC1 1H
P1 14.50 usec
PLW1 11.99499989 W

F2 - Processing parameters
SI 65536
SF 400.1300067 MHz
WDW EM
SSB 0
LB 0.30 Hz
GB 0
PC 1.00

Supplementary Figure 47. ^{1}H NMR spectrum for 1v

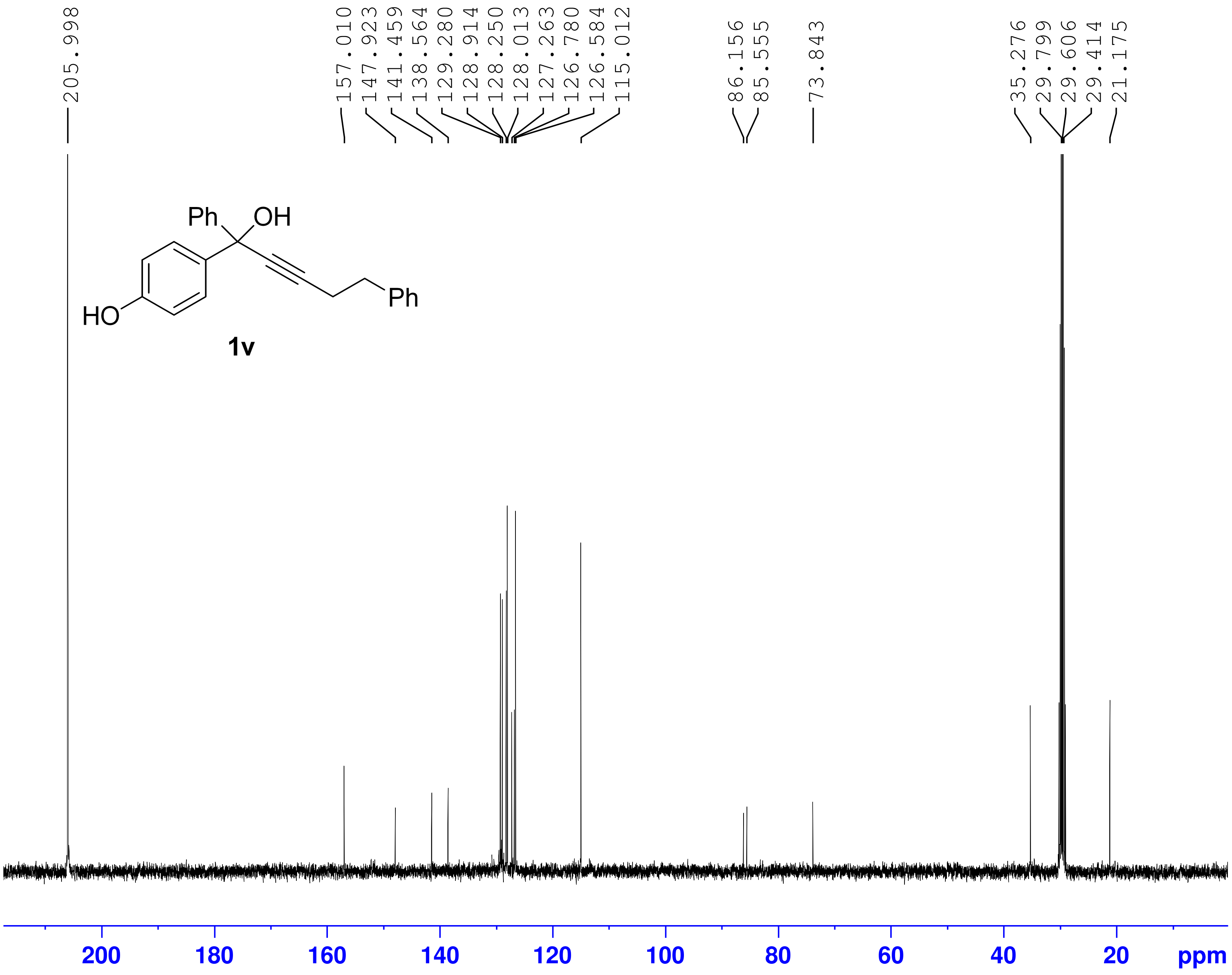

Current Data Parameters
NAME qdy-20116 C
EXPNO 1
PROCNO 1

F2 - Acquisition Parameters
Date_ 20160418
Time 19.49
INSTRUM spect
PROBHD 5 mm PABBO BB/
PULPROG zgpg30
TD 65536
SOLVENT Acetone
NS 139
DS 0
SWH 24038.461 Hz
FIDRES 0.366798 Hz
AQ 1.3631488 sec
RG 196.92
DW 20.800 usec
DE 6.50 usec
TE 297.7 K
D1 2.00000000 sec
D11 0.03000000 sec
TD0 1

======== CHANNEL f1 ========
SFO1 100.6228298 MHz
NUC1 13C
P1 9.70 usec
PLW1 46.98899841 W

======== CHANNEL f2 ========
SFO2 400.1316005 MHz
NUC2 1H
CPDPRG[2 waltz16
PCPD2 90.00 usec
PLW2 11.99499989 W
PLW12 0.34213999 W
PLW13 0.27713001 W

F2 - Processing parameters
SI 32768
SF 100.6127051 MHz
WDW EM
SSB 0
LB 1.00 Hz
GB 0
PC 1.40

Supplementary Figure 48. ^{13}C NMR spectrum for 1v

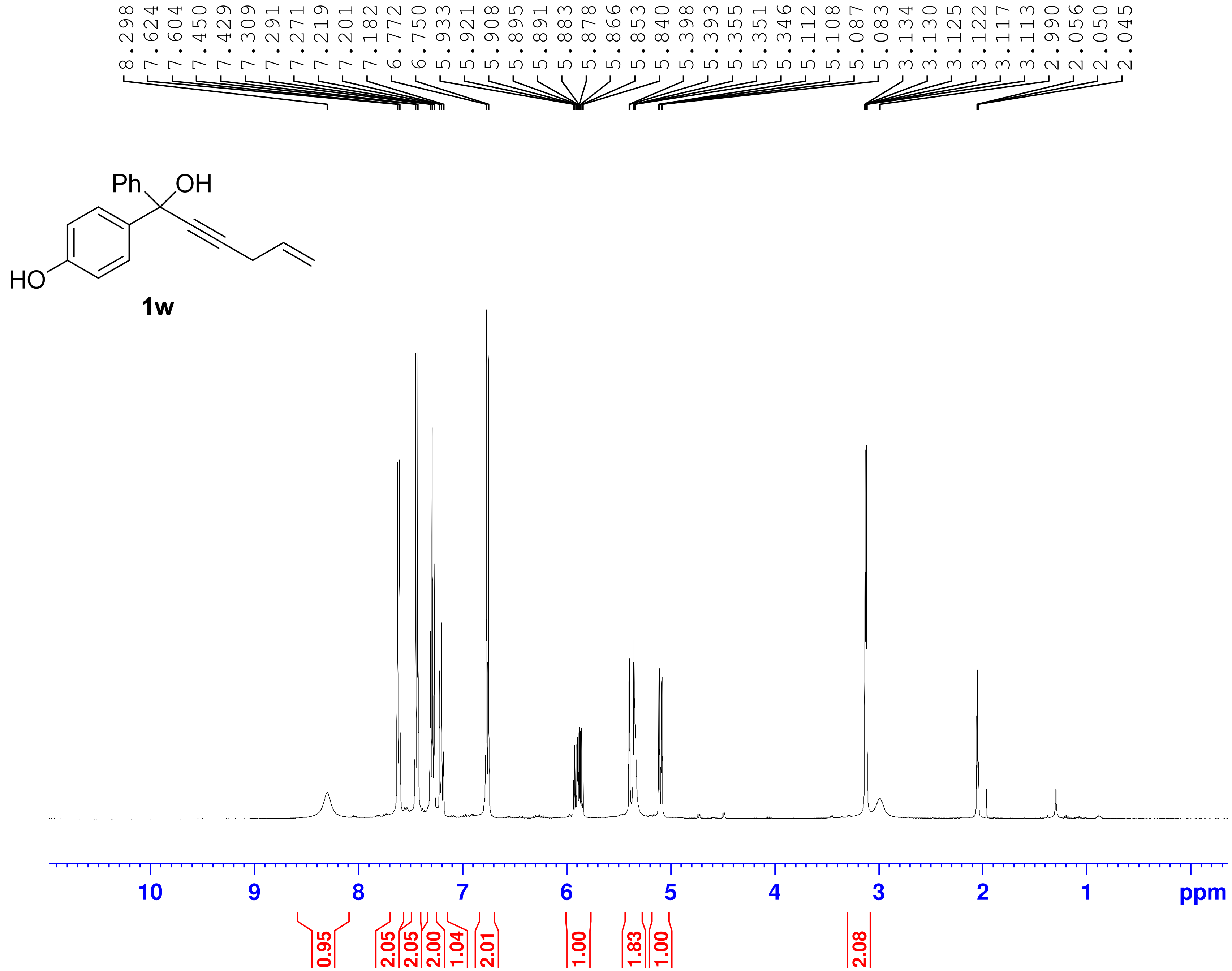

Current Data Parameters
NAME qdy-20114-c H
EXPNO 2
PROCNO 1

F2 - Acquisition Parameters
Date_ 20160416
Time 19.16
INSTRUM spect
PROBHD 5 mm PABBO BB/
PULPROG zg30
TD 65536
SOLVENT Acetone
NS 5
DS 0
SWH 8012.820 Hz
FIDRES 0.122266 Hz
AQ 4.0894465 sec
RG 54.81
DW 62.400 usec
DE 6.50 usec
TE 298.2 K
D1 1.00000000 sec
TD0 1

======== CHANNEL f1 ========
SFO1 400.1324710 MHz
NUC1 1H
P1 14.50 usec
PLW1 11.99499989 W

F2 - Processing parameters
SI 65536
SF 400.1300066 MHz
WDW EM
SSB 0
LB 0.30 Hz
GB 0
PC 1.00

Supplementary Figure 49. ^{1}H NMR spectrum for 1w

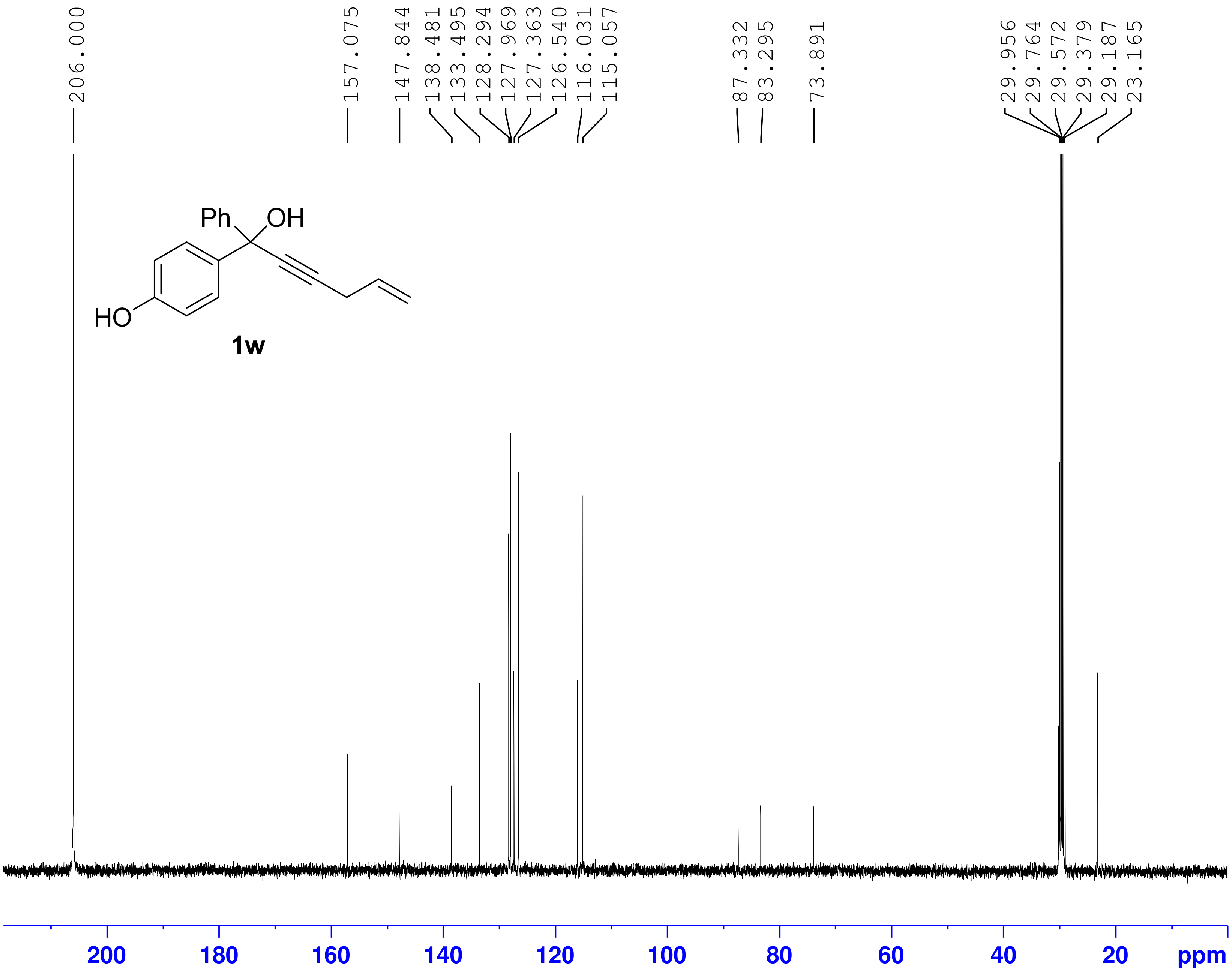


```
Current Data Parameters
NAME      qdy-20114-c C
EXPNO                 2
PROCNO                1

F2 - Acquisition Parameters
Date_          20160416
Time              19.20
INSTRUM           spect
PROBHD   5 mm PABBO BB/
PULPROG         zgpg30
TD               65536
SOLVENT         Acetone
NS                  86
DS                   0
SWH          24038.461 Hz
FIDRES        0.366798 Hz
AQ           1.3631488 sec
RG              196.92
DW              20.800 usec
DE                6.50 usec
TE               299.2 K
D1          2.00000000 sec
D11         0.03000000 sec
TD0                  1

======== CHANNEL f1 ========
SFO1       100.6228298 MHz
NUC1               13C
P1                9.70 usec
PLW1       46.98899841 W

======== CHANNEL f2 ========
SFO2       400.1316005 MHz
NUC2                1H
CPDPRG[2        waltz16
PCPD2             90.00 usec
PLW2       11.99499989 W
PLW12       0.34213999 W
PLW13       0.27713001 W

F2 - Processing parameters
SI               32768
SF         100.6127088 MHz
WDW                 EM
SSB      0
LB                1.00 Hz
GB       0
PC                1.40
```

Supplementary Figure 50. ^{13}C NMR spectrum for 1w

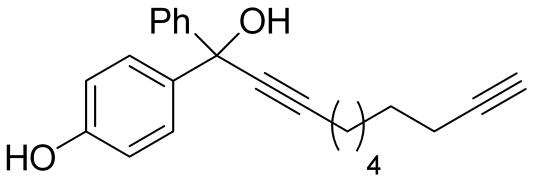

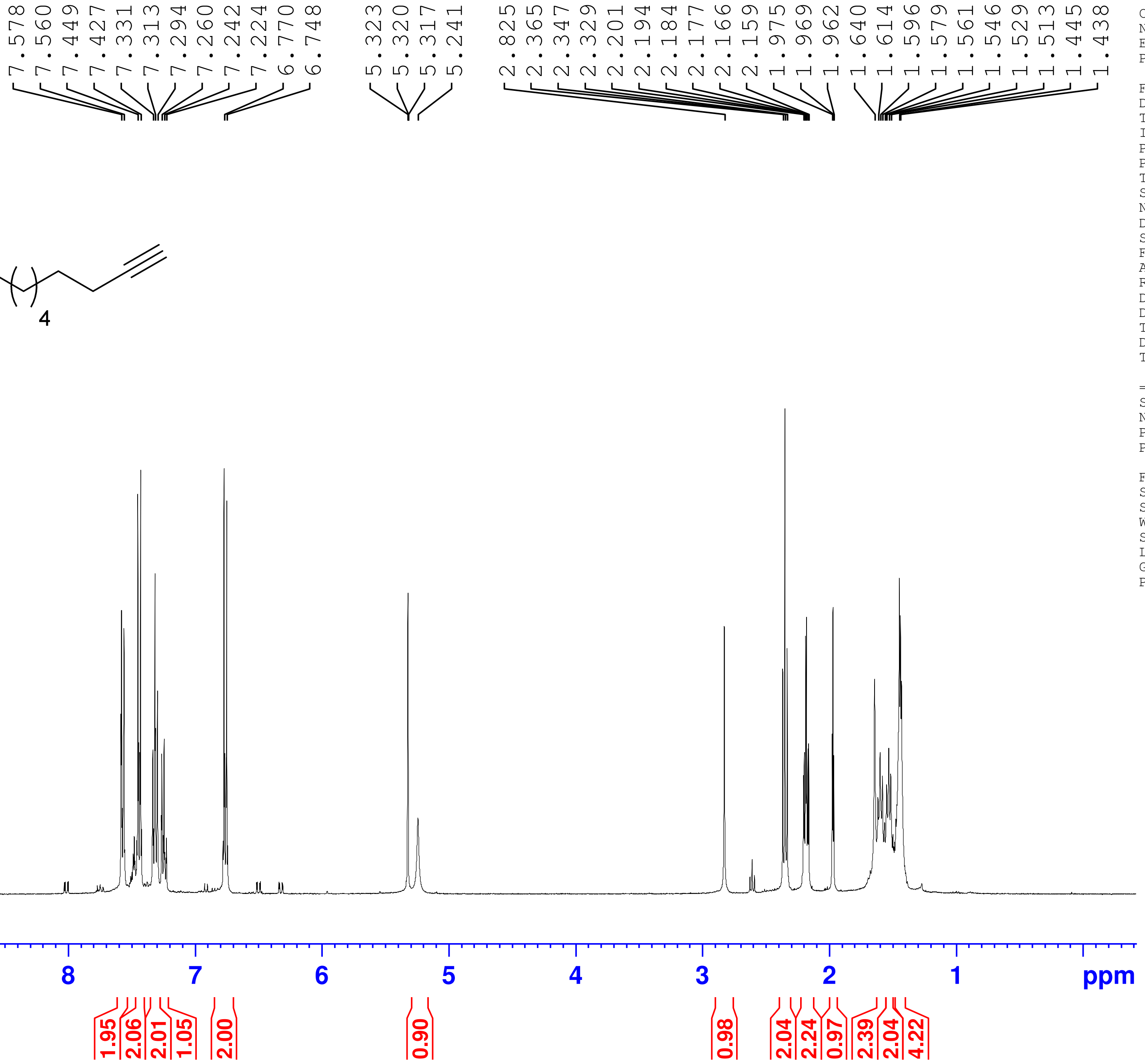

Current Data Parameters
NAME qdy-20082-1 H
EXPNO 3
PROCNO 1

F2 - Acquisition Parameters
Date_ 20160317
Time 16.48
INSTRUM spect
PROBHD 5 mm PABBO BB/
PULPROG zg30
TD 65536
SOLVENT CD2Cl2
NS 7
DS 0
SWH 8012.820 Hz
FIDRES 0.122266 Hz
AQ 4.0894465 sec
RG 103.52
DW 62.400 usec
DE 6.50 usec
TE 296.4 K
D1 1.00000000 sec
TD0 1

======== CHANNEL f1 ========
SFO1 400.1324710 MHz
NUC1 1H
P1 14.50 usec
PLW1 11.99499989 W

F2 - Processing parameters
SI 65536
SF 400.1300153 MHz
WDW EM
SSB 0
LB 0.30 Hz
GB 0
PC 1.00

1x

Supplementary Figure 51. ^{1}H NMR spectrum for 1x

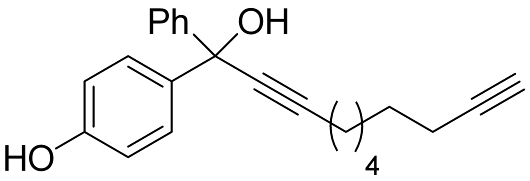

1x

Supplementary Figure 52. ^{13}C NMR spectrum for 1x

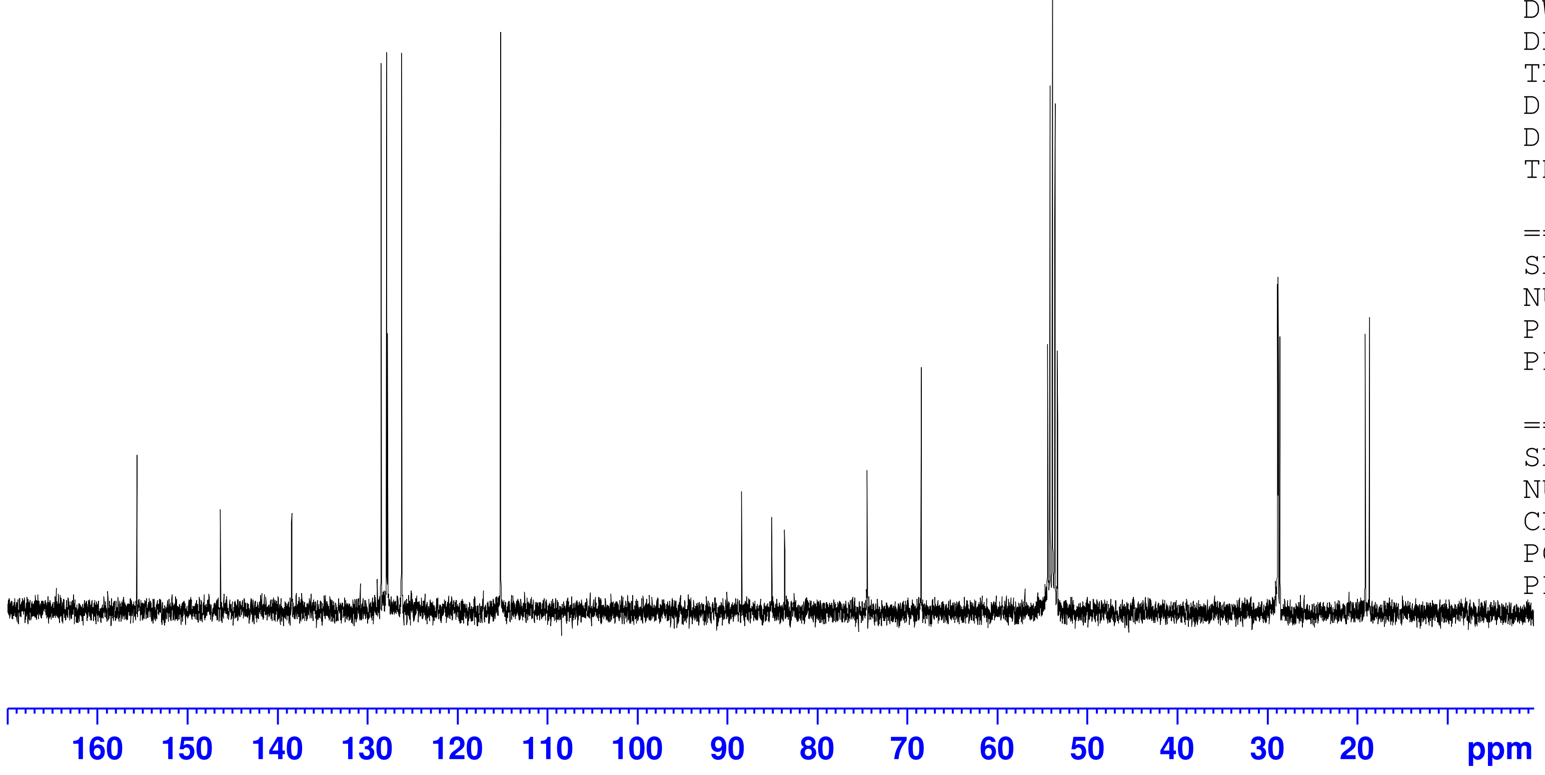


```
Current Data Parameters
NAME        qdy-20082-2 C
EXPNO                   2
PROCNO                  1

F2 - Acquisition Paramete
Date_            20160316
Time                21.30
INSTRUM             spect
PROBHD     5 mm PABBO BB/
PULPROG           zgpg30
TD                 65536
SOLVENT           CD2Cl2
NS                   116
DS                     0
SWH            24038.461 H
FIDRES          0.366798 H
AQ             1.3631488 s
RG                196.92
DW                20.800 u
DE                  6.50 u
TE                 297.5 K
D1            2.00000000 s
D11           0.03000000 s
TD0                    1

======== CHANNEL f1 =====
SFO1         100.6228298 M
NUC1                 13C
P1                  9.70 u
PLW1         46.98899841 W

======== CHANNEL f2 =====
SFO2         400.1316005 M
NUC2                  1H
CPDPRG[2         waltz16
PCPD2              90.00 u
PLW2         11.99499989 W
```

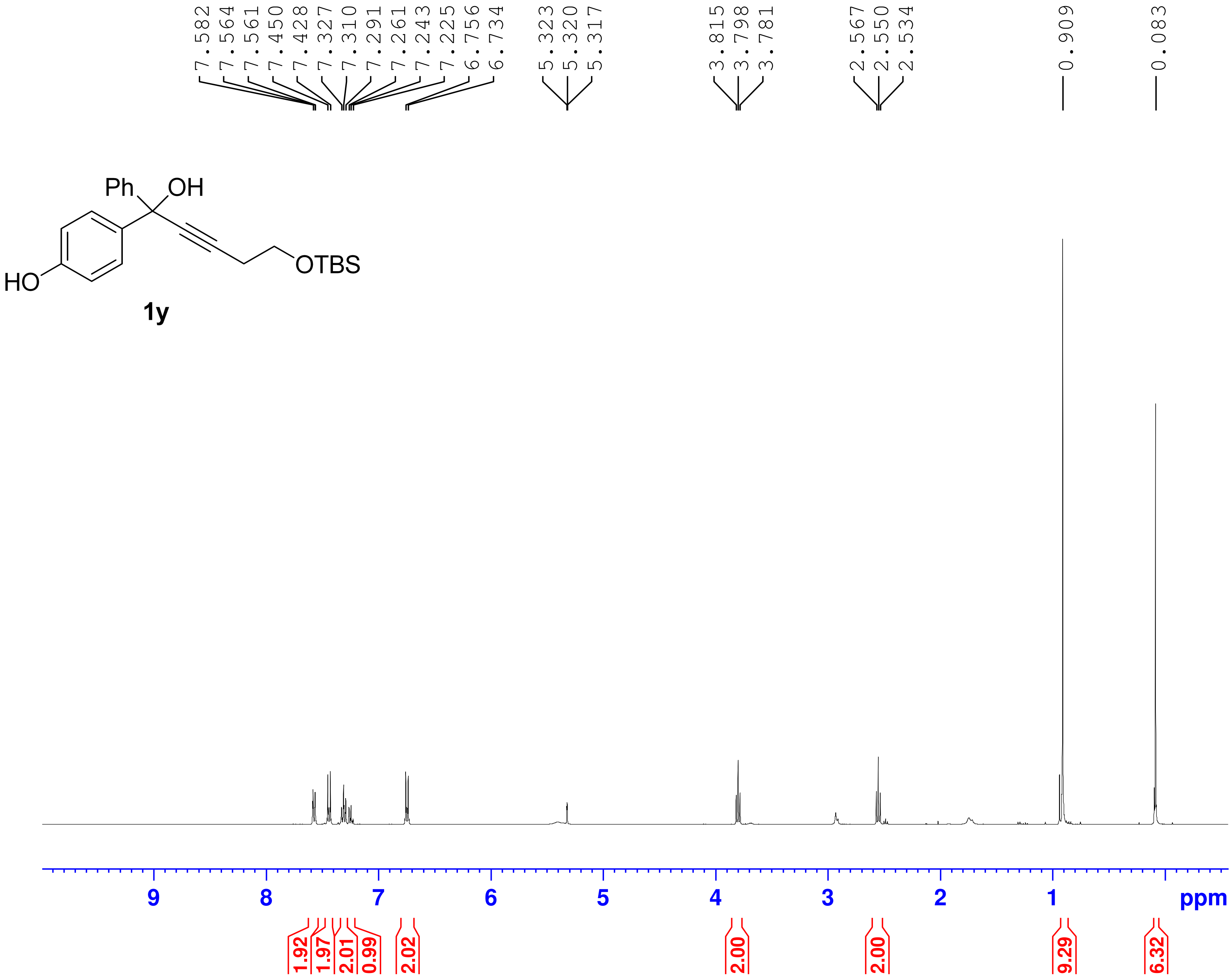

Current Data Parameters
NAME qdy-20085-1 H
EXPNO 2
PROCNO 1

F2 - Acquisition Parameters
Date_ 20160319
Time 19.52
INSTRUM spect
PROBHD 5 mm PABBO BB/
PULPROG zg30
TD 65536
SOLVENT CD2Cl2
NS 16
DS 0
SWH 8012.820 Hz
FIDRES 0.122266 Hz
AQ 4.0894465 sec
RG 82.92
DW 62.400 usec
DE 6.50 usec
TE 297.9 K
D1 1.00000000 sec
TD0 1

======== CHANNEL f1 ========
SFO1 400.1324710 MHz
NUC1 1H
P1 14.50 usec
PLW1 11.99499989 W

F2 - Processing parameters
SI 65536
SF 400.1300153 MHz
WDW EM
SSB 0
LB 0.30 Hz
GB 0
PC 1.00

Supplementary Figure 53. ^{1}H NMR spectrum for 1y

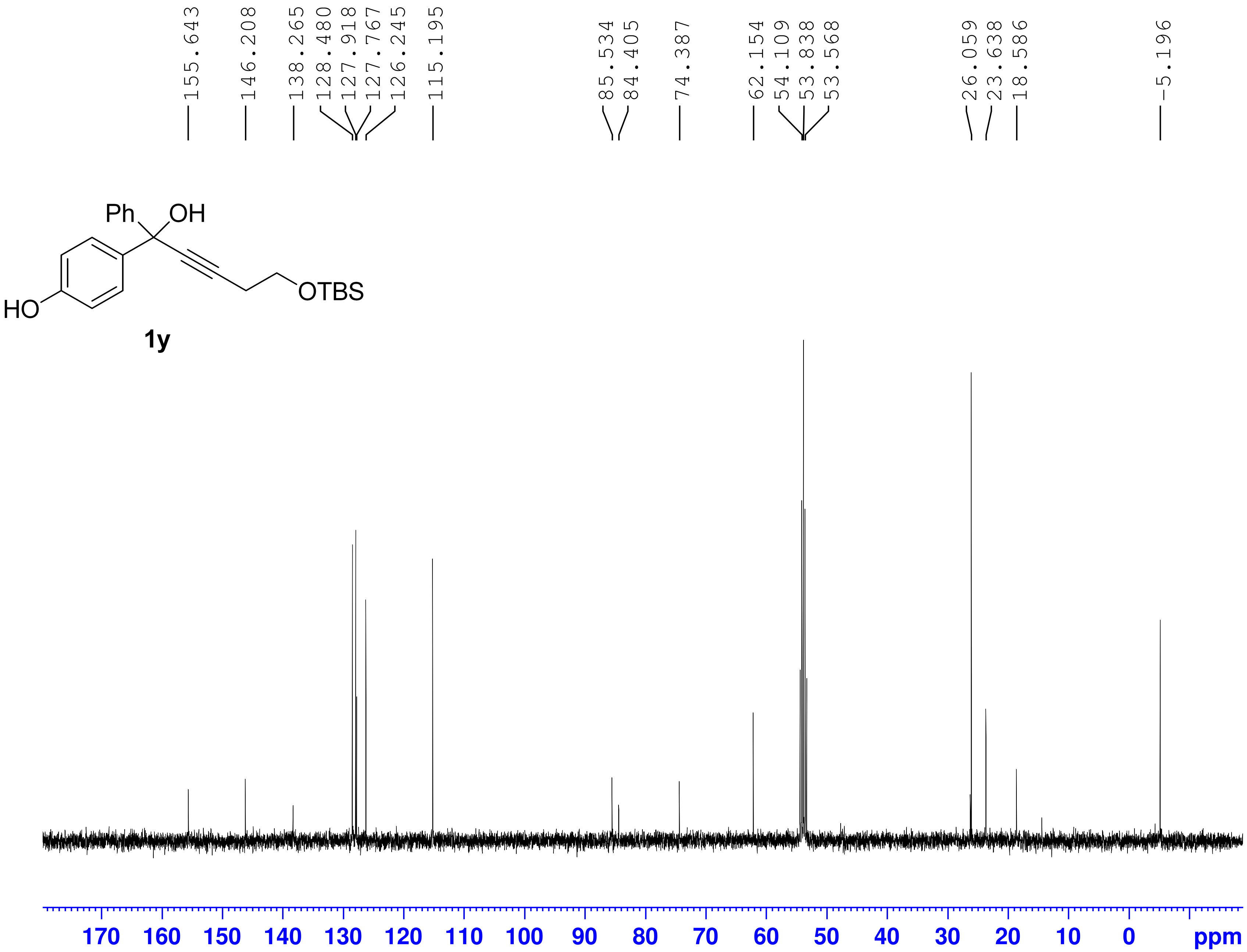


```
Current Data Parameters
NAME          qdy-20085-1 C
EXPNO                     1
PROCNO                    1

F2 - Acquisition Parameters
Date_              20160317
Time                  16.57
INSTRUM               spect
PROBHD     5 mm PABBO BB/
PULPROG             zgpg30
TD                    65536
SOLVENT              CD2Cl2
NS                       74
DS                        0
SWH              24038.461 Hz
FIDRES            0.366798 Hz
AQ               1.3631488 sec
RG                   196.92
DW                   20.800 usec
DE                     6.50 usec
TE                    297.5 K
D1               2.00000000 sec
D11              0.03000000 sec
TD0                       1

======== CHANNEL f1 ========
SFO1          100.6228298 MHz
NUC1                    13C
P1                     9.70 usec
PLW1            46.98899841 W

======== CHANNEL f2 ========
SFO2          400.1316005 MHz
NUC2                     1H
CPDPRG[2            waltz16
PCPD2                 90.00 usec
PLW2            11.99499989 W
PLW12            0.34213999 W
PLW13            0.27713001 W

F2 - Processing parameters
SI                    32768
SF            100.6127277 MHz
WDW                      EM
SSB        0
LB                     1.00 Hz
GB         0
PC                     1.40
```

Supplementary Figure 54. ^{13}C NMR spectrum for 1y

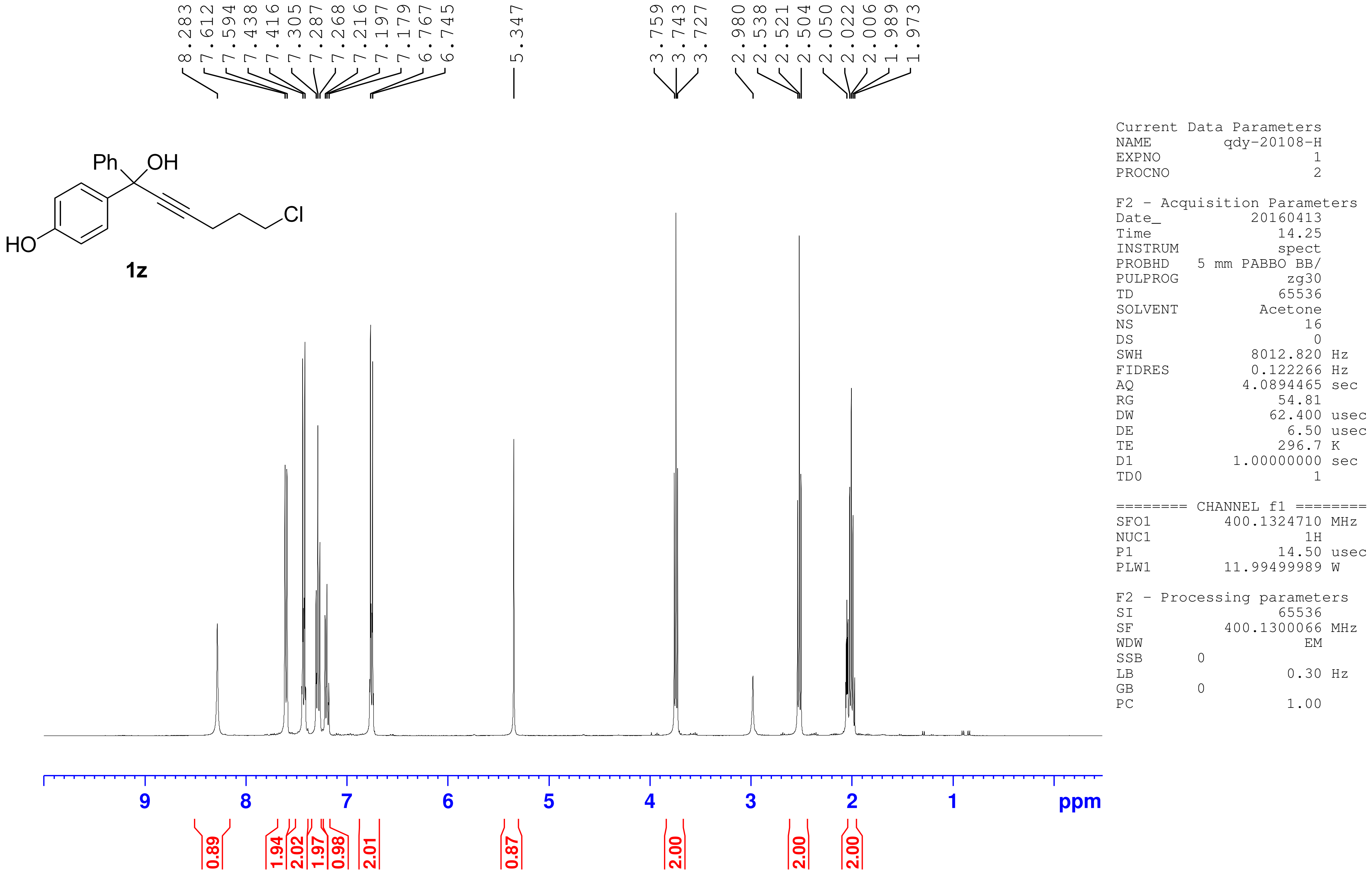

Supplementary Figure 55. ^{1}H NMR spectrum for 1z

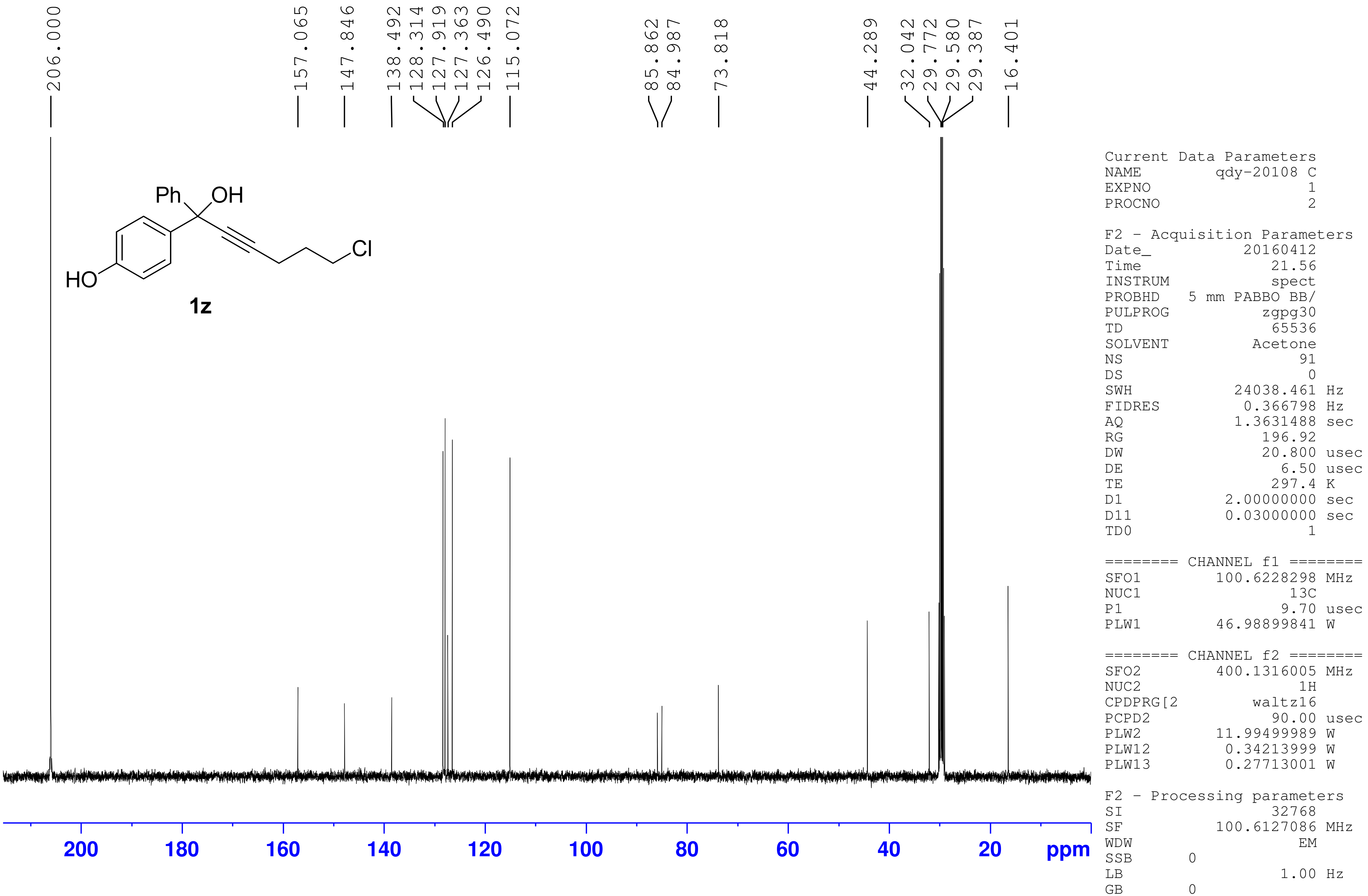

Supplementary Figure 56. ^{13}C NMR spectrum for 1z

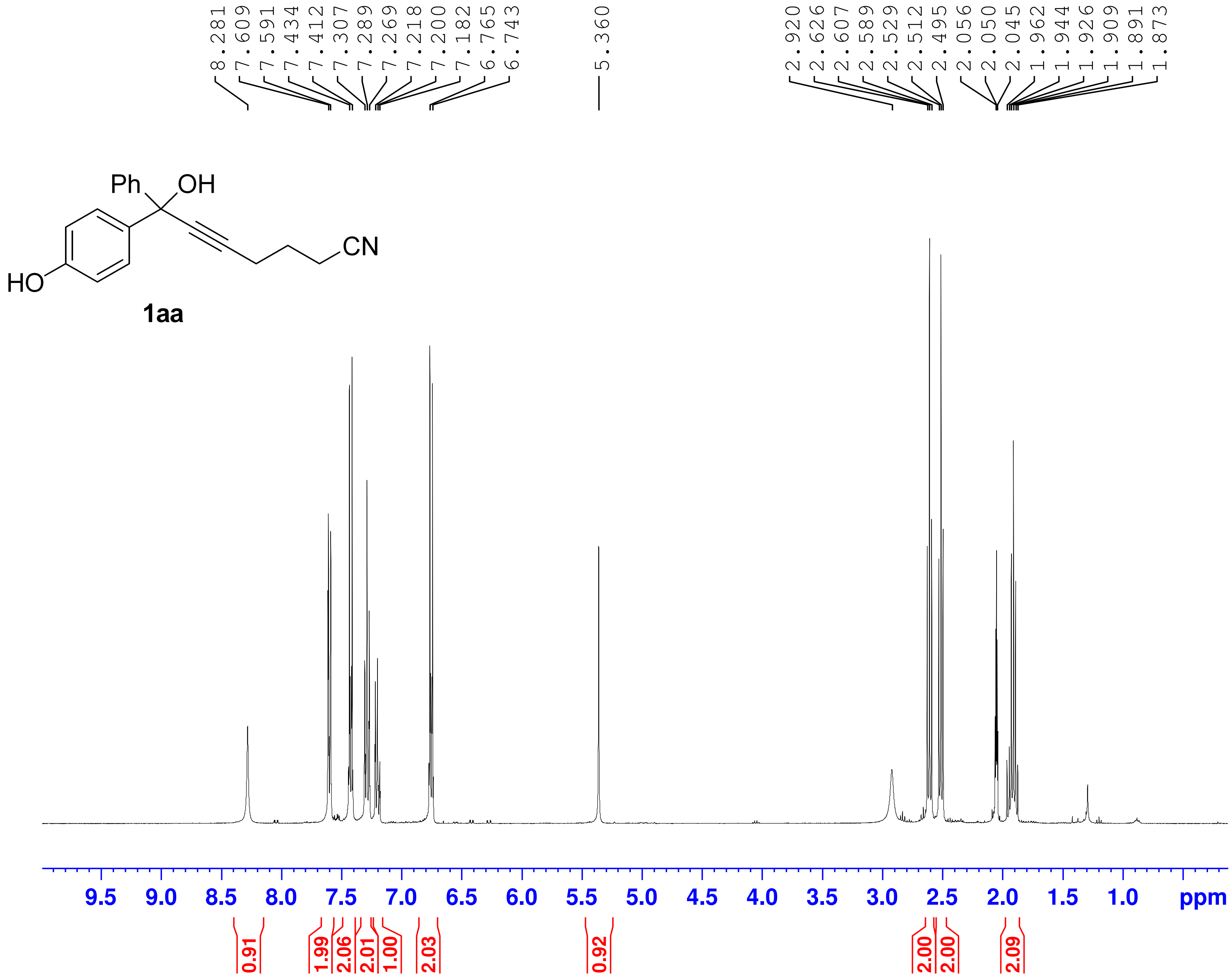


```
Current Data Parameters
NAME          qdy-20123 H
EXPNO                   2
PROCNO                  1

F2 - Acquisition Parameters
Date_            20160422
Time                22.51
INSTRUM              spect
PROBHD    5 mm PABBO BB/
PULPROG              zg30
TD                  65536
SOLVENT           Acetone
NS                      5
DS                      0
SWH             8012.820 Hz
FIDRES          0.122266 Hz
AQ             4.0894465 sec
RG                 112.31
DW                 62.400 usec
DE                   6.50 usec
TE                  297.5 K
D1            1.00000000 sec
TD0                     1

======== CHANNEL f1 ========
SFO1         400.1324710 MHz
NUC1                   1H
P1                  14.50 usec
PLW1         11.99499989 W

F2 - Processing parameters
SI                  65536
SF           400.1300068 MHz
WDW                    EM
SSB        0
LB                   0.30 Hz
GB         0
PC                   1.00
```

Supplementary Figure 57. ^{1}H NMR spectrum for 1aa

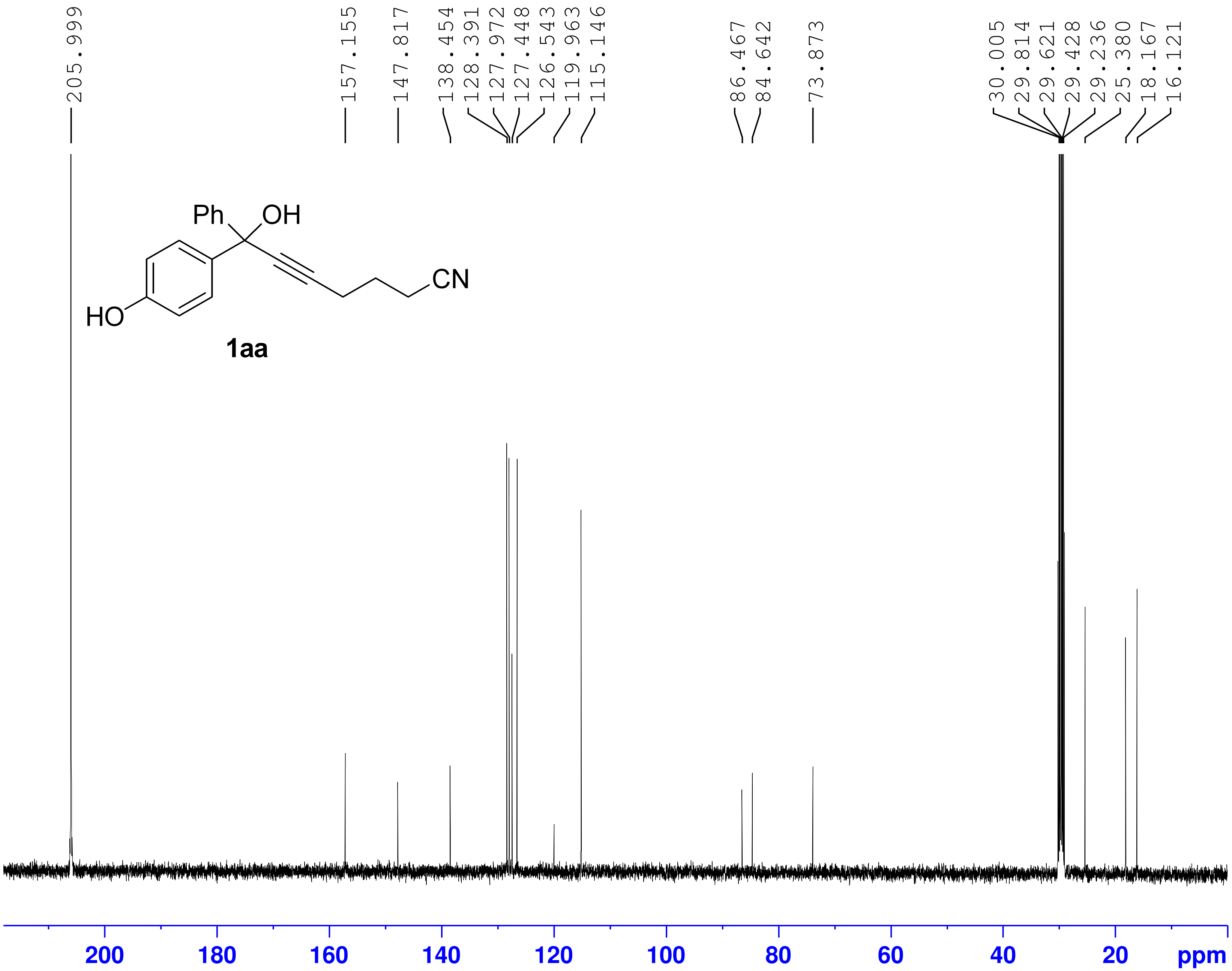


```
Current Data Parameters
NAME          qdy-20123 C
EXPNO                   2
PROCNO                  1

F2 - Acquisition Parameters
Date_            20160422
Time                22.56
INSTRUM             spect
PROBHD    5 mm PABBO BB/
PULPROG           zgpg30
TD                 65536
SOLVENT          Acetone
NS                   200
DS                     0
SWH           24038.461 Hz
FIDRES         0.366798 Hz
AQ            1.3631488 sec
RG                196.92
DW                20.800 usec
DE                  6.50 usec
TE                 298.5 K
D1            2.00000000 sec
D11           0.03000000 sec
TD0                    1

======== CHANNEL f1 ========
SFO1        100.6228298 MHz
NUC1                 13C
P1                  9.70 usec
PLW1         46.98899841 W

======== CHANNEL f2 ========
SFO2        400.1316005 MHz
NUC2                  1H
CPDPRG[2          waltz16
PCPD2              90.00 usec
PLW2         11.99499989 W
PLW12         0.34213999 W
PLW13         0.27713001 W

F2 - Processing parameters
SI                 32768
SF          100.6127022 MHz
WDW                   EM
SSB      0
LB                  1.00 Hz
GB       0
PC                  1.40
```

Supplementary Figure 58. ^{13}C NMR spectrum for 1aa

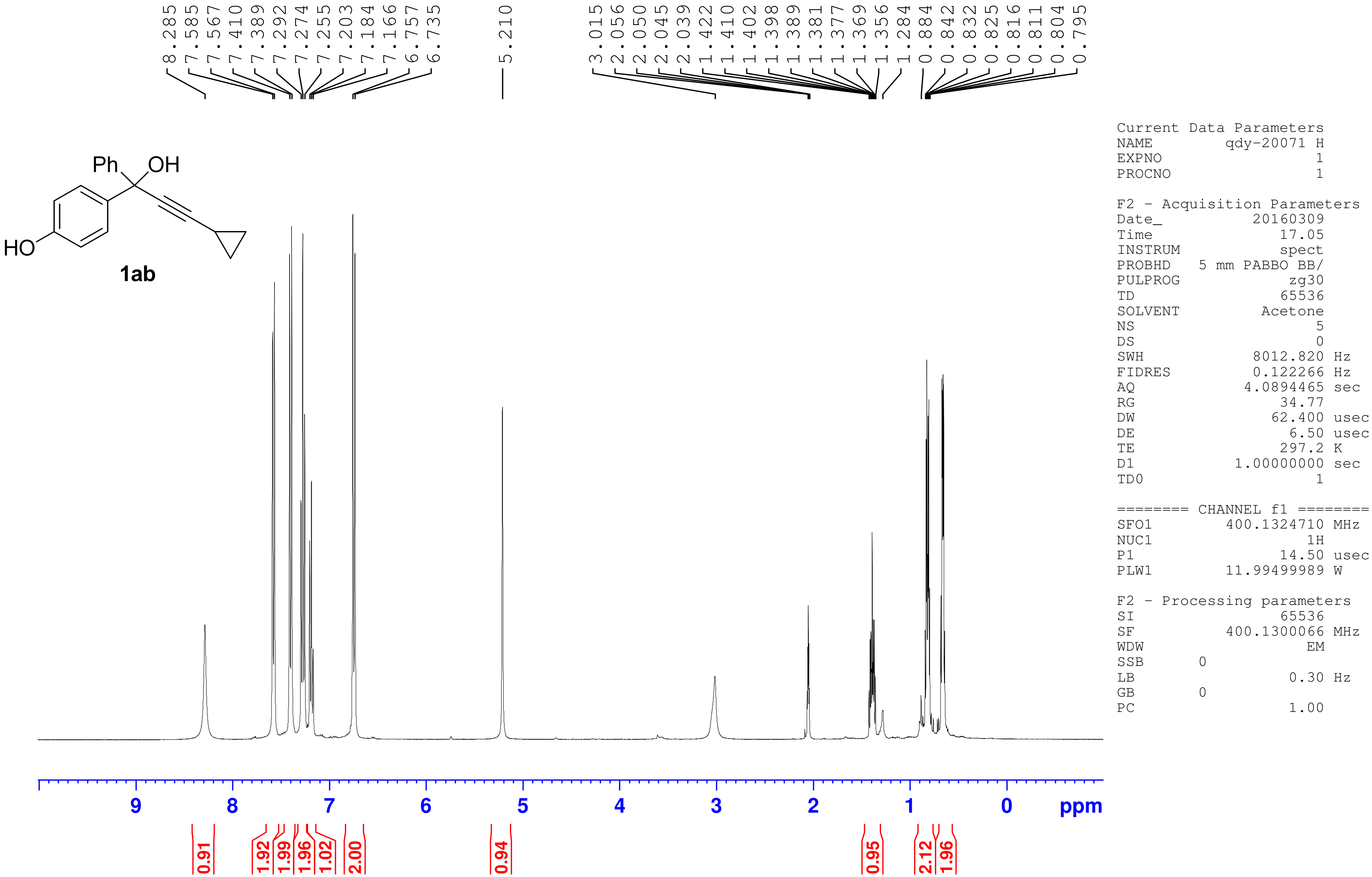

Supplementary Figure 59. ^{1}H NMR spectrum for 1ab

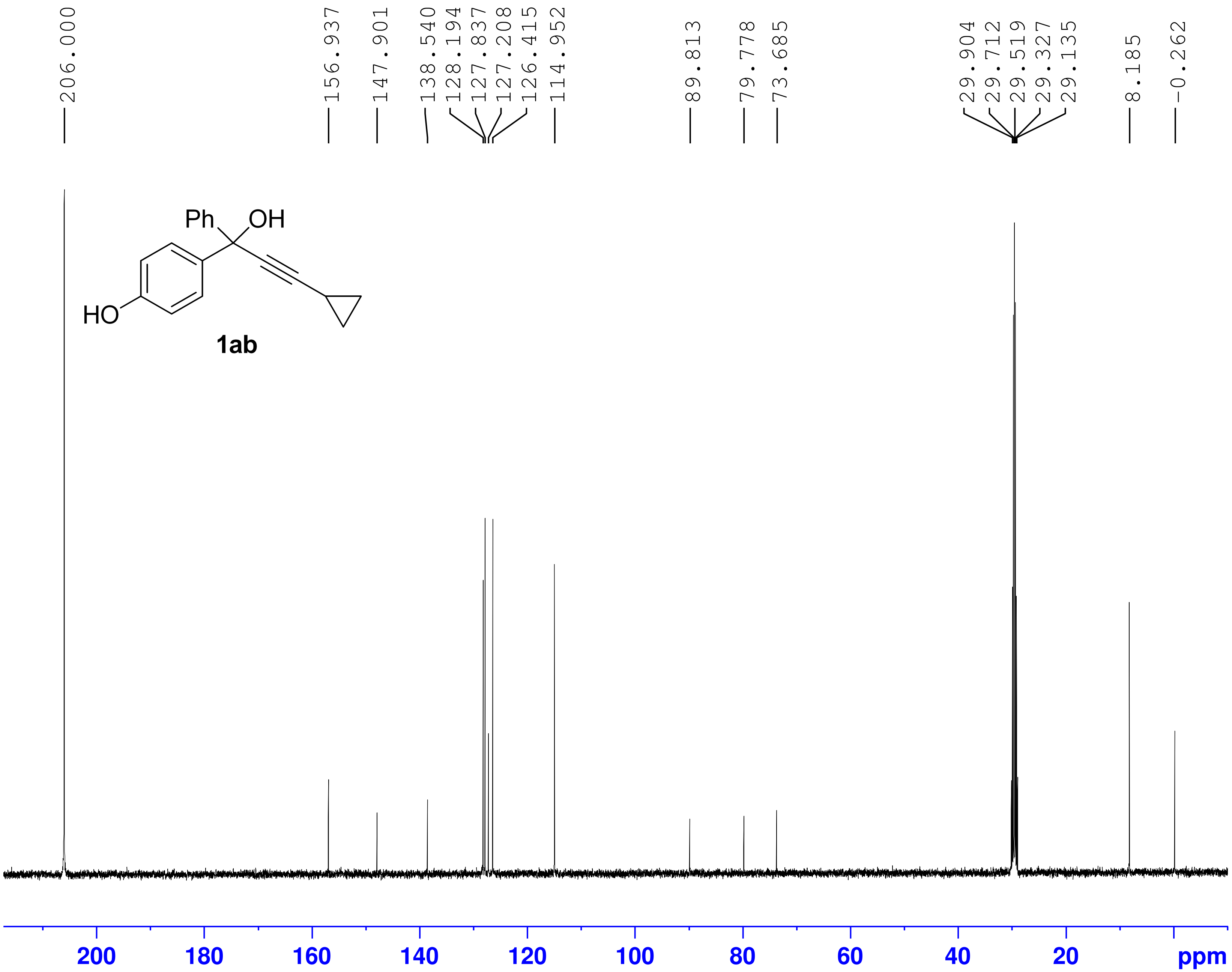

Current Data Parameters
NAME qdy-20071 C
EXPNO 1
PROCNO 1

F2 - Acquisition Parameters
Date_ 20160309
Time 17.01
INSTRUM spect
PROBHD 5 mm PABBO BB/
PULPROG zgpg30
TD 65536
SOLVENT Acetone
NS 96
DS 0
SWH 24038.461 Hz
FIDRES 0.366798 Hz
AQ 1.3631488 sec
RG 196.92
DW 20.800 usec
DE 6.50 usec
TE 297.7 K
D1 2.00000000 sec
D11 0.03000000 sec
TD0 1

======== CHANNEL f1 ========
SFO1 100.6228298 MHz
NUC1 13C
P1 9.70 usec
PLW1 46.98899841 W

======== CHANNEL f2 ========
SFO2 400.1316005 MHz
NUC2 1H
CPDPRG[2 waltz16
PCPD2 90.00 usec
PLW2 11.99499989 W
PLW12 0.34213999 W
PLW13 0.27713001 W

F2 - Processing parameters
SI 32768
SF 100.6127162 MHz
WDW EM
SSB 0
LB 1.00 Hz
GB 0
PC 1.40

Supplementary Figure 60. ^{13}C NMR spectrum for 1ab

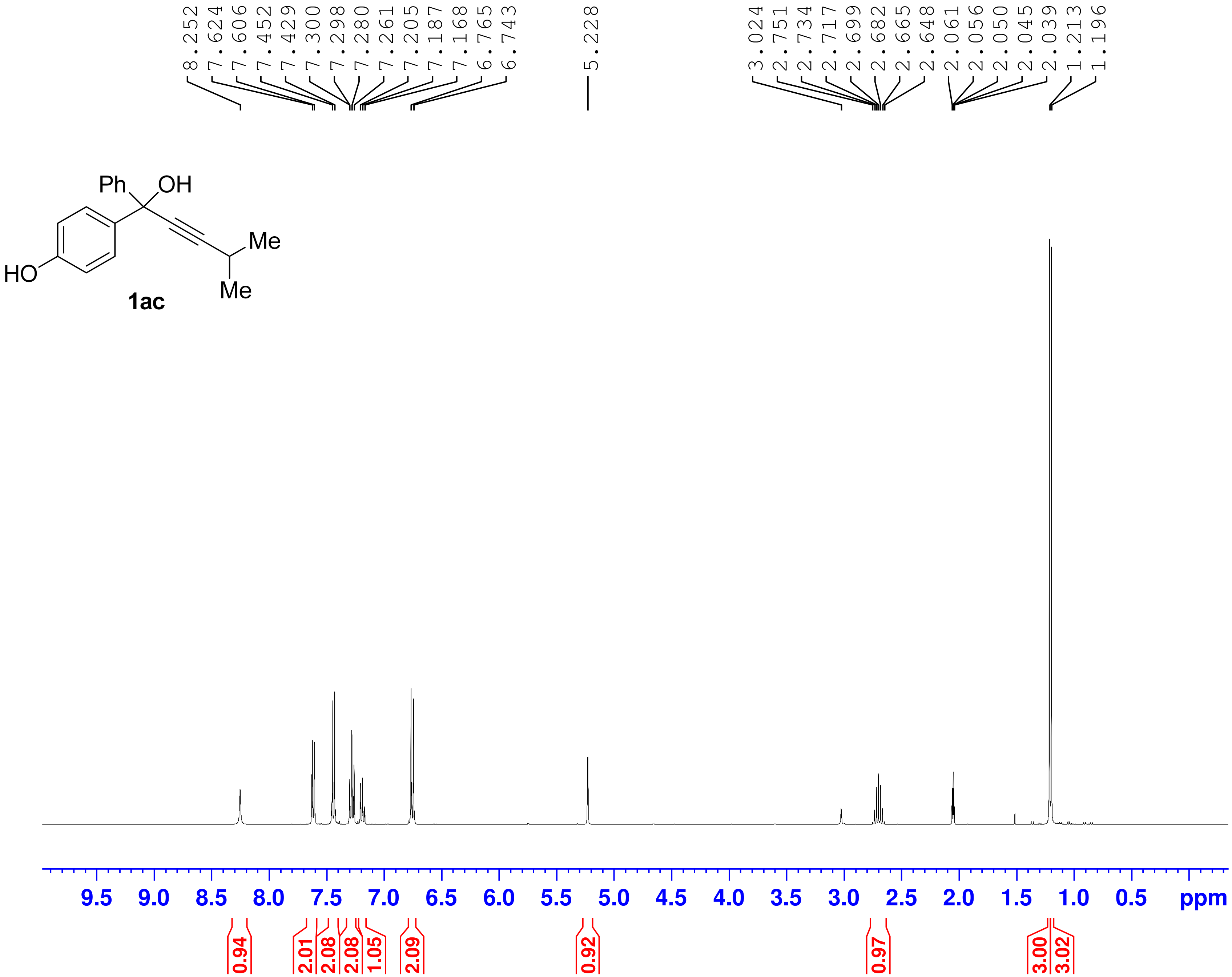


```
Current Data Parameters
NAME       qdy-20082-1 H
EXPNO                  5
PROCNO                 1

F2 - Acquisition Parameters
Date_           20160324
Time               20.16
INSTRUM            spect
PROBHD    5 mm PABBO BB/
PULPROG             zg30
TD                 65536
SOLVENT          Acetone
NS                     7
DS                     0
SWH             8012.820 Hz
FIDRES          0.122266 Hz
AQ             4.0894465 sec
RG                 70.97
DW                62.400 usec
DE                  6.50 usec
TE                 297.1 K
D1            1.00000000 sec
TD0                    1

======== CHANNEL f1 ========
SFO1         400.1324710 MHz
NUC1                  1H
P1                 14.50 usec
PLW1         11.99499989 W

F2 - Processing parameters
SI                 65536
SF           400.1300067 MHz
WDW                   EM
SSB      0
LB                  0.30 Hz
GB       0
PC                  1.00
```

Supplementary Figure 61. ^{1}H NMR spectrum for 1ac

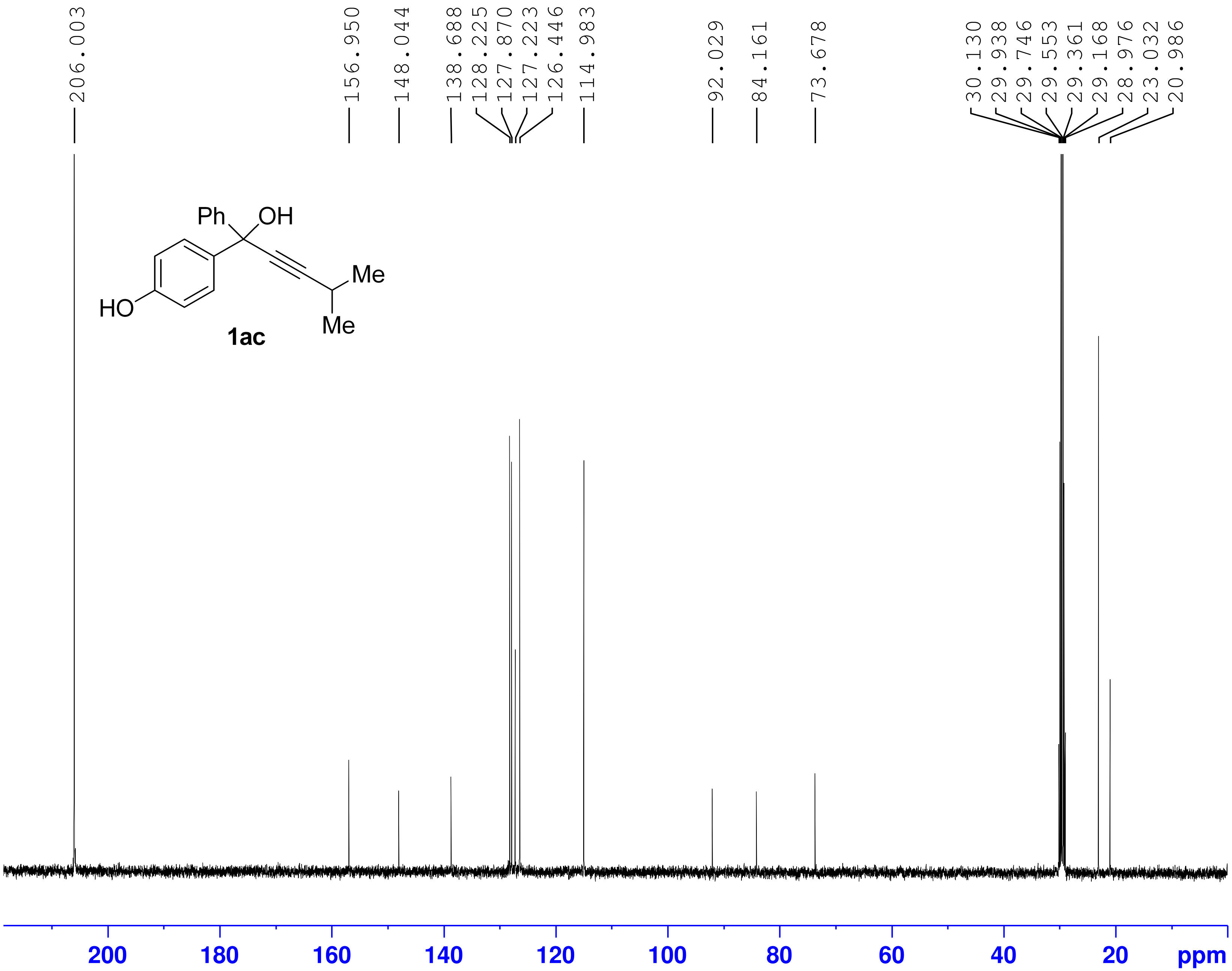

Current Data Parameters
NAME qdy-20082-1 C
EXPNO 2
PROCNO 1

F2 - Acquisition Parameters
Date_ 20160324
Time 20.07
INSTRUM spect
PROBHD 5 mm PABBO BB/
PULPROG zgpg30
TD 65536
SOLVENT Acetone
NS 108
DS 0
SWH 24038.461 Hz
FIDRES 0.366798 Hz
AQ 1.3631488 sec
RG 196.92
DW 20.800 usec
DE 6.50 usec
TE 297.5 K
D1 2.00000000 sec
D11 0.03000000 sec
TD0 1

======== CHANNEL f1 ========
SFO1 100.6228298 MHz
NUC1 13C
P1 9.70 usec
PLW1 46.98899841 W

======== CHANNEL f2 ========
SFO2 400.1316005 MHz
NUC2 1H
CPDPRG[2 waltz16
PCPD2 90.00 usec
PLW2 11.99499989 W
PLW12 0.34213999 W
PLW13 0.27713001 W

F2 - Processing parameters
SI 32768
SF 100.6127125 MHz
WDW EM
SSB 0
LB 1.00 Hz
GB 0
PC 1.40

Supplementary Figure 62. ^{13}C NMR spectrum for 1ac

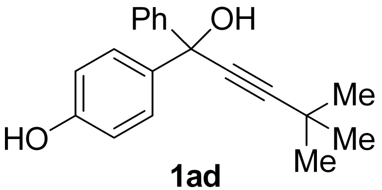

1ad

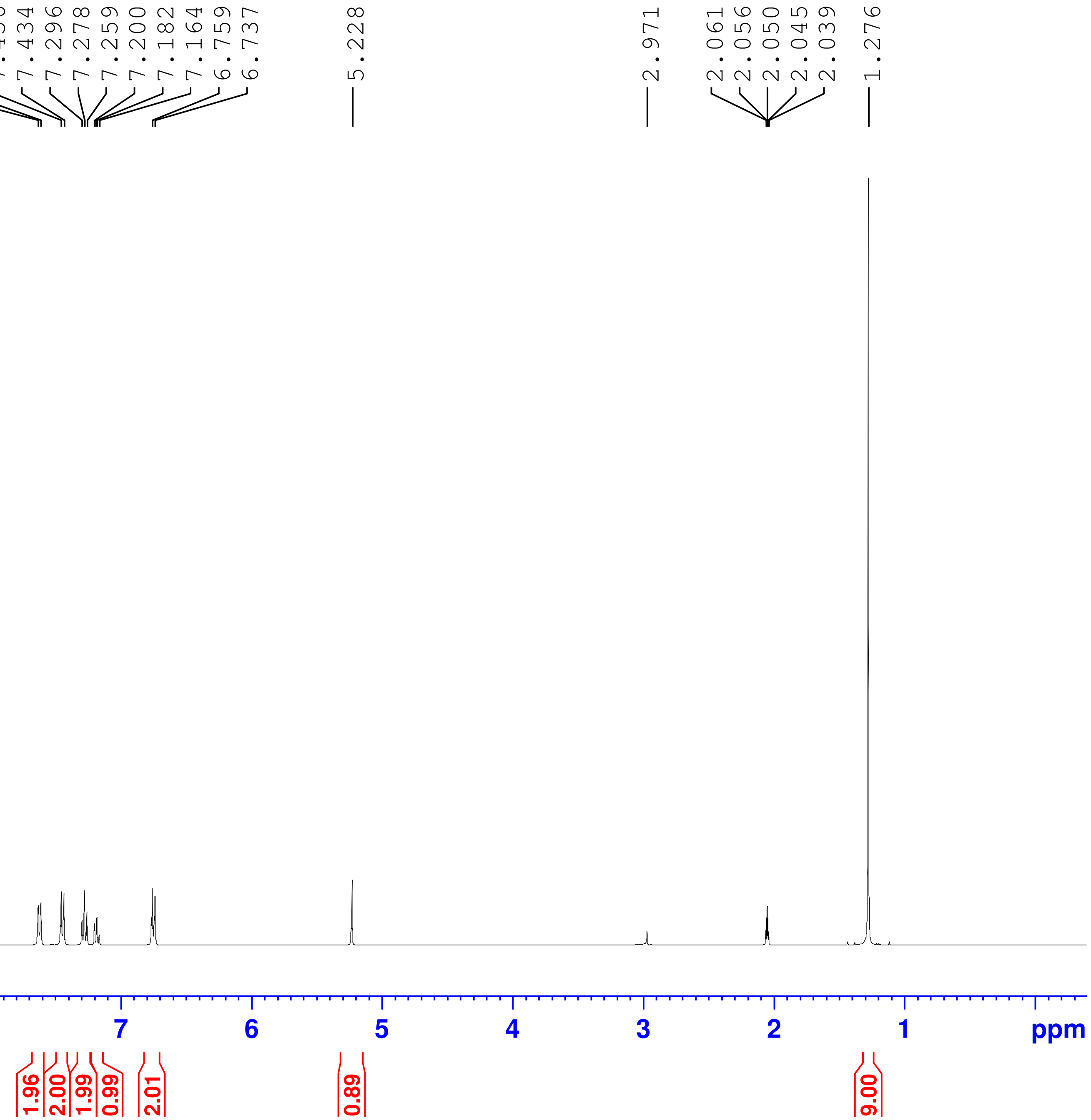

Current Data Parameters
NAME qdy-10194 H
EXPNO 2
PROCNO 1

F2 - Acquisition Parameters
Date_ 20160328
Time 21.55
INSTRUM spect
PROBHD 5 mm PABBO BB/
PULPROG zg30
TD 65536
SOLVENT Acetone
NS 7
DS 0
SWH 8012.820 Hz
FIDRES 0.122266 Hz
AQ 4.0894465 sec
RG 62.93
DW 62.400 usec
DE 6.50 usec
TE 297.3 K
D1 1.00000000 sec
TD0 1

======== CHANNEL f1 ========
SFO1 400.1324710 MHz
NUC1 1H
P1 14.50 usec
PLW1 11.99499989 W

F2 - Processing parameters
SI 65536
SF 400.1300066 MHz
WDW EM
SSB 0
LB 0.30 Hz
GB 0
PC 1.00

Supplementary Figure 63. ^{1}H NMR spectrum for 1ad

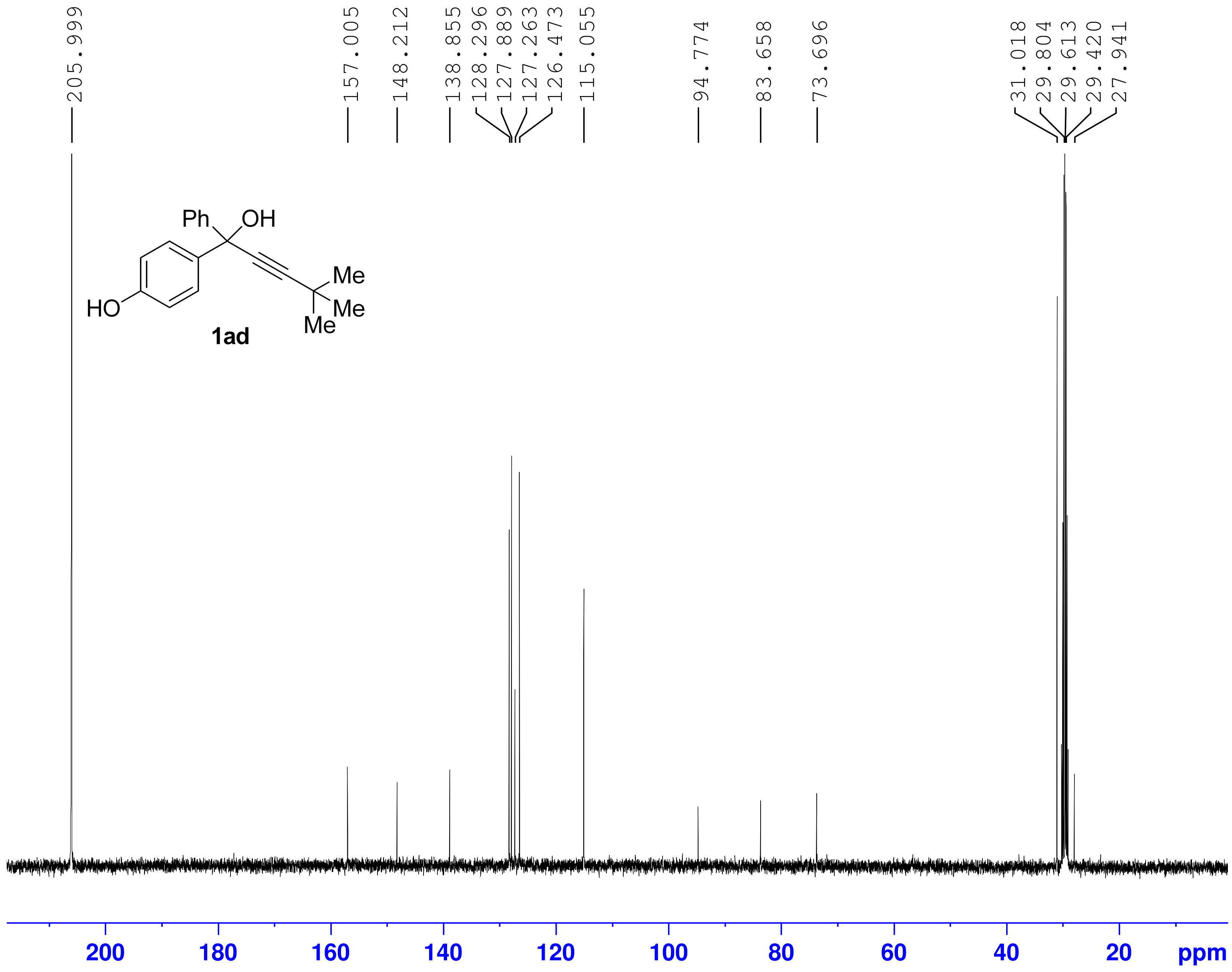

Current Data Parameters
NAME qdy-10194 C
EXPNO 2
PROCNO 1

F2 - Acquisition Parameters
Date_ 20160328
Time 22.02
INSTRUM spect
PROBHD 5 mm PABBO BB/
PULPROG zgpg30
TD 65536
SOLVENT Acetone
NS 130
DS 0
SWH 24038.461 Hz
FIDRES 0.366798 Hz
AQ 1.3631488 sec
RG 196.92
DW 20.800 usec
DE 6.50 usec
TE 298.4 K
D1 2.00000000 sec
D11 0.03000000 sec
TD0 1

======== CHANNEL f1 ========
SFO1 100.6228298 MHz
NUC1 13C
P1 9.70 usec
PLW1 46.98899841 W

======== CHANNEL f2 ========
SFO2 400.1316005 MHz
NUC2 1H
CPDPRG[2 waltz16
PCPD2 90.00 usec
PLW2 11.99499989 W
PLW12 0.34213999 W
PLW13 0.27713001 W

F2 - Processing parameters
SI 32768
SF 100.6127049 MHz
WDW EM
SSB 0
LB 1.00 Hz
GB 0
PC 1.40

Supplementary Figure 64. ^{13}C NMR spectrum for 1ad

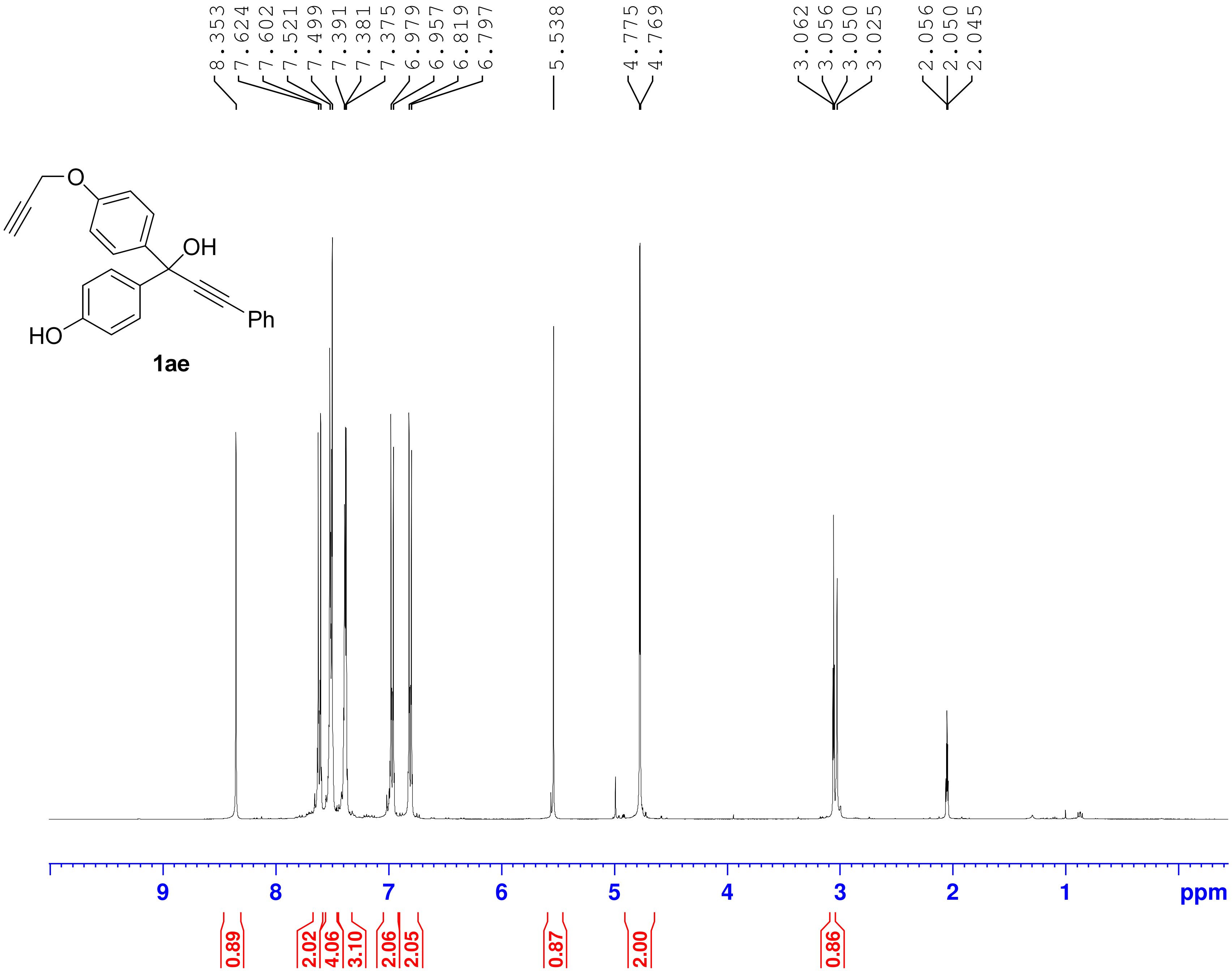


```
Current Data Parameters
NAME          qdy-20068 H
EXPNO                   2
PROCNO                  1

F2 - Acquisition Parameters
Date_            20160310
Time                19.40
INSTRUM             spect
PROBHD    5 mm PABBO BB/
PULPROG              zg30
TD                  65536
SOLVENT           Acetone
NS                     12
DS                      0
SWH              8012.820 Hz
FIDRES           0.122266 Hz
AQ              4.0894465 sec
RG                  49.32
DW                 62.400 usec
DE                   6.50 usec
TE                  296.5 K
D1             1.00000000 sec
TD0                     1

======== CHANNEL f1 ========
SFO1          400.1324710 MHz
NUC1                   1H
P1                  14.50 usec
PLW1          11.99499989 W

F2 - Processing parameters
SI                  65536
SF            400.1300066 MHz
WDW                    EM
SSB       0
LB                   0.30 Hz
GB        0
PC                   1.00
```

Supplementary Figure 65. ^{1}H NMR spectrum for 1ae

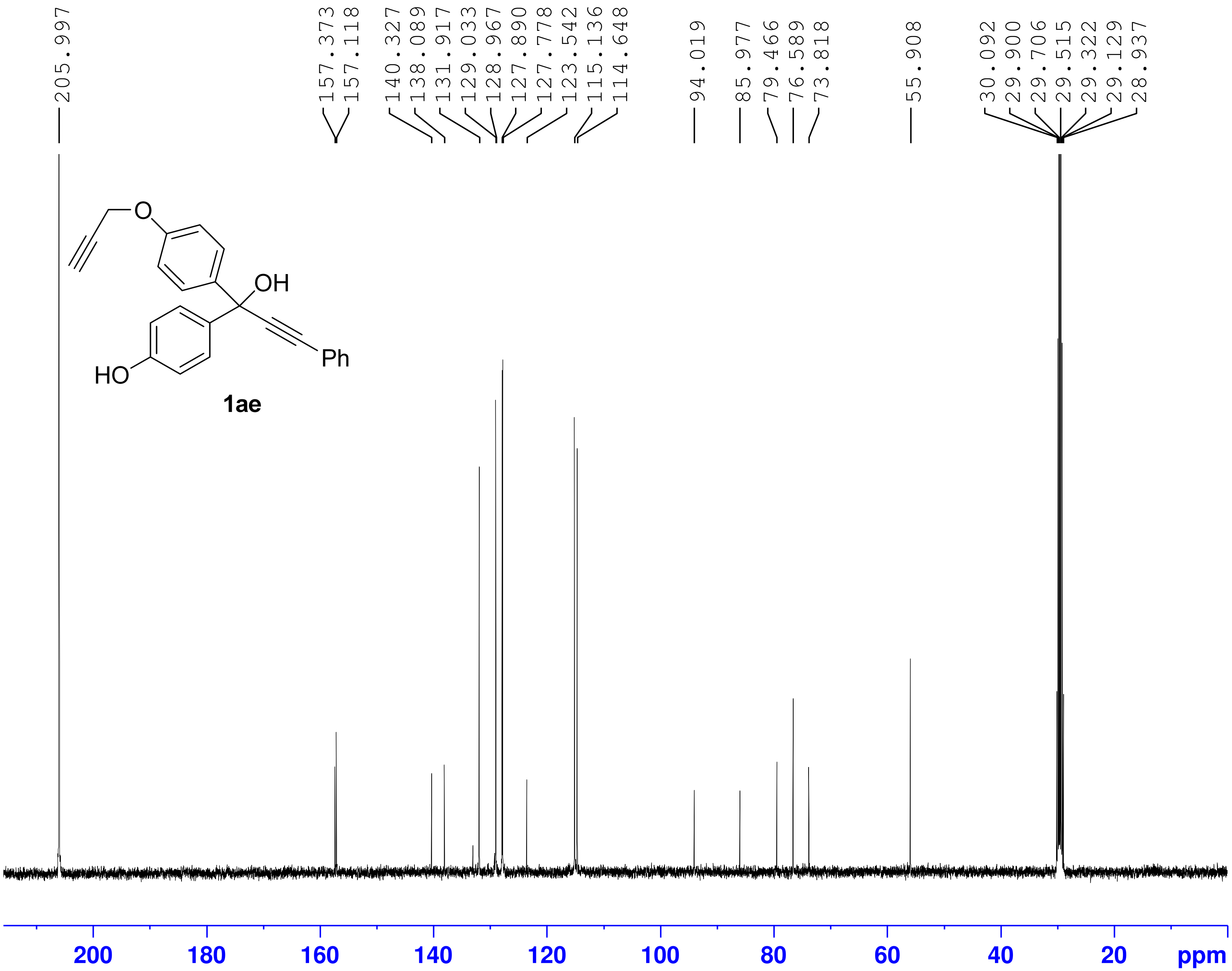


```
Current Data Parameters
NAME          qdy-20068 C
EXPNO                   2
PROCNO                  1

F2 - Acquisition Parameters
Date_            20160310
Time                19.46
INSTRUM             spect
PROBHD    5 mm PABBO BB/
PULPROG           zgpg30
TD                 65536
SOLVENT          Acetone
NS                   115
DS                     0
SWH            24038.461 Hz
FIDRES          0.366798 Hz
AQ             1.3631488 sec
RG                196.92
DW                20.800 usec
DE                  6.50 usec
TE                 297.5 K
D1            2.00000000 sec
D11           0.03000000 sec
TD0                    1

======== CHANNEL f1 ========
SFO1         100.6228298 MHz
NUC1                 13C
P1                  9.70 usec
PLW1         46.98899841 W

======== CHANNEL f2 ========
SFO2         400.1316005 MHz
NUC2                  1H
CPDPRG[2         waltz16
PCPD2              90.00 usec
PLW2         11.99499989 W
PLW12         0.34213999 W
PLW13         0.27713001 W

F2 - Processing parameters
SI                 32768
SF           100.6127177 MHz
WDW                   EM
SSB       0
LB                  1.00 Hz
GB        0
PC                  1.40
```

Supplementary Figure 66. ^{13}C NMR spectrum for 1ae

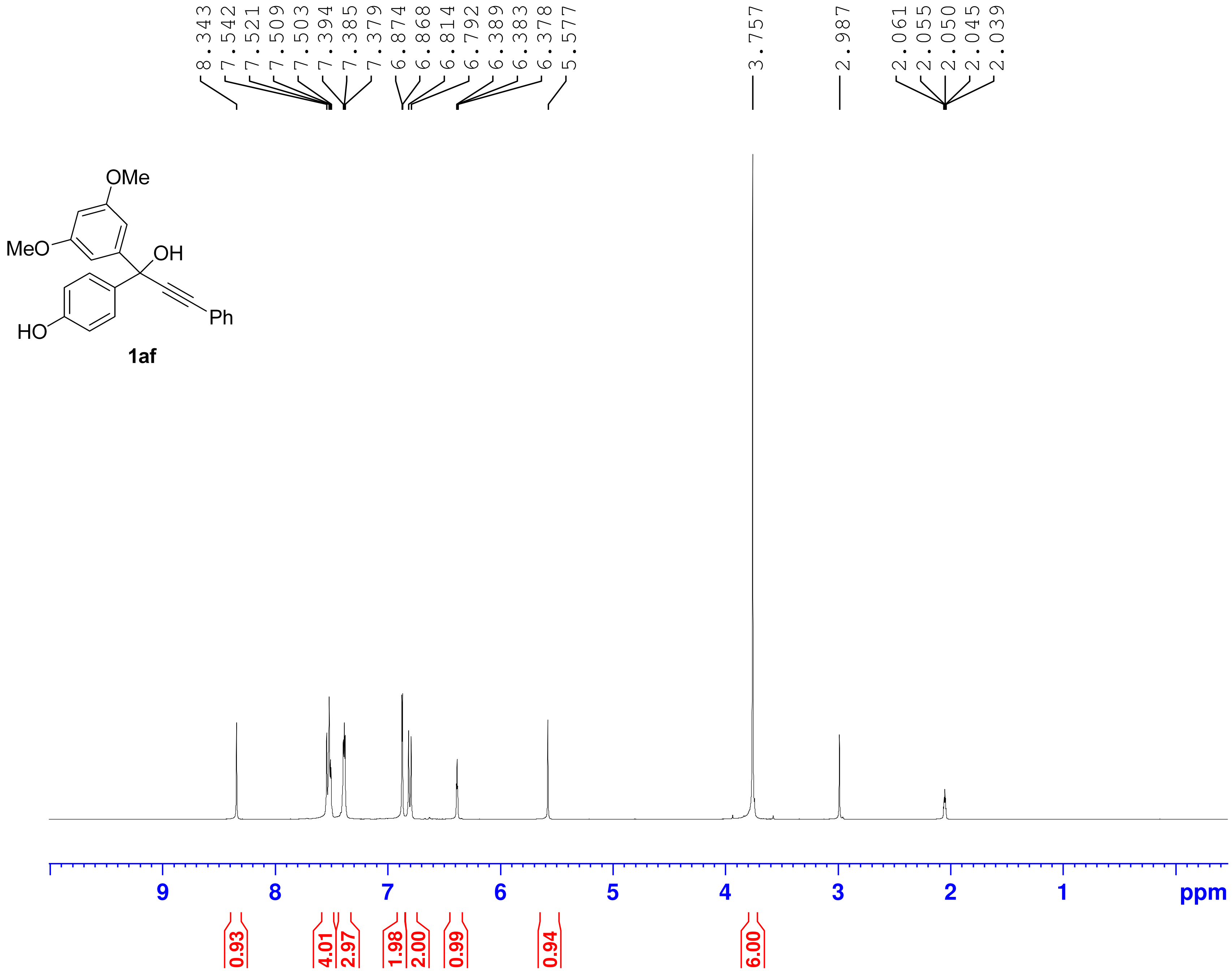

Current Data Parameters	
NAME	qdy-20126 H
EXPNO	1
PROCNO	1
F2 - Acquisition Parameters	
Date_	20160428
Time	19.38
INSTRUM	spect
PROBHD	5 mm PABBO BB/
PULPROG	zg30
TD	65536
SOLVENT	Acetone
NS	8
DS	0
SWH	8012.820 Hz
FIDRES	0.122266 Hz
AQ	4.0894465 sec
RG	62.93
DW	62.400 usec
DE	6.50 usec
TE	297.0 K
D1	1.00000000 sec
TD0	1
======== CHANNEL f1 ========	
SFO1	400.1324710 MHz
NUC1	1H
P1	14.50 usec
PLW1	11.99499989 W
F2 - Processing parameters	
SI	65536
SF	400.1300065 MHz
WDW	EM
SSB	0
LB	0.30 Hz
GB	0
PC	1.00

Supplementary Figure 67. ^{1}H NMR spectrum for 1af

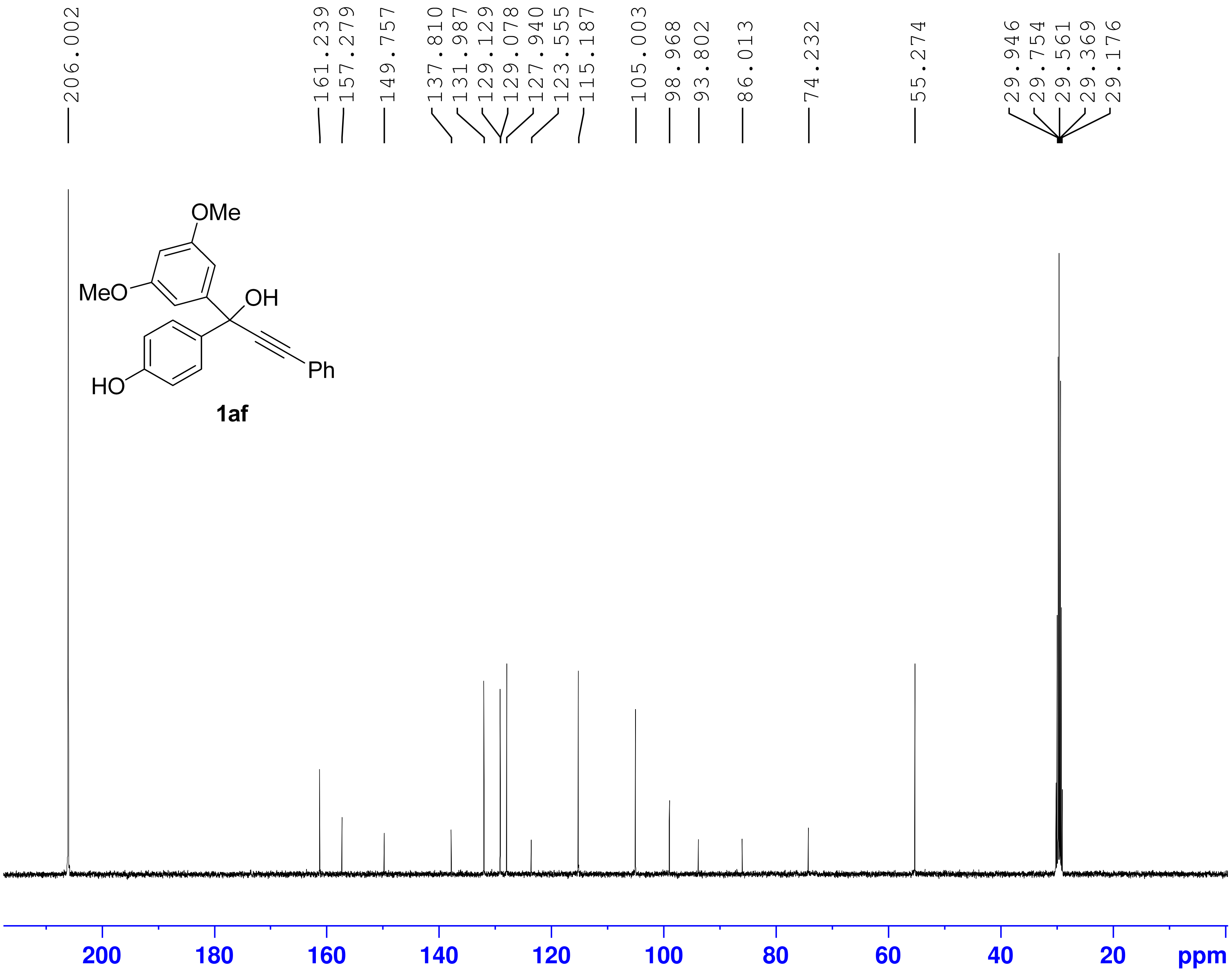

Current Data Parameters
NAME qdy-20126 C
EXPNO 1
PROCNO 1

F2 - Acquisition Parameters
Date_ 20160428
Time 19.42
INSTRUM spect
PROBHD 5 mm PABBO BB/
PULPROG zgpg30
TD 65536
SOLVENT Acetone
NS 105
DS 0
SWH 24038.461 Hz
FIDRES 0.366798 Hz
AQ 1.3631488 sec
RG 196.92
DW 20.800 usec
DE 6.50 usec
TE 297.8 K
D1 2.00000000 sec
D11 0.03000000 sec
TD0 1

======== CHANNEL f1 ========
SFO1 100.6228298 MHz
NUC1 13C
P1 9.70 usec
PLW1 46.98899841 W

======== CHANNEL f2 ========
SFO2 400.1316005 MHz
NUC2 1H
CPDPRG[2 waltz16
PCPD2 90.00 usec
PLW2 11.99499989 W
PLW12 0.34213999 W
PLW13 0.27713001 W

F2 - Processing parameters
SI 32768
SF 100.6127103 MHz
WDW EM
SSB 0
LB 1.00 Hz
GB 0
PC 1.40

Supplementary Figure 68. ^{13}C NMR spectrum for 1af

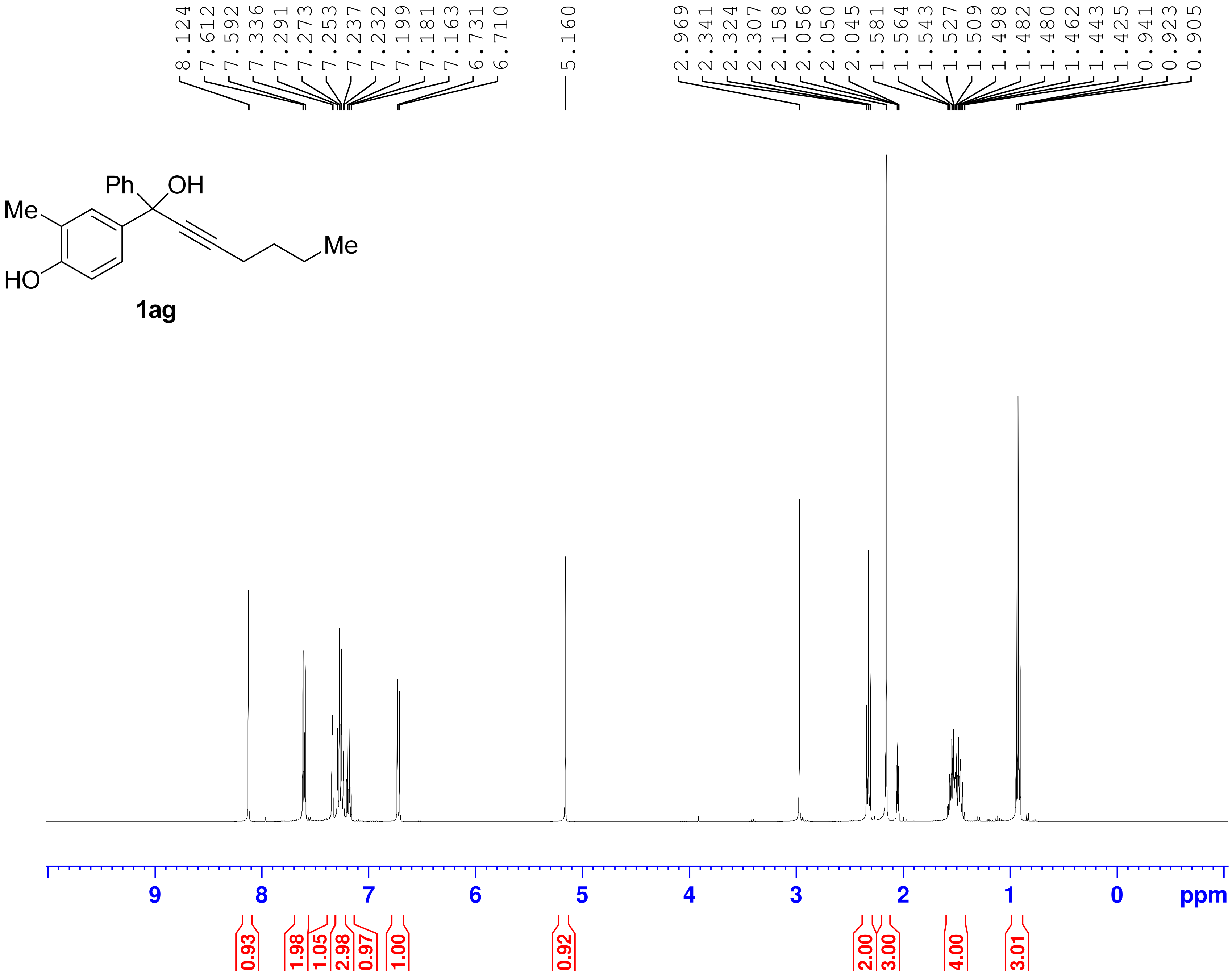


```
Current Data Parameters
NAME          qdy-20136 H
EXPNO                   1
PROCNO                  1

F2 - Acquisition Parameters
Date_            20160504
Time                22.47
INSTRUM             spect
PROBHD   5 mm PABBO BB/
PULPROG              zg30
TD                  65536
SOLVENT           Acetone
NS                      4
DS                      0
SWH              8012.820 Hz
FIDRES           0.122266 Hz
AQ              4.0894465 sec
RG                  49.32
DW                 62.400 usec
DE                   6.50 usec
TE                  298.0 K
D1             1.00000000 sec
TD0                     1

======== CHANNEL f1 ========
SFO1          400.1324710 MHz
NUC1                   1H
P1                  14.50 usec
PLW1          11.99499989 W

F2 - Processing parameters
SI                  65536
SF            400.1300068 MHz
WDW                    EM
SSB      0
LB                   0.30 Hz
GB       0
PC                   1.00
```

Supplementary Figure 69. ^{1}H NMR spectrum for 1ag

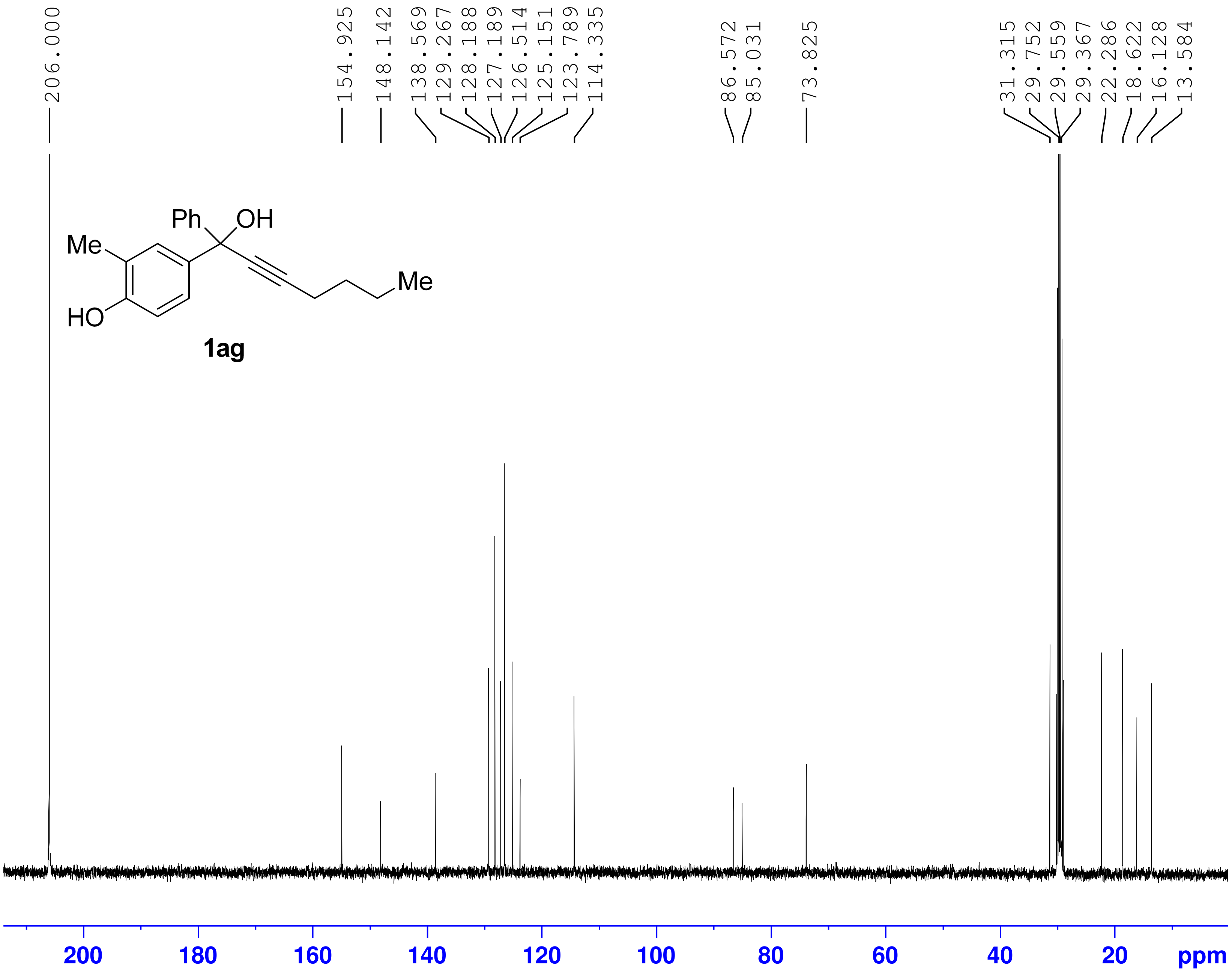

Current Data Parameters
NAME qdy-20136 C
EXPNO 1
PROCNO 1

F2 - Acquisition Parameters
Date_ 20160504
Time 22.39
INSTRUM spect
PROBHD 5 mm PABBO BB/
PULPROG zgpg30
TD 65536
SOLVENT Acetone
NS 95
DS 0
SWH 24038.461 Hz
FIDRES 0.366798 Hz
AQ 1.3631488 sec
RG 196.92
DW 20.800 usec
DE 6.50 usec
TE 298.3 K
D1 2.00000000 sec
D11 0.03000000 sec
TD0 1

======== CHANNEL f1 ========
SFO1 100.6228298 MHz
NUC1 13C
P1 9.70 usec
PLW1 46.98899841 W

======== CHANNEL f2 ========
SFO2 400.1316005 MHz
NUC2 1H
CPDPRG[2 waltz16
PCPD2 90.00 usec
PLW2 11.99499989 W
PLW12 0.34213999 W
PLW13 0.27713001 W

F2 - Processing parameters
SI 32768
SF 100.6127103 MHz
WDW EM
SSB 0
LB 1.00 Hz
GB 0
PC 1.40

Supplementary Figure 70. ^{13}C NMR spectrum for 1ag

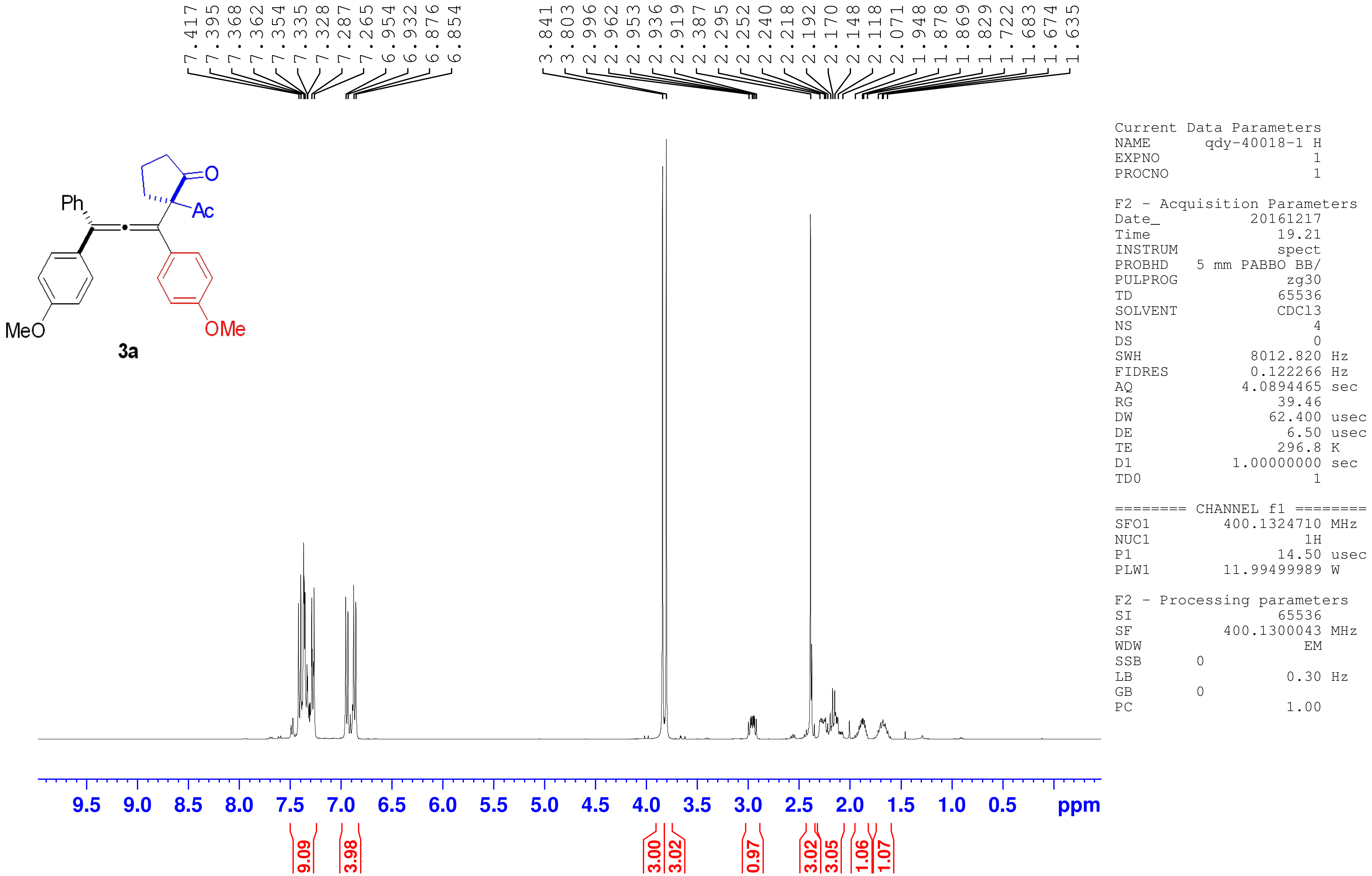

Supplementary Figure 71. ^{1}H NMR spectrum for 3a

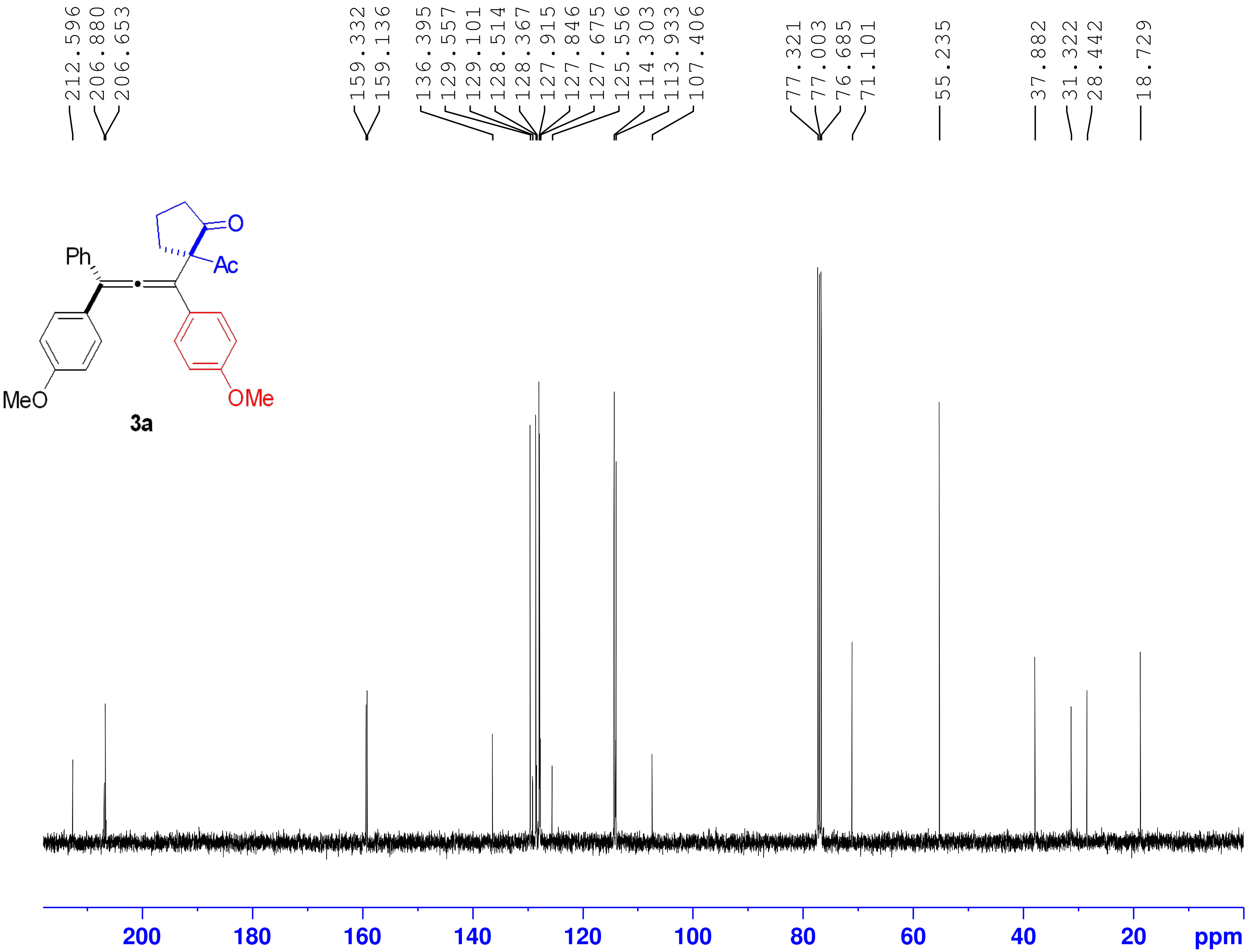


```
Current Data Parameters
NAME        qdy-40018-1 C
EXPNO                   1
PROCNO                  1

F2 - Acquisition Parameters
Date_            20161217
Time                19.23
INSTRUM             spect
PROBHD   5 mm PABBO BB/
PULPROG           zgpg30
TD                 65536
SOLVENT            CDCl3
NS                    50
DS                     0
SWH            24038.461 Hz
FIDRES          0.366798 Hz
AQ             1.3631488 sec
RG                196.92
DW                20.800 usec
DE                  6.50 usec
TE                 297.6 K
D1            2.00000000 sec
D11           0.03000000 sec
TD0                    1

======== CHANNEL f1 ========
SFO1         100.6228298 MHz
NUC1                 13C
P1                  9.70 usec
PLW1         46.98899841 W

======== CHANNEL f2 ========
SFO2         400.1316005 MHz
NUC2                  1H
CPDPRG[2         waltz16
PCPD2              90.00 usec
PLW2         11.99499989 W
PLW12         0.34213999 W
PLW13         0.27713001 W

F2 - Processing parameters
SI                 32768
SF           100.6127780 MHz
WDW                   EM
SSB      0
LB                  1.00 Hz
GB       0
PC                  1.40
```

Supplementary Figure 72. ^{1}H NMR spectrum for 3a

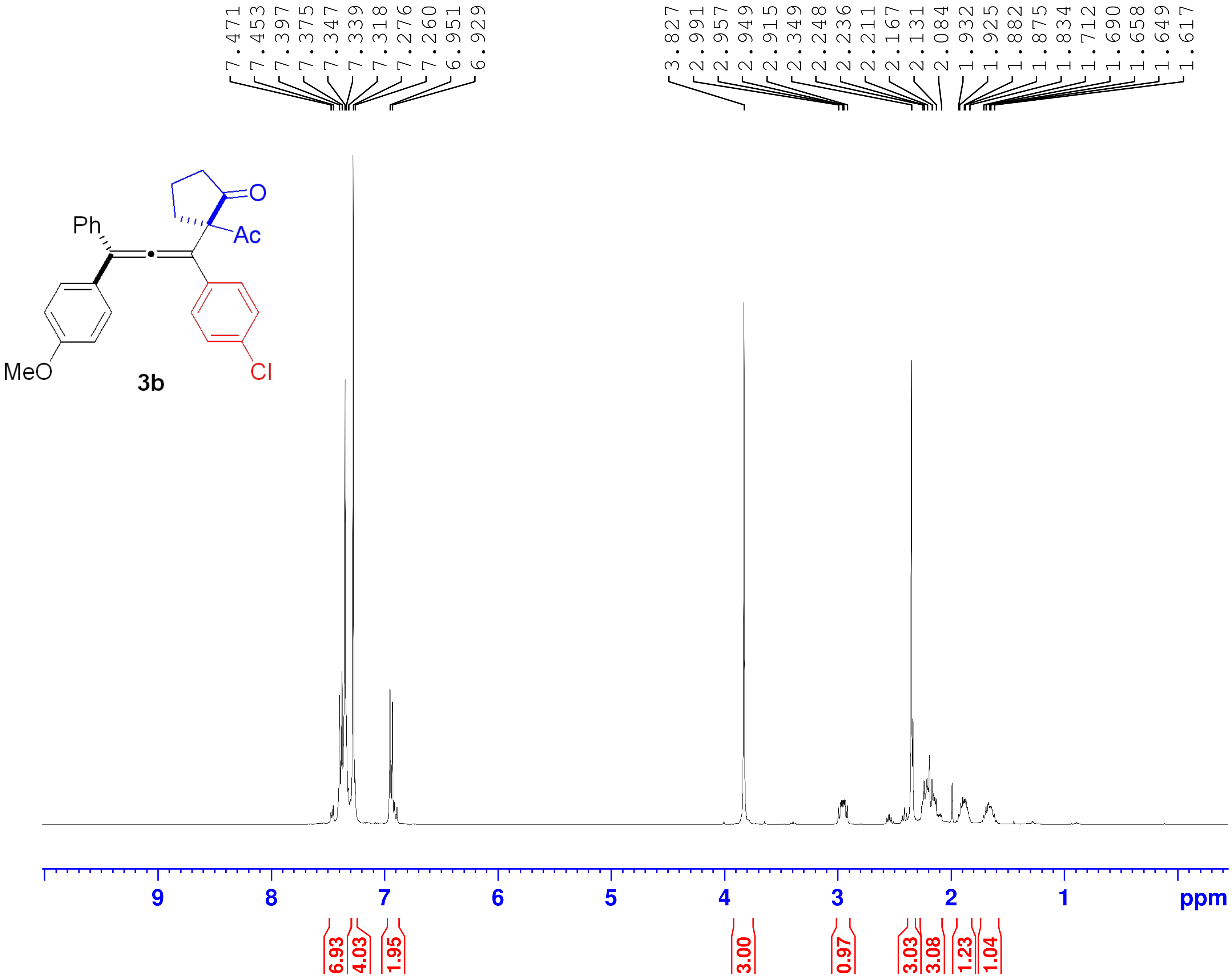


```
Current Data Parameters
NAME        qdy-40018-3 H
EXPNO                   1
PROCNO                  1

F2 - Acquisition Parameters
Date_            20161219
Time                19.28
INSTRUM             spect
PROBHD    5 mm PABBO BB/
PULPROG              zg30
TD                  65536
SOLVENT             CDCl3
NS                      4
DS                      0
SWH             8012.820 Hz
FIDRES          0.122266 Hz
AQ             4.0894465 sec
RG                  34.77
DW                 62.400 usec
DE                   6.50 usec
TE                  295.7 K
D1             1.00000000 sec
TD0                     1

======== CHANNEL f1 ========
SFO1         400.1324710 MHz
NUC1                   1H
P1                  14.50 usec
PLW1          11.99499989 W

F2 - Processing parameters
SI                  65536
SF            400.1300087 MHz
WDW                    EM
SSB      0
LB                   0.30 Hz
GB       0
PC                   1.00
```

Supplementary Figure 73. ^{1}H NMR spectrum for 3b

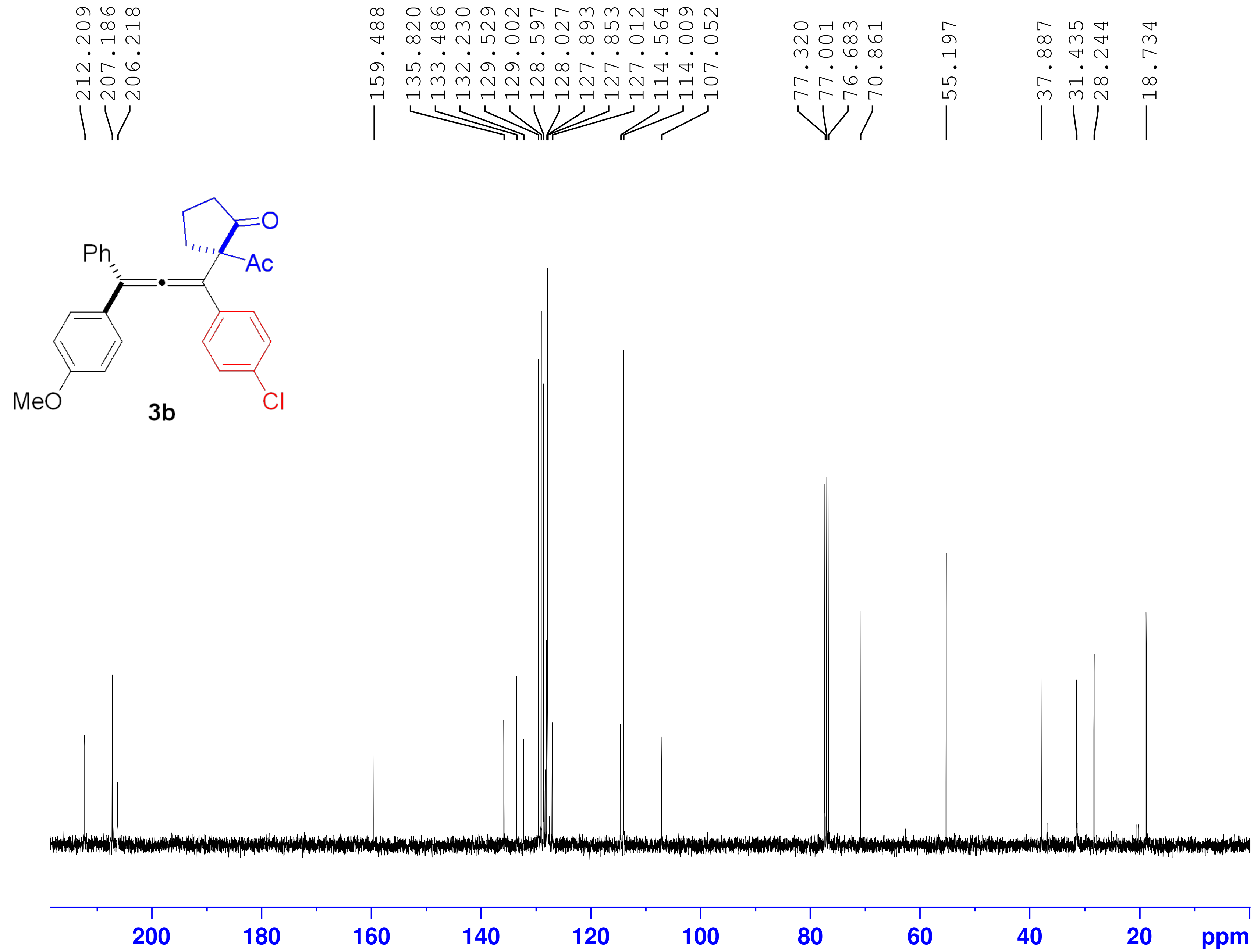

Current Data Parameters
NAME qdy-40018-3 C
EXPNO 1
PROCNO 1

F2 - Acquisition Parameters
Date_ 20161219
Time 19.30
INSTRUM spect
PROBHD 5 mm PABBO BB/
PULPROG zgpg30
TD 65536
SOLVENT CDCl3
NS 67
DS 0
SWH 24038.461 Hz
FIDRES 0.366798 Hz
AQ 1.3631488 sec
RG 196.92
DW 20.800 usec
DE 6.50 usec
TE 296.4 K
D1 2.00000000 sec
D11 0.03000000 sec
TD0 1

======== CHANNEL f1 ========
SFO1 100.6228298 MHz
NUC1 13C
P1 9.70 usec
PLW1 46.98899841 W

======== CHANNEL f2 ========
SFO2 400.1316005 MHz
NUC2 1H
CPDPRG[2 waltz16
PCPD2 90.00 usec
PLW2 11.99499989 W
PLW12 0.34213999 W
PLW13 0.27713001 W

F2 - Processing parameters
SI 32768
SF 100.6127824 MHz
WDW EM
SSB 0
LB 1.00 Hz
GB 0
PC 1.40

Supplementary Figure 74. ^{13}C NMR spectrum for 3b

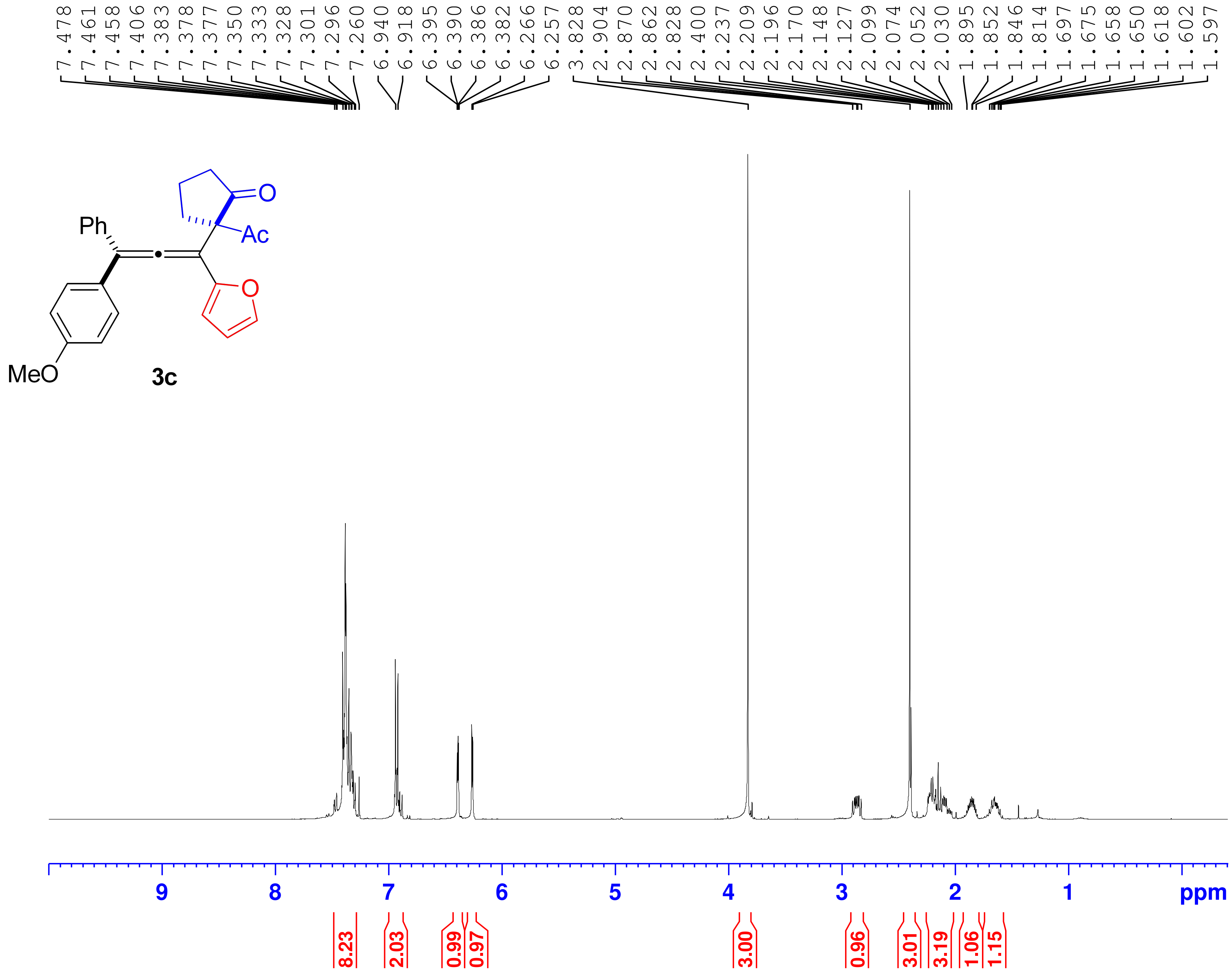

Current Data Parameters
NAME qdy-40025 H
EXPNO 1
PROCNO 1

F2 - Acquisition Parameters
Date_ 20161229
Time 15.05
INSTRUM spect
PROBHD 5 mm DUL 13C-1
PULPROG zg30
TD 65536
SOLVENT CDCl3
NS 4
DS 0
SWH 8012.820 Hz
FIDRES 0.122266 Hz
AQ 4.0894465 sec
RG 49.32
DW 62.400 usec
DE 6.50 usec
TE 296.3 K
D1 1.00000000 sec
TD0 1

======== CHANNEL f1 ========
SFO1 400.1324710 MHz
NUC1 1H
P1 14.30 usec
PLW1 9.10000038 W

F2 - Processing parameters
SI 65536
SF 400.1300090 MHz
WDW EM
SSB 0
LB 0.30 Hz
GB 0
PC 1.00

Supplementary Figure 75. ^{1}H NMR spectrum for 3c

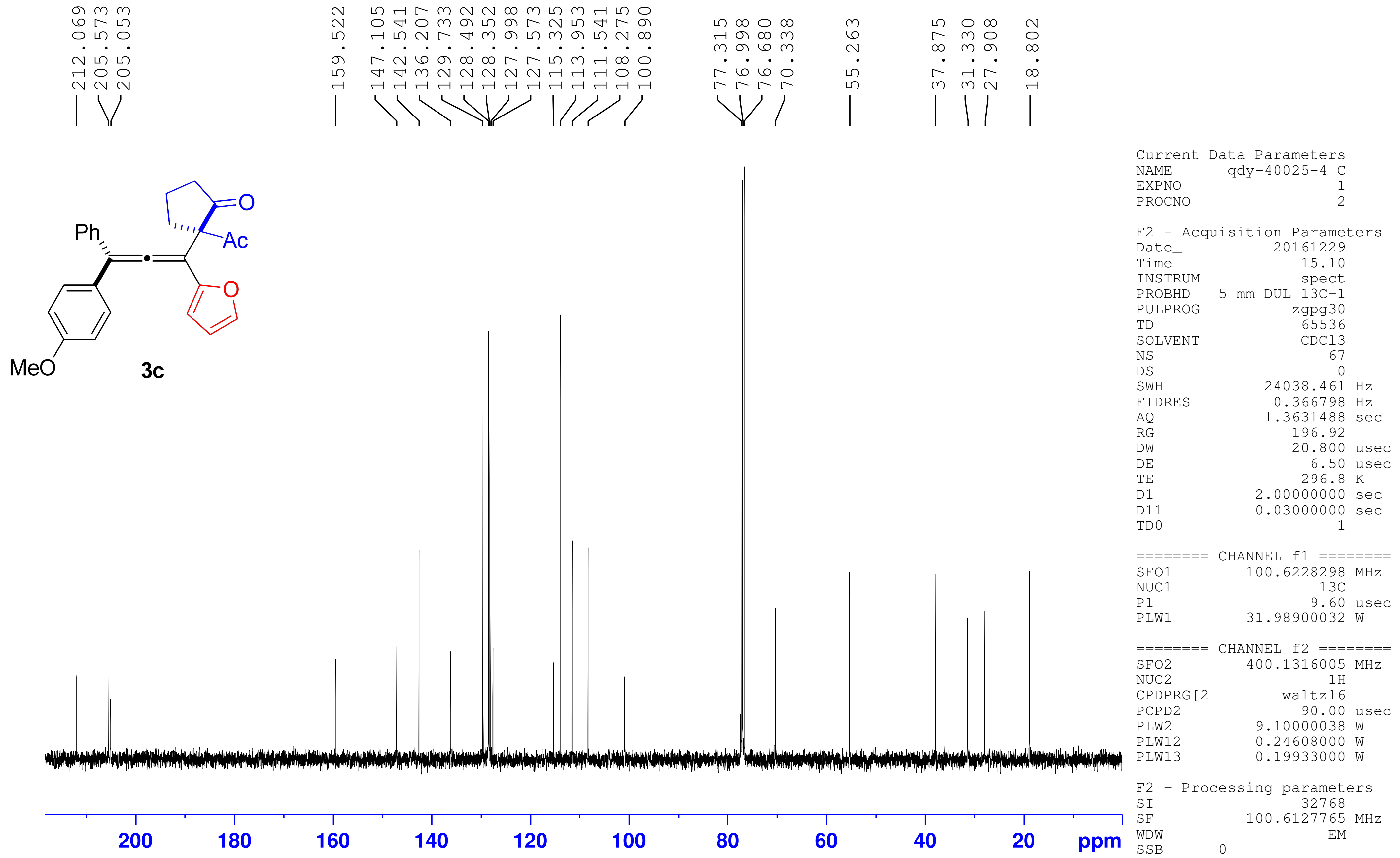

Supplementary Figure 76. ^{13}C NMR spectrum for 3c

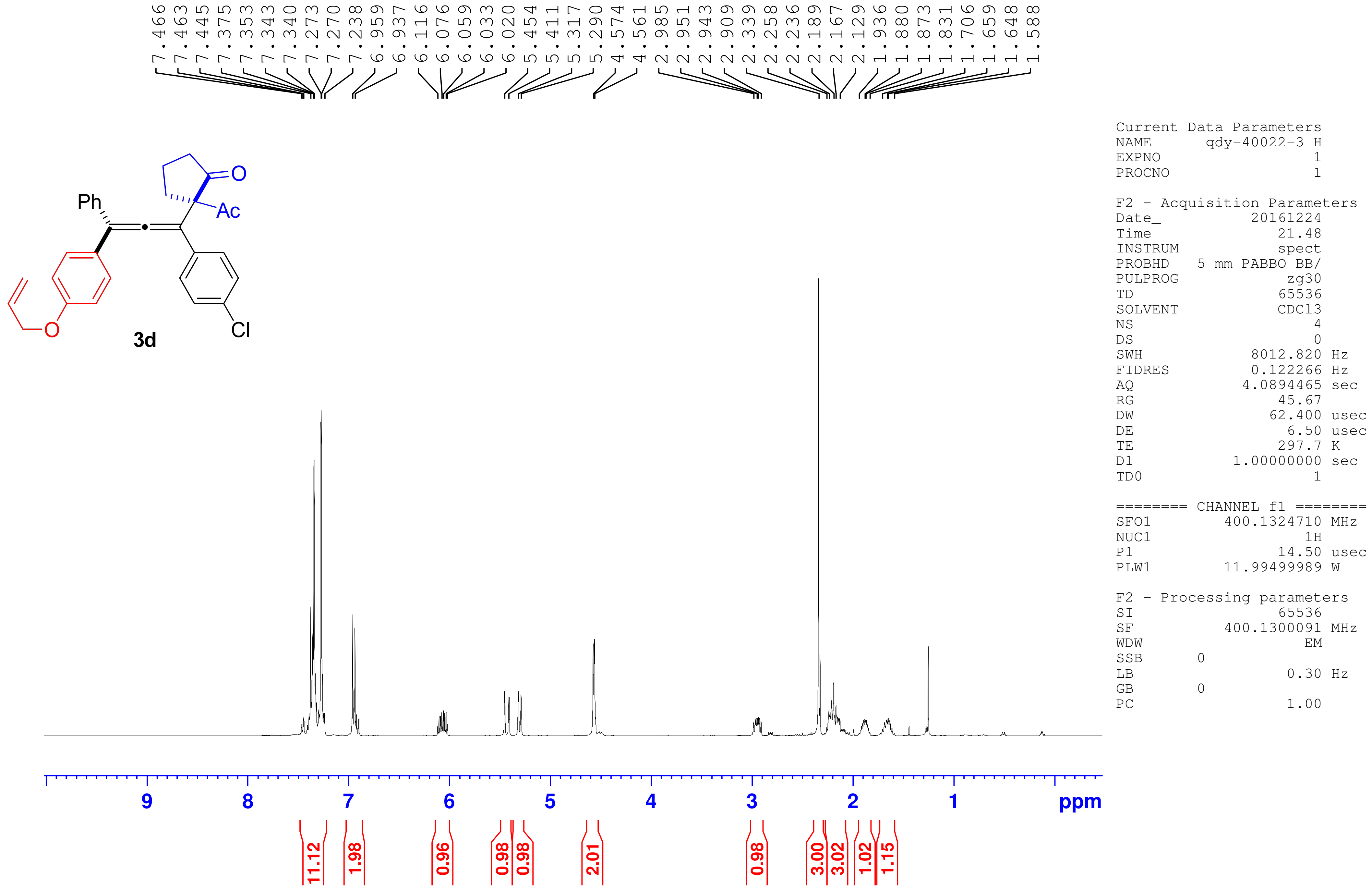

Supplementary Figure 77. ^{1}H NMR spectrum for 3d

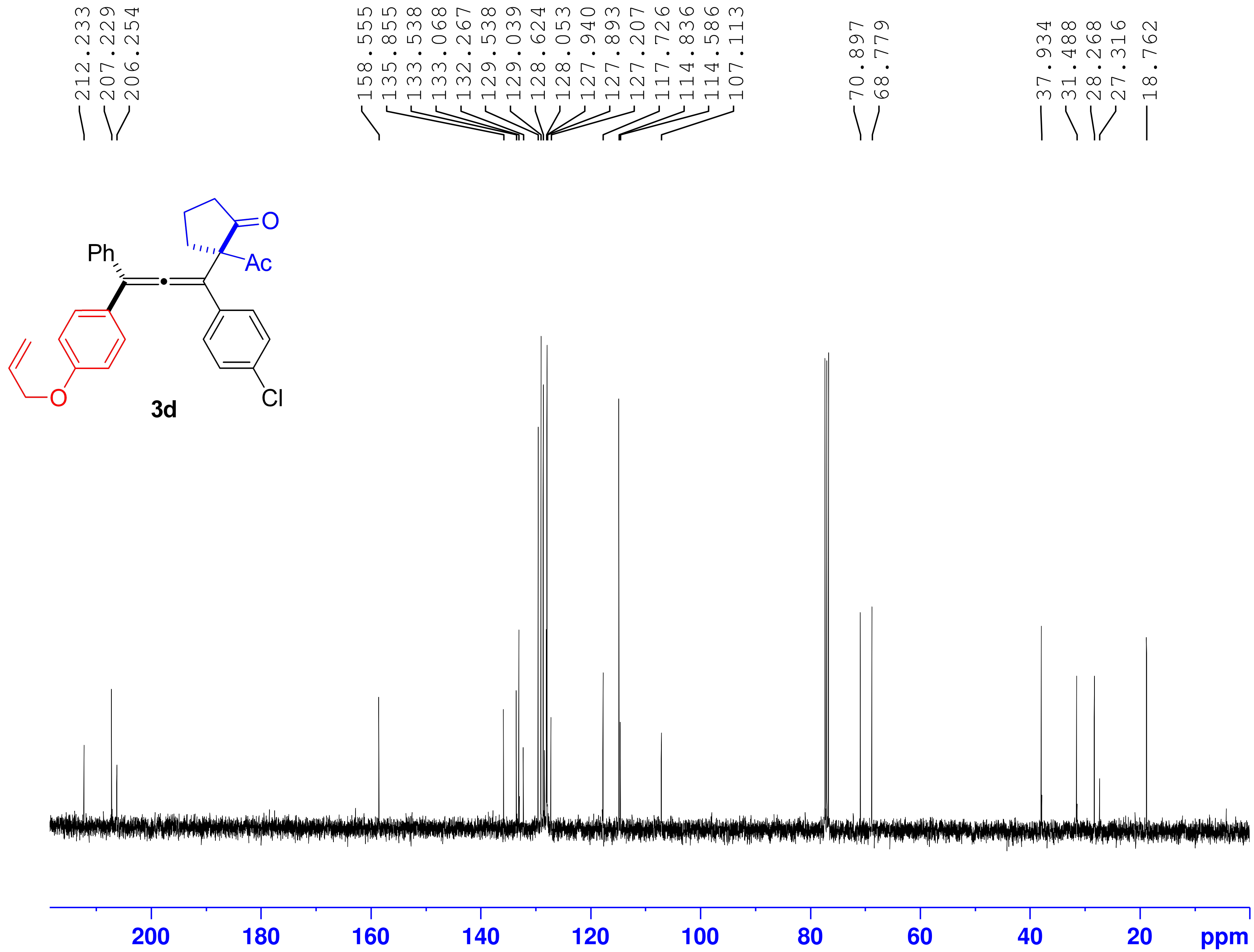

Current Data Parameters
NAME qdy-40022-3 C
EXPNO 1
PROCNO 1

F2 - Acquisition Parameters
Date_ 20161224
Time 21.51
INSTRUM spect
PROBHD 5 mm PABBO BB/
PULPROG zgpg30
TD 65536
SOLVENT CDCl3
NS 51
DS 0
SWH 24038.461 Hz
FIDRES 0.366798 Hz
AQ 1.3631488 sec
RG 196.92
DW 20.800 usec
DE 6.50 usec
TE 298.4 K
D1 2.00000000 sec
D11 0.03000000 sec
TD0 1

======== CHANNEL f1 ========
SFO1 100.6228298 MHz
NUC1 13C
P1 9.70 usec
PLW1 46.98899841 W

======== CHANNEL f2 ========
SFO2 400.1316005 MHz
NUC2 1H
CPDPRG[2 waltz16
PCPD2 90.00 usec
PLW2 11.99499989 W
PLW12 0.34213999 W
PLW13 0.27713001 W

F2 - Processing parameters
SI 32768
SF 100.6127780 MHz
WDW EM
SSB 0
LB 1.00 Hz
GB 0
PC 1.40

Supplementary Figure 78. ^{13}C NMR spectrum for 3d

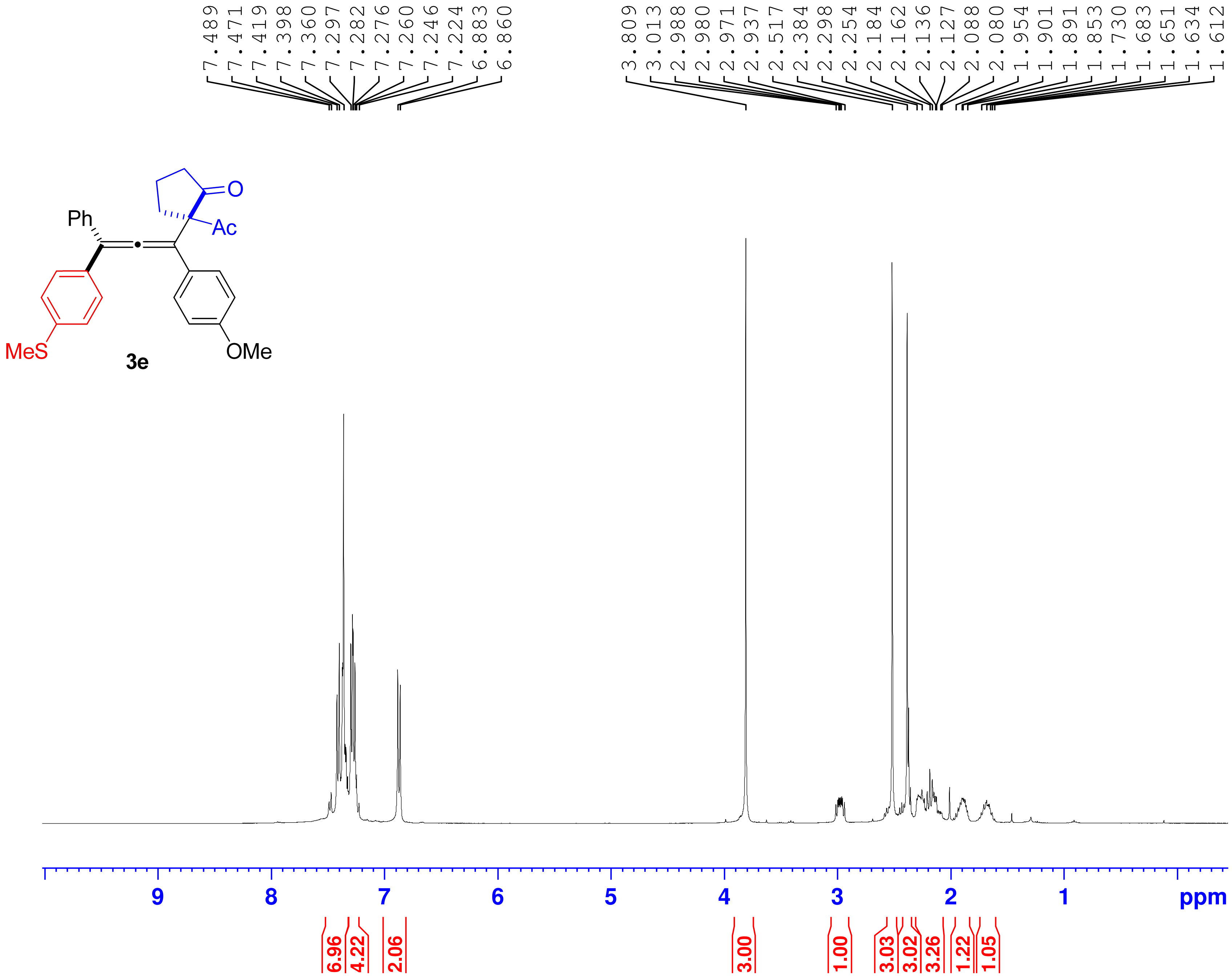

Current Data Parameters
NAME qdy-40018-2 H
EXPNO 1
PROCNO 1

F2 - Acquisition Parameters
Date_ 20161217
Time 19.28
INSTRUM spect
PROBHD 5 mm PABBO BB/
PULPROG zg30
TD 65536
SOLVENT CDCl3
NS 4
DS 0
SWH 8012.820 Hz
FIDRES 0.122266 Hz
AQ 4.0894465 sec
RG 34.77
DW 62.400 usec
DE 6.50 usec
TE 296.8 K
D1 1.00000000 sec
TD0 1

======== CHANNEL f1 ========
SFO1 400.1324710 MHz
NUC1 1H
P1 14.50 usec
PLW1 11.99499989 W

F2 - Processing parameters
SI 65536
SF 400.1300012 MHz
WDW EM
SSB 0
LB 0.30 Hz
GB 0
PC 1.00

Supplementary Figure 79. ^{1}H NMR spectrum for 3e

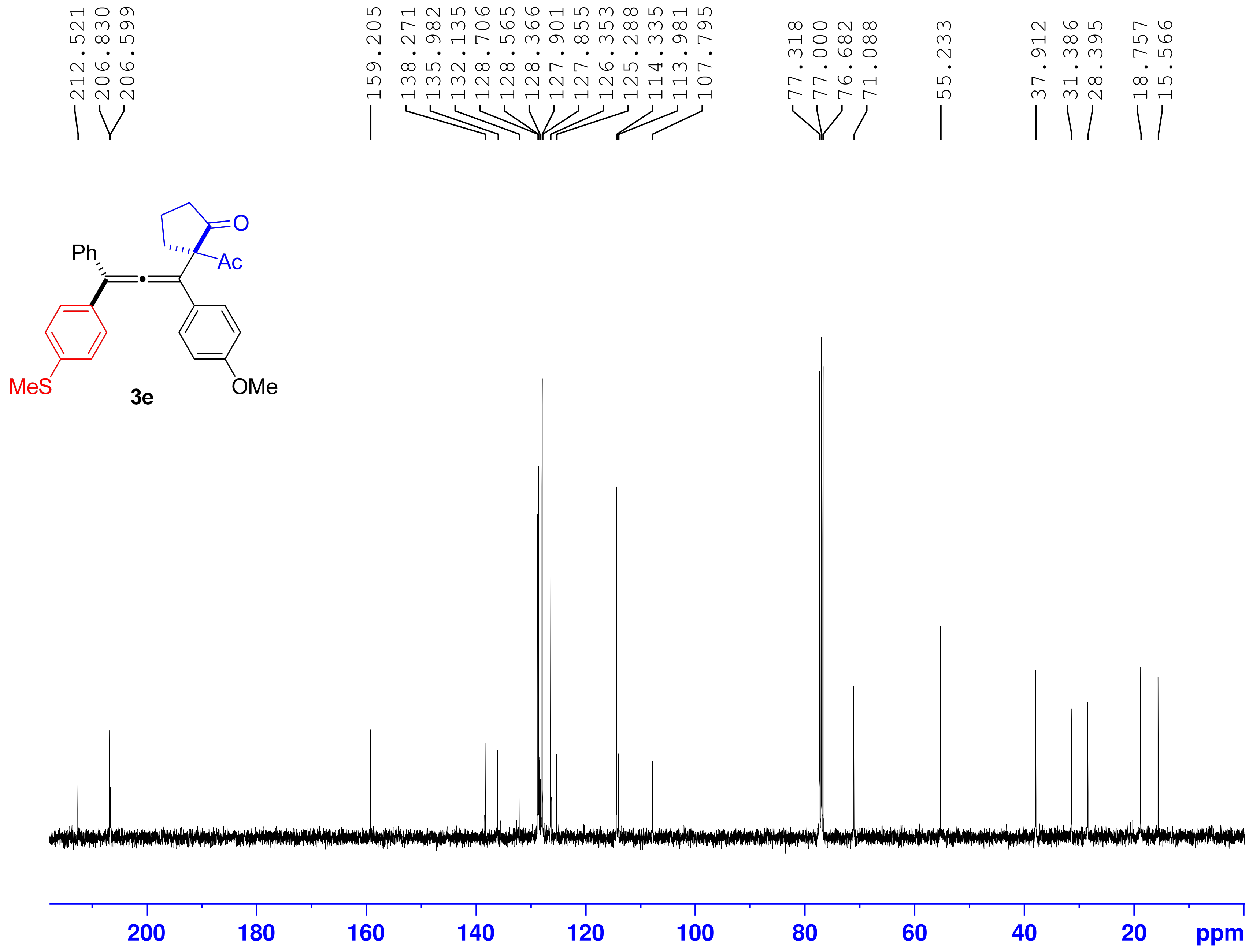


```
Current Data Parameters
NAME       qdy-40018-2 C
EXPNO                  1
PROCNO                 1

F2 - Acquisition Parameters
Date_           20161217
Time               19.31
INSTRUM            spect
PROBHD    5 mm PABBO BB/
PULPROG          zgpg30
TD                 65536
SOLVENT            CDCl3
NS                    76
DS                     0
SWH            24038.461 Hz
FIDRES          0.366798 Hz
AQ             1.3631488 sec
RG                196.92
DW                20.800 usec
DE                  6.50 usec
TE                 297.7 K
D1            2.00000000 sec
D11           0.03000000 sec
TD0                    1

======== CHANNEL f1 ========
SFO1         100.6228298 MHz
NUC1                 13C
P1                  9.70 usec
PLW1         46.98899841 W

======== CHANNEL f2 ========
SFO2         400.1316005 MHz
NUC2                  1H
CPDPRG[2         waltz16
PCPD2              90.00 usec
PLW2         11.99499989 W
PLW12         0.34213999 W
PLW13         0.27713001 W

F2 - Processing parameters
SI                 32768
SF           100.6127795 MHz
WDW                   EM
SSB        0
LB                  1.00 Hz
GB         0
PC                  1.40
```

Supplementary Figure 80. ^{13}C NMR spectrum for 3e

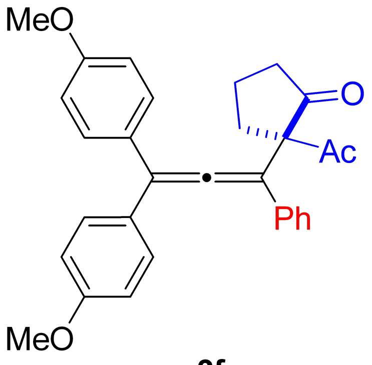

3f

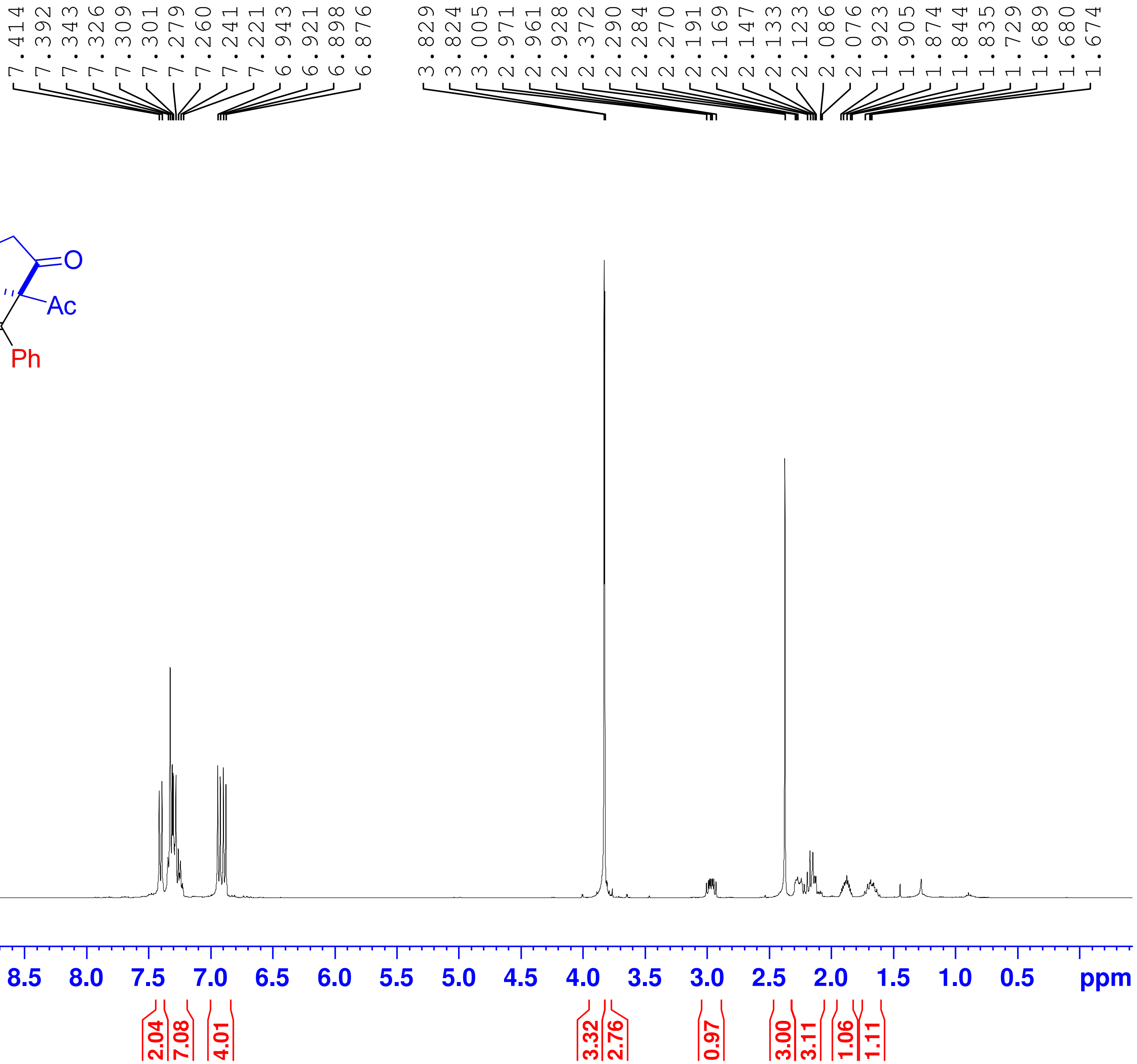

Current Data Parameters
NAME qdy-30187 H
EXPNO 2
PROCNO 1

F2 - Acquisition Parameters
Date_ 20161219
Time 19.46
INSTRUM spect
PROBHD 5 mm PABBO BB/
PULPROG zg30
TD 65536
SOLVENT CDCl3
NS 4
DS 0
SWH 8012.820 Hz
FIDRES 0.122266 Hz
AQ 4.0894465 sec
RG 49.32
DW 62.400 usec
DE 6.50 usec
TE 295.9 K
D1 1.00000000 sec
TD0 1

======== CHANNEL f1 ========
SFO1 400.1324710 MHz
NUC1 1H
P1 14.50 usec
PLW1 11.99499989 W

F2 - Processing parameters
SI 65536
SF 400.1300088 MHz
WDW EM
SSB 0
LB 0.30 Hz
GB 0
PC 1.00

Supplementary Figure 81. ^{1}H NMR spectrum for 3f

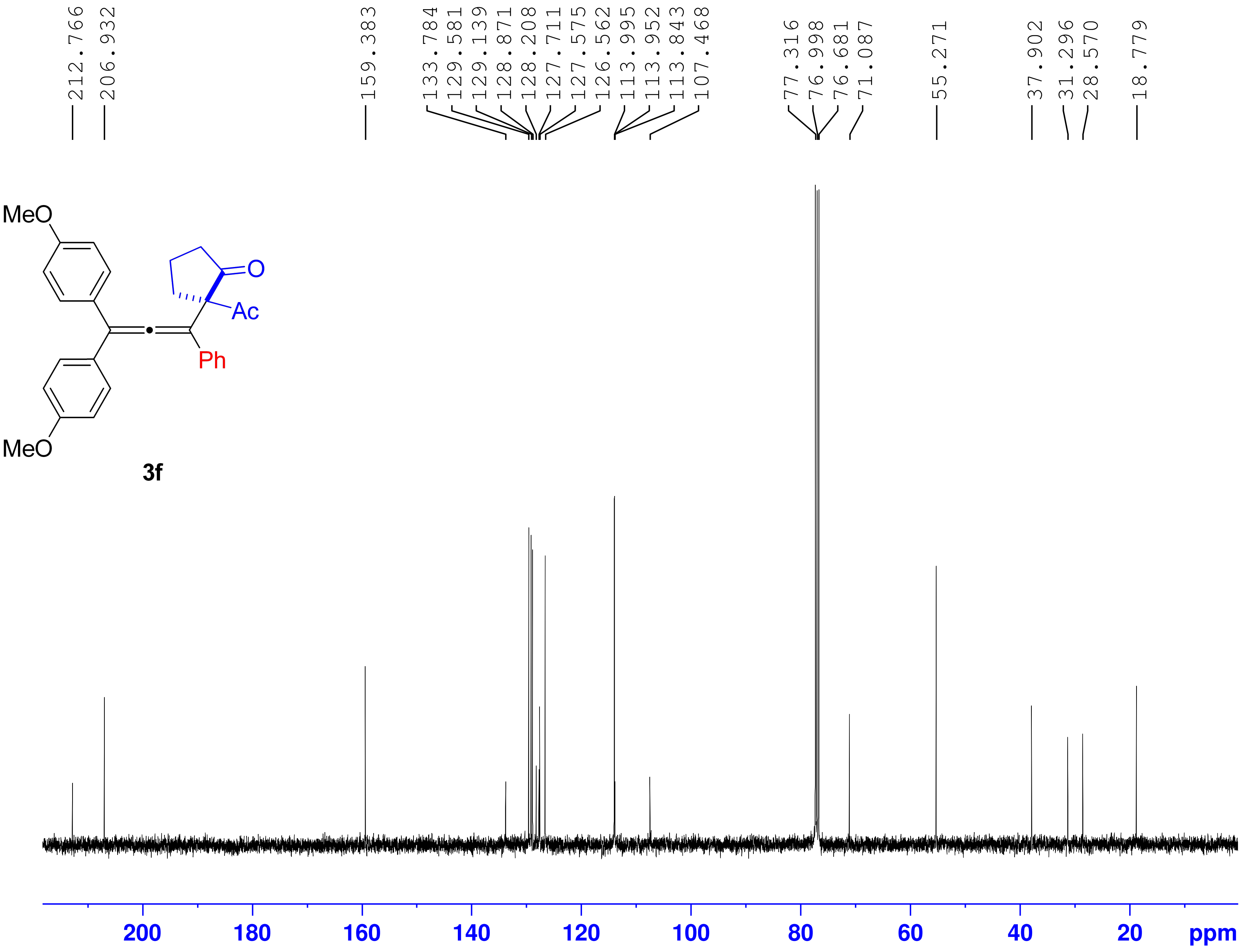


```
Current Data Parameters
NAME          qdy-30187 C
EXPNO                   1
PROCNO                  1

F2 - Acquisition Parameters
Date_            20161207
Time                19.32
INSTRUM             spect
PROBHD    5 mm PABBO BB/
PULPROG           zgpg30
TD                  65536
SOLVENT             CDCl3
NS                     99
DS                      0
SWH             24038.461 Hz
FIDRES           0.366798 Hz
AQ              1.3631488 sec
RG                 196.92
DW                 20.800 usec
DE                   6.50 usec
TE                  296.4 K
D1             2.00000000 sec
D11            0.03000000 sec
TD0                     1

======== CHANNEL f1 ========
SFO1          100.6228298 MHz
NUC1                  13C
P1                   9.70 usec
PLW1          46.98899841 W

======== CHANNEL f2 ========
SFO2          400.1316005 MHz
NUC2                   1H
CPDPRG[2          waltz16
PCPD2               90.00 usec
PLW2          11.99499989 W
PLW12          0.34213999 W
PLW13          0.27713001 W

F2 - Processing parameters
SI                  32768
SF            100.6127744 MHz
WDW                    EM
SSB      0
LB                   1.00 Hz
GB       0
PC                   1.40
```

Supplementary Figure 82. ^{13}C NMR spectrum for 3f

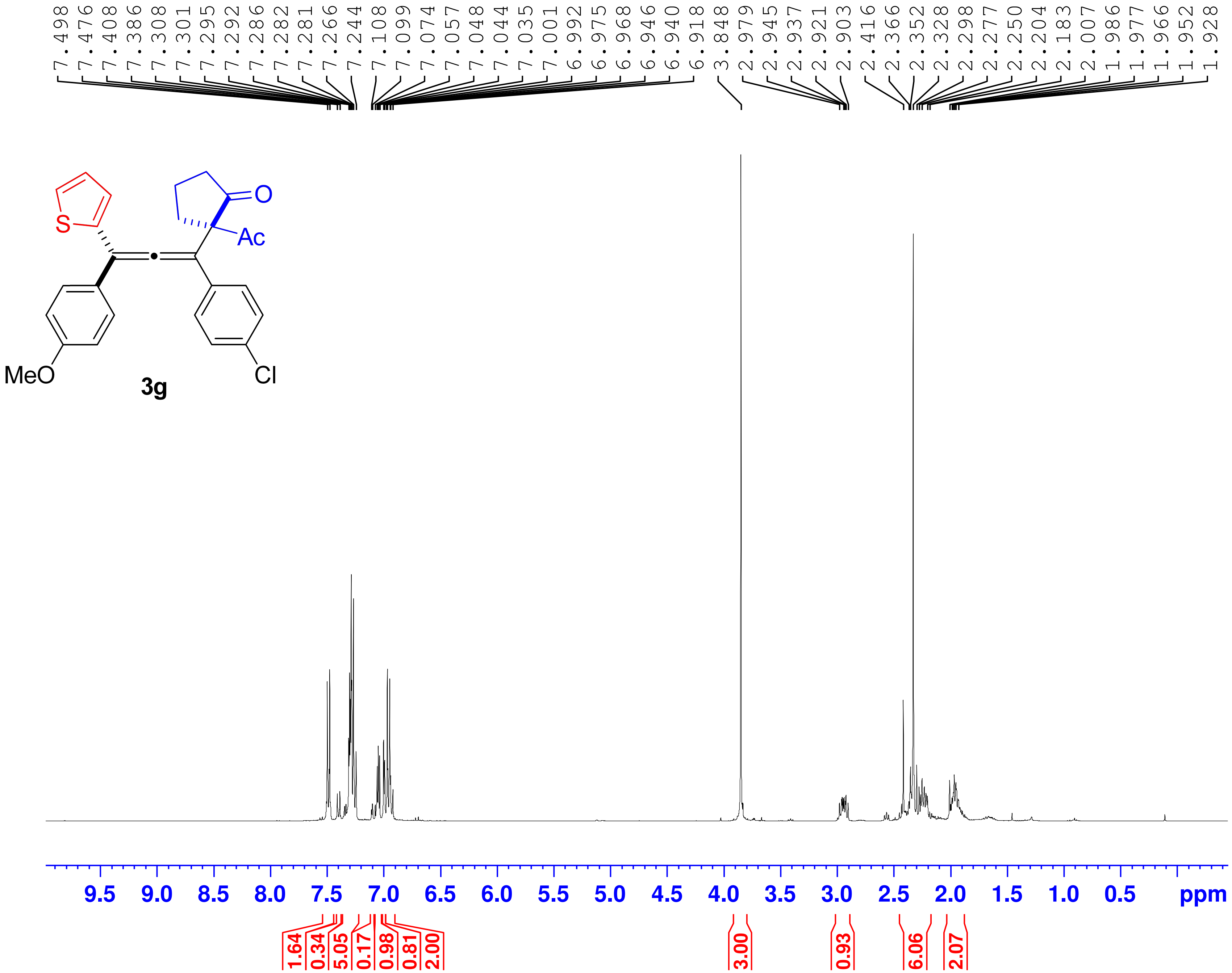


```
Current Data Parameters
NAME        qdy-40018-4 H
EXPNO                   1
PROCNO                  1

F2 - Acquisition Parameters
Date_            20161219
Time                19.21
INSTRUM             spect
PROBHD   5 mm PABBO BB/
PULPROG              zg30
TD                  65536
SOLVENT             CDCl3
NS                      4
DS                      0
SWH             8012.820 Hz
FIDRES          0.122266 Hz
AQ             4.0894465 sec
RG                  45.67
DW                62.400 usec
DE                  6.50 usec
TE                 295.6 K
D1            1.00000000 sec
TD0                     1

======== CHANNEL f1 ========
SFO1         400.1324710 MHz
NUC1                  1H
P1                 14.50 usec
PLW1         11.99499989 W

F2 - Processing parameters
SI                  65536
SF           400.1300009 MHz
WDW                    EM
SSB      0
LB                   0.30 Hz
GB       0
PC                   1.00
```

Supplementary Figure 83. ^{1}H NMR spectrum for 3g

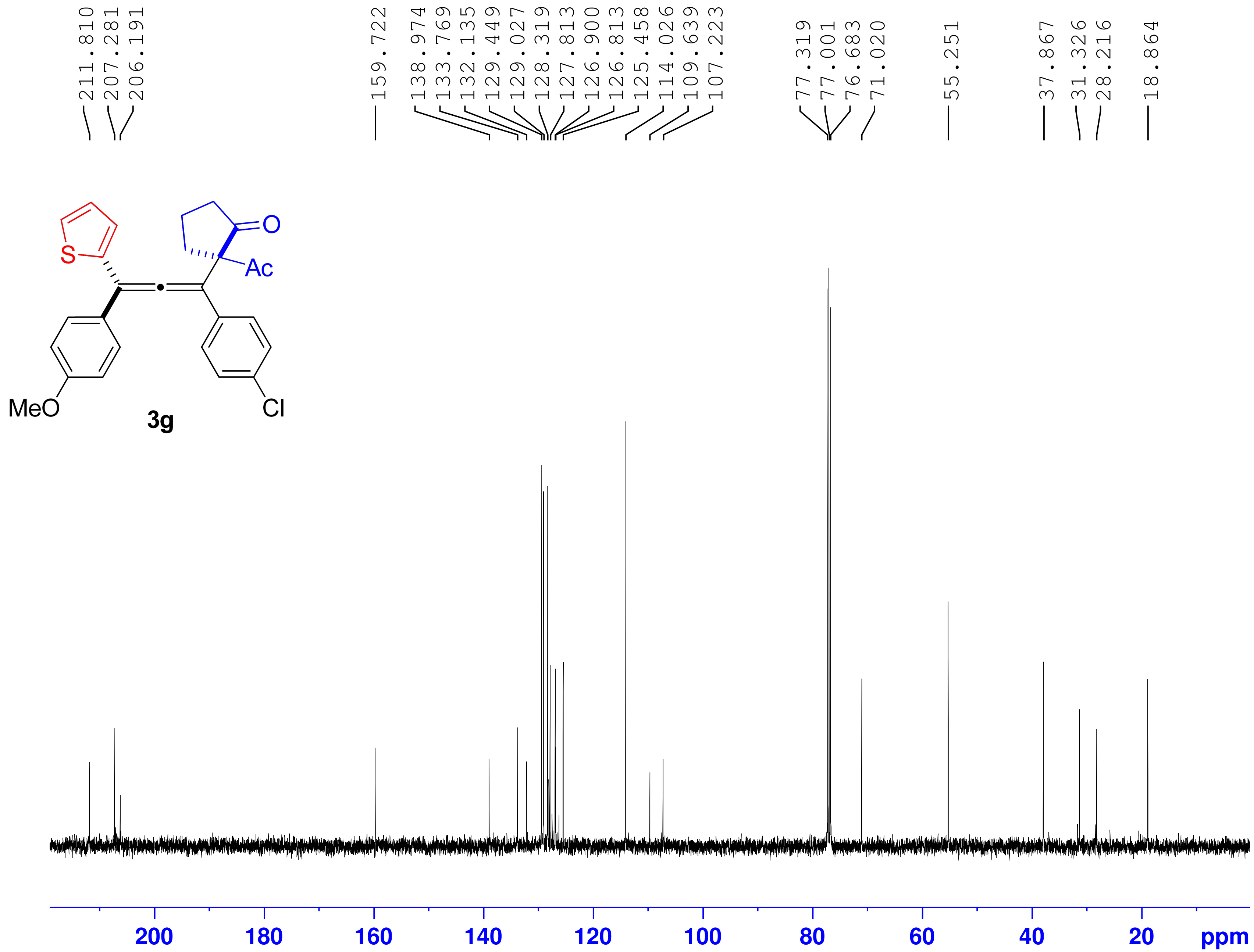

Current Data Parameters
NAME qdy-40018-4 C
EXPNO 1
PROCNO 1

F2 - Acquisition Parameters
Date_ 20161219
Time 19.24
INSTRUM spect
PROBHD 5 mm PABBO BB/
PULPROG zgpg30
TD 65536
SOLVENT CDCl3
NS 60
DS 0
SWH 24038.461 Hz
FIDRES 0.366798 Hz
AQ 1.3631488 sec
RG 196.92
DW 20.800 usec
DE 6.50 usec
TE 296.5 K
D1 2.00000000 sec
D11 0.03000000 sec
TD0 1

======== CHANNEL f1 ========
SFO1 100.6228298 MHz
NUC1 13C
P1 9.70 usec
PLW1 46.98899841 W

======== CHANNEL f2 ========
SFO2 400.1316005 MHz
NUC2 1H
CPDPRG[2 waltz16
PCPD2 90.00 usec
PLW2 11.99499989 W
PLW12 0.34213999 W
PLW13 0.27713001 W

F2 - Processing parameters
SI 32768
SF 100.6127773 MHz
WDW EM
SSB 0
LB 1.00 Hz
GB 0
PC 1.40

Supplementary Figure 84. ^{13}C NMR spectrum for 3g

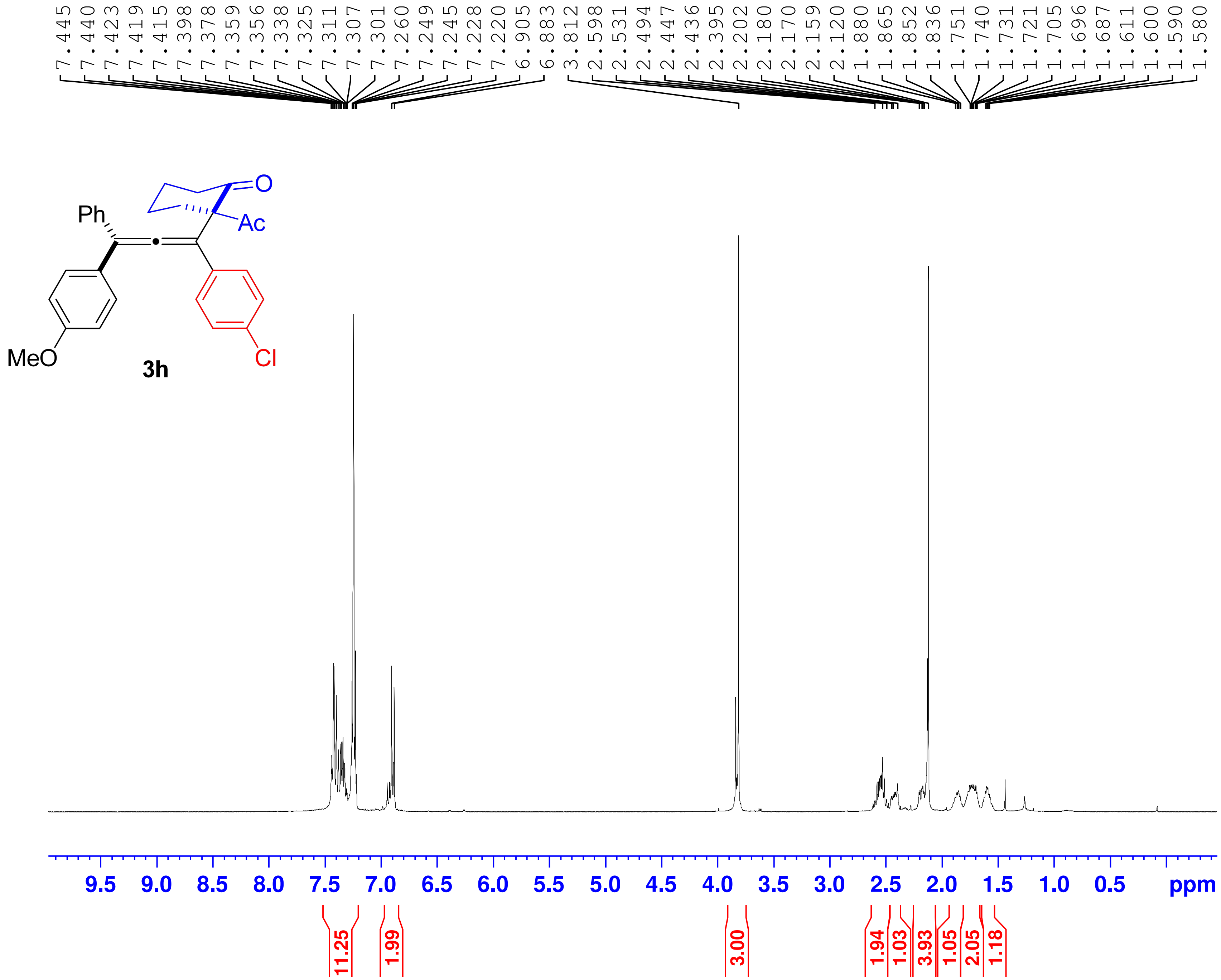


```
Current Data Parameters
NAME       qdy-40023-4 H
EXPNO                  2
PROCNO                 1

F2 - Acquisition Parameters
Date_           20161228
Time               20.29
INSTRUM            spect
PROBHD   5 mm DUL 13C-1
PULPROG             zg30
TD                 65536
SOLVENT            CDCl3
NS                     4
DS                     0
SWH            8012.820 Hz
FIDRES         0.122266 Hz
AQ            4.0894465 sec
RG                126.97
DW                62.400 usec
DE                  6.50 usec
TE                 295.1 K
D1            1.00000000 sec
TD0                    1

======== CHANNEL f1 ========
SFO1        400.1324710 MHz
NUC1                  1H
P1                 14.30 usec
PLW1          9.10000038 W

F2 - Processing parameters
SI                 65536
SF          400.1300091 MHz
WDW                   EM
SSB      0
LB                  0.30 Hz
GB       0
PC                  1.00
```

Supplementary Figure 85. ^{1}H NMR spectrum for 3h

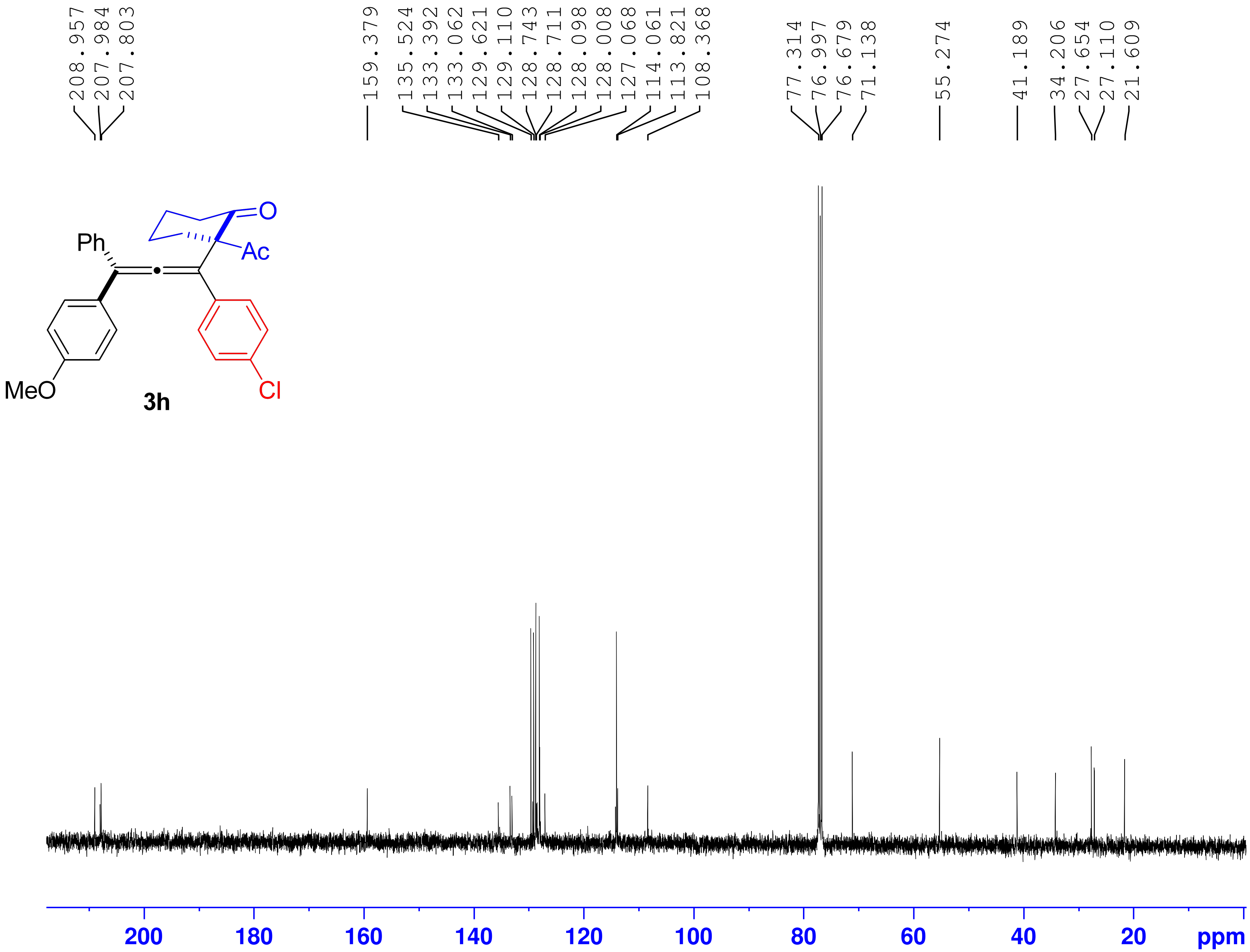


```
Current Data Parameters
NAME          qdy-40023-4 C
EXPNO                     1
PROCNO                    1

F2 - Acquisition Parameters
Date_              20161228
Time                  20.20
INSTRUM               spect
PROBHD     5 mm DUL 13C-1
PULPROG             zgpg30
TD                   65536
SOLVENT              CDCl3
NS                     161
DS                       0
SWH              24038.461 Hz
FIDRES            0.366798 Hz
AQ               1.3631488 sec
RG                  196.92
DW                  20.800 usec
DE                    6.50 usec
TE                   295.1 K
D1              2.00000000 sec
D11             0.03000000 sec
TD0                      1

======== CHANNEL f1 ========
SFO1          100.6228298 MHz
NUC1                   13C
P1                    9.60 usec
PLW1           31.98900032 W

======== CHANNEL f2 ========
SFO2          400.1316005 MHz
NUC2                    1H
CPDPRG[2           waltz16
PCPD2                90.00 usec
PLW2            9.10000038 W
PLW12           0.24608000 W
PLW13           0.19933000 W

F2 - Processing parameters
SI                   32768
SF            100.6127743 MHz
WDW                     EM
SSB      0
LB                    1.00 Hz
GB       0
PC                    1.40
```

Supplementary Figure 86. ^{13}C NMR spectrum for 3h

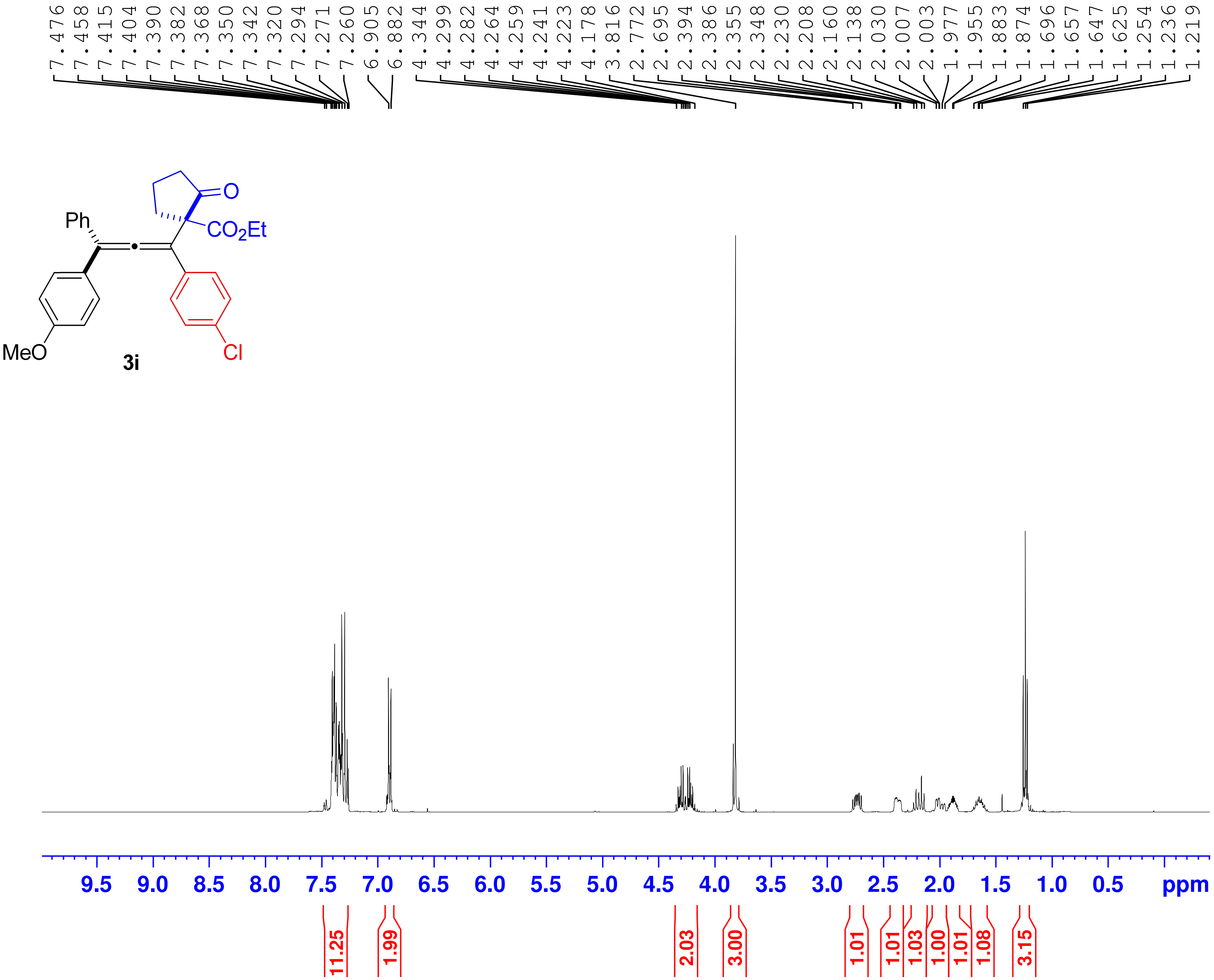


```
Current Data Parameters
NAME        qdy-40023-2 H
EXPNO                   2
PROCNO                  1

F2 - Acquisition Parameters
Date_            20161228
Time                20.11
INSTRUM             spect
PROBHD   5 mm DUL 13C-1
PULPROG              zg30
TD                  65536
SOLVENT             CDCl3
NS                      4
DS                      0
SWH              8012.820 Hz
FIDRES           0.122266 Hz
AQ              4.0894465 sec
RG                  49.32
DW                 62.400 usec
DE                   6.50 usec
TE                  294.5 K
D1             1.00000000 sec
TD0                     1

======== CHANNEL f1 ========
SFO1          400.1324710 MHz
NUC1                   1H
P1                  14.30 usec
PLW1           9.10000038 W

F2 - Processing parameters
SI                  65536
SF            400.1300089 MHz
WDW                    EM
SSB      0
LB                   0.30 Hz
GB       0
PC                   1.00
```

Supplementary Figure 87. ^{1}H NMR spectrum for 3i

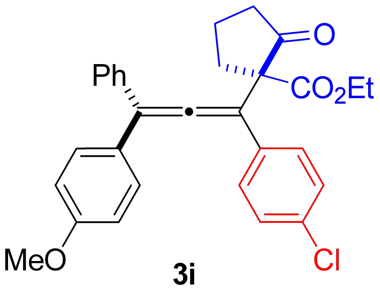

3i

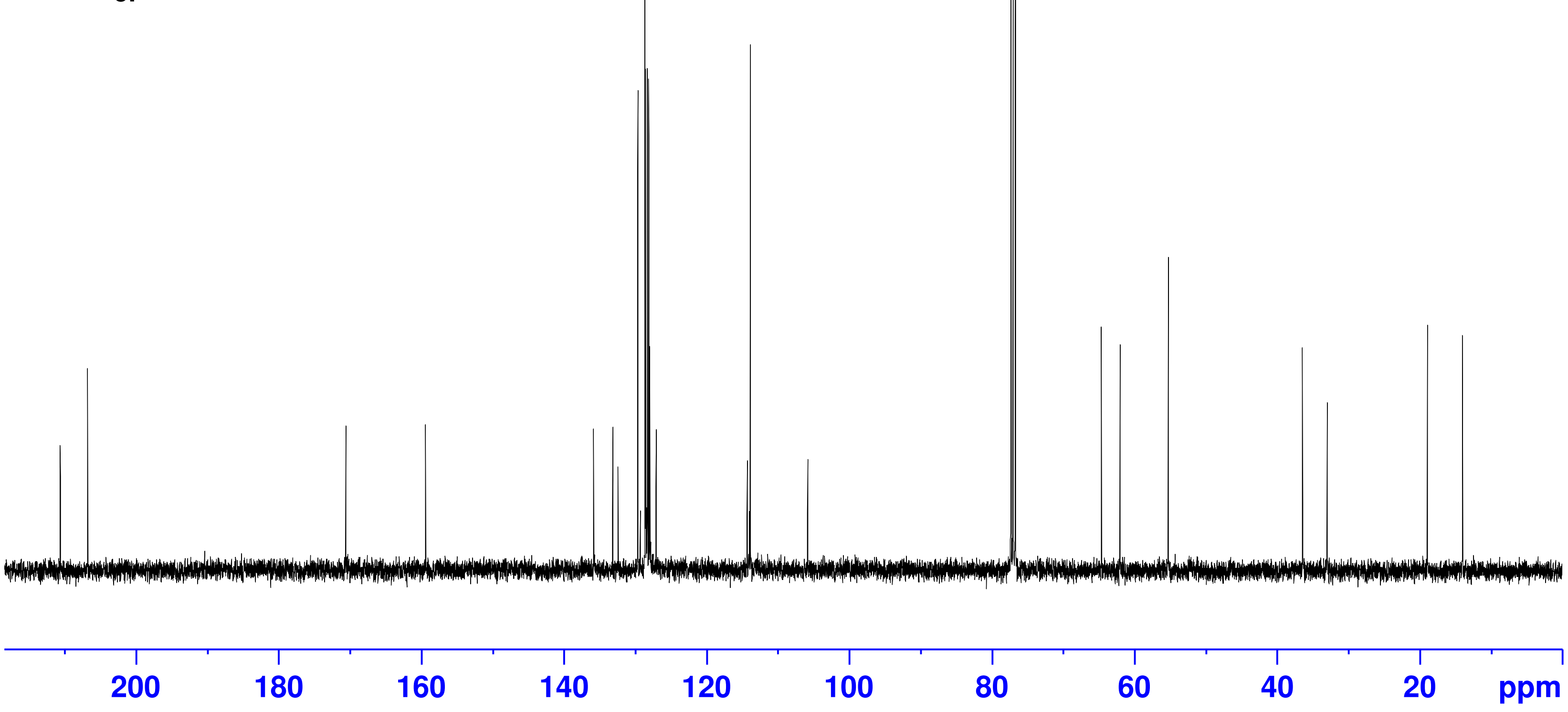


```
Current Data Parameters
NAME       qdy-40023-2 C
EXPNO                  1
PROCNO                 1

F2 - Acquisition Parameters
Date_           20161228
Time               16.52
INSTRUM            spect
PROBHD   5 mm DUL 13C-1
PULPROG          zgpg30
TD                 65536
SOLVENT            CDCl3
NS                    57
DS                     0
SWH            24038.461 Hz
FIDRES          0.366798 Hz
AQ             1.3631488 sec
RG                196.92
DW                20.800 usec
DE                  6.50 usec
TE                 295.8 K
D1            2.00000000 sec
D11           0.03000000 sec
TD0                    1

======== CHANNEL f1 ========
SFO1         100.6228298 MHz
NUC1                 13C
P1                  9.60 usec
PLW1         31.98900032 W

======== CHANNEL f2 ========
SFO2         400.1316005 MHz
NUC2                  1H
CPDPRG[2         waltz16
PCPD2              90.00 usec
PLW2          9.10000038 W
PLW12         0.24608000 W
PLW13         0.19933000 W

F2 - Processing parameters
SI                 32768
SF           100.6127773 MHz
WDW                   EM
SSB       0
LB                  1.00 Hz
GB        0
PC                  1.40
```

Supplementary Figure 88. ^{13}C NMR spectrum for 3i

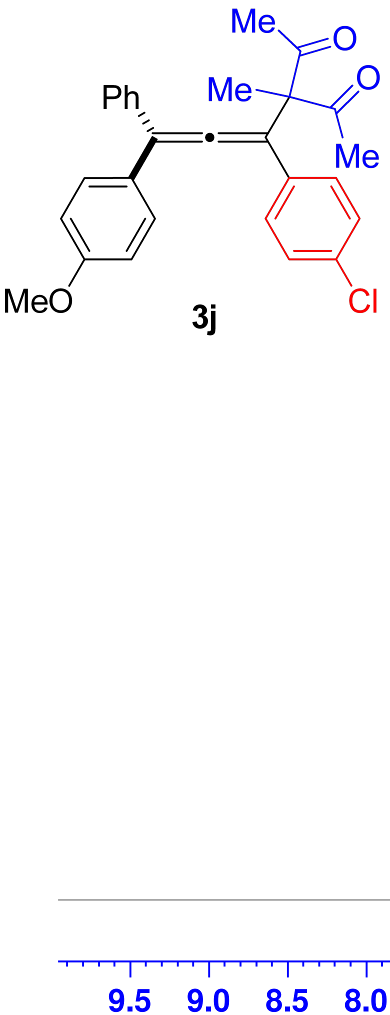

3j

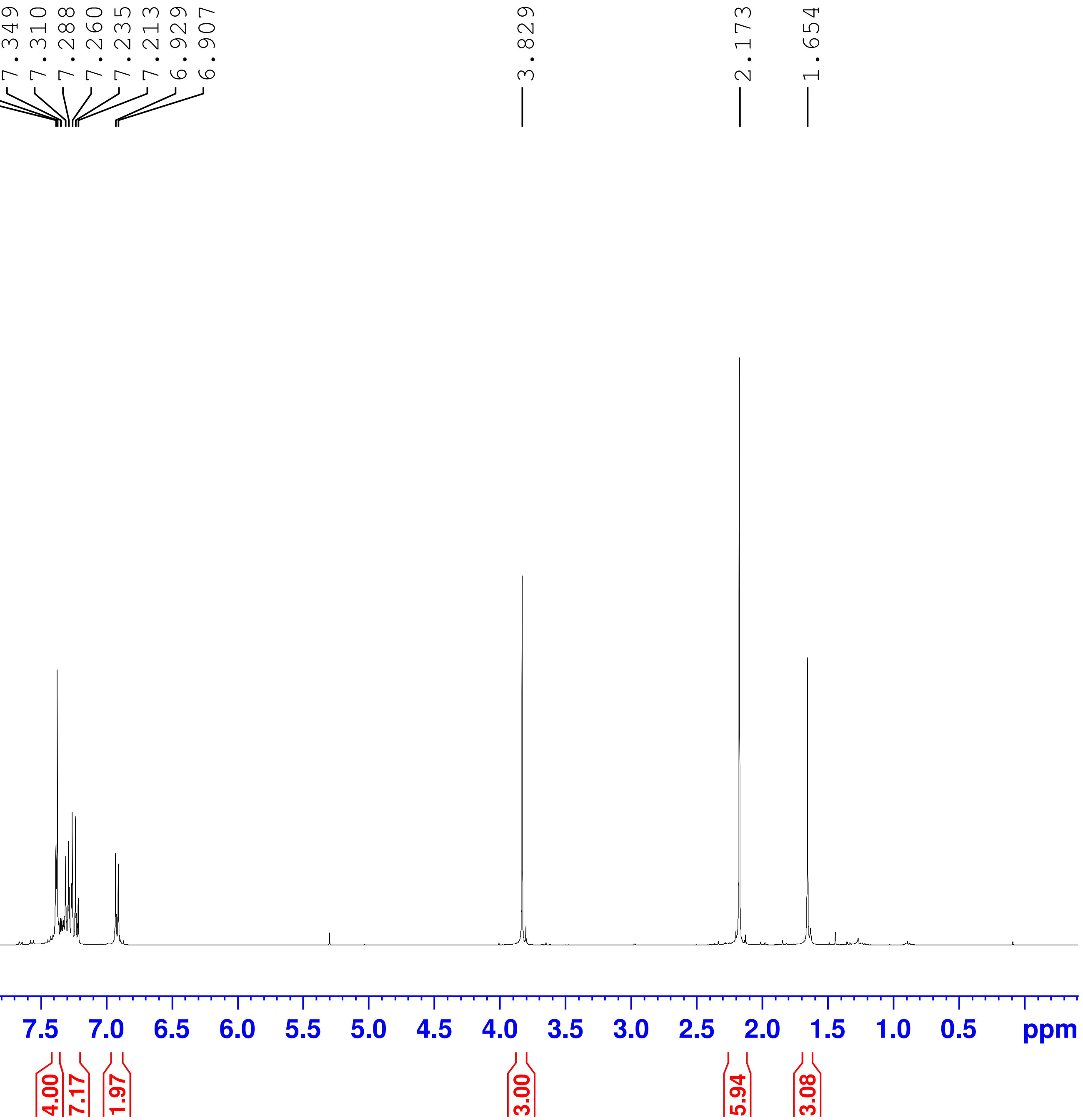

Current Data Parameters
NAME qdy-40023-3 H
EXPNO 1
PROCNO 1

F2 - Acquisition Parameters
Date_ 20161224
Time 16.41
INSTRUM spect
PROBHD 5 mm PABBO BB/
PULPROG zg30
TD 65536
SOLVENT CDCl3
NS 4
DS 0
SWH 8012.820 Hz
FIDRES 0.122266 Hz
AQ 4.0894465 sec
RG 54.81
DW 62.400 usec
DE 6.50 usec
TE 295.2 K
D1 1.00000000 sec
TD0 1

======== CHANNEL f1 ========
SFO1 400.1324710 MHz
NUC1 1H
P1 14.50 usec
PLW1 11.99499989 W

F2 - Processing parameters
SI 65536
SF 400.1300077 MHz
WDW EM
SSB 0
LB 0.30 Hz
GB 0
PC 1.00

Supplementary Figure 89. ¹H NMR spectrum for 3j

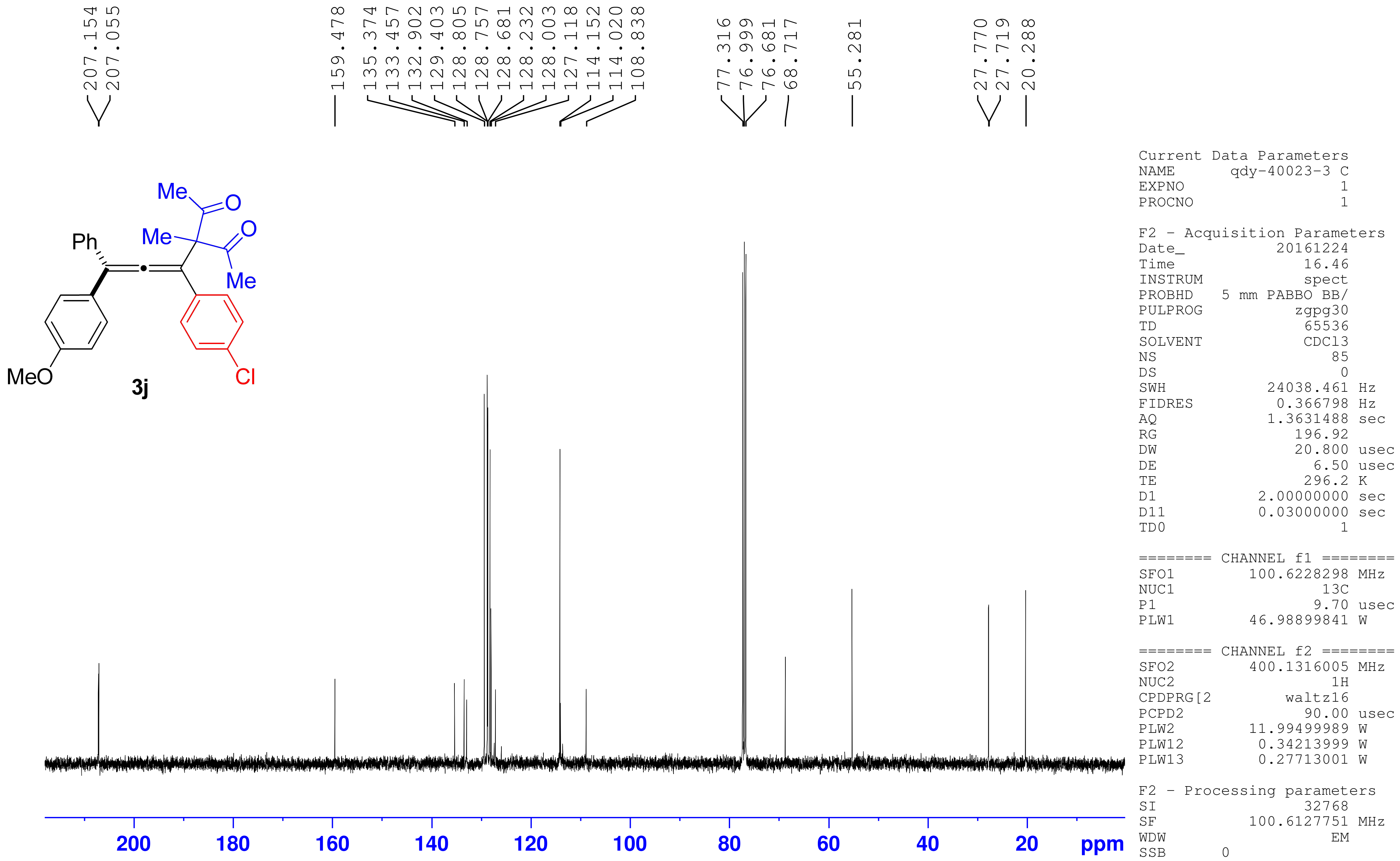

Supplementary Figure 90. ^{13}C **NMR spectrum for 3j**

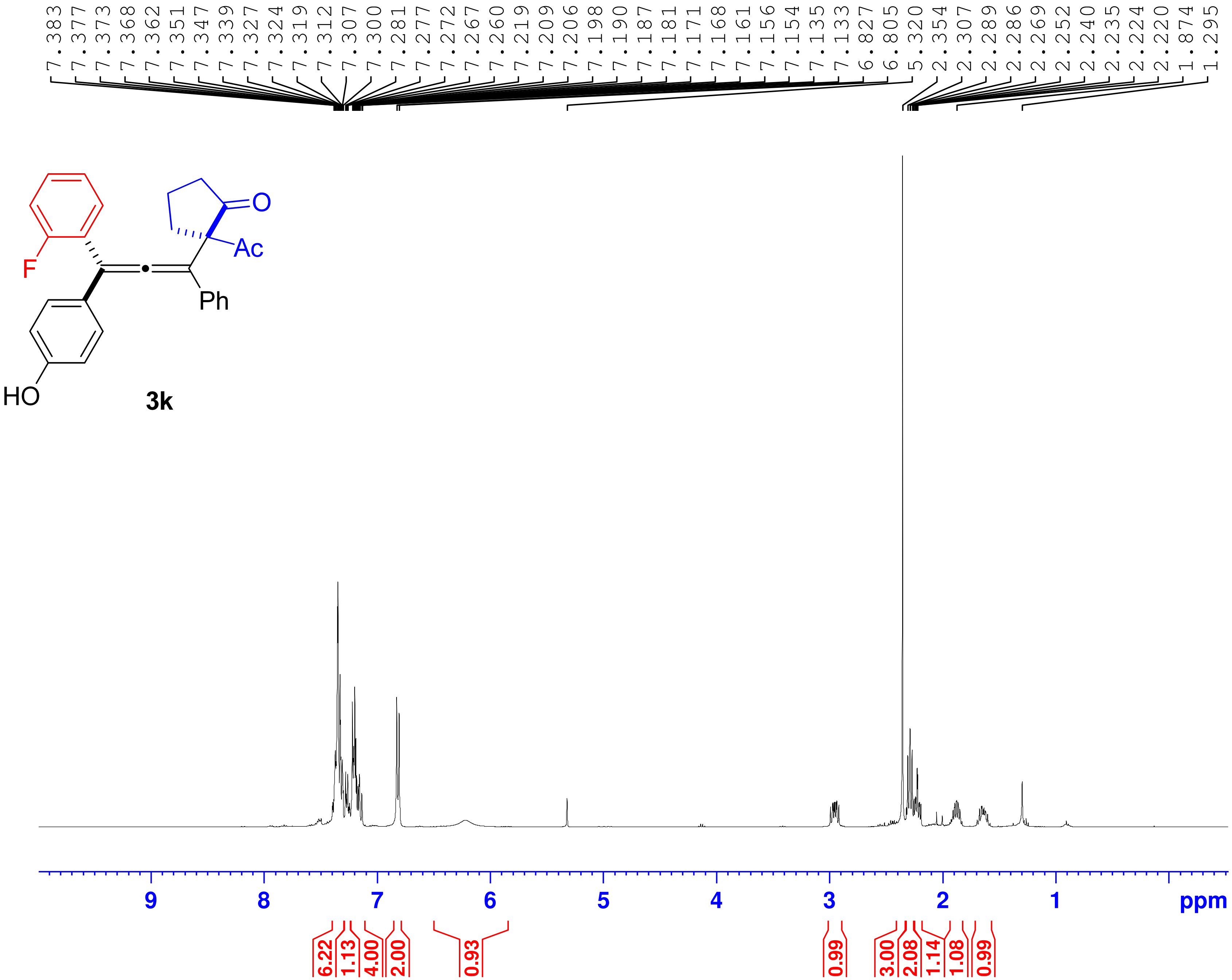

Current Data Parameters
NAME qdy-20093-2 H
EXPNO 1
PROCNO 1

F2 - Acquisition Parameters
Date_ 20160326
Time 13.46
INSTRUM spect
PROBHD 5 mm PABBO BB/
PULPROG zg30
TD 65536
SOLVENT CD2Cl2
NS 5
DS 0
SWH 8012.820 Hz
FIDRES 0.122266 Hz
AQ 4.0894465 sec
RG 34.77
DW 62.400 usec
DE 6.50 usec
TE 296.8 K
D1 1.00000000 sec
TD0 1

======== CHANNEL f1 ========
SFO1 400.1324710 MHz
NUC1 1H
P1 14.50 usec
PLW1 11.99499989 W

F2 - Processing parameters
SI 65536
SF 400.1300152 MHz
WDW EM
SSB 0
LB 0.30 Hz
GB 0
PC 1.00

Supplementary Figure 91. ^{1}H NMR spectrum for 3k

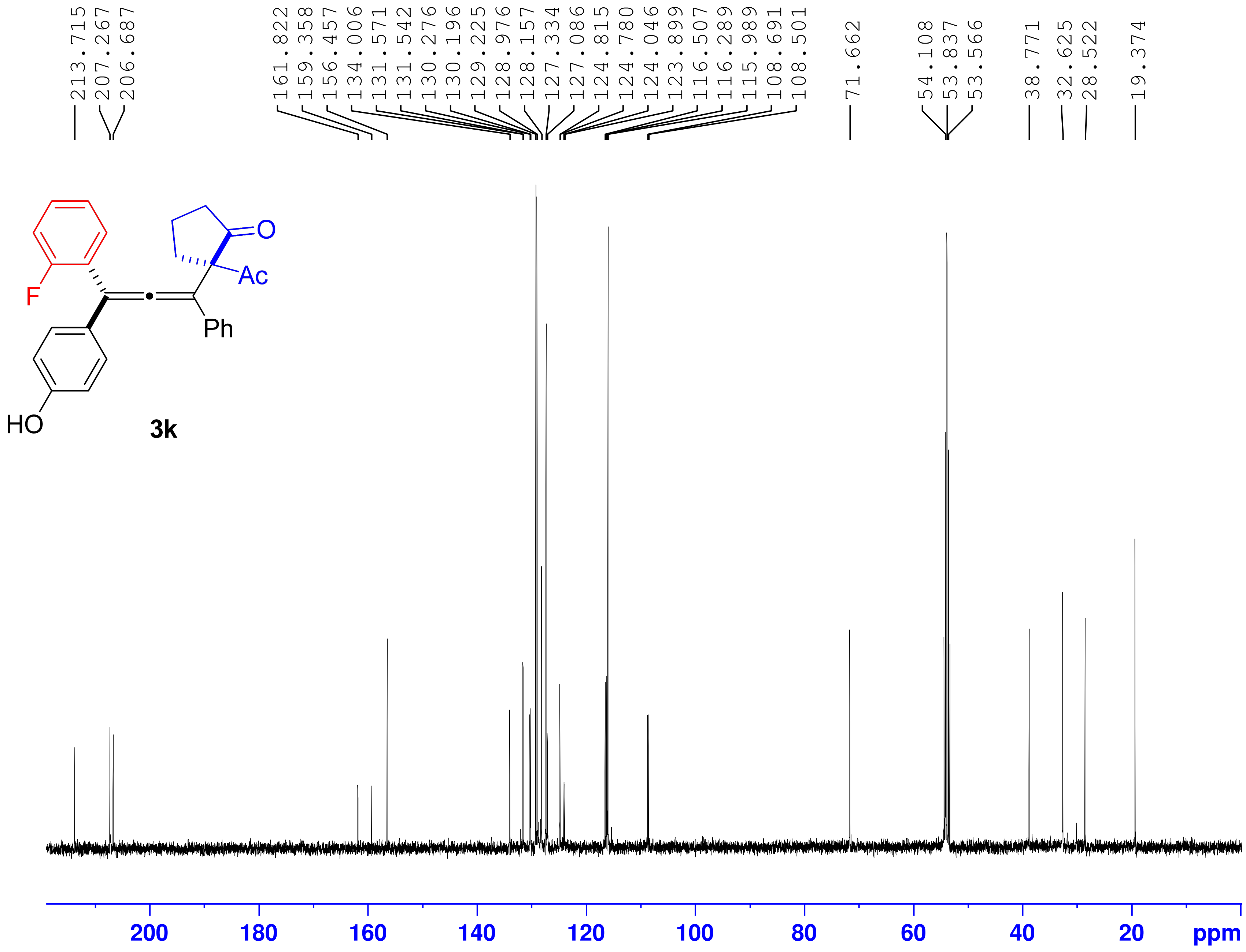


```
Current Data Parameters
NAME       qdy-20093-2 C
EXPNO                  1
PROCNO                 1

F2 - Acquisition Parameters
Date_           20160326
Time               13.52
INSTRUM            spect
PROBHD    5 mm PABBO BB/
PULPROG          zgpg30
TD                 65536
SOLVENT          CD2Cl2
NS                    96
DS                     0
SWH            24038.461 Hz
FIDRES          0.366798 Hz
AQ             1.3631488 sec
RG                196.92
DW                20.800 usec
DE                  6.50 usec
TE                 297.8 K
D1            2.00000000 sec
D11           0.03000000 sec
TD0                    1

======== CHANNEL f1 ========
SFO1         100.6228298 MHz
NUC1                 13C
P1                  9.70 usec
PLW1         46.98899841 W

======== CHANNEL f2 ========
SFO2         400.1316005 MHz
NUC2                  1H
CPDPRG[2         waltz16
PCPD2              90.00 usec
PLW2         11.99499989 W
PLW12         0.34213999 W
PLW13         0.27713001 W

F2 - Processing parameters
SI                 32768
SF           100.6127307 MHz
WDW                   EM
SSB       0
LB                  1.00 Hz
GB        0
PC                  1.40
```

Supplementary Figure 92. ^{13}C NMR spectrum for 3k

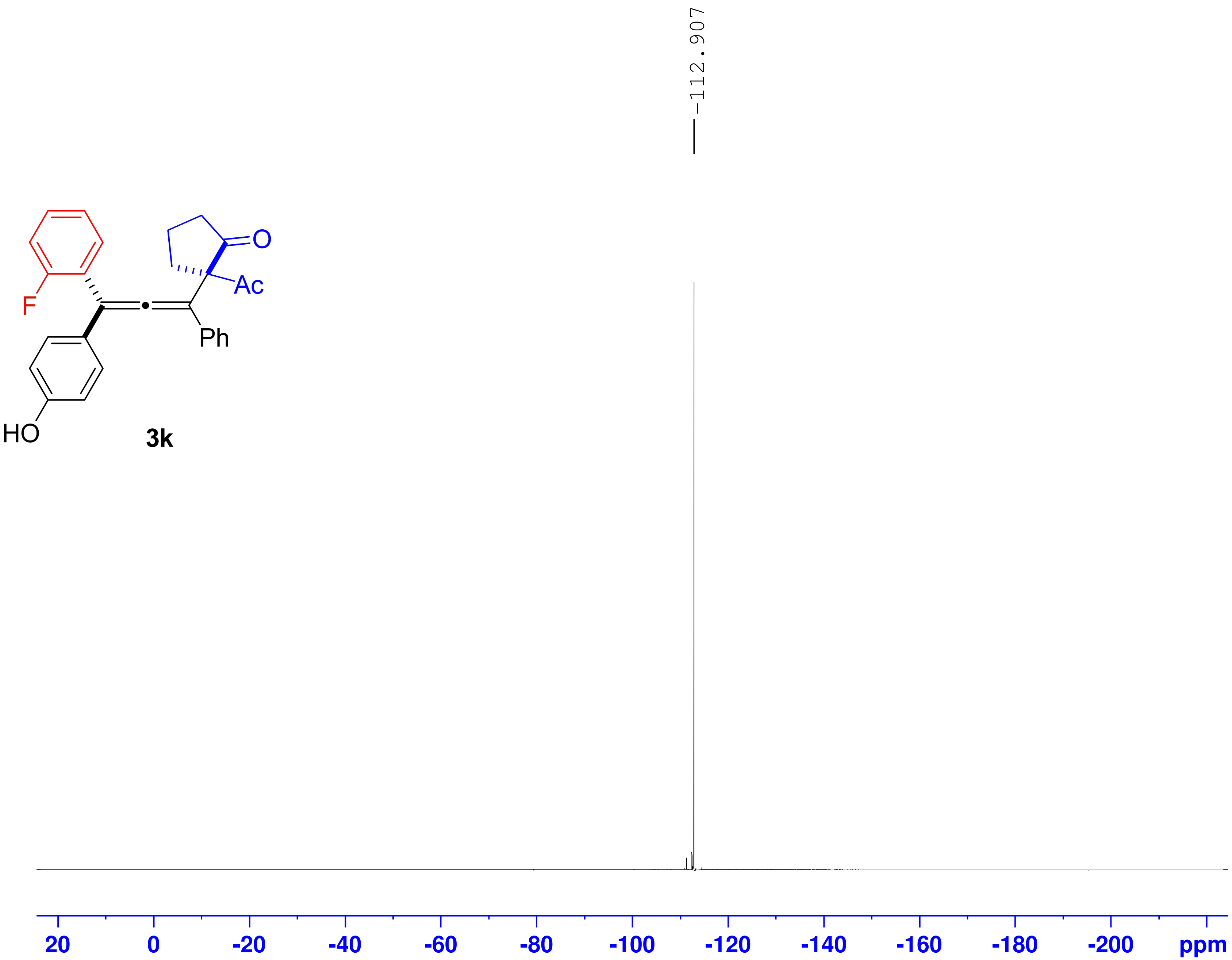


```
Current Data Parameters
NAME        qdy-20093-2 F
EXPNO                   1
PROCNO                  1

F2 - Acquisition Parameters
Date_            20160326
Time                13.56
INSTRUM             spect
PROBHD    5 mm PABBO BB/
PULPROG           zgpg30
TD                 65536
SOLVENT           CD2Cl2
NS                    35
DS                     2
SWH           93750.000 Hz
FIDRES         1.430511 Hz
AQ            0.3495253 sec
RG               196.92
DW                5.333 usec
DE                 6.50 usec
TE                297.6 K
D1           2.00000000 sec
D11          0.03000000 sec
TD0                    1

======== CHANNEL f1 ========
SFO1        376.4607162 MHz
NUC1                 19F
P1                 14.70 usec
PLW1         15.99600029 W

======== CHANNEL f2 ========
SFO2        400.1316005 MHz
NUC2                  1H
CPDPRG[2         waltz16
PCPD2              90.00 usec
PLW2         11.99499989 W
PLW12         0.34213999 W
PLW13         0.27713001 W

F2 - Processing parameters
SI                 32768
SF           376.4983660 MHz
WDW                   EM
SSB      0
LB                  1.00 Hz
GB       0
PC                  1.40
```

Supplementary Figure 93. ^{19}F NMR spectrum for 3k

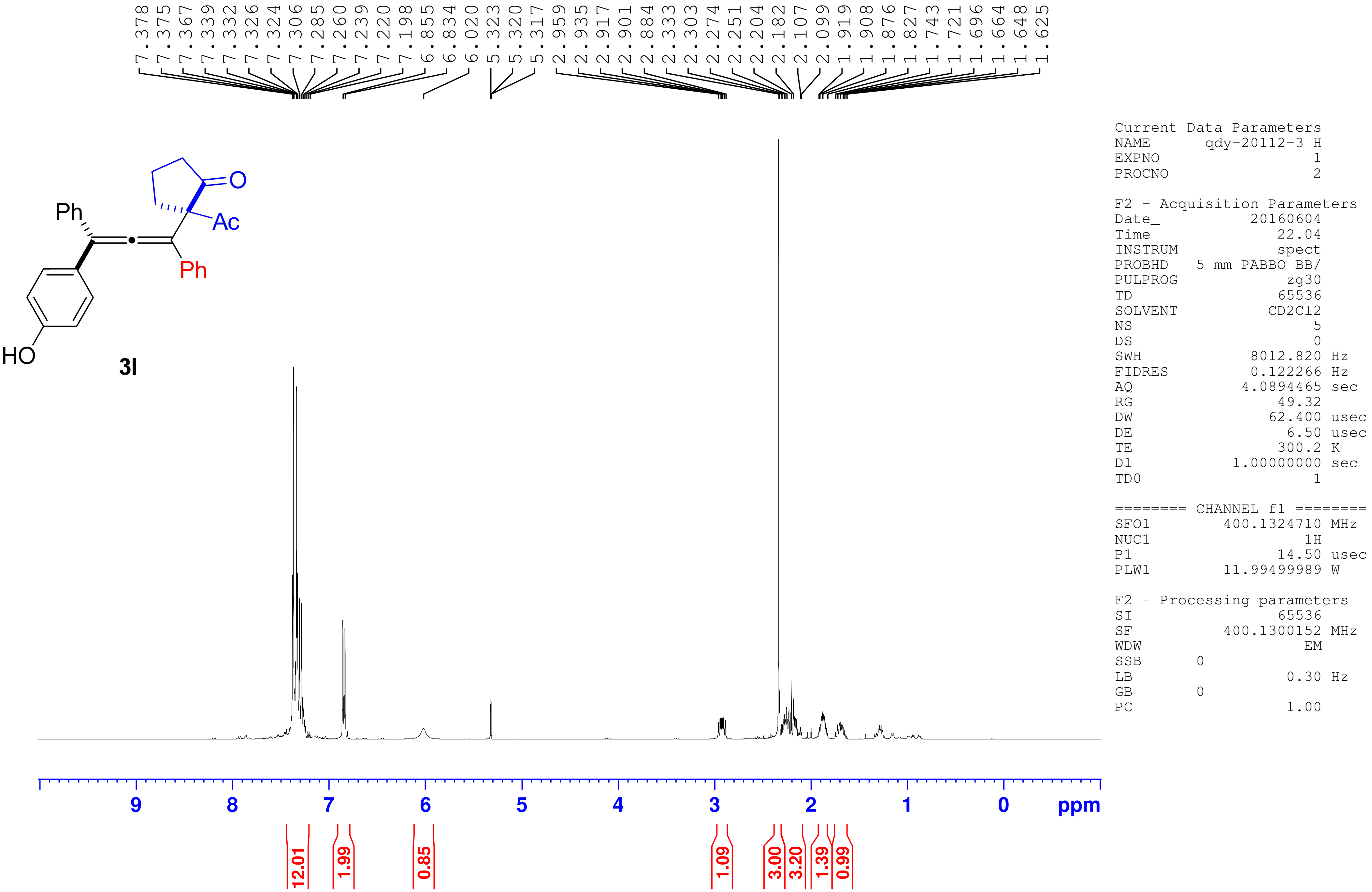

Supplementary Figure 94. ¹H NMR spectrum for 3l

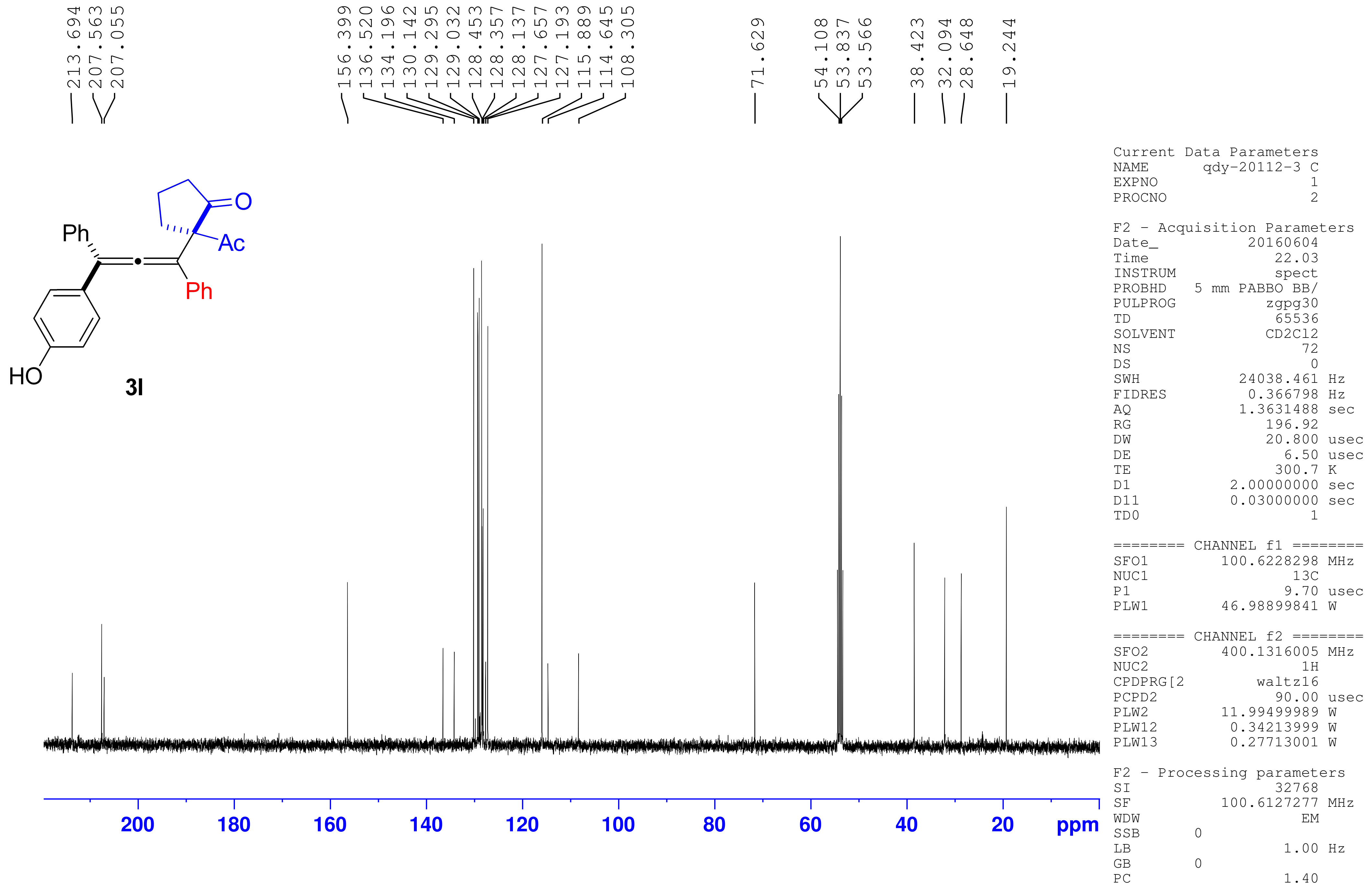

Supplementary Figure 95. ^{13}C NMR spectrum for 3l

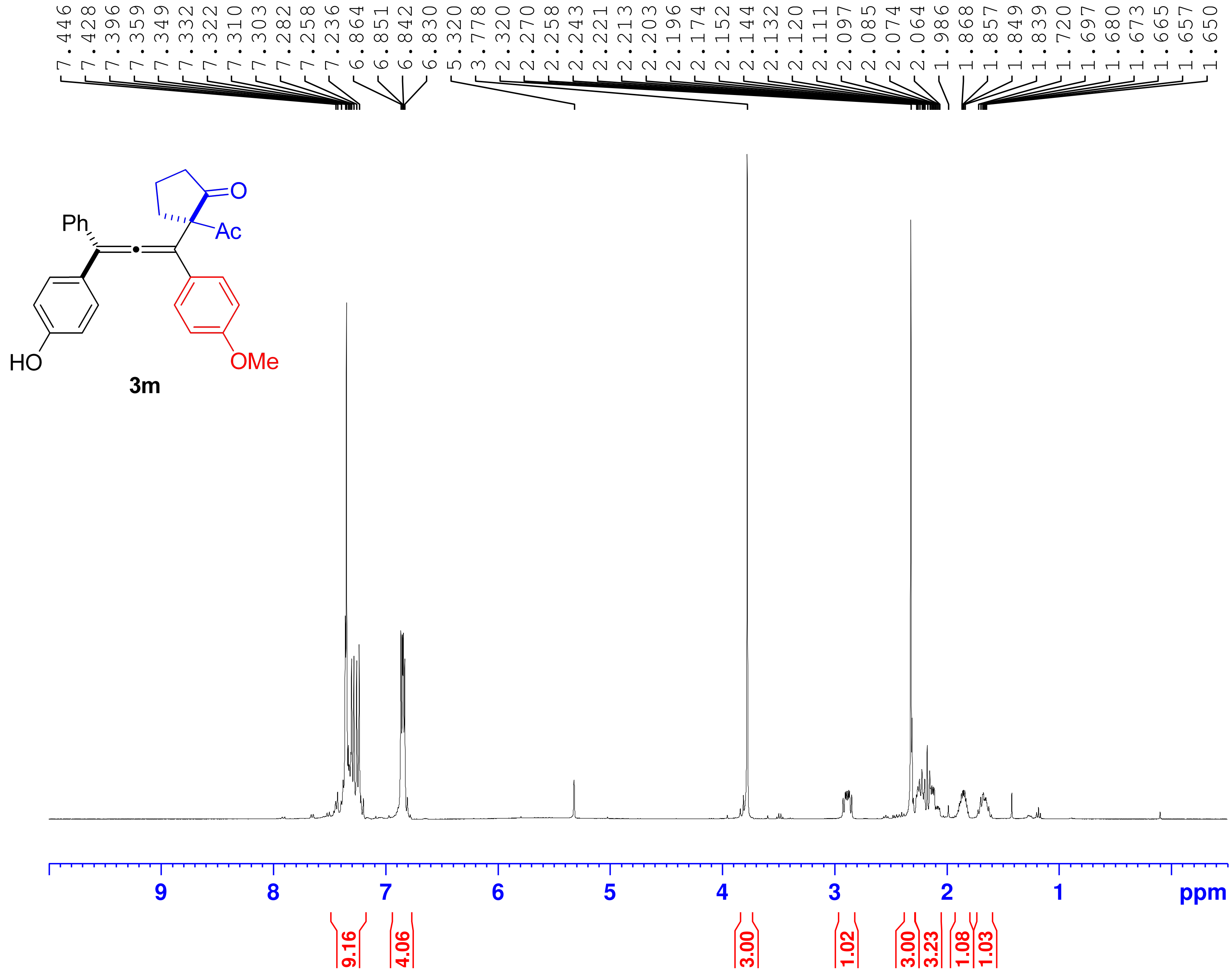


```
Current Data Parameters
NAME       qdy-20012-2 H
EXPNO                  3
PROCNO                 1

F2 - Acquisition Parameters
Date_           20160128
Time               21.45
INSTRUM            spect
PROBHD    5 mm PABBO BB/
PULPROG             zg30
TD                 65536
SOLVENT           CD2Cl2
NS                     6
DS                     0
SWH             8012.820 Hz
FIDRES          0.122266 Hz
AQ             4.0894465 sec
RG                 70.97
DW                62.400 usec
DE                  6.50 usec
TE                 296.6 K
D1            1.00000000 sec
TD0                    1

======== CHANNEL f1 ========
SFO1         400.1324710 MHz
NUC1                  1H
P1                 14.50 usec
PLW1         11.99499989 W

F2 - Processing parameters
SI                 65536
SF           400.1300149 MHz
WDW                   EM
SSB       0
LB                  0.30 Hz
GB        0
PC                  1.00
```

Supplementary Figure 96. ^{1}H NMR spectrum for 3m

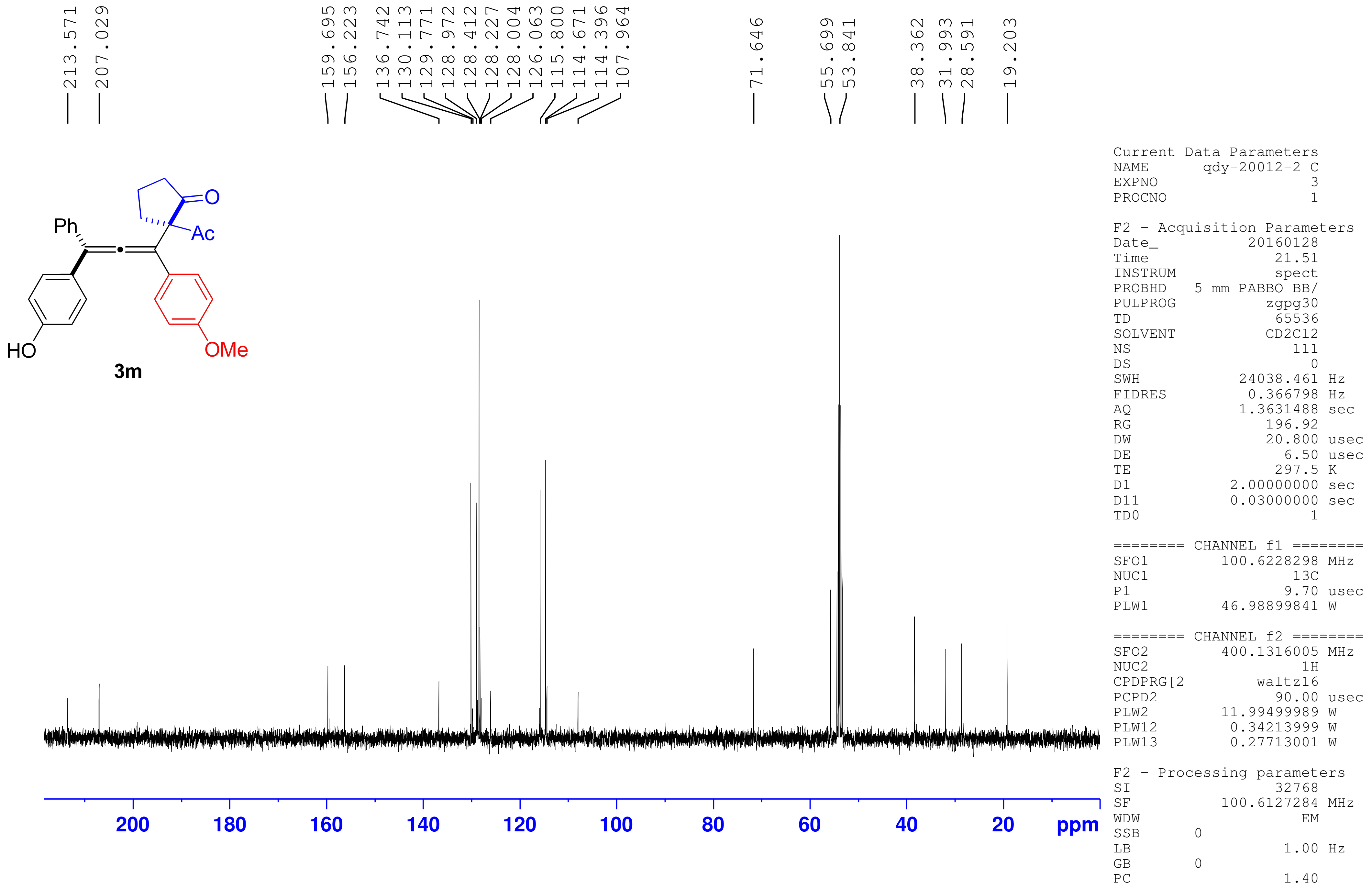

Supplementary Figure 97. ^{13}C NMR spectrum for 3m

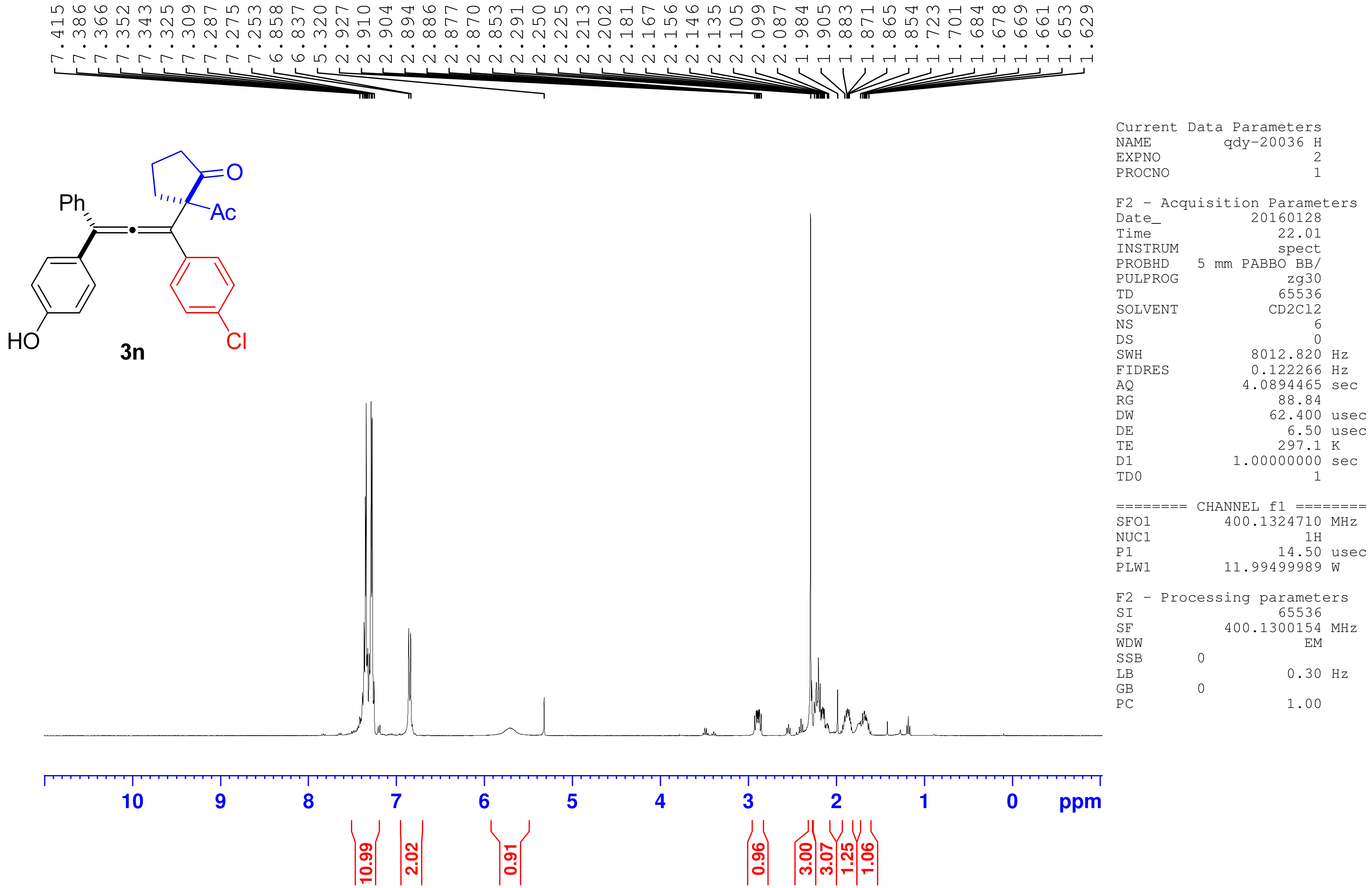

Supplementary Figure 98. ^{1}H NMR spectrum for 3n

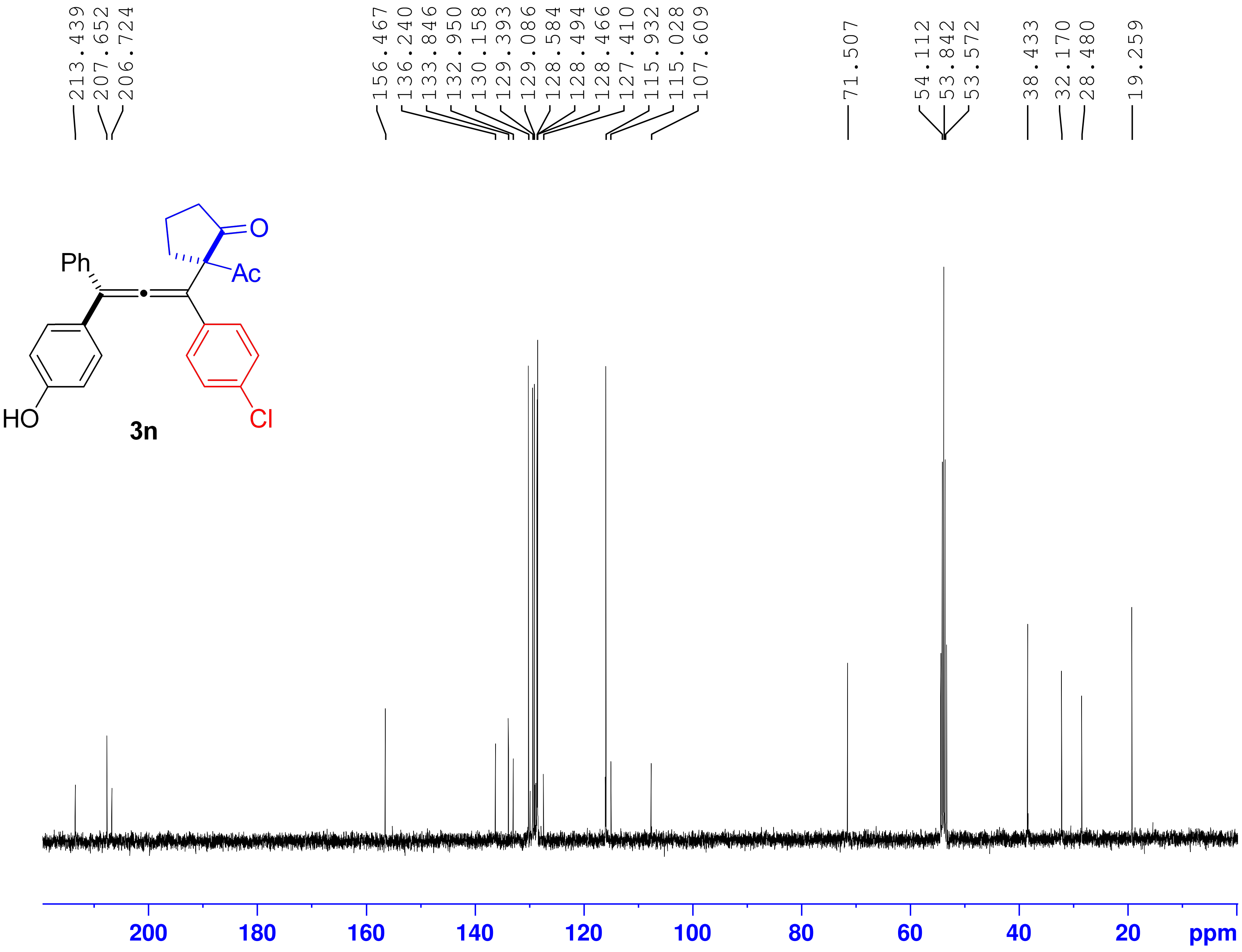

Current Data Parameters
NAME qdy-20036- C
EXPNO 1
PROCNO 1

F2 - Acquisition Parameters
Date_ 20160627
Time 21.46
INSTRUM spect
PROBHD 5 mm PABBO BB/
PULPROG zgpg30
TD 65536
SOLVENT CD2Cl2
NS 56
DS 0
SWH 24038.461 Hz
FIDRES 0.366798 Hz
AQ 1.3631488 sec
RG 196.92
DW 20.800 usec
DE 6.50 usec
TE 300.0 K
D1 2.00000000 sec
D11 0.03000000 sec
TD0 1

======== CHANNEL f1 ========
SFO1 100.6228298 MHz
NUC1 13C
P1 9.70 usec
PLW1 46.98899841 W

======== CHANNEL f2 ========
SFO2 400.1316005 MHz
NUC2 1H
CPDPRG[2 waltz16
PCPD2 90.00 usec
PLW2 11.99499989 W
PLW12 0.34213999 W
PLW13 0.27713001 W

F2 - Processing parameters
SI 32768
SF 100.6127277 MHz
WDW EM
SSB 0
LB 1.00 Hz
GB 0
PC 1.40

Supplementary Figure 99. ^{13}C NMR spectrum for 3n

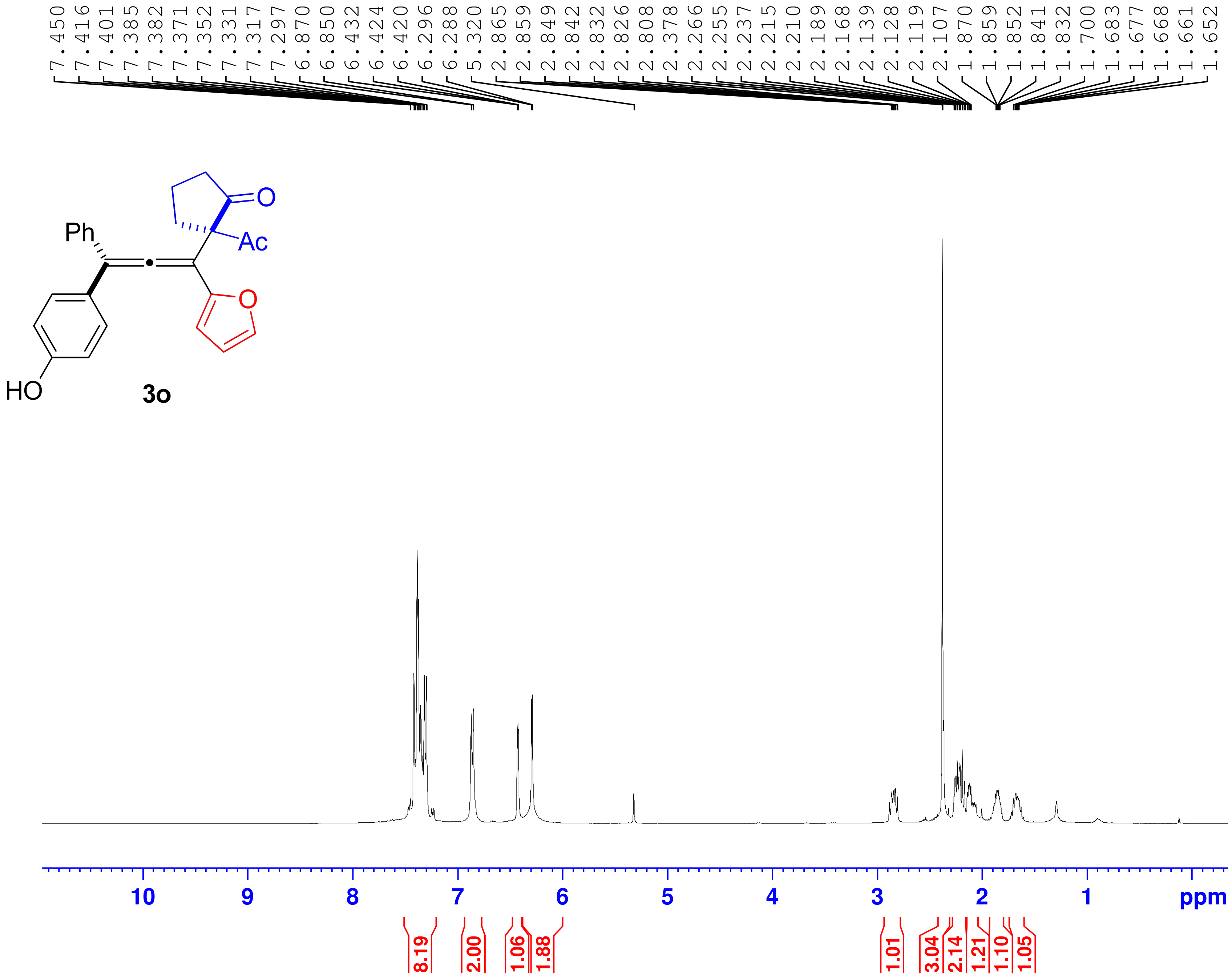

Current Data Parameters	
NAME	qdy-200881-1 H
EXPNO	1
PROCNO	1
F2 - Acquisition Parameters	
Date_	20160318
Time	21.33
INSTRUM	spect
PROBHD	5 mm PABBO BB/
PULPROG	zg30
TD	65536
SOLVENT	CD2Cl2
NS	10
DS	0
SWH	8012.820 Hz
FIDRES	0.122266 Hz
AQ	4.0894465 sec
RG	34.77
DW	62.400 usec
DE	6.50 usec
TE	296.9 K
D1	1.00000000 sec
TD0	1
======== CHANNEL f1 ========	
SFO1	400.1324710 MHz
NUC1	1H
P1	14.50 usec
PLW1	11.99499989 W
F2 - Processing parameters	
SI	65536
SF	400.1300149 MHz
WDW	EM
SSB	0
LB	0.30 Hz
GB	0
PC	1.00

Supplementary Figure 100. ^{1}H NMR spectrum for 3o

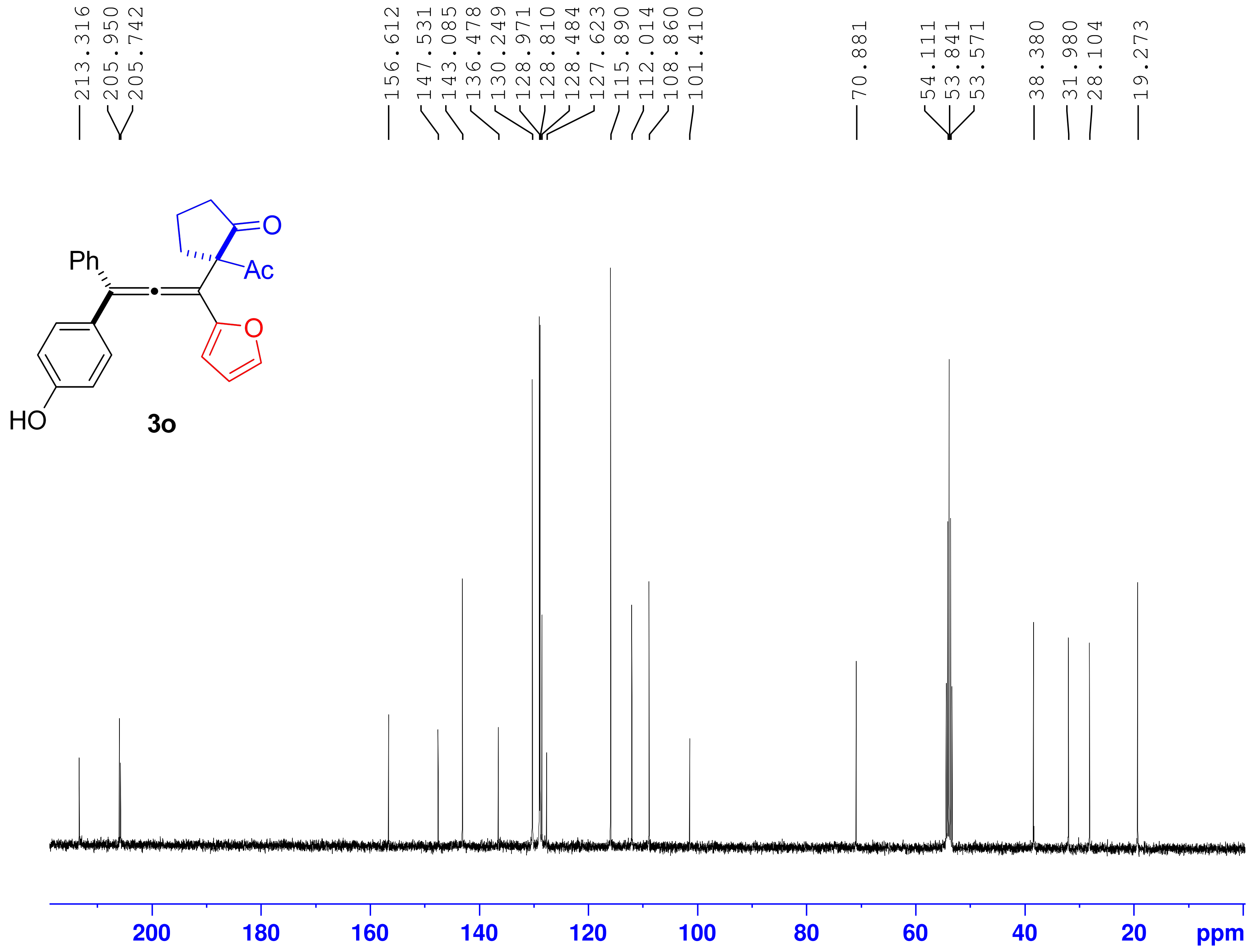


```
Current Data Parameters
NAME      qdy-200881-1 C
EXPNO                  1
PROCNO                 1

F2 - Acquisition Parameters
Date_           20160318
Time               21.37
INSTRUM            spect
PROBHD    5 mm PABBO BB/
PULPROG          zgpg30
TD                 65536
SOLVENT           CD2Cl2
NS                   115
DS                     0
SWH            24038.461 Hz
FIDRES          0.366798 Hz
AQ             1.3631488 sec
RG                196.92
DW                20.800 usec
DE                  6.50 usec
TE                 297.8 K
D1            2.00000000 sec
D11           0.03000000 sec
TD0                    1

======== CHANNEL f1 ========
SFO1         100.6228298 MHz
NUC1                 13C
P1                  9.70 usec
PLW1         46.98899841 W

======== CHANNEL f2 ========
SFO2         400.1316005 MHz
NUC2                  1H
CPDPRG[2         waltz16
PCPD2              90.00 usec
PLW2         11.99499989 W
PLW12         0.34213999 W
PLW13         0.27713001 W

F2 - Processing parameters
SI                 32768
SF           100.6127299 MHz
WDW                   EM
SSB       0
LB                  1.00 Hz
GB        0
PC                  1.40
```

Supplementary Figure 101. ^{13}C NMR spectrum for 3o

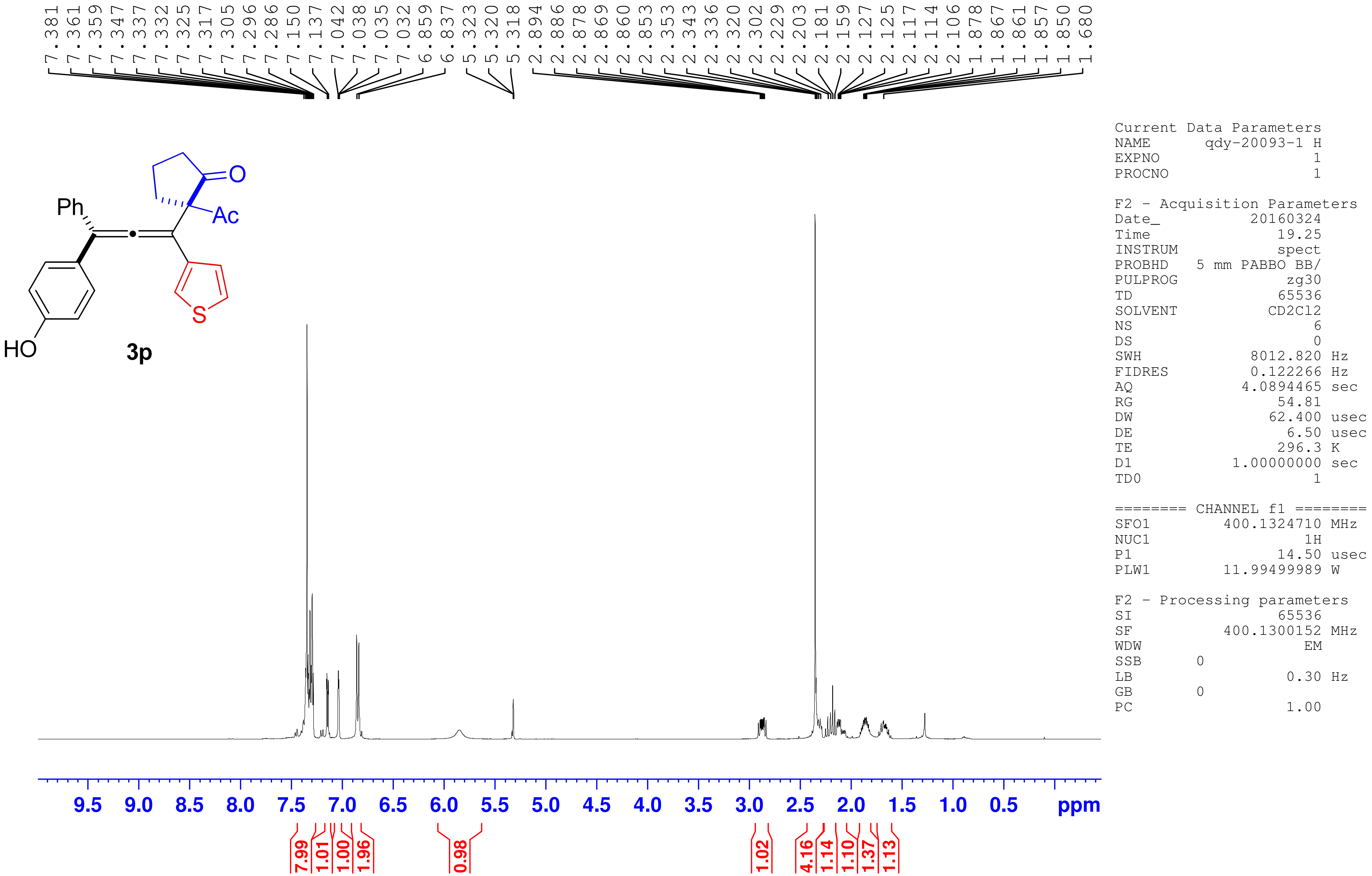

Supplementary Figure 102. ^{1}H NMR spectrum for 3p

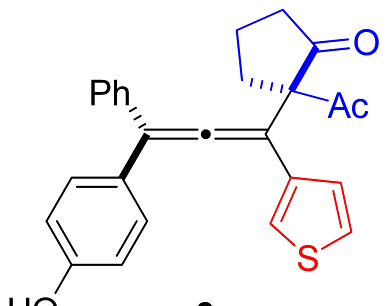

3p

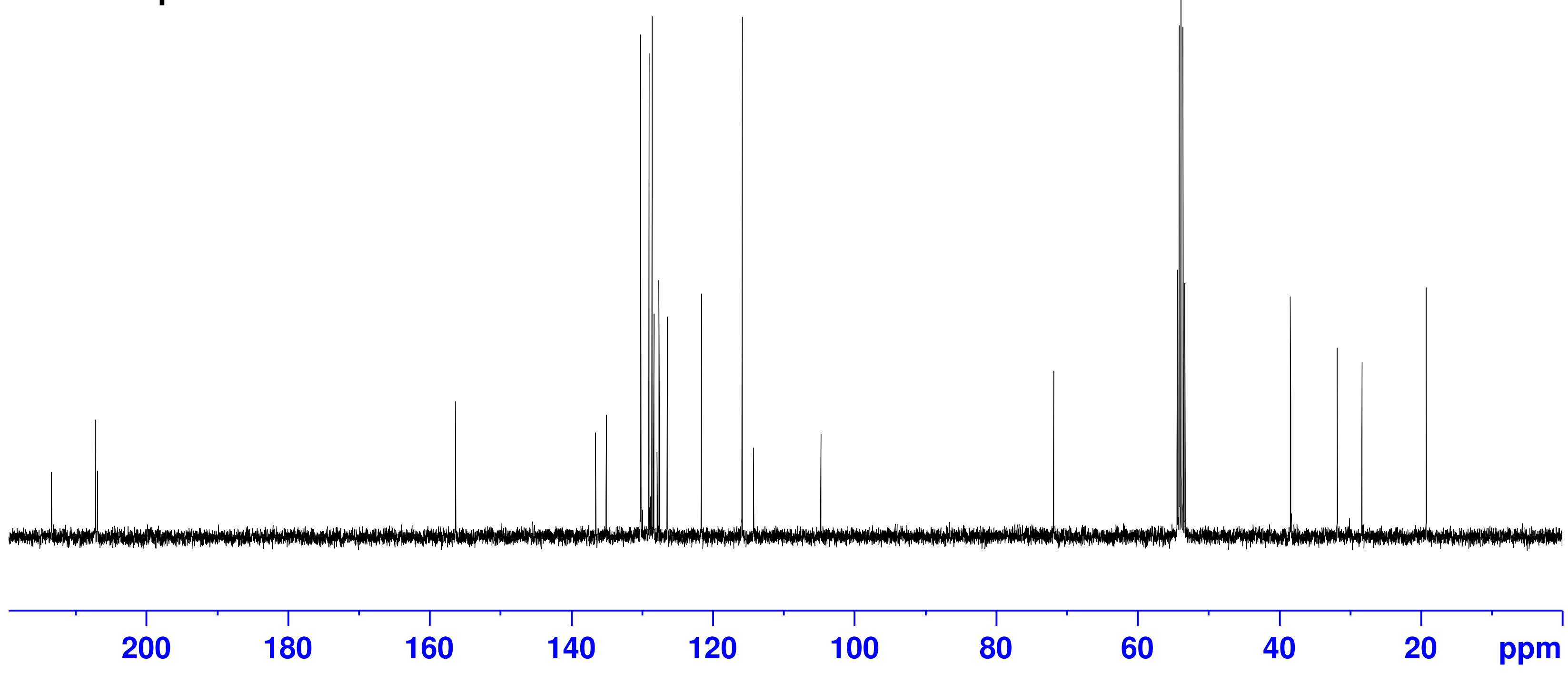

Current Data Parameters
NAME qdy-20093-1 C
EXPNO 1
PROCNO 1

F2 - Acquisition Parameters
Date_ 20160324
Time 19.30
INSTRUM spect
PROBHD 5 mm PABBO BB/
PULPROG zgpg30
TD 65536
SOLVENT CD2Cl2
NS 102
DS 0
SWH 24038.461 Hz
FIDRES 0.366798 Hz
AQ 1.3631488 sec
RG 196.92
DW 20.800 usec
DE 6.50 usec
TE 297.4 K
D1 2.00000000 sec
D11 0.03000000 sec
TD0 1

======== CHANNEL f1 ========
SFO1 100.6228298 MHz
NUC1 13C
P1 9.70 usec
PLW1 46.98899841 W

======== CHANNEL f2 ========
SFO2 400.1316005 MHz
NUC2 1H
CPDPRG[2 waltz16
PCPD2 90.00 usec
PLW2 11.99499989 W
PLW12 0.34213999 W
PLW13 0.27713001 W

F2 - Processing parameters
SI 32768
SF 100.6127292 MHz
WDW EM
SSB 0
LB 1.00 Hz
GB 0
PC 1.40

Supplementary Figure 103. ^{13}C NMR spectrum for 3p

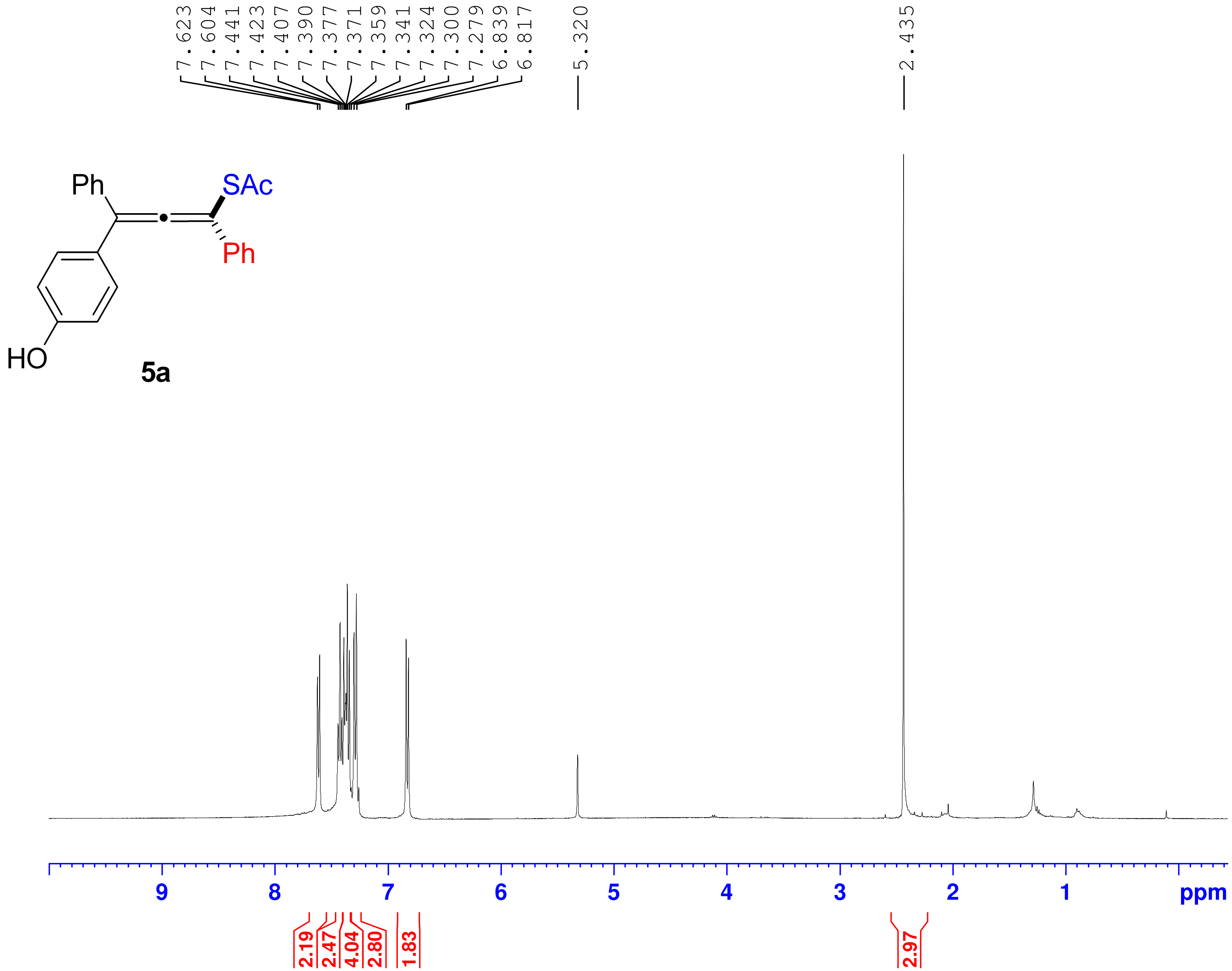


```
Current Data Parameters
NAME        qdy-20087-2 H
EXPNO                   2
PROCNO                  1

F2 - Acquisition Parameters
Date_            20160318
Time                21.57
INSTRUM             spect
PROBHD    5 mm PABBO BB/
PULPROG              zg30
TD                  65536
SOLVENT            CD2Cl2
NS                      7
DS                      0
SWH              8012.820 Hz
FIDRES           0.122266 Hz
AQ              4.0894465 sec
RG                  82.92
DW                 62.400 usec
DE                   6.50 usec
TE                  297.5 K
D1             1.00000000 sec
TD0                     1

======== CHANNEL f1 ========
SFO1          400.1324710 MHz
NUC1                   1H
P1                  14.50 usec
PLW1          11.99499989 W

F2 - Processing parameters
SI                  65536
SF            400.1300151 MHz
WDW                    EM
SSB       0
LB                   0.30 Hz
GB        0
PC                   1.00
```

Supplementary Figure 104. ^{1}H NMR spectrum for 5a

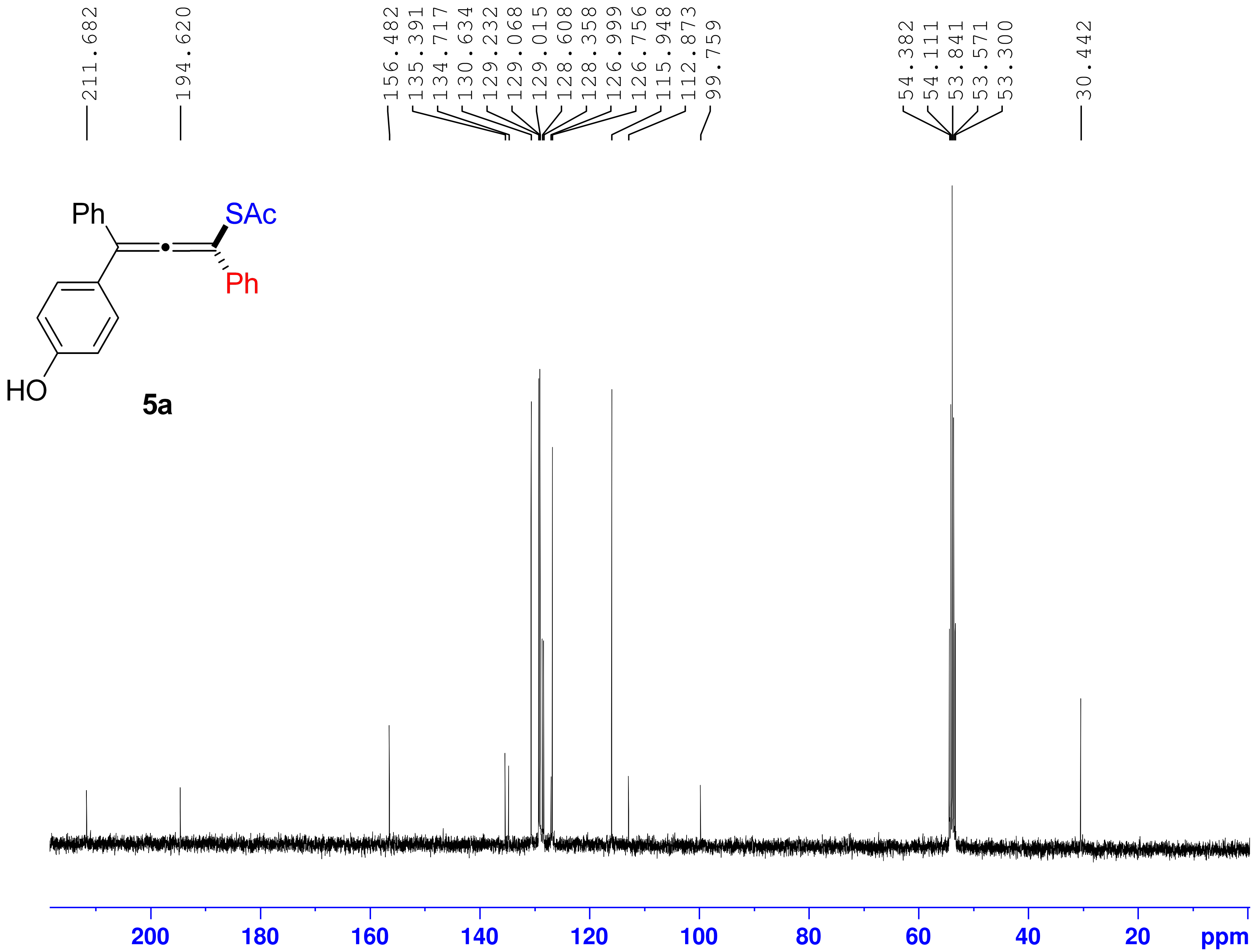

5a


```
Current Data Parameters
NAME       qdy-20087-2 C
EXPNO                  2
PROCNO                 1

F2 - Acquisition Parameters
Date_           20160318
Time               22.04
INSTRUM            spect
PROBHD    5 mm PABBO BB/
PULPROG          zgpg30
TD                 65536
SOLVENT           CD2Cl2
NS                   168
DS                     0
SWH            24038.461 Hz
FIDRES          0.366798 Hz
AQ             1.3631488 sec
RG                196.92
DW                20.800 usec
DE                  6.50 usec
TE                 299.2 K
D1            2.00000000 sec
D11           0.03000000 sec
TD0                    1

======== CHANNEL f1 ========
SFO1         100.6228298 MHz
NUC1                 13C
P1                  9.70 usec
PLW1         46.98899841 W

======== CHANNEL f2 ========
SFO2         400.1316005 MHz
NUC2                  1H
CPDPRG[2         waltz16
PCPD2              90.00 usec
PLW2         11.99499989 W
PLW12         0.34213999 W
PLW13         0.27713001 W

F2 - Processing parameters
SI                 32768
SF           100.6127277 MHz
WDW                   EM
SSB       0
LB                  1.00 Hz
GB        0
PC                  1.40
```

Supplementary Figure 105. ^{13}C NMR spectrum for 5a

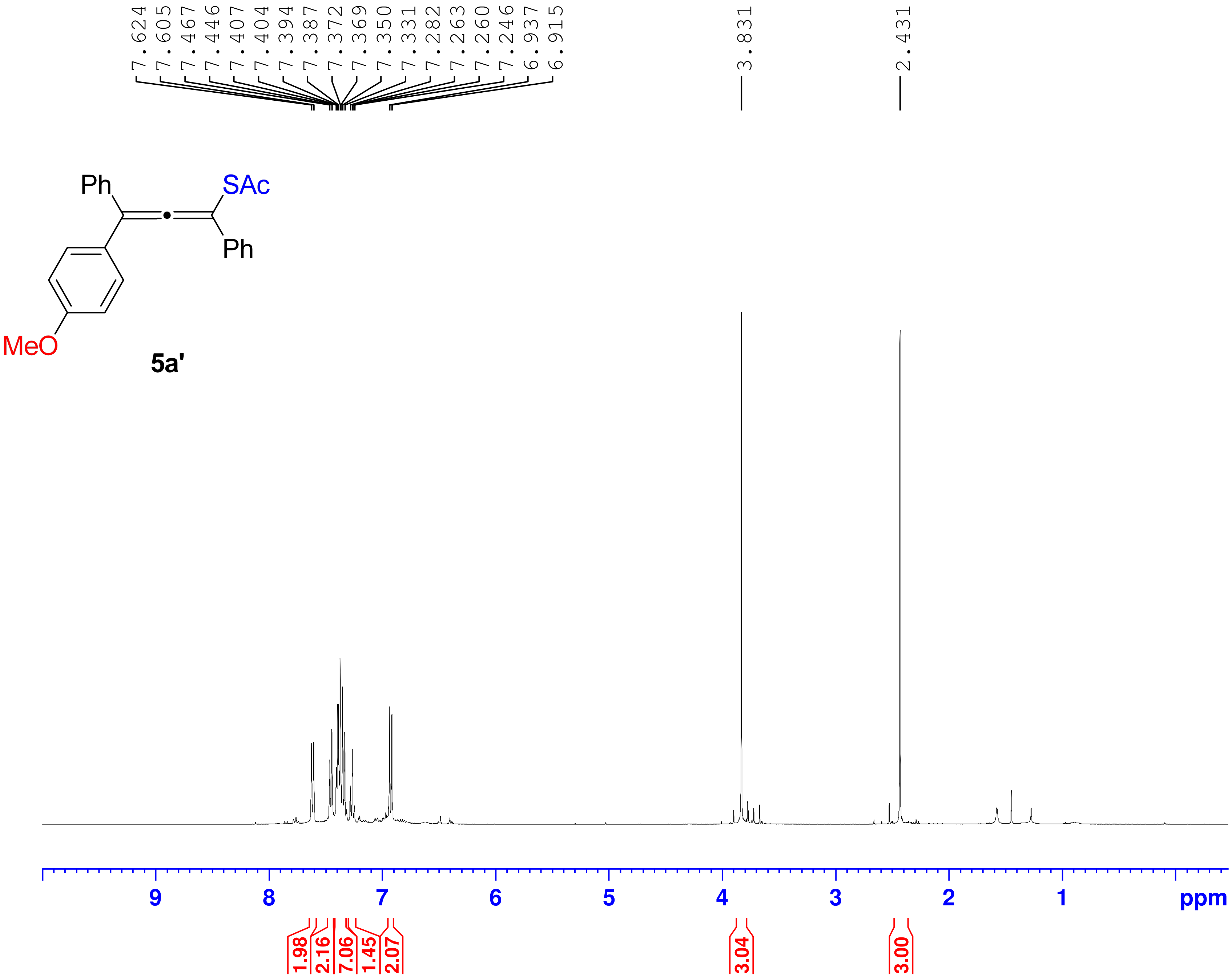

Current Data Parameters
NAME qdy-20150-2 H
EXPNO 2
PROCNO 1

F2 - Acquisition Parameters
Date_ 20160517
Time 15.13
INSTRUM spect
PROBHD 5 mm PABBO BB/
PULPROG zg30
TD 65536
SOLVENT CDCl3
NS 8
DS 0
SWH 8012.820 Hz
FIDRES 0.122266 Hz
AQ 4.0894465 sec
RG 88.84
DW 62.400 usec
DE 6.50 usec
TE 296.3 K
D1 1.00000000 sec
TD0 1

======== CHANNEL f1 ========
SFO1 400.1324710 MHz
NUC1 1H
P1 14.50 usec
PLW1 11.99499989 W

F2 - Processing parameters
SI 65536
SF 400.1300093 MHz
WDW EM
SSB 0
LB 0.30 Hz
GB 0
PC 1.00

Supplementary Figure 106. ^{1}H NMR spectrum for 5a'

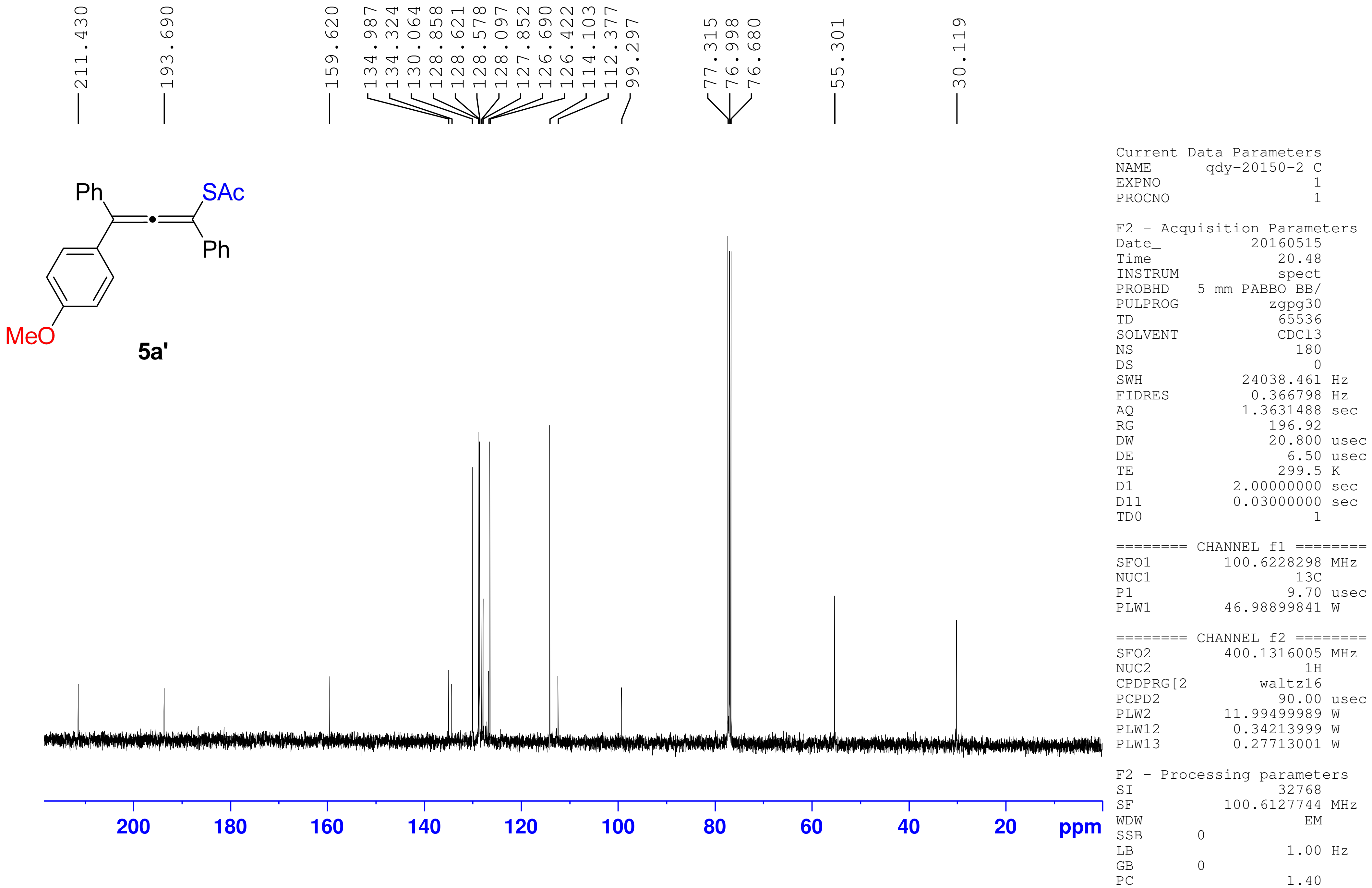

Supplementary Figure 107. ^{13}C NMR spectrum for 5a'

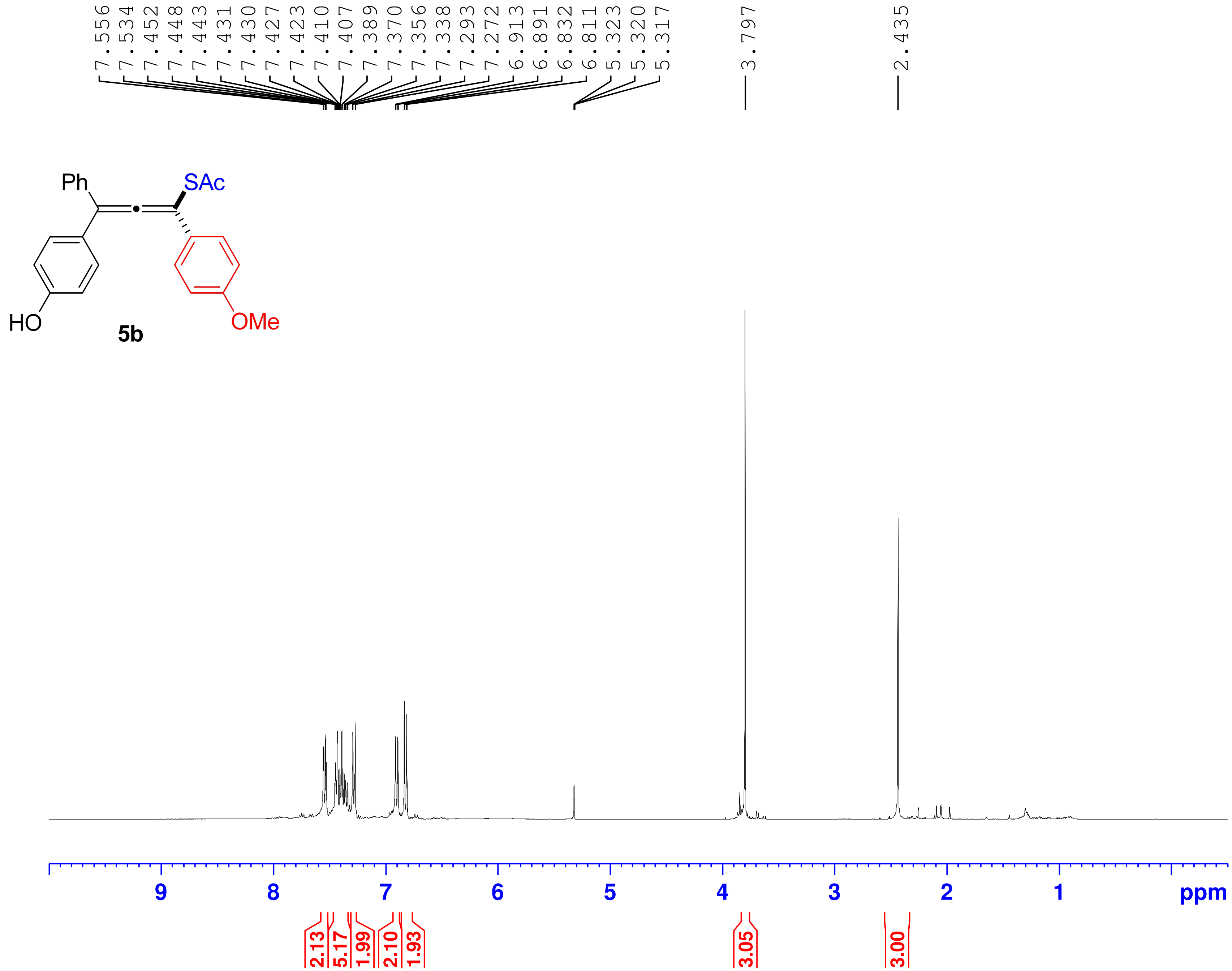


```
Current Data Parameters
NAME         qdy-20133-1 H
EXPNO                    1
PROCNO                   1

F2 - Acquisition Parameters
Date_             20160525
Time                 20.19
INSTRUM              spect
PROBHD    5 mm PABBO BB/
PULPROG               zg30
TD                   65536
SOLVENT             CD2Cl2
NS                       4
DS                       0
SWH               8012.820 Hz
FIDRES            0.122266 Hz
AQ               4.0894465 sec
RG                   54.81
DW                  62.400 usec
DE                    6.50 usec
TE                   297.6 K
D1              1.00000000 sec
TD0                      1

======== CHANNEL f1 ========
SFO1           400.1324710 MHz
NUC1                    1H
P1                   14.50 usec
PLW1           11.99499989 W

F2 - Processing parameters
SI                   65536
SF             400.1300152 MHz
WDW                     EM
SSB      0
LB                    0.30 Hz
GB       0
PC                    1.00
```

Supplementary Figure 108. ^{1}H NMR spectrum for 5b

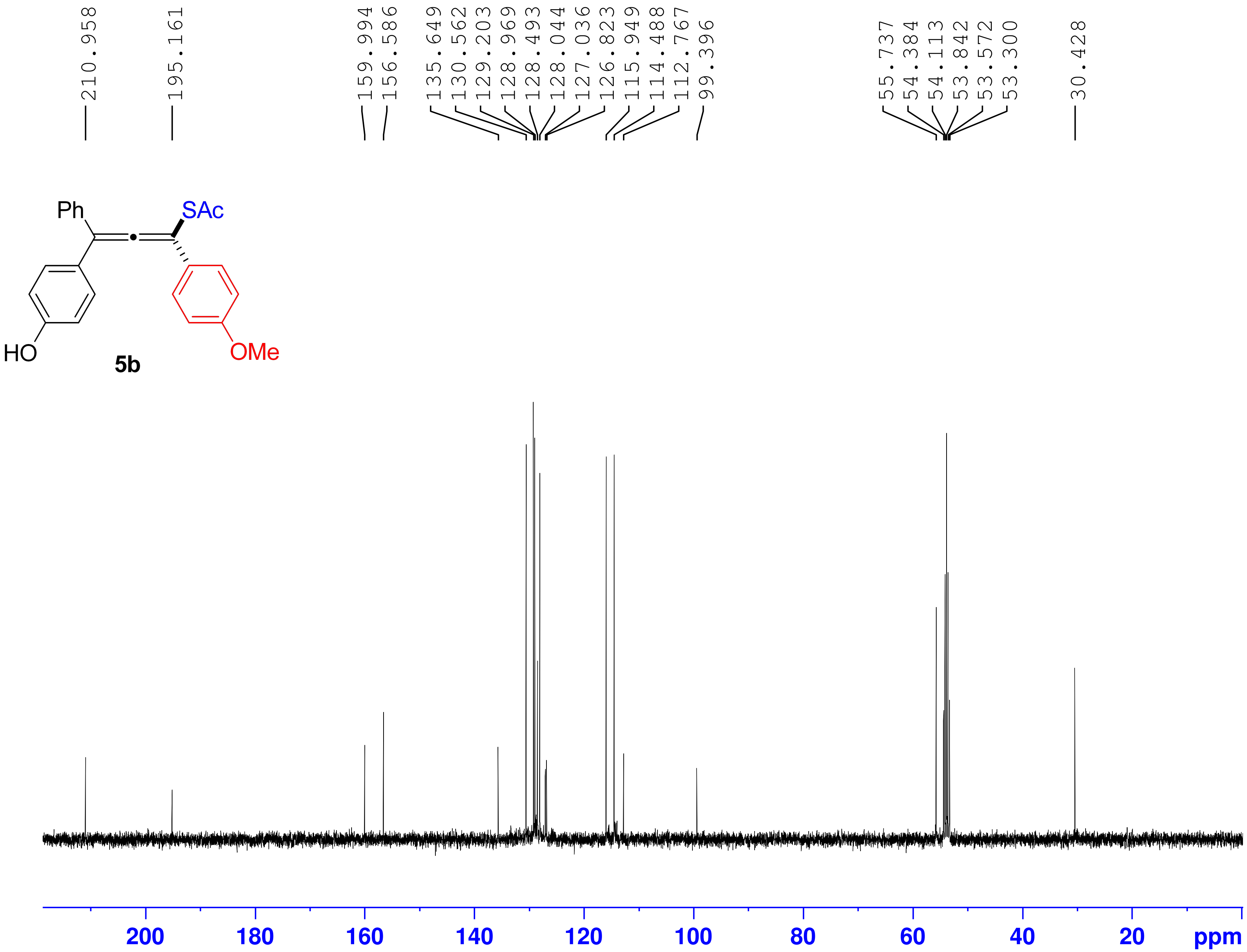

Current Data Parameters
NAME qdy-20133-1 C
EXPNO 1
PROCNO 1

F2 - Acquisition Parameters
Date_ 20160525
Time 13.26
INSTRUM spect
PROBHD 5 mm PABBO BB/
PULPROG zgpg30
TD 65536
SOLVENT CD2Cl2
NS 81
DS 0
SWH 24038.461 Hz
FIDRES 0.366798 Hz
AQ 1.3631488 sec
RG 196.92
DW 20.800 usec
DE 6.50 usec
TE 298.5 K
D1 2.00000000 sec
D11 0.03000000 sec
TD0 1

======== CHANNEL f1 ========
SFO1 100.6228298 MHz
NUC1 13C
P1 9.70 usec
PLW1 46.98899841 W

======== CHANNEL f2 ========
SFO2 400.1316005 MHz
NUC2 1H
CPDPRG[2 waltz16
PCPD2 90.00 usec
PLW2 11.99499989 W
PLW12 0.34213999 W
PLW13 0.27713001 W

F2 - Processing parameters
SI 32768
SF 100.6127314 MHz
WDW EM
SSB 0
LB 1.00 Hz
GB 0
PC 1.40

Supplementary Figure 109. ^{13}C NMR spectrum for 5b

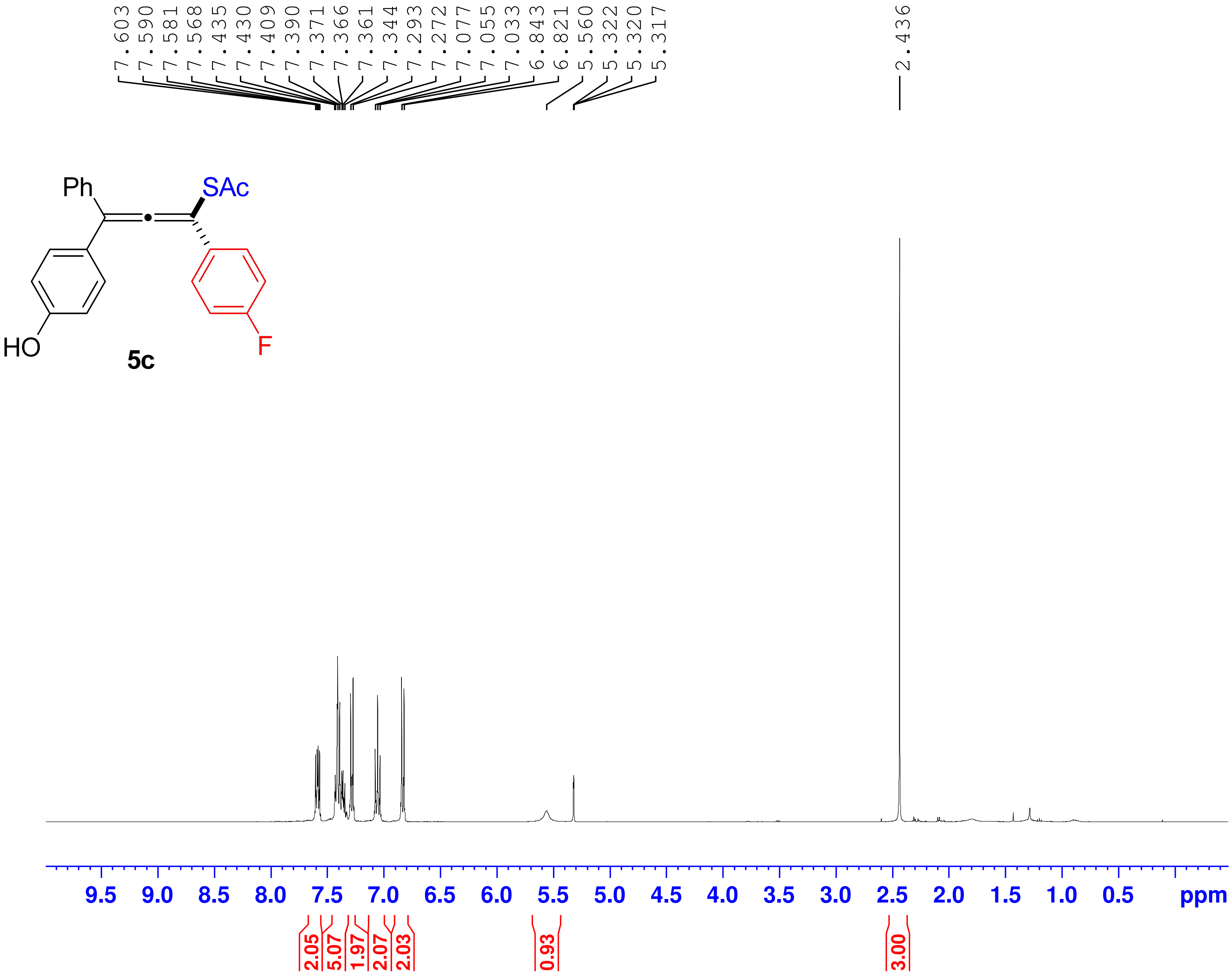

5c


```
Current Data Parameters
NAME      qdy-20144-1 H
EXPNO              2
PROCNO             1

F2 - Acquisition Parameters
Date_          20160511
Time              13.45
INSTRUM           spect
PROBHD   5 mm PABBO BB/
PULPROG            zg30
TD                65536
SOLVENT          CD2Cl2
NS                    9
DS                    0
SWH            8012.820 Hz
FIDRES         0.122266 Hz
AQ            4.0894465 sec
RG                88.84
DW               62.400 usec
DE                 6.50 usec
TE                297.1 K
D1           1.00000000 sec
TD0                   1

======== CHANNEL f1 ========
SFO1        400.1324710 MHz
NUC1                 1H
P1                14.50 usec
PLW1        11.99499989 W

F2 - Processing parameters
SI                65536
SF          400.1300153 MHz
WDW                  EM
SSB      0
LB                 0.30 Hz
GB       0
PC                 1.00
```

Supplementary Figure 110. ^{1}H NMR spectrum for 5c

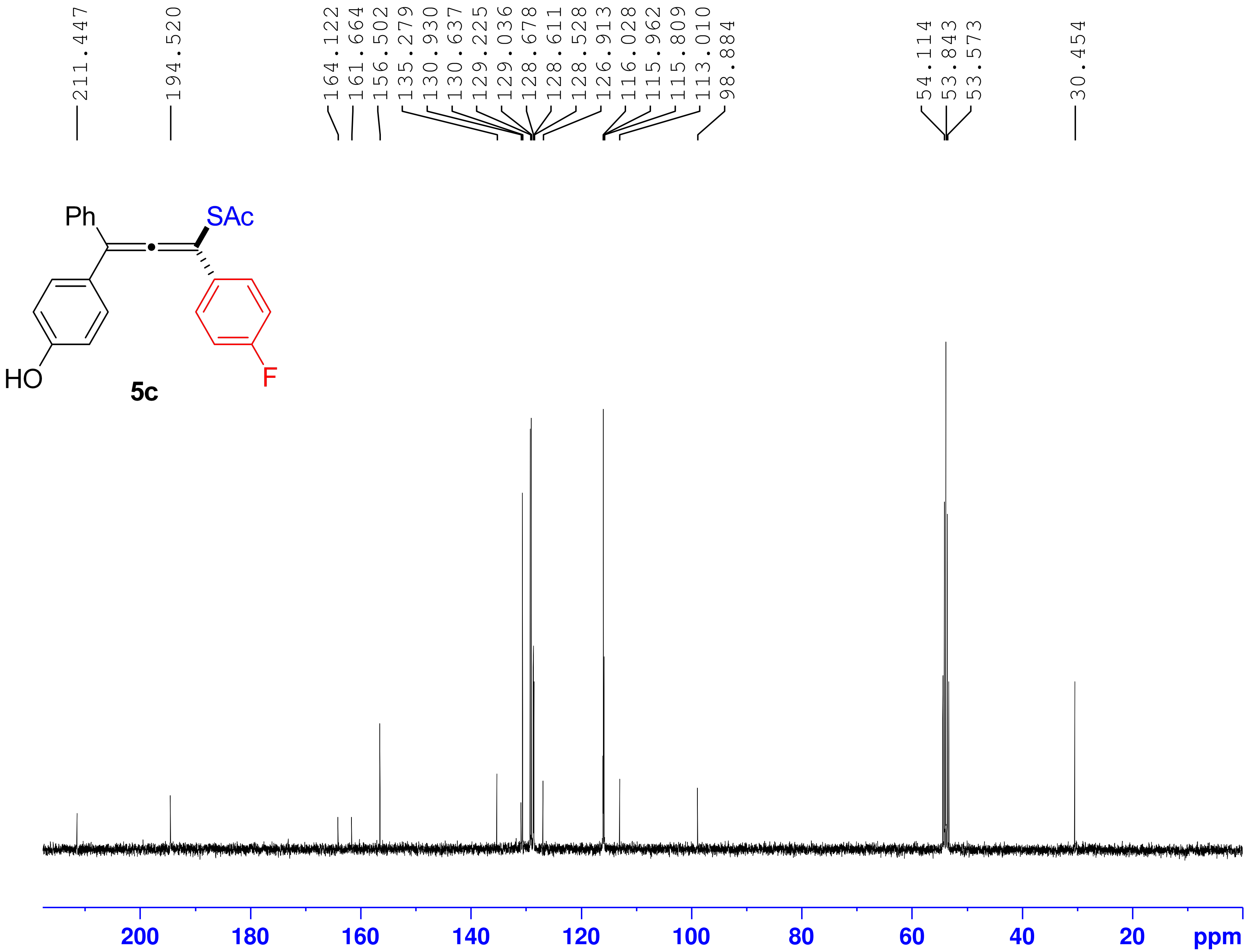


```
Current Data Parameters
NAME       qdy-20144-1 C
EXPNO                  1
PROCNO                 1

F2 - Acquisition Parameters
Date_           20160511
Time               13.33
INSTRUM            spect
PROBHD    5 mm PABBO BB/
PULPROG          zgpg30
TD                65536
SOLVENT          CD2Cl2
NS                  199
DS                    0
SWH           24038.461 Hz
FIDRES         0.366798 Hz
AQ            1.3631488 sec
RG               196.92
DW               20.800 usec
DE                 6.50 usec
TE                297.4 K
D1           2.00000000 sec
D11          0.03000000 sec
TD0                   1

======== CHANNEL f1 ========
SFO1        100.6228298 MHz
NUC1                13C
P1                 9.70 usec
PLW1        46.98899841 W

======== CHANNEL f2 ========
SFO2        400.1316005 MHz
NUC2                 1H
CPDPRG[2        waltz16
PCPD2             90.00 usec
PLW2        11.99499989 W
PLW12        0.34213999 W
PLW13        0.27713001 W

F2 - Processing parameters
SI                32768
SF          100.6127284 MHz
WDW                  EM
SSB      0
LB                 1.00 Hz
GB       0
PC                 1.40
```

Supplementary Figure 111. ^{13}C NMR spectrum for 5c

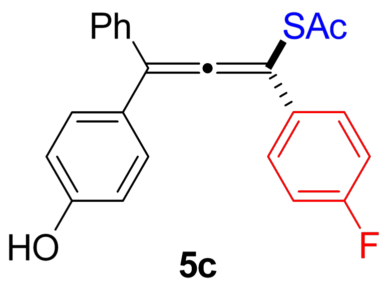

5c

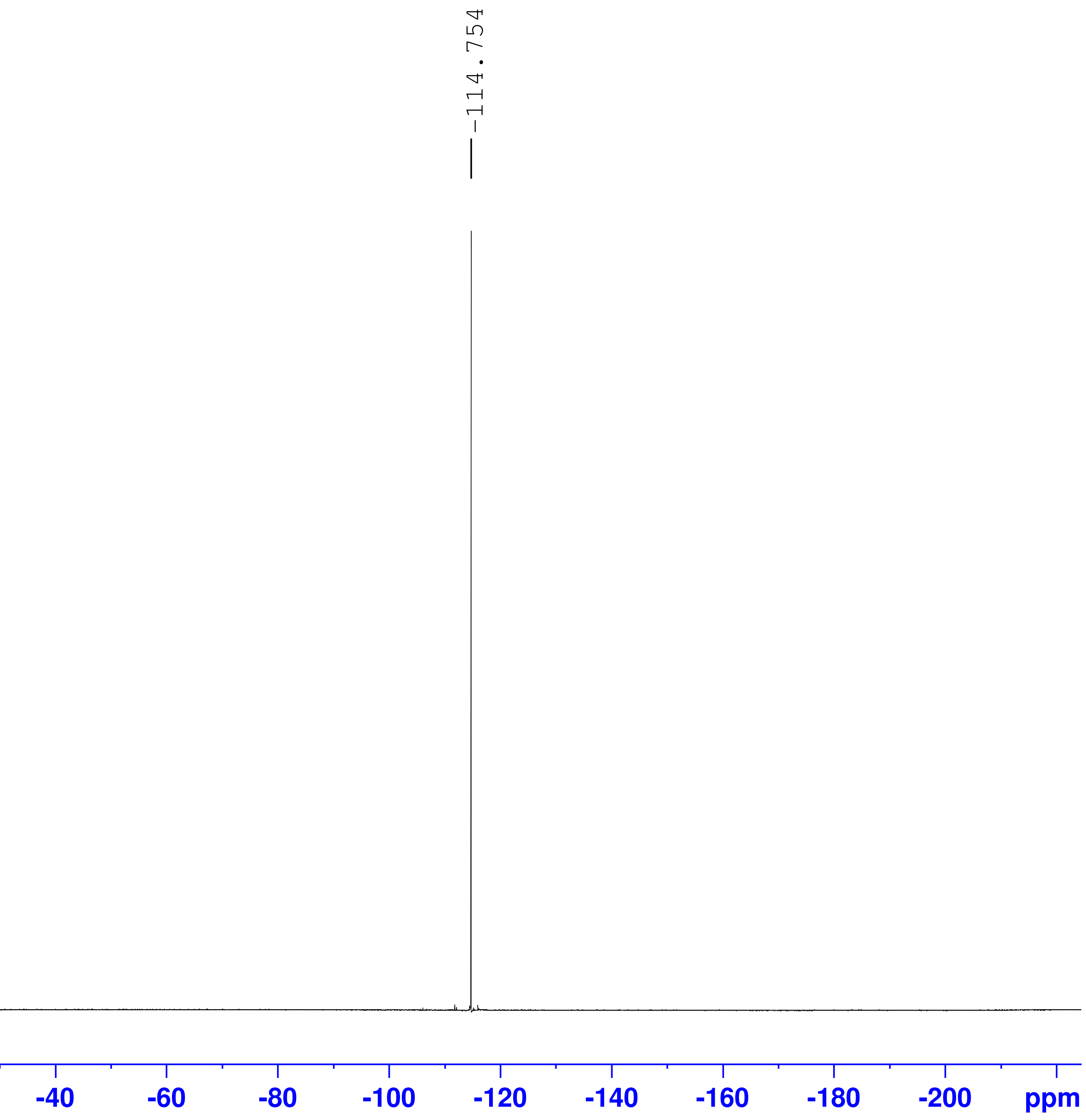


```
Current Data Parameters
NAME       qdy-20144-1 F
EXPNO                  1
PROCNO                 1

F2 - Acquisition Parameters
Date_           20160511
Time               13.50
INSTRUM            spect
PROBHD     5 mm PABBO BB/
PULPROG          zgpg30
TD                 65536
SOLVENT           CD2Cl2
NS                     4
DS                     0
SWH            93750.000 Hz
FIDRES          1.430511 Hz
AQ             0.3495253 sec
RG                196.92
DW                 5.333 usec
DE                  6.50 usec
TE                 296.7 K
D1            2.00000000 sec
D11           0.03000000 sec
TD0                    1

======== CHANNEL f1 ========
SFO1         376.4607162 MHz
NUC1                 19F
P1                 14.70 usec
PLW1         15.99600029 W

======== CHANNEL f2 ========
SFO2         400.1316005 MHz
NUC2                  1H
CPDPRG[2         waltz16
PCPD2              90.00 usec
PLW2         11.99499989 W
PLW12         0.34213999 W
PLW13         0.27713001 W

F2 - Processing parameters
SI                 32768
SF           376.4983660 MHz
WDW                   EM
SSB      0
LB                  1.00 Hz
GB       0
PC                  1.40
```

Supplementary Figure 112. ^{19}F NMR spectrum for 5c

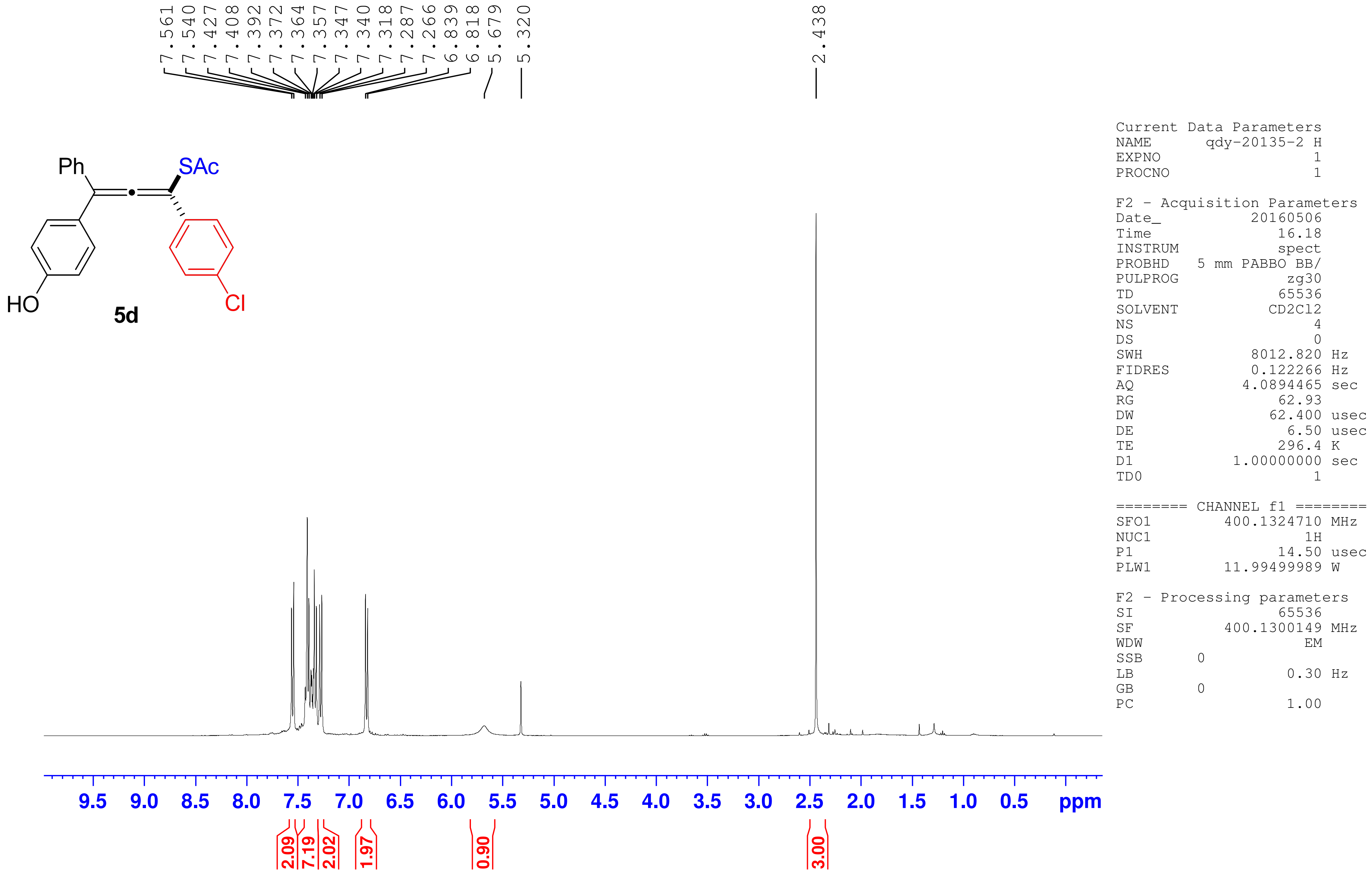

Supplementary Figure 113. ^{1}H NMR spectrum for 5d

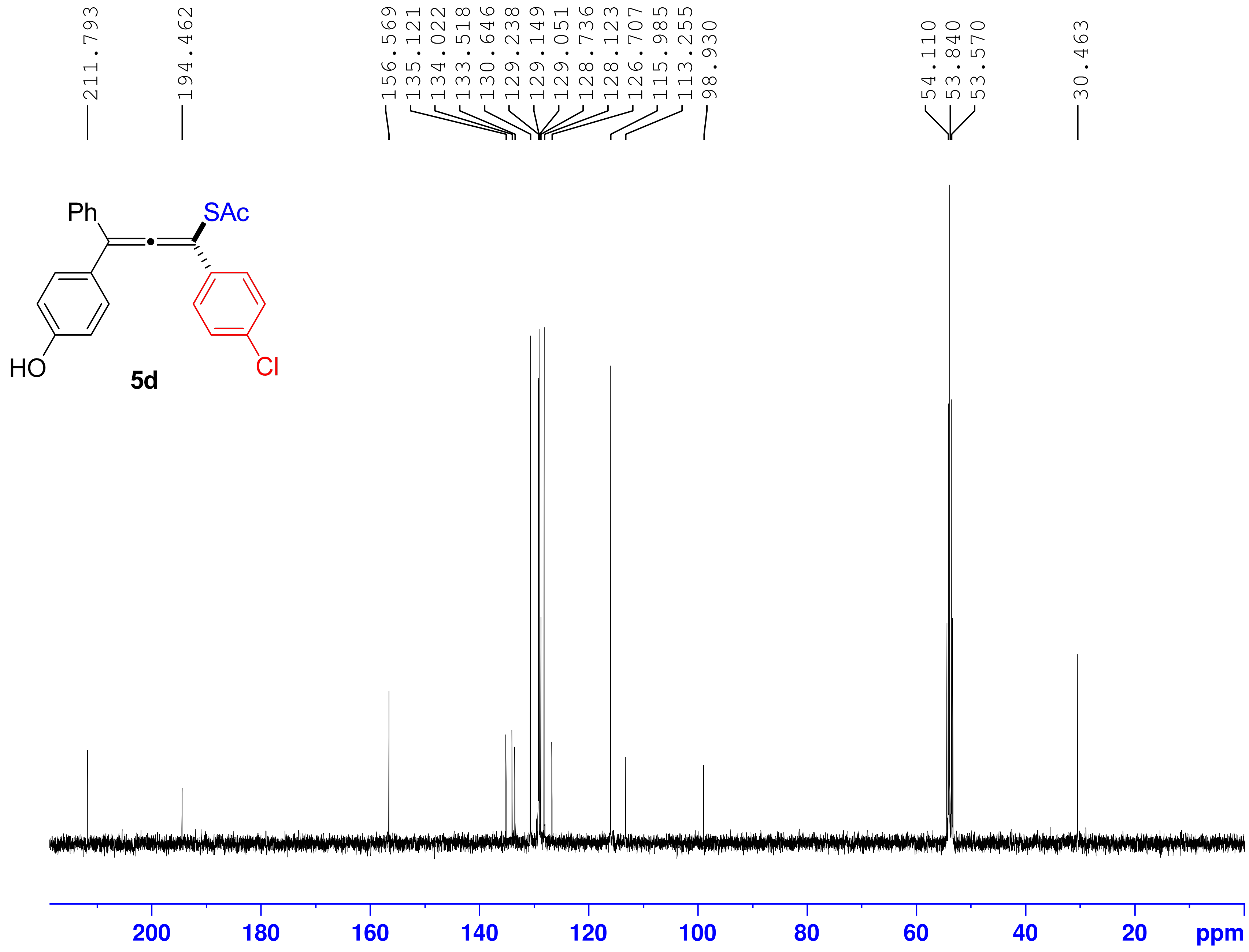

Current Data Parameters
NAME qdy-20135-2 C
EXPNO 2
PROCNO 1

F2 - Acquisition Parameters
Date_ 20160504
Time 22.19
INSTRUM spect
PROBHD 5 mm PABBO BB/
PULPROG zgpg30
TD 65536
SOLVENT CD2Cl2
NS 89
DS 0
SWH 24038.461 Hz
FIDRES 0.366798 Hz
AQ 1.3631488 sec
RG 196.92
DW 20.800 usec
DE 6.50 usec
TE 298.3 K
D1 2.00000000 sec
D11 0.03000000 sec
TD0 1

======== CHANNEL f1 ========
SFO1 100.6228298 MHz
NUC1 13C
P1 9.70 usec
PLW1 46.98899841 W

======== CHANNEL f2 ========
SFO2 400.1316005 MHz
NUC2 1H
CPDPRG[2 waltz16
PCPD2 90.00 usec
PLW2 11.99499989 W
PLW12 0.34213999 W
PLW13 0.27713001 W

F2 - Processing parameters
SI 32768
SF 100.6127292 MHz
WDW EM
SSB 0
LB 1.00 Hz
GB 0
PC 1.40

Supplementary Figure 114. ^{13}C NMR spectrum for 5d

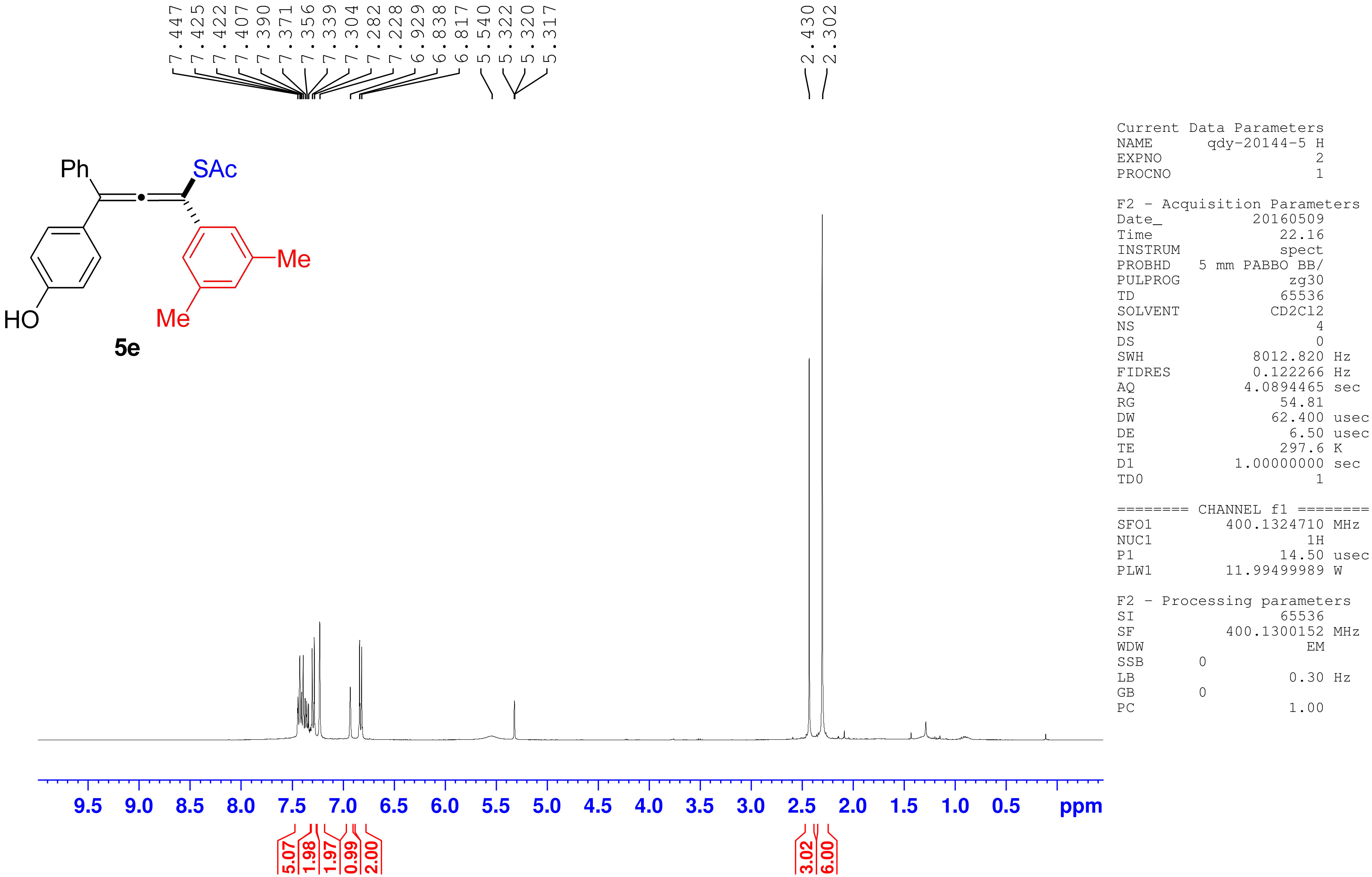

Supplementary Figure 115. ^{1}H NMR spectrum for 5e

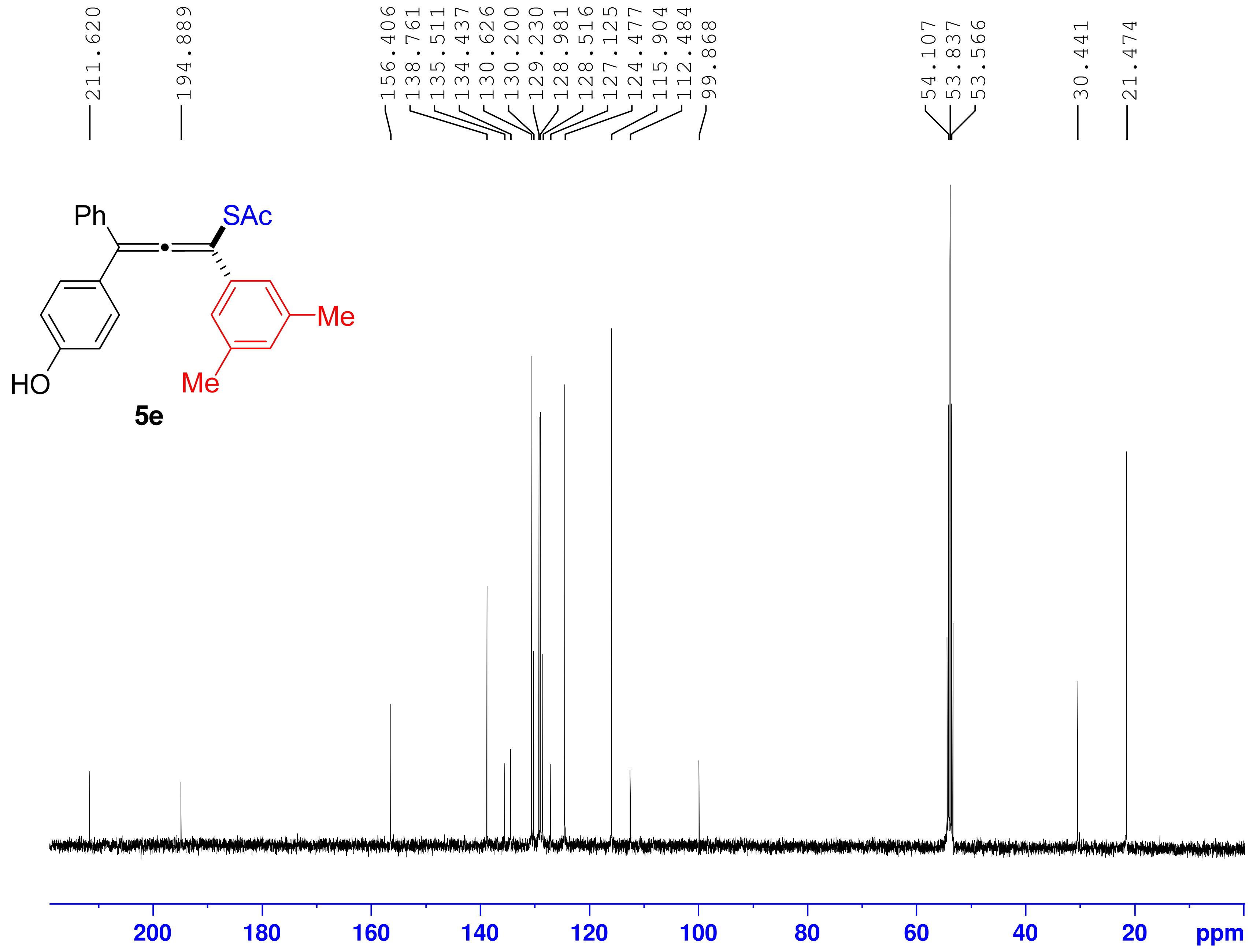

Current Data Parameters
NAME qdy-20144-5 C
EXPNO 1
PROCNO 1

F2 - Acquisition Parameters
Date_ 20160509
Time 20.02
INSTRUM spect
PROBHD 5 mm PABBO BB/
PULPROG zgpg30
TD 65536
SOLVENT CD2Cl2
NS 176
DS 0
SWH 24038.461 Hz
FIDRES 0.366798 Hz
AQ 1.3631488 sec
RG 196.92
DW 20.800 usec
DE 6.50 usec
TE 297.3 K
D1 2.00000000 sec
D11 0.03000000 sec
TD0 1

======== CHANNEL f1 ========
SFO1 100.6228298 MHz
NUC1 13C
P1 9.70 usec
PLW1 46.98899841 W

======== CHANNEL f2 ========
SFO2 400.1316005 MHz
NUC2 1H
CPDPRG[2 waltz16
PCPD2 90.00 usec
PLW2 11.99499989 W
PLW12 0.34213999 W
PLW13 0.27713001 W

F2 - Processing parameters
SI 32768
SF 100.6127292 MHz
WDW EM
SSB 0
LB 1.00 Hz
GB 0
PC 1.40

Supplementary Figure 116. ^{13}C NMR spectrum for 5e

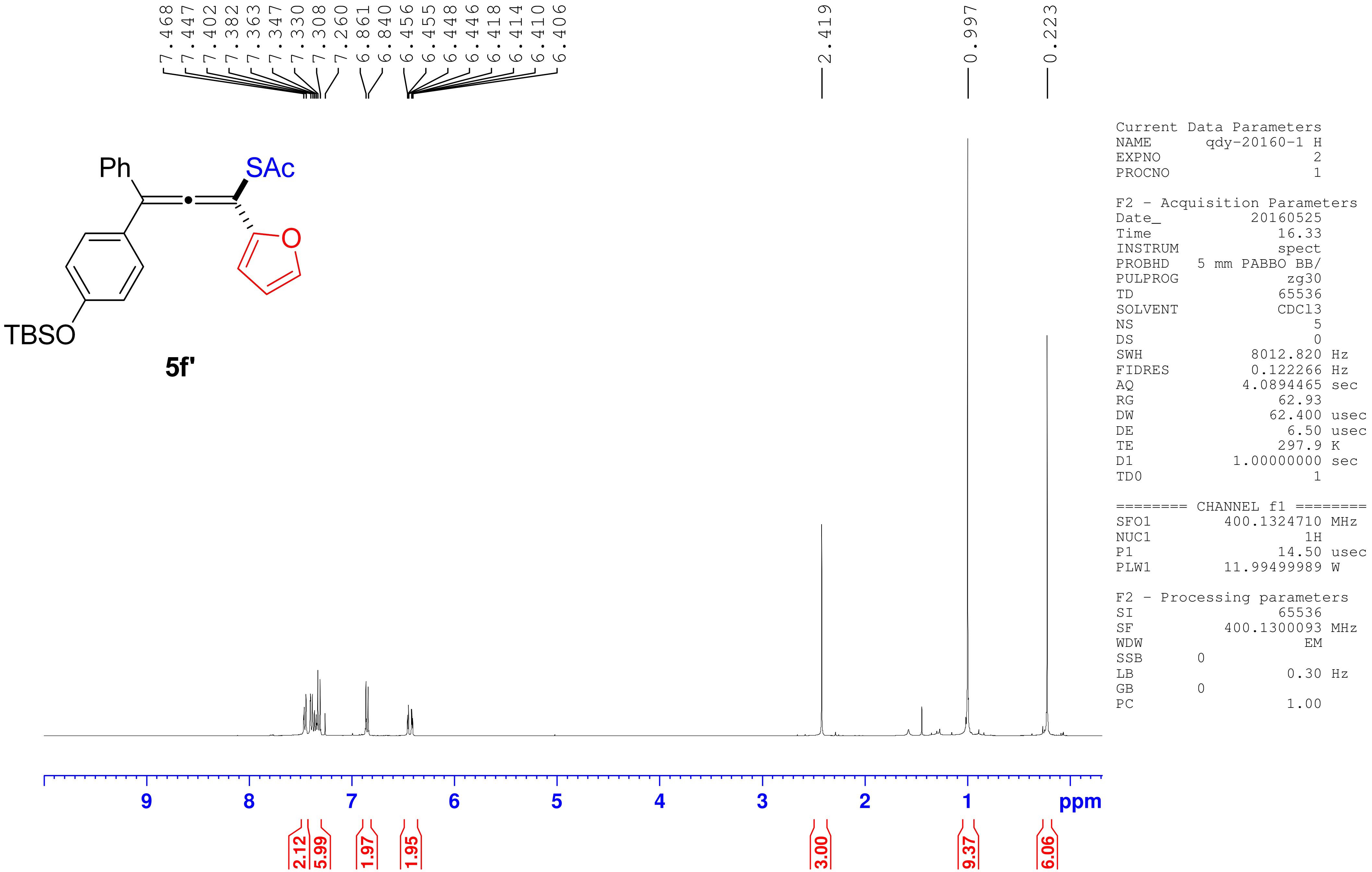

Supplementary Figure 117. ^{1}H NMR spectrum for 5f'

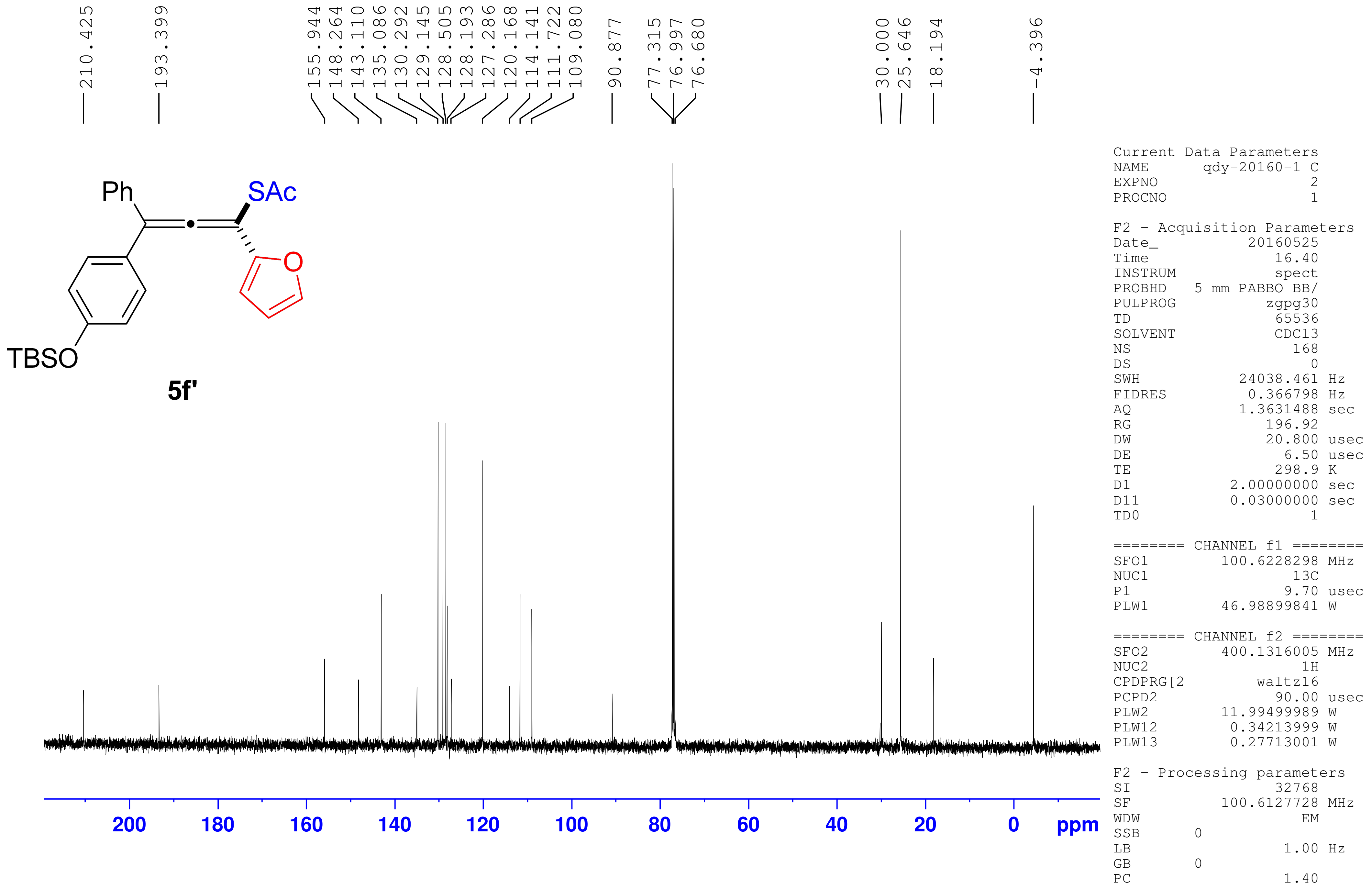

Supplementary Figure 118. ^{13}C NMR spectrum for 5f'

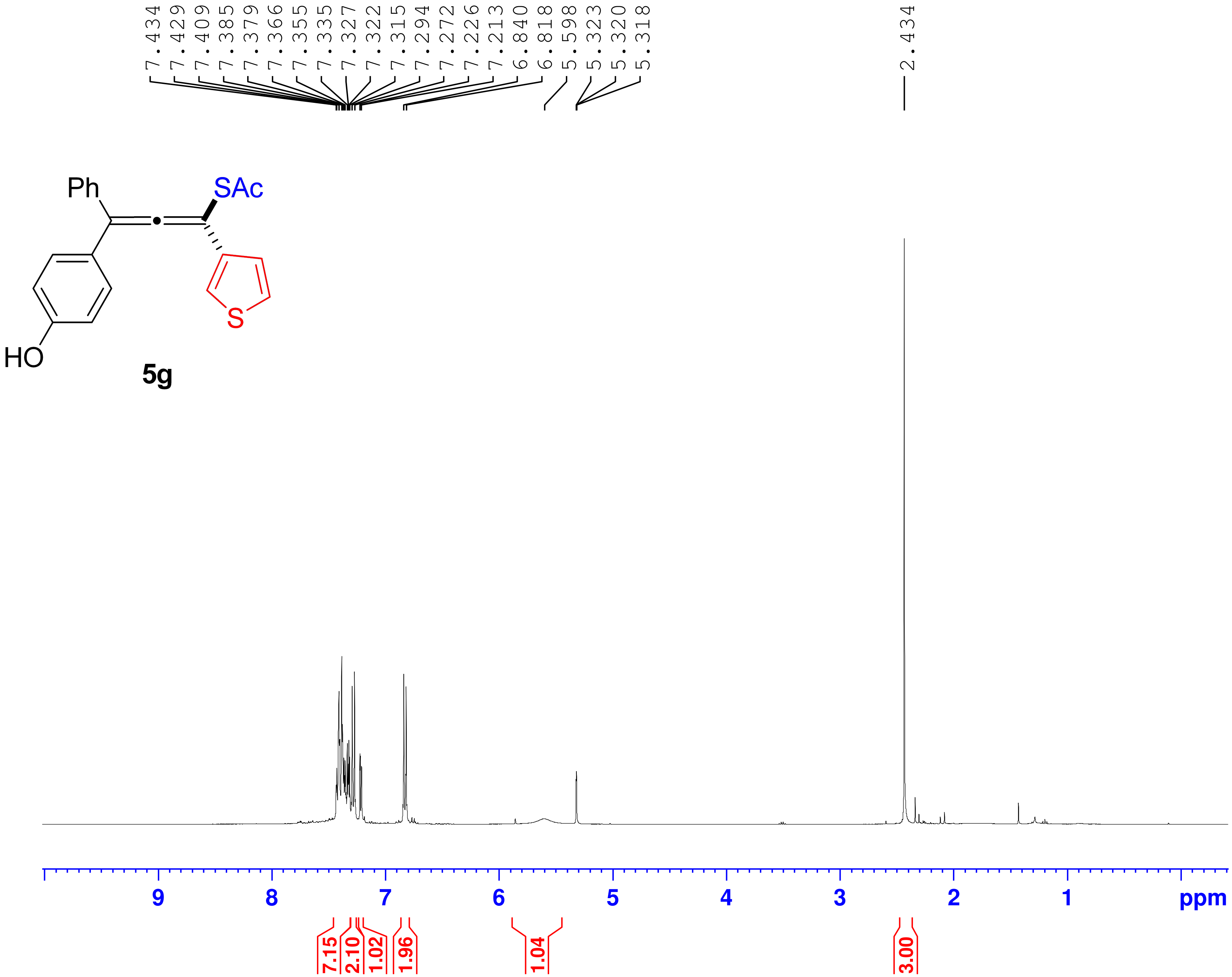

Current Data Parameters
NAME qdy-20115-1 H
EXPNO 4
PROCNO 1

F2 - Acquisition Parameters
Date_ 20160416
Time 19.26
INSTRUM spect
PROBHD 5 mm PABBO BB/
PULPROG zg30
TD 65536
SOLVENT CD2Cl2
NS 8
DS 0
SWH 8012.820 Hz
FIDRES 0.122266 Hz
AQ 4.0894465 sec
RG 82.92
DW 62.400 usec
DE 6.50 usec
TE 298.5 K
D1 1.00000000 sec
TD0 1

======== CHANNEL f1 ========
SFO1 400.1324710 MHz
NUC1 1H
P1 14.50 usec
PLW1 11.99499989 W

F2 - Processing parameters
SI 65536
SF 400.1300152 MHz
WDW EM
SSB 0
LB 0.30 Hz
GB 0
PC 1.00

Supplementary Figure 119. ^{1}H NMR spectrum for 5g

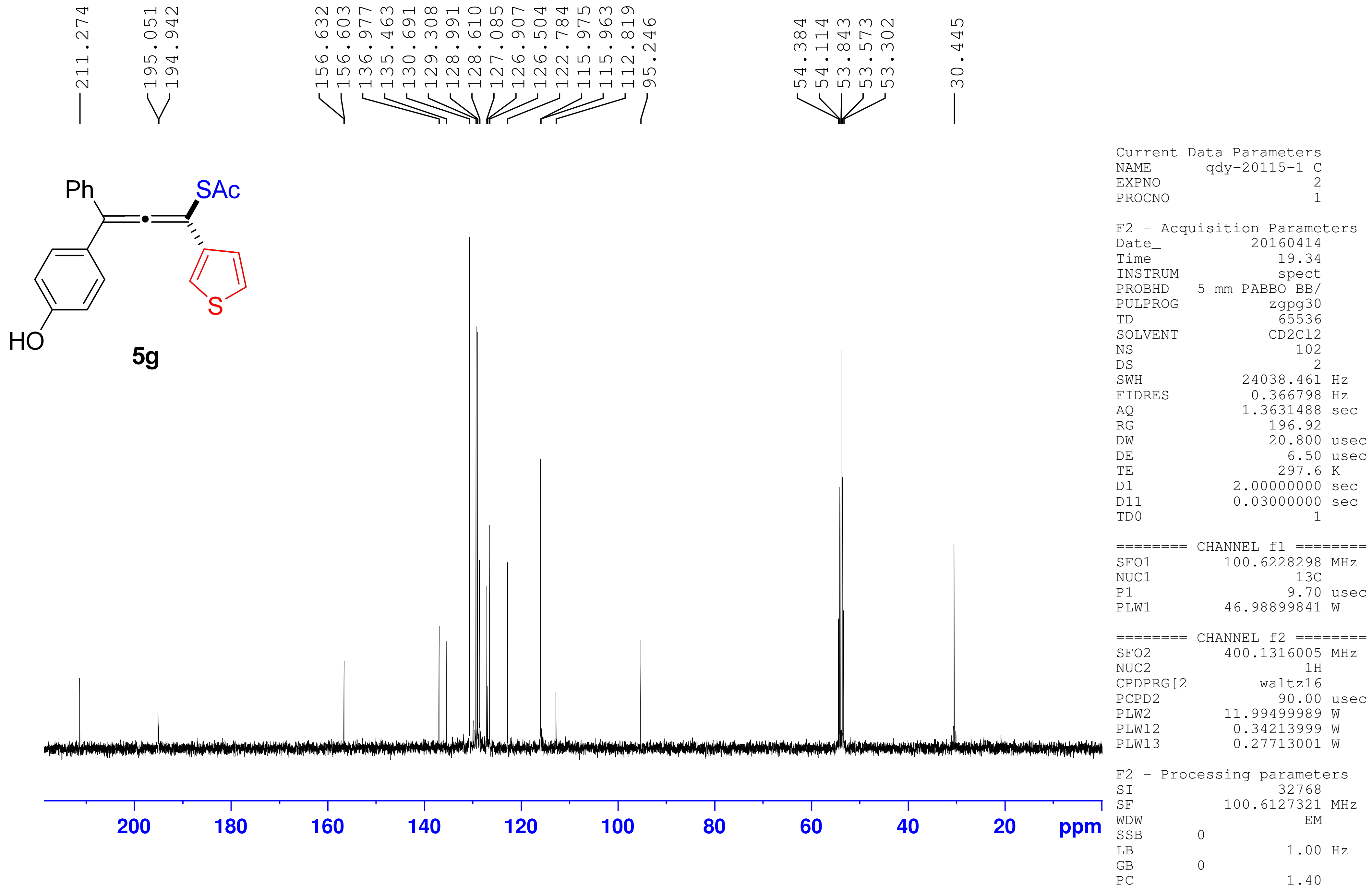

Supplementary Figure 120. ^{13}C NMR spectrum for 5g

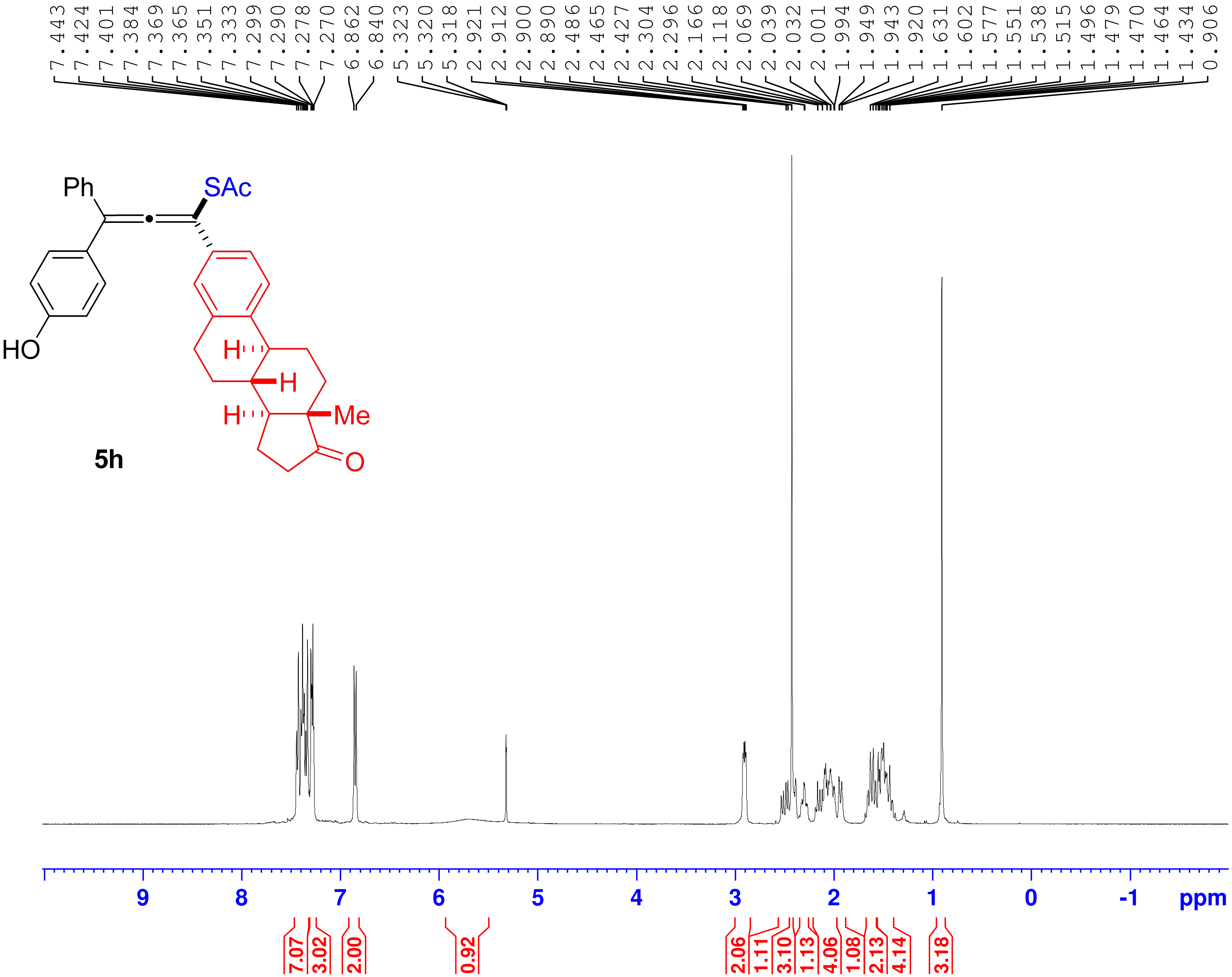

Current Data Parameters
NAME qdy-20117-3 H
EXPNO 1
PROCNO 1

F2 - Acquisition Parameters
Date_ 20160421
Time 19.07
INSTRUM spect
PROBHD 5 mm PABBO BB/
PULPROG zg30
TD 65536
SOLVENT CD2Cl2
NS 6
DS 0
SWH 8012.820 Hz
FIDRES 0.122266 Hz
AQ 4.0894465 sec
RG 54.81
DW 62.400 usec
DE 6.50 usec
TE 297.2 K
D1 1.00000000 sec
TD0 1

======== CHANNEL f1 ========
SFO1 400.1324710 MHz
NUC1 1H
P1 14.50 usec
PLW1 11.99499989 W

F2 - Processing parameters
SI 65536
SF 400.1300151 MHz
WDW EM
SSB 0
LB 0.30 Hz
GB 0
PC 1.00

Supplementary Figure 121. ¹H NMR spectrum for 5h

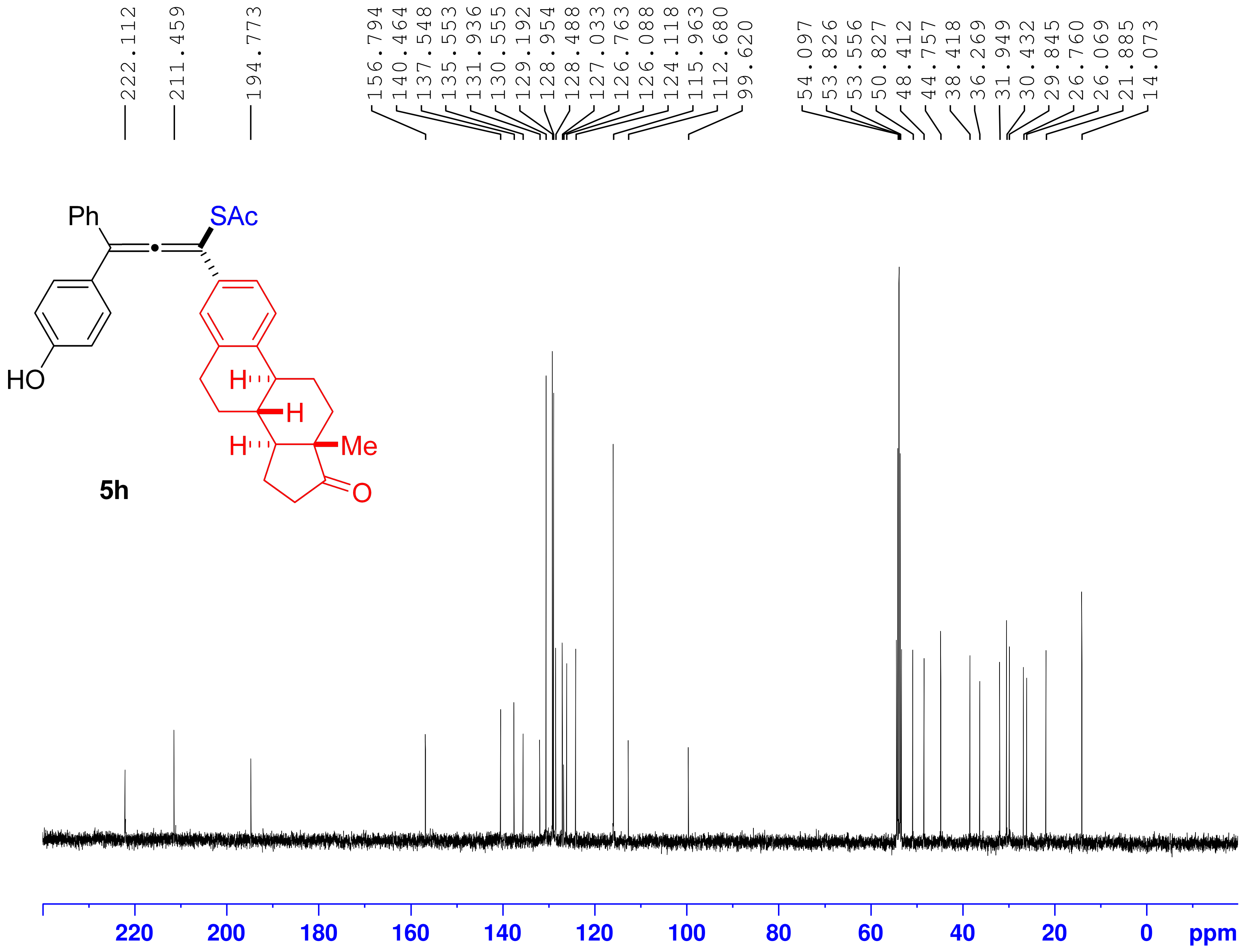


```
Current Data Parameters
NAME        qdy-20117-3 C
EXPNO                   1
PROCNO                  1

F2 - Acquisition Parameters
Date_            20160420
Time                19.42
INSTRUM             spect
PROBHD    5 mm PABBO BB/
PULPROG           zgpg30
TD                  65536
SOLVENT            CD2Cl2
NS                    194
DS                      2
SWH             29761.904 Hz
FIDRES           0.454131 Hz
AQ              1.1010048 sec
RG                 196.92
DW                 16.800 usec
DE                   6.50 usec
TE                  297.6 K
D1             2.00000000 sec
D11            0.03000000 sec
TD0                     1

======== CHANNEL f1 ========
SFO1          100.6228303 MHz
NUC1                  13C
P1                   9.70 usec
PLW1          46.98899841 W

======== CHANNEL f2 ========
SFO2          400.1316005 MHz
NUC2                   1H
CPDPRG[2          waltz16
PCPD2               90.00 usec
PLW2          11.99499989 W
PLW12          0.34213999 W
PLW13          0.27713001 W

F2 - Processing parameters
SI                  32768
SF            100.6127317 MHz
WDW                    EM
SSB      0
LB                   1.00 Hz
GB       0
PC                   1.40
```

Supplementary Figure 122. ^{13}C NMR spectrum for 5h

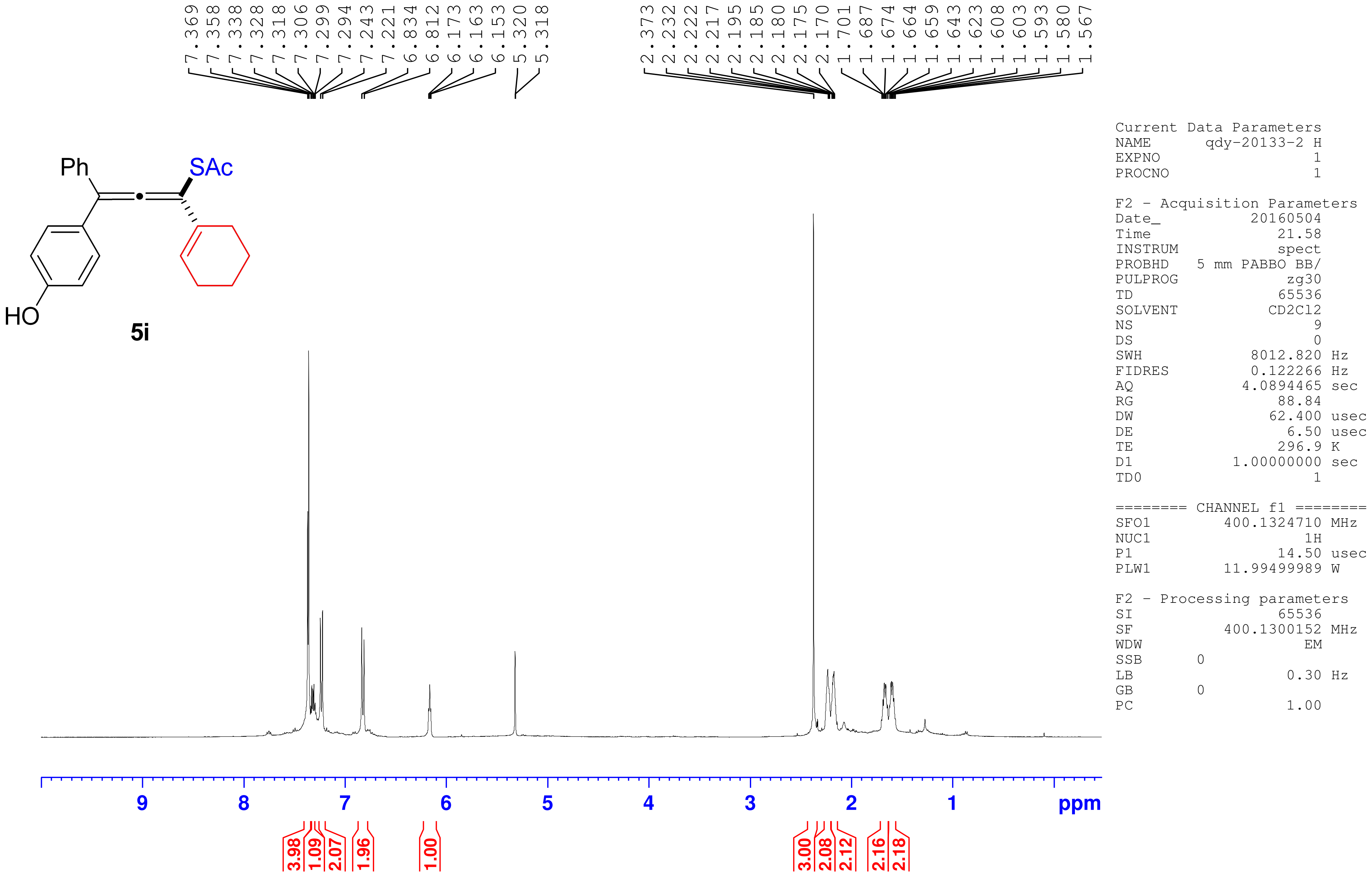

Supplementary Figure 123. ^{1}H NMR spectrum for 5i

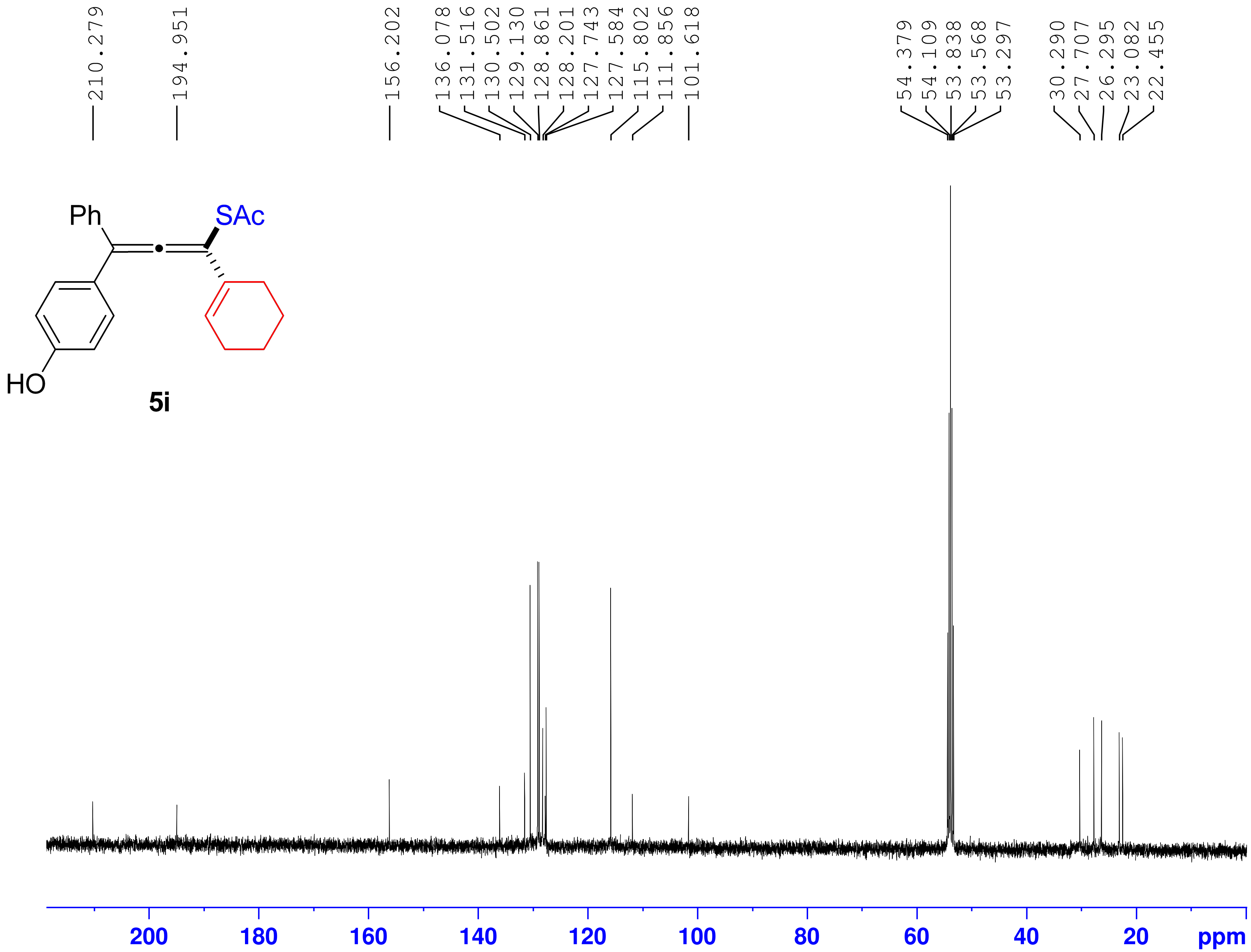

Current Data Parameters
NAME qdy-20133-2 C
EXPNO 2
PROCNO 1

F2 - Acquisition Parameters
Date_ 20160504
Time 22.04
INSTRUM spect
PROBHD 5 mm PABBO BB/
PULPROG zgpg30
TD 65536
SOLVENT CD2Cl2
NS 279
DS 0
SWH 24038.461 Hz
FIDRES 0.366798 Hz
AQ 1.3631488 sec
RG 196.92
DW 20.800 usec
DE 6.50 usec
TE 298.1 K
D1 2.00000000 sec
D11 0.03000000 sec
TD0 1

======== CHANNEL f1 ========
SFO1 100.6228298 MHz
NUC1 13C
P1 9.70 usec
PLW1 46.98899841 W

======== CHANNEL f2 ========
SFO2 400.1316005 MHz
NUC2 1H
CPDPRG[2 waltz16
PCPD2 90.00 usec
PLW2 11.99499989 W
PLW12 0.34213999 W
PLW13 0.27713001 W

F2 - Processing parameters
SI 32768
SF 100.6127277 MHz
WDW EM
SSB 0
LB 1.00 Hz
GB 0
PC 1.40

Supplementary Figure 124. ^{13}C NMR spectrum for 5i

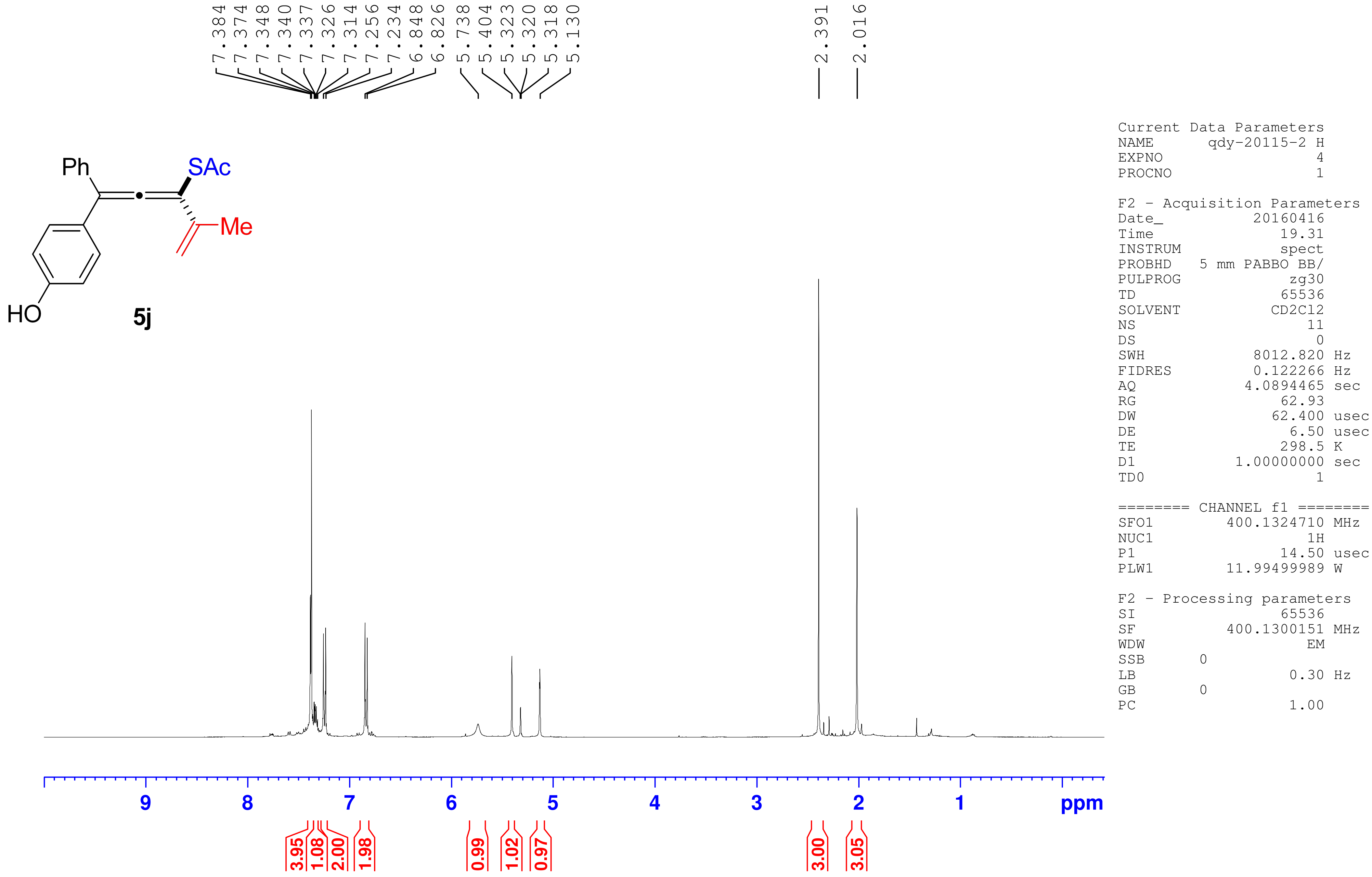

Supplementary Figure 125. ^{1}H NMR spectrum for 5j

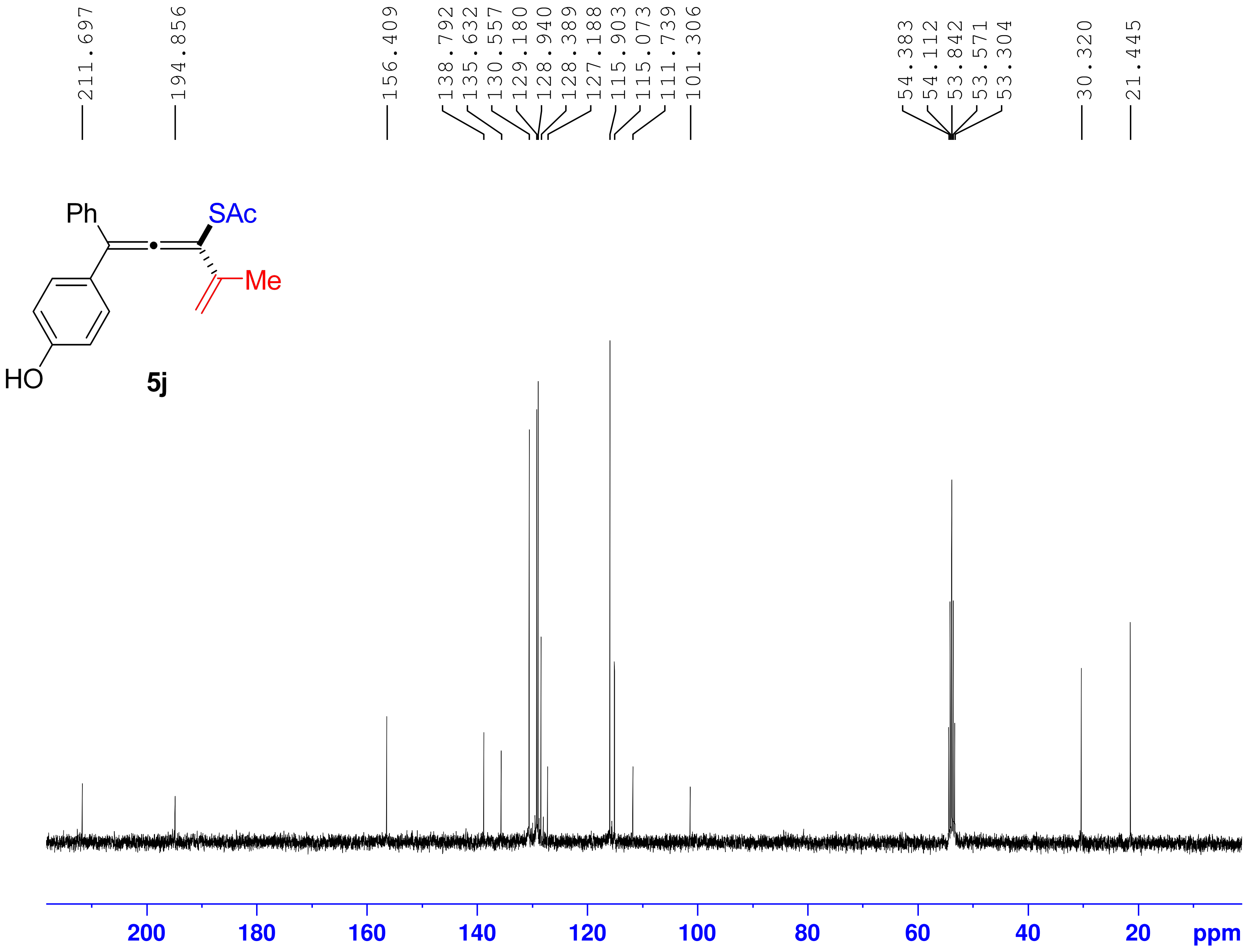


```
Current Data Parameters
NAME        qdy-20115-2 C
EXPNO                   1
PROCNO                  1

F2 - Acquisition Parameters
Date_            20160414
Time                21.58
INSTRUM             spect
PROBHD   5 mm PABBO BB/
PULPROG           zgpg30
TD                 65536
SOLVENT           CD2Cl2
NS                   150
DS                     0
SWH            24038.461 Hz
FIDRES          0.366798 Hz
AQ             1.3631488 sec
RG                196.92
DW                20.800 usec
DE                  6.50 usec
TE                 298.6 K
D1            2.00000000 sec
D11           0.03000000 sec
TD0                    1

======== CHANNEL f1 ========
SFO1         100.6228298 MHz
NUC1                 13C
P1                  9.70 usec
PLW1         46.98899841 W

======== CHANNEL f2 ========
SFO2         400.1316005 MHz
NUC2                  1H
CPDPRG[2         waltz16
PCPD2              90.00 usec
PLW2         11.99499989 W
PLW12         0.34213999 W
PLW13         0.27713001 W

F2 - Processing parameters
SI                 32768
SF           100.6127284 MHz
WDW                   EM
SSB      0
LB                  1.00 Hz
GB       0
PC                  1.40
```

Supplementary Figure 126. ^{13}C NMR spectrum for 5j

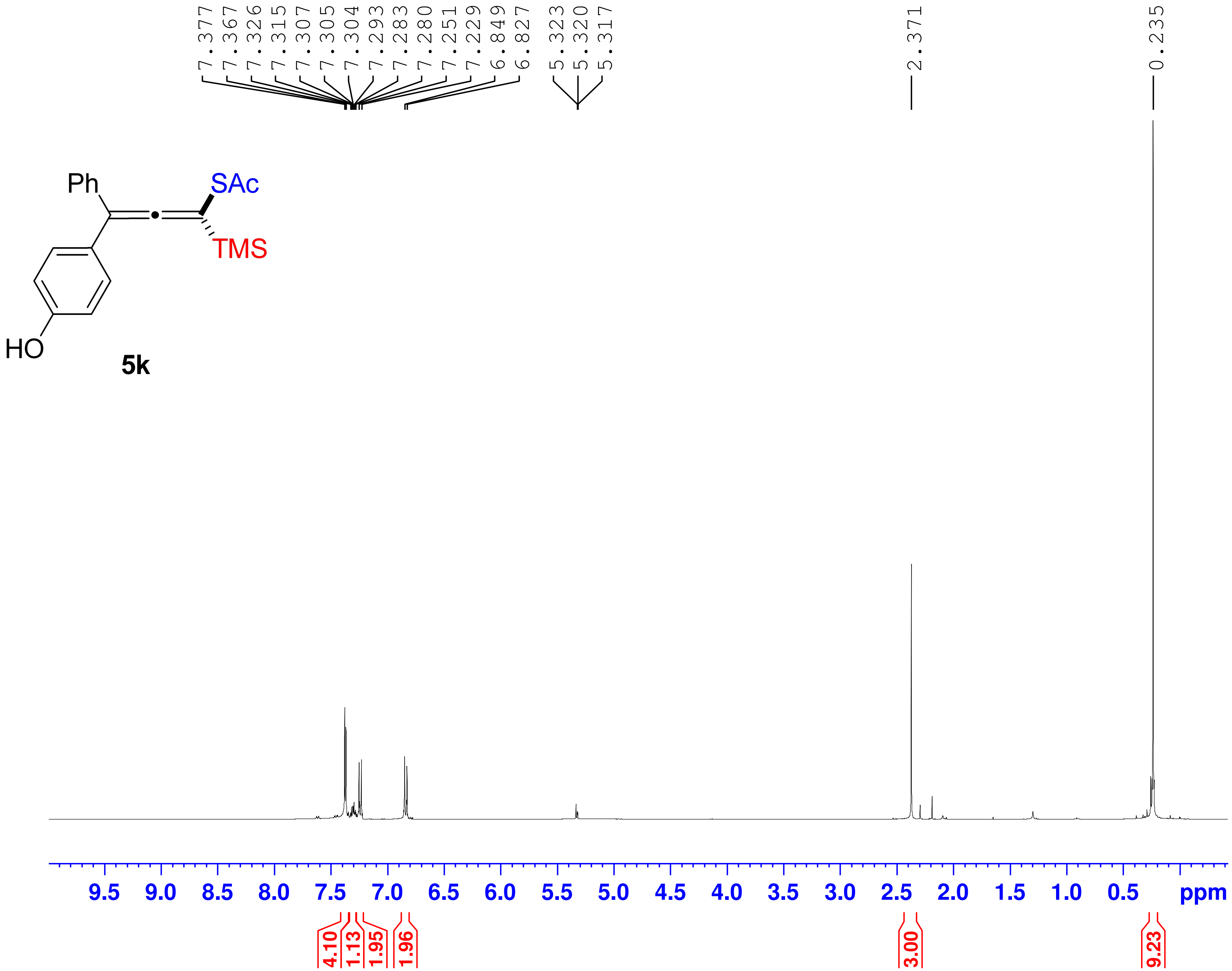


```
Current Data Parameters
NAME       qdy-20099-5 H
EXPNO                  1
PROCNO                 1

F2 - Acquisition Parameters
Date_           20160401
Time               16.57
INSTRUM            spect
PROBHD    5 mm PABBO BB/
PULPROG             zg30
TD                 65536
SOLVENT           CD2Cl2
NS                     7
DS                     0
SWH             8012.820 Hz
FIDRES          0.122266 Hz
AQ             4.0894465 sec
RG                 31.55
DW                62.400 usec
DE                  6.50 usec
TE                 297.0 K
D1            1.00000000 sec
TD0                    1

======== CHANNEL f1 ========
SFO1         400.1324710 MHz
NUC1                  1H
P1                 14.50 usec
PLW1         11.99499989 W

F2 - Processing parameters
SI                 65536
SF           400.1300152 MHz
WDW                   EM
SSB       0
LB                  0.30 Hz
GB        0
PC                  1.00
```

Supplementary Figure 127. ¹H NMR spectrum for 5k

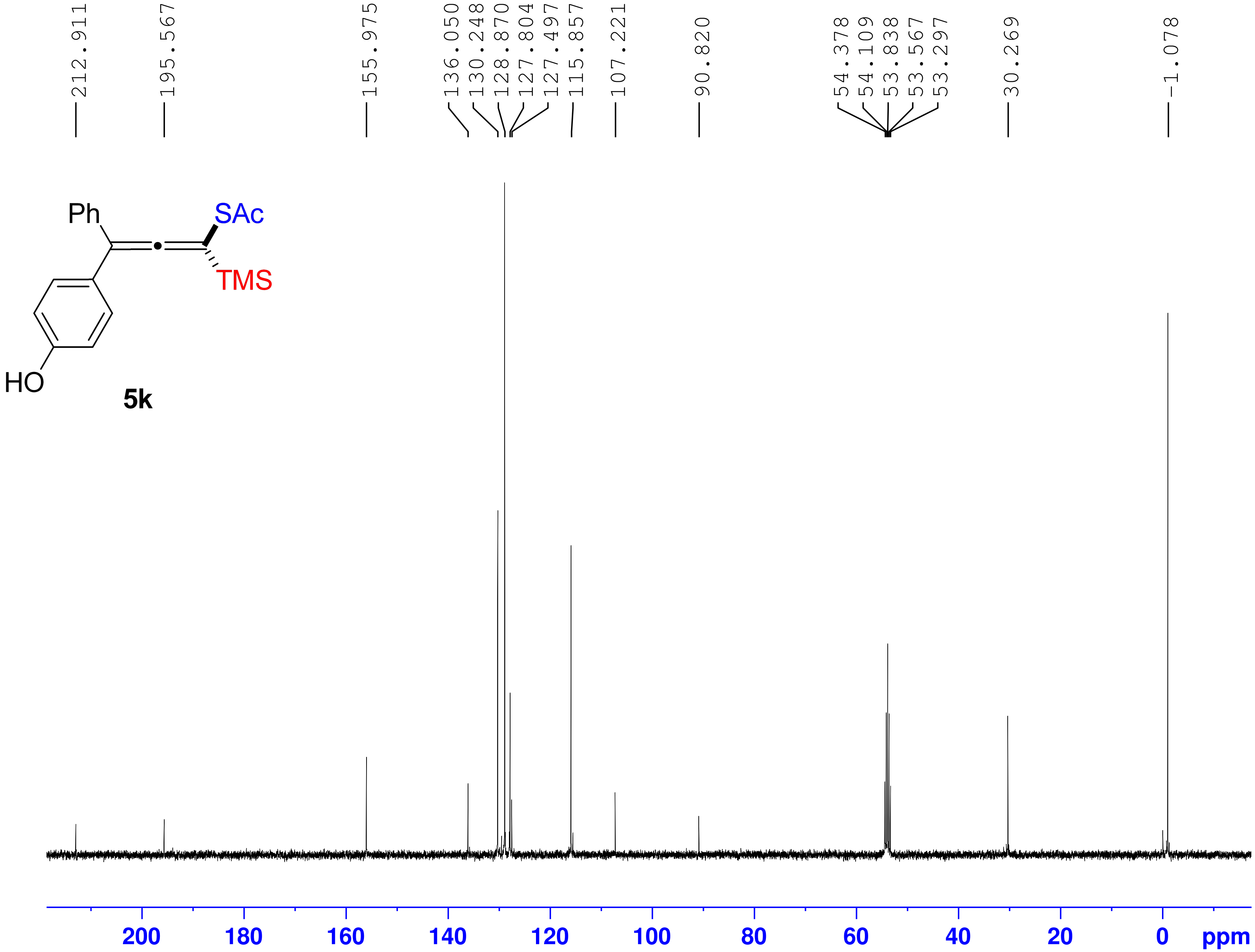

Current Data Parameters
NAME qdy-20099-5 C
EXPNO 1
PROCNO 1

F2 - Acquisition Parameters
Date_ 20160401
Time 17.01
INSTRUM spect
PROBHD 5 mm PABBO BB/
PULPROG zgpg30
TD 65536
SOLVENT CD2Cl2
NS 66
DS 0
SWH 24038.461 Hz
FIDRES 0.366798 Hz
AQ 1.3631488 sec
RG 196.92
DW 20.800 usec
DE 6.50 usec
TE 298.0 K
D1 2.00000000 sec
D11 0.03000000 sec
TD0 1

======== CHANNEL f1 ========
SFO1 100.6228298 MHz
NUC1 13C
P1 9.70 usec
PLW1 46.98899841 W

======== CHANNEL f2 ========
SFO2 400.1316005 MHz
NUC2 1H
CPDPRG[2 waltz16
PCPD2 90.00 usec
PLW2 11.99499989 W
PLW12 0.34213999 W
PLW13 0.27713001 W

F2 - Processing parameters
SI 32768
SF 100.6127291 MHz
WDW EM
SSB 0
LB 1.00 Hz
GB 0
PC 1.40

Supplementary Figure 128. ^{13}C NMR spectrum for 5k

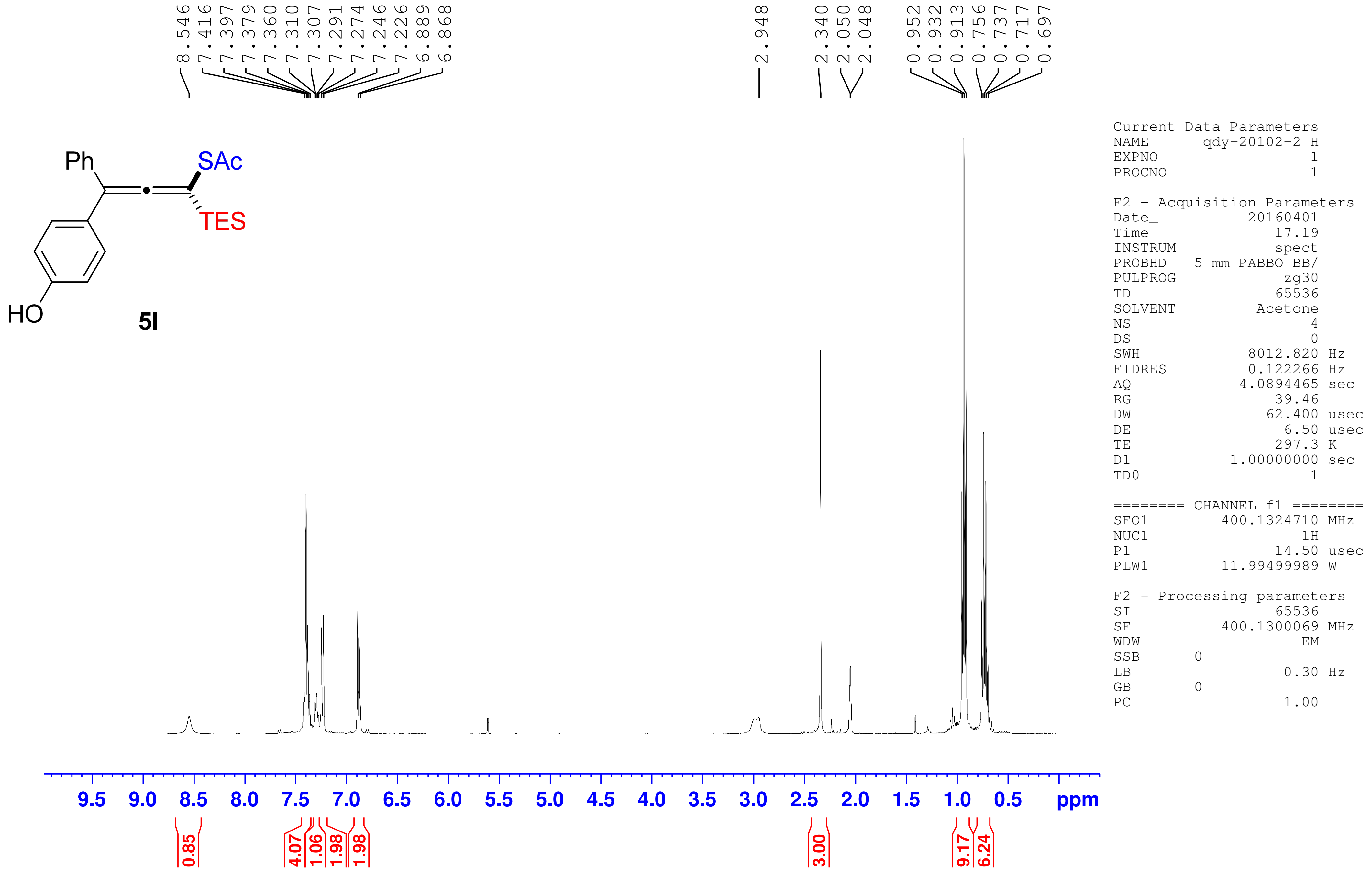

Supplementary Figure 129. ^{1}H NMR spectrum for 5l

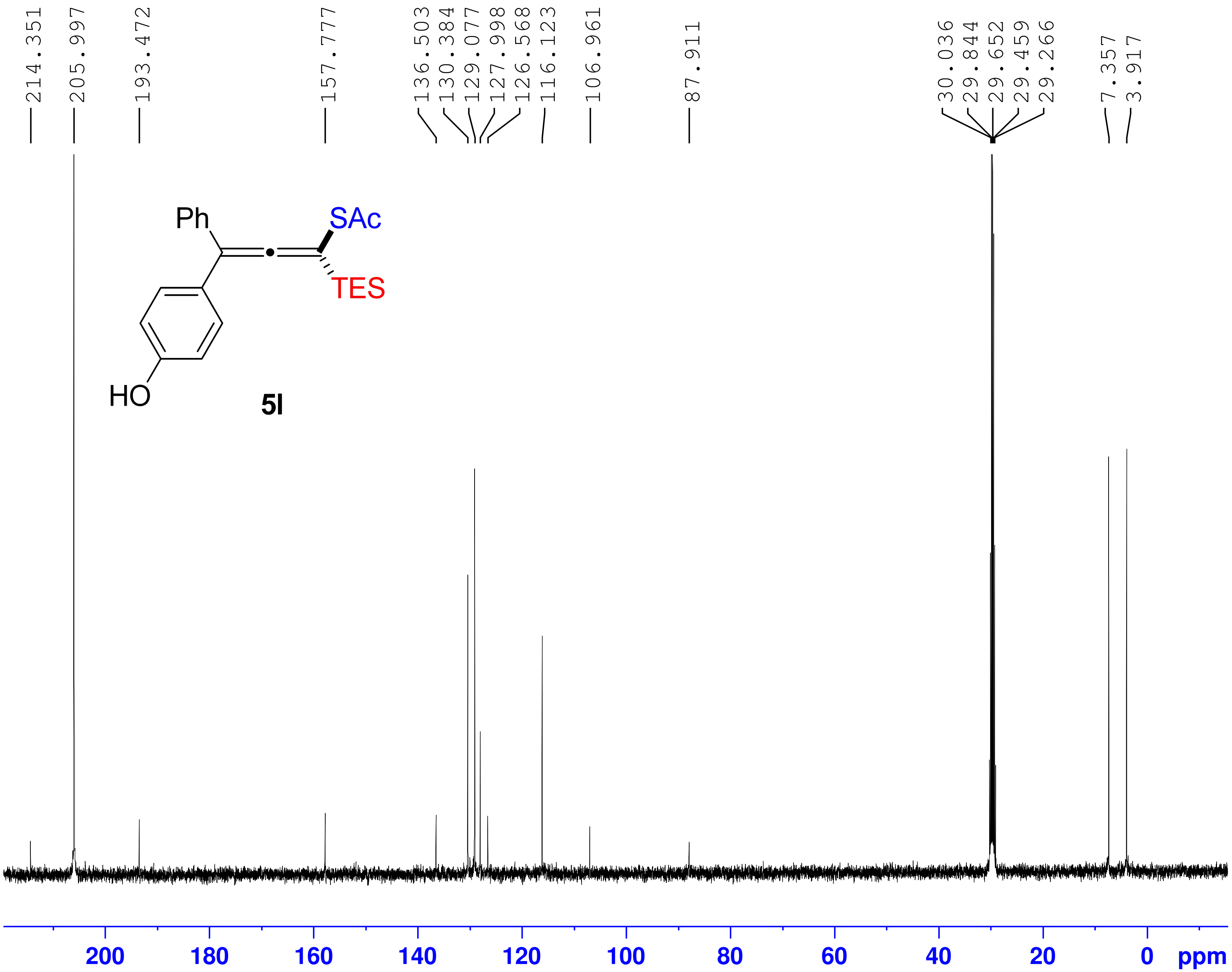

Current Data Parameters		
NAME	qdy-20102-2 C	
EXPNO	3	
PROCNO	1	
F2 - Acquisition Parameters		
Date_	20160402	
Time	14.01	
INSTRUM	spect	
PROBHD	5 mm PABBO BB/	
PULPROG	zgpg30	
TD	65536	
SOLVENT	Acetone	
NS	150	
DS	0	
SWH	24038.461	Hz
FIDRES	0.366798	Hz
AQ	1.3631488	sec
RG	196.92	
DW	20.800	usec
DE	6.50	usec
TE	298.4	K
D1	2.00000000	sec
D11	0.03000000	sec
TD0	1	
======== CHANNEL f1 ========		
SFO1	100.6228298	MHz
NUC1	13C	
P1	9.70	usec
PLW1	46.98899841	W
======== CHANNEL f2 ========		
SFO2	400.1316005	MHz
NUC2	1H	
CPDPRG[2	waltz16	
PCPD2	90.00	usec
PLW2	11.99499989	W
PLW12	0.34213999	W
PLW13	0.27713001	W
F2 - Processing parameters		
SI	32768	
SF	100.6127007	MHz
WDW	EM	
SSB	0	
LB	1.00	Hz
GB	0	
PC	1.40	

Supplementary Figure 130. ^{13}C NMR spectrum for 5l

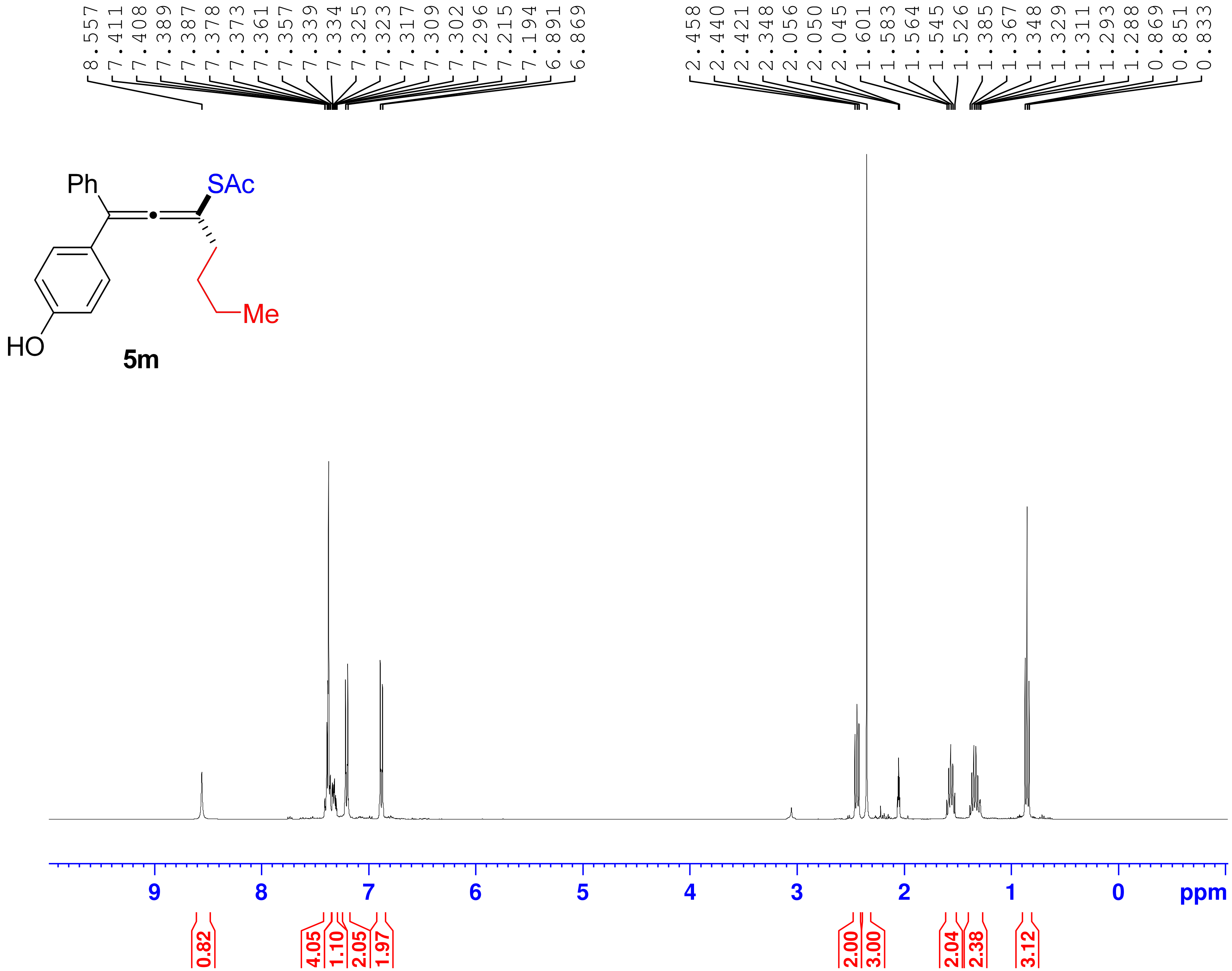


```
Current Data Parameters
NAME       qdy-20099-3 H
EXPNO                  3
PROCNO                 1

F2 - Acquisition Parameters
Date_           20160331
Time               21.45
INSTRUM            spect
PROBHD    5 mm PABBO BB/
PULPROG             zg30
TD                 65536
SOLVENT          Acetone
NS                     6
DS                     0
SWH             8012.820 Hz
FIDRES          0.122266 Hz
AQ             4.0894465 sec
RG                 34.77
DW                62.400 usec
DE                  6.50 usec
TE                 297.5 K
D1            1.00000000 sec
TD0                    1

======== CHANNEL f1 ========
SFO1         400.1324710 MHz
NUC1                  1H
P1                 14.50 usec
PLW1         11.99499989 W

F2 - Processing parameters
SI                 65536
SF           400.1300067 MHz
WDW                   EM
SSB       0
LB                  0.30 Hz
GB        0
PC                  1.00
```

Supplementary Figure 131. ^{1}H NMR spectrum for 5m

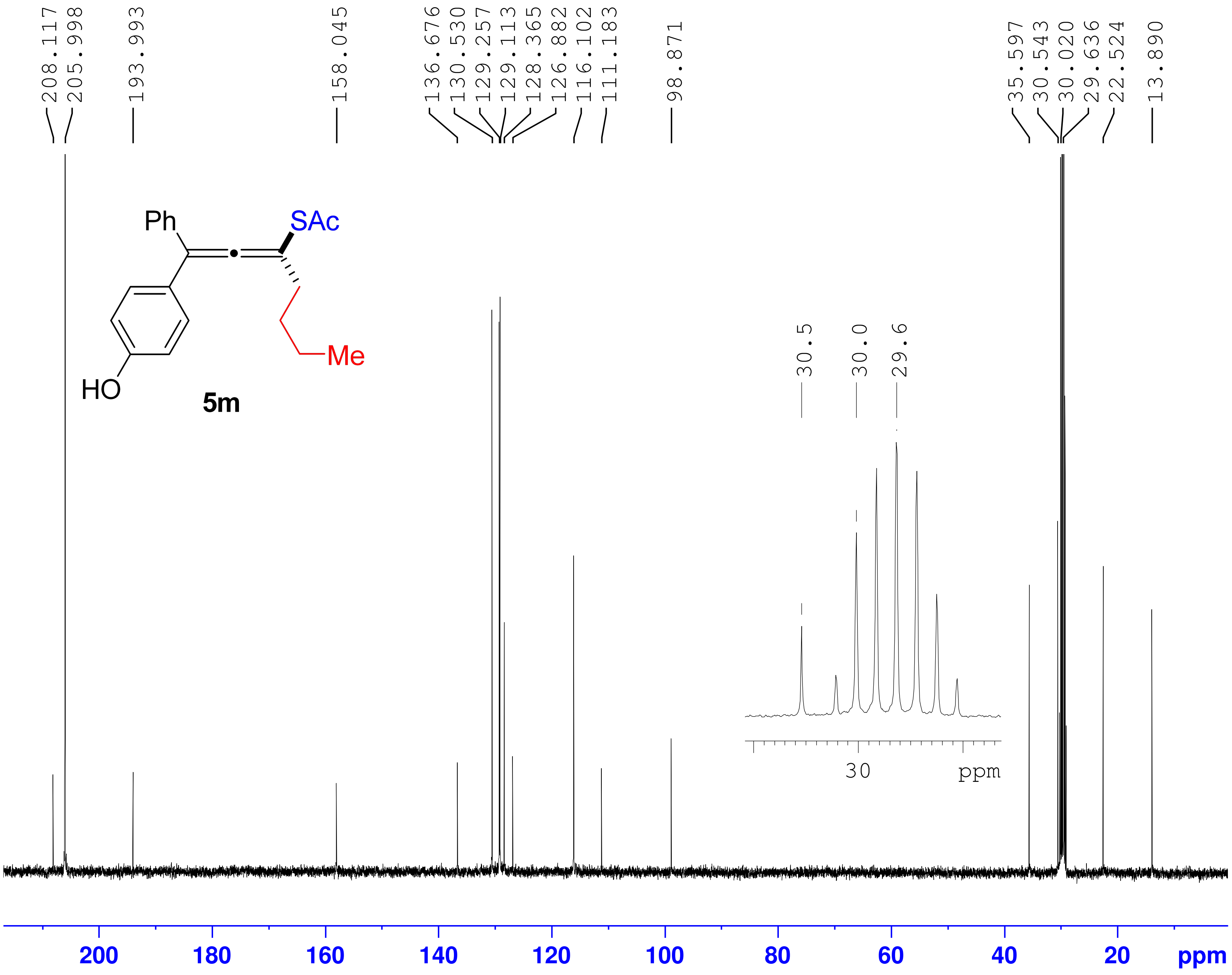

Current Data Parameters
NAME qdy-20099-3 C
EXPNO 3
PROCNO 1

F2 - Acquisition Parameters
Date_ 20160331
Time 21.50
INSTRUM spect
PROBHD 5 mm PABBO BB/
PULPROG zgpg30
TD 65536
SOLVENT Acetone
NS 99
DS 0
SWH 24038.461 Hz
FIDRES 0.366798 Hz
AQ 1.3631488 sec
RG 196.92
DW 20.800 usec
DE 6.50 usec
TE 298.4 K
D1 2.00000000 sec
D11 0.03000000 sec
TD0 1

======== CHANNEL f1 ========
SFO1 100.6228298 MHz
NUC1 13C
P1 9.70 usec
PLW1 46.98899841 W

======== CHANNEL f2 ========
SFO2 400.1316005 MHz
NUC2 1H
CPDPRG[2 waltz16
PCPD2 90.00 usec
PLW2 11.99499989 W
PLW12 0.34213999 W
PLW13 0.27713001 W

F2 - Processing parameters
SI 32768
SF 100.6127044 MHz
WDW EM
SSB 0
LB 1.00 Hz
GB 0
PC 1.40

Supplementary Figure 132. ^{13}C NMR spectrum for 5m

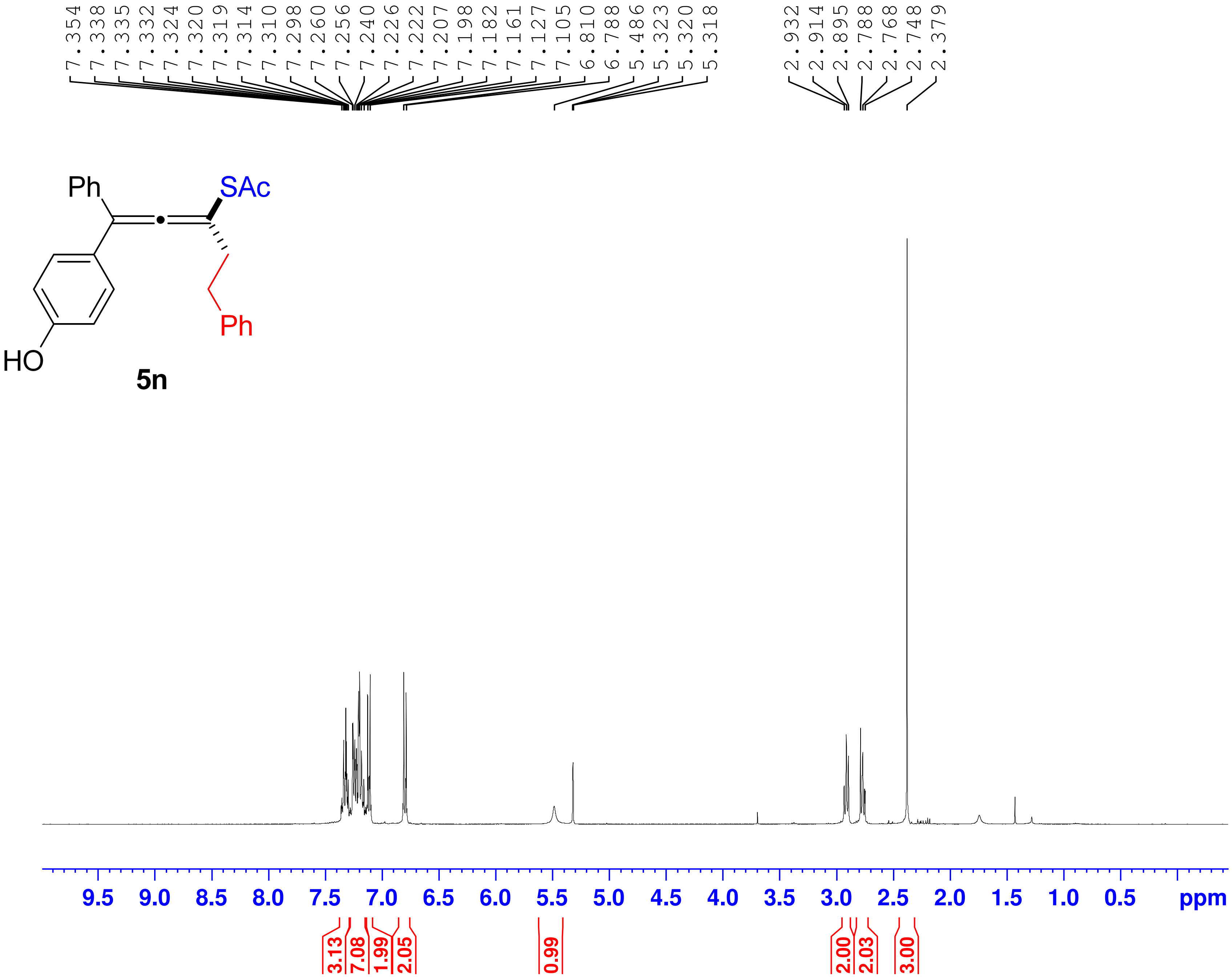

Current Data Parameters
NAME qdy-20118-2 H
EXPNO 2
PROCNO 1

F2 - Acquisition Parameters
Date_ 20160418
Time 21.40
INSTRUM spect
PROBHD 5 mm PABBO BB/
PULPROG zg30
TD 65536
SOLVENT CD2Cl2
NS 4
DS 0
SWH 8012.820 Hz
FIDRES 0.122266 Hz
AQ 4.0894465 sec
RG 82.92
DW 62.400 usec
DE 6.50 usec
TE 296.9 K
D1 1.00000000 sec
TD0 1

======== CHANNEL f1 ========
SFO1 400.1324710 MHz
NUC1 1H
P1 14.50 usec
PLW1 11.99499989 W

F2 - Processing parameters
SI 65536
SF 400.1300152 MHz
WDW EM
SSB 0
LB 0.30 Hz
GB 0
PC 1.00

Supplementary Figure 133. ^{1}H NMR spectrum for 5n

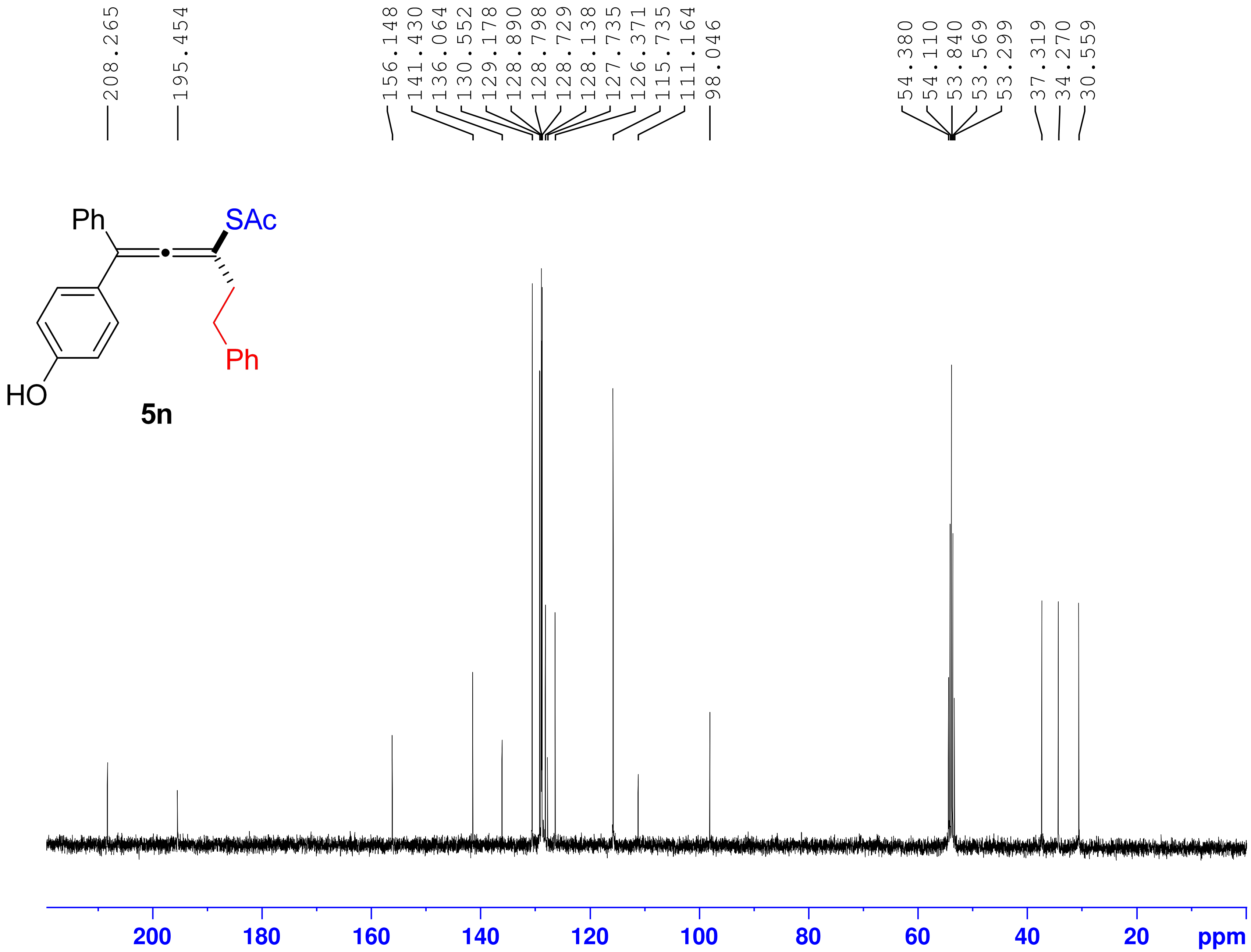


```
Current Data Parameters
NAME        qdy-20118-2 C
EXPNO                   1
PROCNO                  1

F2 - Acquisition Parameters
Date_            20160418
Time                19.30
INSTRUM             spect
PROBHD     5 mm PABBO BB/
PULPROG           zgpg30
TD                 65536
SOLVENT           CD2Cl2
NS                   151
DS                     0
SWH            24038.461 Hz
FIDRES          0.366798 Hz
AQ             1.3631488 sec
RG                196.92
DW                20.800 usec
DE                  6.50 usec
TE                 297.4 K
D1            2.00000000 sec
D11           0.03000000 sec
TD0                    1

======== CHANNEL f1 ========
SFO1         100.6228298 MHz
NUC1                 13C
P1                  9.70 usec
PLW1         46.98899841 W

======== CHANNEL f2 ========
SFO2         400.1316005 MHz
NUC2                  1H
CPDPRG[2          waltz16
PCPD2              90.00 usec
PLW2         11.99499989 W
PLW12         0.34213999 W
PLW13         0.27713001 W

F2 - Processing parameters
SI                 32768
SF           100.6127300 MHz
WDW                   EM
SSB      0
LB                  1.00 Hz
GB       0
PC                  1.40
```

Supplementary Figure 134. ^{13}C NMR spectrum for 5n

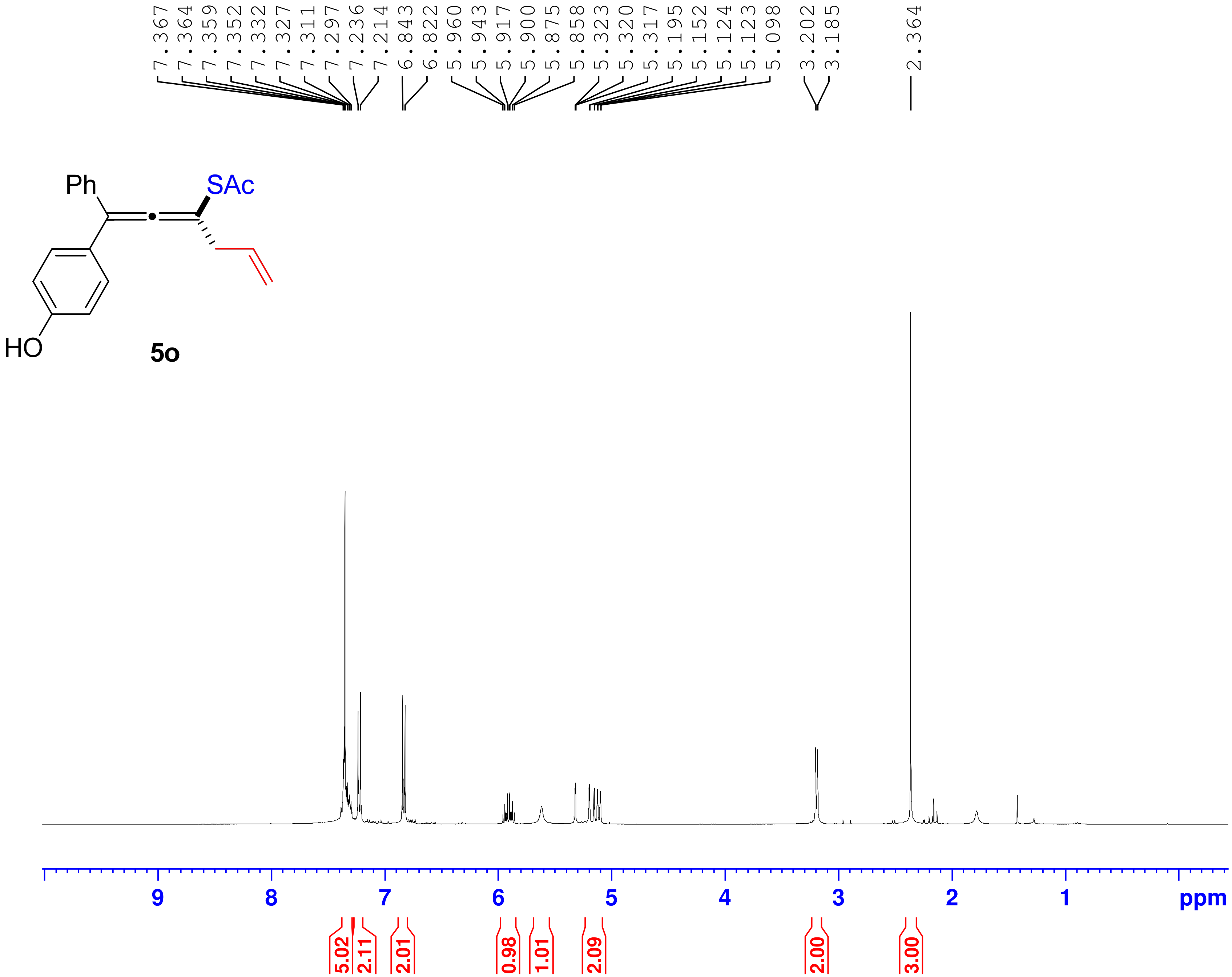

Current Data Parameters
NAME qdy-20118-1 H
EXPNO 1
PROCNO 1

F2 - Acquisition Parameters
Date_ 20160416
Time 19.35
INSTRUM spect
PROBHD 5 mm PABBO BB/
PULPROG zg30
TD 65536
SOLVENT CD2Cl2
NS 7
DS 0
SWH 8012.820 Hz
FIDRES 0.122266 Hz
AQ 4.0894465 sec
RG 70.97
DW 62.400 usec
DE 6.50 usec
TE 298.5 K
D1 1.00000000 sec
TD0 1

======== CHANNEL f1 ========
SFO1 400.1324710 MHz
NUC1 1H
P1 14.50 usec
PLW1 11.99499989 W

F2 - Processing parameters
SI 65536
SF 400.1300153 MHz
WDW EM
SSB 0
LB 0.30 Hz
GB 0
PC 1.00

Supplementary Figure 135. ^{1}H NMR spectrum for 5o

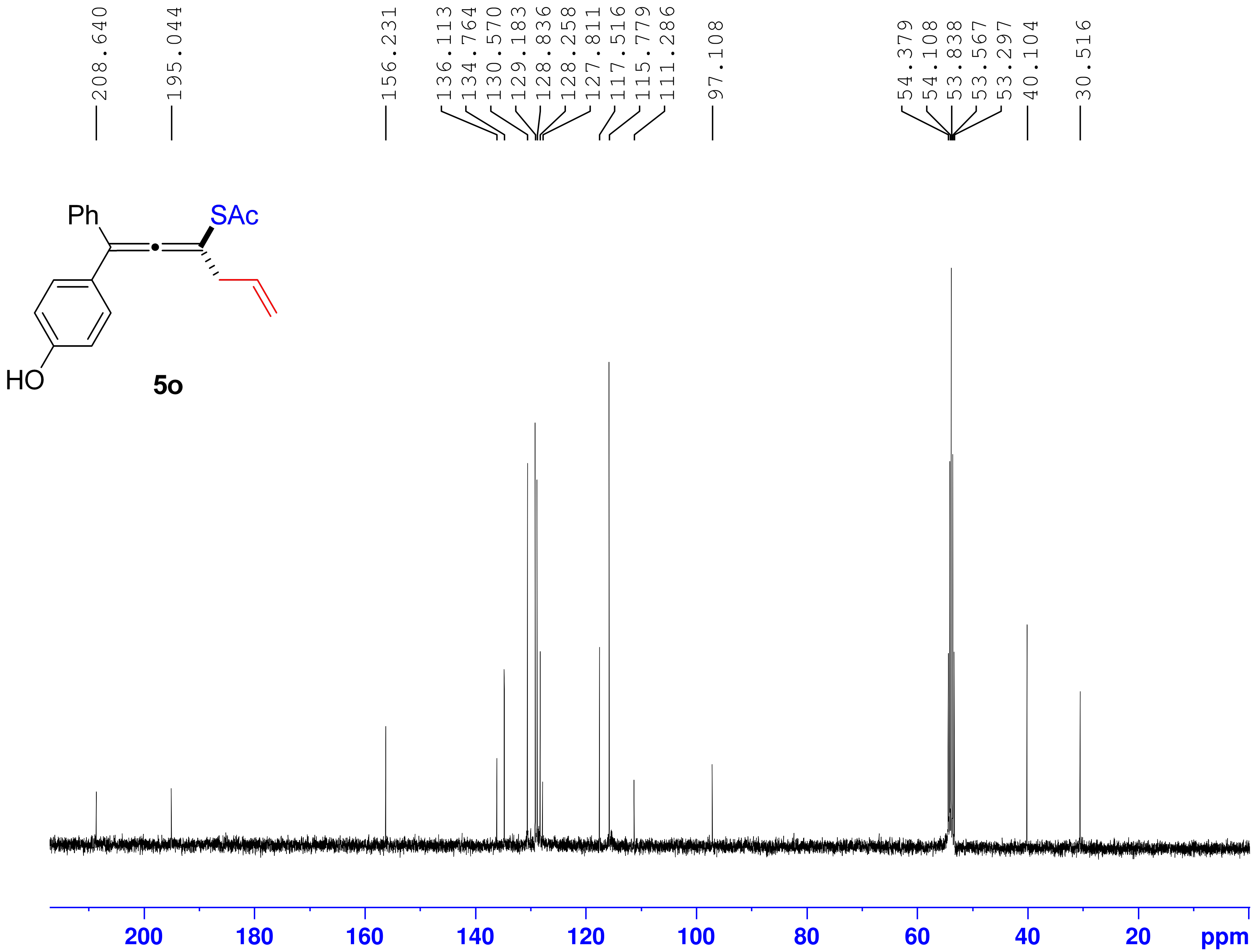

Current Data Parameters	
NAME	qdy-20118-1 C
EXPNO	1
PROCNO	1
F2 - Acquisition Parameters	
Date_	20160416
Time	19.40
INSTRUM	spect
PROBHD	5 mm PABBO BB/
PULPROG	zgpg30
TD	65536
SOLVENT	CD2Cl2
NS	150
DS	0
SWH	24038.461 Hz
FIDRES	0.366798 Hz
AQ	1.3631488 sec
RG	196.92
DW	20.800 usec
DE	6.50 usec
TE	299.4 K
D1	2.00000000 sec
D11	0.03000000 sec
TD0	1
======== CHANNEL f1 ========	
SFO1	100.6228298 MHz
NUC1	13C
P1	9.70 usec
PLW1	46.98899841 W
======== CHANNEL f2 ========	
SFO2	400.1316005 MHz
NUC2	1H
CPDPRG[2	waltz16
PCPD2	90.00 usec
PLW2	11.99499989 W
PLW12	0.34213999 W
PLW13	0.27713001 W
F2 - Processing parameters	
SI	32768
SF	100.6127269 MHz
WDW	EM
SSB	0
LB	1.00 Hz
GB	0
PC	1.40

Supplementary Figure 136. ^{13}C NMR spectrum for 5o

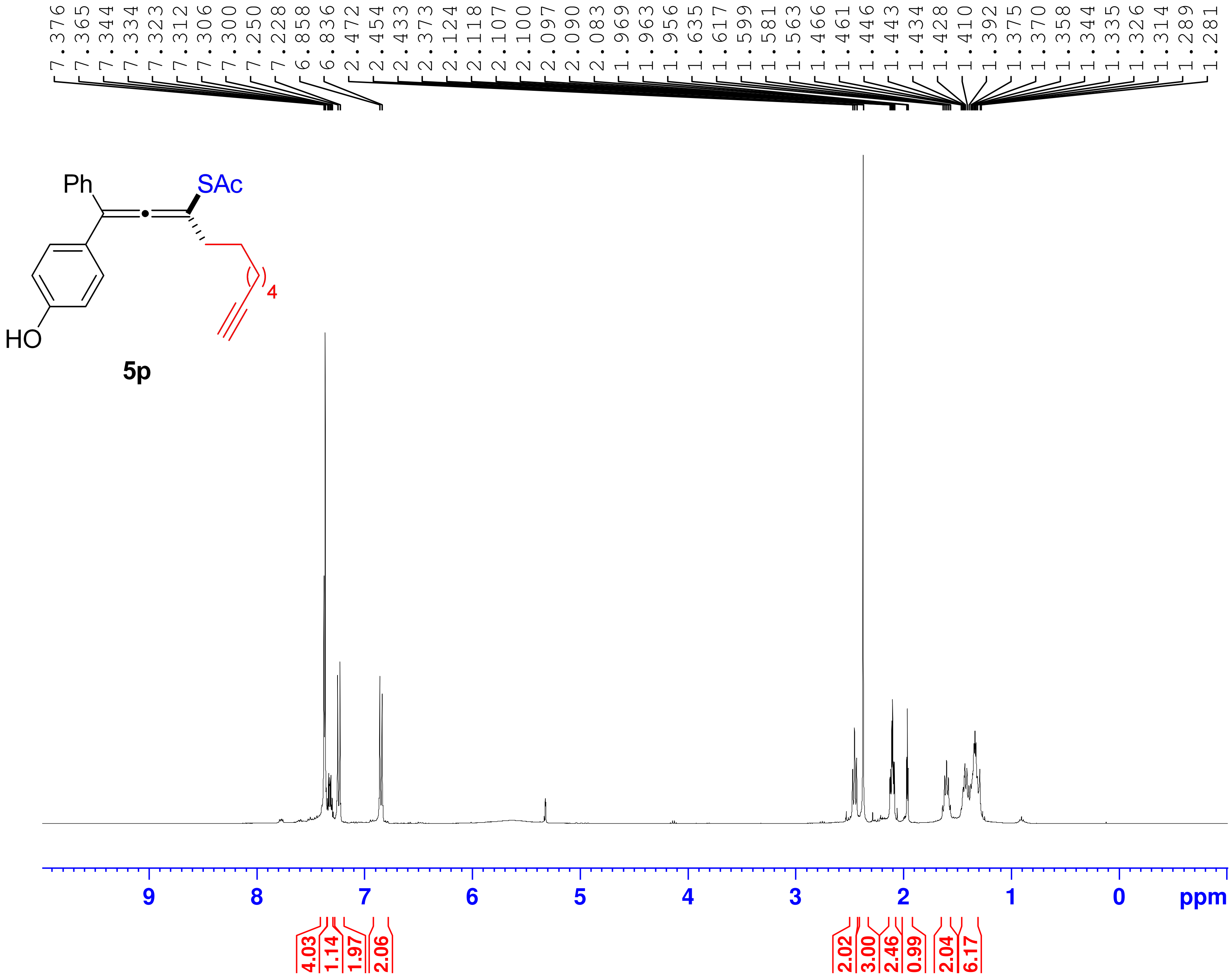


```
Current Data Parameters
NAME       qdy-20099-4 H
EXPNO                  1
PROCNO                 1

F2 - Acquisition Parameters
Date_           20160331
Time               13.27
INSTRUM            spect
PROBHD   5 mm PABBO BB/
PULPROG             zg30
TD                 65536
SOLVENT           CD2Cl2
NS                     6
DS                     0
SWH             8012.820 Hz
FIDRES          0.122266 Hz
AQ             4.0894465 sec
RG                 31.55
DW                62.400 usec
DE                  6.50 usec
TE                 297.5 K
D1            1.00000000 sec
TD0                    1

======== CHANNEL f1 ========
SFO1         400.1324710 MHz
NUC1                  1H
P1                 14.50 usec
PLW1         11.99499989 W

F2 - Processing parameters
SI                 65536
SF           400.1300152 MHz
WDW                   EM
SSB      0
LB                  0.30 Hz
GB       0
PC                  1.00
```

Supplementary Figure 137. ^{1}H NMR spectrum for 5p

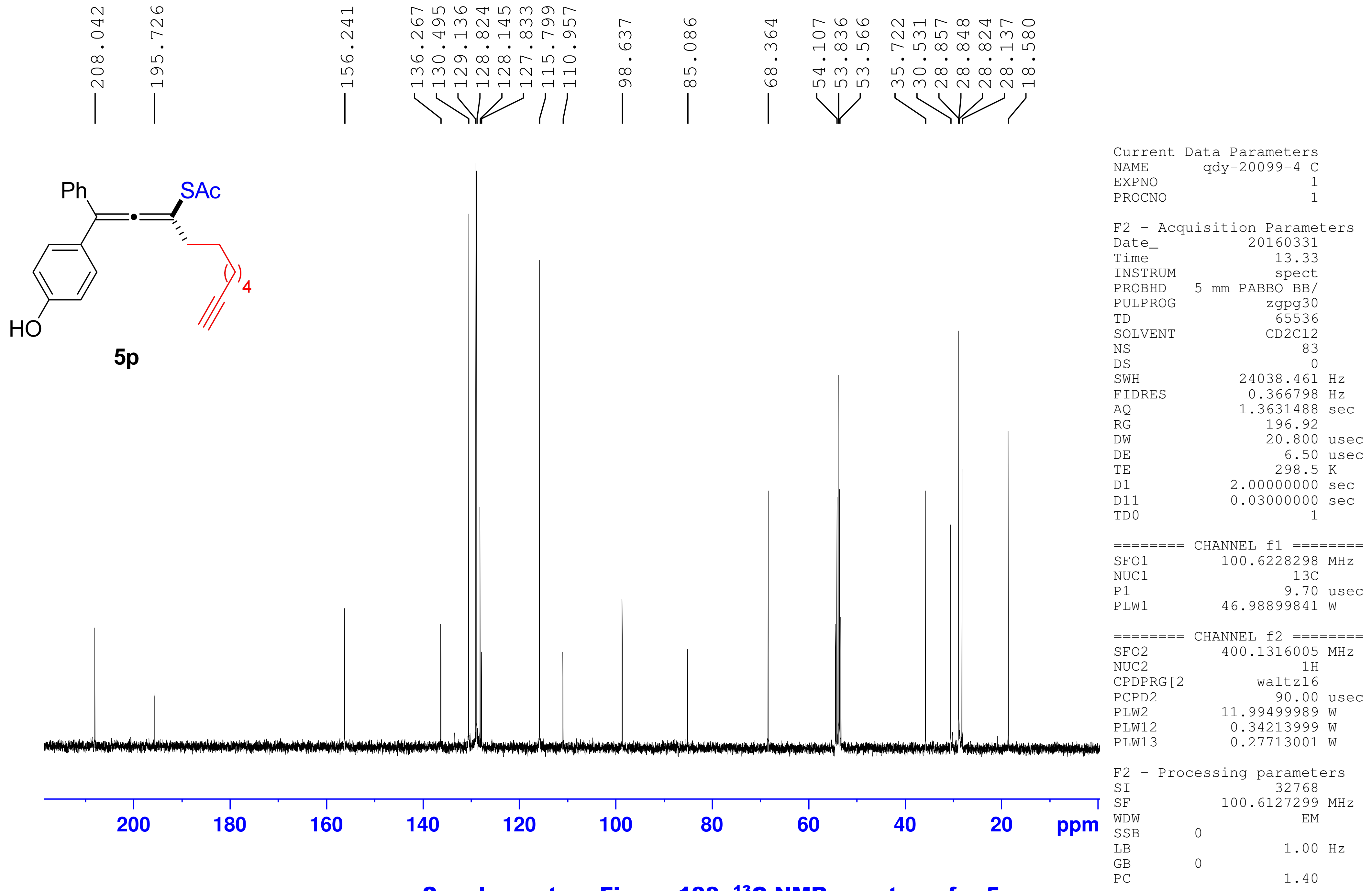

Supplementary Figure 138. ^{13}C NMR spectrum for 5p

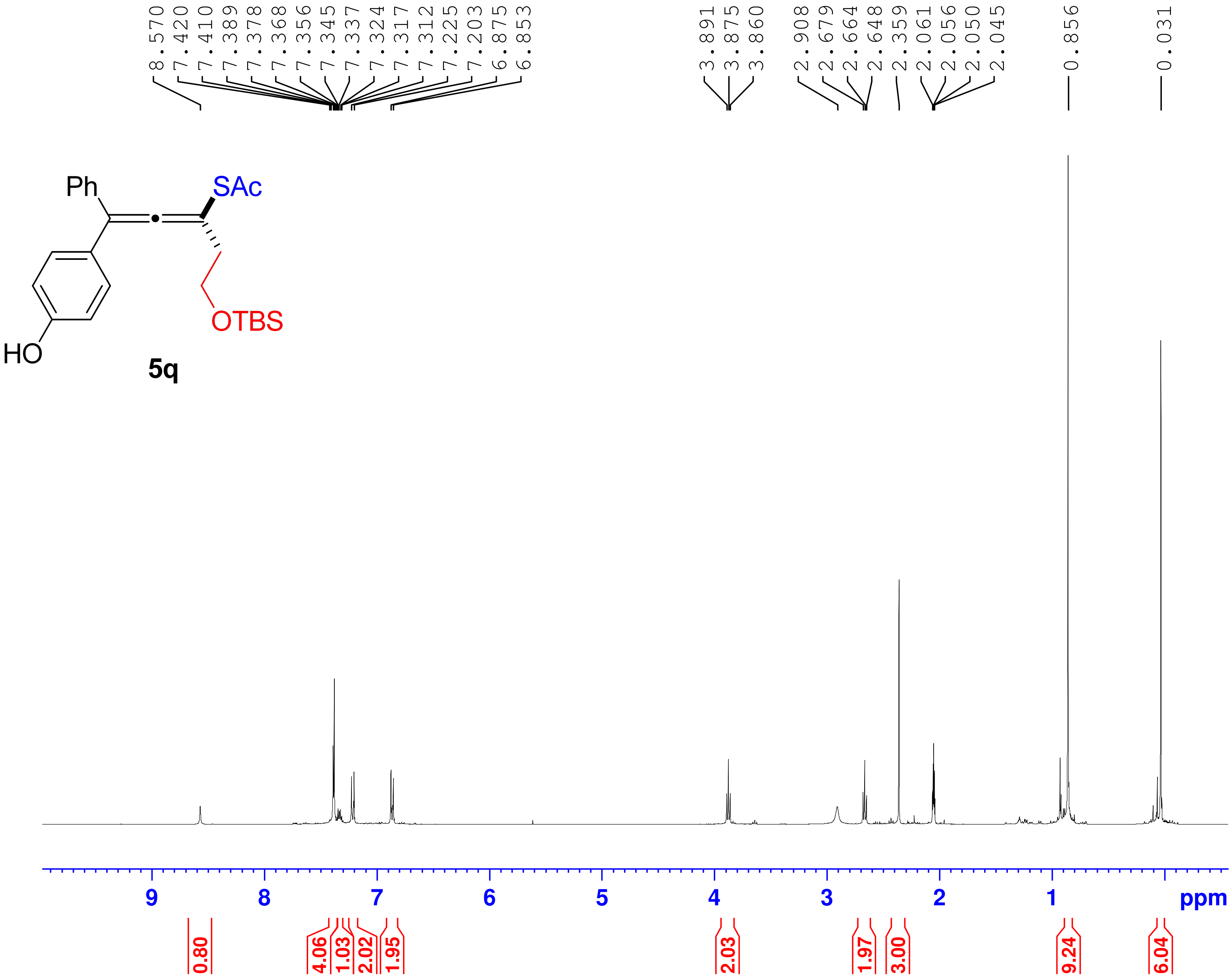

Current Data Parameters
NAME qdy-20103-2 H
EXPNO 3
PROCNO 1

F2 - Acquisition Parameters
Date_ 20160402
Time 13.53
INSTRUM spect
PROBHD 5 mm PABBO BB/
PULPROG zg30
TD 65536
SOLVENT Acetone
NS 10
DS 0
SWH 8012.820 Hz
FIDRES 0.122266 Hz
AQ 4.0894465 sec
RG 54.81
DW 62.400 usec
DE 6.50 usec
TE 297.8 K
D1 1.00000000 sec
TD0 1

======== CHANNEL f1 ========
SFO1 400.1324710 MHz
NUC1 1H
P1 14.50 usec
PLW1 11.99499989 W

F2 - Processing parameters
SI 65536
SF 400.1300067 MHz
WDW EM
SSB 0
LB 0.30 Hz
GB 0
PC 1.00

Supplementary Figure 139. ^{1}H NMR spectrum for 5q

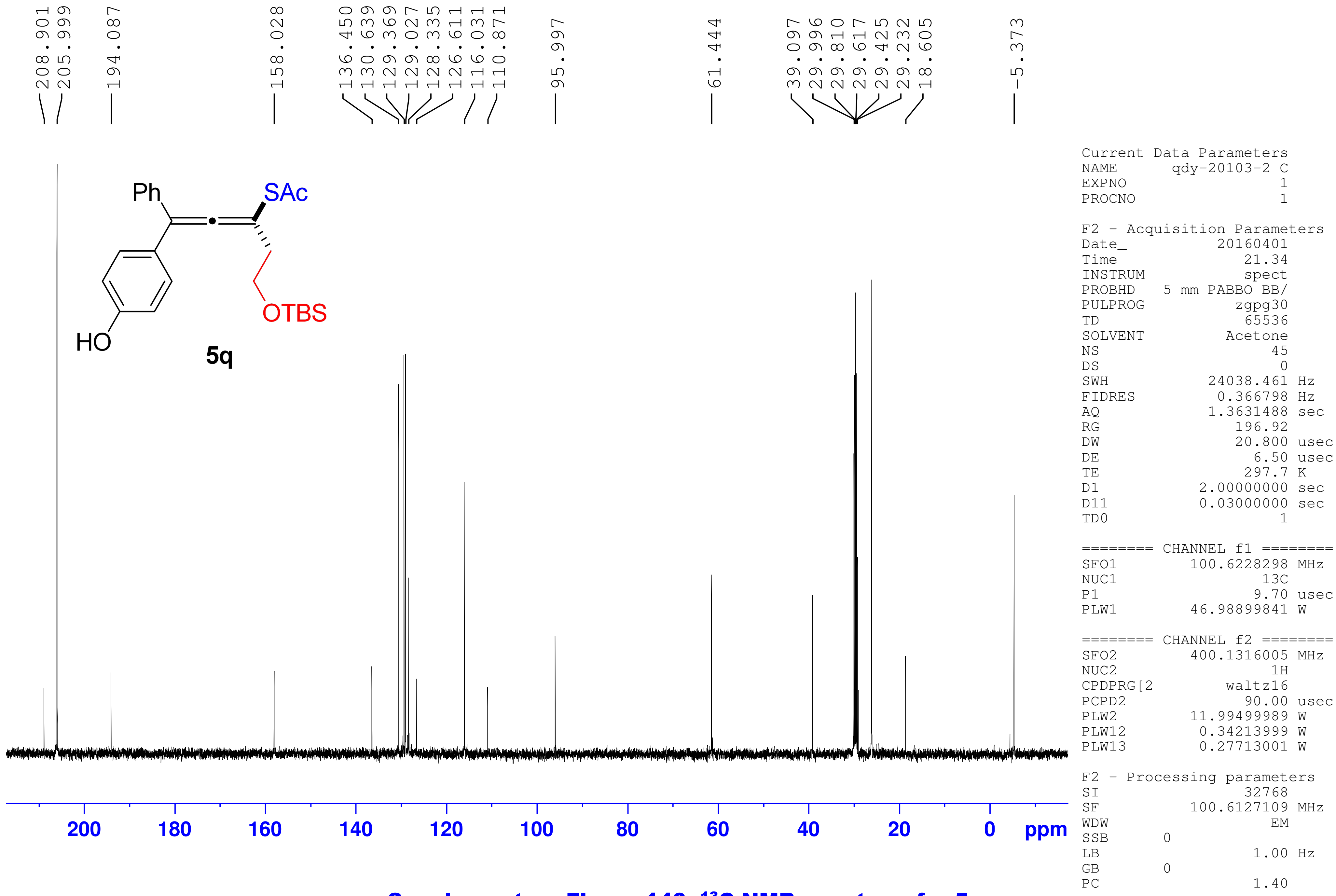

Supplementary Figure 140. ^{13}C NMR spectrum for 5q

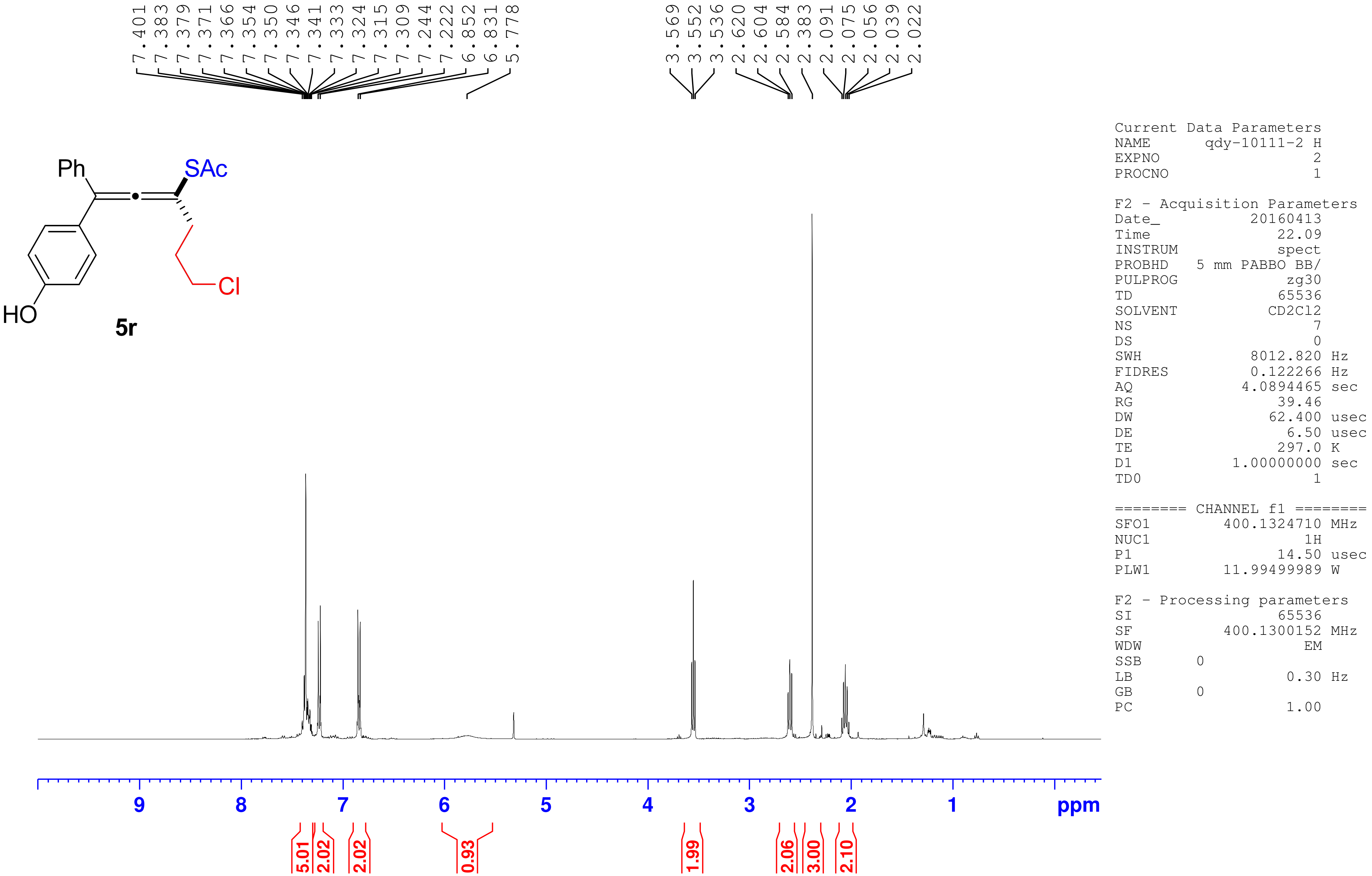

Supplementary Figure 141. ^{1}H NMR spectrum for 5r

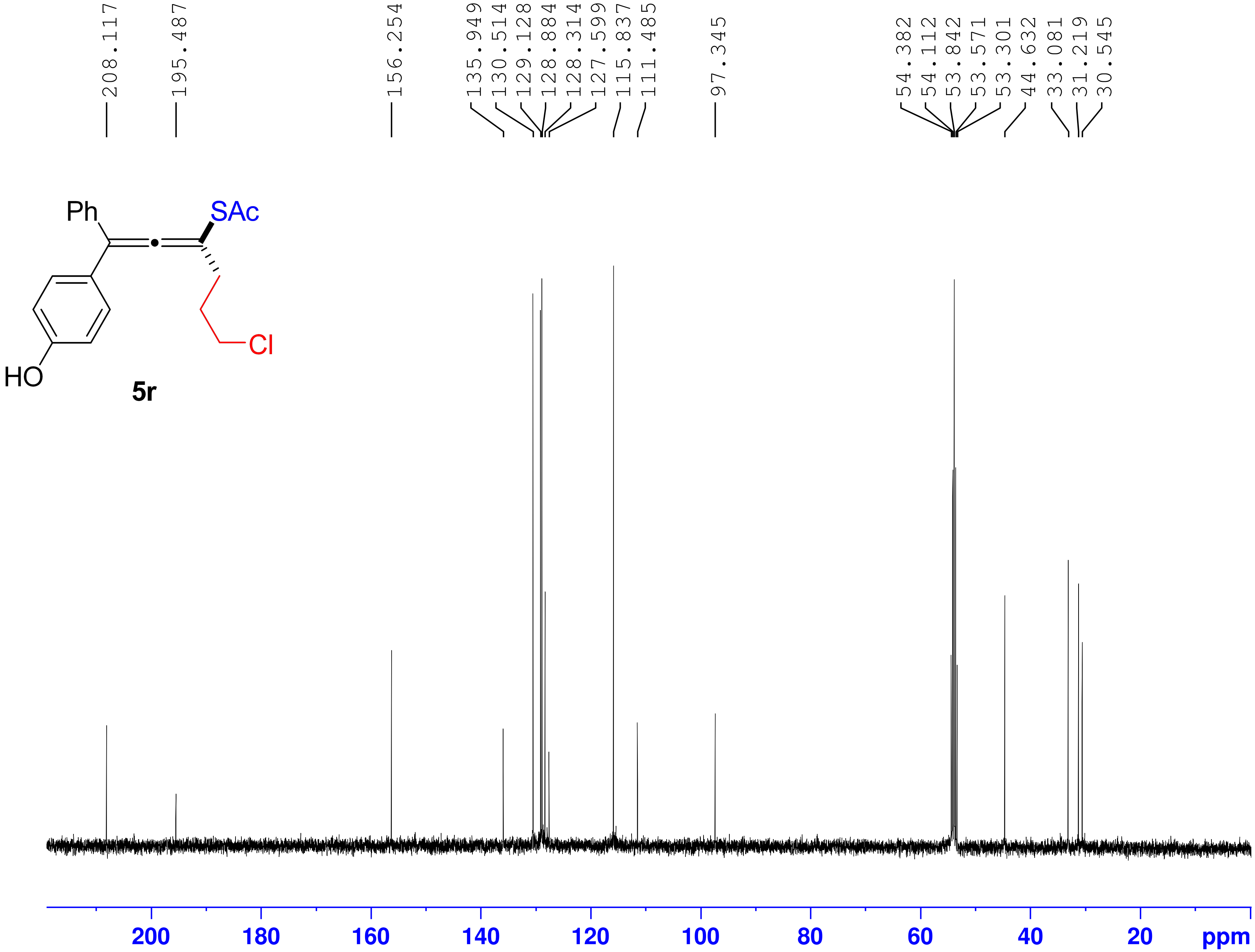

5r

Current Data Parameters
NAME qdy-10111-2 C
EXPNO 2
PROCNO 1

F2 - Acquisition Parameters
Date_ 20160413
Time 22.13
INSTRUM spect
PROBHD 5 mm PABBO BB/
PULPROG zgpg30
TD 65536
SOLVENT CD2Cl2
NS 54
DS 0
SWH 24038.461 Hz
FIDRES 0.366798 Hz
AQ 1.3631488 sec
RG 196.92
DW 20.800 usec
DE 6.50 usec
TE 298.0 K
D1 2.00000000 sec
D11 0.03000000 sec
TD0 1

======== CHANNEL f1 ========
SFO1 100.6228298 MHz
NUC1 13C
P1 9.70 usec
PLW1 46.98899841 W

======== CHANNEL f2 ========
SFO2 400.1316005 MHz
NUC2 1H
CPDPRG[2 waltz16
PCPD2 90.00 usec
PLW2 11.99499989 W
PLW12 0.34213999 W
PLW13 0.27713001 W

F2 - Processing parameters
SI 32768
SF 100.6127306 MHz
WDW EM
SSB 0
LB 1.00 Hz
GB 0
PC 1.40

Supplementary Figure 142. ^{13}C NMR spectrum for 5r

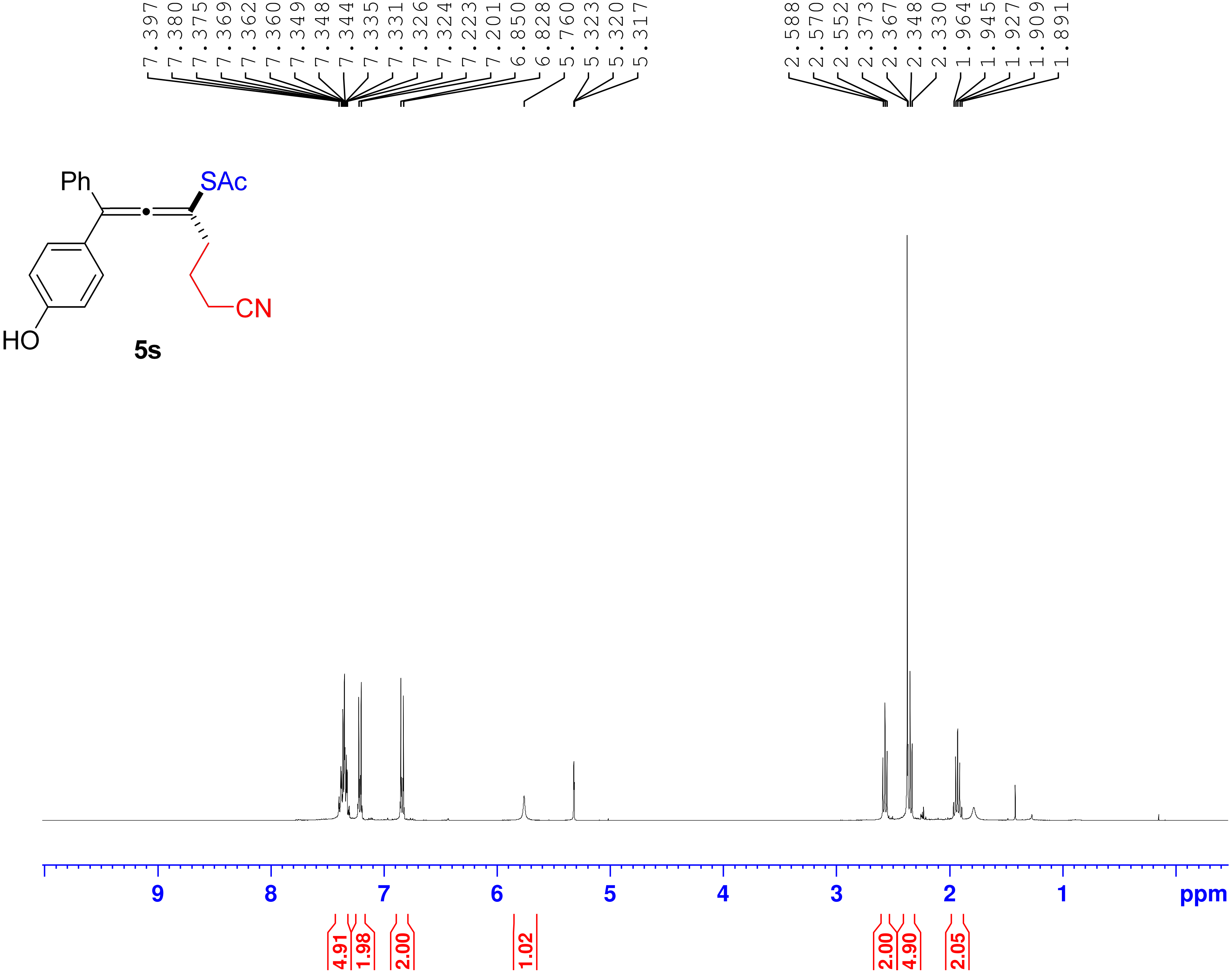

5s

```
Current Data Parameters
NAME        qdy-20123-2 H
EXPNO                   4
PROCNO                  1

F2 - Acquisition Parameters
Date_            20160425
Time                16.46
INSTRUM             spect
PROBHD   5 mm PABBO BB/
PULPROG              zg30
TD                  65536
SOLVENT            CD2Cl2
NS                      5
DS                      0
SWH              8012.820 Hz
FIDRES           0.122266 Hz
AQ              4.0894465 sec
RG                  88.84
DW                 62.400 usec
DE                   6.50 usec
TE                  297.5 K
D1             1.00000000 sec
TD0                     1

======== CHANNEL f1 ========
SFO1          400.1324710 MHz
NUC1                   1H
P1                  14.50 usec
PLW1          11.99499989 W

F2 - Processing parameters
SI                  65536
SF            400.1300153 MHz
WDW                    EM
SSB      0
LB                   0.30 Hz
GB       0
PC                   1.00
```

Supplementary Figure 143. ^{1}H NMR spectrum for 5s

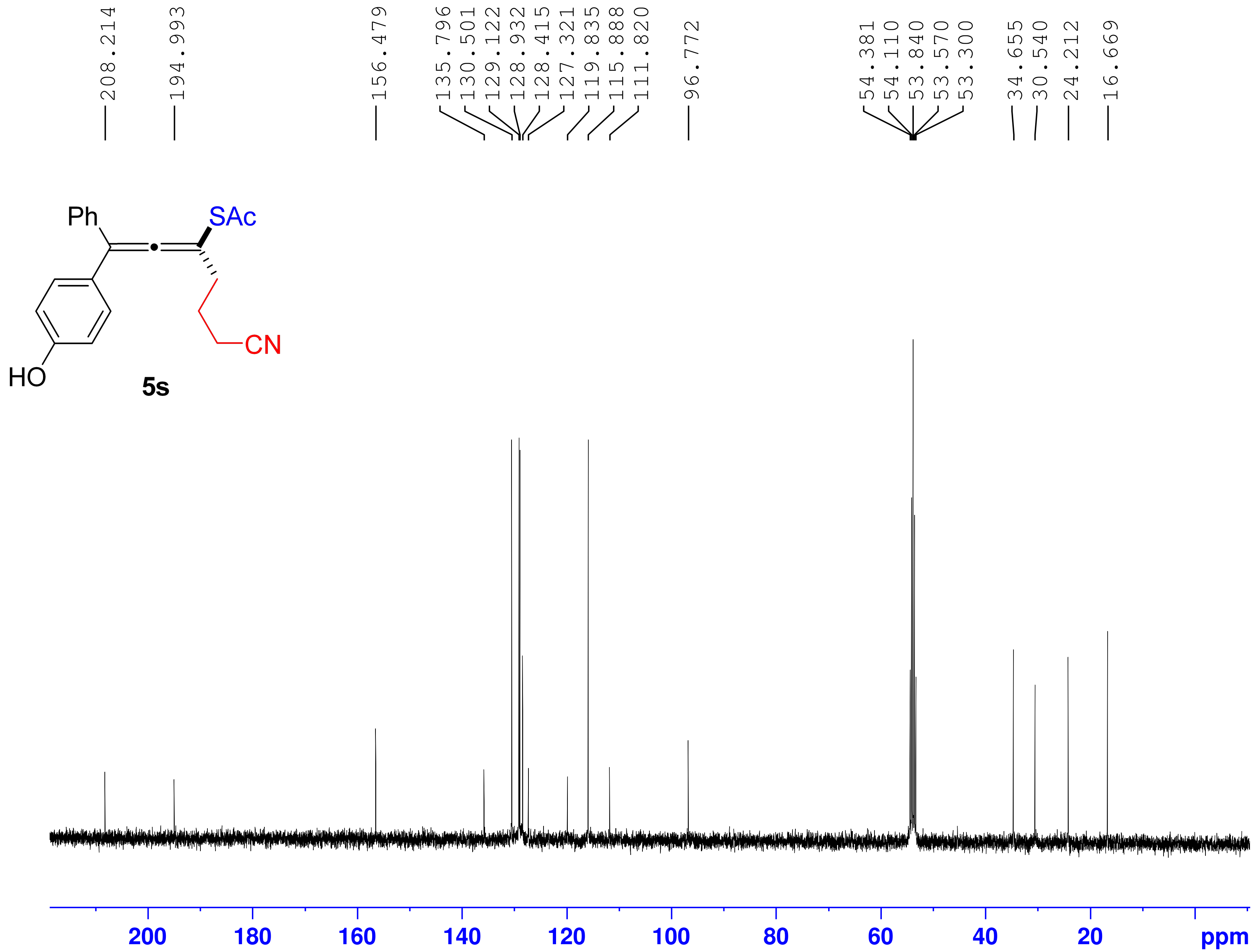

Current Data Parameters
NAME qdy-20123-2 C
EXPNO 1
PROCNO 1

F2 - Acquisition Parameters
Date_ 20160423
Time 21.59
INSTRUM spect
PROBHD 5 mm PABBO BB/
PULPROG zgpg30
TD 65536
SOLVENT CD2Cl2
NS 256
DS 0
SWH 24038.461 Hz
FIDRES 0.366798 Hz
AQ 1.3631488 sec
RG 196.92
DW 20.800 usec
DE 6.50 usec
TE 299.5 K
D1 2.00000000 sec
D11 0.03000000 sec
TD0 1

======== CHANNEL f1 ========
SFO1 100.6228298 MHz
NUC1 13C
P1 9.70 usec
PLW1 46.98899841 W

======== CHANNEL f2 ========
SFO2 400.1316005 MHz
NUC2 1H
CPDPRG[2 waltz16
PCPD2 90.00 usec
PLW2 11.99499989 W
PLW12 0.34213999 W
PLW13 0.27713001 W

F2 - Processing parameters
SI 32768
SF 100.6127284 MHz
WDW EM
SSB 0
LB 1.00 Hz
GB 0
PC 1.40

Supplementary Figure 144. ^{13}C NMR spectrum for 5s

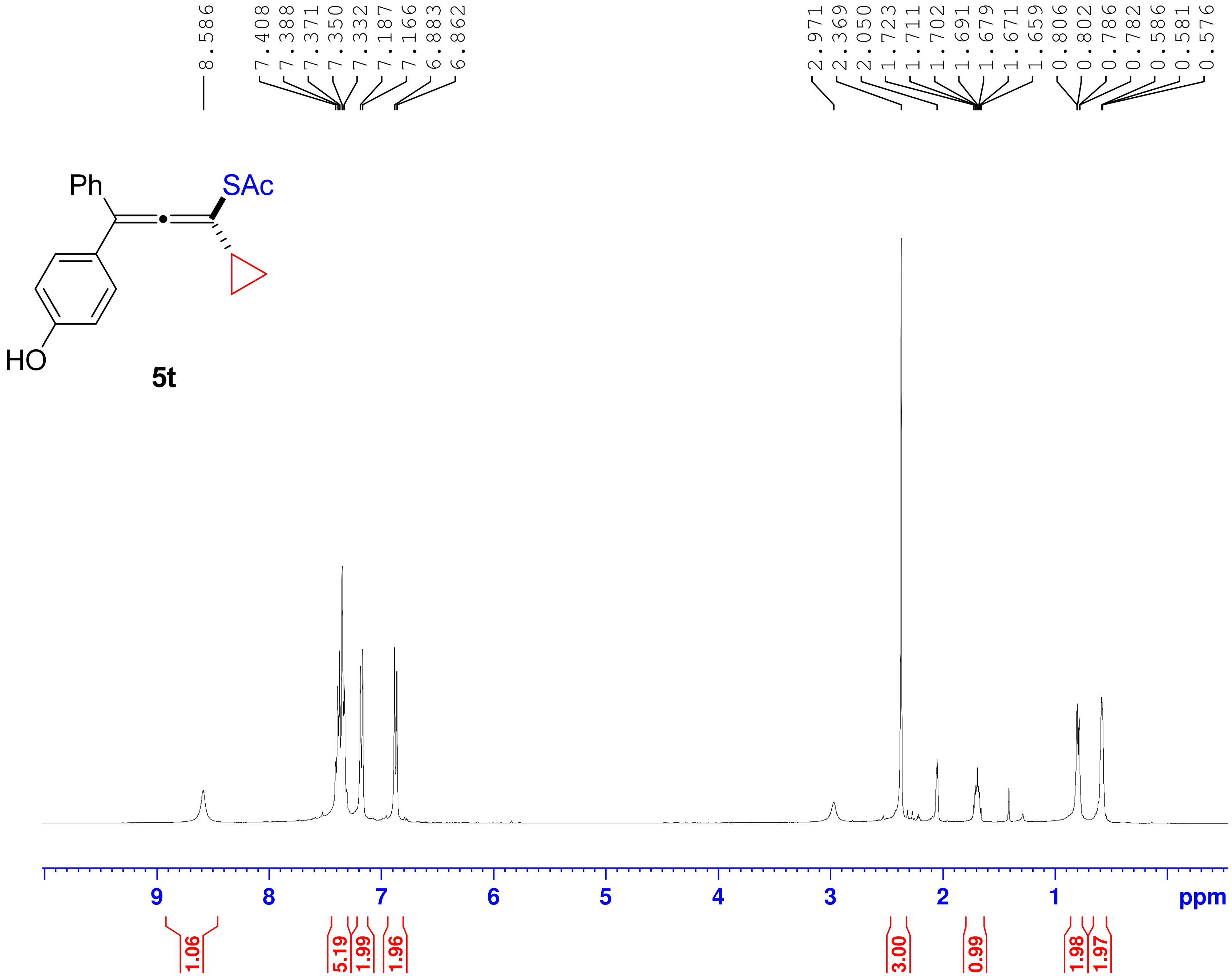

Current Data Parameters
NAME qdy-20103-1 H
EXPNO 2
PROCNO 1

F2 - Acquisition Parameters
Date_ 20160408
Time 13.37
INSTRUM spect
PROBHD 5 mm PABBO BB/
PULPROG zg30
TD 65536
SOLVENT Acetone
NS 16
DS 0
SWH 8012.820 Hz
FIDRES 0.122266 Hz
AQ 4.0894465 sec
RG 54.81
DW 62.400 usec
DE 6.50 usec
TE 297.8 K
D1 1.00000000 sec
TD0 1

======== CHANNEL f1 ========
SFO1 400.1324710 MHz
NUC1 1H
P1 14.50 usec
PLW1 11.99499989 W

F2 - Processing parameters
SI 65536
SF 400.1300066 MHz
WDW EM
SSB 0
LB 0.30 Hz
GB 0
PC 1.00

Supplementary Figure 145. ^{1}H NMR spectrum for 5t

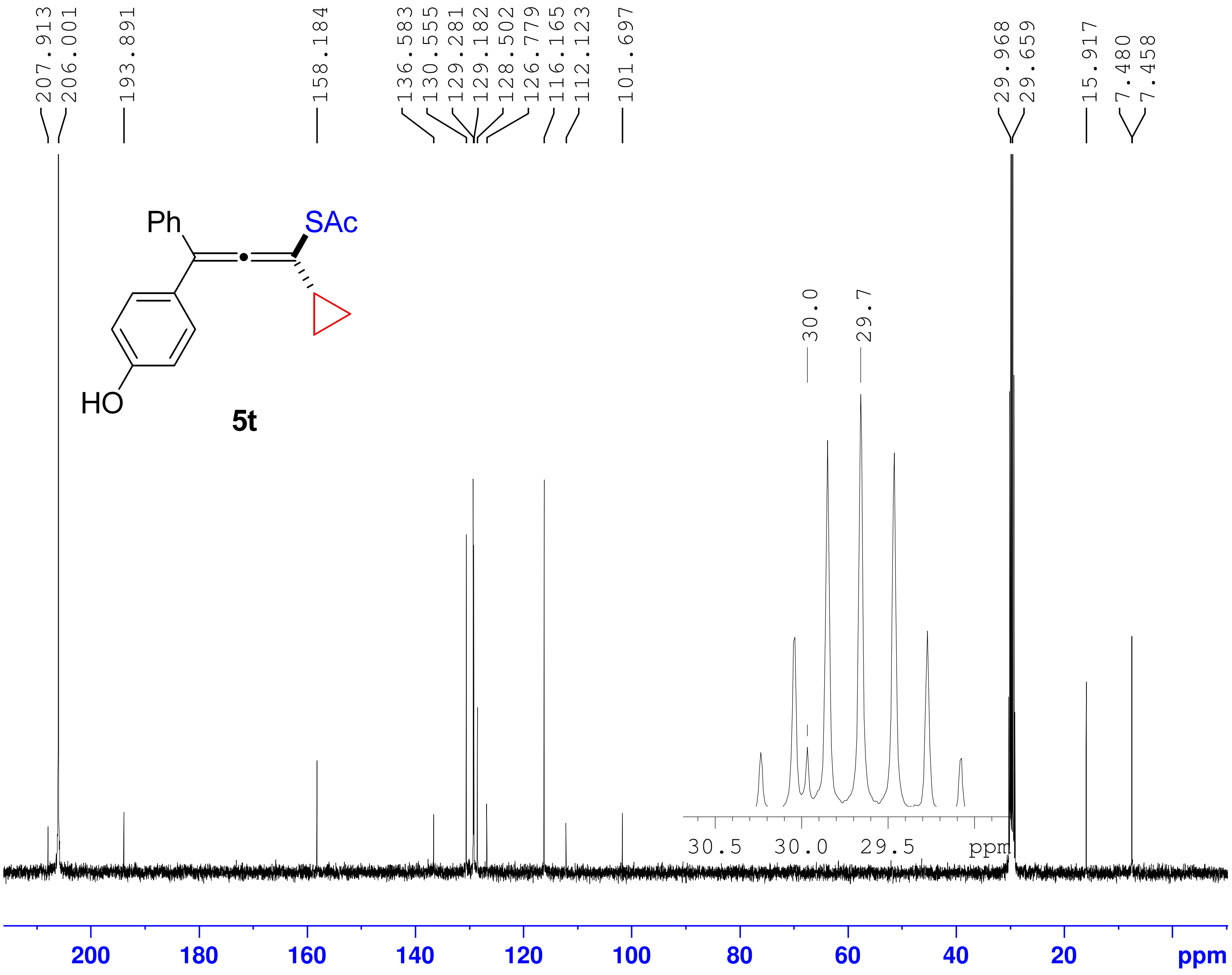


```
Current Data Parameters
NAME       qdy-20103-1 C
EXPNO                  3
PROCNO                 1

F2 - Acquisition Parameters
Date_           20160408
Time               13.44
INSTRUM            spect
PROBHD   5 mm PABBO BB/
PULPROG          zgpg30
TD                65536
SOLVENT          Acetone
NS                   116
DS                     0
SWH           24038.461 Hz
FIDRES         0.366798 Hz
AQ            1.3631488 sec
RG                196.92
DW                20.800 usec
DE                  6.50 usec
TE                 299.3 K
D1            2.00000000 sec
D11           0.03000000 sec
TD0                    1

======== CHANNEL f1 ========
SFO1         100.6228298 MHz
NUC1                 13C
P1                  9.70 usec
PLW1         46.98899841 W

======== CHANNEL f2 ========
SFO2         400.1316005 MHz
NUC2                  1H
CPDPRG[2         waltz16
PCPD2              90.00 usec
PLW2         11.99499989 W
PLW12         0.34213999 W
PLW13         0.27713001 W

F2 - Processing parameters
SI                 32768
SF           100.6126993 MHz
WDW                   EM
SSB      0
LB                  1.00 Hz
GB       0
PC                  1.40
```

Supplementary Figure 146. ^{13}C NMR spectrum for 5t

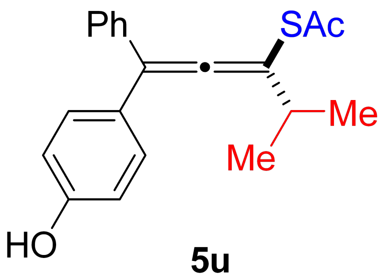

5u

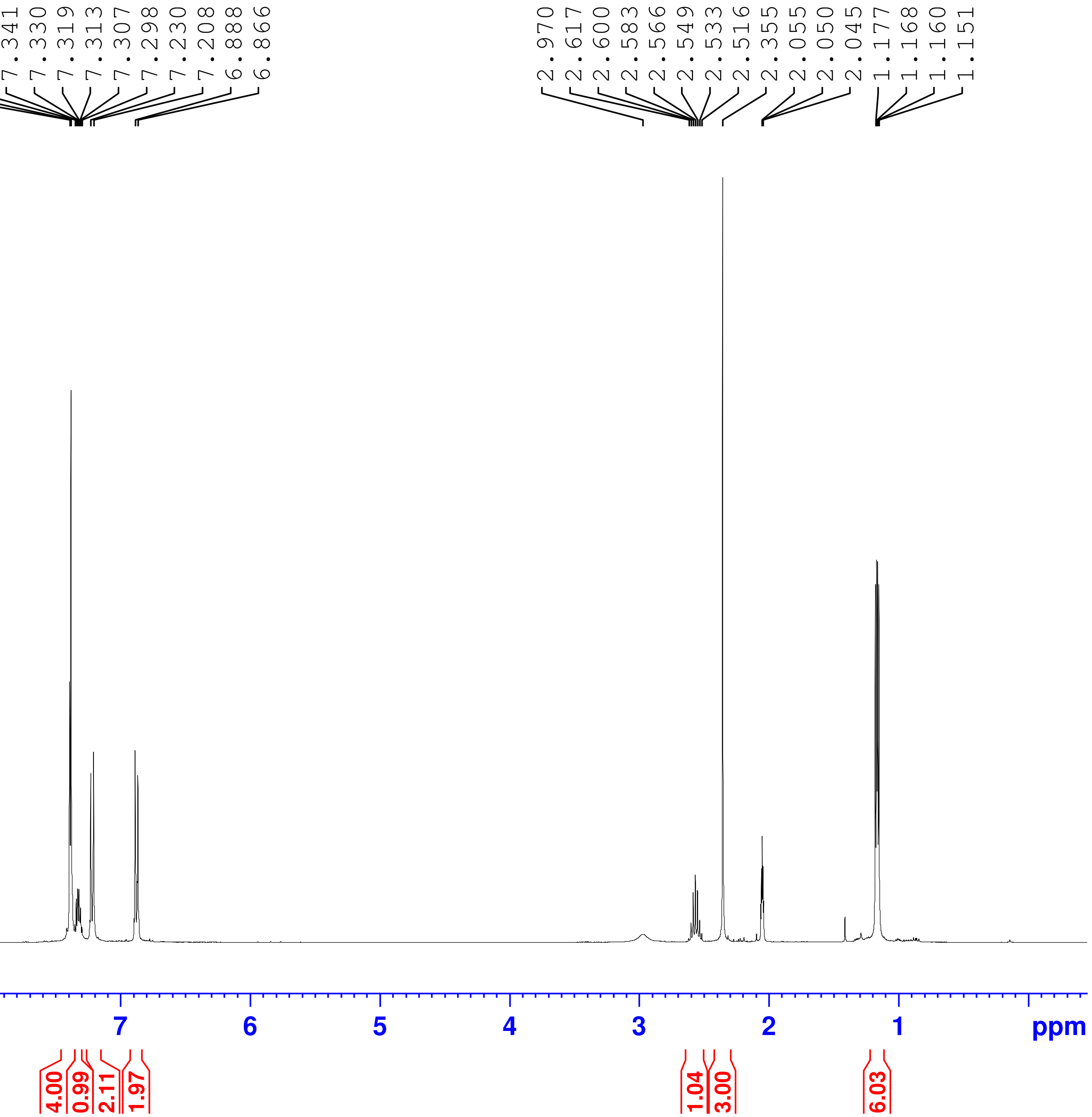


```
Current Data Parameters
NAME          qdy-20012-1 H
EXPNO                     1
PROCNO                    2

F2 - Acquisition Parameters
Date_              20160401
Time                  21.59
INSTRUM               spect
PROBHD     5 mm PABBO BB/
PULPROG                zg30
TD                    65536
SOLVENT             Acetone
NS                        6
DS                        0
SWH              8012.820 Hz
FIDRES           0.122266 Hz
AQ              4.0894465 sec
RG                    62.93
DW                 62.400 usec
DE                   6.50 usec
TE                  297.5 K
D1             1.00000000 sec
TD0                       1

======== CHANNEL f1 ========
SFO1          400.1324710 MHz
NUC1                     1H
P1                  14.50 usec
PLW1          11.99499989 W

F2 - Processing parameters
SI                    65536
SF            400.1300067 MHz
WDW                      EM
SSB       0
LB                   0.30 Hz
GB        0
PC                     1.00
```

Supplementary Figure 147. ^{1}H NMR spectrum for 5u

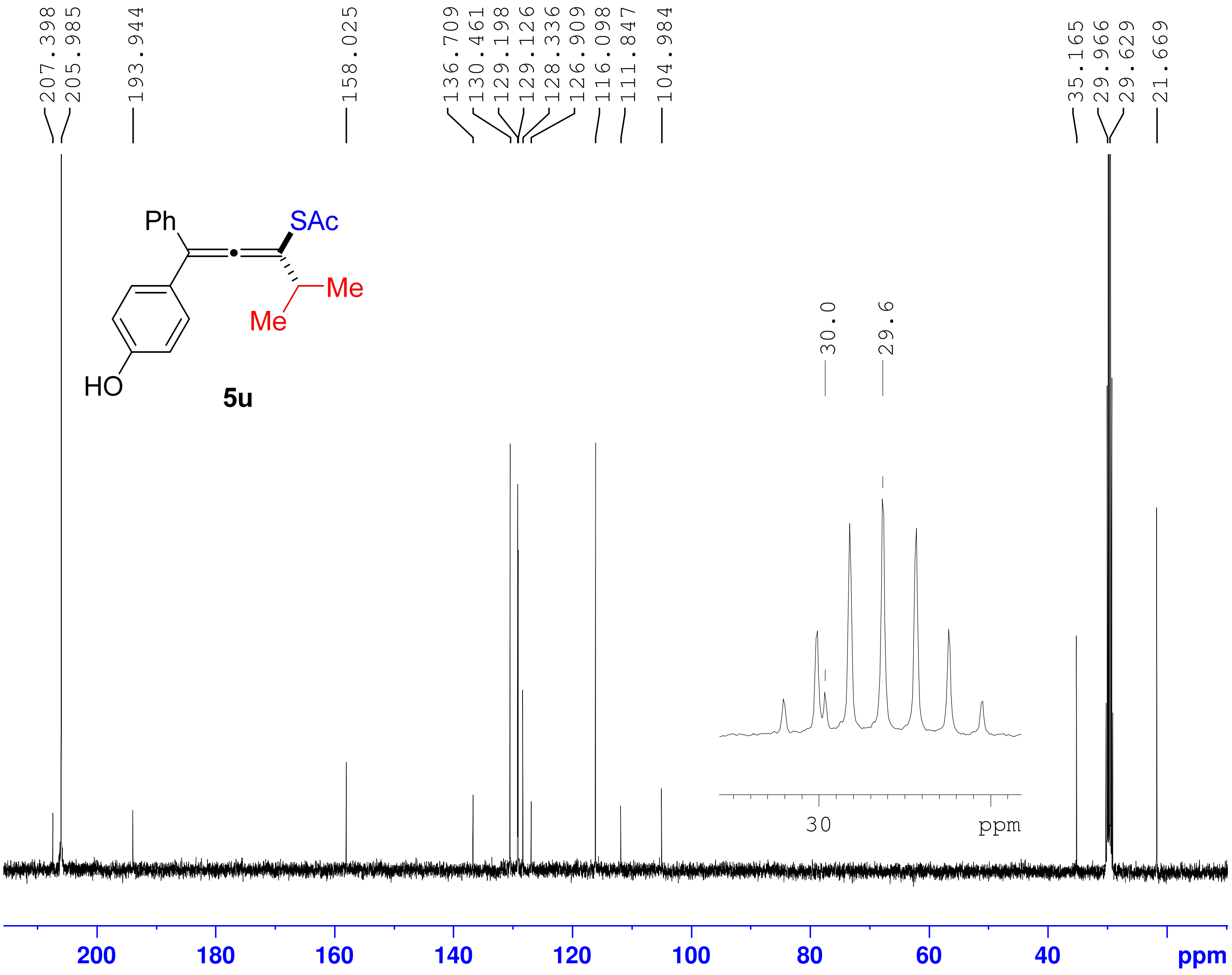

Current Data Parameters
NAME qdy-20012-1 C
EXPNO 1
PROCNO 2

F2 - Acquisition Parameters
Date_ 20160401
Time 21.55
INSTRUM spect
PROBHD 5 mm PABBO BB/
PULPROG zgpg30
TD 65536
SOLVENT Acetone
NS 60
DS 0
SWH 24038.461 Hz
FIDRES 0.366798 Hz
AQ 1.3631488 sec
RG 196.92
DW 20.800 usec
DE 6.50 usec
TE 297.7 K
D1 2.00000000 sec
D11 0.03000000 sec
TD0 1

======== CHANNEL f1 ========
SFO1 100.6228298 MHz
NUC1 13C
P1 9.70 usec
PLW1 46.98899841 W

======== CHANNEL f2 ========
SFO2 400.1316005 MHz
NUC2 1H
CPDPRG[2 waltz16
PCPD2 90.00 usec
PLW2 11.99499989 W
PLW12 0.34213999 W
PLW13 0.27713001 W

F2 - Processing parameters
SI 32768
SF 100.6127029 MHz
WDW EM
SSB 0
LB 1.00 Hz
GB 0
PC 1.40

Supplementary Figure 148. ^{13}C NMR spectrum for 5u

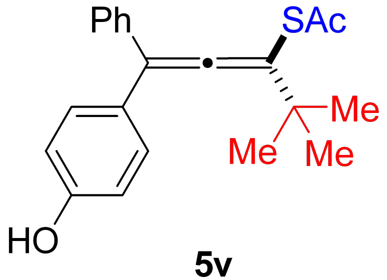

5v

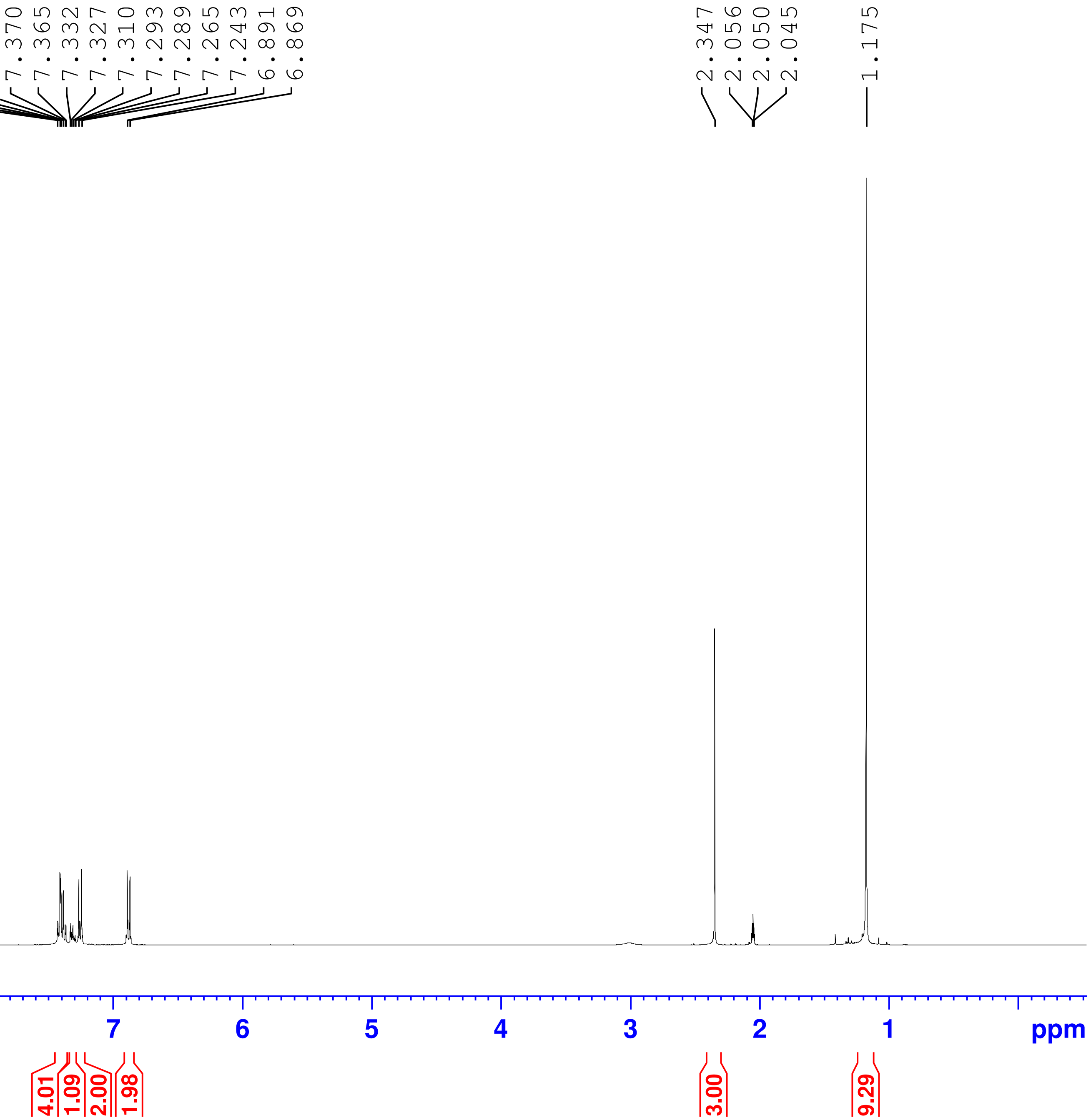

Current Data Parameters
NAME qdy-20099-2 H
EXPNO 1
PROCNO 1

F2 - Acquisition Parameters
Date_ 20160402
Time 14.20
INSTRUM spect
PROBHD 5 mm PABBO BB/
PULPROG zg30
TD 65536
SOLVENT Acetone
NS 8
DS 0
SWH 8012.820 Hz
FIDRES 0.122266 Hz
AQ 4.0894465 sec
RG 70.97
DW 62.400 usec
DE 6.50 usec
TE 298.1 K
D1 1.00000000 sec
TD0 1

======== CHANNEL f1 ========
SFO1 400.1324710 MHz
NUC1 1H
P1 14.50 usec
PLW1 11.99499989 W

F2 - Processing parameters
SI 65536
SF 400.1300067 MHz
WDW EM
SSB 0
LB 0.30 Hz
GB 0
PC 1.00

Supplementary Figure 149. ^{1}H NMR spectrum for 5v

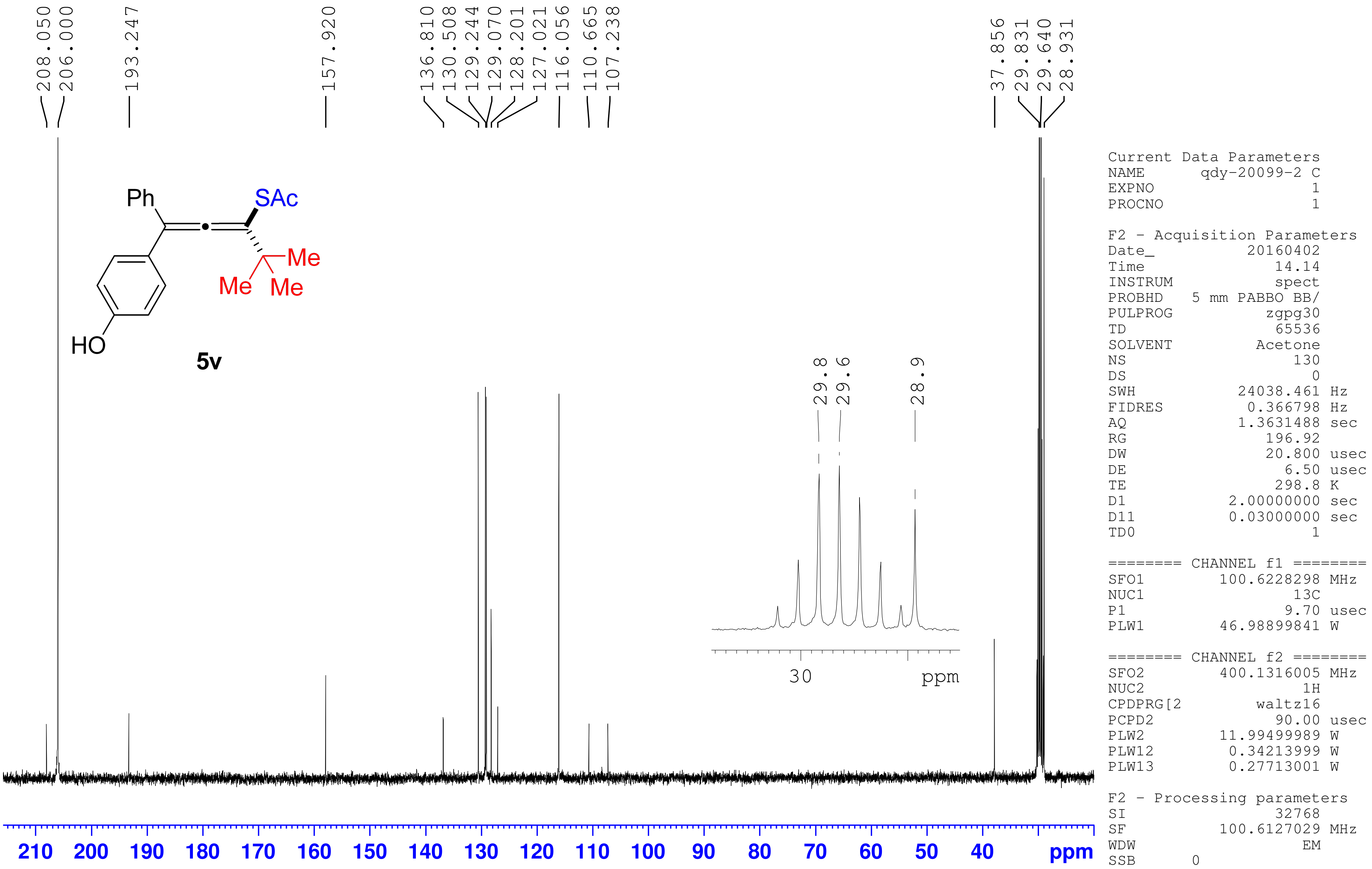

Supplementary Figure 150. ^{13}C **NMR spectrum for 5v**

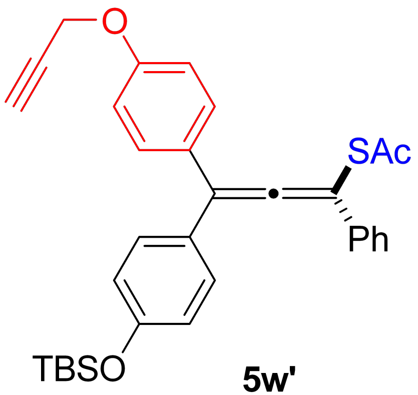

5w'

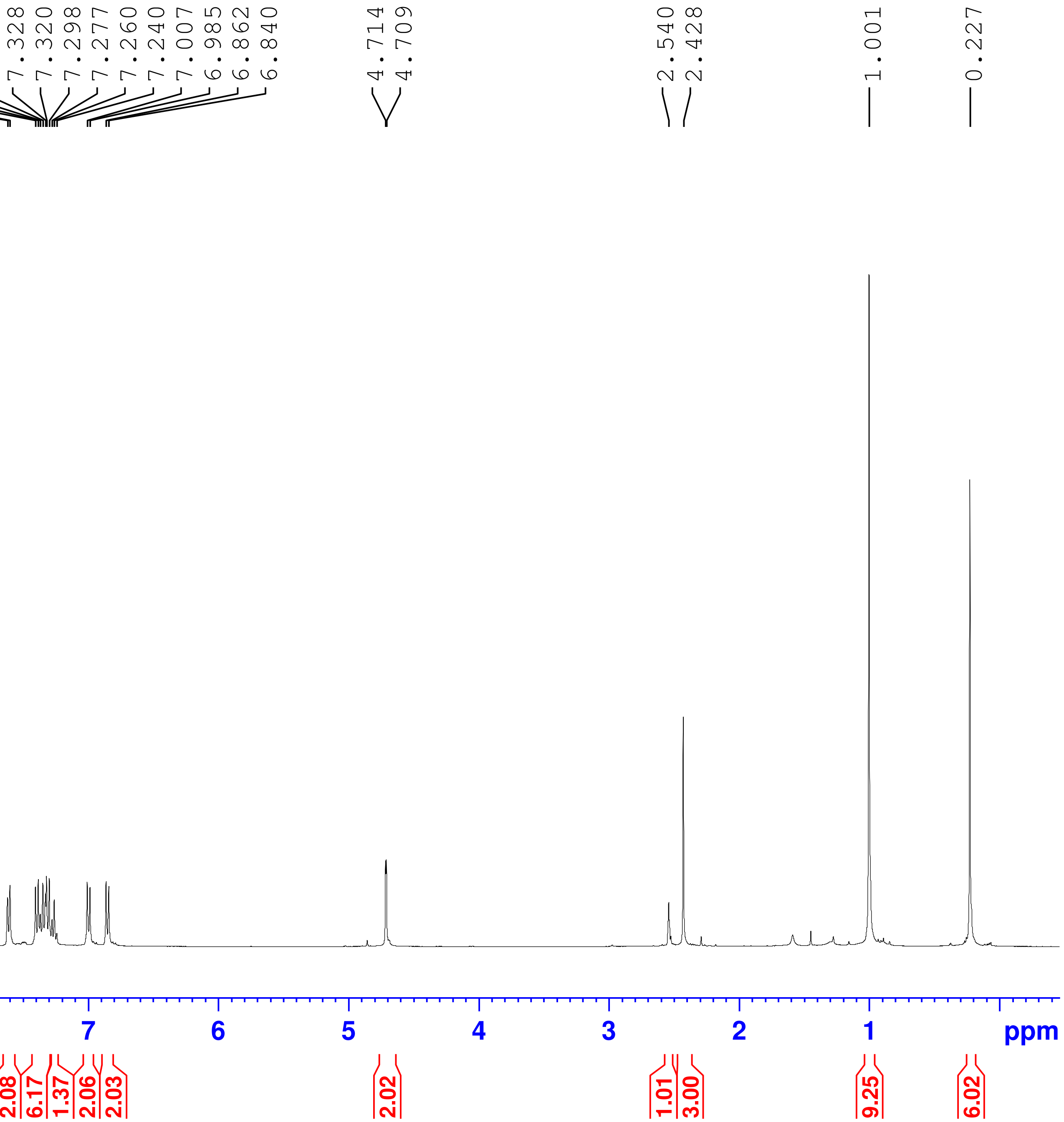


```
Current Data Parameters
NAME        qdy-20160-2 H
EXPNO                   1
PROCNO                  1

F2 - Acquisition Parameters
Date_            20160525
Time                13.41
INSTRUM             spect
PROBHD    5 mm PABBO BB/
PULPROG              zg30
TD                  65536
SOLVENT             CDCl3
NS                      5
DS                      0
SWH             8012.820 Hz
FIDRES          0.122266 Hz
AQ             4.0894465 sec
RG                  49.32
DW                62.400 usec
DE                  6.50 usec
TE                 297.9 K
D1            1.00000000 sec
TD0                     1

======== CHANNEL f1 ========
SFO1         400.1324710 MHz
NUC1                  1H
P1                 14.50 usec
PLW1         11.99499989 W

F2 - Processing parameters
SI                  65536
SF           400.1300089 MHz
WDW                    EM
SSB       0
LB                  0.30 Hz
GB        0
PC                  1.00
```

Supplementary Figure 151. ^{1}H NMR spectrum for 5w'

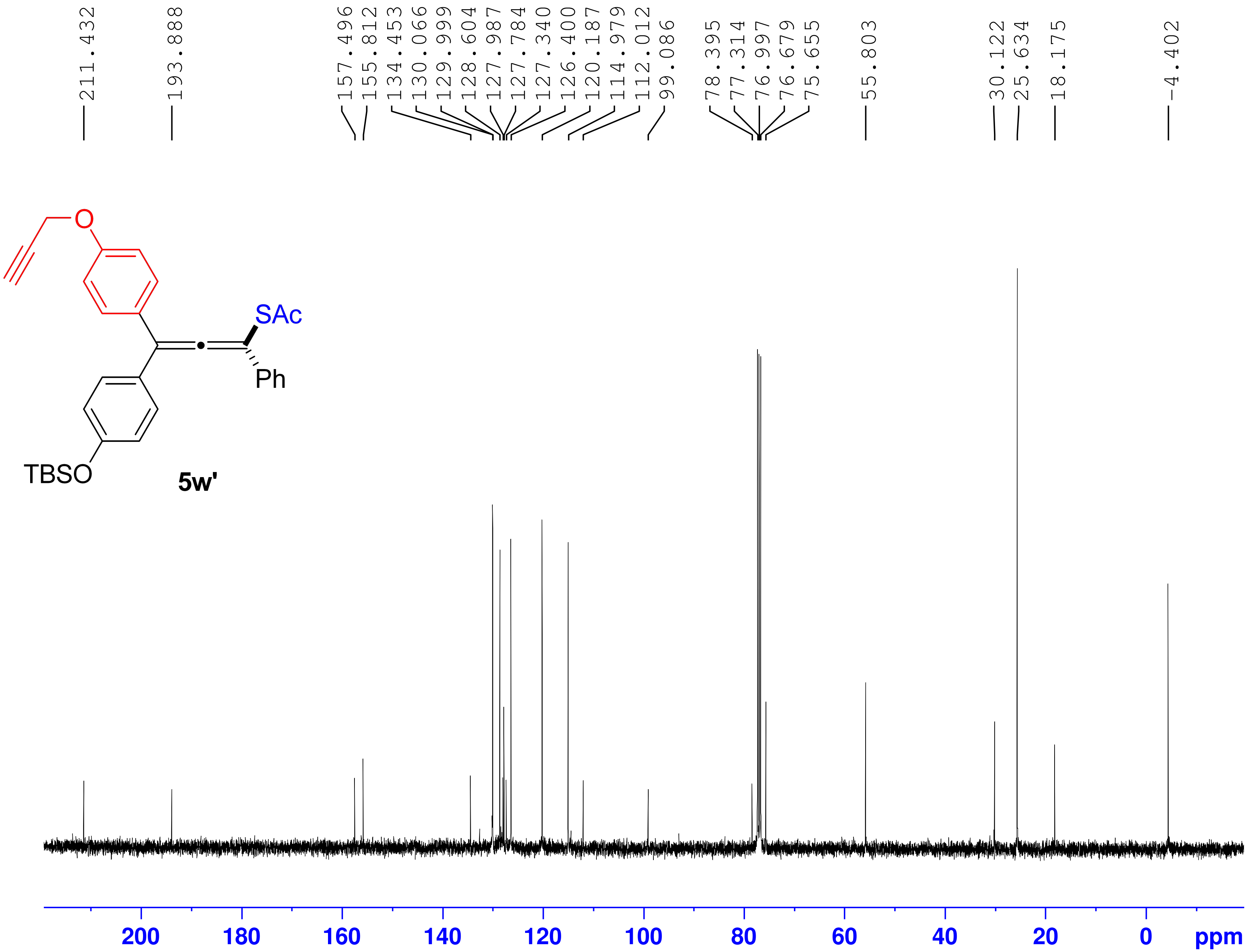


```
Current Data Parameters
NAME      qdy-20160-2 C
EXPNO                 1
PROCNO                1

F2 - Acquisition Parameters
Date_          20160525
Time              13.45
INSTRUM           spect
PROBHD   5 mm PABBO BB/
PULPROG          zgpg30
TD                65536
SOLVENT           CDCl3
NS                  126
DS                    0
SWH           24038.461 Hz
FIDRES         0.366798 Hz
AQ            1.3631488 sec
RG               196.92
DW               20.800 usec
DE                 6.50 usec
TE                299.0 K
D1           2.00000000 sec
D11          0.03000000 sec
TD0                   1

======== CHANNEL f1 ========
SFO1        100.6228298 MHz
NUC1                13C
P1                 9.70 usec
PLW1        46.98899841 W

======== CHANNEL f2 ========
SFO2        400.1316005 MHz
NUC2                 1H
CPDPRG[2        waltz16
PCPD2             90.00 usec
PLW2        11.99499989 W
PLW12        0.34213999 W
PLW13        0.27713001 W

F2 - Processing parameters
SI                32768
SF          100.6127744 MHz
WDW                  EM
SSB       0
LB                 1.00 Hz
GB        0
PC                 1.40
```

Supplementary Figure 152. ^{13}C NMR spectrum for 5w'

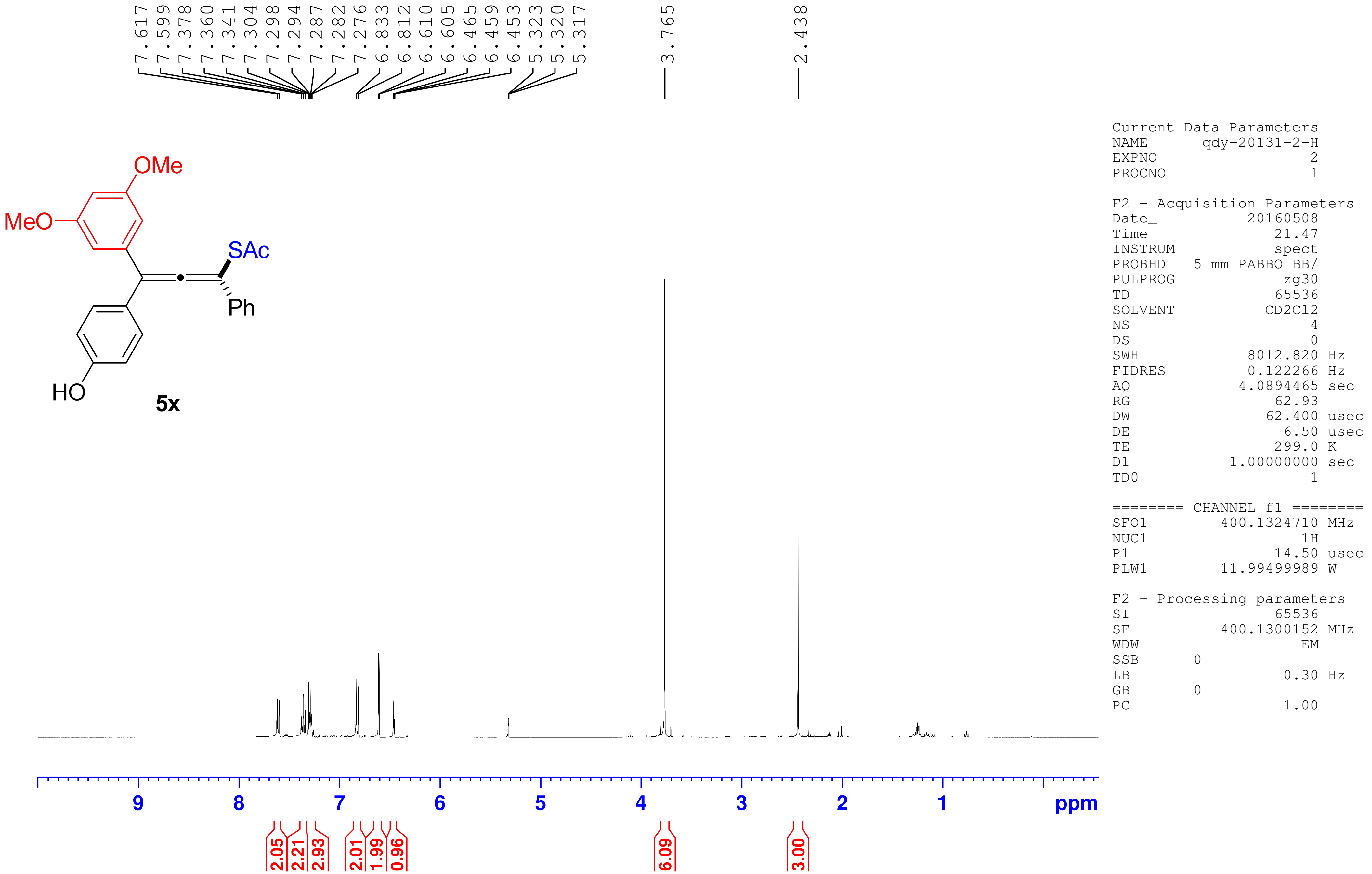

Supplementary Figure 153. ^{1}H NMR spectrum for 5x

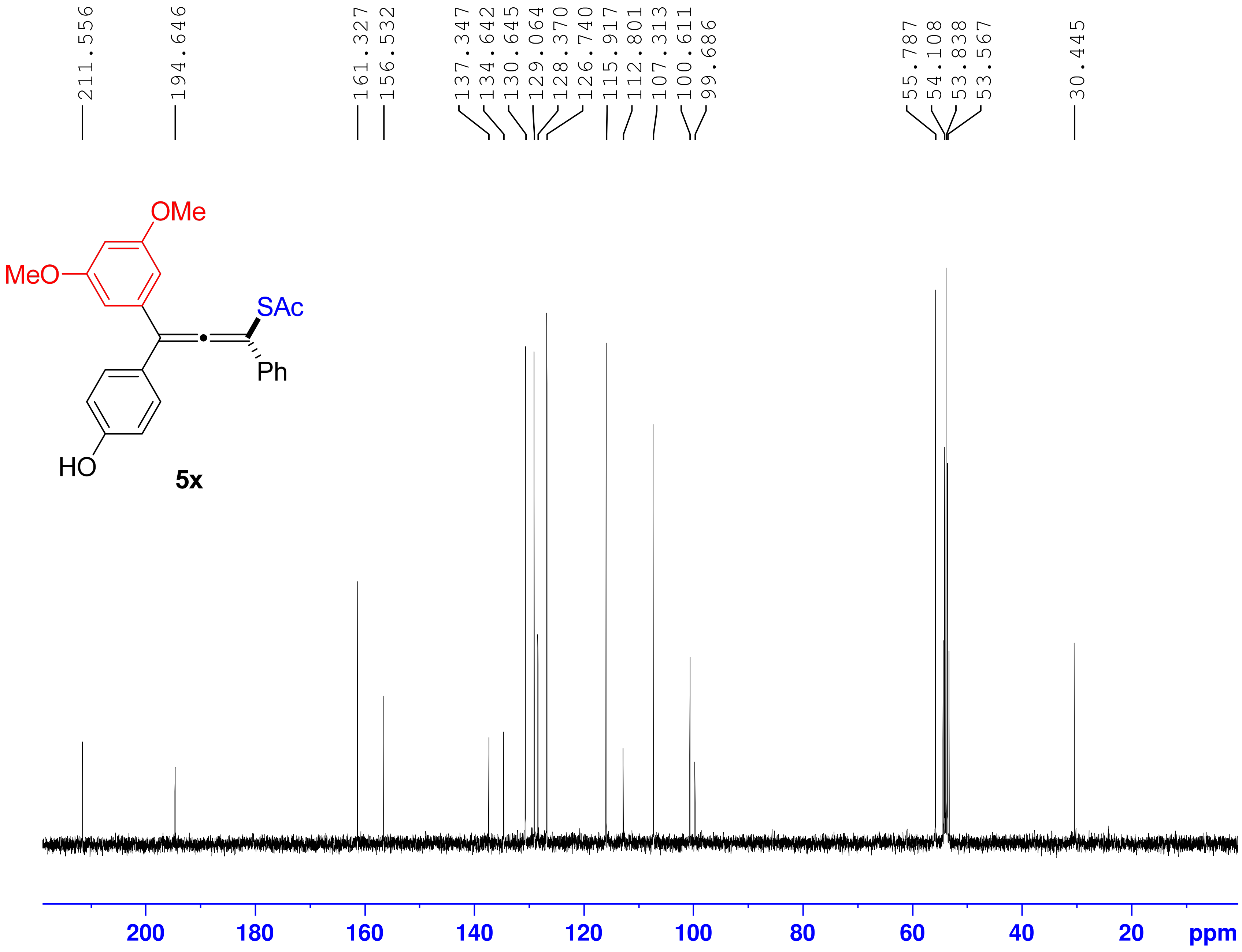

5x

```
Current Data Parameters
NAME        qdy-20131-2 C
EXPNO                   1
PROCNO                  1

F2 - Acquisition Parameters
Date_            20160429
Time                19.23
INSTRUM             spect
PROBHD    5 mm PABBO BB/
PULPROG           zgpg30
TD                 65536
SOLVENT           CD2Cl2
NS                   188
DS                     0
SWH            24038.461 Hz
FIDRES          0.366798 Hz
AQ             1.3631488 sec
RG                196.92
DW                20.800 usec
DE                  6.50 usec
TE                 297.7 K
D1            2.00000000 sec
D11           0.03000000 sec
TD0                    1

======== CHANNEL f1 ========
SFO1         100.6228298 MHz
NUC1                 13C
P1                  9.70 usec
PLW1         46.98899841 W

======== CHANNEL f2 ========
SFO2         400.1316005 MHz
NUC2                  1H
CPDPRG[2         waltz16
PCPD2              90.00 usec
PLW2         11.99499989 W
PLW12         0.34213999 W
PLW13         0.27713001 W

F2 - Processing parameters
SI                 32768
SF           100.6127299 MHz
WDW                   EM
SSB      0
LB                  1.00 Hz
GB       0
PC                  1.40
```

Supplementary Figure 154. ^{13}C NMR spectrum for 5x

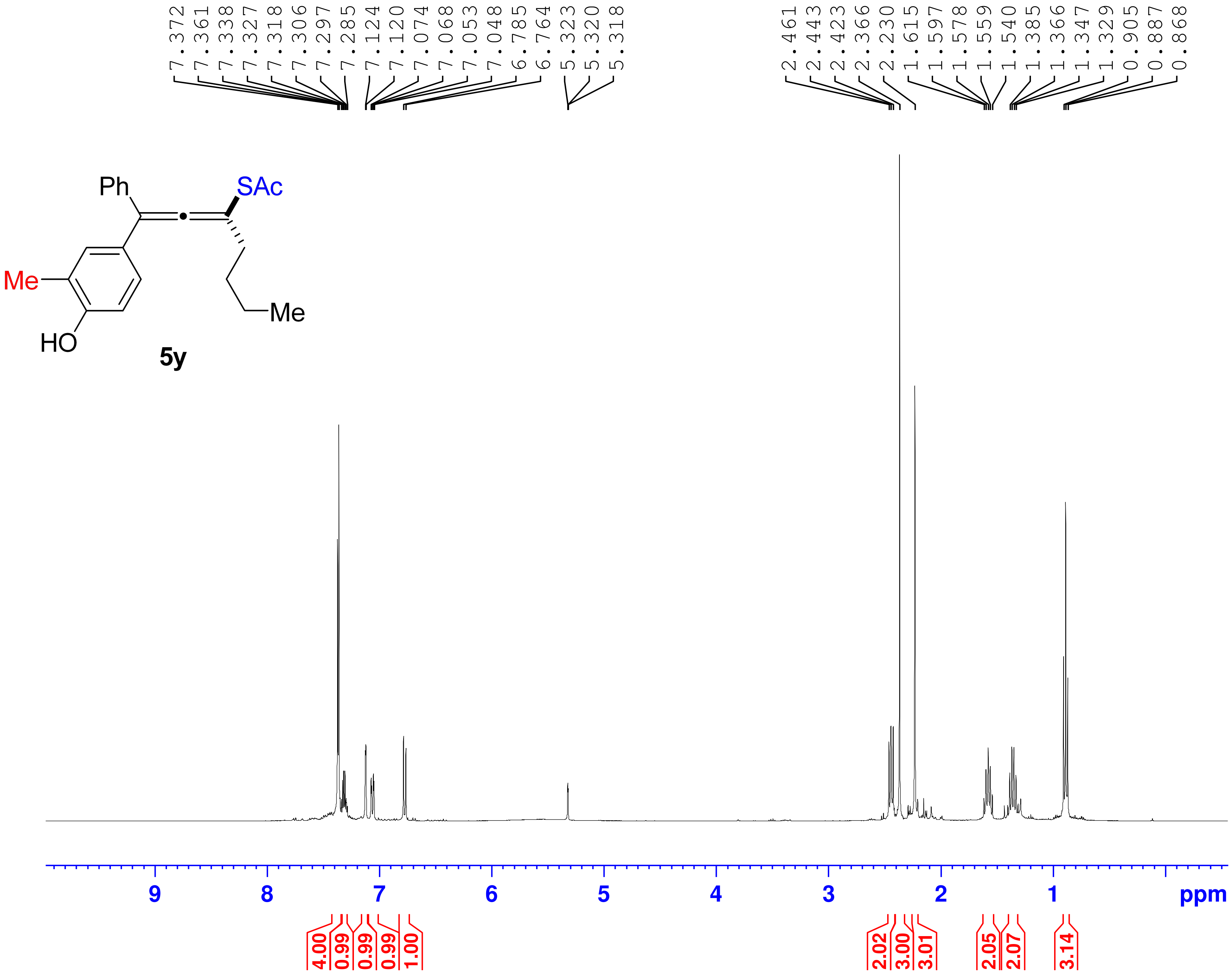


```
Current Data Parameters
NAME      qdy-20140-1 H
EXPNO                 1
PROCNO                1

F2 - Acquisition Parameters
Date_          20160508
Time              21.34
INSTRUM           spect
PROBHD   5 mm PABBO BB/
PULPROG            zg30
TD                65536
SOLVENT          CD2Cl2
NS                    4
DS                    0
SWH            8012.820 Hz
FIDRES         0.122266 Hz
AQ            4.0894465 sec
RG                49.32
DW               62.400 usec
DE                 6.50 usec
TE                298.7 K
D1           1.00000000 sec
TD0                   1

======== CHANNEL f1 ========
SFO1        400.1324710 MHz
NUC1                 1H
P1                14.50 usec
PLW1        11.99499989 W

F2 - Processing parameters
SI                65536
SF          400.1300152 MHz
WDW                  EM
SSB      0
LB                 0.30 Hz
GB       0
PC                 1.00
```

Supplementary Figure 155. ^{1}H NMR spectrum for 5y

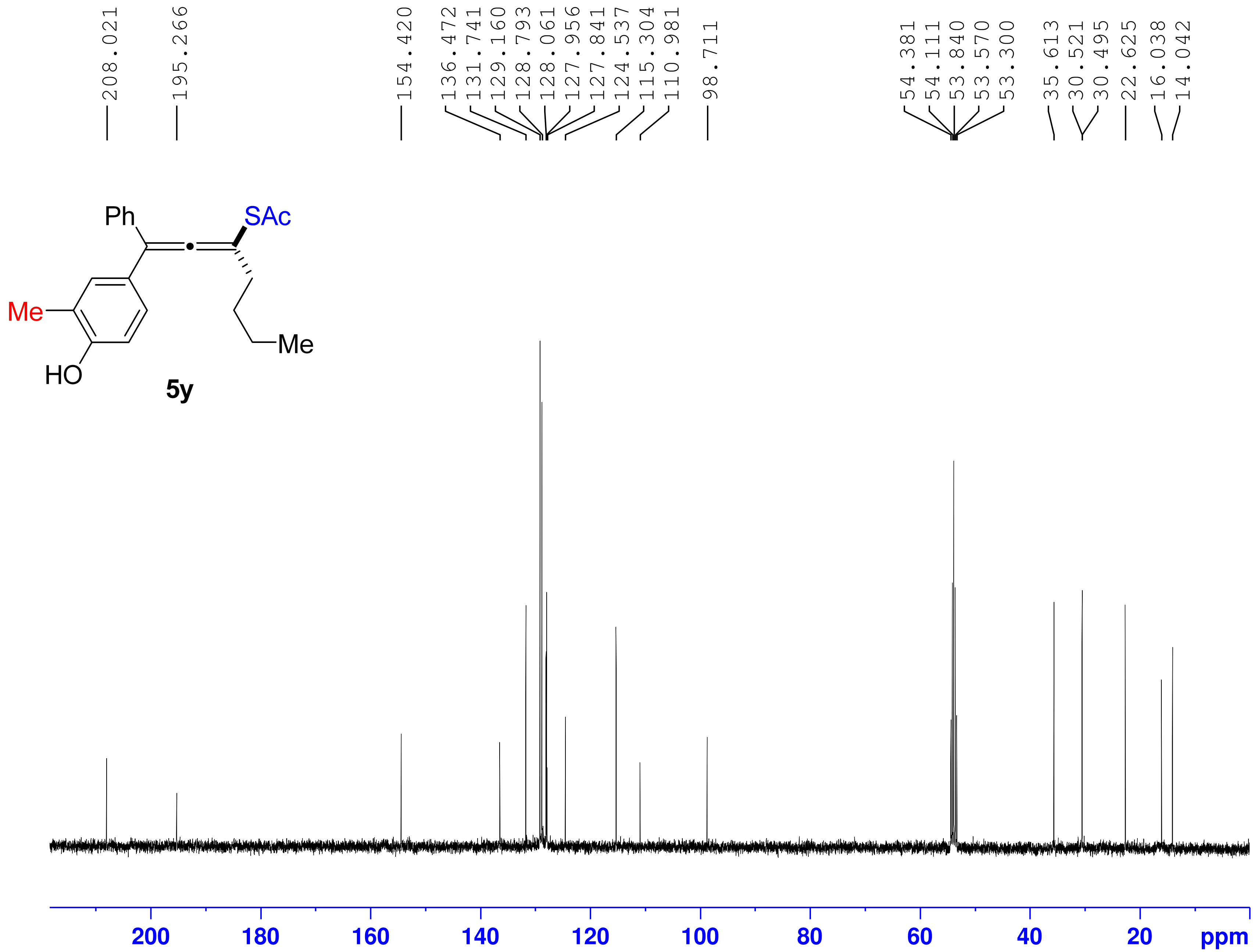

Current Data Parameters
NAME qdy-20140-1 C
EXPNO 1
PROCNO 1

F2 - Acquisition Parameters
Date_ 20160508
Time 21.39
INSTRUM spect
PROBHD 5 mm PABBO BB/
PULPROG zgpg30
TD 65536
SOLVENT CD2Cl2
NS 149
DS 0
SWH 24038.461 Hz
FIDRES 0.366798 Hz
AQ 1.3631488 sec
RG 196.92
DW 20.800 usec
DE 6.50 usec
TE 299.6 K
D1 2.00000000 sec
D11 0.03000000 sec
TD0 1

======== CHANNEL f1 ========
SFO1 100.6228298 MHz
NUC1 13C
P1 9.70 usec
PLW1 46.98899841 W

======== CHANNEL f2 ========
SFO2 400.1316005 MHz
NUC2 1H
CPDPRG[2 waltz16
PCPD2 90.00 usec
PLW2 11.99499989 W
PLW12 0.34213999 W
PLW13 0.27713001 W

F2 - Processing parameters
SI 32768
SF 100.6127262 MHz
WDW EM
SSB 0
LB 1.00 Hz
GB 0
PC 1.40

Supplementary Figure 156. ^{13}C NMR spectrum for 5y

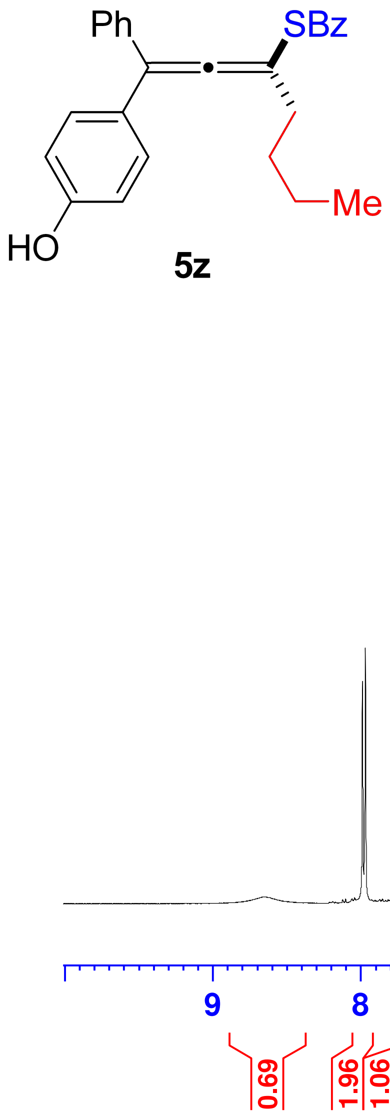

5z

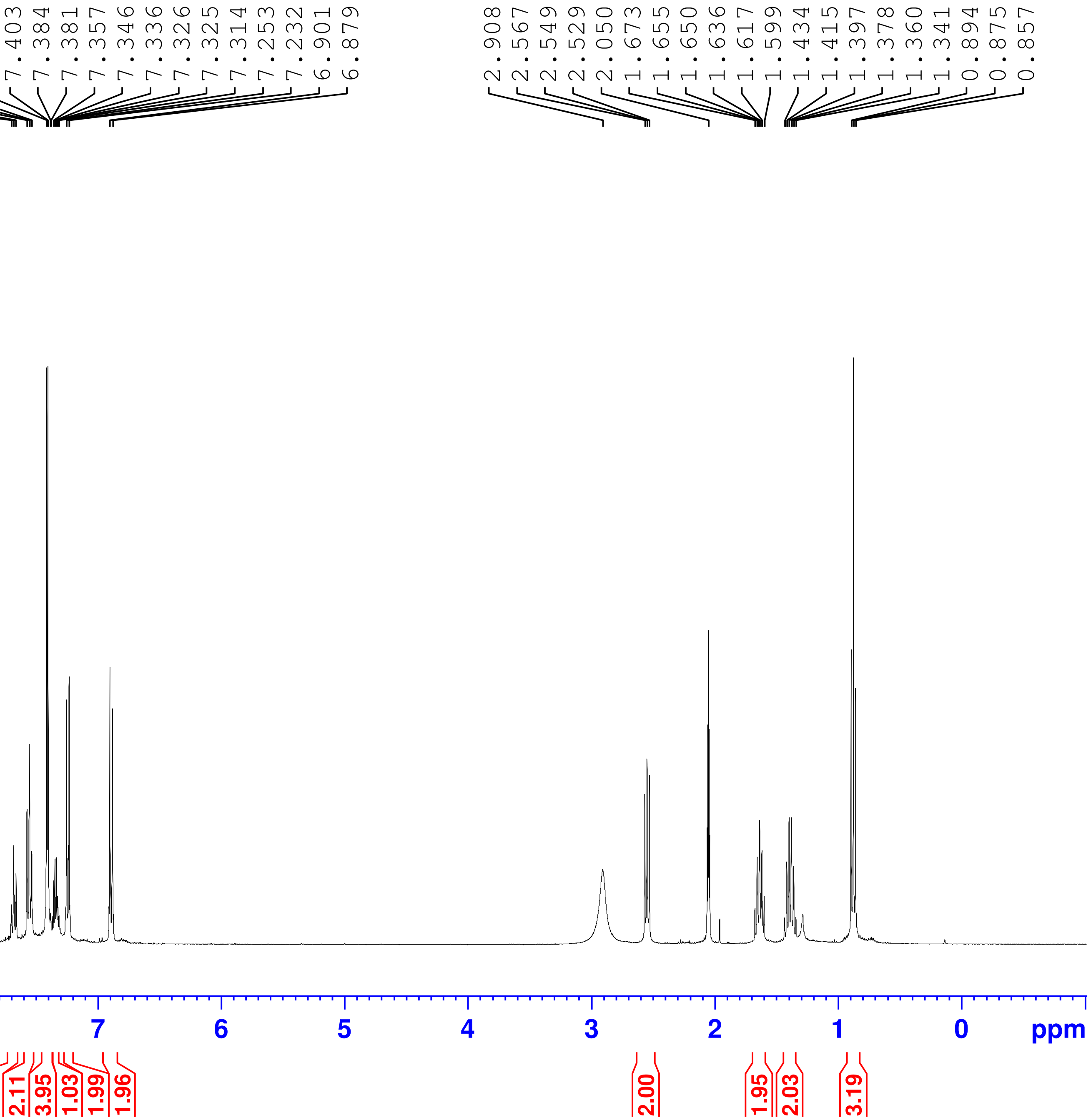


```
Current Data Parameters
NAME          qdy-30054 H
EXPNO                   2
PROCNO                  1

F2 - Acquisition Parameters
Date_            20160816
Time                13.36
INSTRUM             spect
PROBHD     5 mm PABBO BB/
PULPROG              zg30
TD                  65536
SOLVENT           Acetone
NS                      6
DS                      0
SWH              8012.820 Hz
FIDRES           0.122266 Hz
AQ              4.0894465 sec
RG                  82.92
DW                 62.400 usec
DE                   6.50 usec
TE                  298.9 K
D1             1.00000000 sec
TD0                     1

======== CHANNEL f1 ========
SFO1          400.1324710 MHz
NUC1                   1H
P1                  14.50 usec
PLW1          11.99499989 W

F2 - Processing parameters
SI                  65536
SF            400.1300067 MHz
WDW                    EM
SSB         0
LB                   0.30 Hz
GB          0
PC                   1.00
```

Supplementary Figure 157. ¹H NMR spectrum for 5z

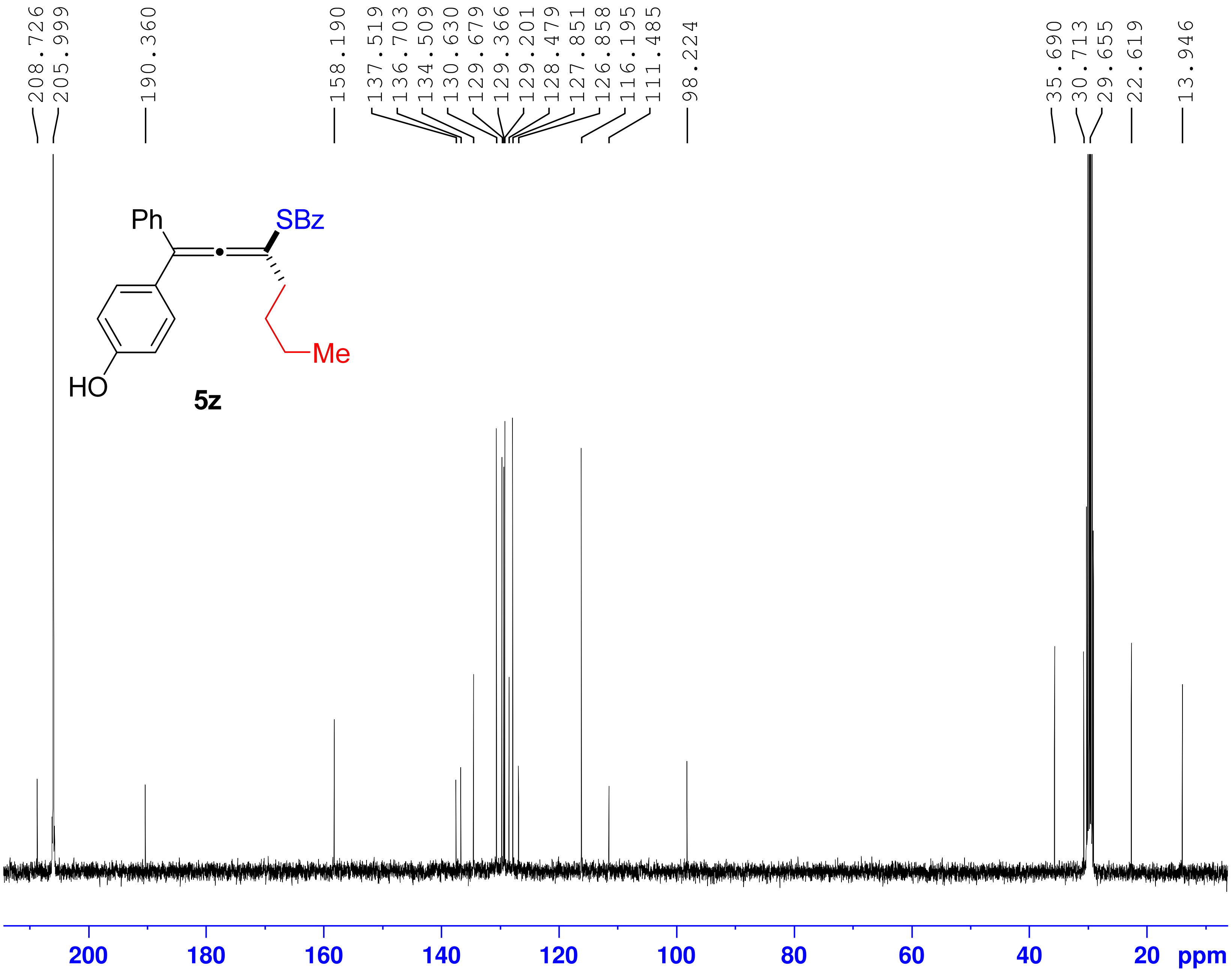


```
Current Data Parameters
NAME          qdy-30054 C
EXPNO                   1
PROCNO                  1

F2 - Acquisition Parameters
Date_            20160815
Time                21.56
INSTRUM             spect
PROBHD    5 mm PABBO BB/
PULPROG           zgpg30
TD                 65536
SOLVENT           Acetone
NS                    244
DS                      0
SWH             24038.461 Hz
FIDRES           0.366798 Hz
AQ              1.3631488 sec
RG                 196.92
DW                 20.800 usec
DE                   6.50 usec
TE                  300.2 K
D1             2.00000000 sec
D11            0.03000000 sec
TD0                     1

======== CHANNEL f1 ========
SFO1         100.6228298 MHz
NUC1                 13C
P1                   9.70 usec
PLW1          46.98899841 W

======== CHANNEL f2 ========
SFO2         400.1316005 MHz
NUC2                  1H
CPDPRG[2            waltz16
PCPD2               90.00 usec
PLW2          11.99499989 W
PLW12          0.34213999 W
PLW13          0.27713001 W

F2 - Processing parameters
SI                  32768
SF           100.6126978 MHz
WDW                    EM
SSB       0
LB                   1.00 Hz
GB        0
PC                   1.40
```

Supplementary Figure 158. ^{13}C NMR spectrum for 5z

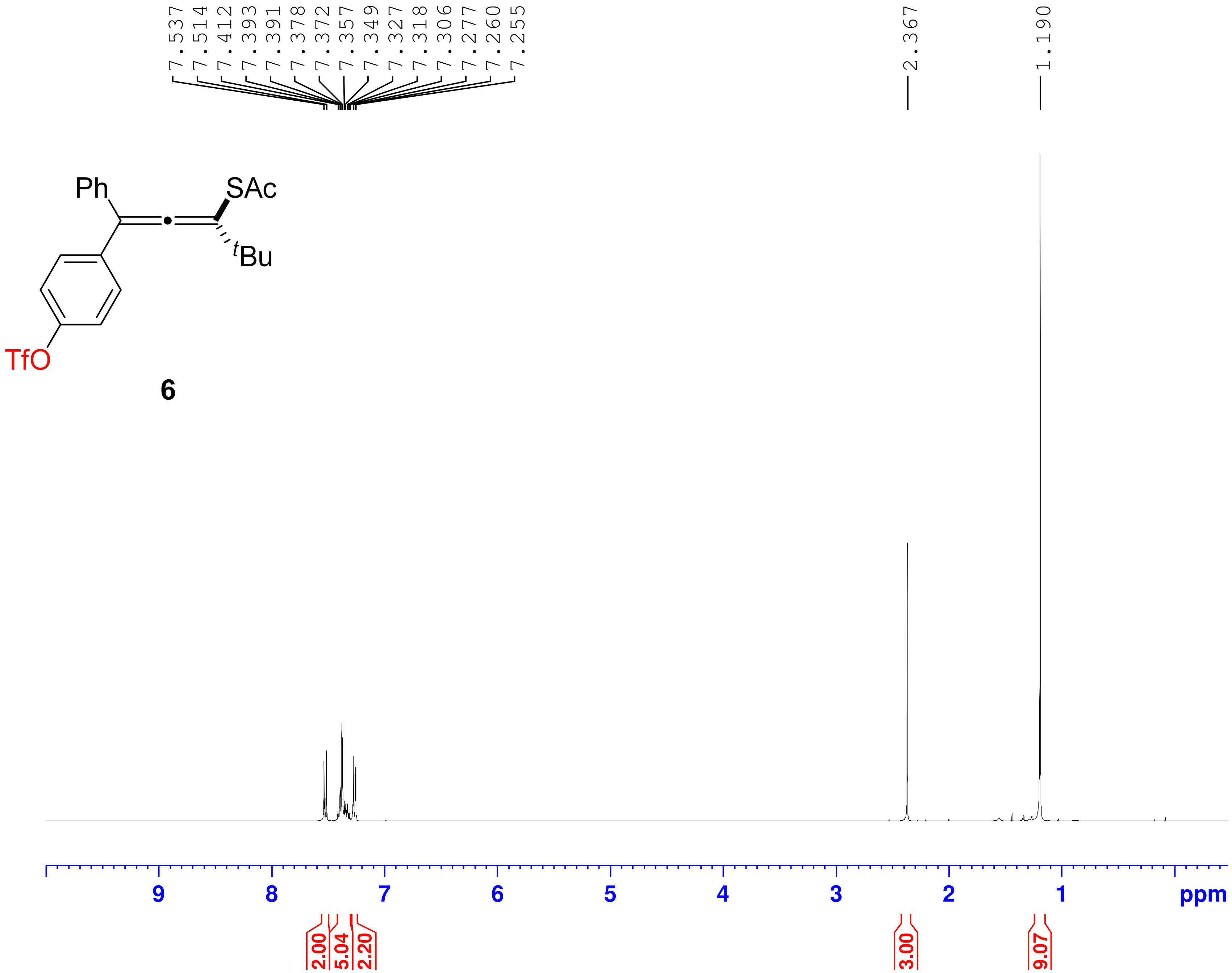

Current Data Parameters
NAME qdy-20181-2 H
EXPNO 1
PROCNO 1

F2 - Acquisition Parameters
Date_ 20160625
Time 14.08
INSTRUM spect
PROBHD 5 mm PABBO BB/
PULPROG zg30
TD 65536
SOLVENT CDCl3
NS 4
DS 0
SWH 8012.820 Hz
FIDRES 0.122266 Hz
AQ 4.0894465 sec
RG 70.97
DW 62.400 usec
DE 6.50 usec
TE 298.8 K
D1 1.00000000 sec
TD0 1

======== CHANNEL f1 ========
SFO1 400.1324710 MHz
NUC1 1H
P1 14.50 usec
PLW1 11.99499989 W

F2 - Processing parameters
SI 65536
SF 400.1300094 MHz
WDW EM
SSB 0
LB 0.30 Hz
GB 0
PC 1.00

Supplementary Figure 159. ^{1}H NMR spectrum for 6

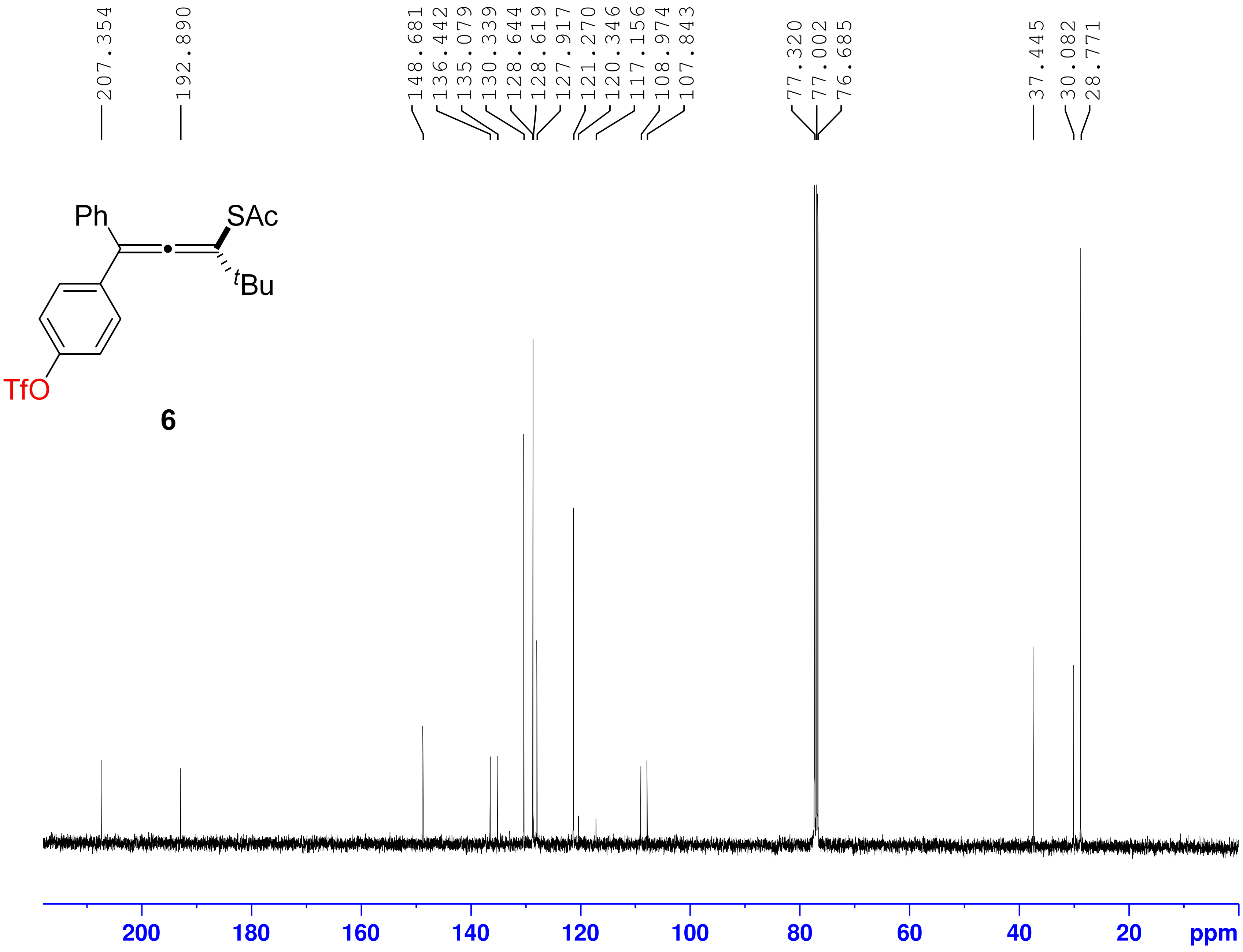


```
Current Data Parameters
NAME        qdy-20181-2 C
EXPNO                   2
PROCNO                  1

F2 - Acquisition Parameters
Date_            20160625
Time                16.26
INSTRUM             spect
PROBHD   5 mm PABBO BB/
PULPROG           zgpg30
TD                 65536
SOLVENT            CDCl3
NS                   170
DS                     0
SWH            24038.461 Hz
FIDRES          0.366798 Hz
AQ             1.3631488 sec
RG                196.92
DW                20.800 usec
DE                  6.50 usec
TE                 299.9 K
D1            2.00000000 sec
D11           0.03000000 sec
TD0                    1

======== CHANNEL f1 ========
SFO1         100.6228298 MHz
NUC1                 13C
P1                  9.70 usec
PLW1         46.98899841 W

======== CHANNEL f2 ========
SFO2         400.1316005 MHz
NUC2                  1H
CPDPRG[2         waltz16
PCPD2              90.00 usec
PLW2         11.99499989 W
PLW12         0.34213999 W
PLW13         0.27713001 W

F2 - Processing parameters
SI                 32768
SF           100.6127707 MHz
WDW                   EM
SSB      0
LB                  1.00 Hz
GB       0
PC                  1.40
```

Supplementary Figure 160. ^{13}C NMR spectrum for 6

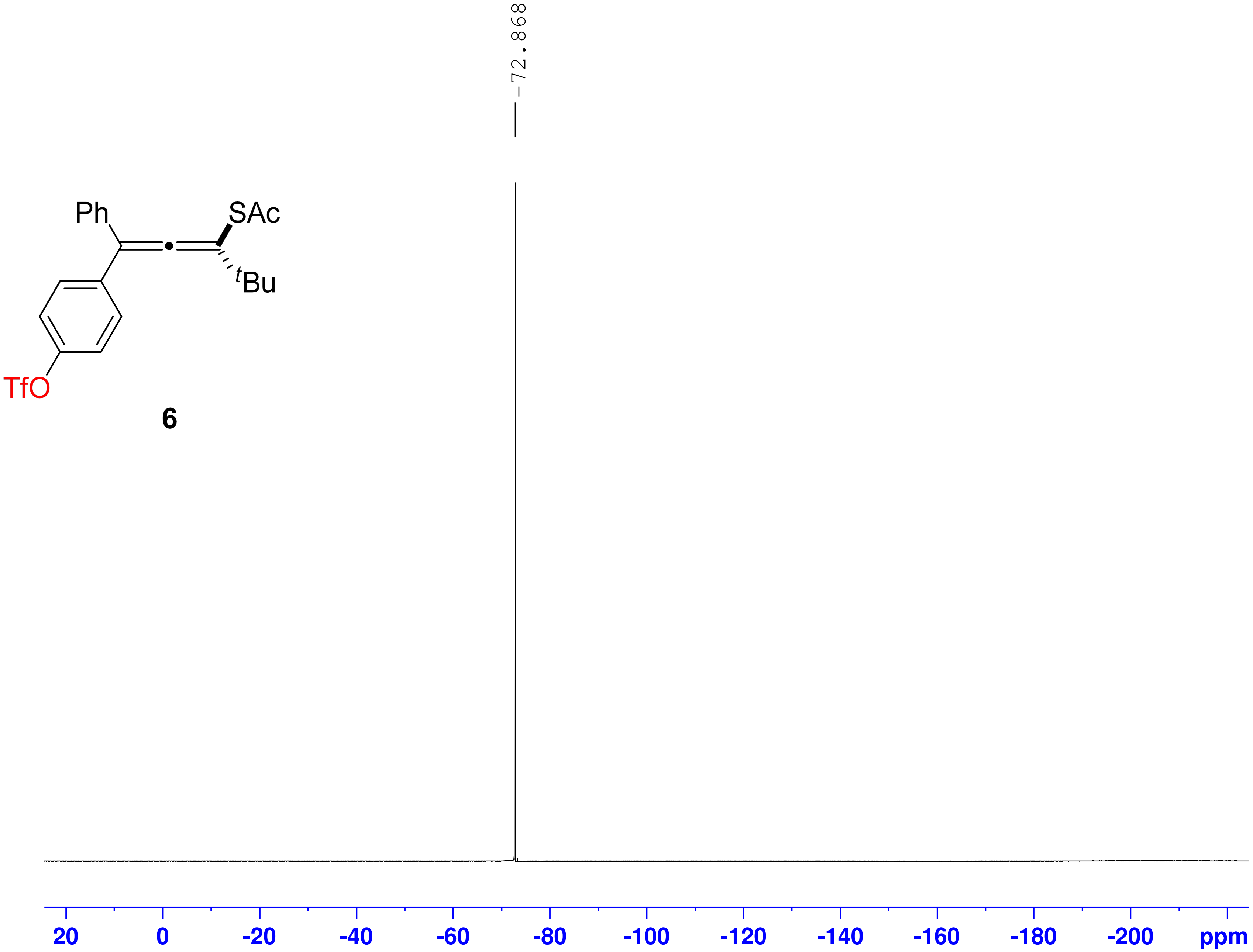


```
Current Data Parameters
NAME         qdy-20181-2 F
EXPNO                    1
PROCNO                   1

F2 - Acquisition Parameters
Date_             20160625
Time                 16.32
INSTRUM              spect
PROBHD     5 mm PABBO BB/
PULPROG            zgpg30
TD                   65536
SOLVENT              CDCl3
NS                       4
DS                       0
SWH            93750.000 Hz
FIDRES          1.430511 Hz
AQ             0.3495253 sec
RG                  196.92
DW                   5.333 usec
DE                    6.50 usec
TE                   299.5 K
D1            2.00000000 sec
D11           0.03000000 sec
TD0                      1

======== CHANNEL f1 ========
SFO1         376.4607162 MHz
NUC1                   19F
P1                   14.70 usec
PLW1         15.99600029 W

======== CHANNEL f2 ========
SFO2         400.1316005 MHz
NUC2                    1H
CPDPRG[2           waltz16
PCPD2                90.00 usec
PLW2         11.99499989 W
PLW12         0.34213999 W
PLW13         0.27713001 W

F2 - Processing parameters
SI                   32768
SF           376.4983660 MHz
WDW                     EM
SSB      0
LB                    1.00 Hz
GB       0
PC                    1.40
```

Supplementary Figure 161. ^{19}F NMR spectrum for 6

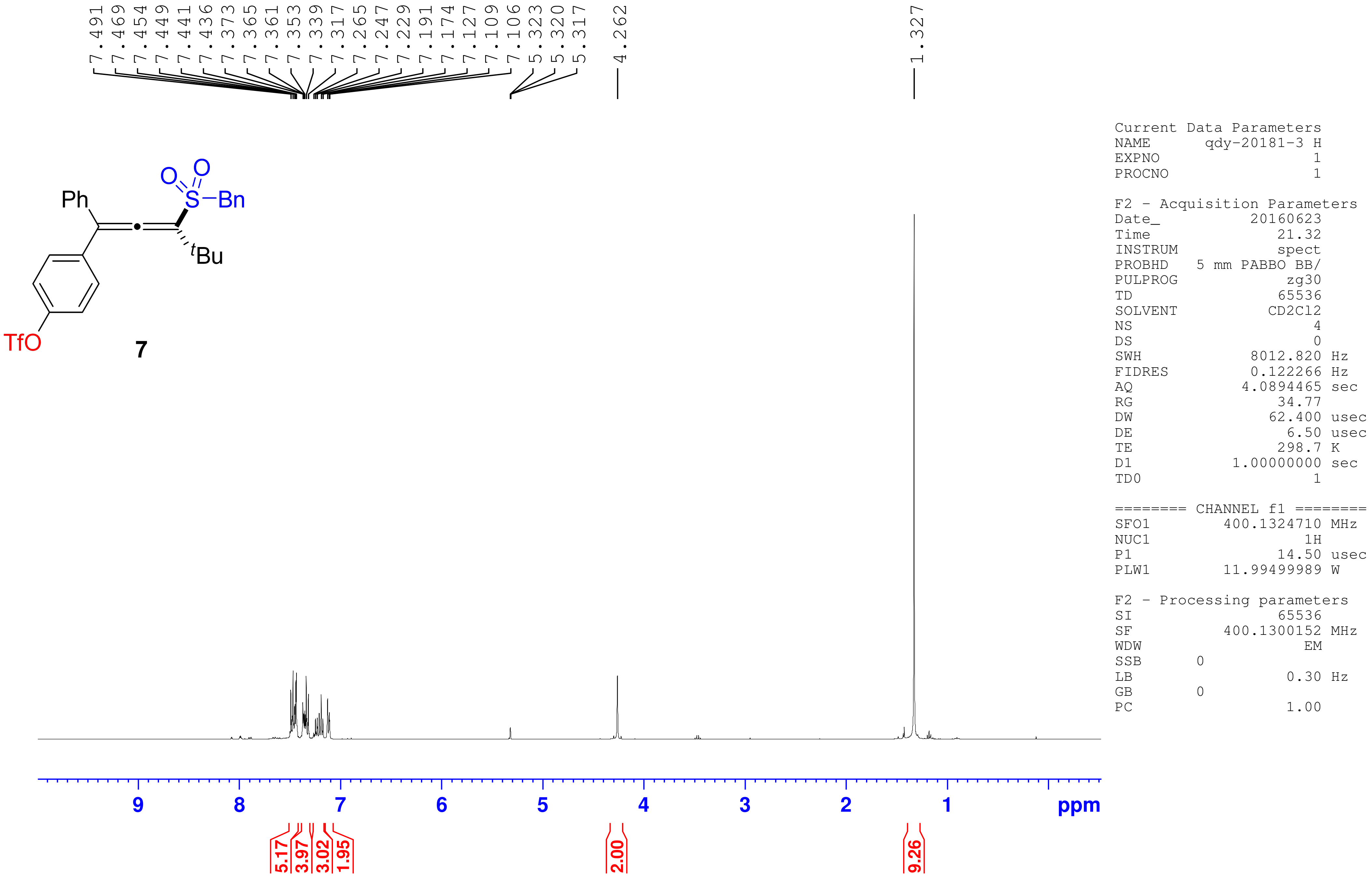

Supplementary Figure 162. ^{1}H NMR spectrum for 7

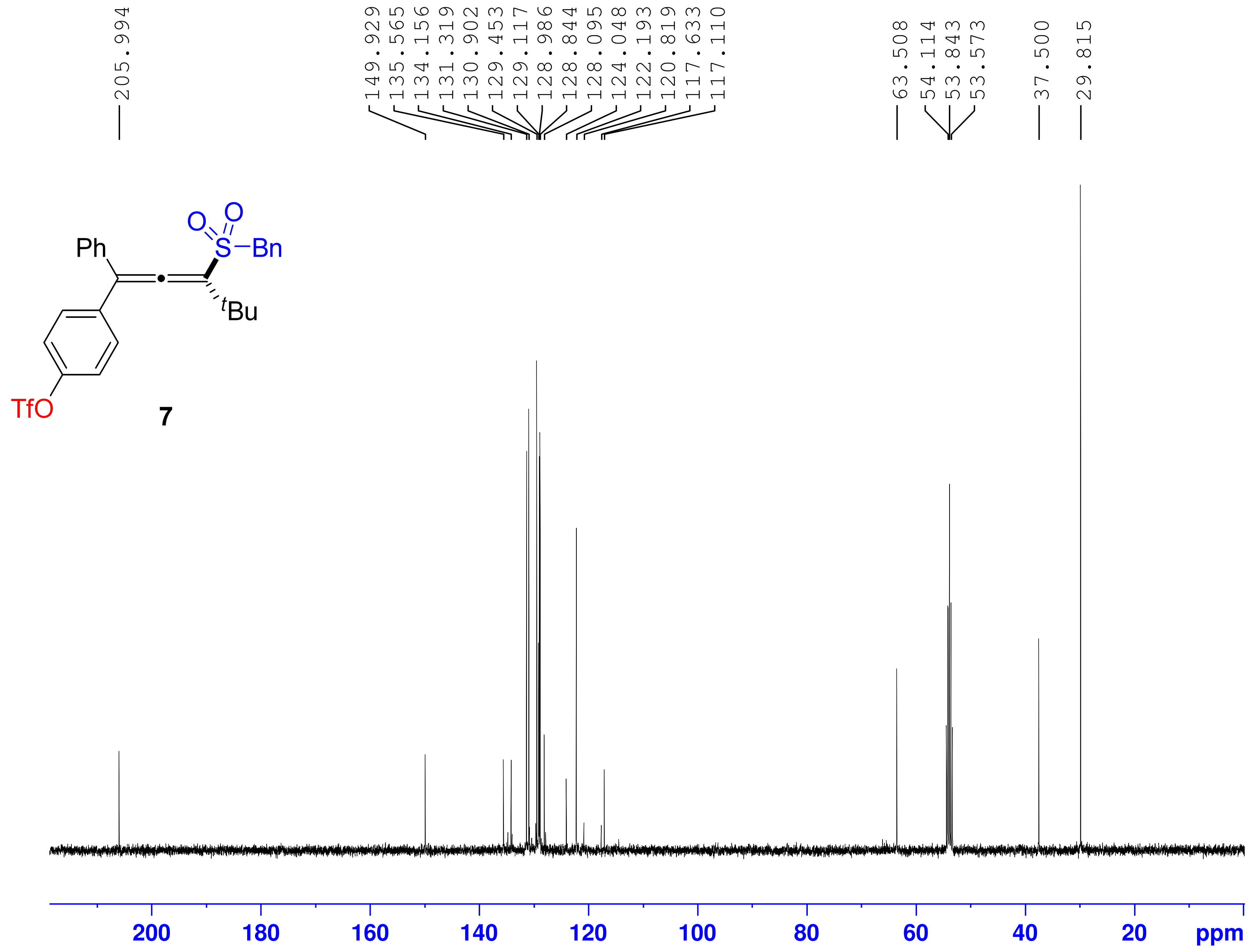

Current Data Parameters
NAME qdy-20181-3 C
EXPNO 1
PROCNO 1

F2 - Acquisition Parameters
Date_ 20160623
Time 21.35
INSTRUM spect
PROBHD 5 mm PABBO BB/
PULPROG zgpg30
TD 65536
SOLVENT CD2Cl2
NS 82
DS 0
SWH 24038.461 Hz
FIDRES 0.366798 Hz
AQ 1.3631488 sec
RG 196.92
DW 20.800 usec
DE 6.50 usec
TE 299.6 K
D1 2.00000000 sec
D11 0.03000000 sec
TD0 1

======== CHANNEL f1 ========
SFO1 100.6228298 MHz
NUC1 13C
P1 9.70 usec
PLW1 46.98899841 W

======== CHANNEL f2 ========
SFO2 400.1316005 MHz
NUC2 1H
CPDPRG[2 waltz16
PCPD2 90.00 usec
PLW2 11.99499989 W
PLW12 0.34213999 W
PLW13 0.27713001 W

F2 - Processing parameters
SI 32768
SF 100.6127255 MHz
WDW EM
SSB 0
LB 1.00 Hz
GB 0
PC 1.40

Supplementary Figure 163. ^{13}C NMR spectrum for 7

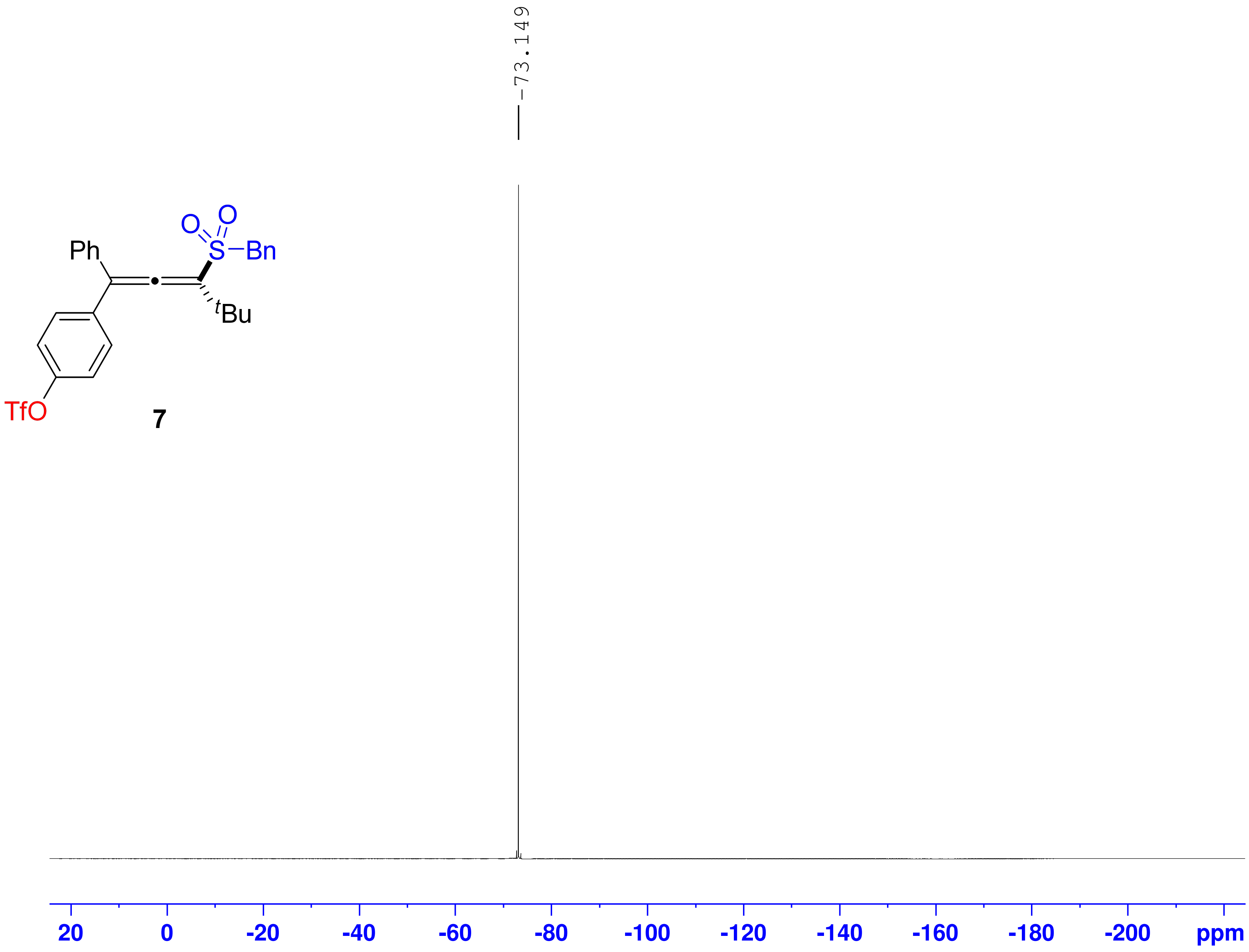


```
Current Data Parameters
NAME        qdy-20181-3 F
EXPNO                   1
PROCNO                  1

F2 - Acquisition Parameters
Date_            20160623
Time                21.39
INSTRUM             spect
PROBHD     5 mm PABBO BB/
PULPROG           zgpg30
TD                 65536
SOLVENT           CD2Cl2
NS                      4
DS                      0
SWH             93750.000 Hz
FIDRES           1.430511 Hz
AQ              0.3495253 sec
RG                 196.92
DW                  5.333 usec
DE                   6.50 usec
TE                  299.1 K
D1             2.00000000 sec
D11            0.03000000 sec
TD0                     1

======== CHANNEL f1 ========
SFO1          376.4607162 MHz
NUC1                  19F
P1                  14.70 usec
PLW1          15.99600029 W

======== CHANNEL f2 ========
SFO2          400.1316005 MHz
NUC2                   1H
CPDPRG[2          waltz16
PCPD2               90.00 usec
PLW2          11.99499989 W
PLW12          0.34213999 W
PLW13          0.27713001 W

F2 - Processing parameters
SI                  32768
SF            376.4983660 MHz
WDW                    EM
SSB      0
LB                   1.00 Hz
GB       0
PC                   1.40
```

Supplementary Figure 164. ^{19}F NMR spectrum for 7

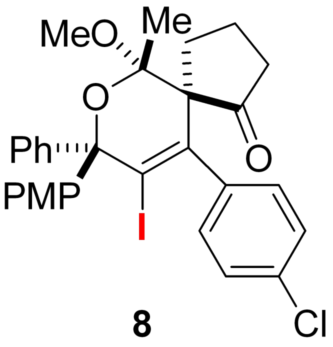

8

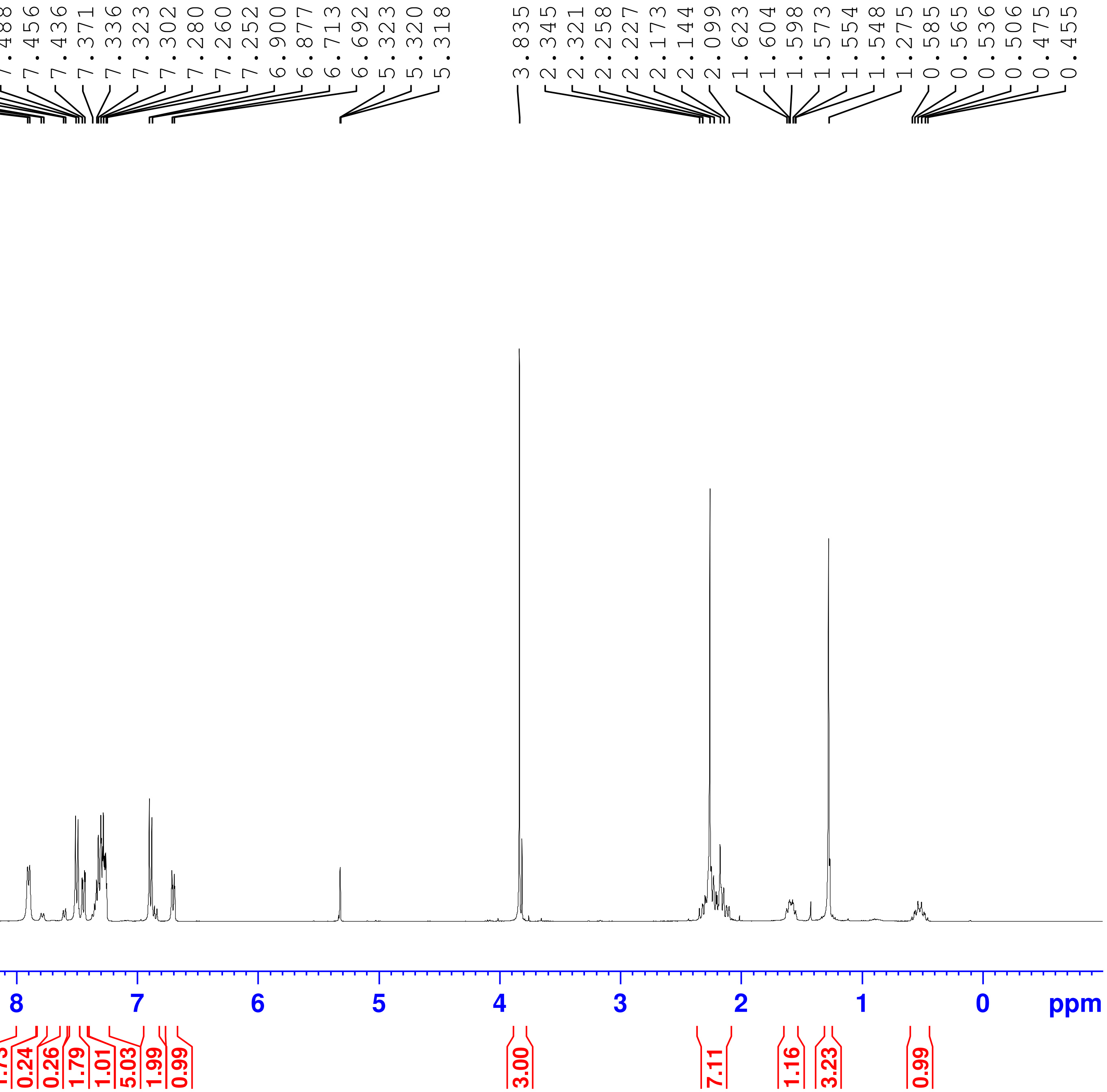


```
Current Data Parameters
NAME       qdy-40038-2 H
EXPNO                  2
PROCNO                 1

F2 - Acquisition Parameters
Date_           20170114
Time               20.10
INSTRUM            spect
PROBHD    5 mm PABBO BB/
PULPROG             zg30
TD                 65536
SOLVENT           CD2Cl2
NS                     4
DS                     0
SWH           8012.820 Hz
FIDRES        0.122266 Hz
AQ           4.0894465 sec
RG                 49.32
DW              62.400 usec
DE                6.50 usec
TE               296.3 K
D1          1.00000000 sec
TD0                    1

======== CHANNEL f1 ========
SFO1        400.1324710 MHz
NUC1                 1H
P1                14.50 usec
PLW1        11.99499989 W

F2 - Processing parameters
SI                 65536
SF          400.1300157 MHz
WDW                   EM
SSB       0
LB                0.30 Hz
GB        0
PC                 1.00
```

Supplementary Figure 165. ^{1}H NMR spectrum for 8

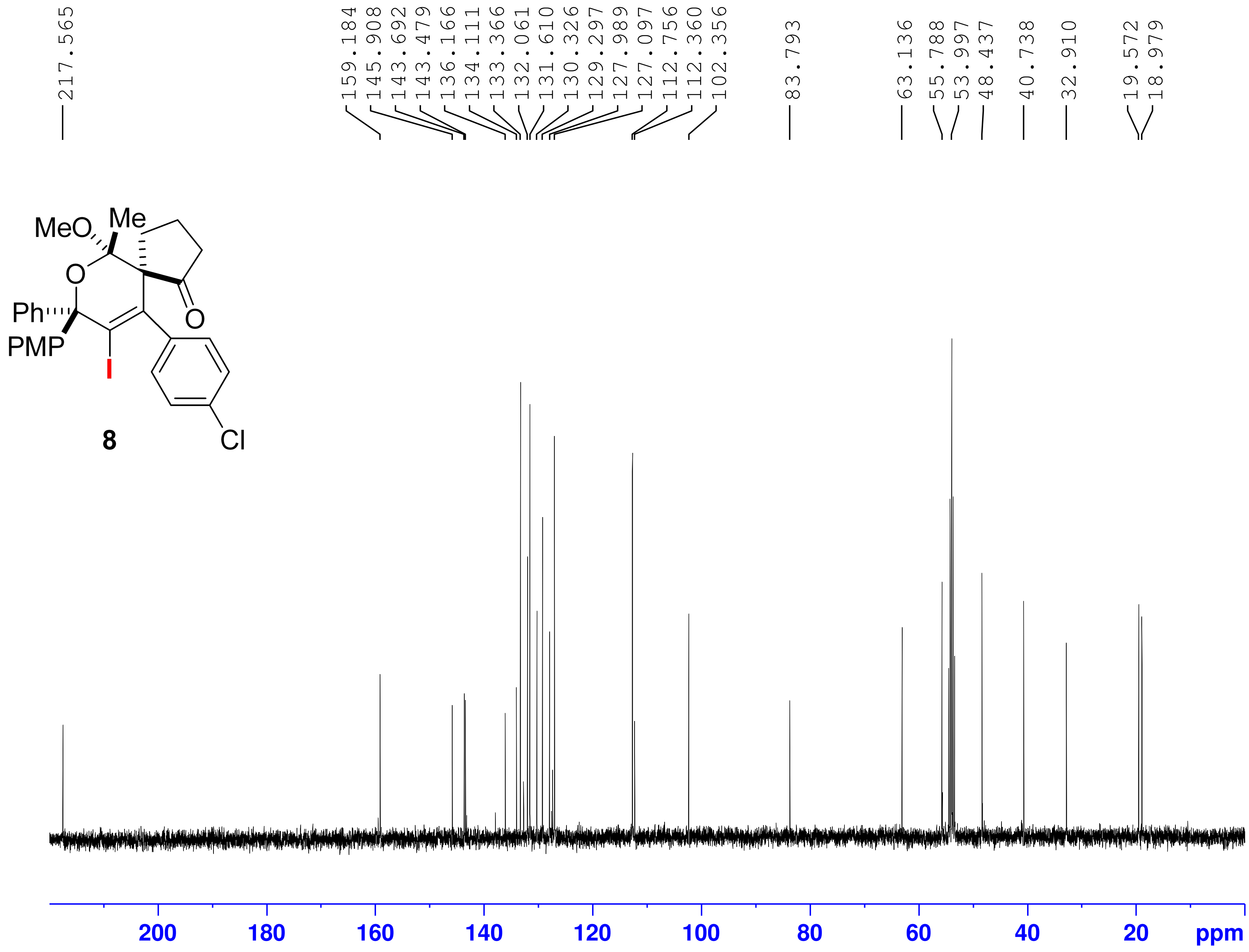


```
Current Data Parameters
NAME       qdy-40038-1 C
EXPNO                  1
PROCNO                 1

F2 - Acquisition Parameters
Date_           20170115
Time               17.36
INSTRUM            spect
PROBHD    5 mm PABBO BB/
PULPROG          zgpg30
TD                 65536
SOLVENT          CD2Cl2
NS                    74
DS                     0
SWH           24038.461 Hz
FIDRES         0.366798 Hz
AQ            1.3631488 sec
RG               196.92
DW               20.800 usec
DE                 6.50 usec
TE                296.9 K
D1           2.00000000 sec
D11          0.03000000 sec
TD0                    1

======== CHANNEL f1 ========
SFO1        100.6228298 MHz
NUC1                13C
P1                 9.70 usec
PLW1        46.98899841 W

======== CHANNEL f2 ========
SFO2        400.1316005 MHz
NUC2                 1H
CPDPRG[2        waltz16
PCPD2             90.00 usec
PLW2        11.99499989 W
PLW12        0.34213999 W
PLW13        0.27713001 W

F2 - Processing parameters
SI                32768
SF          100.6127153 MHz
WDW                  EM
SSB      0
LB                 1.00 Hz
GB       0
PC                 1.40
```

Supplementary Figure 166. ^{13}C NMR spectrum for 8

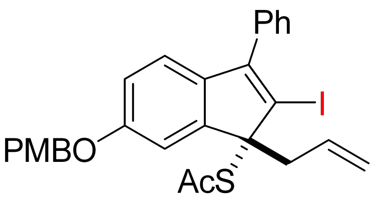

9

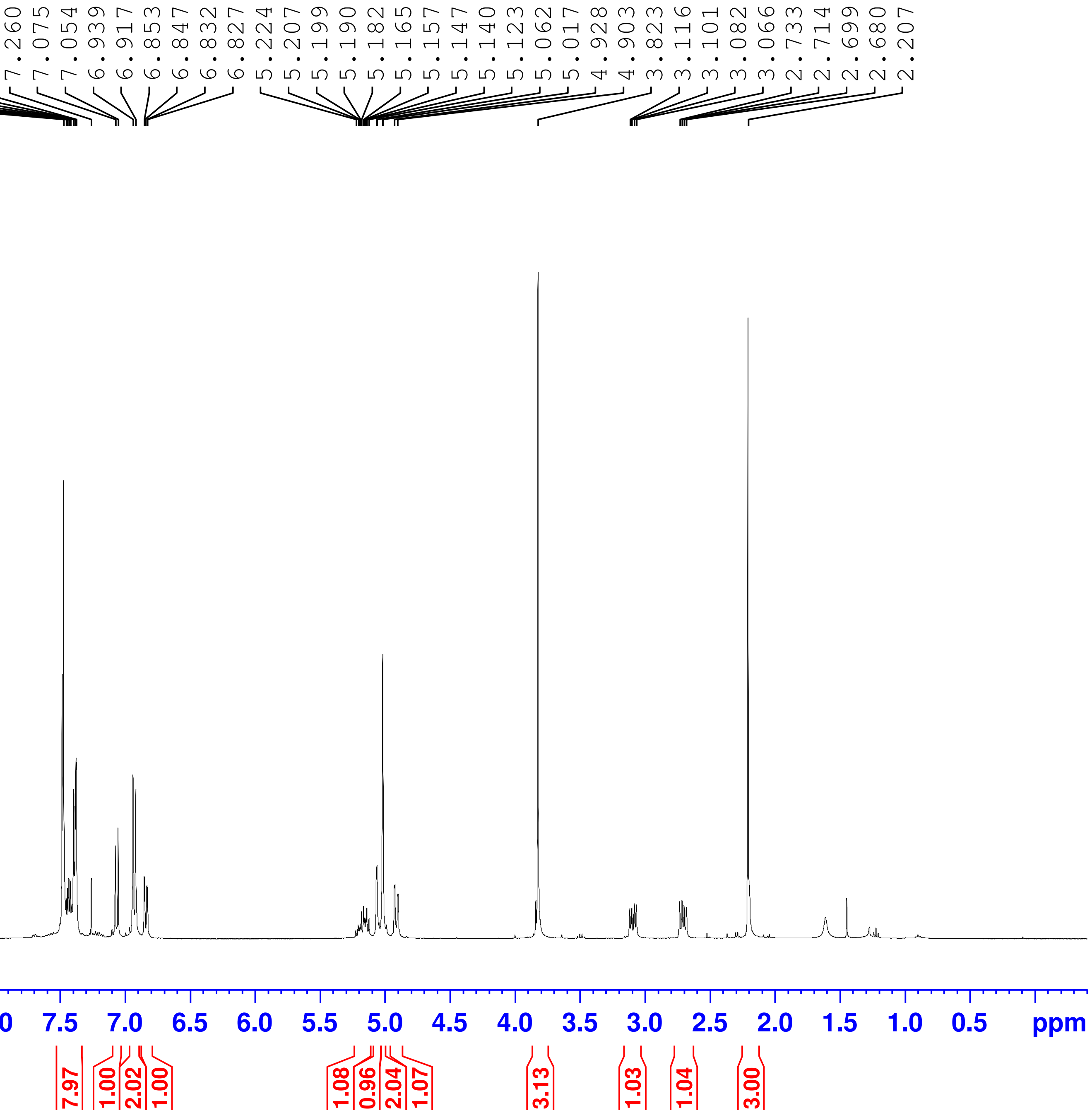

Current Data Parameters
NAME qdy-20152-2 H
EXPNO 1
PROCNO 1

F2 - Acquisition Parameters
Date_ 20160519
Time 19.08
INSTRUM spect
PROBHD 5 mm PABBO BB/
PULPROG zg30
TD 65536
SOLVENT CDCl3
NS 4
DS 0
SWH 8012.820 Hz
FIDRES 0.122266 Hz
AQ 4.0894465 sec
RG 70.97
DW 62.400 usec
DE 6.50 usec
TE 297.9 K
D1 1.00000000 sec
TD0 1

======== CHANNEL f1 ========
SFO1 400.1324710 MHz
NUC1 1H
P1 14.50 usec
PLW1 11.99499989 W

F2 - Processing parameters
SI 65536
SF 400.1300092 MHz
WDW EM
SSB 0
LB 0.30 Hz
GB 0
PC 1.00

Supplementary Figure 167. ^1H NMR spectrum for 9

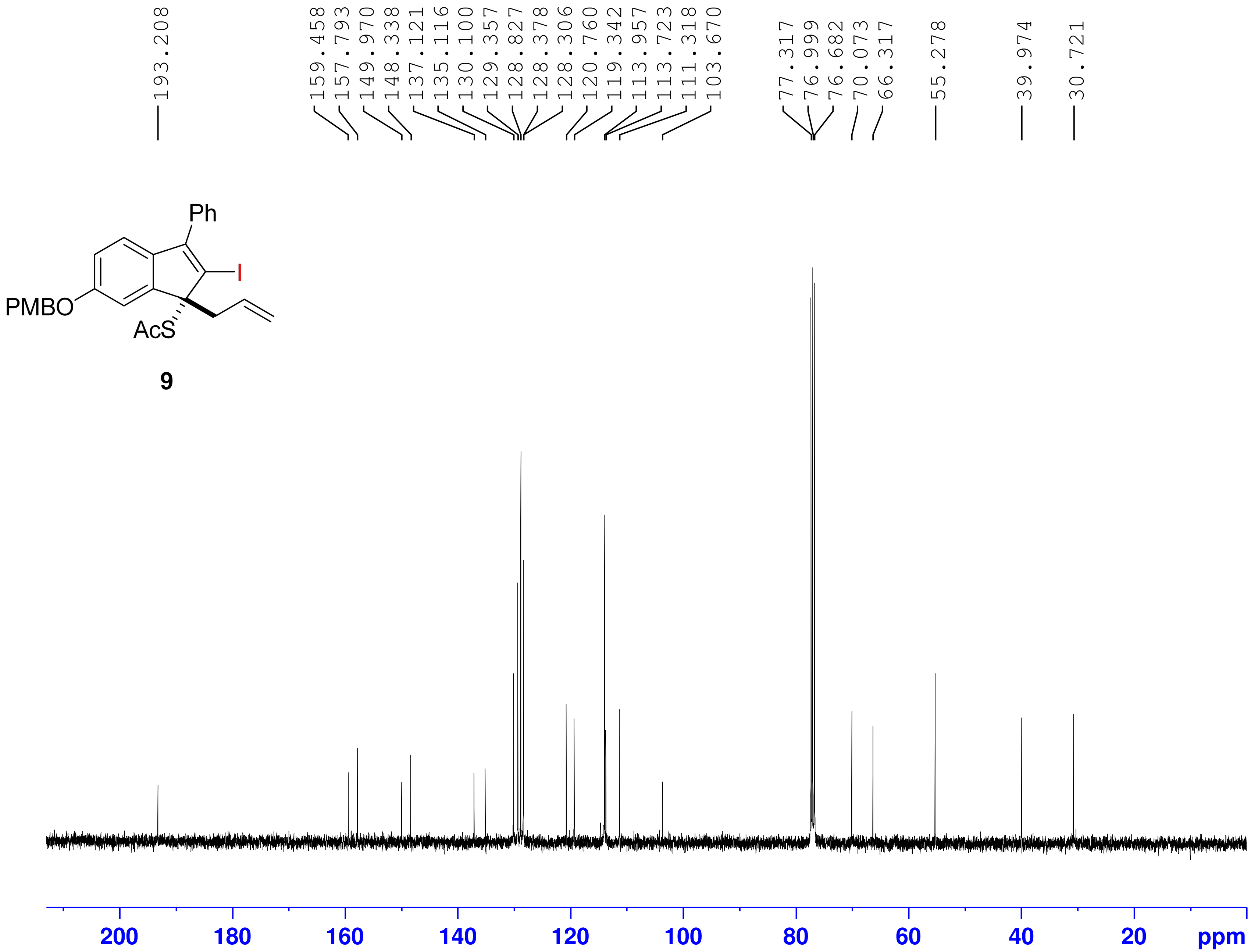


```
Current Data Parameters
NAME        qdy-20152-2 C
EXPNO                   1
PROCNO                  1

F2 - Acquisition Parameters
Date_            20160519
Time                19.20
INSTRUM             spect
PROBHD    5 mm PABBO BB/
PULPROG           zgpg30
TD                  65536
SOLVENT             CDCl3
NS                    156
DS                      0
SWH             24038.461 Hz
FIDRES           0.366798 Hz
AQ              1.3631488 sec
RG                 196.92
DW                 20.800 usec
DE                   6.50 usec
TE                  299.2 K
D1             2.00000000 sec
D11            0.03000000 sec
TD0                     1

======== CHANNEL f1 ========
SFO1          100.6228298 MHz
NUC1                  13C
P1                   9.70 usec
PLW1          46.98899841 W

======== CHANNEL f2 ========
SFO2          400.1316005 MHz
NUC2                   1H
CPDPRG[2          waltz16
PCPD2               90.00 usec
PLW2          11.99499989 W
PLW12          0.34213999 W
PLW13          0.27713001 W

F2 - Processing parameters
SI                  32768
SF            100.6127744 MHz
WDW                    EM
SSB      0
LB                   1.00 Hz
GB       0
PC                   1.40
```

Supplementary Figure 168. ^{13}C NMR spectrum for 9

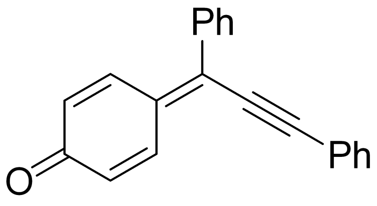

QM

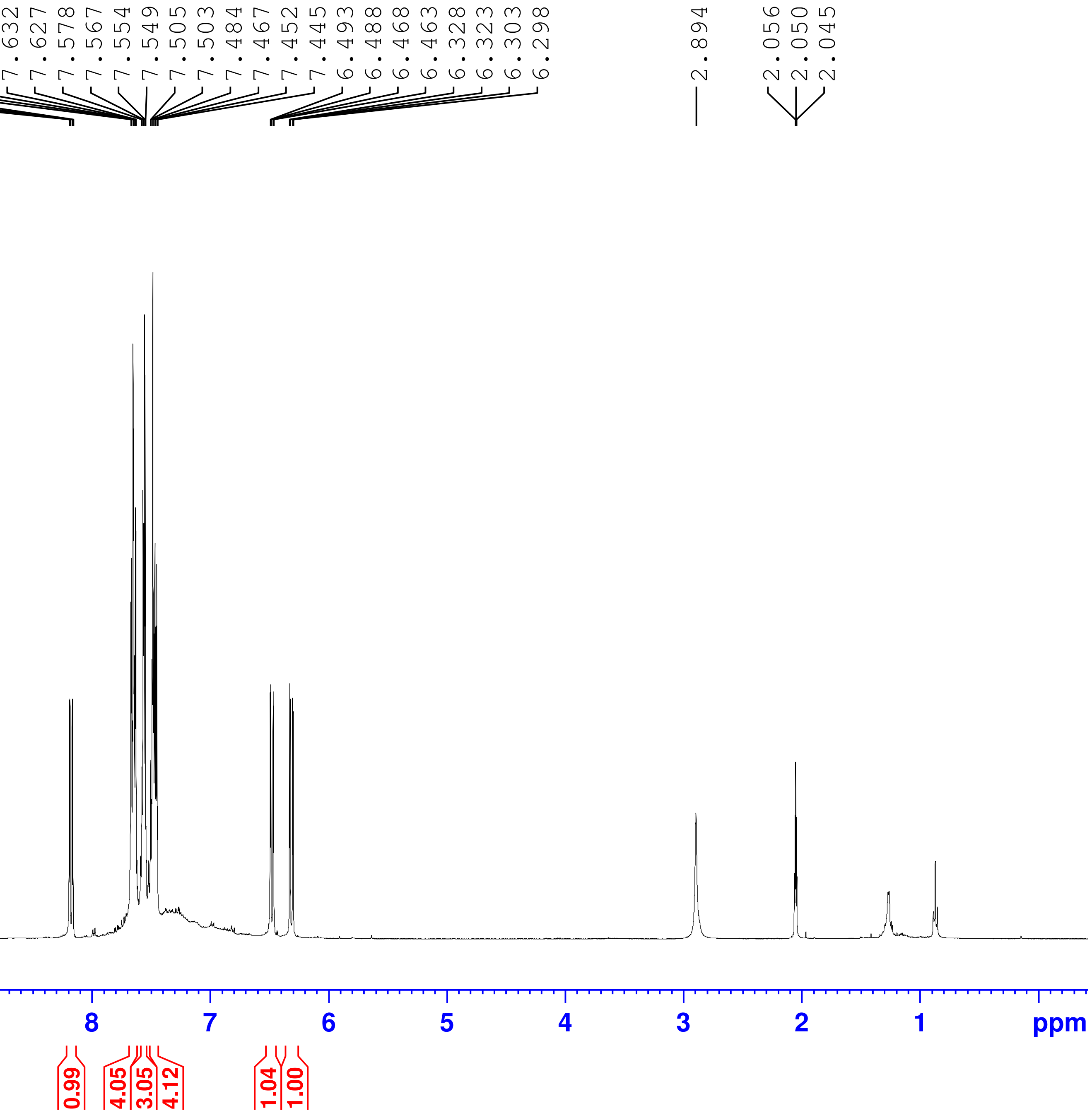

Current Data Parameters
NAME qdy-20167-2 H
EXPNO 2
PROCNO 1

F2 - Acquisition Parameters
Date_ 20160607
Time 15.07
INSTRUM spect
PROBHD 5 mm PABBO BB/
PULPROG zg30
TD 65536
SOLVENT Acetone
NS 8
DS 0
SWH 8012.820 Hz
FIDRES 0.122266 Hz
AQ 4.0894465 sec
RG 54.81
DW 62.400 usec
DE 6.50 usec
TE 296.6 K
D1 1.00000000 sec
TD0 1

======== CHANNEL f1 ========
SFO1 400.1324710 MHz
NUC1 1H
P1 14.50 usec
PLW1 11.99499989 W

F2 - Processing parameters
SI 65536
SF 400.1300066 MHz
WDW EM
SSB 0
LB 0.30 Hz
GB 0
PC 1.00

Supplementary Figure 169. ^{1}H NMR spectrum for QM

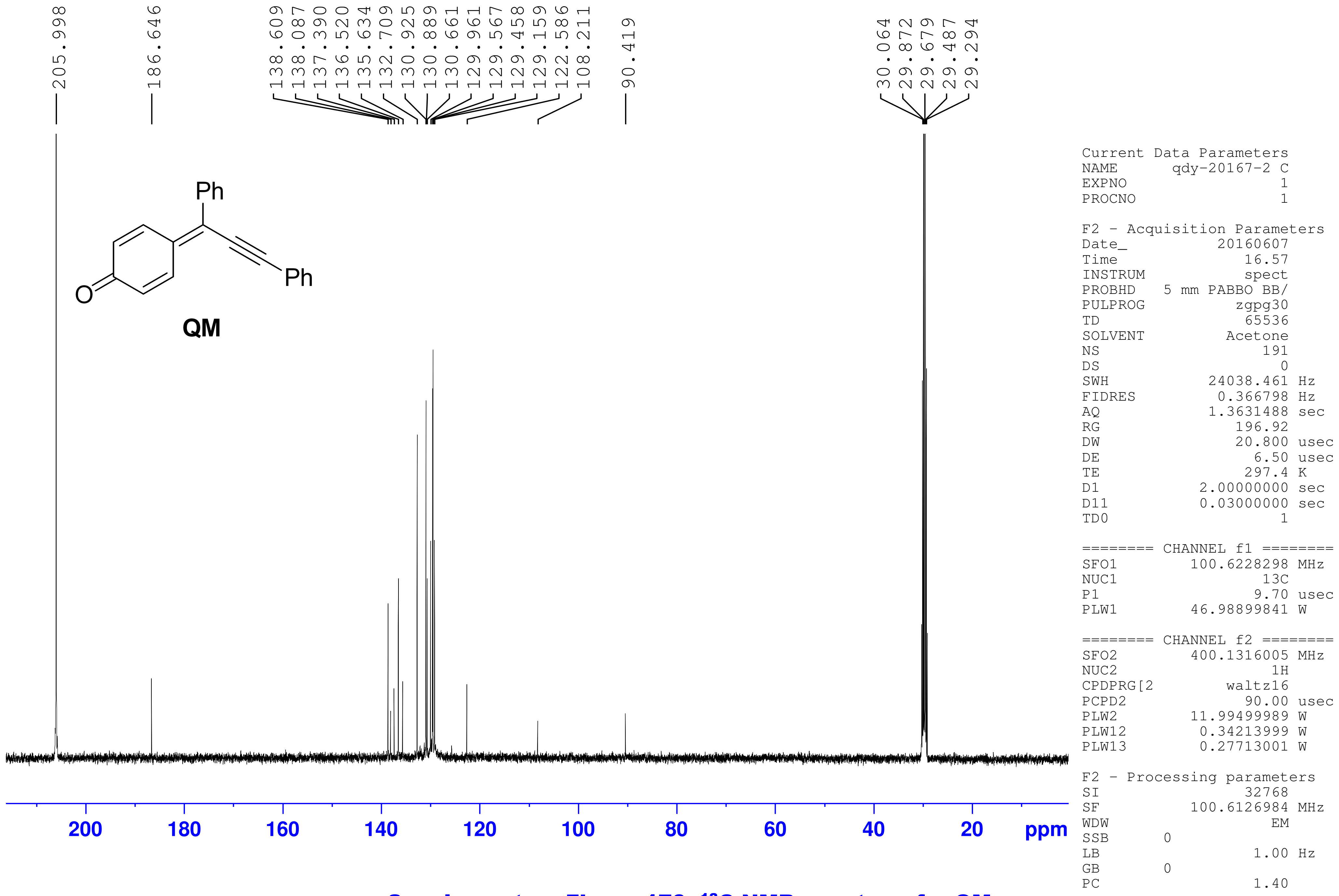

Supplementary Figure 170. ^{13}C NMR spectrum for QM

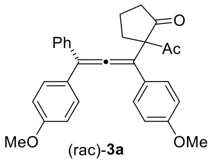

(rac)-**3a**

Acq. Operator : Seq. Line : 22
Acq. Instrument : Instrument 1 Location : Vial 35
Injection Date : 12/10/2016 12:38:52 AM Inj : 1
Inj Volume : 5.000 µl
Acq. Method : C:\CHEM32\1\DATA\QDY 2016-12-09 15-30-48\AD-10-30.M
Last changed : 11/30/2015 12:57:24 PM
Analysis Method : C:\CHEM32\1\METHODS\AD-50-30.M
Last changed : 12/17/2016 10:34:06 PM
(modified after loading)
Additional Info : Peak(s) manually integrated

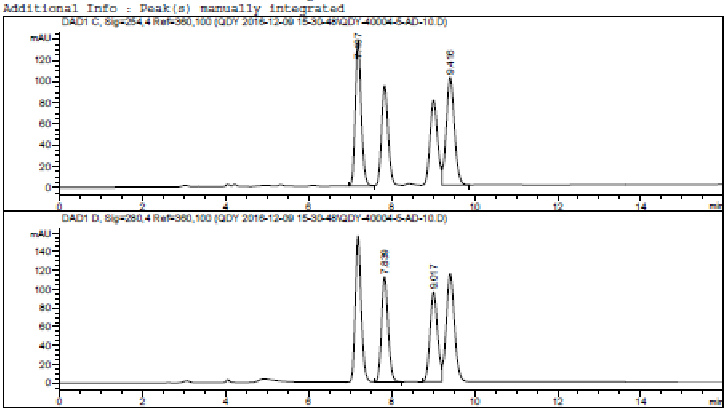

Area Percent Report

Sorted By : Signal
Multiplier : 1.0000
Dilution : 1.0000
Use Multiplier & Dilution Factor with ISTDs

Signal 1: DAD1 C, Sig=254,4 Ref=360,100

Peak #	RetTime [min]	Type	Width [min]	Area [mAU*s]	Height [mAU]	Area %
1	7.197	BB	0.1562	1393.25073	137.51768	49.9470
2	9.416	VB	0.2099	1396.20593	101.89838	50.0530

Totals : 2789.45667 239.41606

Peak #	RetTime [min]	Type	Width [min]	Area [mAU*s]	Height [mAU]	Area %
1	7.839	BB	0.1686	1232.49475	111.78729	50.5441
2	9.017	BV	0.1956	1205.95996	95.34112	49.4559

Totals : 2438.45471 207.12841

*** End of Report ***

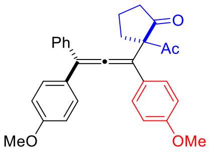

3a

Acq. Operator : Seq. Line : 18
Acq. Instrument : Instrument 1 Location : Vial 31
Injection Date : 12/17/2016 6:13:43 PM Inj : 1
Inj Volume : 5.000 µl
Acq. Method : C:\CHEM32\1\DATA\QDY 2016-12-17 10-09-58\AD-10-25.M
Last changed : 12/17/2016 6:33:07 PM
(modified after loading)
Analysis Method : C:\CHEM32\1\METHODS\AD-50-30.M
Last changed : 12/17/2016 10:21:47 PM
(modified after loading)
Additional Info : Peak(s) manually integrated

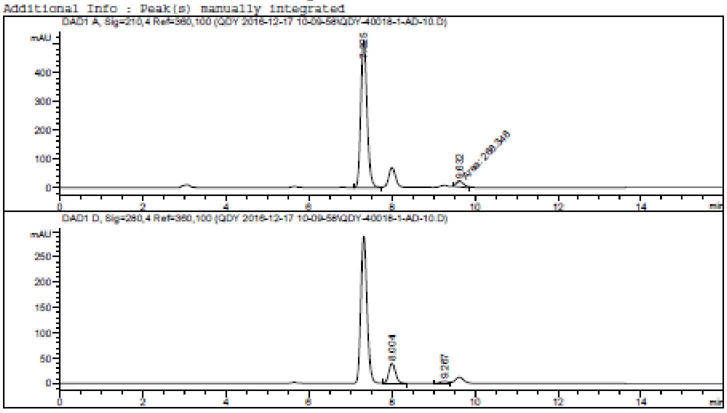

Area Percent Report

Sorted By : Signal
Multiplier : 1.0000
Dilution : 1.0000
Use Multiplier & Dilution Factor with ISTDs

Signal 1: DAD1 A, Sig=210,4 Ref=360,100

Peak #	RetTime [min]	Type	Width [min]	Area [mAU*s]	Height [mAU]	Area %
1	7.325	BV	0.1588	5284.21094	510.25525	95.1671
2	9.632	MM	0.2169	268.34750	20.61820	4.8329

Totals : 5552.55844 530.87345

Signal 2: DAD1 D, Sig=280,4 Ref=360,100

Peak #	RetTime [min]	Type	Width [min]	Area [mAU*s]	Height [mAU]	Area %
1	8.004	BB	0.1768	453.72427	39.86567	89.5725
2	9.267	BV	0.1940	52.81998	4.21946	10.4275

Totals : 506.54425 44.08513

*** End of Report ***

Supplementary Figure 171. HPLC spectrum for 3a

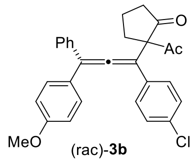

(rac)-**3b**

Acq. Operator : Seq. Line : 8
Acq. Instrument : Instrument 1 Location : Vial 32
Injection Date : 12/11/2016 11:13:09 PM Inj : 1
Inj Volume : 5.000 µl
Acq. Method : C:\CHEM32\1\DATA\QDY 2016-12-11 20-39-38\AD-05-20.M
Last changed : 10/14/2015 3:51:32 PM
Analysis Method : C:\CHEM32\1\METHODS\AD-50-30.M
Last changed : 12/17/2016 10:34:06 PM
(modified after loading)
Additional Info : Peak(s) manually integrated

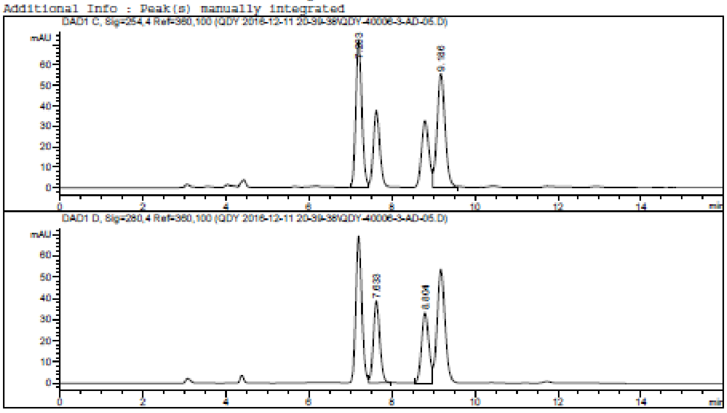

Area Percent Report

Sorted By : Signal
Multiplier : 1.0000
Dilution : 1.0000
Use Multiplier & Dilution Factor with ISTDs

Signal 1: DAD1 C, Sig=254,4 Ref=360,100

Peak #	RetTime [min]	Type	Width [min]	Area [mAU*s]	Height [mAU]	Area %
1	7.203	BV	0.1562	727.00128	71.74940	49.5600
2	9.186	VB	0.2056	739.91150	55.46935	50.4400

Totals : 1466.91278 127.21876

Peak #	RetTime [min]	Type	Width [min]	Area [mAU*s]	Height [mAU]	Area %
1	7.633	VB	0.1661	415.61536	38.45949	50.6287
2	8.804	BV	0.1910	405.29398	33.07433	49.3713

Totals : 820.90933 71.53382

*** End of Report ***

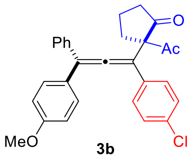

3b

Acq. Operator : Seq. Line : 2
Acq. Instrument : Instrument 1 Location : Vial 33
Injection Date : 12/18/2016 9:26:08 PM Inj : 1
Inj Volume : 5.000 µl
Different Inj Volume from Sequence ! Actual Inj Volume : 1.000 µl
Acq. Method : C:\CHEM32\1\DATA\QDY 2016-12-18 21-11-49\AD-05-20.M
Last changed : 12/18/2016 9:25:14 PM
(modified after loading)
Analysis Method : C:\CHEM32\1\METHODS\IC-05-30-1.3.M
Last changed : 12/18/2016 9:48:04 PM
(modified after loading)
Additional Info : Peak(s) manually integrated

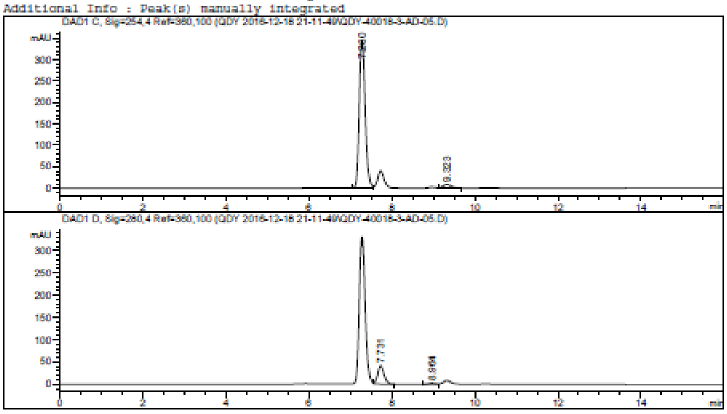

Area Percent Report

Sorted By : Signal
Multiplier : 1.0000
Dilution : 1.0000
Use Multiplier & Dilution Factor with ISTDs

Signal 1: DAD1 C, Sig=254,4 Ref=360,100

Peak #	RetTime [min]	Type	Width [min]	Area [mAU*s]	Height [mAU]	Area %
1	7.280	BV	0.1504	3380.13574	344.81693	96.7568
2	9.323	VB	0.2039	113.29745	8.47690	3.2432

Totals : 3493.43320 353.29382

Signal 2: DAD1 D, Sig=280,4 Ref=360,100

Peak #	RetTime [min]	Type	Width [min]	Area [mAU*s]	Height [mAU]	Area %
1	7.731	VB	0.1628	441.41266	41.25728	94.1989
2	8.964	BV	0.1809	27.18377	2.31690	5.8011

Totals : 468.59643 43.57418

*** End of Report ***

Supplementary Figure 172. HPLC spectrum for 3b

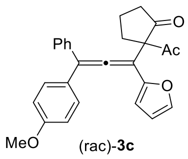

(rac)-**3c**

```
=====================================================================
Acq. Operator   :                                 Seq. Line :   4
Acq. Instrument : Instrument 1                     Location : Vial 31
Injection Date  : 12/26/2016 3:06:26 PM                 Inj :   1
                                                 Inj Volume : 5.000 µl
Acq. Method     : C:\CHEM32\1\DATA\QDY 2016-12-26 14-09-48\ETOH-AD-03-25-0.5.M
Last changed    : 12/21/2016 2:33:56 PM
Analysis Method : C:\CHEM32\1\METHODS\ETOH-IC-01-25-05.M
Last changed    : 12/25/2016 12:31:15 AM
Additional Info : Peak(s) manually integrated
```

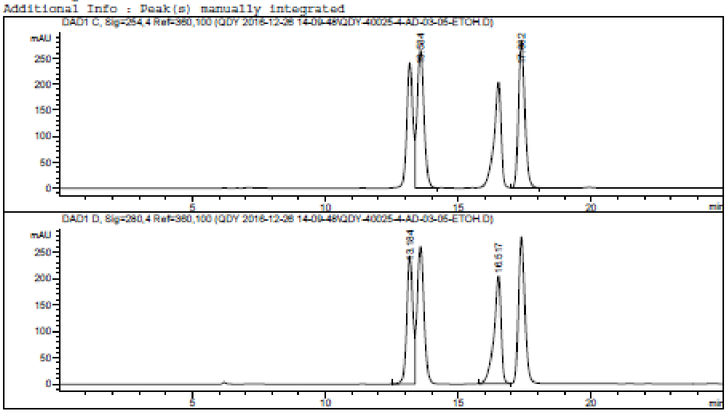


```
=====================================================================
                         Area Percent Report
=====================================================================

Sorted By             :      Signal
Multiplier            :      1.0000
Dilution              :      1.0000
Use Multiplier & Dilution Factor with ISTDs
```

Signal 1: DAD1 C, Sig=254,4 Ref=360,100

Peak #	RetTime [min]	Type	Width [min]	Area [mAU*s]	Height [mAU]	Area %
1	13.584	VB	0.2722	4725.58398	264.86414	49.9491
2	17.382	VB	0.2570	4735.20996	283.24130	50.0509
Totals :				9460.79395	548.10544	

Signal 2: DAD1 D, Sig=280,4 Ref=360,100

Peak #	RetTime [min]	Type	Width [min]	Area [mAU*s]	Height [mAU]	Area %
1	13.184	BV	0.2422	3813.76855	241.50697	50.1114
2	16.517	BV	0.2696	3796.81274	203.57631	49.8886

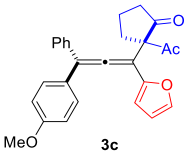

3c

```
=====================================================================
Acq. Operator   :                                 Seq. Line :   2
Acq. Instrument : Instrument 1                     Location : Vial 31
Injection Date  : 12/25/2016 10:00:57 PM                Inj :   1
                                                 Inj Volume : 5.000 µl
Acq. Method     : C:\CHEM32\1\DATA\QDY 2016-12-25 21-36-47\ETOH-AD-03-25-0.5.M
Last changed    : 12/25/2016 10:12:41 PM
                  (modified after loading)
Analysis Method : C:\CHEM32\1\METHODS\ETOH-IC-01-25-05.M
Last changed    : 12/25/2016 12:31:15 AM
Additional Info : Peak(s) manually integrated
```

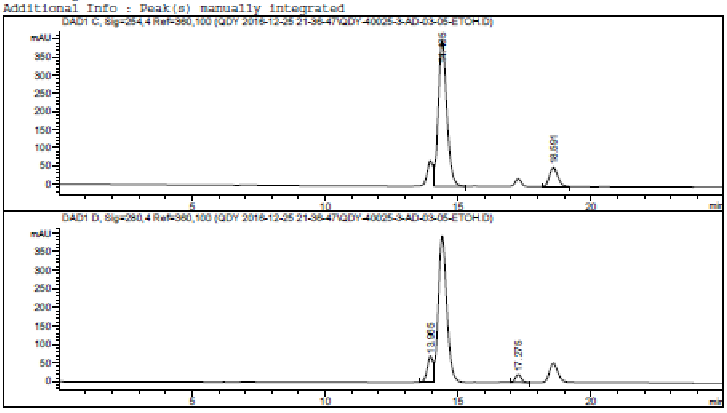


```
=====================================================================
                         Area Percent Report
=====================================================================

Sorted By             :      Signal
Multiplier            :      1.0000
Dilution              :      1.0000
Use Multiplier & Dilution Factor with ISTDs
```

Signal 1: DAD1 C, Sig=254,4 Ref=360,100

Peak #	RetTime [min]	Type	Width [min]	Area [mAU*s]	Height [mAU]	Area %
1	14.405	VB	0.3393	8968.72949	403.54016	88.5462
2	18.591	BB	0.3383	1160.14307	53.21543	11.4538
Totals :				1.01289e4	456.75559	

Peak #	RetTime [min]	Type	Width [min]	Area [mAU*s]	Height [mAU]	Area %
1	13.965	BV	0.2313	1063.85193	70.00453	76.9423
2	17.275	BB	0.2337	318.80972	21.17364	23.0577
Totals :				1382.66165	91.17817	

```
=====================================================================
                          *** End of Report ***
```

Supplementary Figure 173. HPLC spectrum for 3c

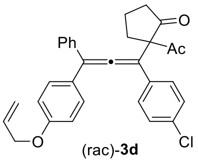

(rac)-**3d**

```
==================================================================
Acq. Operator   :                                  Seq. Line :   2
Acq. Instrument : Instrument 1                      Location : Vial 33
Injection Date  : 12/22/2016 10:42:58 AM                 Inj :   1
                                                  Inj Volume : 5.000 µl
Acq. Method     : C:\CHEM32\1\DATA\QDY 2016-12-22 10-13-49\ETOH-AD-03-25-0.5.M
Last changed    : 12/22/2016 11:03:45 AM
                  (modified after loading)
Analysis Method : C:\CHEM32\1\METHODS\AD-03-25-07.M
Last changed    : 12/21/2016 7:43:26 PM
Additional Info : Peak(s) manually integrated
```

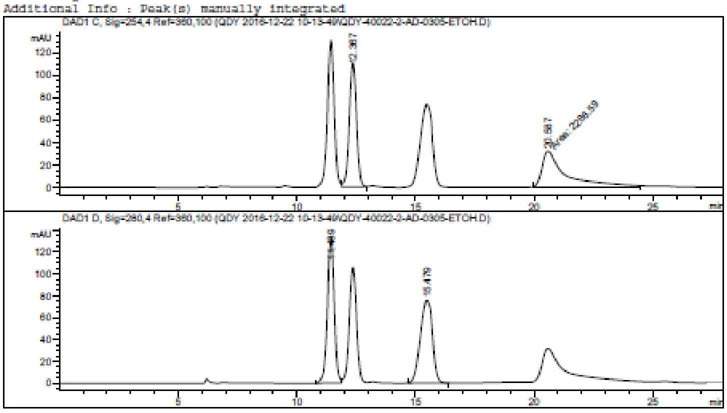


```
=====================================================================
                         Area Percent Report
=====================================================================

Sorted By             :      Signal
Multiplier            :      1.0000
Dilution              :      1.0000
Use Multiplier & Dilution Factor with ISTDs
```

Signal 1: DAD1 C, Sig=254,4 Ref=360,100

Peak #	RetTime [min]	Type	Width [min]	Area [mAU*s]	Height [mAU]	Area %
1	12.367	VB	0.3293	2336.37280	110.21536	50.5384
2	20.587	MM	1.1878	2286.59106	32.08462	49.4616
Totals :				4622.96387	142.29998	

Peak #	RetTime [min]	Type	Width [min]	Area [mAU*s]	Height [mAU]	Area %
1	11.439	BV	0.3084	2670.96265	134.09711	49.8320
2	15.479	BB	0.5162	2688.97021	75.73504	50.1680
Totals :				5359.93286	209.83215	

```
=====================================================================
                         *** End of Report ***
```

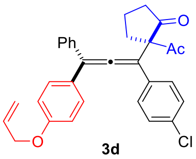

3d

```
==================================================================
Acq. Operator   :                                  Seq. Line :   7
Acq. Instrument : Instrument 1                      Location : Vial 33
Injection Date  : 12/23/2016 12:06:26 PM                 Inj :   1
                                                  Inj Volume : 5.000 µl
Acq. Method     : C:\CHEM32\1\DATA\QDY 2016-12-23 10-16-57\ETOH-AD-03-25-0.5.M
Last changed    : 12/21/2016 2:33:56 PM
Analysis Method : C:\CHEM32\1\METHODS\AD-03-25-07.M
Last changed    : 12/23/2016 1:47:07 PM
                  (modified after loading)
Additional Info : Peak(s) manually integrated
```

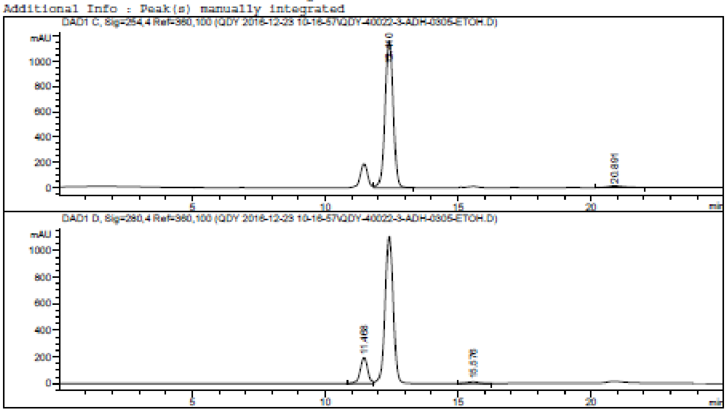


```
=====================================================================
                         Area Percent Report
=====================================================================

Sorted By             :      Signal
Multiplier            :      1.0000
Dilution              :      1.0000
Use Multiplier & Dilution Factor with ISTDs
```

Signal 1: DAD1 C, Sig=254,4 Ref=360,100

Peak #	RetTime [min]	Type	Width [min]	Area [mAU*s]	Height [mAU]	Area %
1	12.410	VB	0.3327	2.48662e4	1166.35120	97.6844
2	20.891	BB	0.6552	589.45648	13.50646	2.3156
Totals :				2.54557e4	1179.85765	

Peak #	RetTime [min]	Type	Width [min]	Area [mAU*s]	Height [mAU]	Area %
1	11.468	BV	0.3041	3771.59863	192.94066	92.3225
2	15.576	BB	0.3789	313.64468	11.61888	7.6775
Totals :				4085.24332	204.55954	

```
=====================================================================
                         *** End of Report ***
```

Supplementary Figure 174. HPLC spectrum for 3d

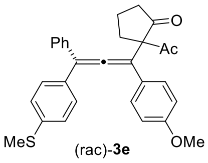

(rac)-**3e**

```
=====================================================================
Acq. Operator   :                               Seq. Line :   3
Acq. Instrument : Instrument 1                   Location : Vial 35
Injection Date  : 12/17/2016 11:07:14 AM              Inj :   1
                                               Inj Volume : 5.000 µl
Acq. Method     : C:\CHEM32\1\DATA\QDY 2016-12-17 10-09-58\AD-10-25.M
Last changed    : 12/17/2016 11:22:08 AM
                  (modified after loading)
Analysis Method : C:\CHEM32\1\METHODS\AD-50-30.M
Last changed    : 12/17/2016 10:28:54 PM
                  (modified after loading)
Additional Info : Peak(s) manually integrated
```

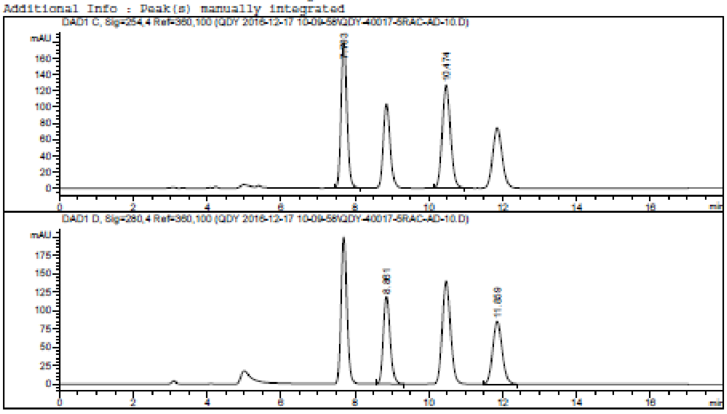


```
=====================================================================
                        Area Percent Report
=====================================================================

Sorted By             :      Signal
Multiplier            :      1.0000
Dilution              :      1.0000
Use Multiplier & Dilution Factor with ISTDs
```

Signal 1: DAD1 C, Sig=254,4 Ref=360,100

Peak #	RetTime [min]	Type	Width [min]	Area [mAU*s]	Height [mAU]	Area %
1	7.703	BB	0.1708	1999.58411	181.13736	50.3226
2	10.474	BB	0.2418	1973.94446	126.68971	49.6774

Totals : 3973.52856 307.82707

Signal 2: DAD1 D, Sig=280,4 Ref=360,100

Peak #	RetTime [min]	Type	Width [min]	Area [mAU*s]	Height [mAU]	Area %
1	8.861	BB	0.1986	1530.18848	118.56132	50.2966
2	11.859	BB	0.2754	1512.13953	85.07885	49.7034

Totals : 3042.32800 203.64017

```
=====================================================================
                        *** End of Report ***
```

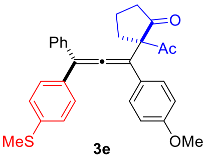

3e

```
=====================================================================
Acq. Operator   :                               Seq. Line :  19
Acq. Instrument : Instrument 1                   Location : Vial 32
Injection Date  : 12/17/2016 6:34:55 PM               Inj :   1
                                               Inj Volume : 5.000 µl
Acq. Method     : C:\CHEM32\1\DATA\QDY 2016-12-17 10-09-58\AD-10-25.M
Last changed    : 12/17/2016 6:33:07 PM
                  (modified after loading)
Analysis Method : C:\CHEM32\1\METHODS\AD-50-30.M
Last changed    : 12/17/2016 10:28:54 PM
                  (modified after loading)
Additional Info : Peak(s) manually integrated
```

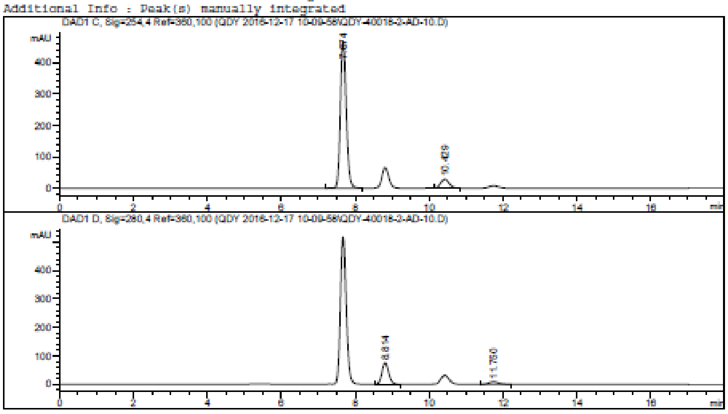


```
=====================================================================
                        Area Percent Report
=====================================================================

Sorted By             :      Signal
Multiplier            :      1.0000
Dilution              :      1.0000
Use Multiplier & Dilution Factor with ISTDs
```

Signal 1: DAD1 C, Sig=254,4 Ref=360,100

Peak #	RetTime [min]	Type	Width [min]	Area [mAU*s]	Height [mAU]	Area %
1	7.674	BB	0.1701	5147.08936	468.59857	91.9216
2	10.429	BB	0.2391	452.34695	29.13834	8.0784

Totals : 5599.43631 497.73691

Signal 2: DAD1 D, Sig=280,4 Ref=360,100

Peak #	RetTime [min]	Type	Width [min]	Area [mAU*s]	Height [mAU]	Area %
1	8.814	BB	0.1962	954.48505	75.15132	85.9938
2	11.750	BB	0.2766	155.46130	8.61222	14.0062

Totals : 1109.94635 83.76354

```
=====================================================================
                        *** End of Report ***
```

Supplementary Figure 175. HPLC spectrum for 3e

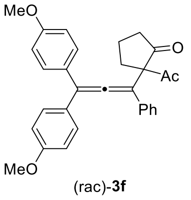

(rac)-**3f**

Acq. Operator :　　Seq. Line : 22
Acq. Instrument : Instrument 1　　Location : Vial 34
Injection Date : 12/1/2016 11:43:21 PM　　Inj : 1
Inj Volume : 5.000 µl
Acq. Method : C:\CHEM32\1\DATA\QDY 2016-12-01 15-06-50\AD-10-30.M
Last changed : 12/1/2016 10:27:40 PM
(modified after loading)
Analysis Method : C:\CHEM32\1\METHODS\ETOH-IC-01-25-05.M
Last changed : 1/1/2017 3:00:17 PM
(modified after loading)
Additional Info : Peak(s) manually integrated

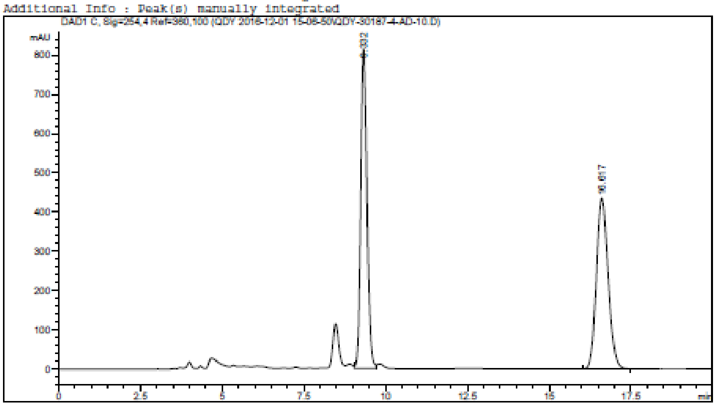

Area Percent Report

Sorted By : Signal
Multiplier : 1.0000
Dilution : 1.0000
Use Multiplier & Dilution Factor with ISTDs

Signal 1: DAD1 C, Sig=254,4 Ref=360,100

Peak #	RetTime [min]	Type	Width [min]	Area [mAU*s]	Height [mAU]	Area %
1	9.332	VV	0.2081	1.09001e4	814.87524	50.0876
2	16.617	BB	0.3873	1.08620e4	435.09927	49.9124

Totals : 2.17621e4 1249.97452

*** End of Report ***

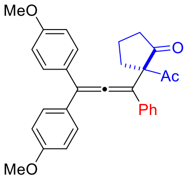

3f

Acq. Operator :　　Seq. Line : 24
Acq. Instrument : Instrument 1　　Location : Vial 31
Injection Date : 12/22/2016 11:57:21 PM　　Inj : 1
Inj Volume : 5.000 µl
Acq. Method : C:\CHEM32\1\DATA\QDY 2016-12-22 16-06-21\AD-10-25.M
Last changed : 9/12/2015 7:30:37 PM
Analysis Method : C:\CHEM32\1\METHODS\AD-03-25-07.M
Last changed : 12/22/2016 7:51:24 PM
(modified after loading)
Additional Info : Peak(s) manually integrated

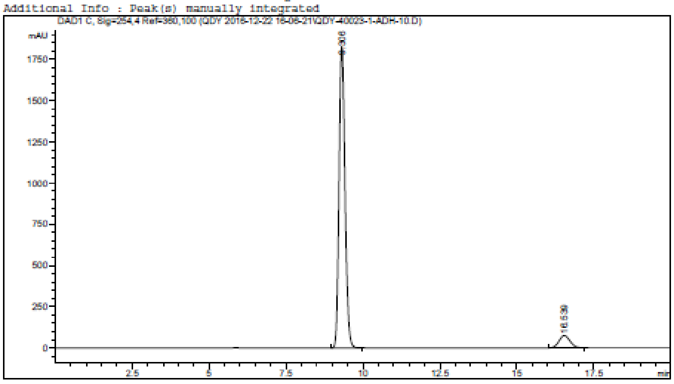

Area Percent Report

Sorted By : Signal
Multiplier : 1.0000
Dilution : 1.0000
Use Multiplier & Dilution Factor with ISTDs

Signal 1: DAD1 C, Sig=254,4 Ref=360,100

Peak #	RetTime [min]	Type	Width [min]	Area [mAU*s]	Height [mAU]	Area %
1	9.306	BB	0.2050	2.42926e4	1828.79016	92.9066
2	16.539	BB	0.3852	1854.72717	74.82449	7.0934

Totals : 2.61473e4 1903.61465

*** End of Report ***

Supplementary Figure 176. HPLC spectrum for 3f

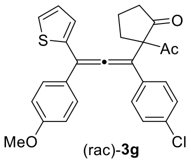

(rac)-**3g**

```
===================================================================
Acq. Operator   :                                  Seq. Line :  19
Acq. Instrument : Instrument 1                      Location : Vial 33
Injection Date  : 12/14/2016 5:19:42 PM                  Inj :   1
                                                  Inj Volume : 5.000 µl
Acq. Method     : C:\CHEM32\1\DATA\QDY 2016-12-14 09-56-02\AD-05-20.M
Last changed    : 12/14/2016 5:18:48 PM
                  (modified after loading)
Analysis Method : C:\CHEM32\1\METHODS\AD-50-30.M
Last changed    : 12/18/2016 9:24:07 PM
                  (modified after loading)
Additional Info : Peak(s) manually integrated
```

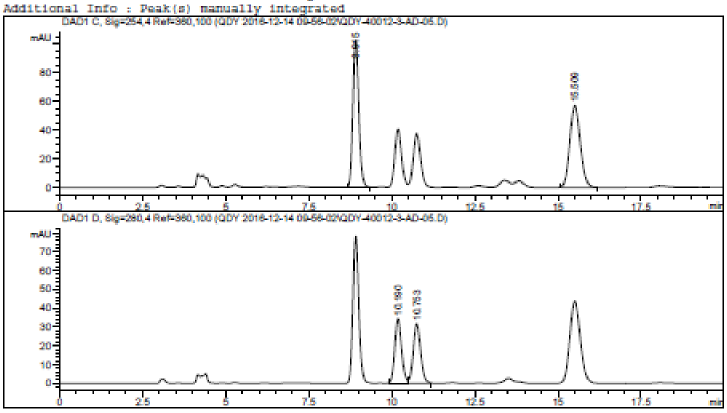


```
===================================================================
                     Area Percent Report
===================================================================

Sorted By             :      Signal
Multiplier            :      1.0000
Dilution              :      1.0000
Use Multiplier & Dilution Factor with ISTDs
```

Signal 1: DAD1 C, Sig=254,4 Ref=360,100

Peak #	RetTime [min]	Type	Width [min]	Area [mAU*s]	Height [mAU]	Area %
1	8.915	BB	0.1908	1271.38049	102.41248	50.2509
2	15.509	BB	0.3401	1258.68274	57.34032	49.7491
Totals :				2530.06323	159.75280	

Signal 2: DAD1 D, Sig=280,4 Ref=360,100

Peak #	RetTime [min]	Type	Width [min]	Area [mAU*s]	Height [mAU]	Area %
1	10.190	BV	0.2195	487.97296	34.39703	49.8834
2	10.753	VB	0.2379	490.25323	31.79630	50.1166
Totals :				978.22620	66.19333	

```
===================================================================
                     *** End of Report ***
```

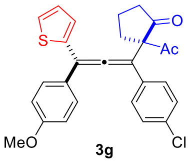

3g

```
===================================================================
Acq. Operator   :                                  Seq. Line :  41
Acq. Instrument : Instrument 1                      Location : Vial 34
Injection Date  : 12/18/2016 2:53:23 AM                  Inj :   1
                                                  Inj Volume : 5.000 µl
Acq. Method     : C:\CHEM32\1\DATA\QDY 2016-12-17 10-09-58\AD-05-20.M
Last changed    : 12/18/2016 2:30:04 AM
                  (modified after loading)
Analysis Method : C:\CHEM32\1\METHODS\AD-50-30.M
Last changed    : 12/18/2016 9:40:47 PM
                  (modified after loading)
Additional Info : Peak(s) manually integrated
```

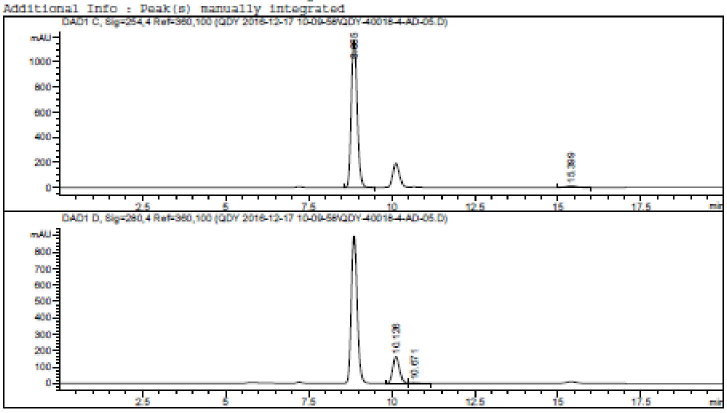


```
===================================================================
                     Area Percent Report
===================================================================

Sorted By             :      Signal
Multiplier            :      1.0000
Dilution              :      1.0000
Use Multiplier & Dilution Factor with ISTDs
```

Signal 1: DAD1 C, Sig=254,4 Ref=360,100

Peak #	RetTime [min]	Type	Width [min]	Area [mAU*s]	Height [mAU]	Area %
1	8.865	BB	0.1898	1.44274e4	1170.38037	97.9690
2	15.399	BB	0.3363	299.08856	13.72236	2.0310
Totals :				1.47265e4	1184.10273	

Signal 2: DAD1 D, Sig=280,4 Ref=360,100

Peak #	RetTime [min]	Type	Width [min]	Area [mAU*s]	Height [mAU]	Area %
1	10.126	BV	0.2185	2298.65723	163.05170	97.5052
2	10.671	VB	0.2264	58.81377	3.98117	2.4948
Totals :				2357.47100	167.03287	

```
===================================================================
                     *** End of Report ***
```

Supplementary Figure 177. HPLC spectrum for 3g

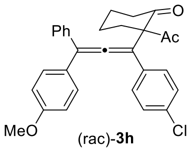

(rac)-**3h**

```
==================================================================
Acq. Operator   :                              Seq. Line :   9
Acq. Instrument : Instrument 1                  Location : Vial 31
Injection Date  : 12/20/2016 1:59:46 PM              Inj :   1
                                              Inj Volume : 5.000 µl
Acq. Method     : C:\CHEM32\1\DATA\QDY 2016-12-20 10-04-00\AD-05-20.M
Last changed    : 12/20/2016 1:58:54 PM
                  (modified after loading)
Analysis Method : C:\CHEM32\1\METHODS\ETOH-IC-01-25-05.M
Last changed    : 12/26/2016 1:11:51 PM
                  (modified after loading)
Additional Info : Peak(s) manually integrated
```

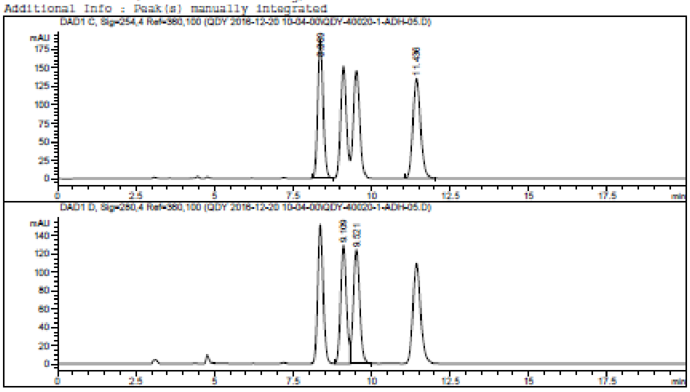


```
=====================================================================
                         Area Percent Report
=====================================================================

Sorted By             :      Signal
Multiplier            :      1.0000
Dilution              :      1.0000
Use Multiplier & Dilution Factor with ISTDs
```

Signal 1: DAD1 C, Sig=254,4 Ref=360,100

Peak #	RetTime [min]	Type	Width [min]	Area [mAU*s]	Height [mAU]	Area %
1	8.369	BB	0.1877	2288.36597	188.41090	49.7870
2	11.436	BB	0.2625	2307.94678	135.61908	50.2130

Totals : 4596.31274 324.02998

Signal 2: DAD1 D, Sig=280,4 Ref=360,100

Peak #	RetTime [min]	Type	Width [min]	Area [mAU*s]	Height [mAU]	Area %
1	9.109	BV	0.2034	1703.65442	129.56859	49.2211
2	9.521	VB	0.2189	1757.57336	124.31697	50.7789

Totals : 3461.22778 253.88556

```
=====================================================================
                          *** End of Report ***
```

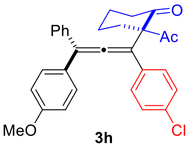

3h

```
==================================================================
Acq. Operator   :                              Seq. Line :   2
Acq. Instrument : Instrument 1                  Location : Vial 34
Injection Date  : 12/28/2016 3:53:13 PM              Inj :   1
                                              Inj Volume : 5.000 µl
Different Inj Volume from Sequence !   Actual Inj Volume : 6.000 µl
Acq. Method     : C:\CHEM32\1\DATA\QDY 2016-12-28 15-38-58\AD-05-20.M
Last changed    : 12/28/2016 3:52:19 PM
                  (modified after loading)
Analysis Method : C:\CHEM32\1\METHODS\ETOH-IC-01-25-05.M
Last changed    : 12/25/2016 12:31:15 AM
Additional Info : Peak(s) manually integrated
```

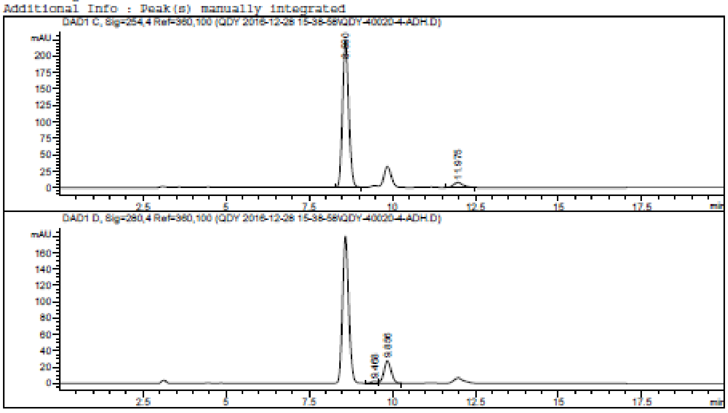


```
=====================================================================
                         Area Percent Report
=====================================================================

Sorted By             :      Signal
Multiplier            :      1.0000
Dilution              :      1.0000
Use Multiplier & Dilution Factor with ISTDs
```

Signal 1: DAD1 C, Sig=254,4 Ref=360,100

Peak #	RetTime [min]	Type	Width [min]	Area [mAU*s]	Height [mAU]	Area %
1	8.590	BB	0.1994	2898.87183	223.33513	94.8538
2	11.975	BB	0.2967	157.27544	8.02446	5.1462

Totals : 3056.14726 231.35959

Signal 2: DAD1 D, Sig=280,4 Ref=360,100

Peak #	RetTime [min]	Type	Width [min]	Area [mAU*s]	Height [mAU]	Area %
1	9.468	BV	0.1947	30.80071	2.44990	6.8343
2	9.856	VB	0.2345	419.87592	27.75845	93.1657

Totals : 450.67662 30.20835

```
=====================================================================
                          *** End of Report ***
```

Supplementary Figure 178. HPLC spectrum for 3h

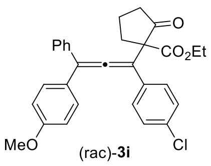


```
Acq. Operator   :                                   Seq. Line :  41
Acq. Instrument : Instrument 1                        Location : Vial 34
Injection Date  : 12/16/2016 1:40:39 AM                    Inj :  1
                                                    Inj Volume : 5.000 µl
Acq. Method     : C:\CHEM32\1\DATA\QDY 2016-12-15 09-57-24\IC-05-30.M
Last changed    : 8/23/2012 1:59:23 PM
Analysis Method : C:\CHEM32\1\METHODS\AD-03-25-07.M
Last changed    : 12/23/2016 11:10:32 AM
                  (modified after loading)
Additional Info : Peak(s) manually integrated
```

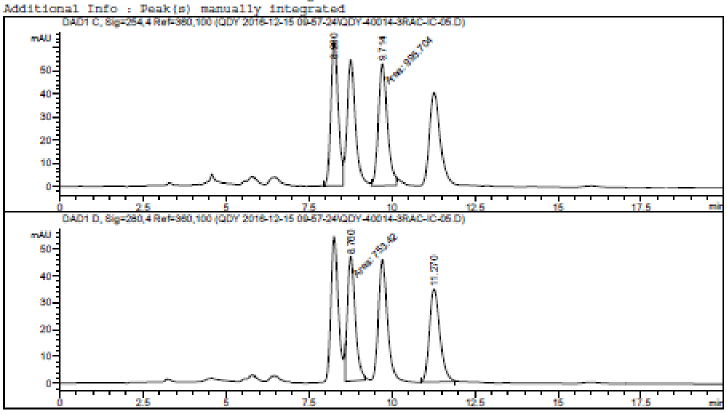

Area Percent Report

```
Sorted By             :      Signal
Multiplier            :      1.0000
Dilution              :      1.0000
Use Multiplier & Dilution Factor with ISTDs
```

Signal 1: DAD1 C, Sig=254,4 Ref=360,100

Peak #	RetTime [min]	Type	Width [min]	Area [mAU*s]	Height [mAU]	Area %
1	8.260	BV	0.2355	966.61340	62.83598	49.2588
2	9.714	MF	0.3144	995.70392	52.78615	50.7412

Totals : 1962.31732 115.62213

Peak #	RetTime [min]	Type	Width [min]	Area [mAU*s]	Height [mAU]	Area %
1	8.760	FM	0.2696	753.42029	46.57753	49.8034
2	11.270	BB	0.3366	759.36938	34.52636	50.1966

Totals : 1512.78967 81.10389

*** End of Report ***

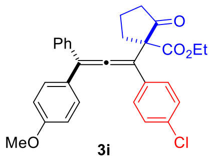

3i

```
Acq. Operator   :                                   Seq. Line :  6
Acq. Instrument : Instrument 1                        Location : Vial 31
Injection Date  : 12/24/2016 12:20:45 AM                   Inj :  1
                                                    Inj Volume : 5.000 µl
Acq. Method     : C:\CHEM32\1\DATA\QDY 2016-12-23 23-08-46\IC-05-30.M
Last changed    : 12/24/2016 12:19:53 AM
                  (modified after loading)
Analysis Method : C:\CHEM32\1\METHODS\AD-03-25-07.M
Last changed    : 12/24/2016 10:24:47 AM
                  (modified after loading)
Additional Info : Peak(s) manually integrated
```

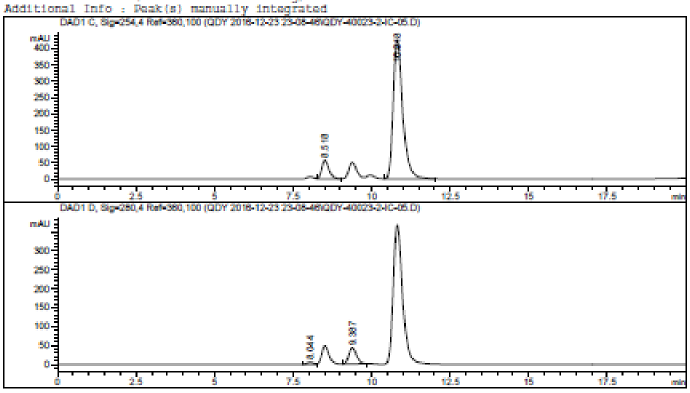

Area Percent Report

```
Sorted By             :      Signal
Multiplier            :      1.0000
Dilution              :      1.0000
Use Multiplier & Dilution Factor with ISTDs
```

Signal 1: DAD1 C, Sig=254,4 Ref=360,100

Peak #	RetTime [min]	Type	Width [min]	Area [mAU*s]	Height [mAU]	Area %
1	8.518	VB	0.2444	920.95679	57.03419	9.2614
2	10.818	BB	0.3211	9023.05469	426.03329	90.7386

Totals : 9944.01147 483.06748

Signal 2: DAD1 D, Sig=280,4 Ref=360,100

Peak #	RetTime [min]	Type	Width [min]	Area [mAU*s]	Height [mAU]	Area %
1	8.044	BV	0.2093	74.39173	5.51589	9.3557
2	9.387	BB	0.2595	720.75873	43.01805	90.6443

Totals : 795.15046 48.53394

*** End of Report ***

Supplementary Figure 179. HPLC spectrum for 3i

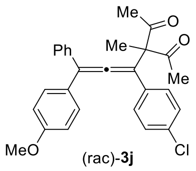

(rac)-**3j**

```
=====================================================================
Acq. Operator   :                               Seq. Line :   6
Acq. Instrument : Instrument 1                   Location : Vial 33
Injection Date  : 12/24/2016 11:58:45 PM              Inj :   1
                                               Inj Volume : 5.000 µl
Acq. Method     : C:\CHEM32\1\DATA\QDY 2016-12-24 22-17-41\AD-05-20.M
Last changed    : 12/24/2016 11:57:53 PM
                  (modified after loading)
Analysis Method : C:\CHEM32\1\METHODS\ETOH-IC-01-25-05.M
Last changed    : 12/25/2016 12:31:15 AM
Additional Info : Peak(s) manually integrated
```

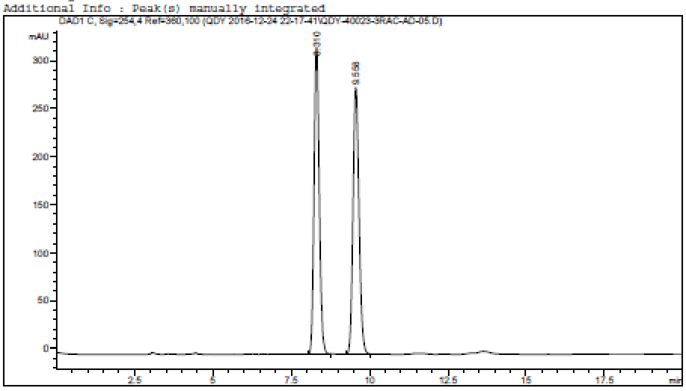


```
=====================================================================
                       Area Percent Report
=====================================================================

Sorted By             :      Signal
Multiplier            :      1.0000
Dilution              :      1.0000
Use Multiplier & Dilution Factor with ISTDs


Signal 1: DAD1 C, Sig=254,4 Ref=360,100

Peak RetTime Type  Width     Area      Height     Area
  #   [min]        [min]   [mAU*s]     [mAU]        %
----|-------|----|-------|----------|----------|--------|
   1   8.310 BB    0.1792 3708.74731  320.02621  49.9828
   2   9.558 BB    0.2079 3711.30029  277.69928  50.0172

Totals :                  7420.04761  597.72549

=====================================================================
                          *** End of Report ***
```

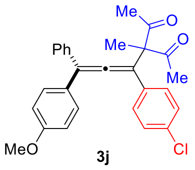

3j

```
=====================================================================
Acq. Operator   :                               Seq. Line :   7
Acq. Instrument : Instrument 1                   Location : Vial 33
Injection Date  : 12/27/2016 4:04:03 PM               Inj :   1
                                               Inj Volume : 5.000 µl
Acq. Method     : C:\CHEM32\1\DATA\QDY 2016-12-27 13-26-35\AD-05-20.M
Last changed    : 12/27/2016 4:03:11 PM
                  (modified after loading)
Analysis Method : C:\CHEM32\1\METHODS\ETOH-IC-01-25-05.M
Last changed    : 12/25/2016 12:31:15 AM
Additional Info : Peak(s) manually integrated
```

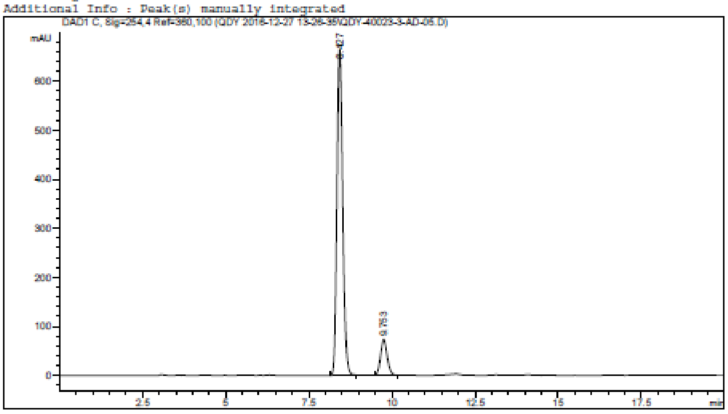


```
=====================================================================
                       Area Percent Report
=====================================================================

Sorted By             :      Signal
Multiplier            :      1.0000
Dilution              :      1.0000
Use Multiplier & Dilution Factor with ISTDs


Signal 1: DAD1 C, Sig=254,4 Ref=360,100

Peak RetTime Type  Width     Area      Height     Area
  #   [min]        [min]   [mAU*s]     [mAU]        %
----|-------|----|-------|----------|----------|--------|
   1   8.427 BB    0.1858 7953.15088  663.68365  88.7915
   2   9.753 BB    0.2127 1003.95966   72.87911  11.2085

Totals :                  8957.11053  736.56277

=====================================================================
                          *** End of Report ***
```

Supplementary Figure 180. HPLC spectrum for 3j

Data File C:\CHEM32\1\DATA\DEF_LC 2016-03-25 12-31-37\QDY-20094-1-IC-05.D
Sample Name:

===

Acq. Operator : Seq. Line : 8
Acq. Instrument : Instrument 1 Location : Vial 36
Injection Date : 3/25/2016 2:13:03 PM Inj : 1
Inj Volume : 5.000 µl
Acq. Method : C:\CHEM32\1\DATA\DEF_LC 2016-03-25 12-31-37\IC-05-30.M
Last changed : 3/25/2016 2:12:13 PM
(modified after loading)
Analysis Method : C:\CHEM32\1\METHODS\IC-15-30.M
Last changed : 8/20/2016 4:34:19 PM
(modified after loading)
Additional Info : Peak(s) manually integrated

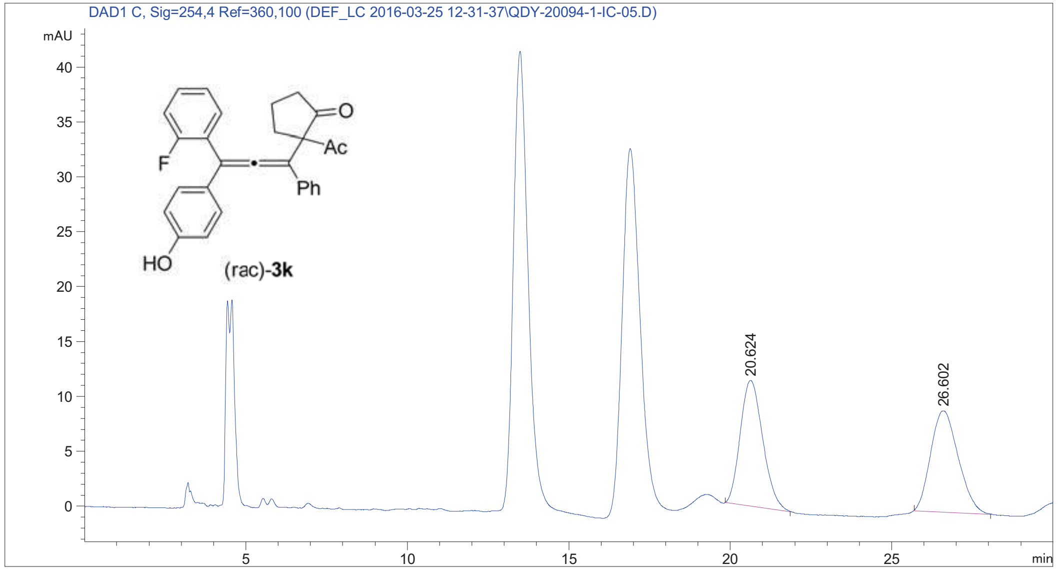

===

Area Percent Report

===

Sorted By : Signal
Multiplier : 1.0000
Dilution : 1.0000
Use Multiplier & Dilution Factor with ISTDs

Signal 1: DAD1 C, Sig=254,4 Ref=360,100

Peak #	RetTime [min]	Type	Width [min]	Area [mAU*s]	Height [mAU]	Area %
1	20.624	BB	0.6286	556.81903	11.46449	50.2834
2	26.602	BB	0.7284	550.54340	9.25036	49.7166

Totals : 1107.36243 20.71485

Data File C:\CHEM32\1\DATA\DEF_LC 2016-03-25 21-42-05\QDY-20093-2-IC-05.D
Sample Name:

===

Acq. Operator : Seq. Line : 2
Acq. Instrument : Instrument 1 Location : Vial 32
Injection Date : 3/25/2016 9:55:53 PM Inj : 1
Inj Volume : 5.000 µl
Acq. Method : C:\CHEM32\1\DATA\DEF_LC 2016-03-25 21-42-05\IC-05-30.M
Last changed : 3/25/2016 9:55:02 PM
(modified after loading)
Analysis Method : C:\CHEM32\1\METHODS\IC-15-30.M
Last changed : 8/20/2016 4:34:19 PM
(modified after loading)
Additional Info : Peak(s) manually integrated

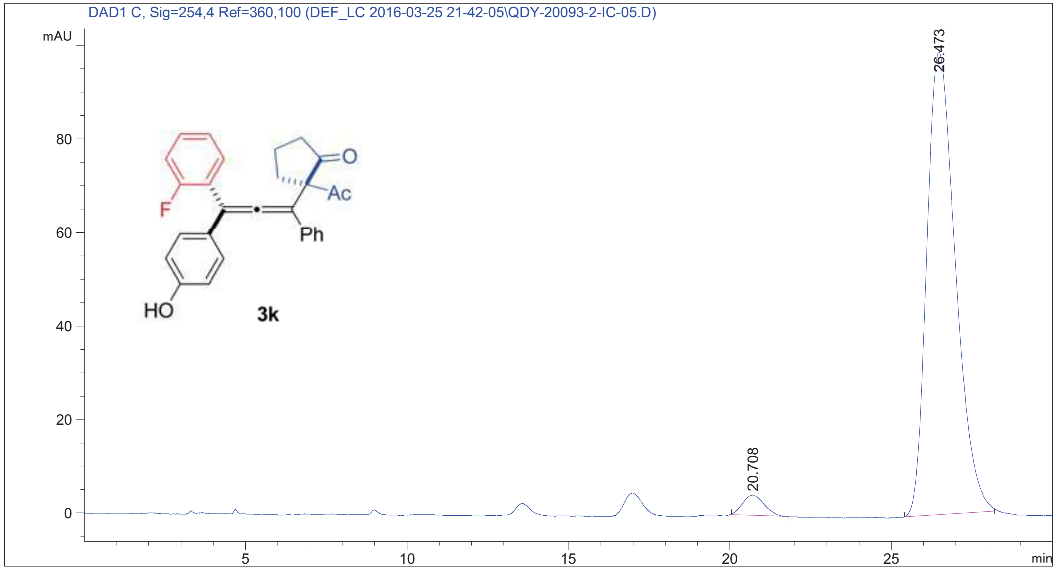

===

Area Percent Report

===

Sorted By : Signal
Multiplier : 1.0000
Dilution : 1.0000
Use Multiplier & Dilution Factor with ISTDs

Signal 1: DAD1 C, Sig=254,4 Ref=360,100

Peak #	RetTime [min]	Type	Width [min]	Area [mAU*s]	Height [mAU]	Area %
1	20.708	BB	0.5561	198.82310	4.29505	3.1520
2	26.473	BB	0.9478	6108.96680	99.00853	96.8480

Totals : 6307.78990 103.30358

Supplementary Figure 181. HPLC spectrum for 3k

```
Data File C:\CHEM32\1\DATA\DEF_LC 2016-01-06 10-08-49\QDY-10193-1-AD-10-05.D
Sample Name:

=====================================================================
Acq. Operator   :                               Seq. Line :  11
Acq. Instrument : Instrument 1                   Location : Vial 34
Injection Date  : 1/6/2016 1:13:34 PM                 Inj :   1
                                               Inj Volume : 5.000 µl
Acq. Method     : C:\CHEM32\1\DATA\DEF_LC 2016-01-06 10-08-49\AD-10-30-0.5.M
Last changed    : 1/6/2016 1:12:39 PM
                  (modified after loading)
Analysis Method : C:\CHEM32\1\METHODS\IC-15-30.M
Last changed    : 8/20/2016 4:08:47 PM
                  (modified after loading)
Additional Info : Peak(s) manually integrated
```

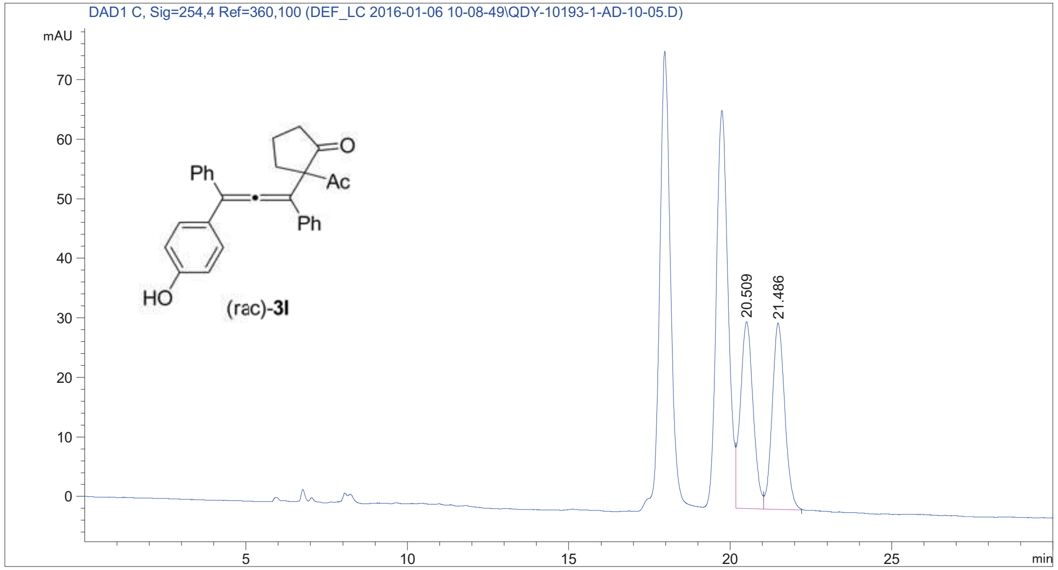

===
Area Percent Report
===

```
Sorted By             :      Signal
Multiplier            :      1.0000
Dilution              :      1.0000
Use Multiplier & Dilution Factor with ISTDs
```

Signal 1: DAD1 C, Sig=254,4 Ref=360,100

Peak #	RetTime [min]	Type	Width [min]	Area [mAU*s]	Height [mAU]	Area %
1	20.509	VV	0.4218	864.40710	31.34101	50.3231
2	21.486	VB	0.4203	853.30853	31.28021	49.6769

Totals : 1717.71564 62.62122


```
Data File C:\CHEM32\1\DATA\QDY 2016-06-03 23-40-32\QDY-20012-3-AD-10-05.D
Sample Name:

=====================================================================
Acq. Operator   :                               Seq. Line :   2
Acq. Instrument : Instrument 1                   Location : Vial 31
Injection Date  : 6/3/2016 11:54:26 PM                Inj :   1
                                               Inj Volume : 5.000 µl
Acq. Method     : C:\CHEM32\1\DATA\QDY 2016-06-03 23-40-32\AD-10-30-0.5.M
Last changed    : 6/3/2016 11:53:33 PM
                  (modified after loading)
Analysis Method : C:\CHEM32\1\METHODS\IC-15-30.M
Last changed    : 8/20/2016 4:08:47 PM
                  (modified after loading)
Additional Info : Peak(s) manually integrated
```

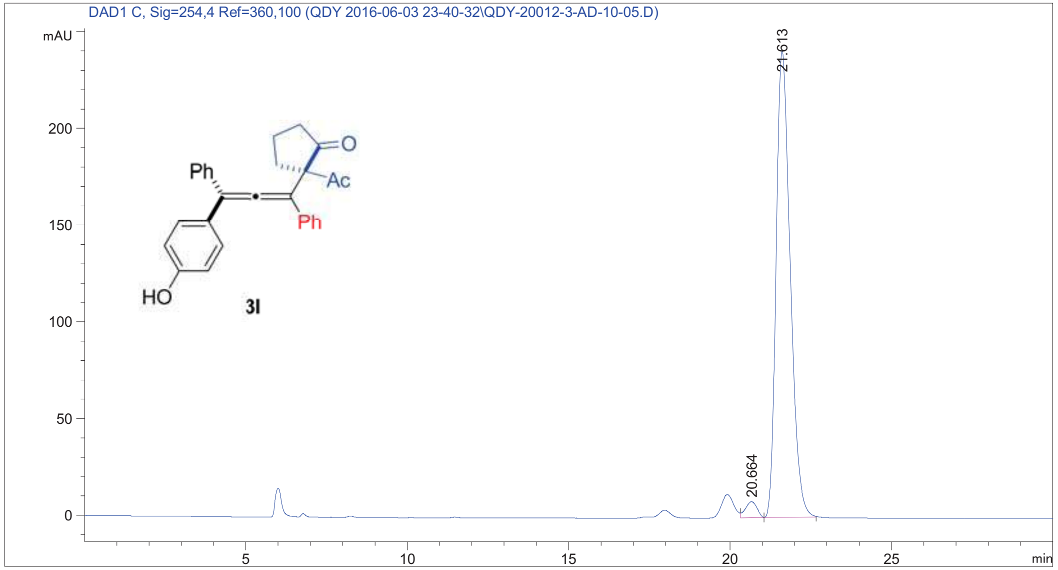

===
Area Percent Report
===

```
Sorted By             :      Signal
Multiplier            :      1.0000
Dilution              :      1.0000
Use Multiplier & Dilution Factor with ISTDs
```

Signal 1: DAD1 C, Sig=254,4 Ref=360,100

Peak #	RetTime [min]	Type	Width [min]	Area [mAU*s]	Height [mAU]	Area %
1	20.664	VV	0.3936	206.00653	8.29917	2.7387
2	21.613	VB	0.4703	7315.97168	240.87839	97.2613

Totals : 7521.97821 249.17756

Supplementary Figure 182. HPLC spectrum for 3l

Data File C:\CHEM32\1\DATA\DEF_LC 2016-01-14 10-09-39\QDY-20010-3-IC-05-05ETOH.D
Sample Name:

===

Acq. Operator : Seq. Line : 14
Acq. Instrument : Instrument 1 Location : Vial 31
Injection Date : 1/14/2016 2:42:09 PM Inj : 1
Inj Volume : 5.000 µl
Acq. Method : C:\CHEM32\1\DATA\DEF_LC 2016-01-14 10-09-39\ETOH-IC-05-30-05.M
Last changed : 1/14/2016 3:13:23 PM
(modified after loading)
Analysis Method : C:\CHEM32\1\METHODS\IC-15-30.M
Last changed : 8/20/2016 4:08:47 PM
(modified after loading)
Additional Info : Peak(s) manually integrated

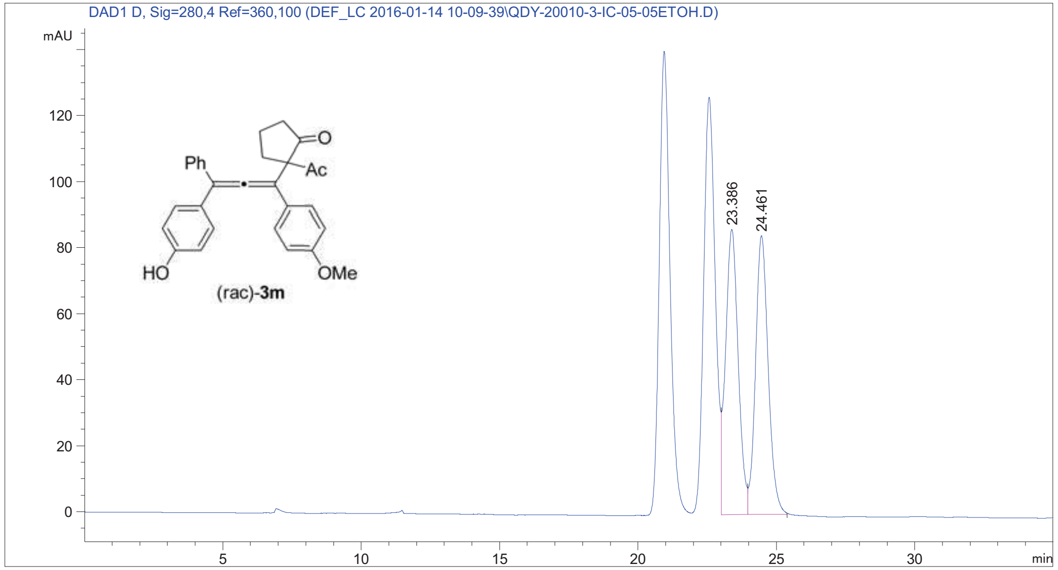

===

Area Percent Report

===

Sorted By : Signal
Multiplier : 1.0000
Dilution : 1.0000
Use Multiplier & Dilution Factor with ISTDs

Signal 1: DAD1 D, Sig=280,4 Ref=360,100

Peak #	RetTime [min]	Type	Width [min]	Area [mAU*s]	Height [mAU]	Area %
1	23.386	VV	0.4855	2753.98926	86.47826	50.5775
2	24.461	VB	0.4898	2691.10376	84.43309	49.4225

Totals : 5445.09302 170.91135

Supplementary Figure 183. HPLC spectrum for 3m

Data File C:\CHEM32\1\DATA\DEF_LC 2016-01-14 10-09-39\QDY-20012-1-IC-05-05ETOH.D
Sample Name:

===

Acq. Operator : Seq. Line : 15
Acq. Instrument : Instrument 1 Location : Vial 35
Injection Date : 1/14/2016 3:18:15 PM Inj : 1
Inj Volume : 5.000 µl
Acq. Method : C:\CHEM32\1\DATA\DEF_LC 2016-01-14 10-09-39\ETOH-IC-05-30-05.M
Last changed : 1/14/2016 3:45:28 PM
(modified after loading)
Analysis Method : C:\CHEM32\1\METHODS\IC-15-30.M
Last changed : 8/20/2016 4:08:47 PM
(modified after loading)
Additional Info : Peak(s) manually integrated

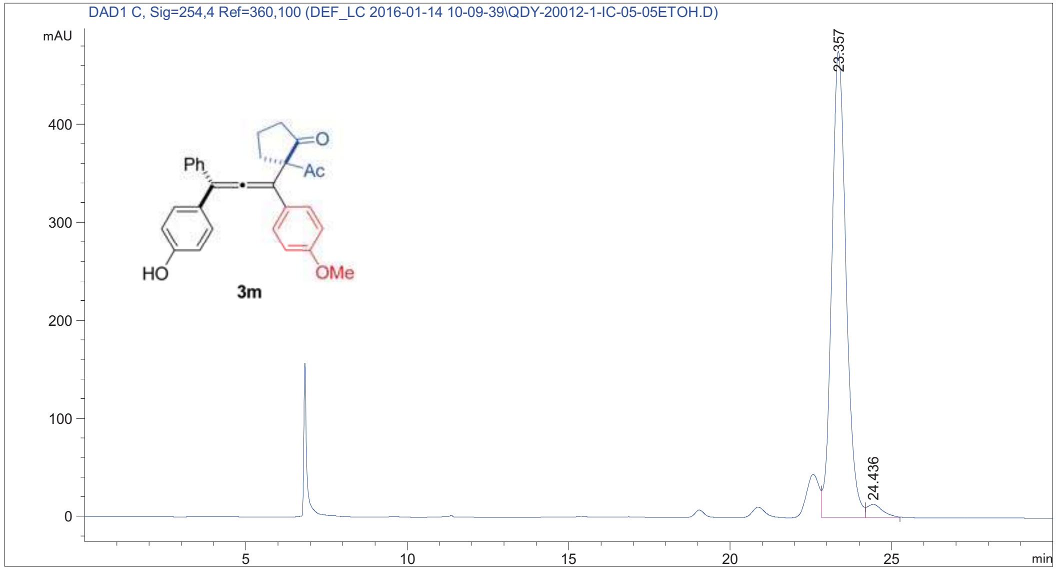

===

Area Percent Report

===

Sorted By : Signal
Multiplier : 1.0000
Dilution : 1.0000
Use Multiplier & Dilution Factor with ISTDs

Signal 1: DAD1 C, Sig=254,4 Ref=360,100

Peak #	RetTime [min]	Type	Width [min]	Area [mAU*s]	Height [mAU]	Area %
1	23.357	VV	0.4742	1.46803e4	475.52399	97.0404
2	24.436	VB	0.4846	447.73767	13.37484	2.9596

Totals : 1.51281e4 488.89882

Data File C:\CHEM32\1\DATA\DEF_LC 2016-01-22 10-07-33\QDY-20028-2-AD-10-05.D
Sample Name:

===

Acq. Operator : Seq. Line : 31
Acq. Instrument : Instrument 1 Location : Vial 33
Injection Date : 1/22/2016 7:46:01 PM Inj : 1
Inj Volume : 5.000 µl
Acq. Method : C:\CHEM32\1\DATA\DEF_LC 2016-01-22 10-07-33\AD-10-30-0.5.M
Last changed : 1/7/2016 11:07:32 PM
Analysis Method : C:\CHEM32\1\METHODS\AD-01-10-2.0.M
Last changed : 6/26/2016 2:13:09 PM
(modified after loading)
Additional Info : Peak(s) manually integrated

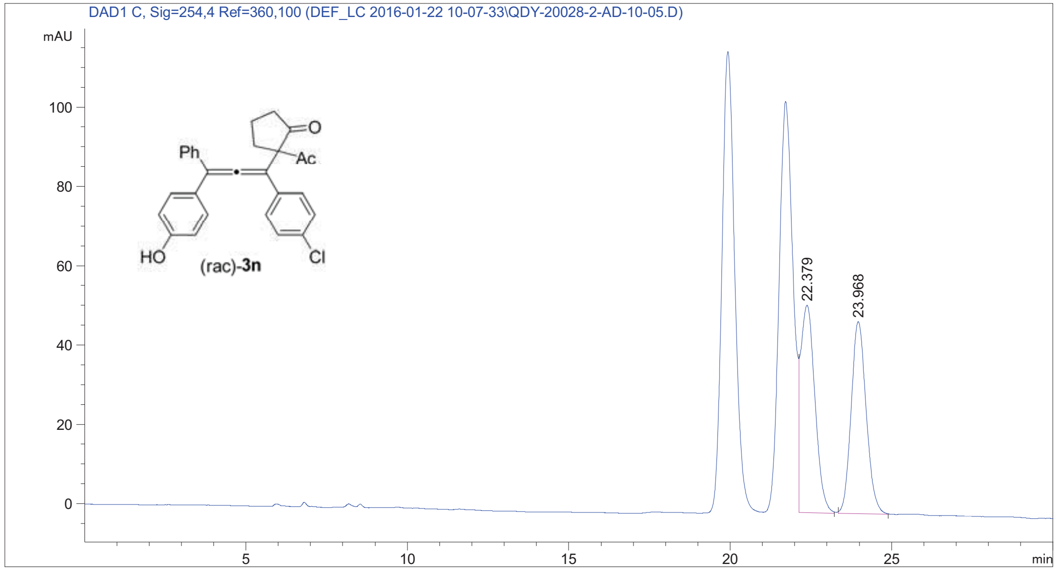

===

Area Percent Report

===

Sorted By : Signal
Multiplier : 1.0000
Dilution : 1.0000
Use Multiplier & Dilution Factor with ISTDs

Signal 1: DAD1 C, Sig=254,4 Ref=360,100

Peak #	RetTime [min]	Type	Width [min]	Area [mAU*s]	Height [mAU]	Area %
1	22.379	VB	0.4605	1617.20544	52.33952	50.8224
2	23.968	BB	0.5012	1564.86450	48.39814	49.1776

Totals : 3182.06995 100.73766

===

Data File C:\CHEM32\1\DATA\DEF_LC 2016-01-28 18-11-20\QDY-20036-AD-10-05.D
Sample Name:

===

Acq. Operator : Seq. Line : 2
Acq. Instrument : Instrument 1 Location : Vial 31
Injection Date : 1/28/2016 6:22:19 PM Inj : 1
Inj Volume : 5.000 µl
Acq. Method : C:\CHEM32\1\DATA\DEF_LC 2016-01-28 18-11-20\AD-10-30-0.5.M
Last changed : 1/28/2016 6:22:31 PM
(modified after loading)
Analysis Method : C:\CHEM32\1\METHODS\AD-01-10-2.0.M
Last changed : 6/26/2016 2:13:09 PM
(modified after loading)
Additional Info : Peak(s) manually integrated

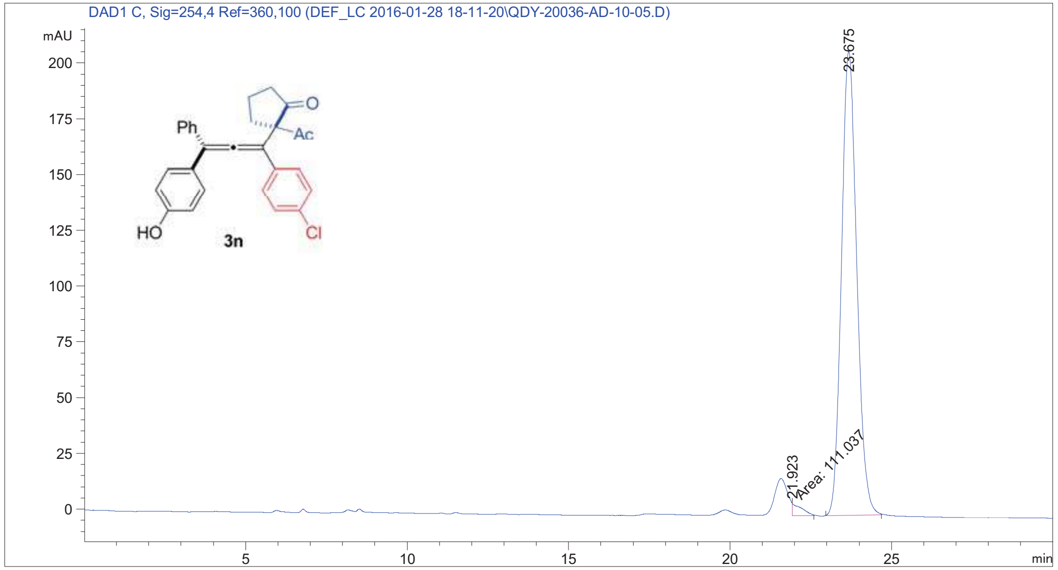

===

Area Percent Report

===

Sorted By : Signal
Multiplier : 1.0000
Dilution : 1.0000
Use Multiplier & Dilution Factor with ISTDs

Signal 1: DAD1 C, Sig=254,4 Ref=360,100

Peak #	RetTime [min]	Type	Width [min]	Area [mAU*s]	Height [mAU]	Area %
1	21.923	FM	0.3323	111.03651	5.56921	1.5975
2	23.675	BB	0.5077	6839.57959	207.98744	98.4025

Totals : 6950.61610 213.55665

Supplementary Figure 184. HPLC spectrum for 3n

Data File C:\CHEM32\1\DATA\DEF_LC 2016-04-07 21-02-15\QDY-20036-AD-10-05.D
Sample Name:

===

Acq. Operator : Seq. Line : 29
Acq. Instrument : Instrument 1 Location : Vial 31
Injection Date : 4/8/2016 9:11:23 AM Inj : 1
Inj Volume : 5.000 µl
Acq. Method : C:\CHEM32\1\DATA\DEF_LC 2016-04-07 21-02-15\AD-10-30-0.5.M
Last changed : 1/7/2016 11:07:32 PM
Analysis Method : C:\CHEM32\1\METHODS\AD-01-10-2.0.M
Last changed : 6/26/2016 2:13:09 PM
(modified after loading)

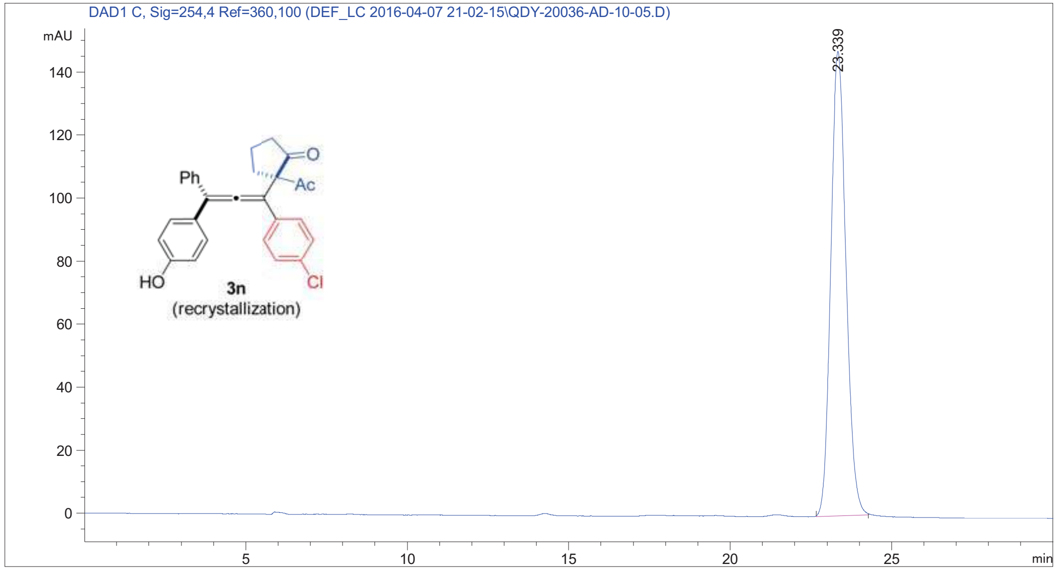

===

Area Percent Report

===

Sorted By : Signal
Multiplier : 1.0000
Dilution : 1.0000
Use Multiplier & Dilution Factor with ISTDs

Signal 1: DAD1 C, Sig=254,4 Ref=360,100

Peak #	RetTime [min]	Type	Width [min]	Area [mAU*s]	Height [mAU]	Area %
1	23.339	BB	0.5087	4807.56592	147.34752	100.0000

Totals : 4807.56592 147.34752

===

*** End of Report ***

Supplementary Figure 185. HPLC spectrum for 3n'

Data File C:\CHEM32\1\DATA\DEF_LC 2016-03-17 13-35-36\QDY-20086-2-AD-10.D
Sample Name:

===

Acq. Operator : Seq. Line : 13
Acq. Instrument : Instrument 1 Location : Vial 32
Injection Date : 3/17/2016 5:12:23 PM Inj : 1
Inj Volume : 5.000 µl
Acq. Method : C:\CHEM32\1\DATA\DEF_LC 2016-03-17 13-35-36\AD-10-30.M
Last changed : 11/30/2015 12:57:24 PM
Analysis Method : C:\CHEM32\1\METHODS\IC-15-30.M
Last changed : 8/20/2016 4:26:25 PM
(modified after loading)
Additional Info : Peak(s) manually integrated

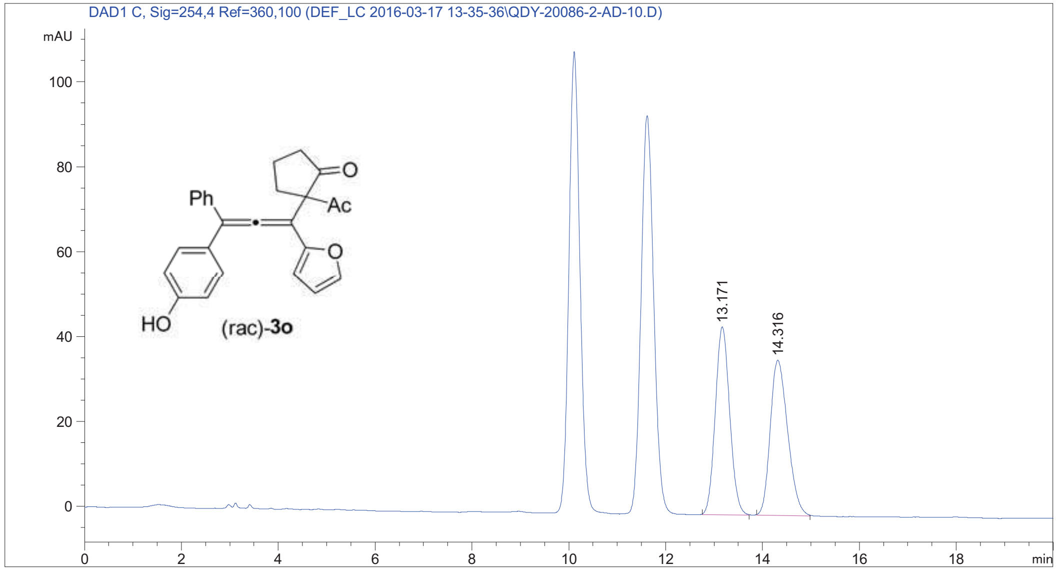

===

Area Percent Report

===

Sorted By : Signal
Multiplier : 1.0000
Dilution : 1.0000
Use Multiplier & Dilution Factor with ISTDs

Signal 1: DAD1 C, Sig=254,4 Ref=360,100

Peak #	RetTime [min]	Type	Width [min]	Area [mAU*s]	Height [mAU]	Area %
1	13.171	BB	0.3233	915.31964	44.26314	50.0637
2	14.316	BB	0.3834	912.99078	36.54956	49.9363

Totals : 1828.31042 80.81270

===

Data File C:\CHEM32\1\DATA\DEF_LC 2016-03-17 13-35-36\QDY-20088-1-ADH-10.D
Sample Name:

===

Acq. Operator : Seq. Line : 70
Acq. Instrument : Instrument 1 Location : Vial 32
Injection Date : 3/18/2016 4:46:31 PM Inj : 1
Inj Volume : 5.000 µl
Acq. Method : C:\CHEM32\1\DATA\DEF_LC 2016-03-17 13-35-36\AD-10-20.M
Last changed : 11/28/2014 10:15:17 AM
Analysis Method : C:\CHEM32\1\METHODS\IC-15-30.M
Last changed : 8/20/2016 4:08:47 PM
(modified after loading)
Additional Info : Peak(s) manually integrated

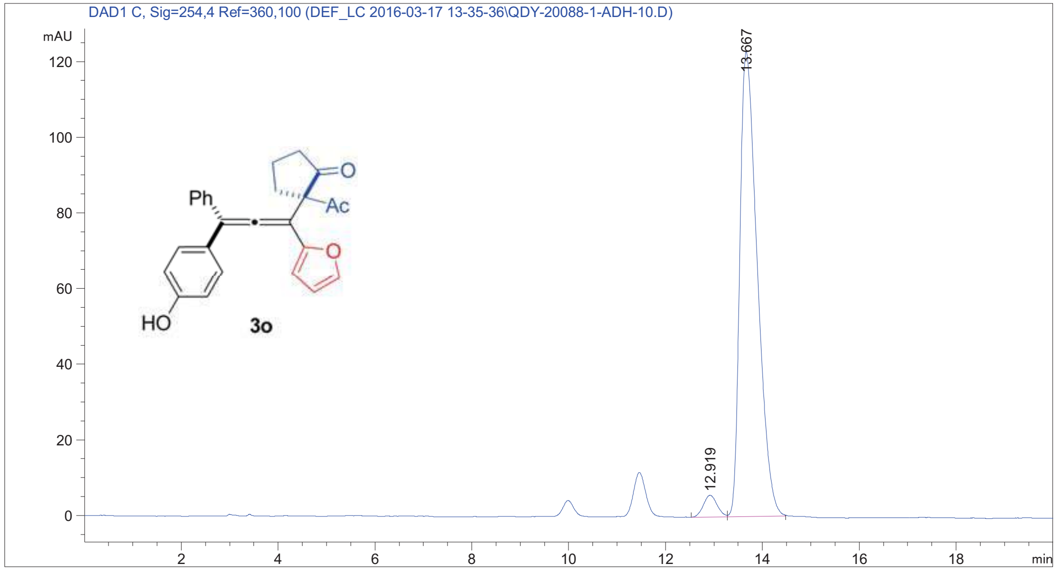

===

Area Percent Report

===

Sorted By : Signal
Multiplier : 1.0000
Dilution : 1.0000
Use Multiplier & Dilution Factor with ISTDs

Signal 1: DAD1 C, Sig=254,4 Ref=360,100

Peak #	RetTime [min]	Type	Width [min]	Area [mAU*s]	Height [mAU]	Area %
1	12.919	BV	0.3037	111.90915	5.73329	3.5141
2	13.667	VB	0.3818	3072.70459	122.87263	96.4859

Totals : 3184.61374 128.60593

===

Supplementary Figure 186. HPLC spectrum for 3o

Data File C:\CHEM32\1\DATA\DEF_LC 2016-03-17 13-35-36\QDY-20086-1-AD-10.D
Sample Name:

===

Acq. Operator : Seq. Line : 16
Acq. Instrument : Instrument 1 Location : Vial 31
Injection Date : 3/17/2016 6:19:13 PM Inj : 1
Inj Volume : 5.000 µl
Acq. Method : C:\CHEM32\1\DATA\DEF_LC 2016-03-17 13-35-36\AD-10-30.M
Last changed : 11/30/2015 12:57:24 PM
Analysis Method : C:\CHEM32\1\METHODS\IC-15-30.M
Last changed : 8/20/2016 4:26:25 PM
(modified after loading)
Additional Info : Peak(s) manually integrated

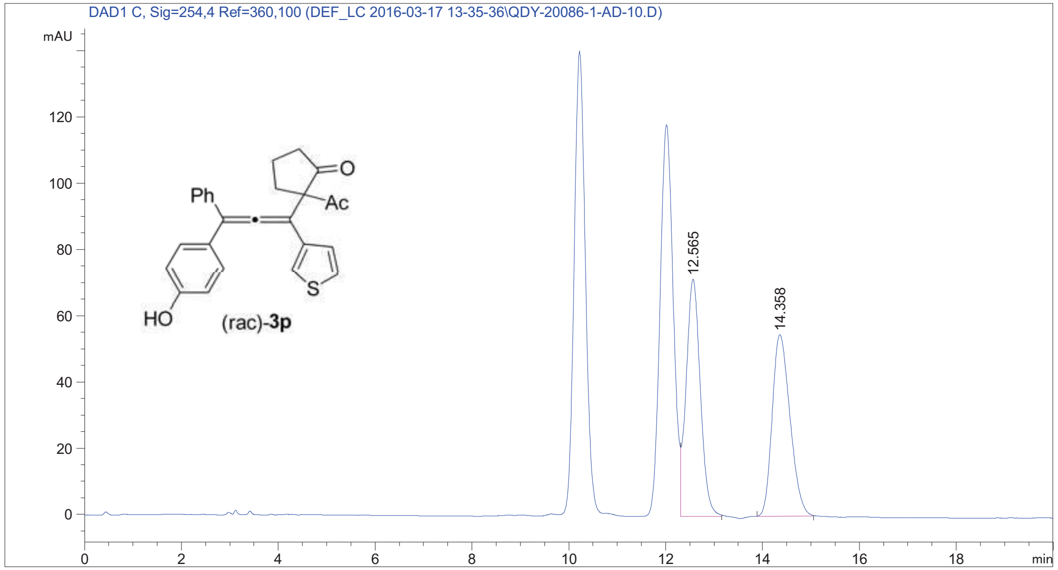

===

Area Percent Report

===

Sorted By : Signal
Multiplier : 1.0000
Dilution : 1.0000
Use Multiplier & Dilution Factor with ISTDs

Signal 1: DAD1 C, Sig=254,4 Ref=360,100

Peak #	RetTime [min]	Type	Width [min]	Area [mAU*s]	Height [mAU]	Area %
1	12.565	VB	0.3114	1455.71436	71.54522	50.8861
2	14.358	BB	0.4009	1405.01794	54.84630	49.1139

Totals : 2860.73230 126.39152

===

Data File C:\CHEM32\1\DATA\DEF_LC 2016-03-24 10-24-21\QDY-20093-1-ADH-10.D
Sample Name:

===

Acq. Operator : Seq. Line : 9
Acq. Instrument : Instrument 1 Location : Vial 32
Injection Date : 3/24/2016 12:34:12 PM Inj : 1
Inj Volume : 5.000 µl
Acq. Method : C:\CHEM32\1\DATA\DEF_LC 2016-03-24 10-24-21\AD-10-30.M
Last changed : 3/24/2016 12:40:12 PM
(modified after loading)
Analysis Method : C:\CHEM32\1\METHODS\IC-15-30.M
Last changed : 8/20/2016 4:26:25 PM
(modified after loading)
Additional Info : Peak(s) manually integrated

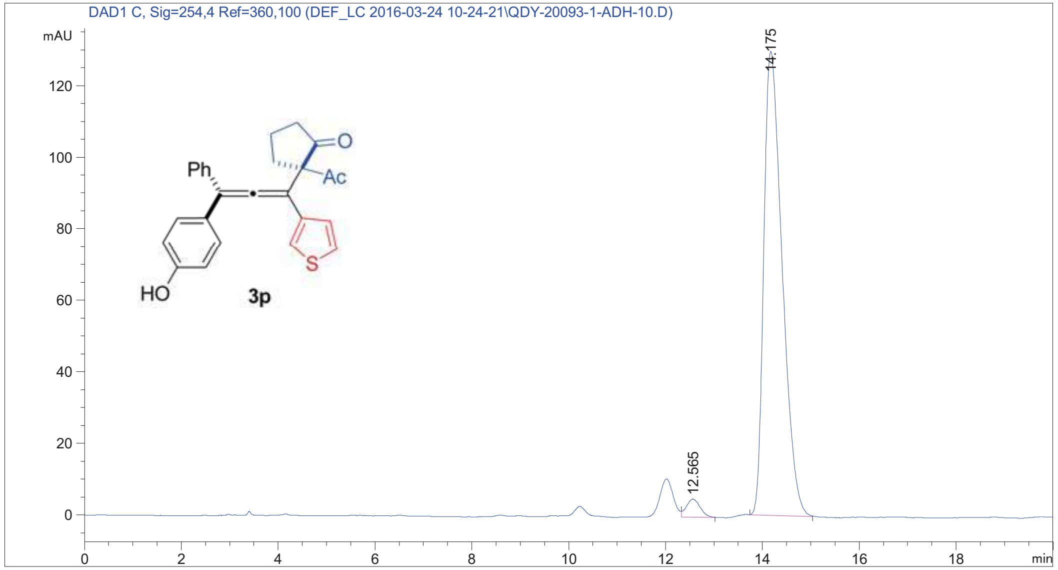

===

Area Percent Report

===

Sorted By : Signal
Multiplier : 1.0000
Dilution : 1.0000
Use Multiplier & Dilution Factor with ISTDs

Signal 1: DAD1 C, Sig=254,4 Ref=360,100

Peak #	RetTime [min]	Type	Width [min]	Area [mAU*s]	Height [mAU]	Area %
1	12.565	VB	0.3027	102.38339	5.08985	2.9131
2	14.175	BB	0.4070	3412.21973	129.67111	97.0869

Totals : 3514.60312 134.76096

Supplementary Figure 187. HPLC spectrum for 3p

Data File C:\CHEM32\1\DATA\DEF_LC 2016-01-15 14-48-07\QDY-20015-1-AD-15.D
Sample Name:

===

Acq. Operator : Seq. Line : 14
Acq. Instrument : Instrument 1 Location : Vial 31
Injection Date : 1/15/2016 7:04:56 PM Inj : 1
Inj Volume : 5.000 µl
Acq. Method : C:\CHEM32\1\DATA\DEF_LC 2016-01-15 14-48-07\AD-15-30.M
Last changed : 1/15/2016 7:21:04 PM
(modified after loading)
Analysis Method : C:\CHEM32\1\METHODS\AD-01-10-2.0.M
Last changed : 6/25/2016 10:45:20 PM
Additional Info : Peak(s) manually integrated

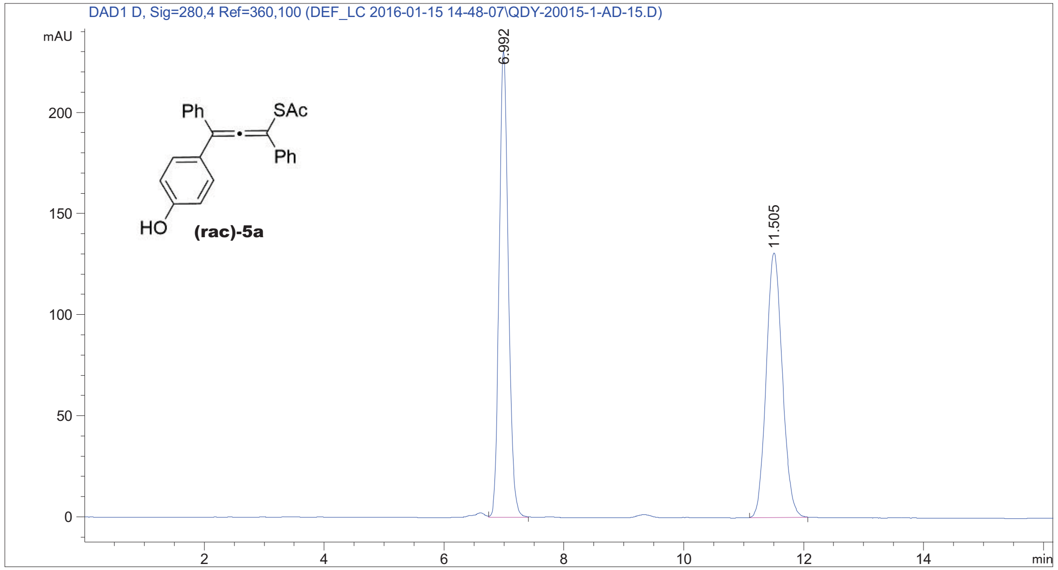

===

Area Percent Report

===

Sorted By : Signal
Multiplier : 1.0000
Dilution : 1.0000
Use Multiplier & Dilution Factor with ISTDs

Signal 1: DAD1 D, Sig=280,4 Ref=360,100

Peak #	RetTime [min]	Type	Width [min]	Area [mAU*s]	Height [mAU]	Area %
1	6.992	VB	0.1642	2452.11304	230.39255	50.0793
2	11.505	BB	0.2897	2444.35181	130.97171	49.9207

Totals : 4896.46484 361.36426

===

Data File C:\CHEM32\1\DATA\DEF_LC 2016-03-17 13-35-36\QDY-20087-3 -AD-15.D
Sample Name:

===

Acq. Operator : Seq. Line : 72
Acq. Instrument : Instrument 1 Location : Vial 39
Injection Date : 3/18/2016 5:15:48 PM Inj : 1
Inj Volume : 5.000 µl
Acq. Method : C:\CHEM32\1\DATA\DEF_LC 2016-03-17 13-35-36\AD-15-22.M
Last changed : 9/15/2015 8:34:07 PM
Analysis Method : C:\CHEM32\1\METHODS\AD-3030.M
Last changed : 6/15/2016 2:43:08 PM
(modified after loading)
Additional Info : Peak(s) manually integrated

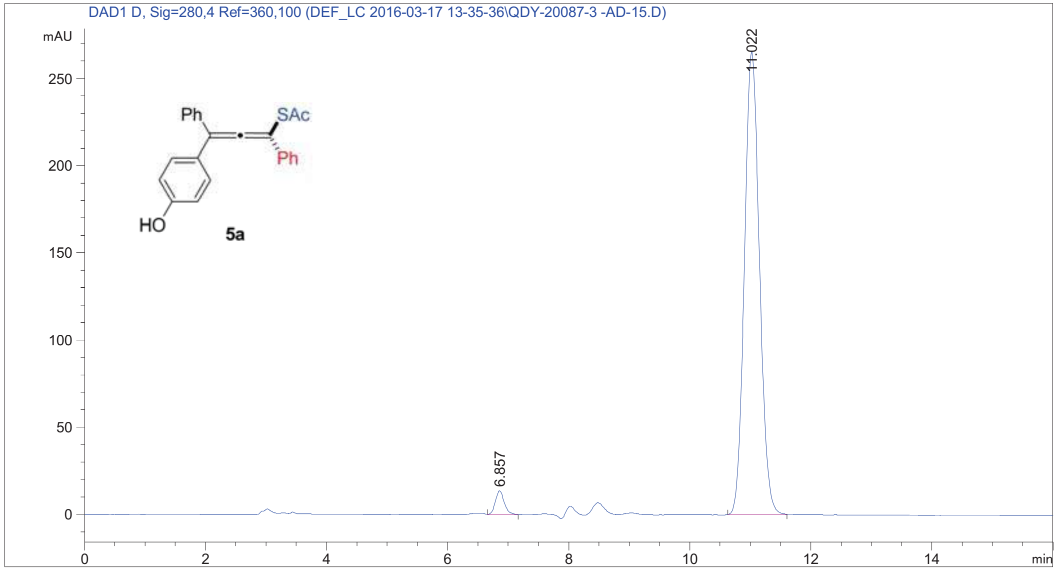

===

Area Percent Report

===

Sorted By : Signal
Multiplier : 1.0000
Dilution : 1.0000
Use Multiplier & Dilution Factor with ISTDs

Signal 1: DAD1 D, Sig=280,4 Ref=360,100

Peak #	RetTime [min]	Type	Width [min]	Area [mAU*s]	Height [mAU]	Area %
1	6.857	VB	0.1572	138.61012	13.56842	2.9376
2	11.022	BB	0.2691	4579.83252	265.64444	97.0624

Totals : 4718.44264 279.21286

===

Supplementary Figure 188. HPLC spectrum for 5a

Data File C:\CHEM32\1\DATA\DEF_LC 2016-03-29 10-50-03\QDY-20098-3-AD-20.D
Sample Name:

===

Acq. Operator : Seq. Line : 17
Acq. Instrument : Instrument 1 Location : Vial 33
Injection Date : 3/29/2016 4:38:58 PM Inj : 1
Inj Volume : 5.000 µl
Acq. Method : C:\CHEM32\1\DATA\DEF_LC 2016-03-29 10-50-03\AD-20-30.M
Last changed : 9/14/2015 3:31:48 PM
Analysis Method : C:\CHEM32\1\METHODS\AD-01-10-2.0.M
Last changed : 6/26/2016 9:16:02 PM
(modified after loading)
Additional Info : Peak(s) manually integrated

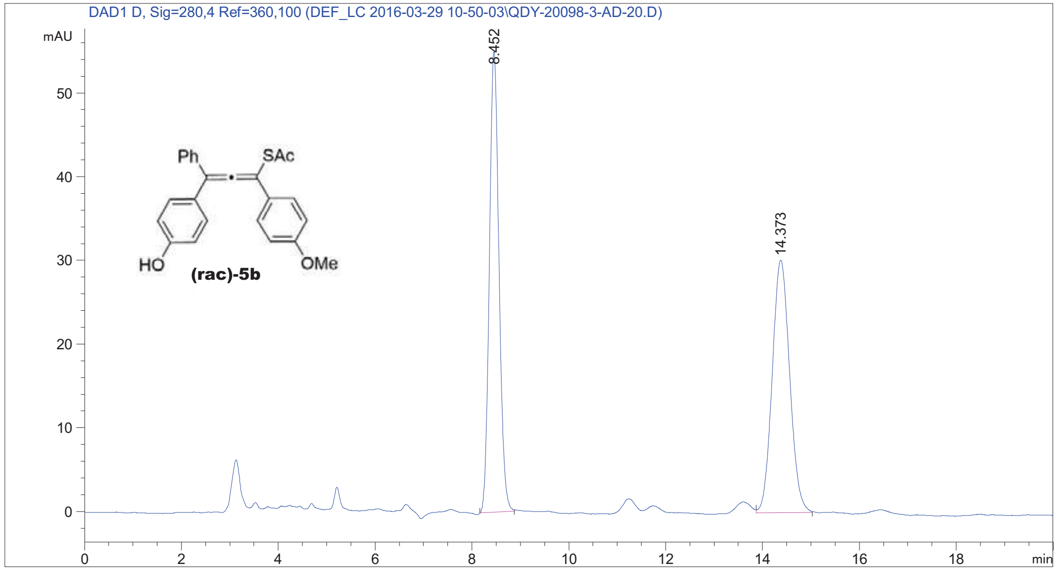

===

Area Percent Report

===

Sorted By : Signal
Multiplier : 1.0000
Dilution : 1.0000
Use Multiplier & Dilution Factor with ISTDs

Signal 1: DAD1 D, Sig=280,4 Ref=360,100

Peak #	RetTime [min]	Type	Width [min]	Area [mAU*s]	Height [mAU]	Area %
1	8.452	BB	0.2145	756.95715	55.01791	50.0821
2	14.373	VB	0.3875	754.47668	30.19891	49.9179

Totals : 1511.43384 85.21682

===

Data File C:\CHEM32\1\DATA\DEF_LC 2016-04-29 18-48-49\QDY-20133-1-ADH-10.D
Sample Name:

===

Acq. Operator : Seq. Line : 12
Acq. Instrument : Instrument 1 Location : Vial 35
Injection Date : 4/29/2016 10:34:54 PM Inj : 1
Inj Volume : 5.000 µl
Acq. Method : C:\CHEM32\1\DATA\DEF_LC 2016-04-29 18-48-49\AD-20-20.M
Last changed : 4/29/2016 10:34:02 PM
(modified after loading)
Analysis Method : C:\CHEM32\1\METHODS\AD-01-10-2.0.M
Last changed : 6/26/2016 9:13:23 PM
(modified after loading)
Additional Info : Peak(s) manually integrated

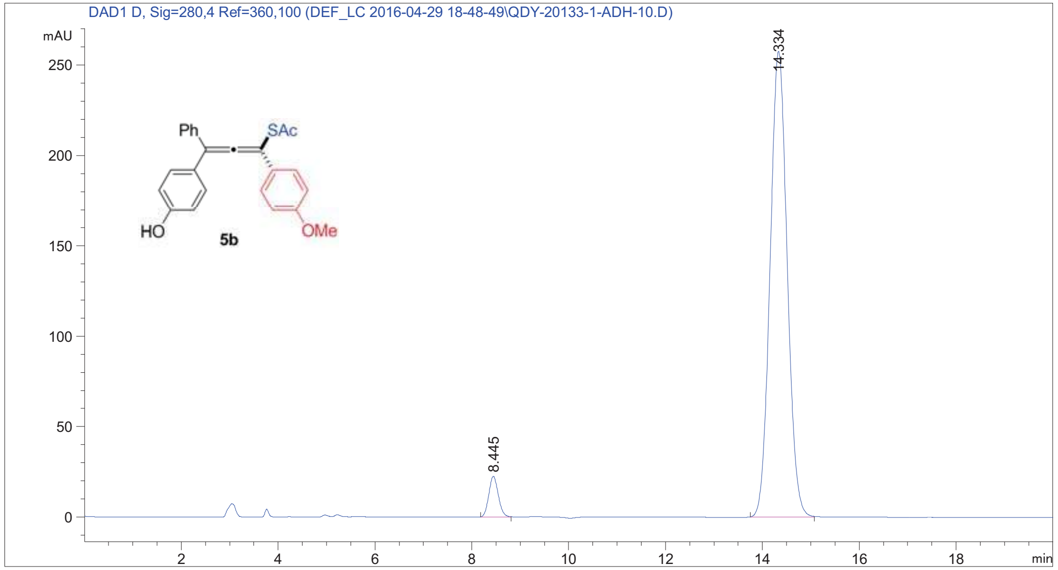

===

Area Percent Report

===

Sorted By : Signal
Multiplier : 1.0000
Dilution : 1.0000
Use Multiplier & Dilution Factor with ISTDs

Signal 1: DAD1 D, Sig=280,4 Ref=360,100

Peak #	RetTime [min]	Type	Width [min]	Area [mAU*s]	Height [mAU]	Area %
1	8.445	BB	0.2113	307.34174	22.50527	4.6406
2	14.334	BB	0.3800	6315.47900	257.61475	95.3594

Totals : 6622.82074 280.12002

Supplementary Figure 189. HPLC spectrum for 5b

Data File C:\CHEM32\1\DATA\DEF_LC 2016-05-08 12-45-48\QDY-20144-1-ADH-15.D
Sample Name:

===

Acq. Operator : Seq. Line : 33
Acq. Instrument : Instrument 1 Location : Vial 31
Injection Date : 5/9/2016 12:25:00 AM Inj : 1
Inj Volume : 5.000 µl
Acq. Method : C:\CHEM32\1\DATA\DEF_LC 2016-05-08 12-45-48\AD-15-22.M
Last changed : 9/15/2015 8:34:07 PM
Analysis Method : C:\CHEM32\1\METHODS\AD-01-10-2.0.M
Last changed : 6/26/2016 8:57:24 PM
(modified after loading)
Additional Info : Peak(s) manually integrated

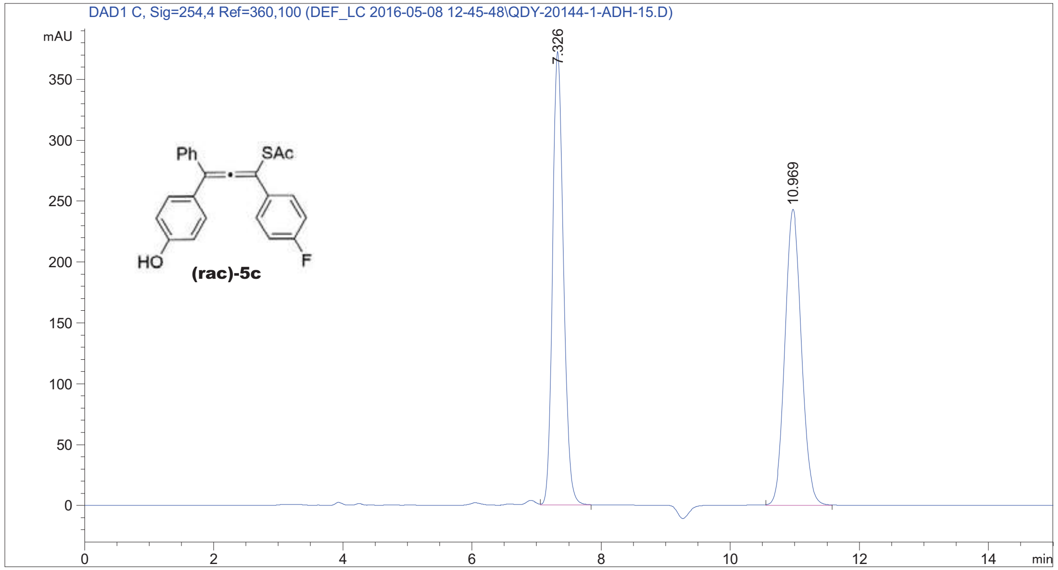

===

Area Percent Report

===

Sorted By : Signal
Multiplier : 1.0000
Dilution : 1.0000
Use Multiplier & Dilution Factor with ISTDs

Signal 1: DAD1 C, Sig=254,4 Ref=360,100

Peak #	RetTime [min]	Type	Width [min]	Area [mAU*s]	Height [mAU]	Area %
1	7.326	VB	0.1828	4366.85938	372.40533	50.2199
2	10.969	BB	0.2757	4328.62256	243.13440	49.7801

Totals : 8695.48193 615.53973

===

Data File C:\CHEM32\1\DATA\DEF_LC 2016-05-10 10-25-50\QDY-20144-4-ADH-15.D
Sample Name:

===

Acq. Operator : Seq. Line : 21
Acq. Instrument : Instrument 1 Location : Vial 33
Injection Date : 5/10/2016 6:18:08 PM Inj : 1
Inj Volume : 5.000 µl
Different Inj Volume from Sequence ! Actual Inj Volume : 1.000 µl
Acq. Method : C:\CHEM32\1\DATA\DEF_LC 2016-05-10 10-25-50\AD-15-22.M
Last changed : 5/10/2016 6:24:54 PM
(modified after loading)
Analysis Method : C:\CHEM32\1\METHODS\AD-01-10-2.0.M
Last changed : 6/26/2016 8:55:19 PM
(modified after loading)
Additional Info : Peak(s) manually integrated

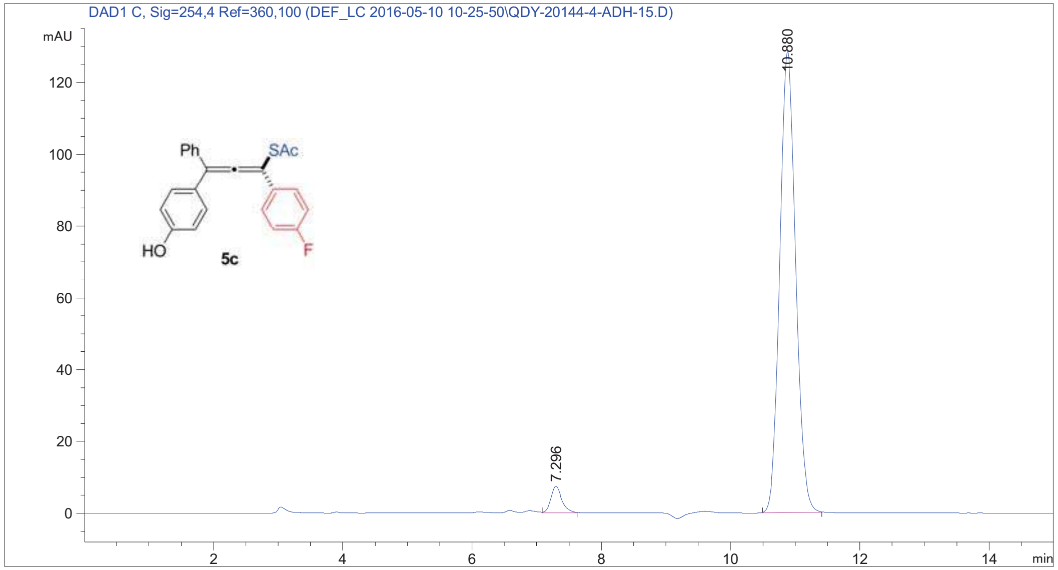

===

Area Percent Report

===

Sorted By : Signal
Multiplier : 1.0000
Dilution : 1.0000
Use Multiplier & Dilution Factor with ISTDs

Signal 1: DAD1 C, Sig=254,4 Ref=360,100

Peak #	RetTime [min]	Type	Width [min]	Area [mAU*s]	Height [mAU]	Area %
1	7.296	BB	0.1765	87.03292	7.43725	3.8065
2	10.880	BB	0.2657	2199.37378	128.49953	96.1935

Totals : 2286.40670 135.93677

Supplementary Figure 190. HPLC spectrum for 5c

Data File C:\CHEM32\1\DATA\DEF_LC 2016-04-29 18-48-49\QDY-20134-1-AD-15.D
Sample Name:

===

Acq. Operator   :   Seq. Line : 18
Acq. Instrument : Instrument 1   Location : Vial 31
Injection Date : 4/30/2016 12:42:59 AM   Inj :   1
                                               Inj Volume : 5.000 µl
Acq. Method   : C:\CHEM32\1\DATA\DEF_LC 2016-04-29 18-48-49\AD-15-30.M
Last changed   : 4/30/2016 1:07:01 AM
                  (modified after loading)
Analysis Method : C:\CHEM32\1\METHODS\AD-01-10-2.0.M
Last changed   : 6/26/2016 9:00:17 PM
                  (modified after loading)
Additional Info : Peak(s) manually integrated

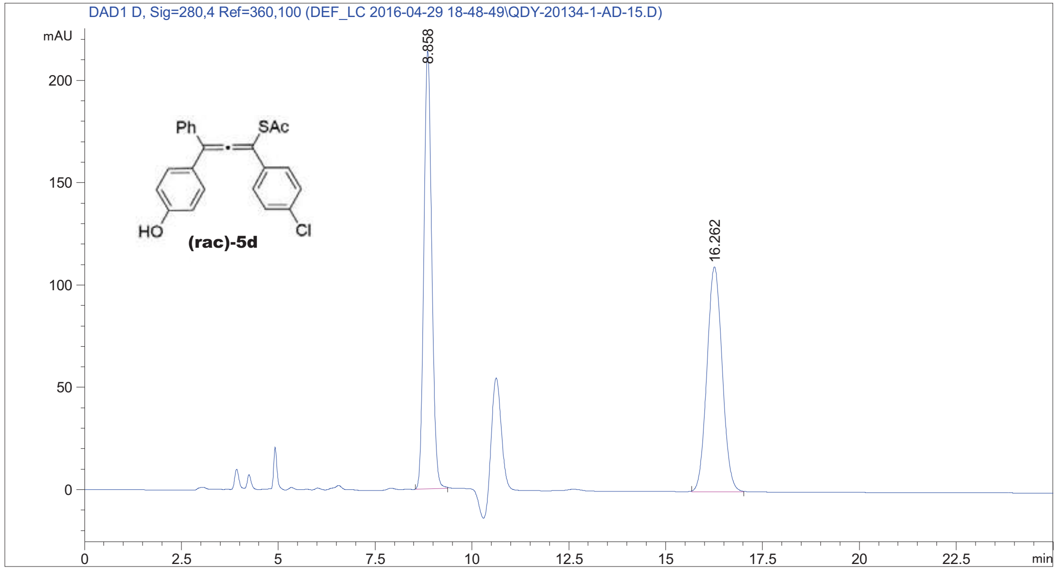

===

Area Percent Report

===

Sorted By : Signal
Multiplier : 1.0000
Dilution : 1.0000
Use Multiplier & Dilution Factor with ISTDs

Signal 1: DAD1 D, Sig=280,4 Ref=360,100

Peak #	RetTime [min]	Type	Width [min]	Area [mAU*s]	Height [mAU]	Area %
1	8.858	BB	0.2209	3023.58618	213.92877	50.0682
2	16.262	BB	0.4280	3015.34619	109.93711	49.9318

Totals :	6038.93237	323.86588

Data File C:\CHEM32\1\DATA\DEF_LC 2016-05-12 09-09-58\QDY-20145-2-AD-15.D
Sample Name:

===

Acq. Operator   :   Seq. Line : 10
Acq. Instrument : Instrument 1   Location : Vial 31
Injection Date : 5/12/2016 11:40:46 AM   Inj :   1
                                               Inj Volume : 5.000 µl
Acq. Method   : C:\CHEM32\1\DATA\DEF_LC 2016-05-12 09-09-58\AD-15-30.M
Last changed   : 5/12/2016 11:39:53 AM
                  (modified after loading)
Analysis Method : C:\CHEM32\1\METHODS\AD-01-10-2.0.M
Last changed   : 6/26/2016 9:00:17 PM
                  (modified after loading)
Additional Info : Peak(s) manually integrated

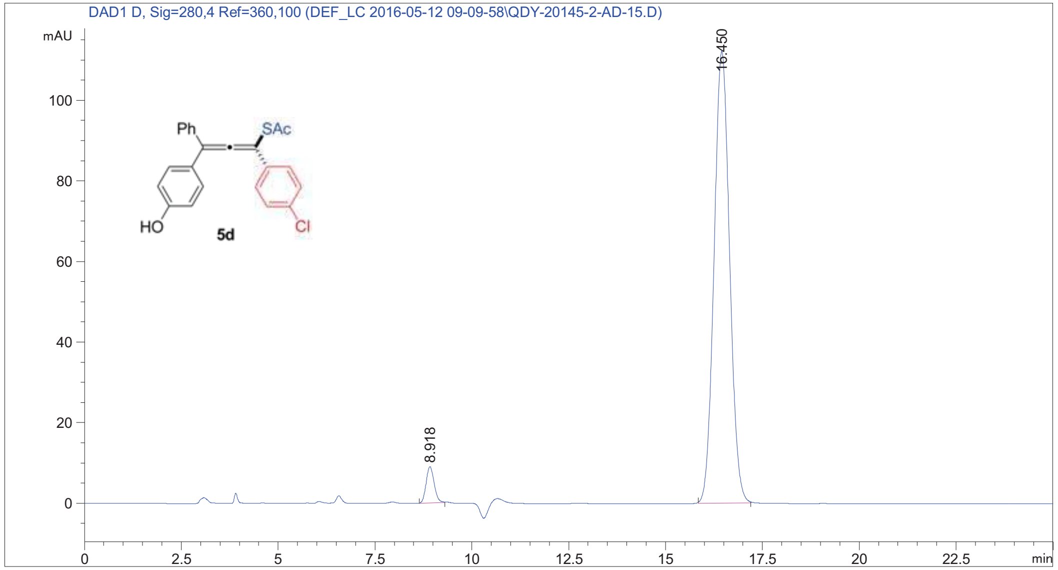

===

Area Percent Report

===

Sorted By : Signal
Multiplier : 1.0000
Dilution : 1.0000
Use Multiplier & Dilution Factor with ISTDs

Signal 1: DAD1 D, Sig=280,4 Ref=360,100

Peak #	RetTime [min]	Type	Width [min]	Area [mAU*s]	Height [mAU]	Area %
1	8.918	BB	0.2243	130.28934	9.02971	4.0040
2	16.450	BB	0.4331	3123.68799	112.10854	95.9960

Totals :	3253.97733	121.13826

Supplementary Figure 191. HPLC spectrum for 5d

Data File C:\CHEM32\1\DATA\DEF_LC 2016-03-05 14-20-31\QDY-20069-3-AD-10.D
Sample Name:

===

Acq. Operator : Seq. Line : 16
Acq. Instrument : Instrument 1 Location : Vial 33
Injection Date : 3/5/2016 7:28:45 PM Inj : 1
Inj Volume : 5.000 µl
Acq. Method : C:\CHEM32\1\DATA\DEF_LC 2016-03-05 14-20-31\AD-10-20.M
Last changed : 3/5/2016 7:11:35 PM
(modified after loading)
Analysis Method : C:\CHEM32\1\METHODS\AD-01-10-2.0.M
Last changed : 6/26/2016 2:31:40 PM
(modified after loading)
Additional Info : Peak(s) manually integrated

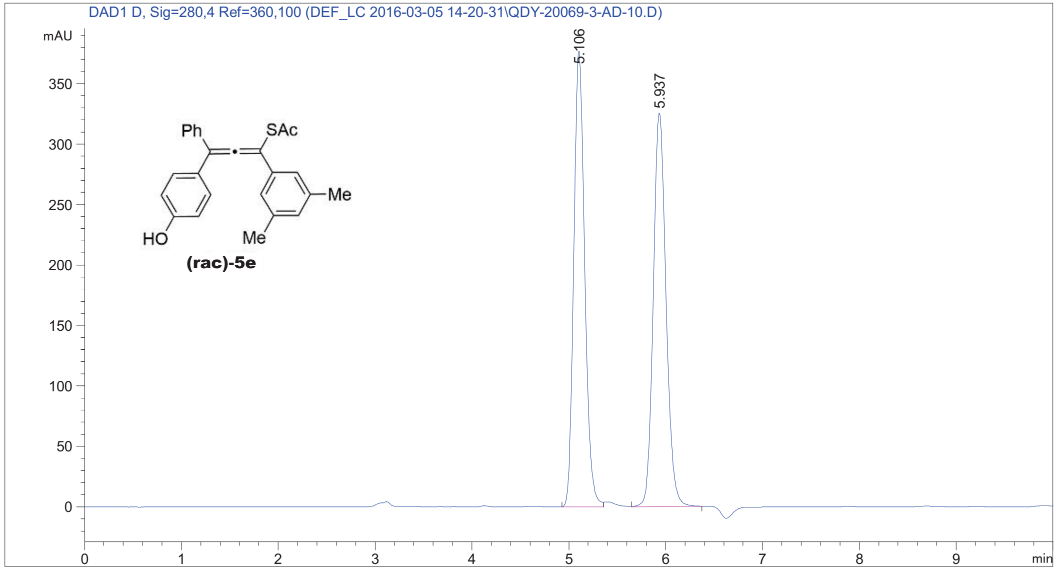

===

Area Percent Report

===

Sorted By : Signal
Multiplier : 1.0000
Dilution : 1.0000
Use Multiplier & Dilution Factor with ISTDs

Signal 1: DAD1 D, Sig=280,4 Ref=360,100

Peak #	RetTime [min]	Type	Width [min]	Area [mAU*s]	Height [mAU]	Area %
1	5.106	BV	0.1192	2947.91675	377.19528	49.7389
2	5.937	VB	0.1407	2978.87012	325.63467	50.2611

Totals : 5926.78687 702.82996

Data File C:\CHEM32\1\DATA\DEF_LC 2016-05-10 10-25-50\QDY-20145-1--AD-10.D
Sample Name:

===

Acq. Operator : Seq. Line : 87
Acq. Instrument : Instrument 1 Location : Vial 31
Injection Date : 5/11/2016 6:35:56 PM Inj : 1
Inj Volume : 5.000 µl
Acq. Method : C:\CHEM32\1\DATA\DEF_LC 2016-05-10 10-25-50\AD-10-20.M
Last changed : 11/28/2014 10:15:17 AM
Analysis Method : C:\CHEM32\1\METHODS\AD-01-10-2.0.M
Last changed : 6/26/2016 2:31:40 PM
(modified after loading)
Additional Info : Peak(s) manually integrated

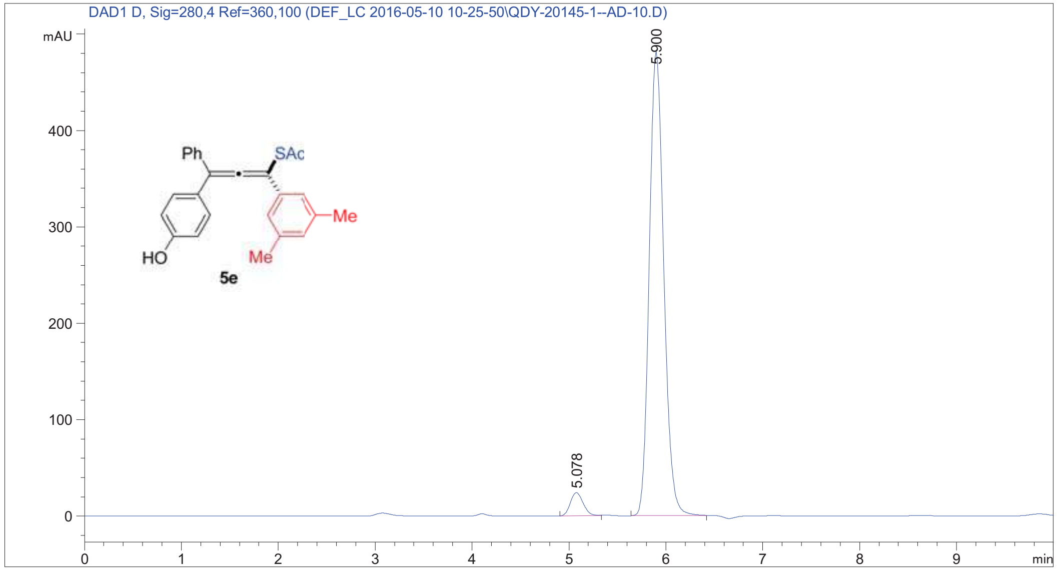

===

Area Percent Report

===

Sorted By : Signal
Multiplier : 1.0000
Dilution : 1.0000
Use Multiplier & Dilution Factor with ISTDs

Signal 1: DAD1 D, Sig=280,4 Ref=360,100

Peak #	RetTime [min]	Type	Width [min]	Area [mAU*s]	Height [mAU]	Area %
1	5.078	BB	0.1423	219.88640	24.14296	4.2105
2	5.900	BB	0.1612	5002.39453	481.36807	95.7895

Totals : 5222.28093 505.51103

===

Supplementary Figure 192. HPLC spectrum for 5e

Data File C:\CHEM32\1\DATA\DEF_LC 2016-04-09 10-10-22\QDY-20109-1.D
Sample Name:

===

Acq. Operator : Seq. Line : 2
Acq. Instrument : Instrument 1 Location : Vial 31
Injection Date : 4/9/2016 10:24:09 AM Inj : 1
Inj Volume : 5.000 µl
Acq. Method : C:\CHEM32\1\DATA\DEF_LC 2016-04-09 10-10-22\AD-15-30.M
Last changed : 4/9/2016 10:50:56 AM
(modified after loading)
Analysis Method : C:\CHEM32\1\METHODS\AD-01-10-2.0.M
Last changed : 6/26/2016 9:19:48 PM
(modified after loading)
Additional Info : Peak(s) manually integrated

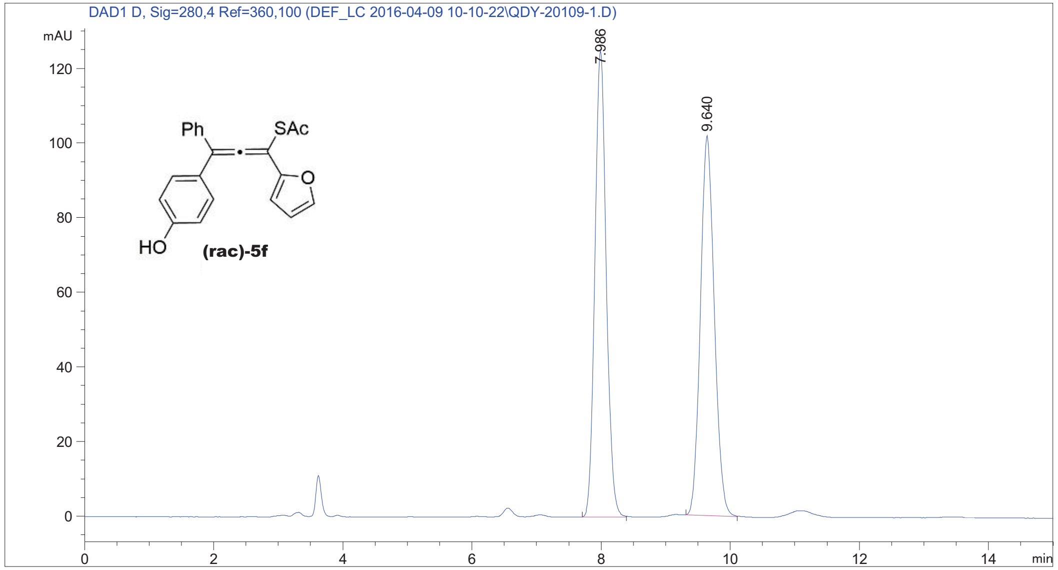

===

Area Percent Report

===

Sorted By : Signal
Multiplier : 1.0000
Dilution : 1.0000
Use Multiplier & Dilution Factor with ISTDs

Signal 1: DAD1 D, Sig=280,4 Ref=360,100

Peak #	RetTime [min]	Type	Width [min]	Area [mAU*s]	Height [mAU]	Area %
1	7.986	BB	0.1911	1530.46655	124.77222	50.0747
2	9.640	BB	0.2329	1525.90210	101.77819	49.9253

Totals : 3056.36865 226.55042

Data File C:\CHEM32\1\DATA\DEF_LC 2016-04-17 00-38-55\QDY-20119-1-ADH-15.D
Sample Name:

===

Acq. Operator : Seq. Line : 4
Acq. Instrument : Instrument 1 Location : Vial 31
Injection Date : 4/17/2016 1:13:12 AM Inj : 1
Inj Volume : 5.000 µl
Acq. Method : C:\CHEM32\1\DATA\DEF_LC 2016-04-17 00-38-55\AD-15-22.M
Last changed : 9/15/2015 8:34:07 PM
Analysis Method : C:\CHEM32\1\METHODS\AD-01-10-2.0.M
Last changed : 6/26/2016 9:19:48 PM
(modified after loading)
Additional Info : Peak(s) manually integrated

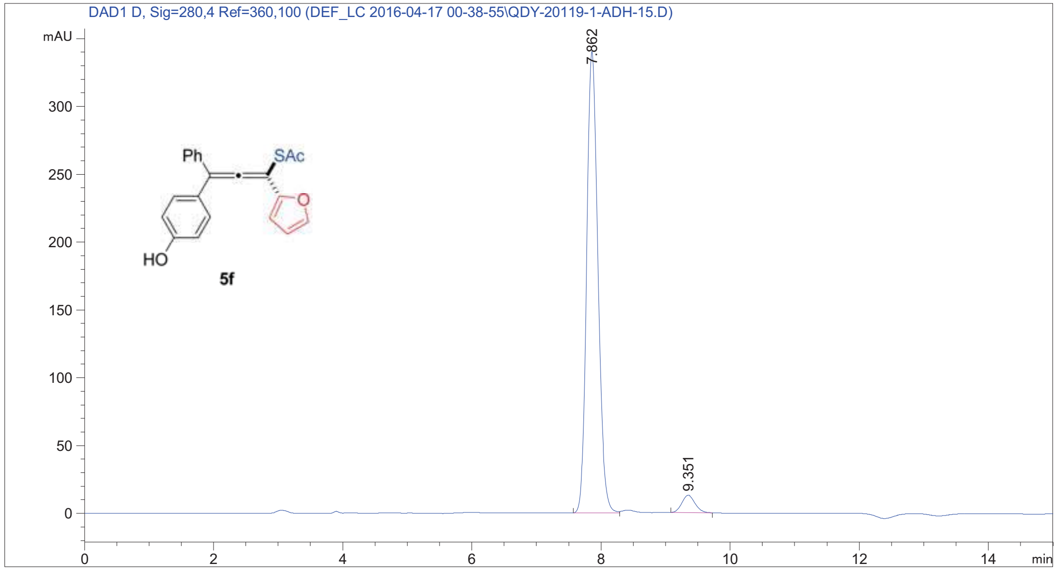

===

Area Percent Report

===

Sorted By : Signal
Multiplier : 1.0000
Dilution : 1.0000
Use Multiplier & Dilution Factor with ISTDs

Signal 1: DAD1 D, Sig=280,4 Ref=360,100

Peak #	RetTime [min]	Type	Width [min]	Area [mAU*s]	Height [mAU]	Area %
1	7.862	BB	0.1866	4096.49463	340.03119	95.6900
2	9.351	BB	0.2203	184.51270	12.94315	4.3100

Totals : 4281.00732 352.97434

Supplementary Figure 193. HPLC spectrum for 5f

===

Data File C:\CHEM32\1\DATA\DEF_LC 2016-03-05 14-20-31\QDY-20069-1-AD-15.D
Sample Name:

===

Acq. Operator : Seq. Line : 6
Acq. Instrument : Instrument 1 Location : Vial 31
Injection Date : 3/5/2016 3:54:17 PM Inj : 1
Inj Volume : 5.000 µl
Acq. Method : C:\CHEM32\1\DATA\DEF_LC 2016-03-05 14-20-31\AD-15-22.M
Last changed : 3/5/2016 4:11:52 PM
(modified after loading)
Analysis Method : C:\CHEM32\1\METHODS\AD-01-10-2.0.M
Last changed : 6/26/2016 9:27:49 PM
(modified after loading)
Additional Info : Peak(s) manually integrated

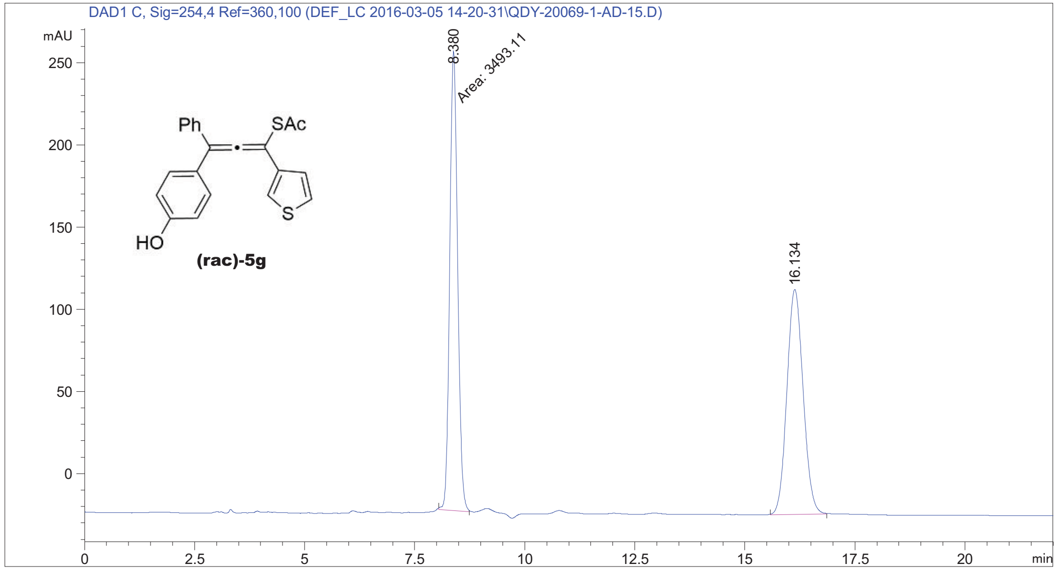

===

Area Percent Report

===

Sorted By : Signal
Multiplier : 1.0000
Dilution : 1.0000
Use Multiplier & Dilution Factor with ISTDs

Signal 1: DAD1 C, Sig=254,4 Ref=360,100

Peak #	RetTime [min]	Type	Width [min]	Area [mAU*s]	Height [mAU]	Area %
1	8.380	MM	0.2083	3493.10522	279.45023	49.9186
2	16.134	BB	0.4008	3504.49414	136.82832	50.0814

Totals : 6997.59937 416.27855

Data File C:\CHEM32\1\DATA\DEF_LC 2016-04-14 10-01-26\QDY-20115-1-ADH--15.D
Sample Name:

===

Acq. Operator : Seq. Line : 35
Acq. Instrument : Instrument 1 Location : Vial 31
Injection Date : 4/14/2016 9:48:14 PM Inj : 1
Inj Volume : 5.000 µl
Acq. Method : C:\CHEM32\1\DATA\DEF_LC 2016-04-14 10-01-26\AD-15-22.M
Last changed : 9/15/2015 8:34:07 PM
Analysis Method : C:\CHEM32\1\METHODS\AD-01-10-2.0.M
Last changed : 6/26/2016 9:27:49 PM
(modified after loading)
Additional Info : Peak(s) manually integrated

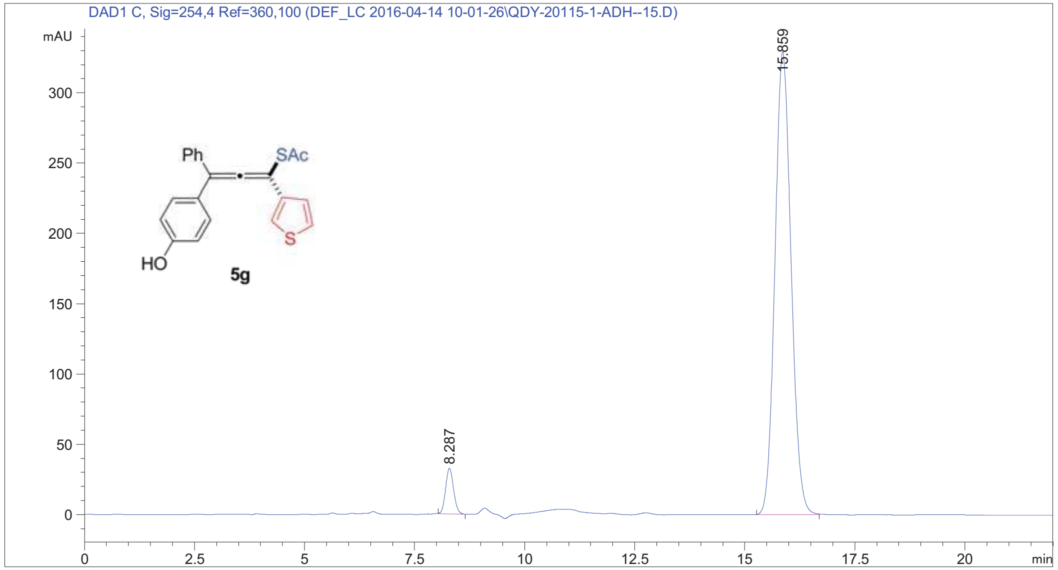

===

Area Percent Report

===

Sorted By : Signal
Multiplier : 1.0000
Dilution : 1.0000
Use Multiplier & Dilution Factor with ISTDs

Signal 1: DAD1 C, Sig=254,4 Ref=360,100

Peak #	RetTime [min]	Type	Width [min]	Area [mAU*s]	Height [mAU]	Area %
1	8.287	BB	0.1976	412.45316	32.60120	4.6671
2	15.859	BB	0.3966	8424.96777	329.19797	95.3329

Totals : 8837.42093 361.79917

===

Supplementary Figure 194. HPLC spectrum for 5g

Data File C:\CHEM32\1\DATA\DEF_LC 2016-04-16 10-10-47\QDY-20113-4-AD-20.D
Sample Name:

===

Acq. Operator : Seq. Line : 7
Acq. Instrument : Instrument 1 Location : Vial 31
Injection Date : 4/16/2016 11:48:40 AM Inj : 1
Inj Volume : 5.000 µl
Acq. Method : C:\CHEM32\1\DATA\DEF_LC 2016-04-16 10-10-47\AD-20-20.M
Last changed : 7/4/2015 10:27:19 AM
Analysis Method : C:\CHEM32\1\METHODS\AD-01-10-2.0.M
Last changed : 6/26/2016 8:40:34 PM
(modified after loading)
Additional Info : Peak(s) manually integrated

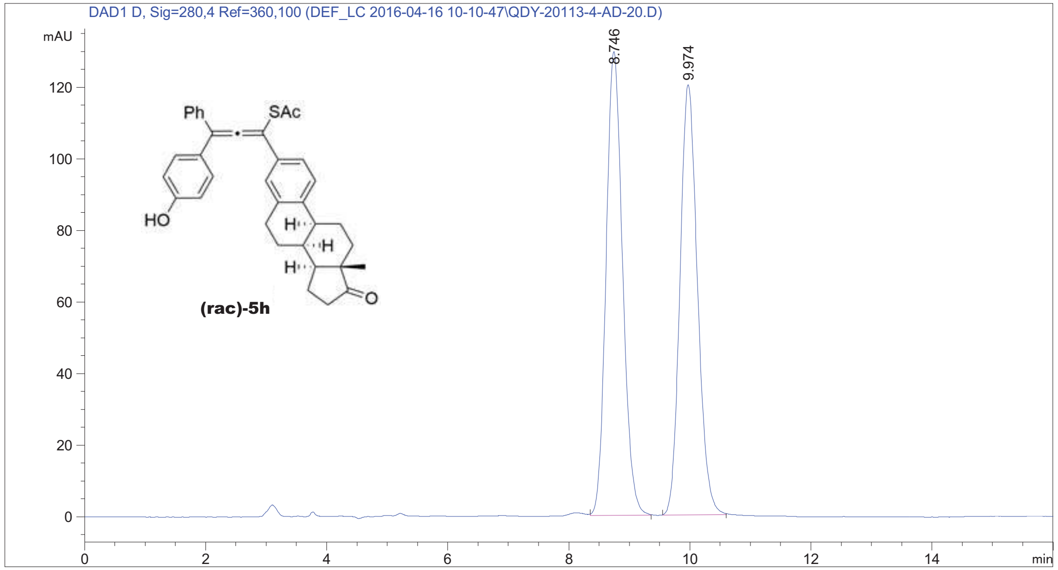

===

Area Percent Report

===

Sorted By : Signal
Multiplier : 1.0000
Dilution : 1.0000
Use Multiplier & Dilution Factor with ISTDs

Signal 1: DAD1 D, Sig=280,4 Ref=360,100

Peak #	RetTime [min]	Type	Width [min]	Area [mAU*s]	Height [mAU]	Area %
1	8.746	BB	0.2927	2451.85254	129.59225	50.4080
2	9.974	BB	0.3102	2412.16650	120.15545	49.5920

Totals : 4864.01904 249.74770

===

Data File C:\CHEM32\1\DATA\DEF_LC 2016-04-16 10-10-47\QDY-20117-4-AD-20.D
Sample Name:

===

Acq. Operator : Seq. Line : 6
Acq. Instrument : Instrument 1 Location : Vial 34
Injection Date : 4/16/2016 11:27:34 AM Inj : 1
Inj Volume : 5.000 µl
Acq. Method : C:\CHEM32\1\DATA\DEF_LC 2016-04-16 10-10-47\AD-20-20.M
Last changed : 7/4/2015 10:27:19 AM
Analysis Method : C:\CHEM32\1\METHODS\AD-01-10-2.0.M
Last changed : 6/26/2016 8:40:34 PM
(modified after loading)
Additional Info : Peak(s) manually integrated

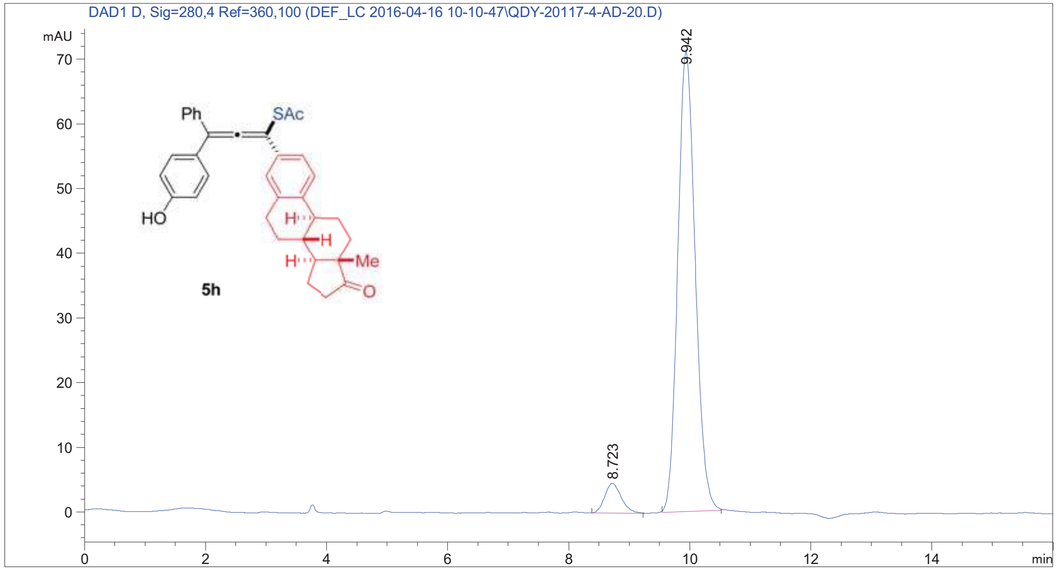

===

Area Percent Report

===

Sorted By : Signal
Multiplier : 1.0000
Dilution : 1.0000
Use Multiplier & Dilution Factor with ISTDs

Signal 1: DAD1 D, Sig=280,4 Ref=360,100

Peak #	RetTime [min]	Type	Width [min]	Area [mAU*s]	Height [mAU]	Area %
1	8.723	BB	0.2887	86.59442	4.57787	5.7274
2	9.942	BB	0.3099	1425.34900	71.10870	94.2726

Totals : 1511.94342 75.68657

===

Supplementary Figure 195. HPLC spectrum for 5h

Data File C:\CHEM32\1\DATA\DEF_LC 2016-01-26 23-17-00\QDY-20033-2-AD-10.D
Sample Name:

===

Acq. Operator : Seq. Line : 4
Acq. Instrument : Instrument 1 Location : Vial 32
Injection Date : 1/27/2016 12:12:58 AM Inj : 1
Inj Volume : 5.000 µl
Acq. Method : C:\CHEM32\1\DATA\DEF_LC 2016-01-26 23-17-00\AD-10-30.M
Last changed : 11/30/2015 12:57:24 PM
Analysis Method : C:\CHEM32\1\METHODS\AD-01-10-2.0.M
Last changed : 6/27/2016 11:28:15 PM
(modified after loading)
Additional Info : Peak(s) manually integrated

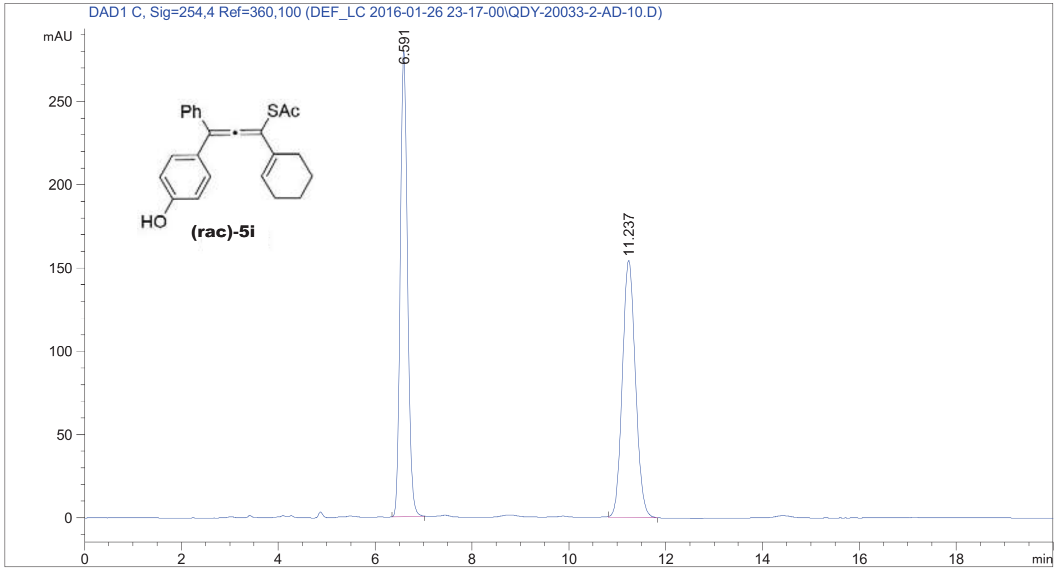

===

Area Percent Report

===

Sorted By : Signal
Multiplier : 1.0000
Dilution : 1.0000
Use Multiplier & Dilution Factor with ISTDs

Signal 1: DAD1 C, Sig=254,4 Ref=360,100

Peak #	RetTime [min]	Type	Width [min]	Area [mAU*s]	Height [mAU]	Area %
1	6.591	BB	0.1619	2919.46143	279.40152	50.0987
2	11.237	BB	0.2918	2907.95361	154.31596	49.9013

Totals : 5827.41504 433.71748

===

Data File C:\CHEM32\1\DATA\DEF_LC 2016-05-07 09-30-19\QDY-20138-2-ADH-10.D
Sample Name:

===

Acq. Operator : Seq. Line : 45
Acq. Instrument : Instrument 1 Location : Vial 32
Injection Date : 5/7/2016 11:46:26 PM Inj : 1
Inj Volume : 5.000 µl
Acq. Method : C:\CHEM32\1\DATA\DEF_LC 2016-05-07 09-30-19\AD-10-20.M
Last changed : 11/28/2014 10:15:17 AM
Analysis Method : C:\CHEM32\1\METHODS\AD-01-10-2.0.M
Last changed : 6/26/2016 2:13:09 PM
(modified after loading)
Additional Info : Peak(s) manually integrated

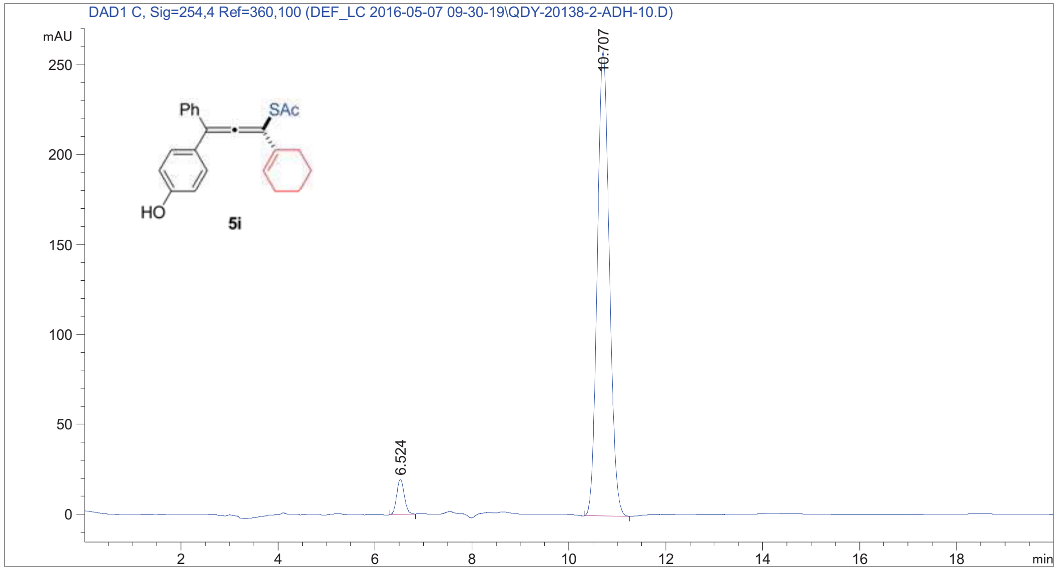

===

Area Percent Report

===

Sorted By : Signal
Multiplier : 1.0000
Dilution : 1.0000
Use Multiplier & Dilution Factor with ISTDs

Signal 1: DAD1 C, Sig=254,4 Ref=360,100

Peak #	RetTime [min]	Type	Width [min]	Area [mAU*s]	Height [mAU]	Area %
1	6.524	BB	0.1698	211.17703	19.58147	4.5187
2	10.707	BB	0.2676	4462.21826	258.18164	95.4813

Totals : 4673.39529 277.76311

===

Supplementary Figure 196. HPLC spectrum for 5i

Data File C:\CHEM32\1\DATA\DEF_LC 2016-03-05 14-20-31\QDY-20069-2--AD-10.D
Sample Name:

===

Acq. Operator : Seq. Line : 15
Acq. Instrument : Instrument 1 Location : Vial 32
Injection Date : 3/5/2016 7:12:41 PM Inj : 1
Inj Volume : 5.000 µl
Acq. Method : C:\CHEM32\1\DATA\DEF_LC 2016-03-05 14-20-31\AD-10-20.M
Last changed : 3/5/2016 7:11:35 PM
(modified after loading)
Analysis Method : C:\CHEM32\1\METHODS\AD-01-10-2.0.M
Last changed : 6/26/2016 9:35:27 PM
(modified after loading)
Additional Info : Peak(s) manually integrated

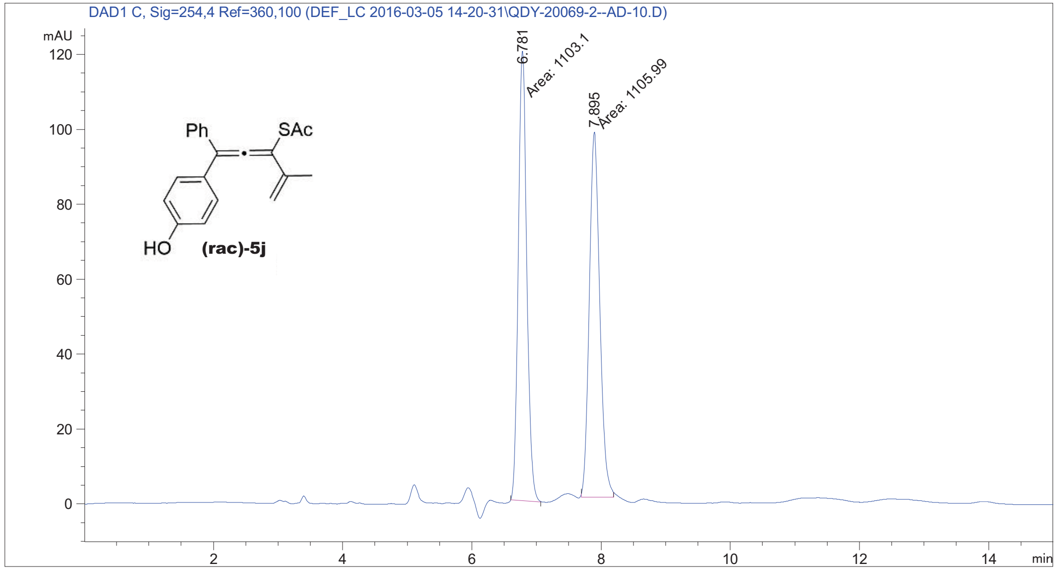

===

Area Percent Report

===

Sorted By : Signal
Multiplier : 1.0000
Dilution : 1.0000
Use Multiplier & Dilution Factor with ISTDs

Signal 1: DAD1 C, Sig=254,4 Ref=360,100

Peak #	RetTime [min]	Type	Width [min]	Area [mAU*s]	Height [mAU]	Area %
1	6.781	MM	0.1531	1103.10168	120.10813	49.9346
2	7.895	MM	0.1890	1105.98950	97.53231	50.0654

Totals : 2209.09119 217.64044

Data File C:\CHEM32\1\DATA\DEF_LC 2016-04-14 10-01-26\QDY-20115-2-AD--10.D
Sample Name:

===

Acq. Operator : Seq. Line : 31
Acq. Instrument : Instrument 1 Location : Vial 32
Injection Date : 4/14/2016 8:33:53 PM Inj : 1
Inj Volume : 5.000 µl
Acq. Method : C:\CHEM32\1\DATA\DEF_LC 2016-04-14 10-01-26\AD-10-25.M
Last changed : 4/14/2016 8:49:15 PM
(modified after loading)
Analysis Method : C:\CHEM32\1\METHODS\AD-01-10-2.0.M
Last changed : 6/28/2016 12:41:50 PM
(modified after loading)
Additional Info : Peak(s) manually integrated

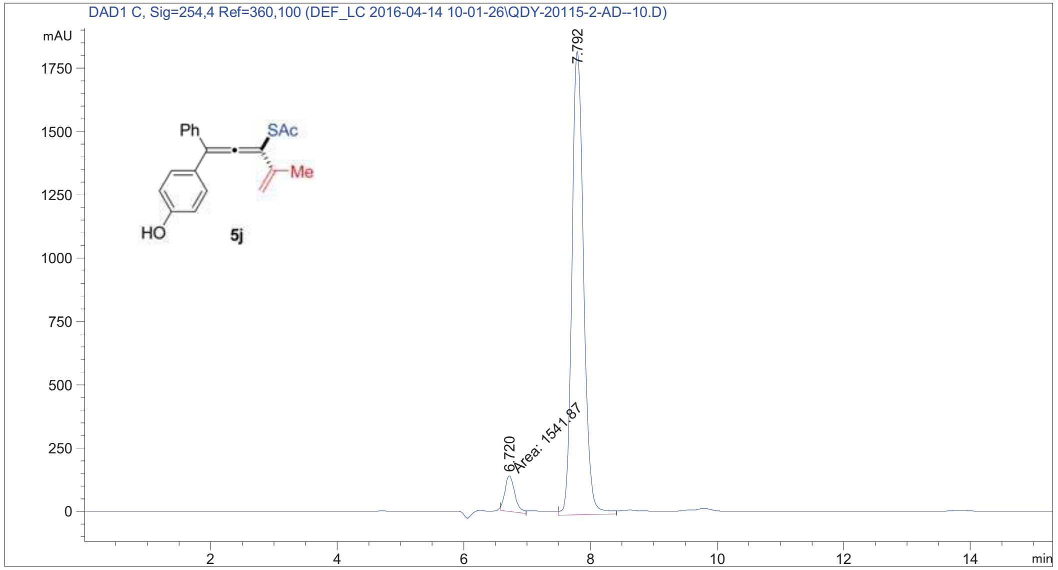

===

Area Percent Report

===

Sorted By : Signal
Multiplier : 1.0000
Dilution : 1.0000
Use Multiplier & Dilution Factor with ISTDs

Signal 1: DAD1 C, Sig=254,4 Ref=360,100

Peak #	RetTime [min]	Type	Width [min]	Area [mAU*s]	Height [mAU]	Area %
1	6.720	MM	0.1825	1541.86816	140.83981	6.1453
2	7.792	BB	0.2001	2.35485e4	1830.42786	93.8547

Totals : 2.50904e4 1971.26767

Supplementary Figure 197. HPLC spectrum for 5j

Data File C:\CHEM32\1\DATA\DEF_LC 2016-01-22 21-44-58\QDY-20028-1-AD-05.D
Sample Name:

===

Acq. Operator : Seq. Line : 7
Acq. Instrument : Instrument 1 Location : Vial 31
Injection Date : 1/22/2016 11:18:59 PM Inj : 1
Inj Volume : 5.000 µl
Acq. Method : C:\CHEM32\1\DATA\DEF_LC 2016-01-22 21-44-58\AD-05-20.M
Last changed : 10/14/2015 3:51:32 PM
Analysis Method : C:\CHEM32\1\METHODS\AD-01-10-2.0.M
Last changed : 6/26/2016 2:05:13 PM
(modified after loading)
Additional Info : Peak(s) manually integrated

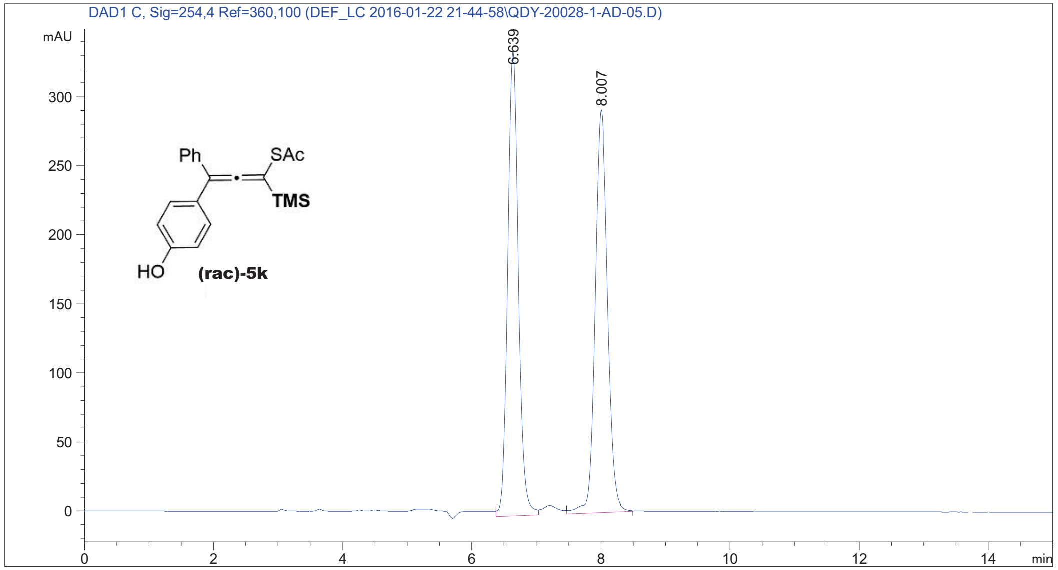

===

Area Percent Report

===

Sorted By : Signal
Multiplier : 1.0000
Dilution : 1.0000
Use Multiplier & Dilution Factor with ISTDs

Signal 1: DAD1 C, Sig=254,4 Ref=360,100

Peak #	RetTime [min]	Type	Width [min]	Area [mAU*s]	Height [mAU]	Area %
1	6.639	BV	0.1785	3878.38232	336.35043	50.0397
2	8.007	VB	0.2049	3872.23242	291.63898	49.9603

Totals : 7750.61475 627.98941

===

Data File C:\CHEM32\1\DATA\DEF_LC 2016-02-19 19-21-26\QDY-20046-2-AD-05.D
Sample Name:

===

Acq. Operator : Seq. Line : 10
Acq. Instrument : Instrument 1 Location : Vial 31
Injection Date : 2/19/2016 9:04:18 PM Inj : 1
Inj Volume : 5.000 µl
Acq. Method : C:\CHEM32\1\DATA\DEF_LC 2016-02-19 19-21-26\AD-05-15.M
Last changed : 7/8/2015 4:31:16 PM
Analysis Method : C:\CHEM32\1\METHODS\AD-01-10-2.0.M
Last changed : 6/26/2016 2:05:13 PM
(modified after loading)

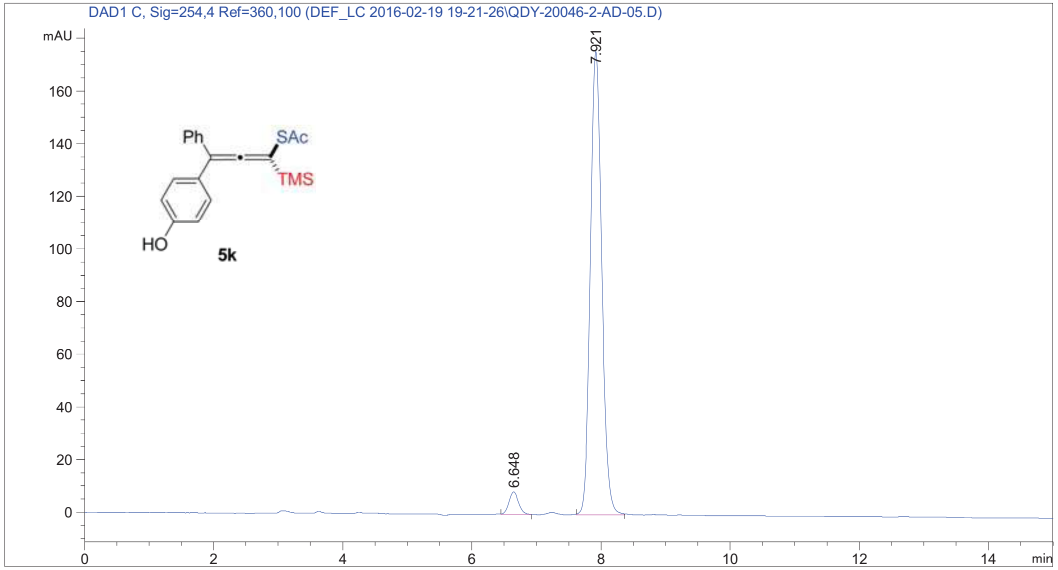

===

Area Percent Report

===

Sorted By : Signal
Multiplier : 1.0000
Dilution : 1.0000
Use Multiplier & Dilution Factor with ISTDs

Signal 1: DAD1 C, Sig=254,4 Ref=360,100

Peak #	RetTime [min]	Type	Width [min]	Area [mAU*s]	Height [mAU]	Area %
1	6.648	BB	0.1604	88.50684	8.57913	3.9644
2	7.921	BB	0.1882	2144.02930	175.93285	96.0356

Totals : 2232.53613 184.51197

Supplementary Figure 198. HPLC spectrum for 5k

===

*** End of Report ***

Data File C:\CHEM32\1\DATA\DEF_LC 2016-03-31 22-59-06\QDY-20102-2-AD-03.D
Sample Name:

===

Acq. Operator : Seq. Line : 7
Acq. Instrument : Instrument 1 Location : Vial 32
Injection Date : 4/1/2016 1:05:56 AM Inj : 1
Inj Volume : 5.000 µl
Acq. Method : C:\CHEM32\1\DATA\DEF_LC 2016-03-31 22-59-06\AD-03-30.M
Last changed : 1/30/2015 10:17:06 AM
Analysis Method : C:\CHEM32\1\METHODS\AD-01-10-2.0.M
Last changed : 6/26/2016 3:55:25 PM
(modified after loading)
Additional Info : Peak(s) manually integrated

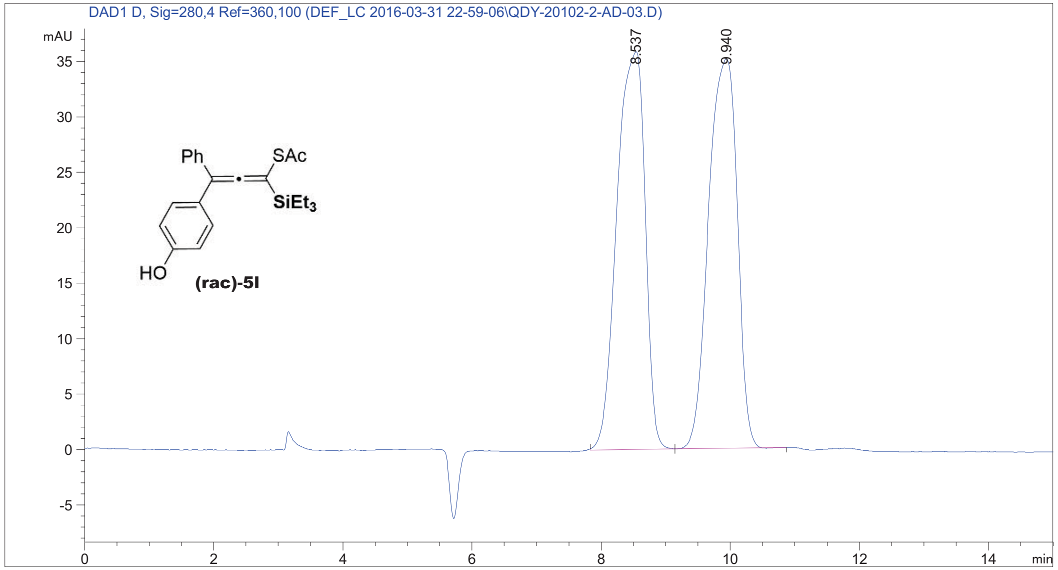

===

Area Percent Report

===

Sorted By : Signal
Multiplier : 1.0000
Dilution : 1.0000
Use Multiplier & Dilution Factor with ISTDs

Signal 1: DAD1 D, Sig=280,4 Ref=360,100

Peak #	RetTime [min]	Type	Width [min]	Area [mAU*s]	Height [mAU]	Area %
1	8.537	BV	0.4946	1090.32153	35.86992	50.1032
2	9.940	VB	0.5005	1085.82886	35.13862	49.8968

Totals : 2176.15039 71.00854

===

Data File C:\CHEM32\1\DATA\DEF_LC 2016-04-16 15-13-11\QDY-20117-2-AD-03.D
Sample Name:

===

Acq. Operator : Seq. Line : 7
Acq. Instrument : Instrument 1 Location : Vial 32
Injection Date : 4/16/2016 5:09:50 PM Inj : 1
Inj Volume : 5.000 µl
Acq. Method : C:\CHEM32\1\DATA\DEF_LC 2016-04-16 15-13-11\AD-03-20.M
Last changed : 4/16/2016 5:22:49 PM
(modified after loading)
Analysis Method : C:\CHEM32\1\METHODS\AD-01-10-2.0.M
Last changed : 6/26/2016 3:55:25 PM
(modified after loading)
Additional Info : Peak(s) manually integrated

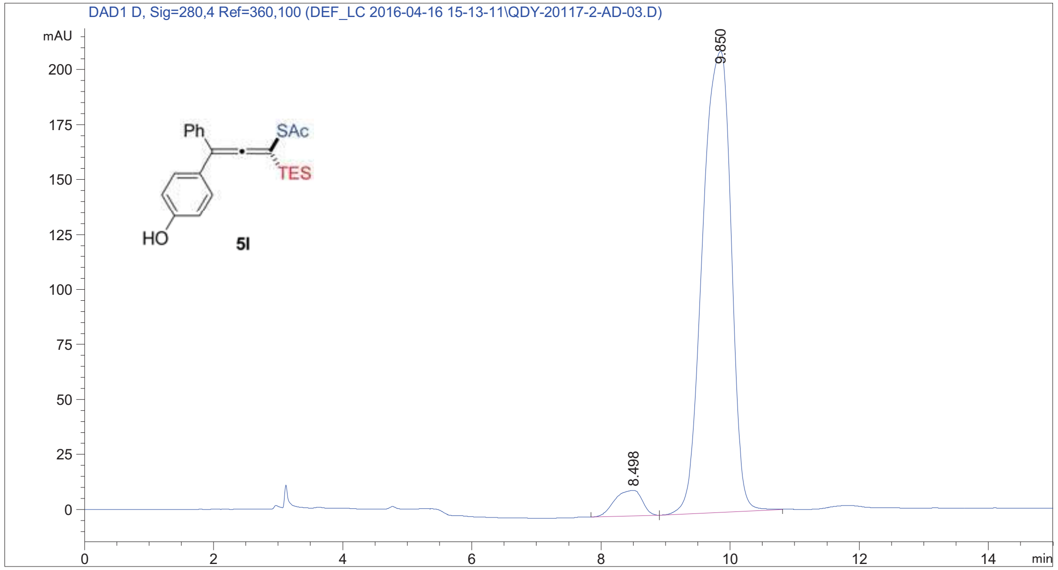

===

Area Percent Report

===

Sorted By : Signal
Multiplier : 1.0000
Dilution : 1.0000
Use Multiplier & Dilution Factor with ISTDs

Signal 1: DAD1 D, Sig=280,4 Ref=360,100

Peak #	RetTime [min]	Type	Width [min]	Area [mAU*s]	Height [mAU]	Area %
1	8.498	BV	0.4241	341.95050	11.59381	5.0289
2	9.850	VB	0.5034	6457.77686	209.60730	94.9711

Totals : 6799.72736 221.20111

Supplementary Figure 199. HPLC spectrum for 5l

Data File C:\CHEM32\1\DATA\DEF_LC 2016-03-12 11-07-21\QDY-20078-2-AD-05.D
Sample Name:

===

Acq. Operator : Seq. Line : 4
Acq. Instrument : Instrument 1 Location : Vial 31
Injection Date : 3/12/2016 12:03:15 PM Inj : 1
Inj Volume : 5.000 µl
Acq. Method : C:\CHEM32\1\DATA\DEF_LC 2016-03-12 11-07-21\AD-05-30.M
Last changed : 3/12/2016 12:20:26 PM
(modified after loading)
Analysis Method : C:\CHEM32\1\METHODS\AD-01-10-2.0.M
Last changed : 6/26/2016 3:10:06 PM
(modified after loading)
Additional Info : Peak(s) manually integrated

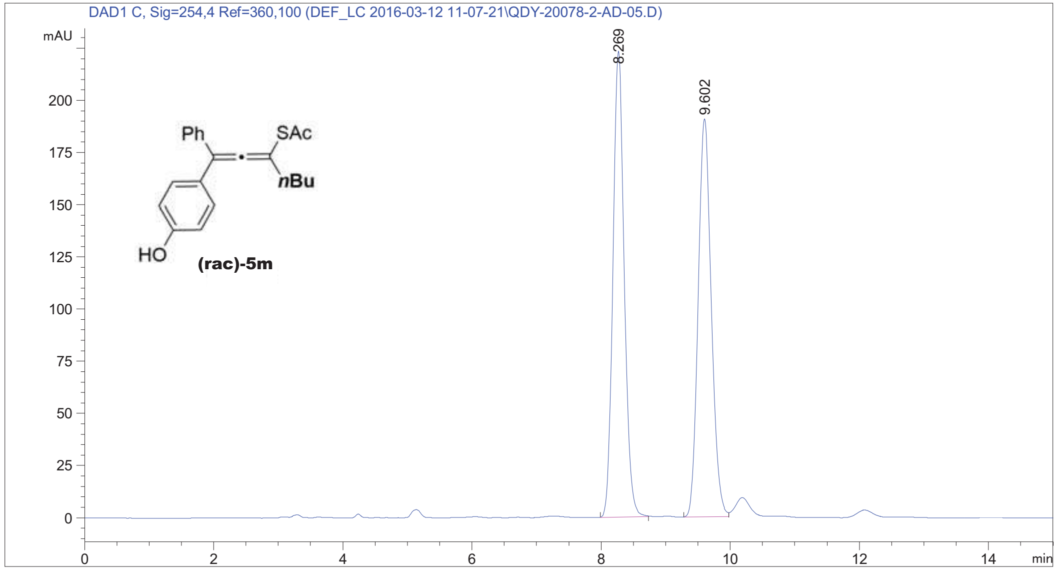

===

Area Percent Report

===

Sorted By : Signal
Multiplier : 1.0000
Dilution : 1.0000
Use Multiplier & Dilution Factor with ISTDs

Signal 1: DAD1 C, Sig=254,4 Ref=360,100

Peak #	RetTime [min]	Type	Width [min]	Area [mAU*s]	Height [mAU]	Area %
1	8.269	BB	0.1876	2708.51978	223.12842	50.1121
2	9.602	BV	0.2190	2696.39819	190.68352	49.8879

Totals : 5404.91797 413.81194

Data File C:\CHEM32\1\DATA\DEF_LC 2016-03-30 13-35-29\QDY-20099-3-AD-05.D
Sample Name:

===

Acq. Operator : Seq. Line : 13
Acq. Instrument : Instrument 1 Location : Vial 33
Injection Date : 3/30/2016 6:42:27 PM Inj : 1
Inj Volume : 5.000 µl
Acq. Method : C:\CHEM32\1\DATA\DEF_LC 2016-03-30 13-35-29\AD-05-15.M
Last changed : 7/8/2015 4:31:16 PM
Analysis Method : C:\CHEM32\1\METHODS\AD-01-10-2.0.M
Last changed : 6/26/2016 2:50:44 PM
(modified after loading)
Additional Info : Peak(s) manually integrated

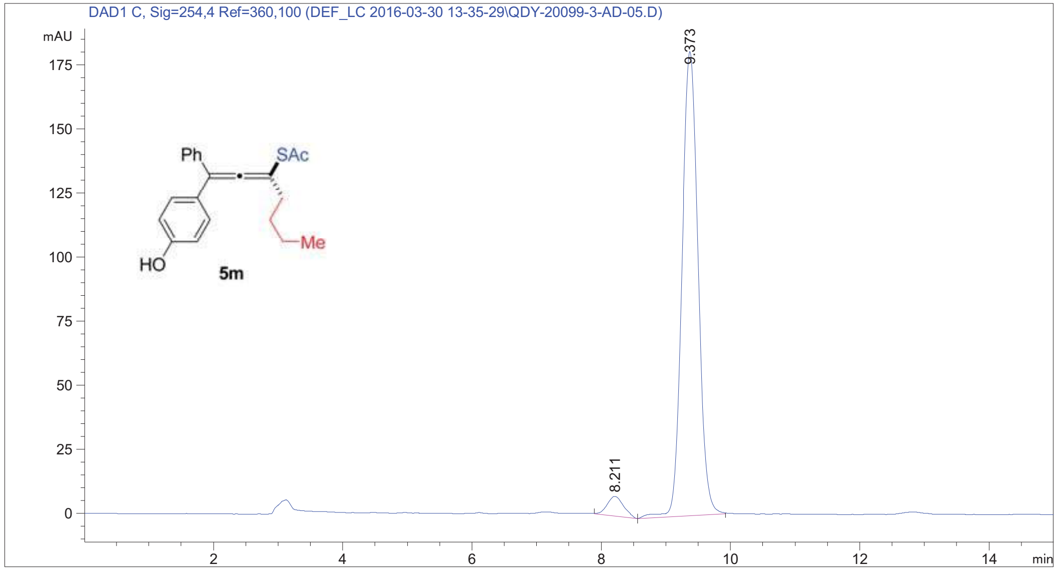

===

Area Percent Report

===

Sorted By : Signal
Multiplier : 1.0000
Dilution : 1.0000
Use Multiplier & Dilution Factor with ISTDs

Signal 1: DAD1 C, Sig=254,4 Ref=360,100

Peak #	RetTime [min]	Type	Width [min]	Area [mAU*s]	Height [mAU]	Area %
1	8.211	BV	0.2769	136.74849	7.71228	4.1759
2	9.373	VB	0.2720	3137.98779	181.20929	95.8241

Totals : 3274.73628 188.92157

===

Supplementary Figure 200. HPLC spectrum for 5m

Data File C:\CHEM32\1\DATA\DEF_LC 2016-04-18 19-47-40\QDY-20118-2-AD-05.D
Sample Name:

===

Acq. Operator : Seq. Line : 24
Acq. Instrument : Instrument 1 Location : Vial 35
Injection Date : 4/19/2016 2:32:40 AM Inj : 1
Inj Volume : 5.000 µl
Acq. Method : C:\CHEM32\1\DATA\DEF_LC 2016-04-18 19-47-40\AD-05-30.M
Last changed : 4/19/2016 2:31:49 AM
(modified after loading)
Analysis Method : C:\CHEM32\1\METHODS\AD-01-10-2.0.M
Last changed : 6/26/2016 4:03:06 PM
(modified after loading)
Additional Info : Peak(s) manually integrated

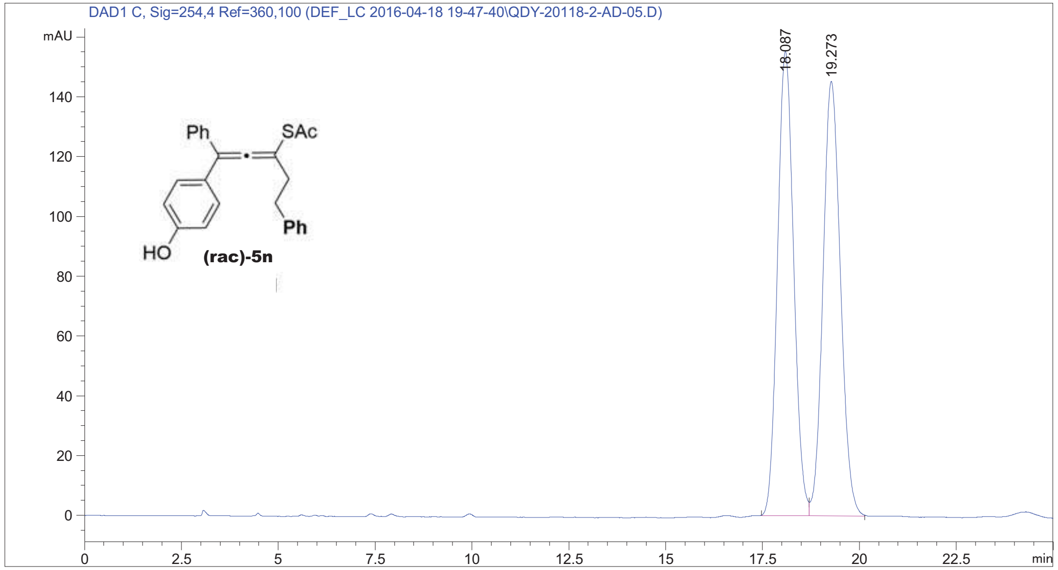

===

Area Percent Report

===

Sorted By : Signal
Multiplier : 1.0000
Dilution : 1.0000
Use Multiplier & Dilution Factor with ISTDs

Signal 1: DAD1 C, Sig=254,4 Ref=360,100

Peak #	RetTime [min]	Type	Width [min]	Area [mAU*s]	Height [mAU]	Area %
1	18.087	BV	0.4539	4498.68213	155.34293	49.6694
2	19.273	VB	0.4899	4558.57422	145.34494	50.3306

Totals : 9057.25635 300.68787

Data File C:\CHEM32\1\DATA\DEF_LC 2016-04-21 13-13-00\QDY-20122-1-ADH-05.D
Sample Name:

===

Acq. Operator : Seq. Line : 35
Acq. Instrument : Instrument 1 Location : Vial 32
Injection Date : 4/22/2016 12:24:04 AM Inj : 1
Inj Volume : 5.000 µl
Acq. Method : C:\CHEM32\1\DATA\DEF_LC 2016-04-21 13-13-00\AD-05-30.M
Last changed : 8/31/2012 5:22:01 PM
Analysis Method : C:\CHEM32\1\METHODS\AD-01-10-2.0.M
Last changed : 6/26/2016 4:03:06 PM
(modified after loading)
Additional Info : Peak(s) manually integrated

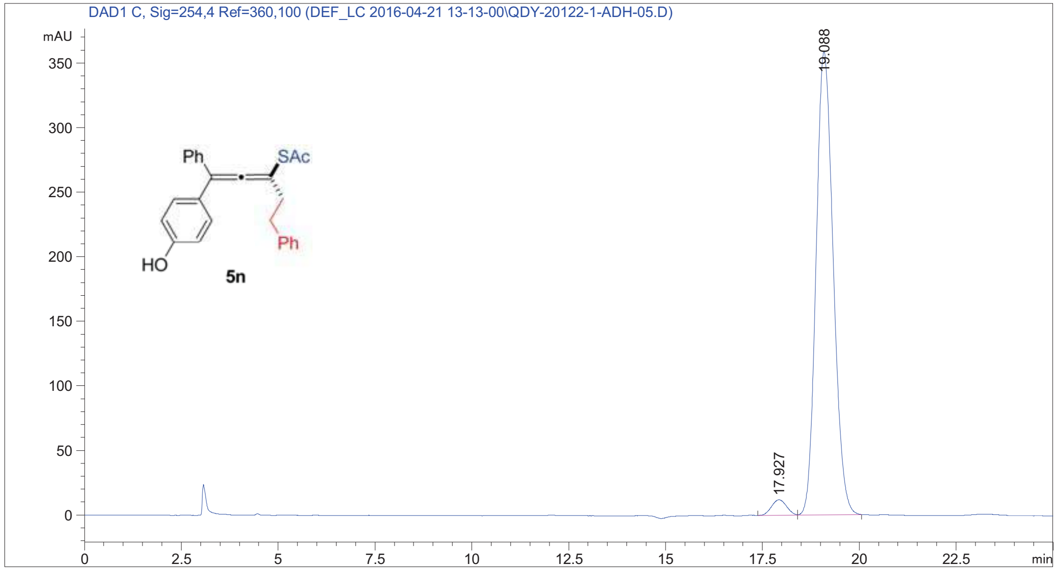

===

Area Percent Report

===

Sorted By : Signal
Multiplier : 1.0000
Dilution : 1.0000
Use Multiplier & Dilution Factor with ISTDs

Signal 1: DAD1 C, Sig=254,4 Ref=360,100

Peak #	RetTime [min]	Type	Width [min]	Area [mAU*s]	Height [mAU]	Area %
1	17.927	BV	0.4259	323.51538	12.02499	2.8466
2	19.088	VB	0.4830	1.10415e4	358.84393	97.1534

Totals : 1.13650e4 370.86892

Supplementary Figure 201. HPLC spectrum for 5n

===

Data File C:\CHEM32\1\DATA\DEF_LC 2016-04-16 15-13-11\QDY-20118-1--AD-05.D
Sample Name:

===

Acq. Operator : Seq. Line : 21
Acq. Instrument : Instrument 1 Location : Vial 34
Injection Date : 4/16/2016 9:20:06 PM Inj : 1
Inj Volume : 5.000 µl
Acq. Method : C:\CHEM32\1\DATA\DEF_LC 2016-04-16 15-13-11\AD-05-20.M
Last changed : 10/14/2015 3:51:32 PM
Analysis Method : C:\CHEM32\1\METHODS\AD-01-10-2.0.M
Last changed : 6/26/2016 1:41:03 PM
(modified after loading)
Additional Info : Peak(s) manually integrated

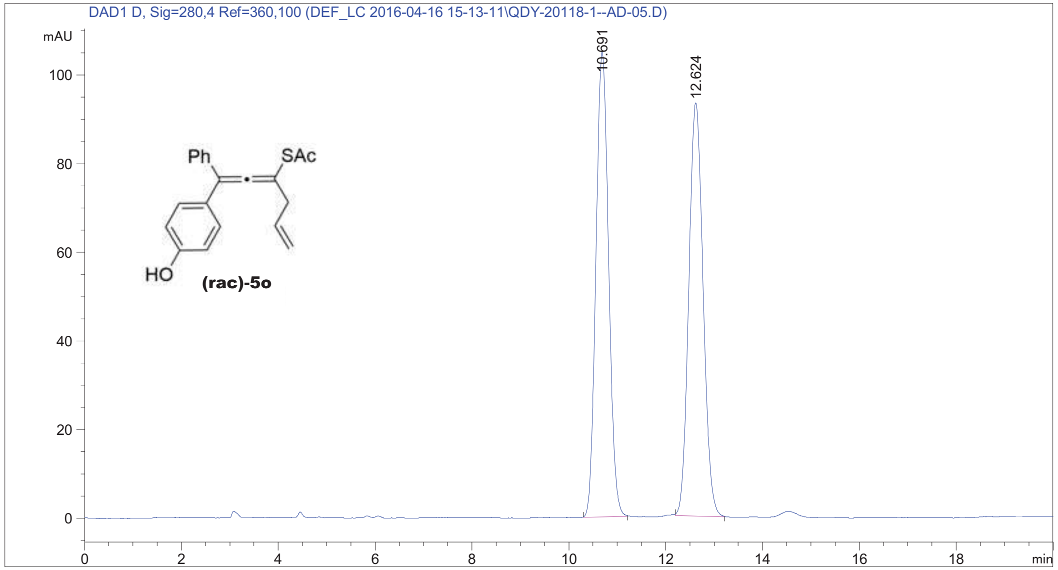

===

Area Percent Report

===

Sorted By : Signal
Multiplier : 1.0000
Dilution : 1.0000
Use Multiplier & Dilution Factor with ISTDs

Signal 1: DAD1 D, Sig=280,4 Ref=360,100

Peak #	RetTime [min]	Type	Width [min]	Area [mAU*s]	Height [mAU]	Area %
1	10.691	BB	0.2858	1906.98474	105.06792	49.9276
2	12.624	BB	0.3212	1912.51782	93.29216	50.0724

Totals : 3819.50256 198.36008

===

Data File C:\CHEM32\1\DATA\QDY 2016-06-16 16-15-19\QDY-20122-3-ADH-05.D
Sample Name:

===

Acq. Operator : Seq. Line : 20
Acq. Instrument : Instrument 1 Location : Vial 32
Injection Date : 6/16/2016 10:44:01 PM Inj : 1
Inj Volume : 5.000 µl
Acq. Method : C:\CHEM32\1\DATA\QDY 2016-06-16 16-15-19\AD-05-20.M
Last changed : 6/16/2016 10:43:09 PM
(modified after loading)
Analysis Method : C:\CHEM32\1\METHODS\AD-01-10-2.0.M
Last changed : 6/26/2016 1:41:03 PM
(modified after loading)
Additional Info : Peak(s) manually integrated

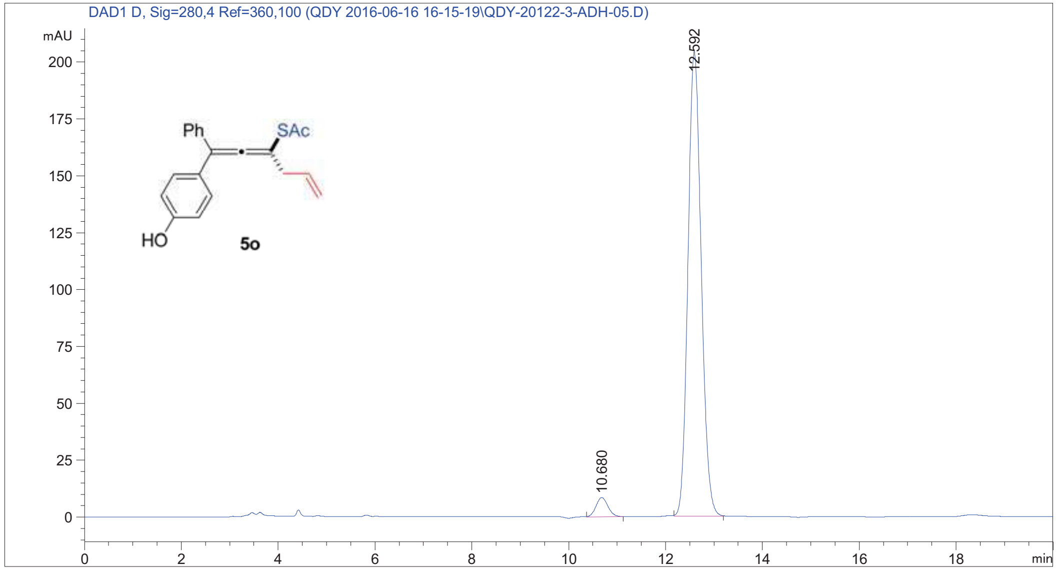

===

Area Percent Report

===

Sorted By : Signal
Multiplier : 1.0000
Dilution : 1.0000
Use Multiplier & Dilution Factor with ISTDs

Signal 1: DAD1 D, Sig=280,4 Ref=360,100

Peak #	RetTime [min]	Type	Width [min]	Area [mAU*s]	Height [mAU]	Area %
1	10.680	BB	0.2657	145.52939	8.50029	3.6314
2	12.592	BB	0.2946	3862.04248	204.24312	96.3686

Totals : 4007.57187 212.74341

Supplementary Figure 202. HPLC spectrum for 5o

Data File C:\CHEM32\1\DATA\DEF_LC 2016-03-19 10-48-51\QDY-20091-2-AD--05.D
Sample Name:

===

Acq. Operator : Seq. Line : 32
Acq. Instrument : Instrument 1 Location : Vial 32
Injection Date : 3/19/2016 10:32:01 PM Inj : 1
Inj Volume : 5.000 µl
Acq. Method : C:\CHEM32\1\DATA\DEF_LC 2016-03-19 10-48-51\AD-05-30.M
Last changed : 3/19/2016 10:31:10 PM
(modified after loading)
Analysis Method : C:\CHEM32\1\METHODS\AD-01-10-2.0.M
Last changed : 6/26/2016 8:35:21 PM
(modified after loading)
Additional Info : Peak(s) manually integrated

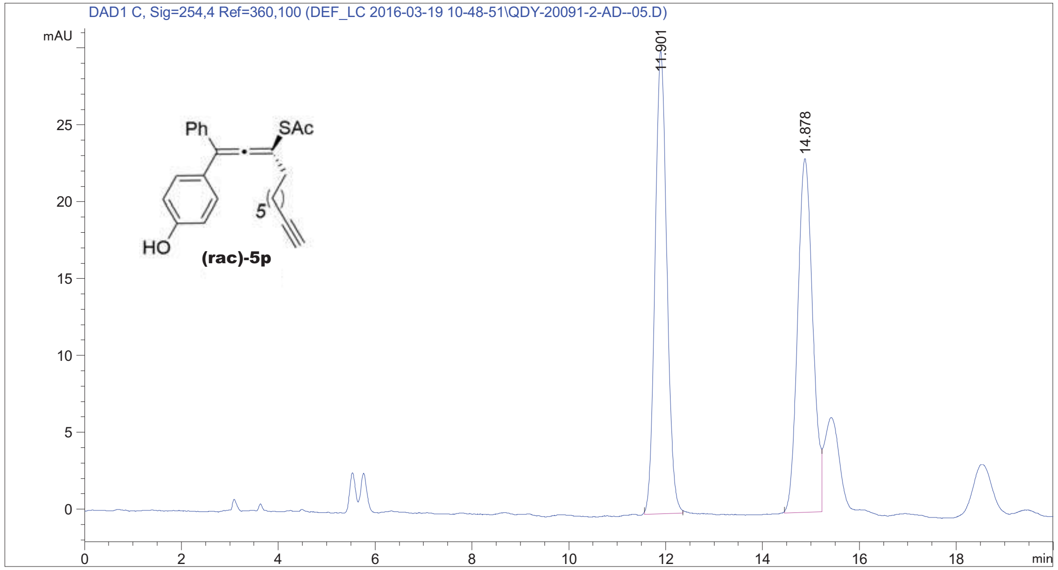

===

Area Percent Report

===

Sorted By : Signal
Multiplier : 1.0000
Dilution : 1.0000
Use Multiplier & Dilution Factor with ISTDs

Signal 1: DAD1 C, Sig=254,4 Ref=360,100

Peak #	RetTime [min]	Type	Width [min]	Area [mAU*s]	Height [mAU]	Area %
1	11.901	BB	0.2634	508.70099	30.07041	50.4435
2	14.878	BV	0.3393	499.75665	23.01589	49.5565

Totals : 1008.45764 53.08629

Data File C:\CHEM32\1\DATA\DEF_LC 2016-03-31 10-15-06\QDY-20099-4-ADH-05.D
Sample Name:

===

Acq. Operator : Seq. Line : 5
Acq. Instrument : Instrument 1 Location : Vial 34
Injection Date : 3/31/2016 11:24:51 AM Inj : 1
Inj Volume : 5.000 µl
Acq. Method : C:\CHEM32\1\DATA\DEF_LC 2016-03-31 10-15-06\AD-05-20.M
Last changed : 3/31/2016 11:27:42 AM
(modified after loading)
Analysis Method : C:\CHEM32\1\METHODS\AD-01-10-2.0.M
Last changed : 6/26/2016 8:32:29 PM
(modified after loading)
Additional Info : Peak(s) manually integrated

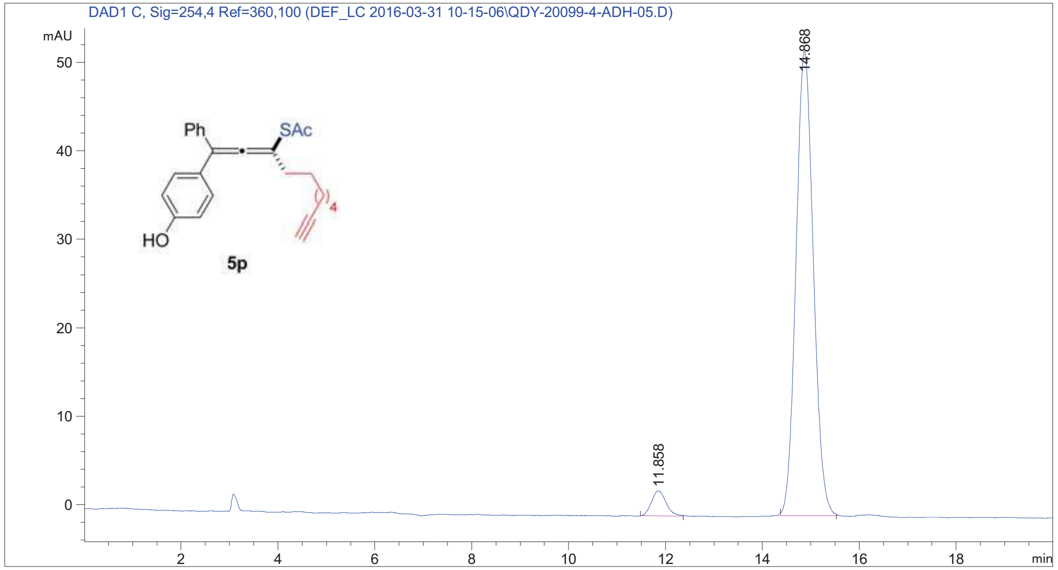

===

Area Percent Report

===

Sorted By : Signal
Multiplier : 1.0000
Dilution : 1.0000
Use Multiplier & Dilution Factor with ISTDs

Signal 1: DAD1 C, Sig=254,4 Ref=360,100

Peak #	RetTime [min]	Type	Width [min]	Area [mAU*s]	Height [mAU]	Area %
1	11.858	BB	0.2872	58.77607	2.81997	4.3129
2	14.868	BB	0.3859	1304.01526	52.48608	95.6871

Totals : 1362.79133 55.30606

Supplementary Figure 203. HPLC spectrum for 5p

Data File C:\CHEM32\1\DATA\DEF_LC 2016-04-01 15-35-39\QDY-20103-2-RAC-OD-05-05.D
Sample Name:

===

Acq. Operator : Seq. Line : 85
Acq. Instrument : Instrument 1 Location : Vial 33
Injection Date : 4/2/2016 10:04:16 PM Inj : 1
Inj Volume : 5.000 µl
Acq. Method : C:\CHEM32\1\DATA\DEF_LC 2016-04-01 15-35-39\OD-05-40-0.5.M
Last changed : 12/11/2014 6:35:45 PM
Analysis Method : C:\CHEM32\1\METHODS\AD-01-10-2.0.M
Last changed : 6/26/2016 4:11:48 PM
(modified after loading)
Additional Info : Peak(s) manually integrated

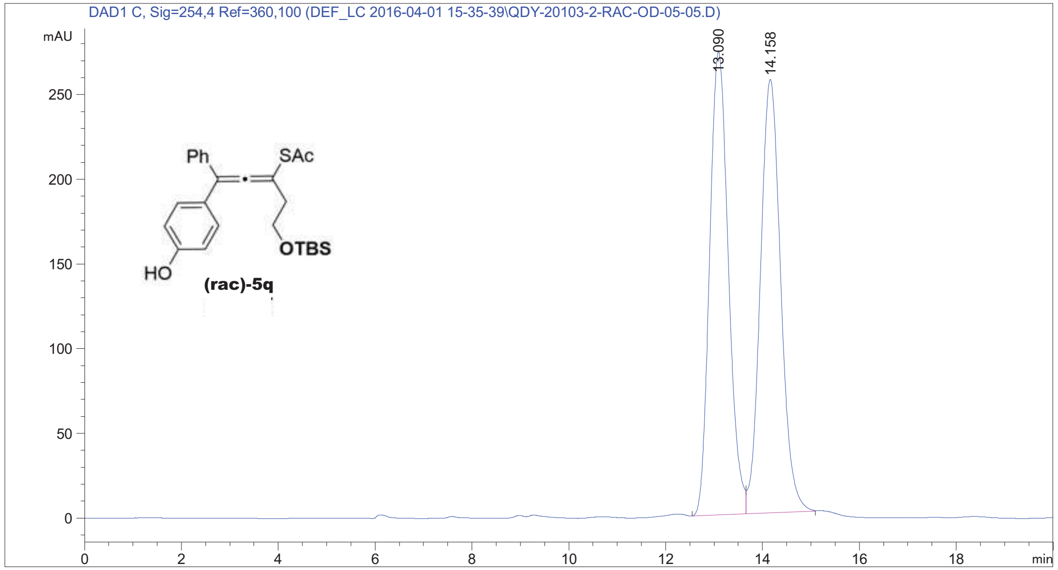

===

Area Percent Report

===

Sorted By : Signal
Multiplier : 1.0000
Dilution : 1.0000
Use Multiplier & Dilution Factor with ISTDs

Signal 1: DAD1 C, Sig=254,4 Ref=360,100

Peak #	RetTime [min]	Type	Width [min]	Area [mAU*s]	Height [mAU]	Area %
1	13.090	BV	0.4200	7359.00879	273.43948	49.6634
2	14.158	VB	0.4540	7458.77197	255.94975	50.3366

Totals : 1.48178e4 529.38924

===

Data File C:\CHEM32\1\DATA\DEF_LC 2016-04-07 15-16-52\QDY-20103-2-OD-05-05.D
Sample Name:

===

Acq. Operator : Seq. Line : 2
Acq. Instrument : Instrument 1 Location : Vial 32
Injection Date : 4/7/2016 3:30:20 PM Inj : 1
Inj Volume : 5.000 µl
Acq. Method : C:\CHEM32\1\DATA\DEF_LC 2016-04-07 15-16-52\OD-05-20-0.5.M
Last changed : 4/7/2016 10:27:36 AM
Analysis Method : C:\CHEM32\1\METHODS\AD-01-10-2.0.M
Last changed : 6/26/2016 4:09:19 PM
(modified after loading)
Additional Info : Peak(s) manually integrated

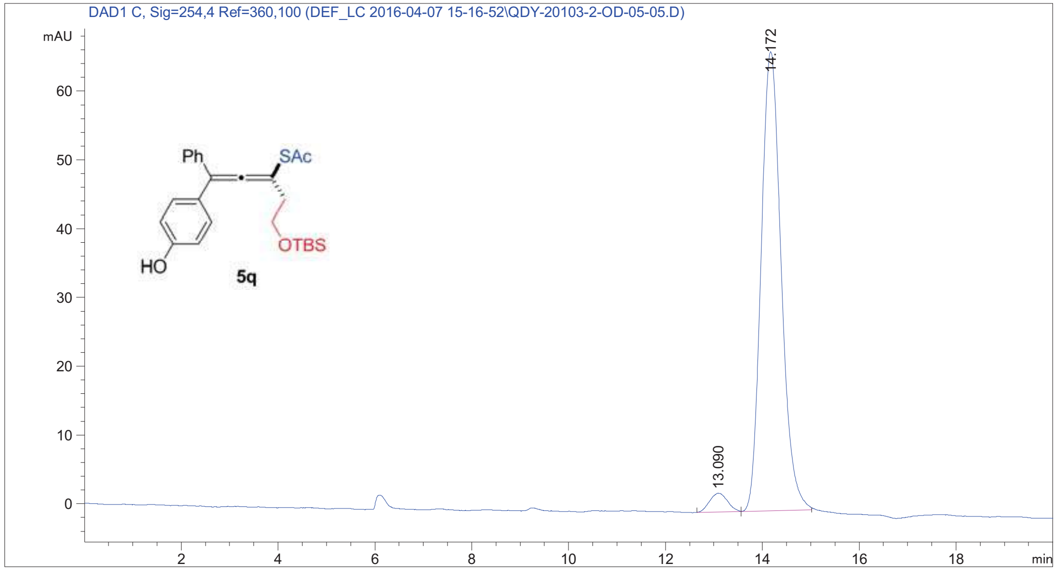

===

Area Percent Report

===

Sorted By : Signal
Multiplier : 1.0000
Dilution : 1.0000
Use Multiplier & Dilution Factor with ISTDs

Signal 1: DAD1 C, Sig=254,4 Ref=360,100

Peak #	RetTime [min]	Type	Width [min]	Area [mAU*s]	Height [mAU]	Area %
1	13.090	BV	0.3273	69.24229	2.71682	3.4859
2	14.172	VB	0.4450	1917.12439	66.75624	96.5141

Totals : 1986.36668 69.47306

===

Supplementary Figure 204. HPLC spectrum for 5q

Data File C:\CHEM32\1\DATA\DEF_LC 2016-04-12 14-34-50\QDY-20111-2-RAC-AD-10.D
Sample Name:

===

Acq. Operator : Seq. Line : 9
Acq. Instrument : Instrument 1 Location : Vial 31
Injection Date : 4/12/2016 5:37:31 PM Inj : 1
Inj Volume : 5.000 µl
Acq. Method : C:\CHEM32\1\DATA\DEF_LC 2016-04-12 14-34-50\AD-10-20.M
Last changed : 11/28/2014 10:15:17 AM
Analysis Method : C:\CHEM32\1\METHODS\AD-01-10-2.0.M
Last changed : 6/26/2016 4:17:43 PM
(modified after loading)
Additional Info : Peak(s) manually integrated

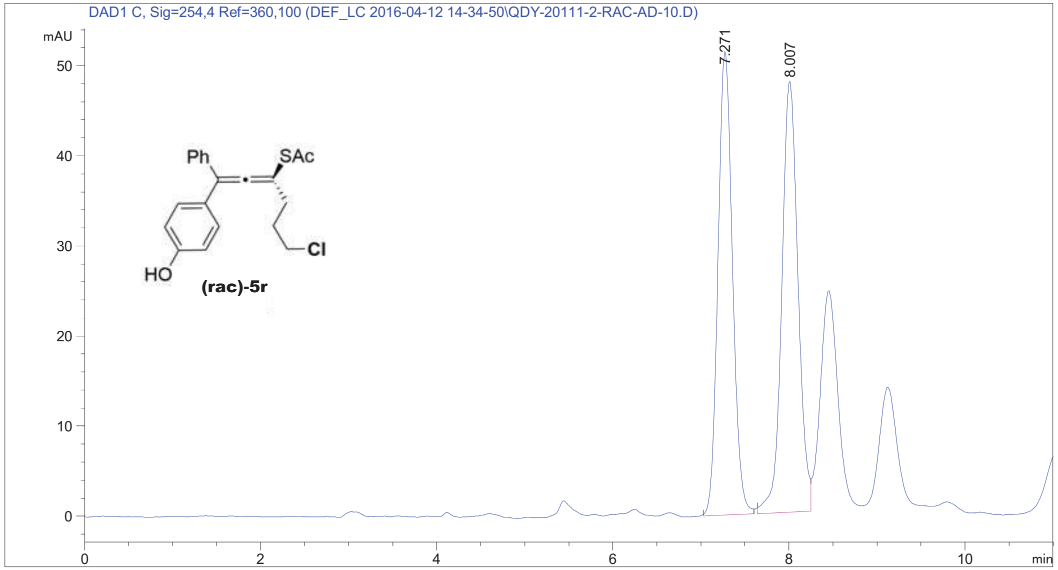

===

Area Percent Report

===

Sorted By : Signal
Multiplier : 1.0000
Dilution : 1.0000
Use Multiplier & Dilution Factor with ISTDs

Signal 1: DAD1 C, Sig=254,4 Ref=360,100

Peak #	RetTime [min]	Type	Width [min]	Area [mAU*s]	Height [mAU]	Area %
1	7.271	BB	0.1783	592.31207	51.44891	49.0472
2	8.007	BV	0.1981	615.32520	47.82840	50.9528

Totals : 1207.63727 99.27731

===

Instrument 1 6/26/2016 4:18:33 PM Page 1 of 1

Data File C:\CHEM32\1\DATA\DEF_LC 2016-04-12 14-34-50\QDY-20111-2-AD-10.D
Sample Name:

===

Acq. Operator : Seq. Line : 8
Acq. Instrument : Instrument 1 Location : Vial 32
Injection Date : 4/12/2016 5:16:25 PM Inj : 1
Inj Volume : 5.000 µl
Acq. Method : C:\CHEM32\1\DATA\DEF_LC 2016-04-12 14-34-50\AD-10-20.M
Last changed : 11/28/2014 10:15:17 AM
Analysis Method : C:\CHEM32\1\METHODS\AD-01-10-2.0.M
Last changed : 6/26/2016 4:17:43 PM
(modified after loading)
Additional Info : Peak(s) manually integrated

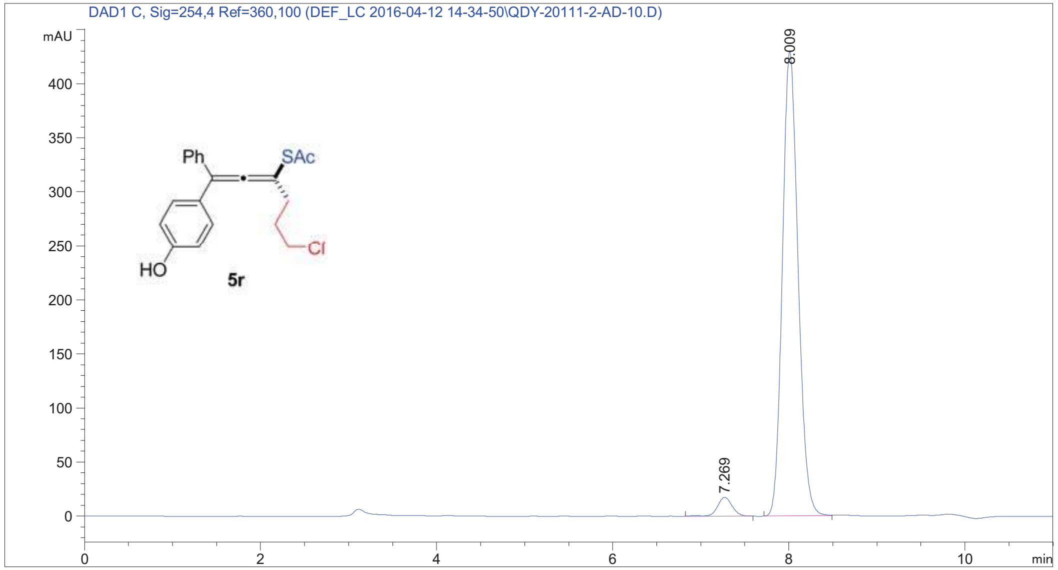

===

Area Percent Report

===

Sorted By : Signal
Multiplier : 1.0000
Dilution : 1.0000
Use Multiplier & Dilution Factor with ISTDs

Signal 1: DAD1 C, Sig=254,4 Ref=360,100

Peak #	RetTime [min]	Type	Width [min]	Area [mAU*s]	Height [mAU]	Area %
1	7.269	BB	0.1810	204.44051	17.41126	3.7023
2	8.009	BB	0.1925	5317.61865	429.46994	96.2977

Totals : 5522.05916 446.88120

===

Supplementary Figure 205. HPLC spectrum for 5r

Instrument 1 6/26/2016 4:20:35 PM Page 1 of 1

Data File C:\CHEM32\1\DATA\DEF_LC 2016-04-23 00-18-02\QDY-20123-2-AD-10.D
Sample Name:

```
=====================================================================
Acq. Operator   :                               Seq. Line :   7
Acq. Instrument : Instrument 1                   Location : Vial 32
Injection Date  : 4/23/2016 2:07:44 AM                Inj :   1
                                               Inj Volume : 5.000 µl
Acq. Method     : C:\CHEM32\1\DATA\DEF_LC 2016-04-23 00-18-02\AD-10-30.M
Last changed    : 11/30/2015 12:57:24 PM
Analysis Method : C:\CHEM32\1\METHODS\AD-01-10-2.0.M
Last changed    : 6/26/2016 1:41:03 PM
                  (modified after loading)
Additional Info : Peak(s) manually integrated
```

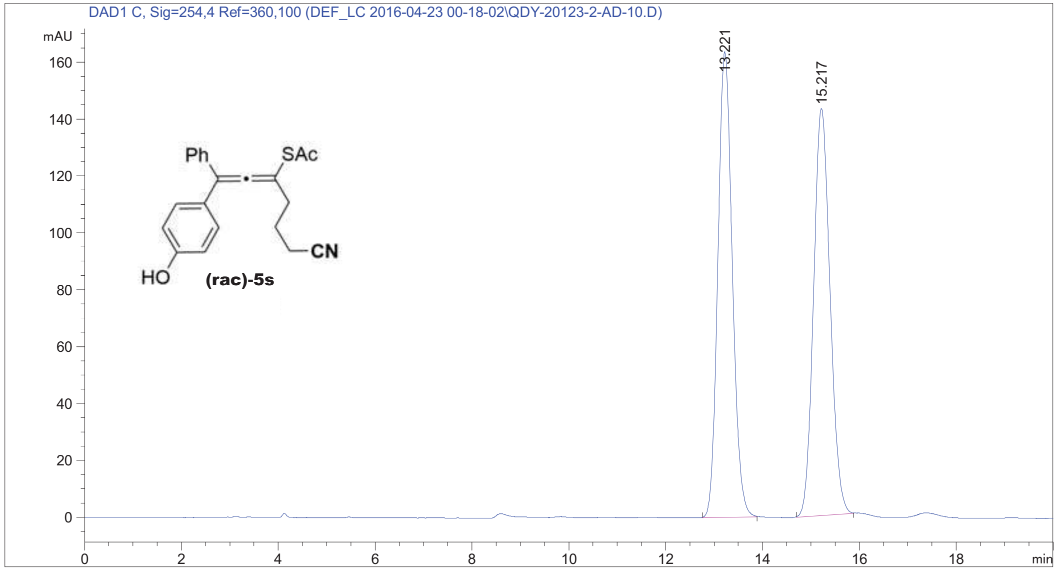


```
=====================================================================
                         Area Percent Report
=====================================================================

Sorted By             :      Signal
Multiplier            :      1.0000
Dilution              :      1.0000
Use Multiplier & Dilution Factor with ISTDs


Signal 1: DAD1 C, Sig=254,4 Ref=360,100

Peak RetTime Type  Width     Area      Height     Area
  #   [min]        [min]   [mAU*s]     [mAU]        %
----|-------|----|-------|----------|----------|--------|
   1  13.221 BB    0.3219 3394.72974  163.70085  50.0480
   2  15.217 BB    0.3682 3388.21924  143.13855  49.9520

Totals :                  6782.94897  306.83940

=====================================================================
```


Data File C:\CHEM32\1\DATA\DEF_LC 2016-04-28 23-38-49\QDY-20124-2-ADH-10.D
Sample Name:

```
=====================================================================
Acq. Operator   :                               Seq. Line :   2
Acq. Instrument : Instrument 1                   Location : Vial 34
Injection Date  : 4/28/2016 11:51:09 PM               Inj :   1
                                               Inj Volume : 5.000 µl
Acq. Method     : C:\CHEM32\1\DATA\DEF_LC 2016-04-28 23-38-49\AD-10-25.M
Last changed    : 4/28/2016 11:50:16 PM
                  (modified after loading)
Analysis Method : C:\CHEM32\1\METHODS\IC-20-70.M
Last changed    : 6/18/2016 10:41:44 AM
                  (modified after loading)
Additional Info : Peak(s) manually integrated
```

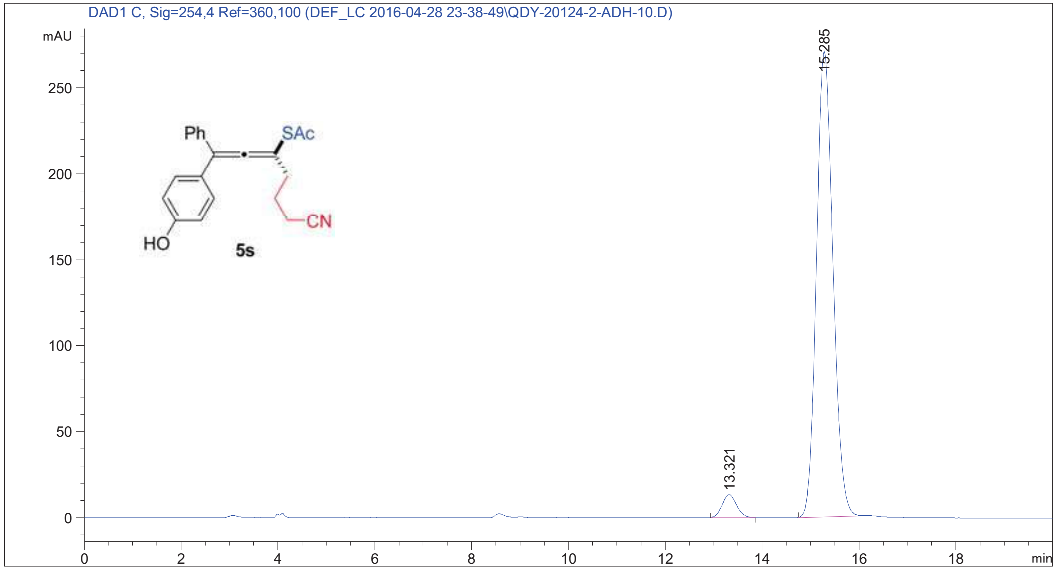


```
=====================================================================
                         Area Percent Report
=====================================================================

Sorted By             :      Signal
Multiplier            :      1.0000
Dilution              :      1.0000
Use Multiplier & Dilution Factor with ISTDs


Signal 1: DAD1 C, Sig=254,4 Ref=360,100

Peak RetTime Type  Width     Area      Height     Area
  #   [min]        [min]   [mAU*s]     [mAU]        %
----|-------|----|-------|----------|----------|--------|
   1  13.321 BB    0.3241  282.51279   13.50160   4.2193
   2  15.285 BB    0.3685 6413.16797  270.58994  95.7807

Totals :                  6695.68076  284.09153
```

Supplementary Figure 206. HPLC spectrum for 5s

Data File C:\CHEM32\1\DATA\DEF_LC 2016-04-06 09-31-16\QDY-20103-1-RAC-AD-03.D
Sample Name:

===

Acq. Operator : Seq. Line : 45
Acq. Instrument : Instrument 1 Location : Vial 32
Injection Date : 4/6/2016 11:40:19 PM Inj : 1
Inj Volume : 5.000 µl
Acq. Method : C:\CHEM32\1\DATA\DEF_LC 2016-04-06 09-31-16\AD-03-30.M
Last changed : 1/30/2015 10:17:06 AM
Analysis Method : C:\CHEM32\1\METHODS\AD-01-10-2.0.M
Last changed : 6/28/2016 11:26:02 AM
(modified after loading)
Additional Info : Peak(s) manually integrated

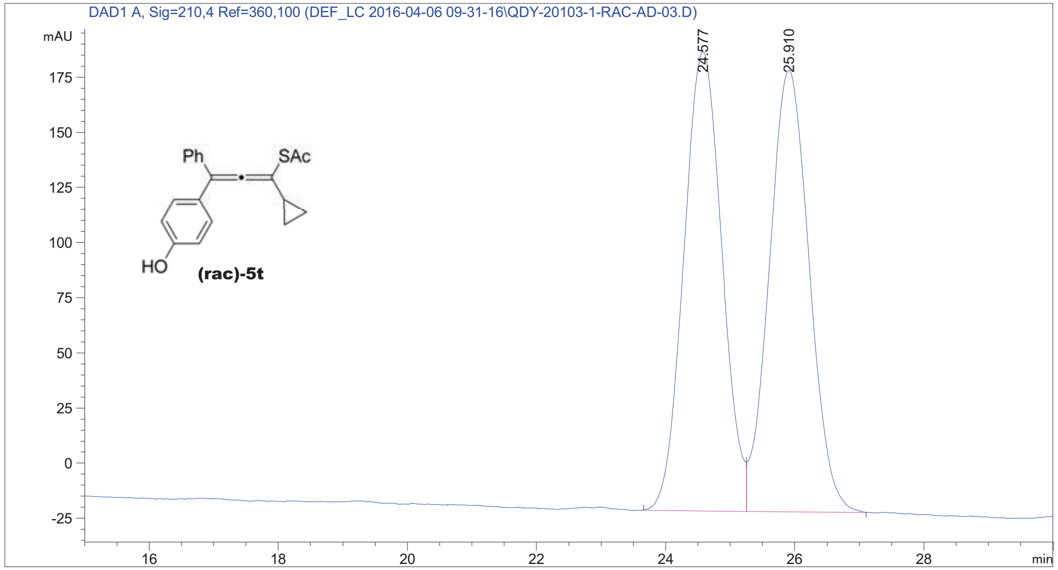

===

Area Percent Report

===

Sorted By : Signal
Multiplier : 1.0000
Dilution : 1.0000
Use Multiplier & Dilution Factor with ISTDs

Signal 1: DAD1 A, Sig=210,4 Ref=360,100

Peak #	RetTime [min]	Type	Width [min]	Area [mAU*s]	Height [mAU]	Area %
1	24.577	BV	0.6475	8555.16602	208.15483	49.7271
2	25.910	VB	0.6621	8649.06543	200.16136	50.2729

Totals : 1.72042e4 408.31619

===

Data File C:\CHEM32\1\DATA\DEF_LC 2016-04-01 15-35-39\QDY-10103-1-ADH-03.D
Sample Name:

===

Acq. Operator : Seq. Line : 6
Acq. Instrument : Instrument 1 Location : Vial 31
Injection Date : 4/1/2016 4:59:28 PM Inj : 1
Inj Volume : 5.000 µl
Different Inj Volume from Sequence ! Actual Inj Volume : 6.000 µl
Acq. Method : C:\CHEM32\1\DATA\DEF_LC 2016-04-01 15-35-39\AD-03-30.M
Last changed : 1/30/2015 10:17:06 AM
Analysis Method : C:\CHEM32\1\METHODS\AD-01-10-2.0.M
Last changed : 6/28/2016 11:20:17 AM
(modified after loading)
Additional Info : Peak(s) manually integrated

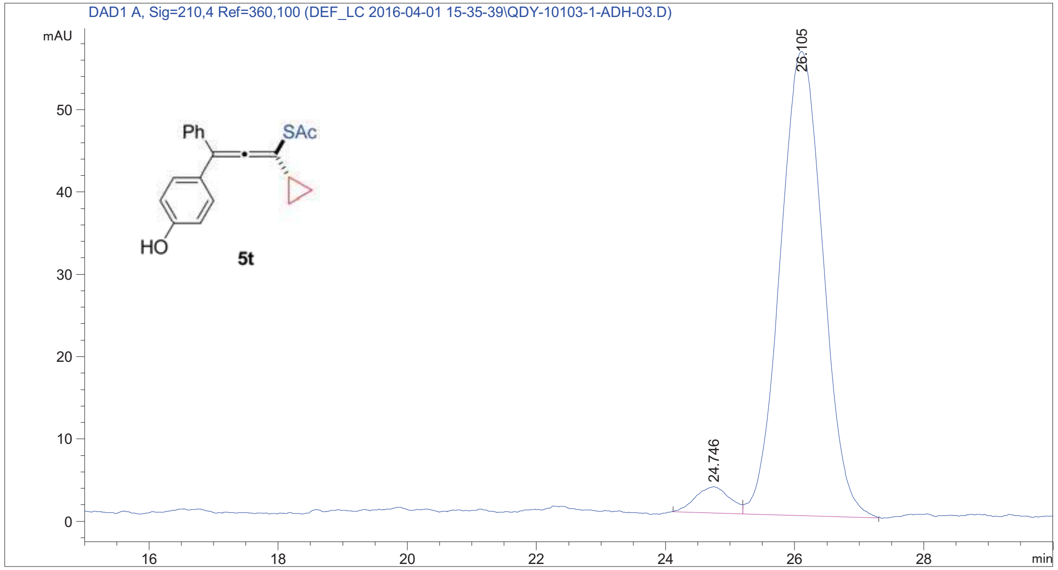

===

Area Percent Report

===

Sorted By : Signal
Multiplier : 1.0000
Dilution : 1.0000
Use Multiplier & Dilution Factor with ISTDs

Signal 1: DAD1 A, Sig=210,4 Ref=360,100

Peak #	RetTime [min]	Type	Width [min]	Area [mAU*s]	Height [mAU]	Area %
1	24.746	BV	0.4412	117.73360	3.20055	4.3359
2	26.105	VB	0.6777	2597.57642	56.36382	95.6641

Totals : 2715.31002 59.56437

Supplementary Figure 207. HPLC spectrum for 5t

Data File C:\CHEM32\1\DATA\DEF_LC 2016-03-31 22-59-06\QDY-20102-1-AD-02.D
Sample Name:

===

Acq. Operator : Seq. Line : 5
Acq. Instrument : Instrument 1 Location : Vial 31
Injection Date : 4/1/2016 12:08:16 AM Inj : 1
Inj Volume : 5.000 µl
Acq. Method : C:\CHEM32\1\DATA\DEF_LC 2016-03-31 22-59-06\AD-02-20.M
Last changed : 4/1/2016 12:19:44 AM
(modified after loading)
Analysis Method : C:\CHEM32\1\METHODS\AD-01-10-2.0.M
Last changed : 6/26/2016 4:00:03 PM
(modified after loading)
Additional Info : Peak(s) manually integrated

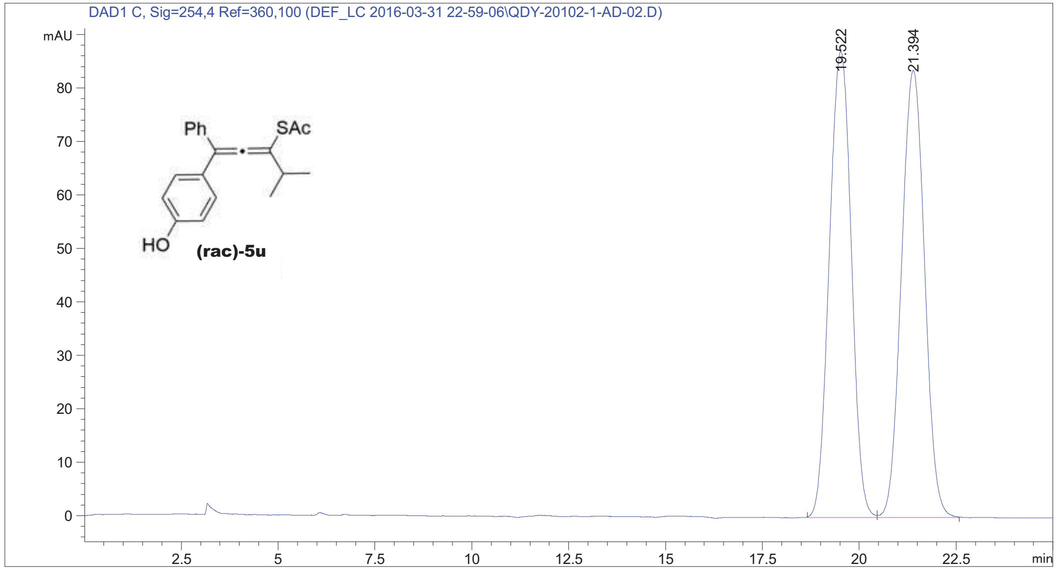

===

Area Percent Report

===

Sorted By : Signal
Multiplier : 1.0000
Dilution : 1.0000
Use Multiplier & Dilution Factor with ISTDs

Signal 1: DAD1 C, Sig=254,4 Ref=360,100

Peak #	RetTime [min]	Type	Width [min]	Area [mAU*s]	Height [mAU]	Area %
1	19.522	BV	0.6306	3440.79224	87.14542	49.7928
2	21.394	VB	0.6621	3469.42822	83.61929	50.2072

Totals : 6910.22046 170.76472

Data File C:\CHEM32\1\DATA\DEF_LC 2016-04-01 15-35-39\QDY-10103-3-AD-02.D
Sample Name:

===

Acq. Operator : Seq. Line : 55
Acq. Instrument : Instrument 1 Location : Vial 35
Injection Date : 4/2/2016 12:03:22 PM Inj : 1
Inj Volume : 5.000 µl
Acq. Method : C:\CHEM32\1\DATA\DEF_LC 2016-04-01 15-35-39\AD-02-30.M
Last changed : 2/4/2015 1:17:54 PM
Analysis Method : C:\CHEM32\1\METHODS\AD-01-10-2.0.M
Last changed : 6/26/2016 4:03:06 PM
(modified after loading)
Additional Info : Peak(s) manually integrated

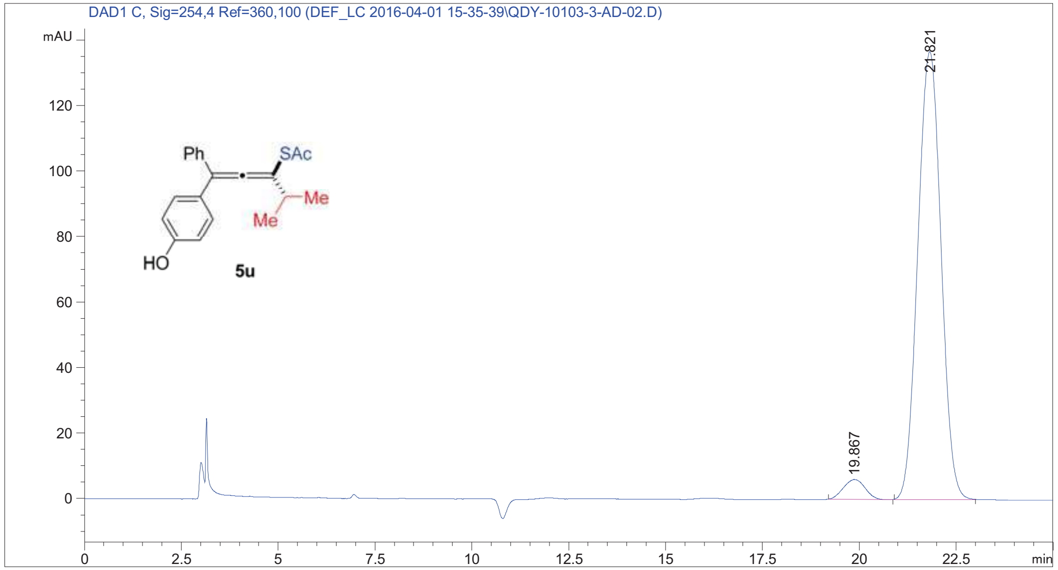

===

Area Percent Report

===

Sorted By : Signal
Multiplier : 1.0000
Dilution : 1.0000
Use Multiplier & Dilution Factor with ISTDs

Signal 1: DAD1 C, Sig=254,4 Ref=360,100

Peak #	RetTime [min]	Type	Width [min]	Area [mAU*s]	Height [mAU]	Area %
1	19.867	BB	0.5267	232.61073	6.04875	3.9310
2	21.821	BB	0.6612	5684.76416	136.70180	96.0690

Totals : 5917.37489 142.75055

===

Supplementary Figure 208. HPLC spectrum for 5u

Data File C:\CHEM32\1\DATA\DEF_LC 2016-01-20 16-36-43\QDY-20024-2-AD-05.D
Sample Name:

===

Acq. Operator : Seq. Line : 6
Acq. Instrument : Instrument 1 Location : Vial 31
Injection Date : 1/20/2016 5:56:11 PM Inj : 1
Inj Volume : 5.000 µl
Acq. Method : C:\CHEM32\1\DATA\DEF_LC 2016-01-20 16-36-43\AD-05-30.M
Last changed : 1/20/2016 5:55:21 PM
(modified after loading)
Analysis Method : C:\CHEM32\1\METHODS\AD-01-10-2.0.M
Last changed : 6/26/2016 2:39:22 PM
(modified after loading)
Additional Info : Peak(s) manually integrated

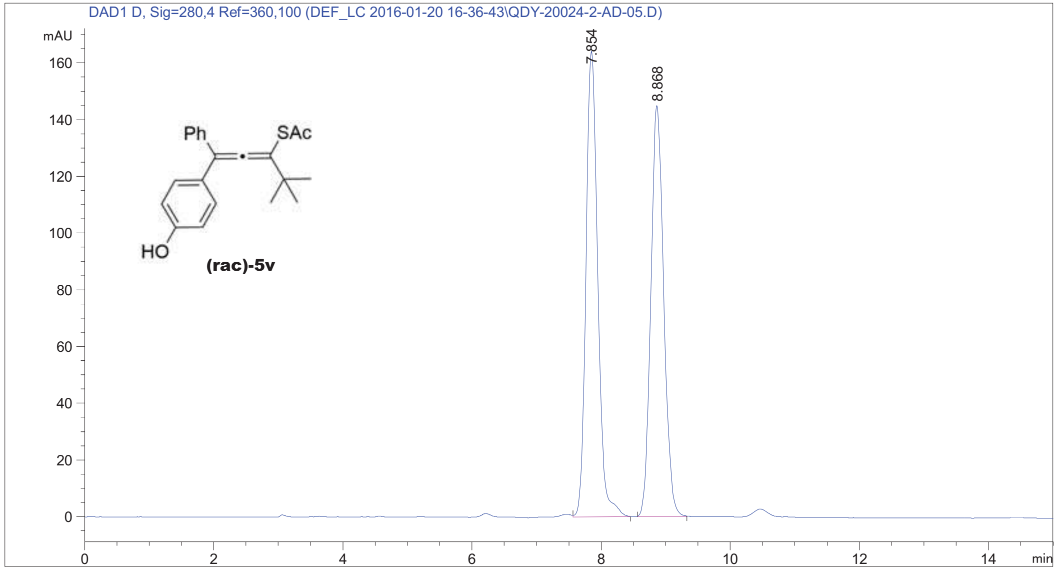

===

Area Percent Report

===

Sorted By : Signal
Multiplier : 1.0000
Dilution : 1.0000
Use Multiplier & Dilution Factor with ISTDs

Signal 1: DAD1 D, Sig=280,4 Ref=360,100

Peak #	RetTime [min]	Type	Width [min]	Area [mAU*s]	Height [mAU]	Area %
1	7.854	VB	0.1955	2075.83936	164.15967	50.7349
2	8.868	BB	0.2163	2015.70105	144.91257	49.2651

Totals : 4091.54041 309.07224

Data File C:\CHEM32\1\DATA\DEF_LC 2016-03-25 18-16-02\QDY-20046-3-AD-05.D
Sample Name:

===

Acq. Operator : Seq. Line : 2
Acq. Instrument : Instrument 1 Location : Vial 31
Injection Date : 3/25/2016 6:29:38 PM Inj : 1
Inj Volume : 5.000 µl
Acq. Method : C:\CHEM32\1\DATA\DEF_LC 2016-03-25 18-16-02\AD-05-20.M
Last changed : 10/14/2015 3:51:32 PM
Analysis Method : C:\CHEM32\1\METHODS\AD-01-10-2.0.M
Last changed : 6/26/2016 2:39:22 PM
(modified after loading)

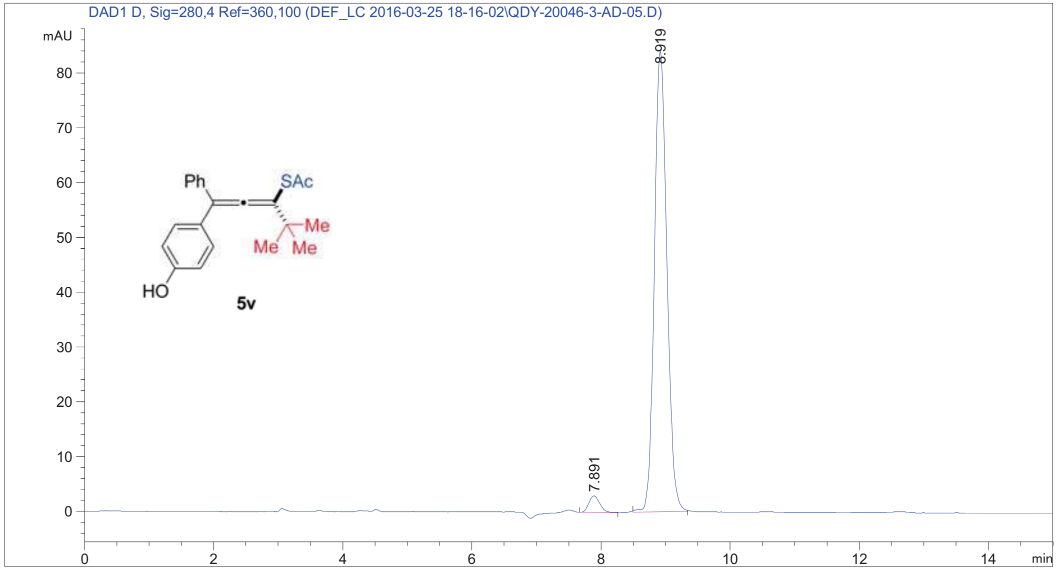

===

Area Percent Report

===

Sorted By : Signal
Multiplier : 1.0000
Dilution : 1.0000
Use Multiplier & Dilution Factor with ISTDs

Signal 1: DAD1 D, Sig=280,4 Ref=360,100

Peak #	RetTime [min]	Type	Width [min]	Area [mAU*s]	Height [mAU]	Area %
1	7.891	BB	0.1840	35.07615	3.00973	3.0115
2	8.919	BB	0.2069	1129.64929	83.97672	96.9885

Totals : 1164.72544 86.98645

===

*** End of Report ***

Supplementary Figure 209. HPLC spectrum for 5v

Data File C:\CHEM32\1\DATA\DEF_LC 2016-03-05 14-20-31\QDY-20069-4-AD-20.D
Sample Name:

===

Acq. Operator : Seq. Line : 19
Acq. Instrument : Instrument 1 Location : Vial 34
Injection Date : 3/5/2016 8:13:02 PM Inj : 1
Inj Volume : 5.000 µl
Acq. Method : C:\CHEM32\1\DATA\DEF_LC 2016-03-05 14-20-31\AD-20-30.M
Last changed : 3/5/2016 8:29:34 PM
(modified after loading)
Analysis Method : C:\CHEM32\1\METHODS\AD-01-10-2.0.M
Last changed : 6/26/2016 2:50:44 PM
(modified after loading)
Additional Info : Peak(s) manually integrated

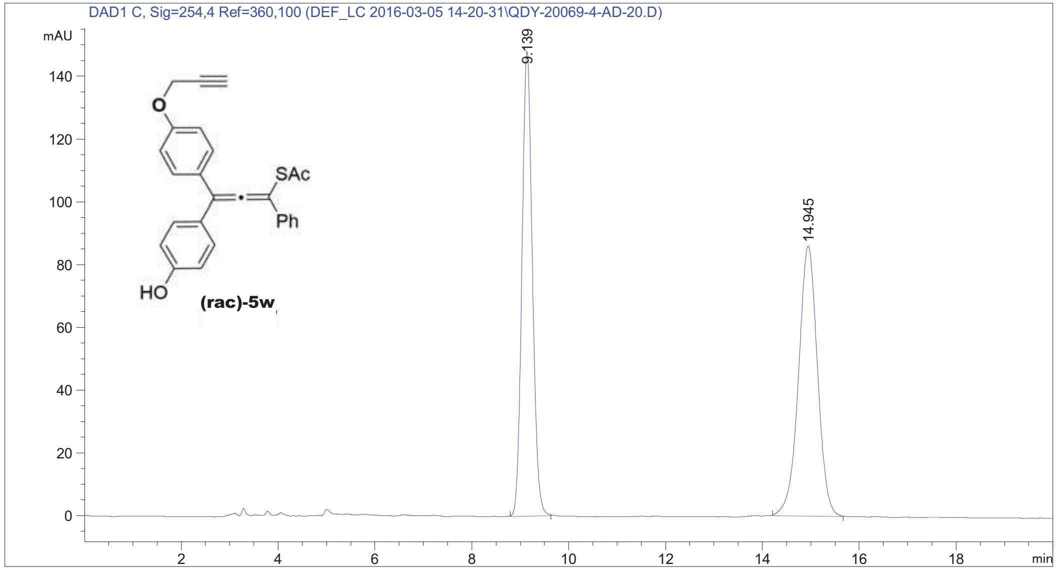

===

Area Percent Report

===

Sorted By : Signal
Multiplier : 1.0000
Dilution : 1.0000
Use Multiplier & Dilution Factor with ISTDs

Signal 1: DAD1 C, Sig=254,4 Ref=360,100

Peak #	RetTime [min]	Type	Width [min]	Area [mAU*s]	Height [mAU]	Area %
1	9.139	BB	0.2304	2214.57837	148.15285	48.5121
2	14.945	BB	0.4185	2350.42090	86.10274	51.4879
Totals :				4564.99927	234.25558	

Data File C:\CHEM32\1\DATA\DEF_LC 2016-04-29 18-48-49\QDY-20133-3-AD-10.D
Sample Name:

===

Acq. Operator : Seq. Line : 14
Acq. Instrument : Instrument 1 Location : Vial 36
Injection Date : 4/29/2016 11:17:11 PM Inj : 1
Inj Volume : 5.000 µl
Acq. Method : C:\CHEM32\1\DATA\DEF_LC 2016-04-29 18-48-49\AD-20-20.M
Last changed : 4/29/2016 10:34:02 PM
(modified after loading)
Analysis Method : C:\CHEM32\1\METHODS\AD-01-10-2.0.M
Last changed : 6/26/2016 2:50:44 PM
(modified after loading)
Additional Info : Peak(s) manually integrated

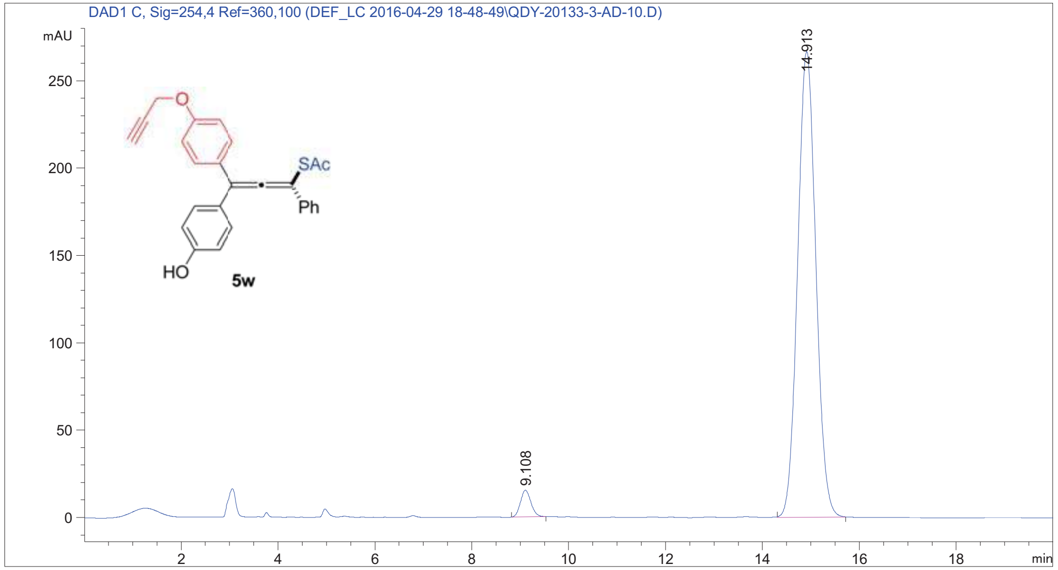

===

Area Percent Report

===

Sorted By : Signal
Multiplier : 1.0000
Dilution : 1.0000
Use Multiplier & Dilution Factor with ISTDs

Signal 1: DAD1 C, Sig=254,4 Ref=360,100

Peak #	RetTime [min]	Type	Width [min]	Area [mAU*s]	Height [mAU]	Area %
1	9.108	BB	0.2353	233.07611	15.33749	3.2413
2	14.913	BB	0.4063	6957.69434	266.73419	96.7587
Totals :				7190.77045	282.07168	

Supplementary Figure 210. HPLC spectrum for 5w

Data File C:\CHEM32\1\DATA\DEF_LC 2016-04-27 16-08-08\QDY-20130-2-AD-15.D
Sample Name:

===

Acq. Operator : Seq. Line : 27
Acq. Instrument : Instrument 1 Location : Vial 32
Injection Date : 4/28/2016 2:11:46 AM Inj : 1
Inj Volume : 5.000 µl
Acq. Method : C:\CHEM32\1\DATA\DEF_LC 2016-04-27 16-08-08\AD-15-30.M
Last changed : 4/28/2016 1:39:09 AM
(modified after loading)
Analysis Method : C:\CHEM32\1\METHODS\AD-01-10-2.0.M
Last changed : 6/26/2016 3:48:19 PM
(modified after loading)
Additional Info : Peak(s) manually integrated

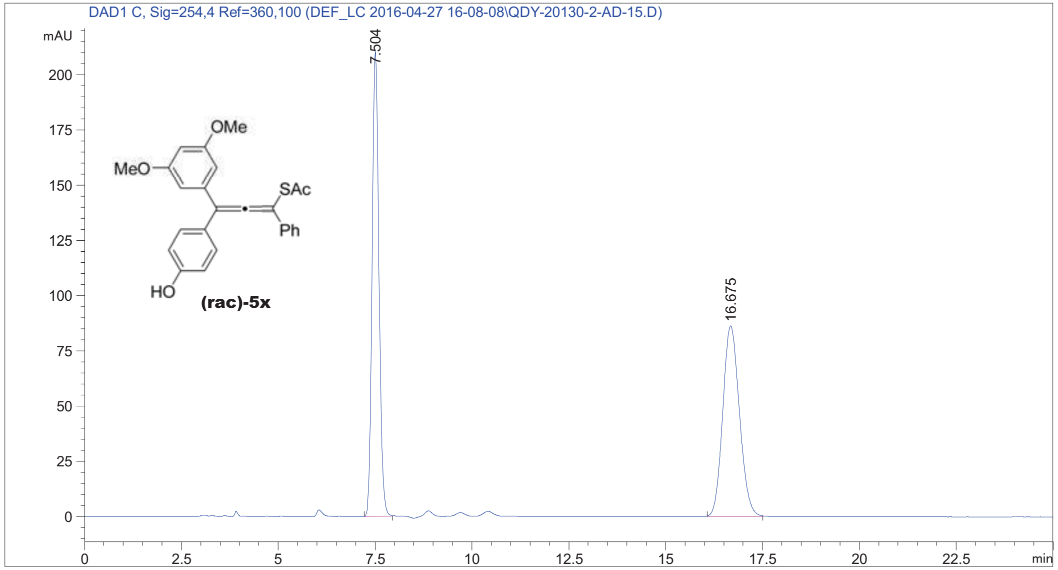

===

Area Percent Report

===

Sorted By : Signal
Multiplier : 1.0000
Dilution : 1.0000
Use Multiplier & Dilution Factor with ISTDs

Signal 1: DAD1 C, Sig=254,4 Ref=360,100

Peak #	RetTime [min]	Type	Width [min]	Area [mAU*s]	Height [mAU]	Area %
1	7.504	BB	0.1895	2587.34521	210.38361	50.0512
2	16.675	BB	0.4629	2582.05249	86.33402	49.9488
Totals :				5169.39771	296.71763	

Data File C:\CHEM32\1\DATA\DEF_LC 2016-04-28 23-38-49\QDY-20131-2-ADH-15.D
Sample Name:

===

Acq. Operator : Seq. Line : 7
Acq. Instrument : Instrument 1 Location : Vial 32
Injection Date : 4/29/2016 1:42:50 AM Inj : 1
Inj Volume : 5.000 µl
Acq. Method : C:\CHEM32\1\DATA\DEF_LC 2016-04-28 23-38-49\AD-15-30.M
Last changed : 4/29/2016 1:41:58 AM
(modified after loading)
Analysis Method : C:\CHEM32\1\METHODS\AD-01-10-2.0.M
Last changed : 6/26/2016 3:48:19 PM
(modified after loading)
Additional Info : Peak(s) manually integrated

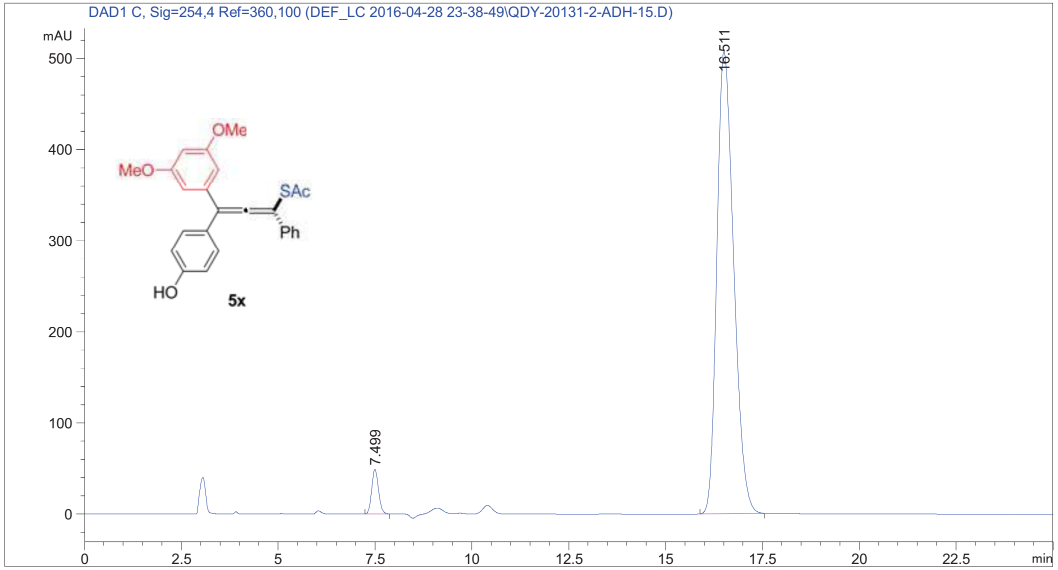

===

Area Percent Report

===

Sorted By : Signal
Multiplier : 1.0000
Dilution : 1.0000
Use Multiplier & Dilution Factor with ISTDs

Signal 1: DAD1 C, Sig=254,4 Ref=360,100

Peak #	RetTime [min]	Type	Width [min]	Area [mAU*s]	Height [mAU]	Area %
1	7.499	BB	0.1892	601.64008	49.02268	3.7736
2	16.511	BB	0.4687	1.53417e4	507.45258	96.2264
Totals :				1.59434e4	556.47525	

Supplementary Figure 211. HPLC spectrum for 5x

Data File C:\CHEM32\1\DATA\DEF_LC 2016-05-03 22-13-04\QDY-20134-6-AD-05.D
Sample Name:

===

Acq. Operator : Seq. Line : 9
Acq. Instrument : Instrument 1 Location : Vial 33
Injection Date : 5/4/2016 12:44:24 AM Inj : 1
Inj Volume : 5.000 µl
Acq. Method : C:\CHEM32\1\DATA\DEF_LC 2016-05-03 22-13-04\AD-05-20.M
Last changed : 5/4/2016 12:43:31 AM
(modified after loading)
Analysis Method : C:\CHEM32\1\METHODS\AD-01-10-2.0.M
Last changed : 6/26/2016 8:40:34 PM
(modified after loading)
Additional Info : Peak(s) manually integrated

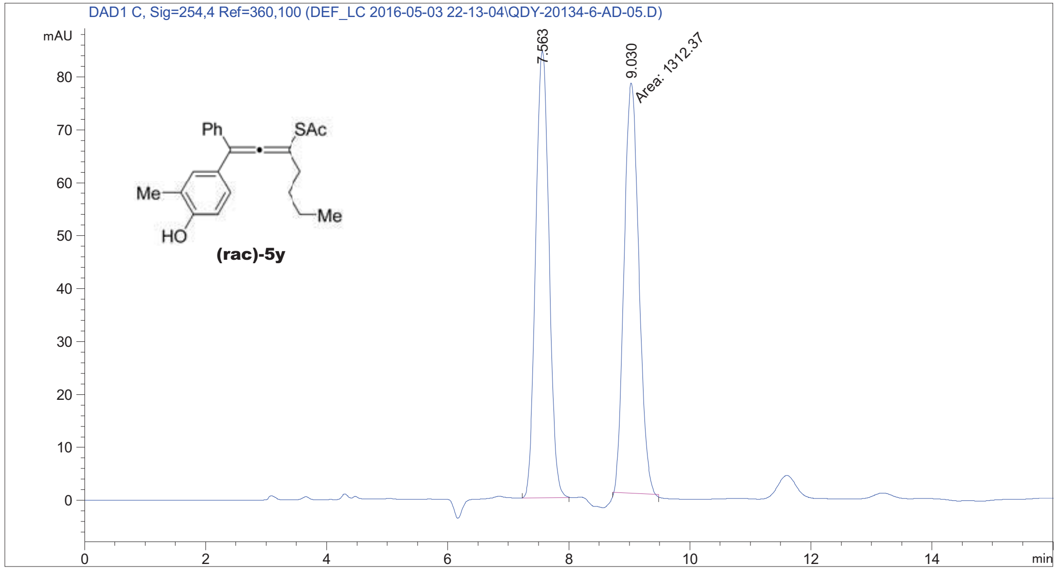

===

Area Percent Report

===

Sorted By : Signal
Multiplier : 1.0000
Dilution : 1.0000
Use Multiplier & Dilution Factor with ISTDs

Signal 1: DAD1 C, Sig=254,4 Ref=360,100

Peak #	RetTime [min]	Type	Width [min]	Area [mAU*s]	Height [mAU]	Area %
1	7.563	BB	0.2457	1298.89539	84.33715	49.7420
2	9.030	MM	0.2822	1312.37000	77.51844	50.2580

Totals : 2611.26538 161.85559

Data File C:\CHEM32\1\DATA\DEF_LC 2016-05-06 09-29-35\QDY-20140-1-ADH-05.D
Sample Name:

===

Acq. Operator : Seq. Line : 23
Acq. Instrument : Instrument 1 Location : Vial 31
Injection Date : 5/6/2016 4:22:16 PM Inj : 1
Inj Volume : 5.000 µl
Acq. Method : C:\CHEM32\1\DATA\DEF_LC 2016-05-06 09-29-35\AD-05-20.M
Last changed : 5/6/2016 4:21:24 PM
(modified after loading)
Analysis Method : C:\CHEM32\1\METHODS\AD-01-10-2.0.M
Last changed : 6/26/2016 8:40:34 PM
(modified after loading)
Additional Info : Peak(s) manually integrated

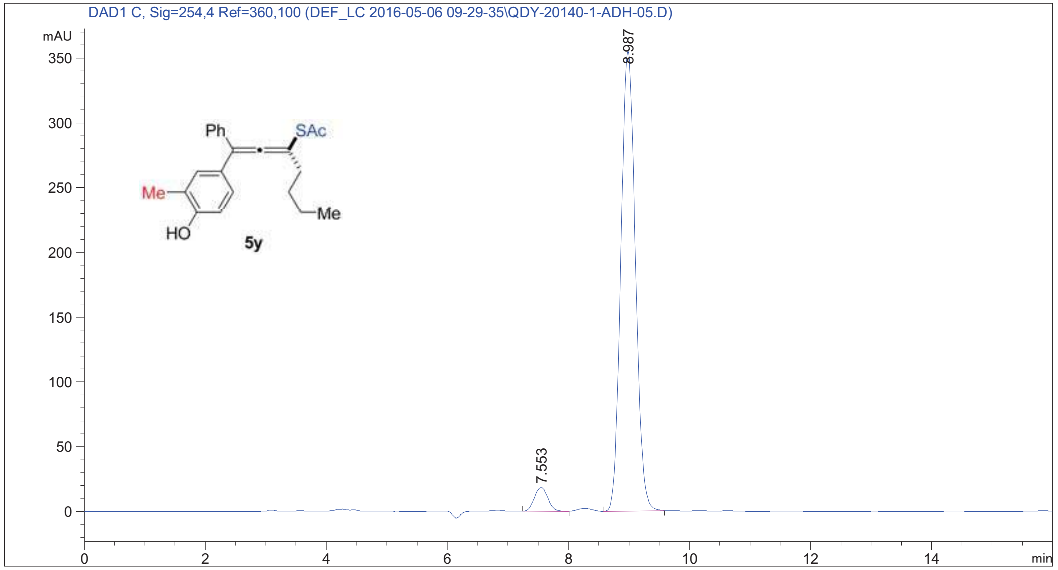

===

Area Percent Report

===

Sorted By : Signal
Multiplier : 1.0000
Dilution : 1.0000
Use Multiplier & Dilution Factor with ISTDs

Signal 1: DAD1 C, Sig=254,4 Ref=360,100

Peak #	RetTime [min]	Type	Width [min]	Area [mAU*s]	Height [mAU]	Area %
1	7.553	BB	0.2492	285.28354	18.37008	4.5947
2	8.987	VB	0.2628	5923.73438	354.80585	95.4053

Totals : 6209.01791 373.17592

Supplementary Figure 212. HPLC spectrum for 5y

Data File C:\CHEM32\1\DATA\QDY 2016-08-15 15-27-20\QDY-30054-1-AD-05.D
Sample Name:

===

Acq. Operator : Seq. Line : 22
Acq. Instrument : Instrument 1 Location : Vial 33
Injection Date : 8/15/2016 10:28:34 PM Inj : 1
Inj Volume : 5.000 µl
Acq. Method : C:\CHEM32\1\DATA\QDY 2016-08-15 15-27-20\AD-05-20.M
Last changed : 10/14/2015 3:51:32 PM
Analysis Method : C:\CHEM32\1\METHODS\OD-2.5-30.M
Last changed : 8/6/2016 3:47:03 PM
Additional Info : Peak(s) manually integrated

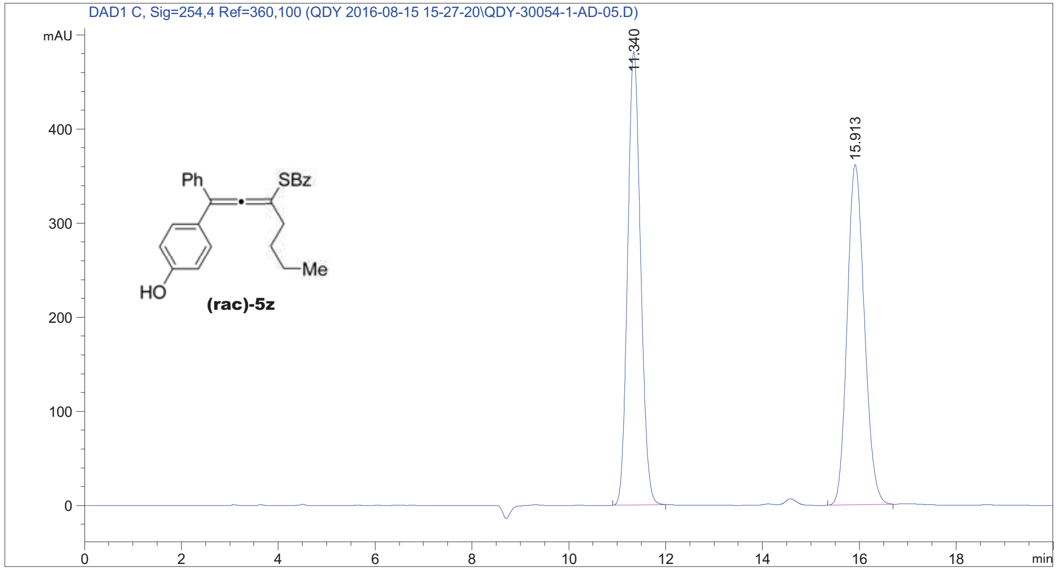

===

Area Percent Report

===

Sorted By : Signal
Multiplier : 1.0000
Dilution : 1.0000
Use Multiplier & Dilution Factor with ISTDs

Signal 1: DAD1 C, Sig=254,4 Ref=360,100

Peak #	RetTime [min]	Type	Width [min]	Area [mAU*s]	Height [mAU]	Area %
1	11.340	BB	0.2887	8956.43457	482.23285	50.1377
2	15.913	BB	0.3834	8907.23926	361.53693	49.8623

Totals : 1.78637e4 843.76978

===

*** End of Report ***

Instrument 1 8/15/2016 11:00:25 PM Page 1 of 1

Data File C:\CHEM32\1\DATA\QDY 2016-08-18 13-18-55\QDY-30054-6-AD--05.D
Sample Name:

===

Acq. Operator : Seq. Line : 29
Acq. Instrument : Instrument 1 Location : Vial 33
Injection Date : 8/18/2016 10:32:40 PM Inj : 1
Inj Volume : 5.000 µl
Acq. Method : C:\CHEM32\1\DATA\QDY 2016-08-18 13-18-55\AD-05-20.M
Last changed : 10/14/2015 3:51:32 PM
Analysis Method : C:\CHEM32\1\METHODS\IC-15-30.M
Last changed : 8/18/2016 11:16:41 PM
(modified after loading)
Additional Info : Peak(s) manually integrated

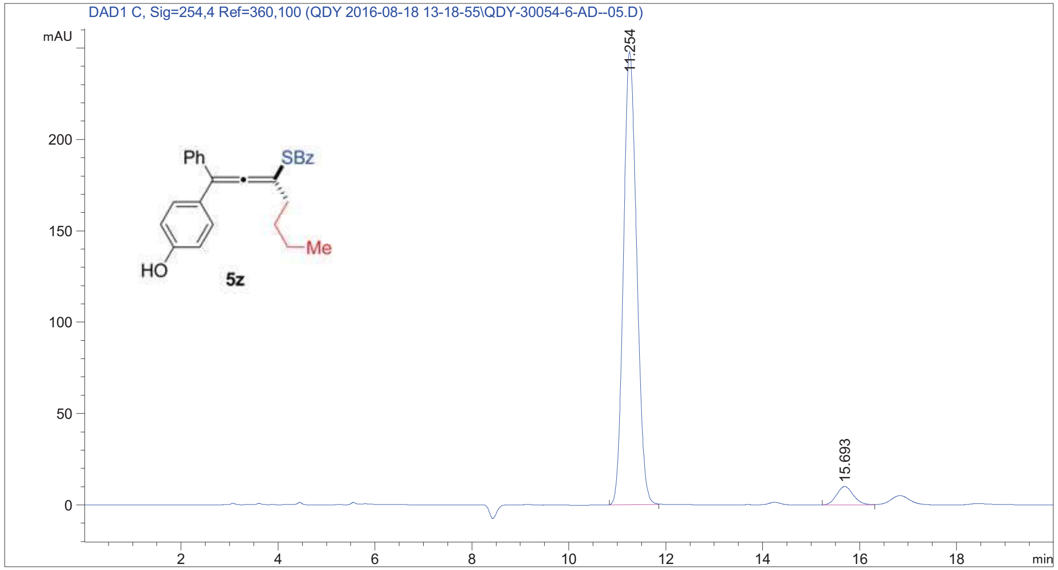

===

Area Percent Report

===

Sorted By : Signal
Multiplier : 1.0000
Dilution : 1.0000
Use Multiplier & Dilution Factor with ISTDs

Signal 1: DAD1 C, Sig=254,4 Ref=360,100

Peak #	RetTime [min]	Type	Width [min]	Area [mAU*s]	Height [mAU]	Area %
1	11.254	BB	0.2970	4696.32715	247.93793	95.0618
2	15.693	BB	0.3739	243.95964	10.09740	4.9382

Totals : 4940.28679 258.03533

===

Instrument 1 8/18/2016 11:16:50 PM Page 1 of 1

Supplementary Figure 213. HPLC spectrum for 5z

```
Data File C:\CHEM32\1\DATA\QDY 2016-06-23 22-12-46\QDY-20174-2-OD-01-05.D
Sample Name:

    =====================================================================
    Acq. Operator   :                               Seq. Line :    7
    Acq. Instrument : Instrument 1                   Location : Vial 33
    Injection Date  : 6/23/2016 11:51:23 PM               Inj :    1
                                                   Inj Volume : 5.000 µl
    Acq. Method     : C:\CHEM32\1\DATA\QDY 2016-06-23 22-12-46\OD-01-20-0.5.M
    Last changed    : 6/23/2016 11:50:31 PM
                      (modified after loading)
    Analysis Method : C:\CHEM32\1\METHODS\AD-25-90.M
    Last changed    : 6/24/2016 12:31:56 AM
                      (modified after loading)
    Additional Info : Peak(s) manually integrated
```

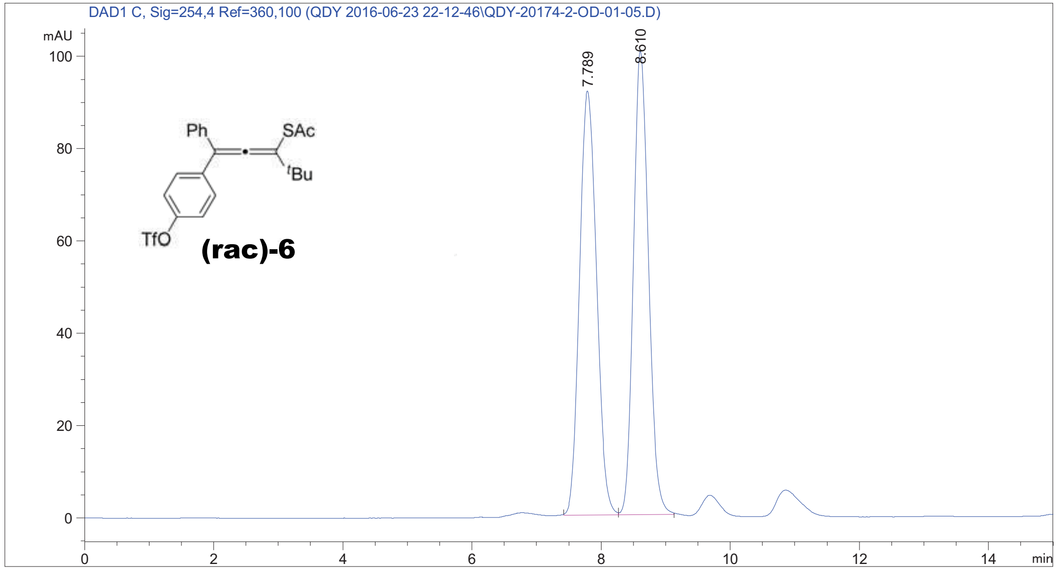


```
    =====================================================================
                            Area Percent Report
    =====================================================================

    Sorted By             :      Signal
    Multiplier            :      1.0000
    Dilution              :      1.0000
    Use Multiplier & Dilution Factor with ISTDs


    Signal 1: DAD1 C, Sig=254,4 Ref=360,100

    Peak RetTime Type  Width     Area      Height     Area
      #   [min]        [min]   [mAU*s]     [mAU]        %
    ----|-------|----|-------|----------|----------|--------|
       1   7.789 BV    0.2921 1686.98352   91.91465  50.7609
       2   8.610 VB    0.2561 1636.40552  100.41180  49.2391

    Totals :                  3323.38904  192.32645
```

```
Data File C:\CHEM32\1\DATA\QDY 2016-06-23 16-11-05\QDY-20181-2-OD-01-05.D
Sample Name:

    =====================================================================
    Acq. Operator   :                               Seq. Line :    4
    Acq. Instrument : Instrument 1                   Location : Vial 32
    Injection Date  : 6/23/2016 5:07:25 PM                Inj :    1
                                                   Inj Volume : 5.000 µl
    Acq. Method     : C:\CHEM32\1\DATA\QDY 2016-06-23 16-11-05\OD-01-20-0.5.M
    Last changed    : 6/23/2016 5:06:32 PM
                      (modified after loading)
    Analysis Method : C:\CHEM32\1\METHODS\AD-25-90.M
    Last changed    : 6/23/2016 11:05:26 PM
                      (modified after loading)
    Additional Info : Peak(s) manually integrated
```

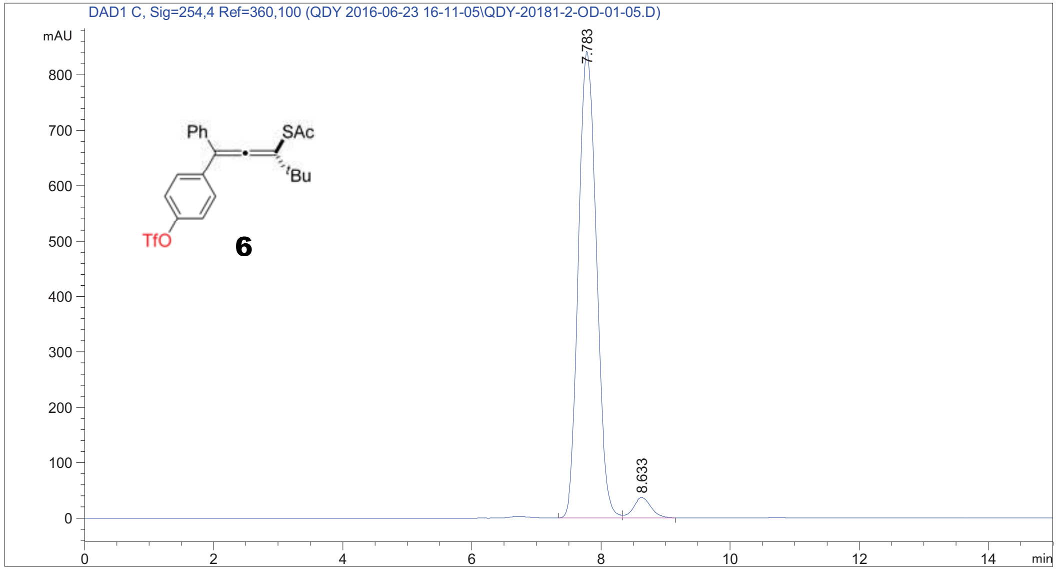


```
    =====================================================================
                            Area Percent Report
    =====================================================================

    Sorted By             :      Signal
    Multiplier            :      1.0000
    Dilution              :      1.0000
    Use Multiplier & Dilution Factor with ISTDs


    Signal 1: DAD1 C, Sig=254,4 Ref=360,100

    Peak RetTime Type  Width     Area      Height     Area
      #   [min]        [min]   [mAU*s]     [mAU]        %
    ----|-------|----|-------|----------|----------|--------|
       1   7.783 BV    0.3130 1.63889e4  842.21082  95.9034
       2   8.633 VB    0.2896  700.05945  36.86123   4.0966

    Totals :                  1.70890e4  879.07204
```

Supplementary Figure 214. HPLC spectrum for 6

```
Data File C:\CHEM32\1\DATA\QDY 2016-06-20 22-33-20\QDY-20179-2-AD-05.D
Sample Name:

    =====================================================================
    Acq. Operator   :                               Seq. Line :    2
    Acq. Instrument : Instrument 1                   Location : Vial 33
    Injection Date  : 6/20/2016 10:47:22 PM                Inj :    1
                                                   Inj Volume : 5.000 µl
    Different Inj Volume from Sequence !     Actual Inj Volume : 10.000 µl
    Acq. Method     : C:\CHEM32\1\DATA\QDY 2016-06-20 22-33-20\AD-05-30.M
    Last changed    : 6/20/2016 10:59:56 PM
                      (modified after loading)
    Analysis Method : C:\CHEM32\1\METHODS\AD-01-10-2.0.M
    Last changed    : 6/26/2016 1:59:23 PM
                      (modified after loading)
    Additional Info : Peak(s) manually integrated
```

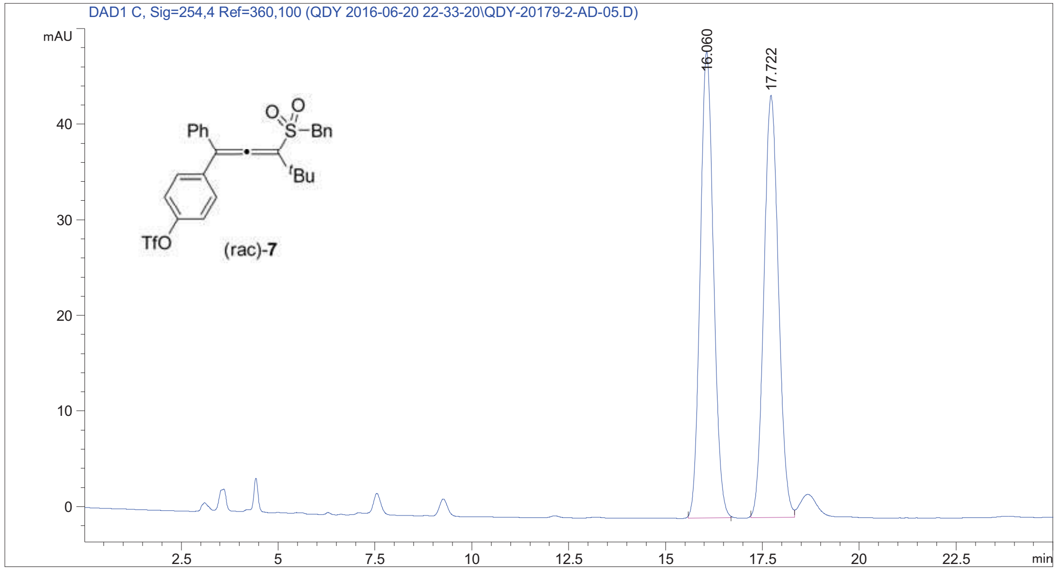


```
    =====================================================================
                             Area Percent Report
    =====================================================================

    Sorted By             :      Signal
    Multiplier            :      1.0000
    Dilution              :      1.0000
    Use Multiplier & Dilution Factor with ISTDs


    Signal 1: DAD1 C, Sig=254,4 Ref=360,100

    Peak RetTime Type  Width     Area      Height     Area
      #   [min]        [min]   [mAU*s]     [mAU]        %
    ----|-------|----|-------|----------|----------|--------|
       1  16.060 BB    0.3694 1159.83057   48.78622  50.0884
       2  17.722 BV    0.4073 1155.73547   44.15915  49.9116

    Totals :                  2315.56604   92.94537
```



```
Data File C:\CHEM32\1\DATA\QDY 2016-06-25 14-37-21\QDY-20181-3-BOTTOM.D
Sample Name:

    =====================================================================
    Acq. Operator   :                               Seq. Line :    3
    Acq. Instrument : Instrument 1                   Location : Vial 32
    Injection Date  : 6/25/2016 3:17:25 PM                 Inj :    1
                                                   Inj Volume : 5.000 µl
    Different Inj Volume from Sequence !     Actual Inj Volume : 3.000 µl
    Acq. Method     : C:\CHEM32\1\DATA\QDY 2016-06-25 14-37-21\AD-05-30.M
    Last changed    : 6/25/2016 2:57:48 PM
                      (modified after loading)
    Analysis Method : C:\CHEM32\1\METHODS\AD-01-10-2.0.M
    Last changed    : 6/26/2016 1:59:23 PM
                      (modified after loading)
    Additional Info : Peak(s) manually integrated
```

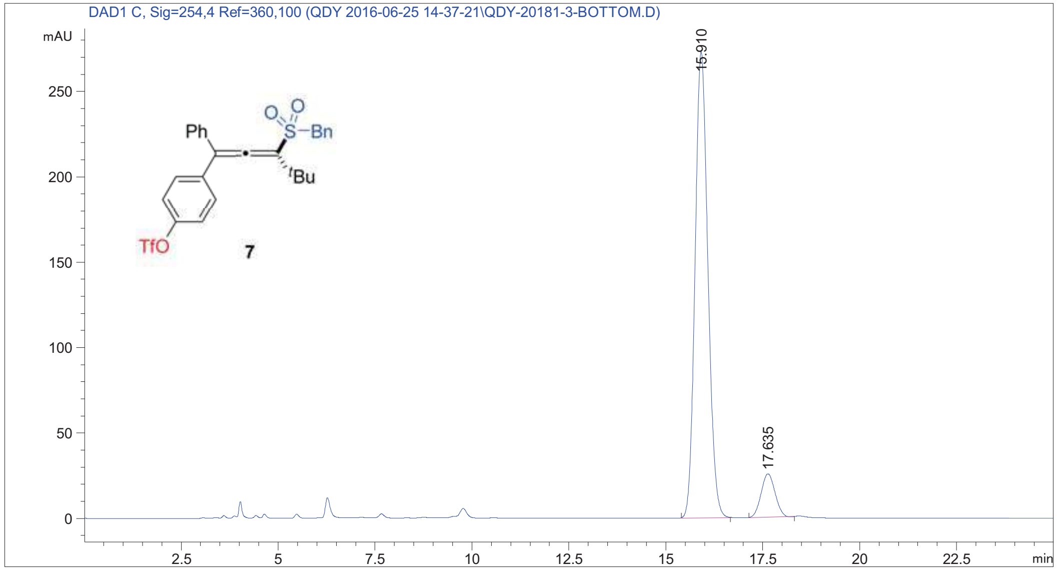


```
    =====================================================================
                             Area Percent Report
    =====================================================================

    Sorted By             :      Signal
    Multiplier            :      1.0000
    Dilution              :      1.0000
    Use Multiplier & Dilution Factor with ISTDs


    Signal 1: DAD1 C, Sig=254,4 Ref=360,100

    Peak RetTime Type  Width     Area      Height     Area
      #   [min]        [min]   [mAU*s]     [mAU]        %
    ----|-------|----|-------|----------|----------|--------|
       1  15.910 BB    0.3581 6275.77734  273.06549  90.8933
       2  17.635 BB    0.3896  628.77716   25.33145   9.1067

    Totals :                  6904.55450  298.39694
```

Supplementary Figure 215. HPLC spectrum for 7

Data File C:\CHEM32\1\DATA\QDY 2017-01-13 10-06-53\QDY-40037-2-AD-05.D
Sample Name:

===

Acq. Operator : Seq. Line : 12
Acq. Instrument : Instrument 1 Location : Vial 31
Injection Date : 1/13/2017 2:11:06 PM Inj : 1
Inj Volume : 5.000 µl
Acq. Method : C:\CHEM32\1\DATA\QDY 2017-01-13 10-06-53\AD-05-20.M
Last changed : 1/13/2017 2:10:15 PM
(modified after loading)
Analysis Method : C:\CHEM32\1\METHODS\ETOH-IC-01-25-05.M
Last changed : 1/15/2017 4:11:48 PM
(modified after loading)
Additional Info : Peak(s) manually integrated

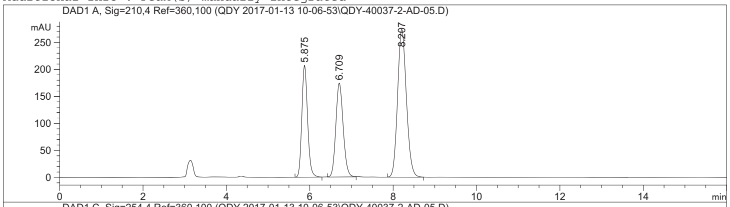

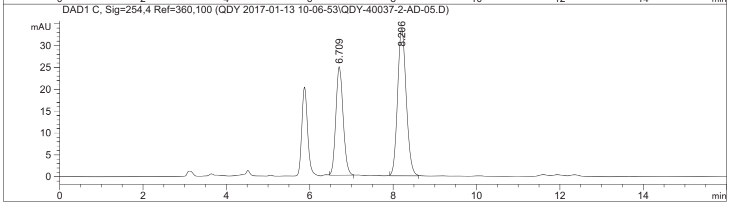

===

Area Percent Report

===

Sorted By : Signal
Multiplier : 1.0000
Dilution : 1.0000
Use Multiplier & Dilution Factor with ISTDs

Signal 1: DAD1 A, Sig=210,4 Ref=360,100

Peak #	RetTime [min]	Type	Width [min]	Area [mAU*s]	Height [mAU]	Area %
1	5.875	BB	0.1481	1992.61841	207.29070	24.4290
2	6.709	BB	0.1912	2136.81909	174.07649	26.1968
3	8.207	BB	0.2275	4027.34155	273.93179	49.3742

Totals : 8156.77905 655.29898

Data File C:\CHEM32\1\DATA\QDY 2017-01-13 10-06-53\QDY-40037-2-AD-05.D
Sample Name:

Signal 2: DAD1 C, Sig=254,4 Ref=360,100

Peak #	RetTime [min]	Type	Width [min]	Area [mAU*s]	Height [mAU]	Area %
1	6.709	BB	0.1883	302.87158	24.83160	38.4254
2	8.206	BB	0.2240	485.33475	33.69499	61.5746

Totals : 788.20633 58.52659

===

*** End of Report ***

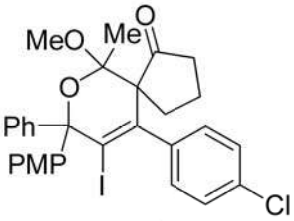

(rac)-8

Data File C:\CHEM32\1\DATA\QDY 2017-01-13 10-06-53\QDY-40037-5-ADH-05.D
Sample Name:

===

Acq. Operator : Seq. Line : 32
Acq. Instrument : Instrument 1 Location : Vial 33
Injection Date : 1/13/2017 8:13:46 PM Inj : 1
Inj Volume : 5.000 µl
Acq. Method : C:\CHEM32\1\DATA\QDY 2017-01-13 10-06-53\AD-05-20.M
Last changed : 10/14/2015 3:51:32 PM
Analysis Method : C:\CHEM32\1\METHODS\ETOH-IC-01-25-05.M
Last changed : 1/15/2017 4:11:48 PM
(modified after loading)
Additional Info : Peak(s) manually integrated

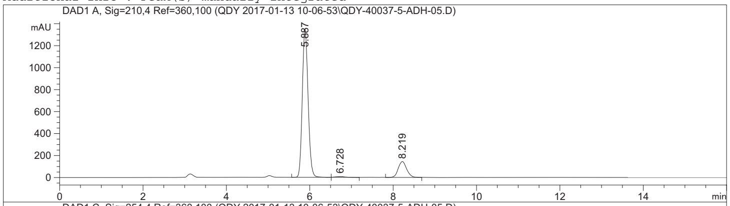

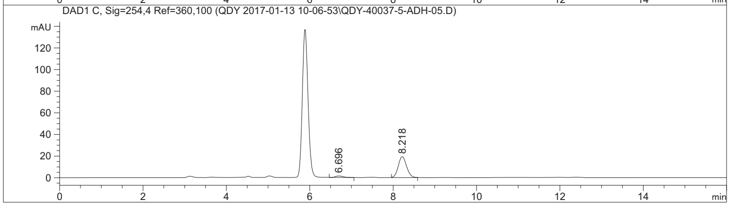

===

Area Percent Report

===

Sorted By : Signal
Multiplier : 1.0000
Dilution : 1.0000
Use Multiplier & Dilution Factor with ISTDs

Signal 1: DAD1 A, Sig=210,4 Ref=360,100

Peak #	RetTime [min]	Type	Width [min]	Area [mAU*s]	Height [mAU]	Area %
1	5.887	BB	0.1505	1.29937e4	1347.09326	85.8763
2	6.728	BB	0.2030	110.11534	8.28580	0.7278
3	8.219	VB	0.2175	2026.89929	144.63719	13.3959

Totals : 1.51307e4 1500.01626

Data File C:\CHEM32\1\DATA\QDY 2017-01-13 10-06-53\QDY-40037-5-ADH-05.D
Sample Name:

Signal 2: DAD1 C, Sig=254,4 Ref=360,100

Peak #	RetTime [min]	Type	Width [min]	Area [mAU*s]	Height [mAU]	Area %
1	6.696	BB	0.2009	17.99112	1.42895	6.2794
2	8.218	BB	0.2132	268.51770	19.43753	93.7206

Totals : 286.50882 20.86649

===

*** End of Report ***

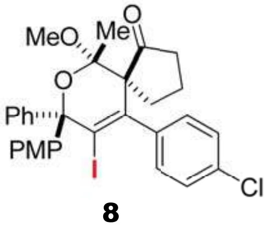

8

Supplementary Figure 216. HPLC spectrum for 8 (AD)

Data File C:\CHEM32\1\DATA\QDY 2017-01-13 23-25-59\QDY-40037-2-IC-0505.D
Sample Name:

Acq. Operator : Seq. Line : 2
Acq. Instrument : Instrument 1 Location : Vial 31
Injection Date : 1/13/2017 11:40:24 PM Inj : 1
Inj Volume : 5.000 µl
Acq. Method : C:\CHEM32\1\DATA\QDY 2017-01-13 23-25-59\IC-05-25-05.M
Last changed : 1/13/2017 11:22:04 PM
Analysis Method : C:\CHEM32\1\METHODS\ETOH-IC-01-25-05.M
Last changed : 1/15/2017 4:07:21 PM
(modified after loading)
Additional Info : Peak(s) manually integrated

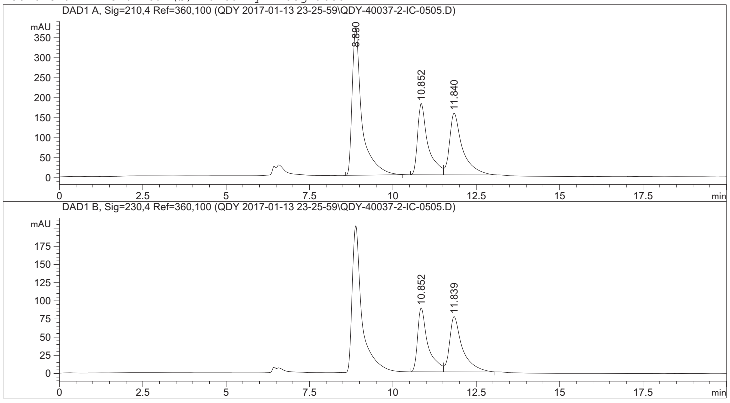

Area Percent Report

Sorted By : Signal
Multiplier : 1.0000
Dilution : 1.0000
Use Multiplier & Dilution Factor with ISTDs

Signal 1: DAD1 A, Sig=210,4 Ref=360,100

Peak #	RetTime [min]	Type	Width [min]	Area [mAU*s]	Height [mAU]	Area %
1	8.890	BB	0.3003	7469.63574	365.63831	48.1861
2	10.852	BV	0.3130	3867.48804	178.32393	24.9489
3	11.840	VB	0.3852	4164.50977	154.16713	26.8650

Totals : 1.55016e4 698.12936

Data File C:\CHEM32\1\DATA\QDY 2017-01-13 23-25-59\QDY-40037-2-IC-0505.D
Sample Name:

Signal 2: DAD1 B, Sig=230,4 Ref=360,100

Peak #	RetTime [min]	Type	Width [min]	Area [mAU*s]	Height [mAU]	Area %
1	10.852	BV	0.3127	1905.97717	87.99619	48.2916
2	11.839	VB	0.3832	2040.83240	76.01822	51.7084

Totals : 3946.80957 164.01440

*** End of Report ***

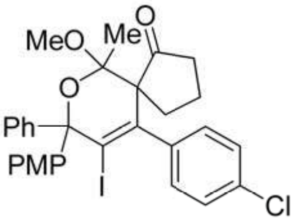

(rac)-8

Data File C:\CHEM32\1\DATA\QDY 2017-01-13 23-25-59\QDY-40037-5-IC-0505.D
Sample Name:

Acq. Operator : Seq. Line : 3
Acq. Instrument : Instrument 1 Location : Vial 33
Injection Date : 1/14/2017 12:06:40 AM Inj : 1
Inj Volume : 5.000 µl
Acq. Method : C:\CHEM32\1\DATA\QDY 2017-01-13 23-25-59\IC-05-25-05.M
Last changed : 1/13/2017 11:22:04 PM
Analysis Method : C:\CHEM32\1\METHODS\ETOH-IC-01-25-05.M
Last changed : 1/15/2017 4:07:21 PM
(modified after loading)
Additional Info : Peak(s) manually integrated

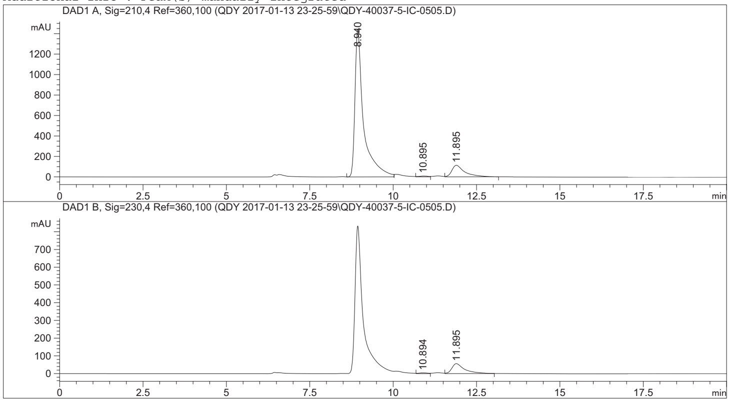

Area Percent Report

Sorted By : Signal
Multiplier : 1.0000
Dilution : 1.0000
Use Multiplier & Dilution Factor with ISTDs

Signal 1: DAD1 A, Sig=210,4 Ref=360,100

Peak #	RetTime [min]	Type	Width [min]	Area [mAU*s]	Height [mAU]	Area %
1	8.940	VV	0.2556	2.59539e4	1444.38599	88.9591
2	10.895	VV	0.2718	167.76729	8.98649	0.5750
3	11.895	VB	0.3894	3053.43042	113.65852	10.4659

Totals : 2.91751e4 1567.03100

Data File C:\CHEM32\1\DATA\QDY 2017-01-13 23-25-59\QDY-40037-5-IC-0505.D
Sample Name:

Signal 2: DAD1 B, Sig=230,4 Ref=360,100

Peak #	RetTime [min]	Type	Width [min]	Area [mAU*s]	Height [mAU]	Area %
1	10.894	BV	0.2714	84.35708	4.52643	5.3166
2	11.895	VB	0.3883	1502.31970	56.11365	94.6834

Totals : 1586.67678 60.64008

*** End of Report ***

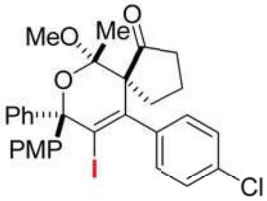

8

Supplementary Figure 217. HPLC spectrum for 8 (IC)

Data File C:\CHEM32\1\DATA\DEF_LC 2016-05-12 13-40-59\QDY-20149-2--AD-05.D
Sample Name:

===

```
Acq. Operator   :                               Seq. Line :  38
Acq. Instrument : Instrument 1                   Location : Vial 32
Injection Date  : 5/13/2016 1:51:46 AM                Inj :   1
                                               Inj Volume : 5.000 µl
Acq. Method     : C:\CHEM32\1\DATA\DEF_LC 2016-05-12 13-40-59\AD-05-20.M
Last changed    : 10/14/2015 3:51:32 PM
Analysis Method : C:\CHEM32\1\METHODS\AD-01-10-2.0.M
Last changed    : 6/26/2016 2:05:13 PM
                  (modified after loading)
Additional Info : Peak(s) manually integrated
```

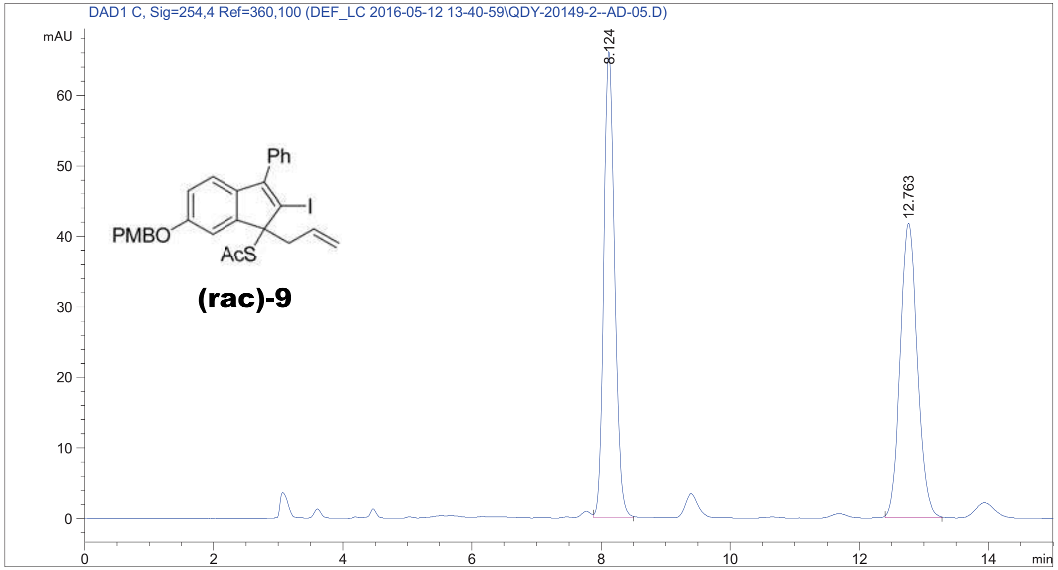

===

Area Percent Report

===

Sorted By : Signal
Multiplier : 1.0000
Dilution : 1.0000
Use Multiplier & Dilution Factor with ISTDs

Signal 1: DAD1 C, Sig=254,4 Ref=360,100

Peak #	RetTime [min]	Type	Width [min]	Area [mAU*s]	Height [mAU]	Area %
1	8.124	VB	0.1801	770.64471	66.04201	49.8542
2	12.763	BB	0.2865	775.15118	41.77250	50.1458

Totals : 1545.79590 107.81450

===

Data File C:\CHEM32\1\DATA\QDY 2016-06-17 14-27-06\QDY-20177-2-AD--05.D
Sample Name:

===

```
Acq. Operator   :                               Seq. Line :  19
Acq. Instrument : Instrument 1                   Location : Vial 32
Injection Date  : 6/17/2016 9:56:27 PM                Inj :   1
                                               Inj Volume : 5.000 µl
Acq. Method     : C:\CHEM32\1\DATA\QDY 2016-06-17 14-27-06\AD-05-30.M
Last changed    : 6/17/2016 10:20:15 PM
                  (modified after loading)
Analysis Method : C:\CHEM32\1\METHODS\IC-20-70.M
Last changed    : 6/17/2016 10:40:01 PM
                  (modified after loading)
Additional Info : Peak(s) manually integrated
```

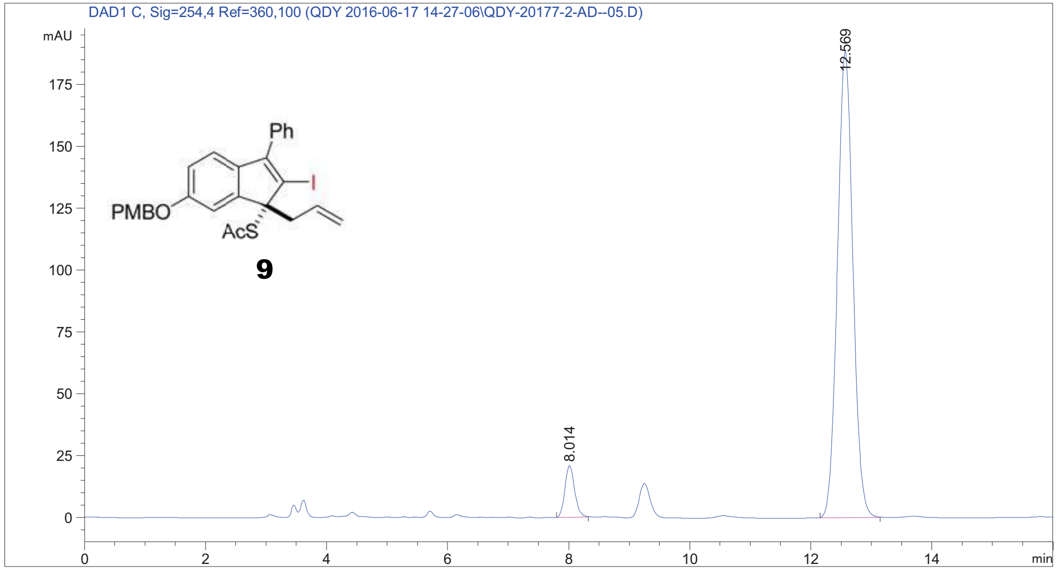

===

Area Percent Report

===

Sorted By : Signal
Multiplier : 1.0000
Dilution : 1.0000
Use Multiplier & Dilution Factor with ISTDs

Signal 1: DAD1 C, Sig=254,4 Ref=360,100

Peak #	RetTime [min]	Type	Width [min]	Area [mAU*s]	Height [mAU]	Area %
1	8.014	BB	0.1721	233.15421	20.90103	6.5009
2	12.569	BB	0.2756	3353.31763	188.42543	93.4991

Totals : 3586.47183 209.32646

Supplementary Figure 218. HPLC spectrum for 9

Supplementary Table 1. Evaluation of Brønsted acid in asymmetric allene synthesis of propargylic alcohol **1** and **2**.[a]

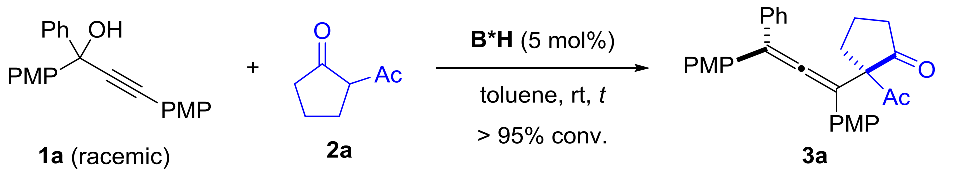

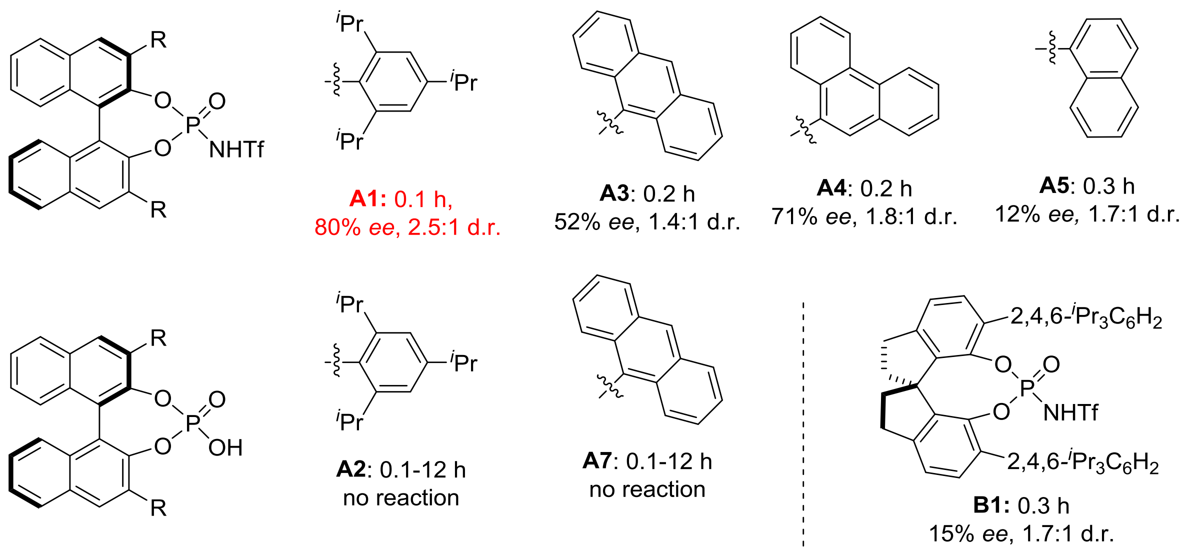

[a] Reaction conditions: **1a** (0.05 mmol), **2a** (0.075 mmol), **B*H** (2.5 μmol), toluene (1.0 mL). The d.r. was determined by ^{1}H NMR analysis. The *ee* was determined by HPLC with a chiral stationary phase. PMP = (*p*-MeO)C_6H_4.

Supplementary Table 2. Solvent and temperature effect on asymmetric allene synthesis of propargylic alcohol **1** and **2**.[a]

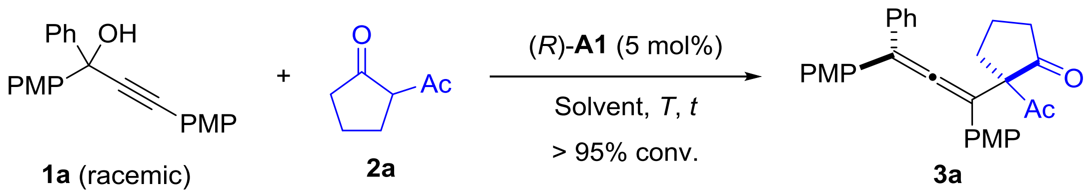

Entry	Solvent	*T* [°C]	*t* [h]	d.r.[b]	*ee* [%][c]
1	toluene	25	0.1	2.5:1	80
2	xylene	25	0.2	2.5:1	73
3	PhF	25	0.2	2.4:1	84
4	CH_2Cl_2	25	0.1	1.6:1	73
5	DCE	25	0.1	1.4:1	72
6	$CHCl_3$	25	0.5	1.6:1	76
7	CCl_4	25	0.1	4.6:1	85
8	Et_2O	25	12	2.8:1	84
9	MTBE	25	12	2.0:1	81
10	CH_3CN	25	0.5	1:1	7
11	hexane	25	3	2.1:1	65
12	cyclohexane	25	1	3.5:1	80
13	hexane/CCl_4 (1:1)	25	0.3	4.3:1	83
14	cyclohexane/CCl_4 (1:1)	25	0.3	4.6:1	84
15	cyclohexane/CCl_4 (3:1)	25	0.5	4.6:1	83
16	cyclohexane/CCl_4 (1:3)	25	0.2	4.6:1	84
17	CCl_4	0	1.5	5.3:1	88
18	CCl_4	−20	3	6.1:1	90[d]

[a] Reaction conditions: **1a** (0.05 mmol), **2a** (0.075 mmol), **A1** (2.5 μmol), solvent (1.0 mL). [b] Determined by ^1H NMR analysis. [c] Determined by HPLC with a chiral stationary phase. [d] **3a** was obtained in 92% isolated yield. PMP = 4-$MeOC_6H_4$.

Supplementary Table 3. Other parameters on asymmetric allene synthesis of propargylic alcohol **1** and **2**.[a]

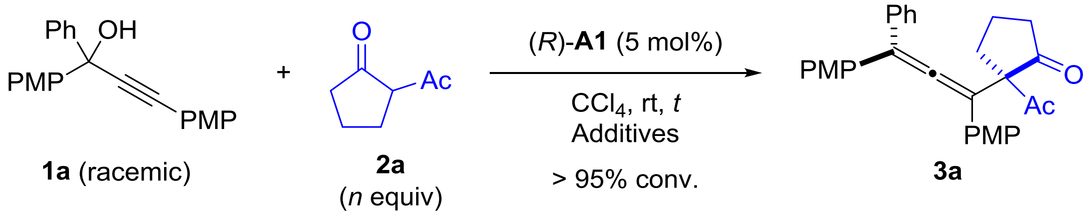

Entry	*n*	Additives	*t* [h]	d.r.[b]	*ee* [%][c]
1	1.1	-	0.3	4.2:1	84
2	1.5	-	0.1	4.6:1	85
3	2.0	-	0.1	4.3:1	85
4[d]	1.5	-	0.1	4.4:1	85
5	1.5	4Å MS (15 mg)	24	4.7:1	85
6	1.5	3Å MS (15 mg)	12	4.7:1	85
7	1.5	$MgSO_4$ (15 mg)	0.1	4.6:1	85

[a] Reaction conditions: **1a** (0.05 mmol), **2a** (0.075 mmol), **A1** (2.5 μmol), solvent (1.0 mL). [b] Determined by ^{1}H NMR analysis. [c] Determined by HPLC with a chiral stationary phase. [d] c = 0.1 M. PMP = 4-$MeOC_6H_4$.

Supplementary Table 4. Reaction optimization on the asymmetric allene synthesis of propargylic alcohol **1** and **4**.

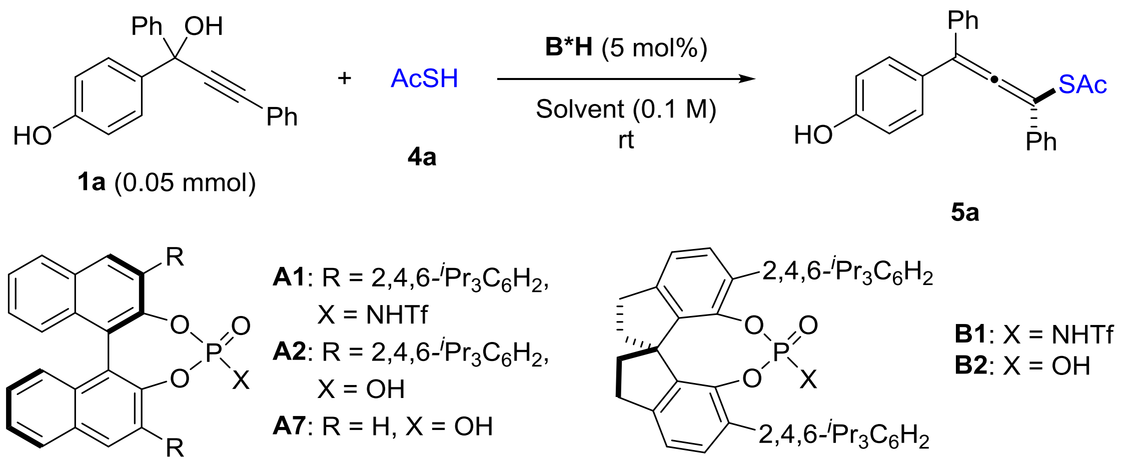

Entry	**B*H**	Solvent	*t* [h]	Yield [%][a]	*ee* [%][a]
1	(*R*)-**A1**	CCl_4	0.5	87	46
2	(*R*)-**A2**	CCl_4	18	85	60
3	(*R*)-**A7**	CCl_4	1.5	65	20
4	(*S*)-**B1**	CCl_4	0.3	83	85
5	(*S*)-**B1**	DCM	0.3	73	81
6	(*S*)-**B2**	DCM	24	51	75
7	(*R*)-**A1**	DCM	0.5	90	43
8	(*S*)-**B1**	Et_2O	12	<5	-
9	(*S*)-**B1**	toluene	1	93	85
10[b]	(*S*)-**B1**	toluene	5	87	89
11[c]	(*S*)-**B1**	toluene	12	<5	-
12[b,d]	(*S*)-**B1**	toluene	6	85	90
13[b,d,e]	(*S*)-**B1**	toluene	12	84	94

[a] Yield was determined by ^1H NMR analysis of the crude mixture. Ee was determined by HPLC with a chiral stationary phase. [b] Run with 3Å molecular sieves (MS, 15 mg). [c] Run 4Å MS (15 mg). [d] c = 0.05 M. [e] Run at –5 °C.

Supplementary Methods

General Procedure A

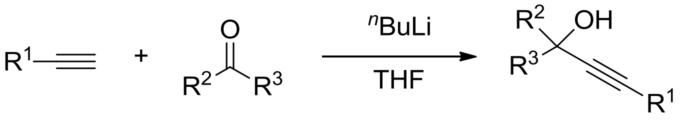

S1 **S2** **1**

At –78 °C under N_2, to an oven-dried flask charged with a solution of the terminal alkyne **S1** (7.5 mmol, 1.5 equiv) in dry THF (10 mL) was added ^{n}BuLi (7.7 mmol, 3.2 mL, 2.4 M in hexane) dropwise. The reaction was stirred for 1 h at the same temperature and then a solution of the corresponding ketone **S2** (5.0 mmol, 1.0 equiv) in THF (5 mL) was added via syringe. The reaction mixture was then gradually warmed up to room temperature. Upon completion (~3 h), the reaction mixture was cooled to 0 °C and a saturated aqueous NH_4Cl solution (10 mL) was added dropwise. The organic layer was separated. The aqueous layer was extracted with ethyl acetate (3×20 mL). The combined organic layers were washed with brine (50 mL), dried over anhydrous Na_2SO_4, filtered, and concentrated. The residue was purified by silica gel chromatography to afford the pure alcohol **1**.

General Procedure B

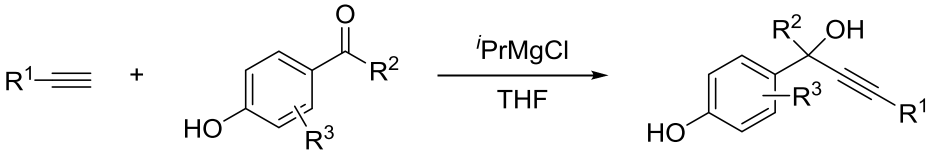

S1 **S3** **1**

Under N_2, to an oven-dried flask charged with a solution of the alkyne **S1** (12.5 mmol, 2.5 equiv) in dry THF (30 mL) was added ^{i}PrMgCl (12.6 mmol, 6.3 mL, 2.0 M in THF) dropwise at 0 °C. After addition, the mixture was warmed to room temperature and stirred at the same temperature for 1 hour. Next, the reaction mixture was cooled to 0 °C, and a solution of the ketone **S3** (5.0 mmol) in THF (5 mL)

was added dropwise. The reaction mixture was then warmed to 60 °C and stirred at the same temperature. The reaction progress was monitored by thin layer chromatography. Upon completion (6-12 h), the reaction mixture was cooled to 0 °C and slowly treated with a saturated aqueous NH_4Cl solution (10 mL). The layers were separated. The aqueous layer was extracted with EtOAc (3×30 mL). The combined organic layers were dried over anhydrous Na_2SO_4, filtered, and concentrated. The residue was purified by silica gel column chromatography to afford the pure alcohol **1**.

1,3-bis(4-Methoxyphenyl)-1-phenylprop-2-yn-1-ol (1a) was prepared as pale yellow oil according to the General Procedure A (purification by flash column chromatography: 10→20% EtOAc in hexanes, 96% yield).

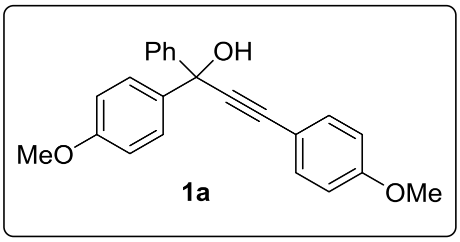

^{1}H NMR (400 MHz, $CDCl_3$) δ 7.68 (d, J = 7.2 Hz, 2H), 7.59 (d, J = 8.8 Hz, 2H), 7.45 (d, J = 8.8 Hz, 2H), 7.36 (t, J = 7.2 Hz, 2H), 7.28 (t, J = 7.2 Hz, 1H), 6.88 (d, J = 8.8 Hz, 2H), 6.86 (d, J = 8.8 Hz, 2H), 3.81 (s, 3H), 3.80 (s, 3H), 2.97 (br s, 1H) ppm.

^{13}C NMR (100 MHz, $CDCl_3$) δ 159.8, 159.0, 145.4, 137.5, 133.2, 128.2, 127.5, 127.4, 126.0, 114.5, 113.9, 113.5, 90.6, 86.9, 74.5, 55.3, 55.2 ppm.

IR (thin film) 3450, 2951, 1603, 1507, 1238, 1169, 1021, 827, 661 cm^{-1}.

HRMS (CI+) Calcd for $C_{23}H_{20}O_3$ [M$^+$]: 344.1412, Found: 344.1420.

3-(4-Chlorophenyl)-1-(4-methoxyphenyl)-1-phenylprop-2-yn-1-ol (1b) as colourless oil according to the General Procedure A (purification by flash column chromatography: 10 →20% EtOAc in hexanes, 81% yield).

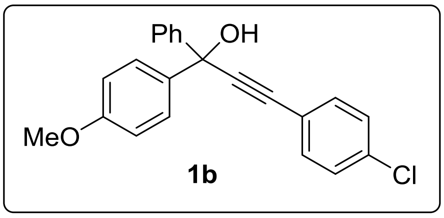

^{1}H NMR (400 MHz, $CDCl_3$) δ 7.66 (d, J = 7.2 Hz, 2H), 7.57 (d, J = 8.8 Hz, 2H), 7.43 (d, J = 8.8 Hz, 2H), 7.37 (t, J = 7.2 Hz, 2H), 7.32-7.27 (m, 3H), 6.88 (d, J = 8.8 Hz, 2H), 3.80 (s, 3H), 2.95 (br s, 1H) ppm.

^{13}C NMR (100 MHz, $CDCl_3$) δ 159.1, 145.0, 137.1, 134.6, 133.0, 128.6, 128.3, 127.7, 127.4, 126.0, 120.9, 113.6, 92.9, 85.8, 74.5, 55.2 ppm.

IR (thin film) 3440, 2933, 1607, 1488, 1246, 1169, 1030, 827, 697 cm^{-1}.

HRMS (CI+) Calcd for $C_{22}H_{17}{}^{35}ClO_2$ [M^+]: 348.0917, Found: 348.0926.

3-(Furan-2-yl)-1-(4-methoxyphenyl)-1-phenylprop-2-yn-1-ol (1c) as brown oil according to the General Procedure A (purification by flash column chromatography: 10→20% EtOAc in hexanes, 81% yield).

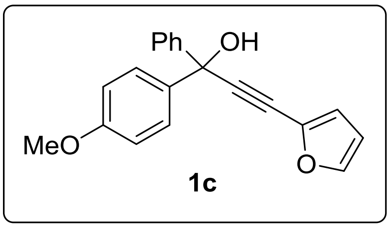

^{1}H NMR (400 MHz, $CDCl_3$) δ 7.64 (d, J = 8.0 Hz, 2H), 7.55 (d, J = 8.8 Hz, 2H), 7.41 (s, 1H), 7.36 (t, J = 7.2 Hz, 2H), 7.30 (d, J = 7.2 Hz, 1H), 6.87 (d, J = 8.8 Hz, 2H), 6.65-6.64 (m, 1H), 6.42-6.39 (m, 1H), 3.80 (s, 3H), 2.94 (br s, 1H) ppm.

^{13}C NMR (100 MHz, $CDCl_3$) δ 159.1, 144.5, 143.8, 136.7, 136.4, 128.3, 127.8, 127.4, 126.0, 115.8, 113.6, 110.9, 95.9, 77.3, 74.5, 55.2 ppm.

IR (thin film) 3418, 2934, 1606, 1508, 1247, 1171, 989, 829, 704 cm^{-1}.

HRMS (CI+) Calcd for $C_{20}H_{16}O_3$ [M^+]: 304.1099, Found: 304.1112.

1-(4-(Allyloxy)phenyl)-3-(4-chlorophenyl)-1-phenylprop-2-yn-1-ol (1d) as colourless oil according to the General Procedure A (purification by flash column chromatography: 10→20% EtOAc in hexanes, 85% yield).

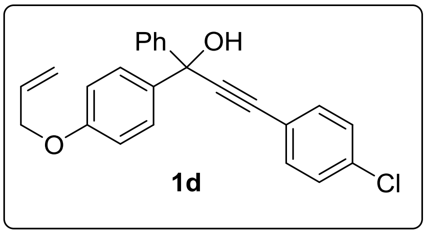

^{1}H NMR (400 MHz, $CDCl_3$) δ 7.66 (d, J = 7.2 Hz, 2H), 7.56 (d, J = 8.8 Hz, 2H), 7.43 (d, J = 8.8 Hz, 2H), 7.37 (t, J = 7.2 Hz, 2H), 7.33-7.27 (m, 3H), 6.88 (d, J = 8.8 Hz, 2H), 6.11-5.99 (m, 1H), 5.41 (d, J = 17.6 Hz, 1H), 5.30 (d, J = 10.4 Hz, 1H), 4.53 (d, J = 5.2 Hz, 2H), 2.92 (br s, 1H) ppm.

^{13}C NMR (100 MHz, $CDCl_3$) δ 158.2, 144.9, 137.2, 134.7, 133.1, 133.0, 128.6, 128.3, 127.7, 127.4, 126.0, 120.9, 117.7, 114.4, 92.9, 85.8, 74.5, 68.8 ppm.

IR (thin film) 3429, 2960, 1606, 1488, 1243, 1170, 1091, 986, 827, 698 cm^{-1}.

HRMS (CI+) Calcd for $C_{24}H_{19}{}^{35}ClO_2$ [M$^+$]: 374.1074, Found: 374.1080.

1,3-bis(4-Methoxyphenyl)-1-phenylprop-2-yn-1-ol (1a) was prepared as pale yellow oil according to the literature procedure [1] (purification by flash column chromatography: 10→20% EtOAc in hexanes, 96% yield).

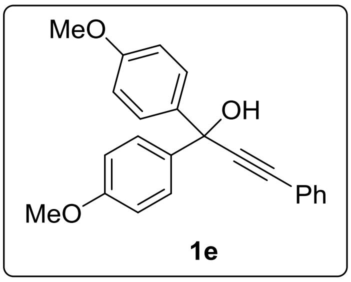

^{1}H NMR (400 MHz, $CDCl_3$) δ 7.59 (d, J = 8.8 Hz, 4H), 7.53-7.50 (m, 2H), 7.35-7.31 (m, 3H), 6.89 (d, J = 8.8 Hz, 4H), 3.80 (s, 6H), 2.98 (br s, 1H) ppm.

^{13}C NMR (100 MHz, $CDCl_3$) δ 159.0, 137.5, 131.7, 128.5, 128.3, 127.3, 122.5, 113.5, 92.1, 86.8, 74.1, 55.2 ppm.

3-(4-Methoxyphenyl)-1-(4-(methylthio)phenyl)-1-phenylprop-2-yn-1-ol (1f) as brown oil according to the General Procedure A (purification by flash column chromatography: 10→20% EtOAc in hexanes, 87% yield).

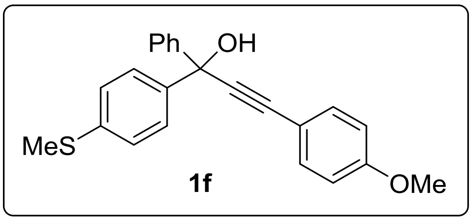

^{1}H NMR (400 MHz, $CDCl_3$) δ 7.68 (d, J = 7.2 Hz, 2H), 7.60 (d, J = 8.8 Hz, 2H), 7.45 (d, J = 8.8 Hz, 2H), 7.36 (t, J = 7.2 Hz, 2H), 7.29 (t, J = 7.2 Hz, 1H), 7.23 (d, J = 8.8 Hz, 2H), 6.86 (d, J = 8.8 Hz, 2H), 3.81 (s, 3H), 2.99 (br s, 1H), 3.47 (s, 3H) ppm.

^{13}C NMR (100 MHz, $CDCl_3$) δ 159.8, 145.0, 142.2, 137.9, 133.2, 128.2, 127.7, 126.6, 126.2, 126.0, 114.4, 113.9, 90.2, 87.2, 74.5, 55.3, 15.2 ppm.

IR (thin film) 3432, 2919, 1604, 1508, 1245, 1170, 1028, 829, 700 cm^{-1}.

HRMS (CI+) Calcd for $C_{23}H_{20}O_2S$ [M$^+$]: 360.1184, Found: 360.1196.

3-(4-Chlorophenyl)-1-(4-methoxyphenyl)-1-(thiophen-2-yl)prop-2-yn-1-ol (1g) as brown oil according to the General Procedure A (purification by flash column chromatography: 10→20% EtOAc in hexanes, 70% yield).

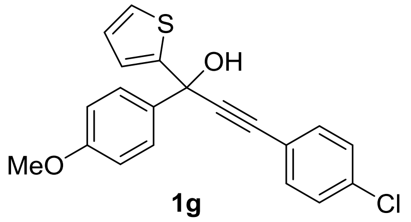

1g

^{1}H NMR (400 MHz, $CDCl_3$) *δ* 7.66 (d, *J* = 8.8 Hz, 2H), 7.43 (d, *J* = 8.8 Hz, 2H), 7.31 (d, *J* = 8.8 Hz, 2H), 7.29-7.26 (m, 1H), 7.12-1.09 (m, 1H), 6.96-6.92 (m, 1H), 6.91 (d, *J* = 8.8 Hz, 2H), 3.81 (s, 3H), 3.17 (br s, 1H) ppm.

^{13}C NMR (100 MHz, $CDCl_3$) *δ* 159.4, 150.3, 136.3, 134.8, 133.0, 128.6, 127.1, 126.5, 125.9, 125.3, 120.6, 113.6, 92.2, 85.2, 71.9, 55.3 ppm.

IR (thin film) 3418, 2933, 1606, 1507, 1247, 1171, 1031, 826, 702 cm^{-1}.

HRMS (CI+) Calcd for $C_{20}H_{15}{}^{35}ClO_2S$ [M^+]: 354.0481, Found: 354.0491.

4-(1-(2-Fluorophenyl)-1-hydroxy-3-phenylprop-2-yn-1-yl)phenol (1h) was prepared as pale yellow solid according to the General Procedure B (purification by flash column chromatography: 20→30% EtOAc in hexanes, 93% yield).

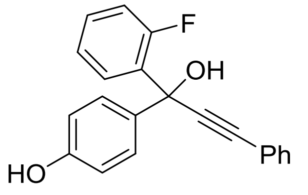

1h

^{1}H NMR (400 MHz, acetone-d_6) *δ* 8.49 (br s, 1H), 7.99 (t, *J* = 8.0 Hz, 1H), 7.51-7.48 (m, 4H), 7.40-7.31 (m, 4H), 7.25 (t, *J* = 7.6 Hz, 1H), 7.06-6.98 (m, 1H), 6.80 (d, *J* = 8.8 Hz, 2H), 5.69(br s, 1H) ppm.

^{13}C NMR (100 MHz, acetone-d_6) *δ* 160.3 (d, $J_{C\text{-}F}$ = 247 Hz), 157.5, 136.5, 134.1 (d, $J_{C\text{-}F}$ = 11 Hz), 132.0, 130.0 (d, $J_{C\text{-}F}$ = 8 Hz), 129.1, 128.2 (2C), 127.6 (d, $J_{C\text{-}F}$ = 3 Hz), 124.2 (d, $J_{C\text{-}F}$ = 4 Hz), 123.5, 116.5 (d, $J_{C\text{-}F}$ = 22 Hz), 115.1, 92.2, 85.7, 71.3 ppm.

^{19}F NMR (376 MHz, acetone-d_6) *δ* –112.1 ppm.

IR (thin film) 3383, 1613, 1512, 1453, 1224, 1170, 989, 837, 757 cm^{-1}.

HRMS (CI+) Calcd for $C_{21}H_{15}FO_2$ [M^+]: 318.1056, Found: 318.1072.

4-(1-Hydroxy-1,3-diphenylprop-2-yn-1-yl)phenol (1i) was prepared as pale yellow solid according to the General Procedure B (purification by flash column chromatography: 20→30% EtOAc in hexanes, 87% yield).

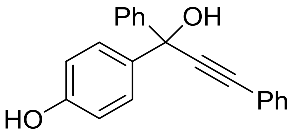

1i

^{1}H NMR (400 MHz, acetone-*d*$_6$) δ 8.34 (br s, 1H), 7.70 (d, *J* = 8.4 Hz, 2H), 7.58-7.50 (m, 4H), 7.41-7.37 (m, 3H), 7.34 (t, *J* = 7.2 Hz, 2H), 7.24 (t, *J* = 7.2 Hz, 1H), 6.81 (d, *J* = 8.8 Hz, 2H), 5.61 (br s, 1H) ppm.

^{13}C NMR (100 MHz, acetone-*d*$_6$) δ 157.2, 147.4, 138.0, 132.0, 129.1, 129.0, 128.5, 128.0, 127.6, 126.5, 123.6, 115.2, 93.9, 86.2, 74.2 ppm.

IR (thin film) 3382, 1598, 1511, 1446, 1236, 1170, 986, 836, 756 cm^{-1}.

HRMS (CI+) Calcd for $C_{21}H_{16}O_2$ [M$^+$]: 300.1150, Found: 300.1152.

4-(1-Hydroxy-3-(4-methoxyphenyl)-1-phenylprop-2-yn-1-yl)phenol (1j) was prepared as red solid according to the General Procedure B (purification by flash column chromatography: 20→30% EtOAc in hexanes, 85% yield).

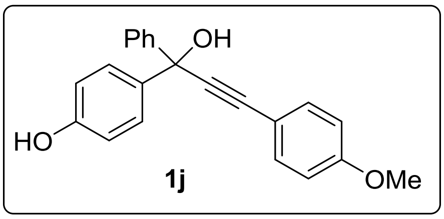

^{1}H NMR (400 MHz, acetone-*d*$_6$) δ 8.33 (br s, 1H), 7.69 (d, *J* = 7.2 Hz, 2H), 7.51 (d, *J* = 8.4 Hz, 2H), 7.45 (d, *J* = 8.8 Hz, 2H), 7.32 (t, *J* = 7.2 Hz, 2H), 7.23 (t, *J* = 7.2 Hz, 1H), 6.94 (d, *J* = 7.2 Hz, 2H), 6.80 (d, *J* = 8.8 Hz, 2H), 5.51 (br s, 1H), 3.81 (s, 3H) ppm.

^{13}C NMR (100 MHz, acetone-*d*$_6$) δ 160.6, 157.2, 147.6, 138.3, 133.5, 128.4, 128.0, 127.5, 126.6, 115.5, 115.2, 114.7, 92.4, 86.2, 74.2, 55.4 ppm.

IR (thin film) 3283, 2976, 1606, 1510, 1448, 1249, 1170, 1043, 833, 699 cm^{-1}.

HRMS (CI+) Calcd for $C_{22}H_{18}O_3$ [M$^+$]: 330.1256, Found: 330.1255.

4-(3-(4-Chlorophenyl)-1-hydroxy-1-phenylprop-2-yn-1-yl)phenol (1k) was prepared as white solid according to the General Procedure B (purification by flash column chromatography: 20→30% EtOAc in hexanes, 95% yield).

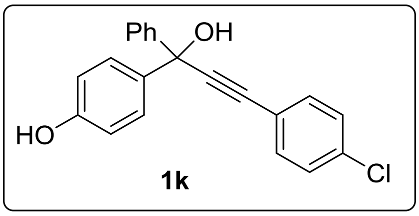

^{1}H NMR (400 MHz, acetone-*d*$_6$) δ 8.29 (br s, 1H), 7.68 (d, *J* = 8.0 Hz, 2H), 7.51 (t, *J* = 8.4 Hz, 4H), 7.43 (d, *J* = 8.0 Hz, 2H), 7.33 (t, *J* = 7.6 Hz, 2H), 7.24 (d, *J* = 7.6 Hz, 1H), 6.80 (d, *J* = 7.6 Hz, 2H), 5.58 (br s, 1H) ppm.

^{13}**C NMR** (100 MHz, acetone-d_6) δ 157.6, 147.5, 138.1, 134.9, 133.9, 129.7, 128.8, 128.3, 128.0, 126.7, 122.7, 115.6, 95.4, 85.3, 74.6 ppm.

IR (thin film) 3289, 1511, 1490, 1170, 982, 830, 699 cm^{-1}.

HRMS (CI+) Calcd for $C_{21}H_{15}{}^{35}ClO_2$ [M$^+$]: 334.0761, Found: 334.0762.

1-(4-Methoxyphenyl)-1,3-diphenylprop-2-yn-1-ol (1i') was prepared as yellow solid in 92% yield according to the literature procedure [2] (purification by flash column chromatography: 10→20% EtOAc in hexanes).

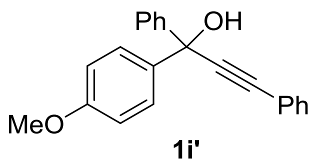

1i'

^{1}**H NMR** (400 MHz, acetone-d_6) δ 7.71 (d, J = 7.6 Hz, 2H), 7.62 (d, J = 8.8, 2H), 7.54–7.50 (m, 2H), 7.41-7.31 (m, 5H), 7.25 (t, J = 7.6 Hz, 1H), 6.90 (d, J = 8.8 Hz, 2H), 5.65 (s, 1H), 3.77 (s, 3H) ppm.

^{13}**C NMR** (100 MHz, acetone-d_6) δ 159.7, 147.3, 139.2, 132.1, 129.2, 128.6 (2C), 128.0, 127.7, 126.6, 123.6, 113.9, 93.9, 86.4, 74.3, 55.3 ppm.

4-(3-(Furan-2-yl)-1-hydroxy-1-phenylprop-2-yn-1-yl)phenol (1l) was prepared as pale yellow solid according to the General Procedure B (purification by flash column chromatography: 20→30% EtOAc in hexanes, 75% yield).

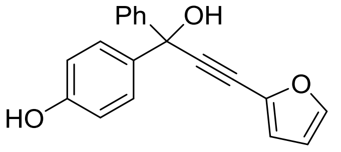

1l

^{1}**H NMR** (400 MHz, acetone-d_6) δ 8.38 (br s, 1H), 7.65 (d, J = 7.2 Hz, 2H), 7.61-7.59 (m, 1H), 7.47 (d, J = 8.8 Hz, 2H), 7.34 (t, J = 7.6 Hz, 2H), 7.25 (t, J = 7.2 Hz, 1H), 6.81 (d, J = 8.8 Hz, 2H), 6.74-6.72 (m, 1H), 6.52-6.49 (m, 1H), 5.72 (br s, 1H) ppm.

^{13}**C NMR** (100 MHz, acetone-d_6) δ 157.3, 146.7, 144.7, 137.3, 137.1, 128.5, 127.9, 127.7, 126.5, 115.9, 115.3, 111.6, 97.7, 76.6, 74.3 ppm.

IR (thin film) 3330, 1613, 1511, 1448, 1213, 1171, 1050, 834, 698 cm^{-1}.

HRMS (CI+) Calcd for $C_{19}H_{14}O_3$ [M$^+$]: 290.0943, Found: 290.0952.

4-(1-Hydroxy-1-phenyl-3-(thiophen-3-yl)prop-2-yn-1-yl)phenol (1m) was prepared as pale yellow solid according to the General Procedure B (purification by flash column chromatography: 20→ 30% EtOAc in hexanes, 83% yield).

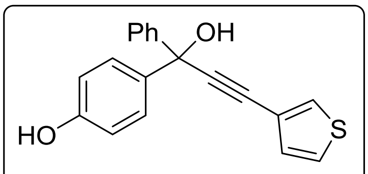

^{1}H NMR (400 MHz, acetone-d_6) δ 8.32 (br s, 1H), 7.71-7.65 (m, 3H), 7.53-7.47 (m, 3H), 7.32 (t, J = 7.6 Hz, 2H), 7.25-7.18 (m, 2H), 6.79 (d, J = 8.4 Hz, 2H), 5.56 (br s, 1H) ppm.

^{13}C NMR (100 MHz, acetone-d_6) δ 157.3, 147.4, 138.1, 130.3, 129.6, 128.5, 128.1, 127.6, 126.7, 126.6, 122.6, 115.2, 93.3, 81.6, 74.3 ppm.

IR (thin film) 3379, 2884, 1613, 1511, 1358, 1167, 1099, 784, 700 cm^{-1}.

HRMS (CI+) Calcd for $C_{19}H_{14}O_2S$ [M$^+$]: 306.0715, Found: 306.0718.

4-(3-(4-Fluorophenyl)-1-hydroxy-1-phenylprop-2-yn-1-yl)phenol (1n) was prepared as white solid according to the General Procedure B (purification by flash column chromatography: 20→ 30% EtOAc in hexanes, 82% yield).

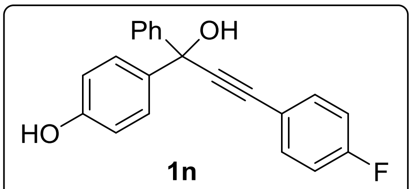

^{1}H NMR (400 MHz, acetone-d_6) δ 8.35 (br s, 1H), 7.69 (d, J = 7.2 Hz, 2H), 7.59-7.50 (m, 4H), 7.33 (t, J = 7.2 Hz, 2H), 7.23 (t, J = 7.2 Hz, 1H), 7.17 (t, J = 8.8 Hz, 2H), 6.81 (d, J = 8.8 Hz, 2H), 5.60 (br s, 1H) ppm.

^{13}C NMR (100 MHz, acetone-d_6) δ 163.1 (d, $J_{C\text{-}F}$ = 246 Hz), 157.2, 147.2, 137.9, 134.1 (d, $J_{C\text{-}F}$ = 8.5 Hz), 128.4, 127.9, 127.5, 126.5, 119.8 (d, $J_{C\text{-}F}$ = 3.5 Hz), 116.2 (d, $J_{C\text{-}F}$ = 22.3 Hz), 115.2, 93.7, 85.0, 74.2 ppm.

^{19}F NMR (376 MHz, acetone-d_6) δ -112.4 ppm.

IR (thin film) 3288, 2978, 1600, 1509, 1449, 1230, 1170, 987, 835, 699 cm^{-1}.

HRMS (CI+) Calcd for $C_{21}H_{15}FO_2$ [M$^+$]: 318.1056, Found: 318.1058.

4-(3-(3,5-Dimethylphenyl)-1-hydroxy-1-phenylprop-2-yn-1-yl)phenol (1o) was prepared as pale yellow solid according to the General Procedure B (purification by flash column chromatography: 20→30% EtOAc in hexanes, 87% yield).

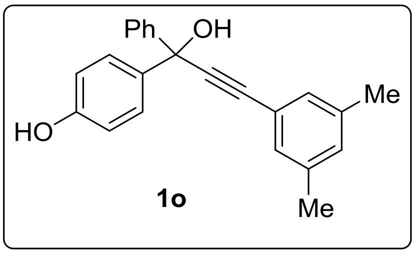

^{1}H NMR (400 MHz, acetone-d_6) δ 8.32 (br s, 1H), 7.68 (d, J = 7.2 Hz, 2H), 7.50 (d, J = 8.8 Hz, 2H), 7.33 (t, J = 7.6 Hz, 2H), 7.23 (t, J = 7.6 Hz, 1H), 7.12 (s, 2H), 7.02 (s, 1H), 6.80 (d, J = 8.4 Hz, 2H), 5.54 (br s, 1H), 2.28 (s, 6H) ppm.

^{13}C NMR (100 MHz, acetone-d_6) δ 157.3, 147.6, 138.7, 138.2, 130.8, 129.7, 128.5, 128.1, 127.6, 126.6, 123.4, 115.3, 93.3, 86.6, 74.3, 20.8 ppm.

IR (thin film) 3285, 2977, 1598, 1511, 1449, 1167, 982, 833, 699 cm^{-1}.

HRMS (CI+) Calcd for $C_{23}H_{20}O_2$ [M^+]: 328.1463, Found: 328.1470.

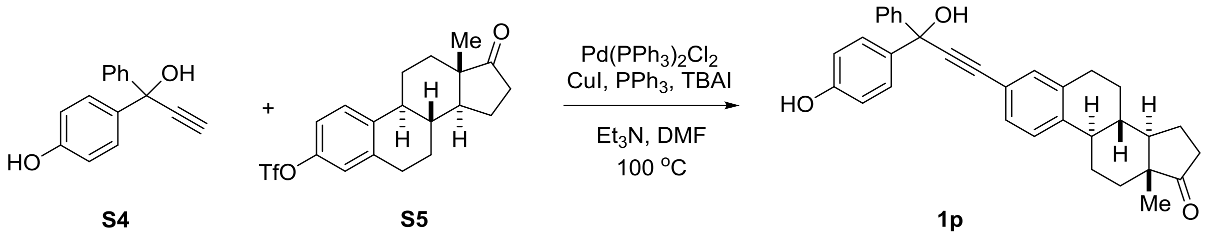

(8*R*,9*S*,13*S*,14*S*)-3-(3-Hydroxy-3-(4-hydroxyphenyl)-3-phenylprop-1-yn-1-yl)-13-methyl-6,7,8,9,11,12,13,14,15,16-decahydro-17*H*-cyclopenta[a]phenanthren-17-one (1p) was synthesized according to the literature procedure. [3] An oven-dried Schlenk tube was charged with triflate **S5**[4] (2.6 g, 6.5 mmol, 1.3 equiv), $Pd(PPh_3)_2Cl_2$ (351.6 mg, 0.5 mmol, 0.1 equiv), PPh_3 (164.9 mg, 0.65 mmol, 0.13 equiv), and CuI (49.4 mg, 0.25 mmol, 0.05 equiv). Then anhydrous DMF (5 mL) and anhydrous Et_3N (5 mL) were added under N_2. After degassing, the alkyne **S4** (1.1g, 5.0 mmol) was added, and the reaction mixture was stirred at 95 °C for 24 h. Then, the reaction mixture was cooled to room temperature, diluted with Et_2O, and washed with brine. The layers were separated and the organic layer was dried over Na_2SO_4, filtered, and concentrated.

The crude product was purified by flash column chromatography (10→30% EtOAc in hexanes) to afford the pure alkyne **1p** as a red solid (0.92 g, 39% yield).

^{1}H NMR (400 MHz, acetone-*d₆*) δ 8.37 (br s, 1H), 7.72 (d, J = 7.2 Hz, 2H), 7.55 (d, J = 8.8 Hz, 2H), 7.39-7.33 (m, 3H), 7.31-7.25 (m, 3H), 6.83 (d, J = 8.8 Hz, 1H), 5.57 (br s, 1H), 2.94-2.90 (m, 2H), 2.51-2.41 (m, 2H), 2.35-2.30 (m, 1H), 2.15-1.98 (m, 4H), 1.92-1.87 (m, 1H), 1.72-1.38 (m, 6H), 0.92 (s, 3H) ppm.

^{13}C NMR (100 MHz, acetone-d_6) δ 219.2, 157.3, 147.6, 141.4, 138.2, 137.6, 132.5, 129.3, 128.5, 128.1, 127.6, 126.6, 126.2, 120.8, 115.3, 93.2, 86.5, 74.3, 50.9, 48.1, 45.0, 38.6, 35.8, 32.3, 29.5, 26.8, 26.1, 21.9, 13.9 ppm.

IR (thin film) 3396, 2932, 1724, 1614, 1511, 1264, 1056, 833, 699 cm^{-1}.

HRMS (CI+) Calcd for $C_{33}H_{32}O_3$ [M$^+$]: 476.2351, Found: 476.2334.

4-(3-(Cyclohex-1-en-1-yl)-1-hydroxy-1-phenylprop-2-yn-1-yl)phenol (1q) was prepared as pale yellow solid according to the General Procedure B (purification by flash column chromatography: 20→30% EtOAc in hexanes) in 93% yield.

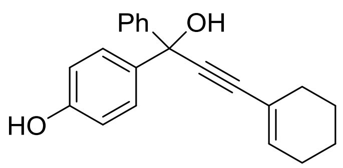

1q

^{1}H NMR (400 MHz, acetone-d_6) δ 8.29 (br s, 1H), 7.61 (d, J = 7.2 Hz, 2H), 7.43 (d, J = 8.8 Hz, 2H), 7.29 (t, J = 7.2 Hz, 2H), 7.20 (t, J = 7.2 Hz, 1H), 6.76 (d, J = 7.6 Hz, 2H), 6.12-6.09 (m, 1H), 5.36 (br s, 1H), 2.17-2.07 (m, 4H), 1.66-1.56 (m, 4H) ppm.

^{13}C NMR (100 MHz, acetone-d_6) δ 157.1, 147.8, 138.4, 134.9, 128.4, 128.0, 127.4, 126.6, 121.1, 115.1, 91.3, 88.1, 74.1, 25.9, 22.7, 21.9 ppm.

IR (thin film) 3396, 2931, 1614, 1511, 1448, 1227, 1166, 833, 699 cm^{-1}.

HRMS (CI+) Calcd for $C_{21}H_{20}O_2$ [M$^+$]: 304.1463, Found: 304.1477.

4-(1-Hydroxy-4-methyl-1-phenylpent-4-en-2-yn-1-yl)phenol (1r) was prepared as pale yellow solid according to the General Procedure B (purification by flash column chromatography: 20→30% EtOAc in hexanes) in 86% yield.

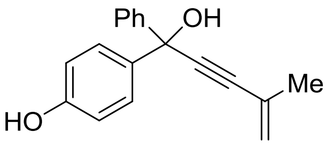

1r

^{1}H NMR (400 MHz, acetone-*d*$_6$) δ 8.38 (br s, 1H), 7.61 (d, *J* = 8.8 Hz, 2H), 7.43 (d, *J* = 8.4 Hz, 2H), 7.31 (t, *J* = 7.6 Hz, 2H), 7.21 (t, *J* = 7.6 Hz, 1H), 6.77 (d, *J* = 8.8 Hz, 2H), 5.49 (br s, 1H), 5.32-5.28 (m, 2H), 1.92 (t, *J* = 1.6 Hz, 3H) ppm.

^{13}C NMR (100 MHz, acetone-*d*$_6$) δ 157.1, 147.4, 138.0, 128.3, 127.9, 127.4, 127.3, 126.4, 121.8, 115.1, 93.0, 87.2, 74.0, 23.2 ppm.

IR (thin film) 3384, 1613, 1511, 1448, 1237, 1172, 996, 837, 699 cm^{-1}.

HRMS (CI+) Calcd for $C_{18}H_{16}O_2$ [M$^+$]: 264.1150, Found: 264.1153.

4-(1-Hydroxy-1-phenyl-3-(trimethylsilyl)prop-2-yn-1-yl)phenol (1s) was prepared as white solid according to the General Procedure B (purification by flash column chromatography: 20→30% EtOAc in hexanes) in 92% yield.

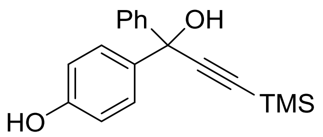

1s

^{1}H NMR (400 MHz, acetone-*d*$_6$) δ 8.32 (br s, 1H), 7.62 (d, *J* = 7.2 Hz, 2H), 7.44 (d, *J* = 8.8 Hz, 2H), 7.30 (t, *J* = 7.2 Hz, 2H), 7.21 (t, *J* = 7.2 Hz, 1H), 6.77 (d, *J* = 8.8 Hz, 2H), 5.47 (br s, 1H), 0.20 (s, 9H) ppm.

^{13}C NMR (100 MHz, acetone-*d*$_6$) δ 155.2, 147.2, 137.9, 128.4, 127.9, 127.6, 126.5, 115.2, 110.4, 90.2, 74.1, -0.3 ppm.

IR (thin film) 3355, 2974, 2168, 1613, 1512, 1449, 1251, 1059, 842, 766 cm^{-1}.

HRMS (CI+) Calcd for $C_{18}H_{20}O_2Si$ [M$^+$]: 296.1233, Found: 296.1237.

4-(1-Hydroxy-1-phenyl-3-(triethylsilyl)prop-2-yn-1-yl)phenol (1t) was prepared as white solid according to the General Procedure B (purification by flash column chromatography: 20→30% EtOAc in hexanes) in 50% yield.

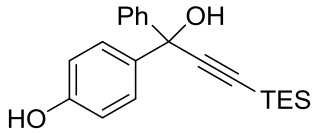

1t

^{1}H NMR (400 MHz, CDCl$_3$) δ 7.62 (d, *J* = 8.4 Hz, 2H), 7.47 (d, *J* = 8.8 Hz, 2H), 7.33 (t, *J* = 7.2 Hz, 2H), 7.27 (t, *J* = 7.2 Hz, 1H), 6.76 (d, *J* = 8.8 Hz, 2H), 5.40 (br s, 1H), 1.04 (t, *J* = 8.0 Hz, 9H), 0.69 (q, *J* = 8.0 Hz, 6H) ppm.

^{13}**C NMR** (100 MHz, $CDCl_2$) δ 155.7, 145.6, 137.8, 128.6, 127.94, 127.86, 126.2, 115.3, 109.7, 89.8, 74.8, 7.7, 4.7 ppm.

IR (thin film) 3321, 2956, 1598, 1511, 1450, 1172, 1004, 835, 728 cm^{-1}.

HRMS (CI+) Calcd for $C_{21}H_{26}O_2Si$ [M$^+$]: 338.1702, Found: 338.1714.

4-(1-Hydroxy-1-phenylhept-2-yn-1-yl)phenol (1u) was prepared as white solid according to the General Procedure B (purification by flash column chromatography: 20→30% EtOAc in hexanes) in 89% yield.

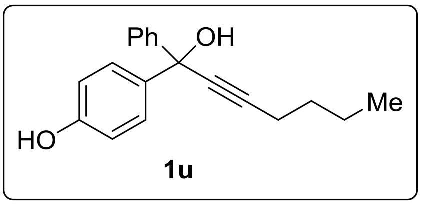

^{1}**H NMR** (400 MHz, acetone-d_6) δ 8.32 (br s, 1H), 7.60 (d, J = 7.2 Hz, 2H), 7.43 (d, J = 8.8 Hz, 2H), 7.28 (t, J = 7.2 Hz, 2H), 7.18 (t, J = 7.2 Hz, 1H), 6.75 (d, J = 8.8 Hz, 2H), 5.26 (br s, 1H), 2.32 (t, J = 7.2 Hz, 2H), 2.06-1.41 (m, 4H), 0.92 (t, J = 7.2 Hz, 3H) ppm.

^{13}**C NMR** (100 MHz, acetone-d_6) δ 157.0, 148.0, 138.6, 128.2, 127.9, 127.2, 126.5, 114.9, 86.6, 84.9, 73.8, 31.3, 22.2, 18.6, 13.5 ppm.

IR (thin film) 3385, 2958, 1614, 1511, 1449, 1171, 1001, 829, 699 cm^{-1}.

HRMS (CI+) Calcd for $C_{19}H_{20}O_2$ [M$^+$]: 280.1463, Found: 280.1466.

4-(1-Hydroxy-1,5-diphenylpent-2-yn-1-yl)phenol (1v) was prepared as brown oil according to the General Procedure B (purification by flash column chromatography: 20→30% EtOAc in hexanes, 67% yield).

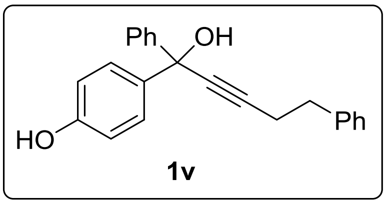

^{1}**H NMR** (400 MHz, acetone-d_6) δ 8.29 (br s, 1H), 7.52 (d, J = 8.4 Hz, 2H), 7.35-7.15 (m, 10H), 6.72 (d, J = 8.8 Hz, 2H), 5.21 (br s, 1H), 2.87 (t, J = 7.2 Hz, 2H), 2.63 (t, J = 7.2 Hz, 2H) ppm.

^{13}**C NMR** (100 MHz, acetone-d_6) δ 157.0, 147.9, 141.5, 138.6, 129.3, 128.9, 128.3, 128.0, 127.3, 126.8, 126.6, 115.0, 86.2, 85.6, 73.8, 35.3, 21.2 ppm.

IR (thin film) 3340, 2977, 1611, 1511, 1449, 1171, 1018, 832, 699 cm^{-1}.

HRMS (CI+) Calcd for $C_{23}H_{20}O_2$ [M$^+$]: 328.1463, Found: 328.1452.

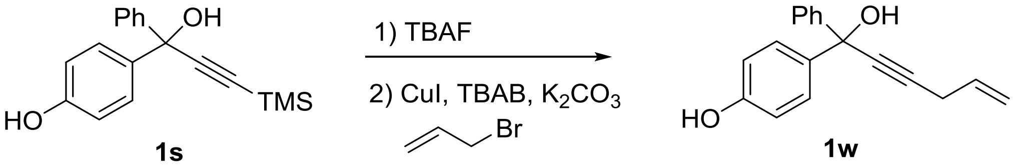

4-(1-Hydroxy-1-phenylhex-5-en-2-yn-1-yl)phenol (1w) was prepared from alcohol **1s** according to the literature procedure.[5] At room temperature, to a stirred solution of alcohol **1s** (3.0 g, 10.0 mmol) in THF (20 mL) was added a solution of tetrabutylammonium fluoride (3.1 g, 12.0 mmol, 1.2 equiv) in THF (12 mL) dropwise. The mixture was stirred for 1 h and then concentrated to evaporate most of the THF solvent. The residue was taken up in diethyl ether and water (1:1, 20 ml). The resulting layers were separated and the aqueous layer was extracted with diethyl ether (3 × 20 mL). The combined organic layers were dried, filtered, and concentrated. The residue was purified by silica gel column chromatography (20→30% EtOAc in hexanes) to give the terminal alkyne (1.95 g, 87% yield) as pale brown solid. Under N_2, to a stirred solution of the above terminal alkyne (1.12 g, 5.0 mmol) in dry DMF (5 mL) were sequentially added K_2CO_3 (0.97 g, 7.0 mmol), tetrabutylammonium bromide (241 mg, 0.75 mmol), and CuI (48 mg, 0.25 mmol) at room temperature. Allyl bromide (0.65 mL, 7.5 mmol) was added 15 min later. Next, the reaction mixture was stirred for 24 h before it was poured into water and extracted with ether (20 mL×3). The combined organic layers were washed with brine, dried over $MgSO_4$, filtered, and concentration. The residue was purified by silica gel flash column chromatography (10→20% EtOAc in hexanes) to give the pure **1w** (1.13 g, 66% yield) as pale yellow solid.

^{1}H NMR (400 MHz, acetone-*d*$_6$) δ 8.30 (br s, 1H), 7.61 (d, *J* = 8.0 Hz, 2H), 7.44 (d, *J* = 8.0 Hz, 2H), 7.29 (t, *J* = 7.6 Hz, 2H), 7.20 (t, *J* = 7.6 Hz, 1H), 6.76 (d, *J* = 8.8 Hz, 2H), 5.94-5.83 (m, 1H), 5.41-5.30 (m, 1H), 5.36 (br s, 1H), 5.12-5.07 (m, 1H), 3.14-2.98 (m, 2H) ppm.

^{13}C NMR (100 MHz, acetone-*d*$_6$) δ 157.1, 147.8, 138.5, 133.5, 128.3, 128.0, 127.4, 126.5, 116.0, 115.1, 87.3, 83.3, 73.9, 23.2 ppm.

IR (thin film) 3380, 1612, 1511, 1448, 1233, 1172, 994, 830, 700 cm^{-1}.

HRMS (CI+) Calcd for $C_{18}H_{16}O_2$ [M^+]: 264.1150, Found: 264.1140.

4-(1-Hydroxy-1-phenylundeca-2,10-diyn-1-yl)phenol (1x) was prepared as brown oil according to the General Procedure B (purification by flash column chromatography: 20→30% EtOAc in hexanes, 78% yield).

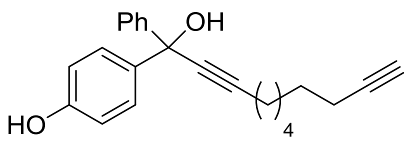

1x

^{1}H NMR (400 MHz, CD_2Cl_2) δ 7.57 (d, *J* = 7.2 Hz, 2H), 7.44 (d, *J* = 8.8 Hz, 2H), 7.31 (t, *J* = 7.2 Hz, 2H), 7.24 (t, *J* = 7.2 Hz, 1H), 6.76 (t, *J* = 8.8 Hz, 2H), 5.24 (br s, 1H), 2.83 (br s, 1H), 2.35 (t, *J* = 7.2 Hz, 2H), 2.21-2.15 (m, 2H), 1.98-1.96 (m, 1H), 1.65-1.38 (m, 8H) ppm.

^{13}C NMR (100 MHz, CD_2Cl_2) δ 155.6, 146.3, 138.4, 128.5, 127.9, 127.8, 126.2, 115.2, 88.4, 85.0, 83.6, 74.5, 68.4, 28.81, 28.77, 28.7, 28.6, 19.1, 18.6 ppm.

IR (thin film) 3384, 3296, 2935, 1611, 1511, 1447, 1231, 1171, 831, 699 cm^{-1}.

HRMS (CI+) Calcd for $C_{23}H_{24}O_2$ [M^+]: 332.1776, Found: 332.1774.

4-(5-((*tert*-Butyldimethylsilyl)oxy)-1-hydroxy-1-phenylpent-2-yn-1-yl)phenol (1y) was prepared as yellow oil according to the General Procedure B (purification by flash column chromatography: 20→30% EtOAc in hexanes, 75% yield).

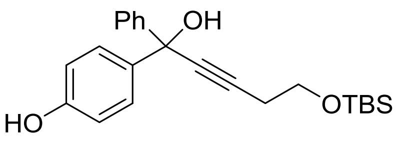

1y

^{1}H NMR (400 MHz, CD_2Cl_2) δ 7.57 (d, *J* = 8.4 Hz, 2H), 7.44 (d, *J* = 8.8 Hz, 2H), 7.31 (t, *J* = 7.6 Hz, 2H), 7.24 (t, *J* = 7.2 Hz, 1H), 6.75 (t, *J* = 8.8 Hz, 2H), 3.80 (t, *J* = 6.8 Hz, 2H), 2.55 (t, *J* = 6.8 Hz, 2H), 0.91 (s, 9H), 0.08 (s, 6H) ppm.

^{13}C NMR (100 MHz, CD_2Cl_2) δ 155.6, 146.2, 138.3, 128.5, 127.9, 127.8, 126.2, 115.2, 85.5, 84.4, 74.4, 62.2, 26.1, 23.6, 18.6, –5.2 ppm.

IR (thin film) 3363, 2929, 1613, 1511, 1448, 1254, 1105, 834, 698 cm^{-1}.

HRMS (CI+) Calcd for $C_{23}H_{30}O_3Si$ [M^+]: 382.1964, Found: 382.1962.

4-(6-Chloro-1-hydroxy-1-phenylhex-2-yn-1-yl)phenol (1z) was prepared as pale yellow solid according to the General Procedure A (purification by flash column chromatography: 20→30% EtOAc in hexanes, 98% yield).

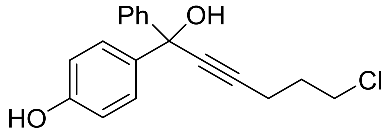

1z

^{1}H NMR (400 MHz, acetone-d_6) δ 8.28 (br s, 1H), 7.60 (d, J = 7.2 Hz, 2H), 7.43 (d, J = 8.8 Hz, 2H), 7.29 (t, J = 7.2 Hz, 2H), 7.20 (t, J = 7.2 Hz, 1H), 6.76 (d, J = 8.8 Hz, 2H), 5.35 (br s, 1H), 3.74 (t, J = 6.4 Hz, 2H), 2.52 (t, J = 6.8 Hz, 2H), 2.00 (q, J = 6.4 Hz, 2H) ppm.

^{13}C NMR (100 MHz, acetone-d_6) δ 157.0, 147.8, 138.4, 128.3, 127.9, 127.3, 126.4, 115.0, 85.8, 84.9, 73.8, 44.2, 32.0, 16.3 ppm.

IR (thin film) 3388, 2961, 1613, 1511, 1448, 1234, 1172, 1016, 831, 700 cm^{-1}.

HRMS (CI+) Calcd for $C_{18}H_{17}{}^{35}ClO_2$ [M$^+$]: 300.0917, Found: 300.0913.

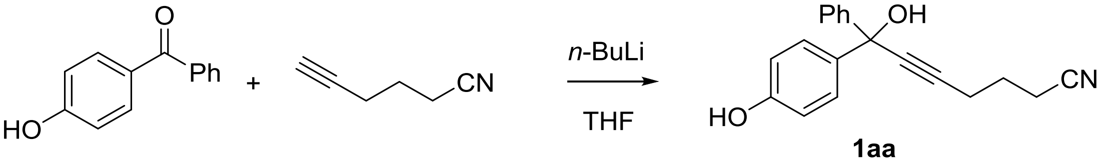

1aa

7-Hydroxy-7-(4-hydroxyphenyl)-7-phenylhept-5-ynenitrile (1aa). At –78 °C under N_2, to an oven-dried flask charged with a solution of hex-5-ynenitrile (1.3 mL, 12.5 mmol) in dry THF (50 mL) was added ^{n}BuLi (12.5 mmol, 5.2 mL, 2.4 M in hexane) dropwise. The reaction was stirred for 1 h at the same temperature and then a solution of the ketone (1.0 g, 5.0 mmol) in THF (3 mL) was added via syringe. The reaction mixture was then warmed to 60 °C and stirred overnight. Upon completion (~12 h), the reaction mixture was cooled to 0 °C. A saturated aqueous NH_4Cl solution (20 mL) was added dropwise. The organic layer was separated. The aqueous layer was extracted with Et_2O (3×20 mL). The combined organic layers were dried over anhydrous Na_2SO_4, filtered, and concentrated. The residue was purified by silica gel chromatography to afford the pure alcohol **1aa** as brown oil (20→30% EtOAc in hexanes, 0.87 g, 60% yield).

^{1}H NMR (400 MHz, acetone-*d*$_6$) δ 8.28 (br s, 1H), 7.60 (d, J = 7.2 Hz, 2H), 7.42 (d, J = 8.8 Hz, 2H), 7.29 (t, J = 7.6 Hz, 2H), 7.20 (t, J = 7.2 Hz, 1H), 6.76 (d, J = 8.8 Hz, 2H), 5.36 (br s, 1H), 2.61 (t, J = 7.2 Hz, 2H), 2.51 (t, J = 6.8 Hz, 2H), 1.97-1.87 (m, 2H) ppm.

^{13}C NMR (100 MHz, acetone-*d*$_6$) δ 157.2, 147.8, 138.5, 128.4, 128.0, 127.4, 126.5, 120.0, 115.2, 86.5, 84.6, 73.9, 25.4, 18.2, 16.1 ppm.

HRMS (CI+) Calcd for $C_{19}H_{18}NO_2$ [M+H$^+$]: 292.1338, Found: 292.1335.

IR (thin film) 3385, 2977, 2250, 1609, 1512, 1446, 1372, 1230, 835, 701 cm^{-1}.

4-(3-Cyclopropyl-1-hydroxy-1-phenylprop-2-yn-1-yl)phenol (1ab) was prepared as white solid according to the General Procedure B (purification by flash column chromatography: 20→30% EtOAc in hexanes, 88% yield).

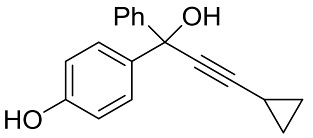

1ab

^{1}H NMR (400 MHz, acetone-*d*$_6$) δ 8.29 (br s, 1H), 7.58 (d, J = 7.2 Hz, 2H), 7.40 (d, J = 8.4 Hz, 2H), 7.27 (t, J = 7.2 Hz, 2H), 7.18 (t, J = 7.2 Hz, 1H), 6.75 (d, J = 8.8 Hz, 2H), 5.21 (br s, 1H), 1.42-1.35 (m, 1H), 0.85-0.75 (m, 2H), 0.58-0.68 (m, 2H) ppm.

^{13}C NMR (100 MHz, acetone-*d*$_6$) δ 156.9, 147.9, 138.5, 128.2, 127.8, 127.2, 126.4, 115.0, 89.8, 79.8, 73.7, 8.2, -0.3 ppm.

IR (thin film) 3355, 2236, 1613, 1511, 1448, 1235, 1171, 994, 836, 699 cm^{-1}.

HRMS (CI+) Calcd for $C_{18}H_{16}O_2$ [M$^+$]: 264.1150, Found: 264.1150.

4-(1-Hydroxy-4-methyl-1-phenylpent-2-yn-1-yl)phenol (1ac) was prepared as white solid according to the General Procedure B (purification by flash column chromatography: 20→30% EtOAc in hexanes, 96% yield).

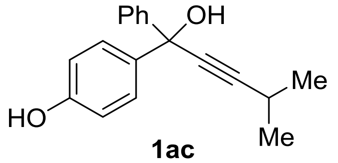

1ac

^{1}H NMR (400 MHz, acetone-*d*$_6$) δ 8.25 (br s, 1H), 7.62 (d, J = 7.2 Hz, 2H), 7.44 (d, J = 8.8 Hz, 2H), 7.28 (t, J = 7.6 Hz, 2H), 7.19 (t, J = 7.6 Hz, 1H), 6.76 (d, J = 8.8 Hz, 2H), 5.23 (br s, 1H), 2.76-2.64 (m, 1H), 1.21 (s, 3H), 1.20 (s, 3H) ppm.

^{13}C NMR (100 MHz, acetone-*d*$_6$) δ 157.0, 148.0, 138.7, 128.2, 127.9, 127.2, 126.5, 115.0, 92.0, 84.2, 73.7, 23.0, 21.0 ppm.

IR (thin film) 3388, 2971, 1613, 1511, 1448, 1235, 1169, 994, 835, 699 cm^{-1}.

HRMS (CI+) Calcd for $C_{18}H_{18}O_2$ [M$^+$]: 266.1307, Found: 266.1308.

4-(1-Hydroxy-4,4-dimethyl-1-phenylpent-2-yn-1-yl)phenol (1ad) was prepared as white solid according to the General Procedure B (purification by flash column chromatography: 20→30% EtOAc in hexanes, 86% yield).

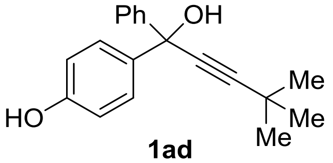

1ad

^{1}H NMR (400 MHz, acetone-*d*$_6$) δ 8.25 (br s, 1H), 7.62 (d, *J* = 8.8 Hz, 2H), 7.45 (d, *J* = 8.4 Hz, 2H), 7.28 (t, *J* = 7.2 Hz, 2H), 7.18 (t, *J* = 7.2 Hz, 1H), 6.75 (d, *J* = 8.8 Hz, 2H), 5.23 (br s, 1H), 1.28 (s, 9H) ppm.

^{13}C NMR (100 MHz, acetone-*d*$_6$) δ 157.0, 148.2, 138.9, 128.3, 127.9, 127.3, 126.5, 115.1, 94.8, 83.7, 73.7, 31.0, 27.9 ppm.

IR (thin film) 3407, 2970, 1614, 1511, 1449, 1264, 1171, 1002, 832, 698 cm^{-1}.

HRMS (CI+) Calcd for $C_{19}H_{20}O_2$ [M$^+$]: 280.1463, Found: 280.1458.

4-(1-Hydroxy-3-phenyl-1-(4-(prop-2-yn-1-yloxy)phenyl)prop-2-yn-1-yl)phenol (1ae) was prepared as brown oil according to the General Procedure B (purification by flash column chromatography: 20→30% EtOAc in hexanes, 75% yield).

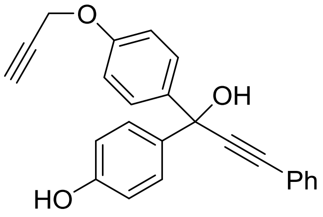

1ae

^{1}H NMR (400 MHz, acetone-*d*$_6$) δ 8.36 (br s, 1H), 7.61 (d, *J* = 8.8 Hz, 2H), 7.51 (d, *J* = 8.8 Hz, 4H), 7.40-7.37 (m, 3H), 6.97 (d, *J* = 8.8 Hz, 2H), 6.81 (d, *J* = 8.8 Hz, 2H), 5.54 (br s, 1H), 4.77 (d, *J* = 2.4 Hz, 2H), 3.06 (t, *J* = 2.4 Hz, 1H) ppm.

^{13}C NMR (100 MHz, acetone-*d*$_6$) δ 157.4, 157.1, 140.3, 138.1, 131.9, 129.03, 128.97, 127.9, 127.8, 123.5, 115.1, 114.7, 94.0, 86.0, 79.5, 76.6, 73.8, 55.9 ppm.

IR (thin film) 3391, 3290, 1597, 1508, 1225, 1170, 1026, 836, 691 cm^{-1}.

HRMS (CI+) Calcd for $C_{24}H_{18}O_3$ [M$^+$]: 354.1256, Found: 354.1245.

4-(1-(3,5-Dimethoxyphenyl)-1-hydroxy-3-phenylprop-2-yn-1-yl)phenol (1af) was prepared as pale yellow solid according to the General Procedure B (purification by flash column chromatography: 20→30% EtOAc in hexanes, 85% yield).

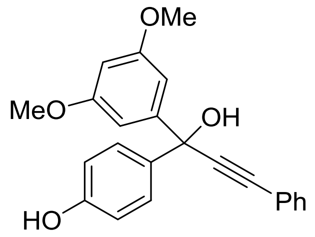

1af

^{1}H NMR (400 MHz, acetone-d_6) δ 8.34 (br s, 1H), 7.55-7.49 (m, 4H), 7.40-7.37 (m, 3H), 6.88-6.86 (m, 2H), 6.80 (d, J = 8.8 Hz, 2H), 6.39-6.37 (m, 1H), 5.58 (br s, 1H), 3.76 (s, 6H) ppm.

^{13}C NMR (100 MHz, acetone-d_6) δ 161.2, 157.3, 149.8, 137.8, 132.0, 129.13, 129.08, 127.9, 123.6, 115.2, 105.0, 99.0, 93.8, 86.0, 74.2, 55.3 ppm.

IR (thin film) 3417, 2974, 1597, 1511, 1458, 1204, 1155, 1052, 837, 759, 691 cm^{-1}.

HRMS (CI+) Calcd for $C_{23}H_{20}O_4$ [M$^+$]: 360.1362, Found: 360.1372.

4-(1-Hydroxy-1-phenylhept-2-yn-1-yl)-2-methylphenol (1ag) was prepared as pale yellow solid according to the General Procedure B (purification by flash column chromatography: 20→30% EtOAc in hexanes, 89% yield).

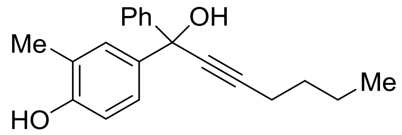

1ag

^{1}H NMR (400 MHz, acetone-d_6) δ 8.12 (br s, 1H), 7.60 (d, J = 8.8 Hz, 2H), 7.34 (s, 1H), 7.30-7.23 (m, 3H), 7.18 (t, J = 7.2 Hz, 1H), 6.72 (d, J = 8.4 Hz, 1H), 5.16 (br s, 1H), 2.32 (d, J = 6.8 Hz, 2H), 2.16 (s, 3H), 1.59-1.42 (m, 4H), 0.92 (t, J = 7.2 Hz, 3H) ppm.

^{13}C NMR (100 MHz, acetone-d_6) δ 154.9, 148.1, 138.6, 129.3, 128.2, 127.2, 126.5, 125.2, 123.8, 114.3, 86.6, 85.0, 73.8, 31.3, 22.3, 18.6, 16.1, 13.6 ppm.

IR (thin film) 3386, 2957, 1610, 1505, 1449, 1260, 1114, 1012, 819, 699 cm^{-1}.

HRMS (CI+) Calcd for $C_{20}H_{22}O_2$ [M$^+$]: 294.1620, Found: 294.1610.

General Procedure C

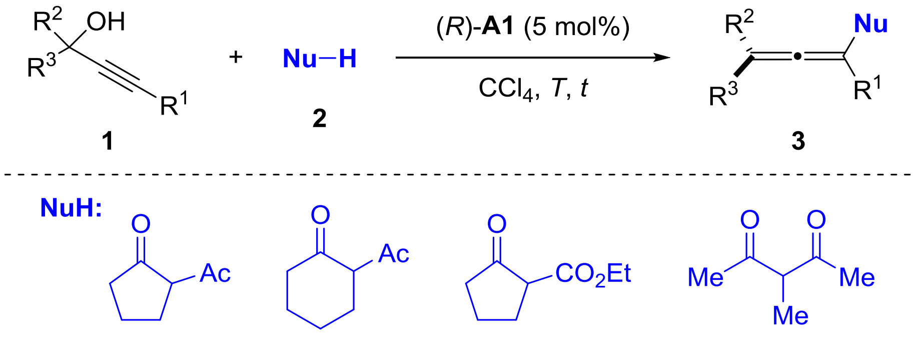

At room temperature, an oven-dried 10-mL vial was charged with a mixture of the alcohol substrate **1** (0.2 mmol), the carbon nucleophile **2** (0.3 mmol, 1.5 equiv), and CCl_4 (3.8 mL). The reaction mixture was cooled to –20 °C (for **3a-j**) or 0 °C (for **3k-p**) unless otherwise specified, and a solution of the catalyst (*R*)-**A1** (10 μmol, 5 mol%) in CCl_4 (0.2 mL) was added in one portion. The reaction mixture was stirred at the same temperature. The progress was monitored by thin layer chromatography. Upon completion, the crude mixture was directly subjected to silica gel flash chromatography to afford the pure product **3**.

Note: All the racemic products (used for HPLC reference in determining the ee value), were prepared using the same reaction with the simplest racemic phosphoric acid catalyst or TsOH.

(*R*)-2-Acetyl-2-((*R*)-1,3-bis(4-methoxyphenyl)-3-phenylpropa-1,2-dien-1-yl)cyclopentan-1-one (3a) was prepared as pale yellow oil from propargylic alcohol **1a** (0.2 mmol, 68.9 mg) and **2a** (0.3 mmol, 36.3 μL) according to General Procedure **C** (3 h, purified by flash column chromatography: 5-10%

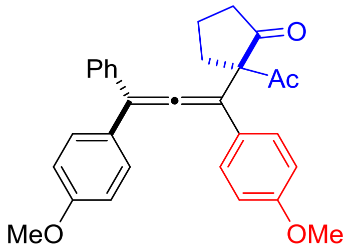

3a

EtOAc in hexanes, 80.6 mg, 89% yield, 6:1 d.r., 90% ee).

$[\alpha]_D^{25}$: +218.7 (c = 1.0, $CHCl_3$). HPLC analysis of the product: Daicel CHIRALPAK AD-H column; 10% *i*-PrOH in hexanes; 1.0 mL/min; retention times: 7.3 min (major), 9.6 min (minor).

^{1}H NMR (400 MHz, $CDCl_3$) δ 7.42-7.28 (m, 9H), 6.94 (d, J = 8.8 Hz, 2H), 6.86 (d, J = 8.8 Hz, 2H), 3.84 (s, 3H), 3.80 (s, 3H), 3.01-2.91 (m, 1H), 2.39 (s, 3H), 2.31-2.06 (m, 3H), 1.95-1.82 (m, 1H), 1.73-1.63 (m, 1H) ppm.

^{13}C NMR (100 MHz, $CDCl_3$) δ 212.6, 206.9, 206.7, 159.3, 159.1, 136.4, 129.6, 129.1, 128.5, 128.4, 127.9, 127.8, 127.7, 125.6, 114.3, 113.9, 107.4, 71.1, 55.2 (2C), 37.9, 31.3, 28.4, 18.7 ppm.

IR (thin film) 2955, 1701, 1605, 1507, 1245, 1030, 906, 832, 730 cm^{-1}.

HRMS (CI+) Calcd for $C_{30}H_{28}O_4$ [M$^+$]: 452.1988, Found: 452.1998.

(*R*)-2-Acetyl-2-((*R*)-1-(4-chlorophenyl)-3-(4-methoxyphenyl)-3-phenylpropa-1,2-dien-1-yl)cyclopentan-1-one (3b) was prepared as pale yellow foam from propargylic alcohol **1b** (0.2 mmol, 69.6 mg) **2a** (0.3 mmol, 36.3 μL) according to General Procedure **C** (3 h, purified by flash column chromatography: 5-10% EtOAc in hexanes, 81.6 mg, 90% yield, 7:1 d.r., 94% ee).

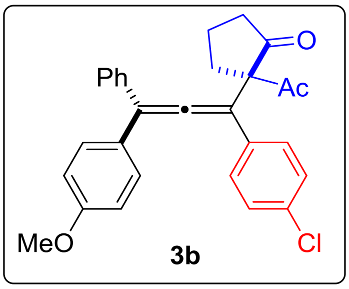

$[\alpha]_D^{25}$: +236.0 (c = 1.0, $CHCl_3$). HPLC analysis of the product: Daicel CHIRALPAK AD-H column; 5% *i*-PrOH in hexanes; 1.0 mL/min; retention times: 7.3 min (major), 9.3 min (minor).

^{1}H NMR (400 MHz, $CDCl_3$) δ 7.48-7.31 (m, 7H), 7.31-7.26 (m, 4H), 6.94 (d, J = 8.8 Hz, 2H), 3.83 (s, 3H), 3.01-2.91 (m, 1H), 2.35 (s, 3H), 2.25-2.12 (m, 3H), 1.94-1.82 (m, 1H), 1.72-1.61 (m, 1H) ppm.

^{13}C NMR (100 MHz, $CDCl_3$) δ 212.2, 207.2, 206.2, 159.5, 135.8, 133.5, 132.2, 129.5, 129.0, 128.6, 128.0, 127.9, 127.8, 127.0, 114.6, 114.0, 107.1, 70.9, 55.2, 37.9, 31.4, 28.2, 18.7 ppm.

IR (thin film) 2960, 1702, 1605, 1508, 1246, 1031, 906, 830, 697 cm^{-1}.

HRMS (CI+) Calcd for $C_{29}H_{25}{}^{35}ClO_3$ [M^+]: 456.1492, Found: 456.1482.

(*R*)-2-Acetyl-2-((*S*)-1-(furan-2-yl)-3-(4-methoxyphenyl)-3-phenylpropa-1,2-dien-1-yl)

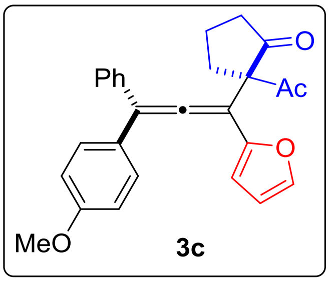

3c

cyclopentan-1-one (3c) was prepared as pale yellow foam from propargylic alcohol **1c** (0.2 mmol, 60.8 mg) **2a** (0.3 mmol, 36.3 μL) according to General Procedure **C** (3 h, purified by flash column chromatography: 5-10% EtOAc in hexanes, 75.8 mg, 92% yield, 7:1 d.r., 77% ee).

$[\alpha]_D^{25}$: +167.2 (c = 1.0, $CHCl_3$). HPLC analysis of the product: Daicel CHIRALPAK AD-H column; 3% EtOH in hexanes; 0.5 mL/min; retention times: 14.4 min (major), 18.6 min (minor).

^{1}H NMR (400 MHz, $CDCl_3$) δ 7.48-7.27 (m, 8H), 6.93 (d, J = 8.8 Hz, 2H), 6.40-6.38 (m, 1H), 6.27-6.25 (m, 1H), 3.83 (s, 3H), 2.91-2.82 (m, 1H), 2.40 (s, 3H), 2.25-2.05 (m, 3H), 1.91-1.81 (m, 1H), 1.70-1.59 (m, 1H) ppm.

^{13}C NMR (100 MHz, $CDCl_3$) δ 212.1, 205.6, 205.1, 159.5, 147.1, 142.5, 136.2, 129.7, 128.5, 128.4, 128.0, 127.6, 115.3, 114.0, 111.5, 108.3, 100.9, 70.4, 55.3, 37.9, 31.3, 27.9, 18.8 ppm.

IR (thin film) 2961, 1704, 1604, 1508, 1246, 1028, 904, 833, 696 cm^{-1}.

HRMS (CI+) Calcd for $C_{27}H_{24}O_4$ [M^+]: 412.1675, Found: 412.1674.

(*R*)-2-Acetyl-2-((*R*)-3-(4-(allyloxy)phenyl)-1-(4-chlorophenyl)-3-phenylpropa-1,2-di

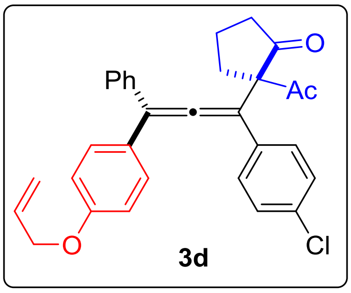

3d

en-1-yl)cyclopentan-1-one (3d) was prepared as pale yellow foam from propargylic alcohol **1d** (0.2 mmol, 74.8 mg) **2a** (0.3 mmol, 36.3 μL) according to General Procedure **C** (3 h, purified by flash column

chromatography: 5-10% EtOAc in hexanes, 90.6 mg, 94% yield, 6:1 d.r., 92% ee).

$[\alpha]_D^{25}$: +156.6 (c = 1.0, $CHCl_3$). HPLC analysis of the product: Daicel CHIRALPAK AD-H column; 3% EtOH in hexanes; 0.5 mL/min; retention times: 12.4 min (major), 20.9 min (minor).

^{1}H NMR (400 MHz, $CDCl_3$) δ 7.47-7.23 (m, 11H), 6.95 (d, J = 8.8 Hz, 2H), 6.12-6.01 (m, 1H), 5.43 (d, J = 17.2 Hz, 1H), 5.30 (d, J = 10.8 Hz, 1H), 4.57 (d, J = 5.2 Hz, 2H), 2.99-2.90 (m, 1H), 2.34 (s, 3H), 2.27-2.12 (m, 3H), 1.94-1.82 (m, 1H), 1.71-1.59 (m, 1H) ppm.

^{13}C NMR (100 MHz, $CDCl_3$) δ 212.2, 207.2, 206.3, 158.6, 135.9, 133.5, 133.1, 132.3, 129.5, 129.0, 128.6, 128.1, 127.94, 127.89, 127.2, 117.7, 114.8, 114.6, 107.1, 70.9, 68.8, 37.9, 31.5, 28.3, 18.8 ppm.

IR (thin film) 2967, 1702, 1604, 1507, 1243, 1175, 1010, 915, 830, 700 cm^{-1}.

HRMS (CI+) Calcd for $C_{31}H_{27}{}^{35}ClO_3$ [M$^+$]: 482.1649, Found: 482.1635.

(*R*)-2-Acetyl-2-((*R*)-1-(4-methoxyphenyl)-3-(4-(methylthio)phenyl)-3-phenylpropa-1,2-dien-1-yl)cyclopentan-1-one (3e) was prepared as pale yellow oil from propargylic alcohol **1e** (0.2 mmol, 72.1 mg) **2a** (0.3 mmol, 36.3 μL) according to General Procedure **C** (3 h, purified by flash column chromatography: 5-10% EtOAc in hexanes, 89.9 mg, 96% yield, 6:1 d.r., 84% ee).

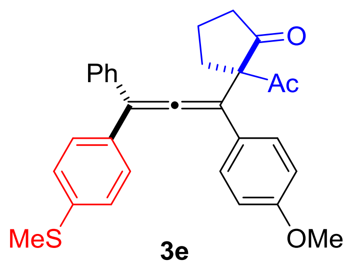

3e

$[\alpha]_D^{25}$: +213.8 (c = 1.0, $CHCl_3$). HPLC analysis of the product: Daicel CHIRALPAK AD-H column; 10% *i*-PrOH in hexanes; 1.0 mL/min; retention times: 7.7 min (major), 10.4 min (minor).

^{1}H NMR (400 MHz, $CDCl_3$) δ 7.49-7.30 (m, 7H), 7.30-7.21 (m, 4H), 6.87 (d, J = 8.8 Hz, 2H), 3.81 (s, 3H), 3.02-2.93 (m, 1H), 2.52 (s, 3H), 2.38 (s, 3H), 2.31-2.07 (m, 3H), 1.96-1.84 (m, 1H), 1.74-1.61 (m, 1H) ppm.

^{13}**C NMR** (100 MHz, $CDCl_3$) δ 212.5, 206.8, 206.6, 159.2, 138.3, 136.0, 132.1, 128.7, 128.6, 128.4, 127.9, 127.8, 126.4, 125.3, 114.3, 114.0, 107.8, 71.1, 55.2, 37.9, 31.4, 28.4, 18.8, 15.6 ppm.

IR (thin film) 2957, 1702, 1604, 1509, 1248, 1029, 906, 831, 730 cm^{-1}.

HRMS (CI+) Calcd for $C_{30}H_{28}O_3S$ [M^+]: 468.1759, Found: 468.1773.

(*R*)-2-Acetyl-2-(3,3-bis(4-methoxyphenyl)-1-phenylpropa-1,2-dien-1-yl)cyclopentan-1-one (3e) was prepared as pale yellow oil from propargylic alcohol **1f** (0.2 mmol, 68.9 mg) and **2a** (0.3 mmol, 36.3 μL) according to General Procedure **C** (2 h, purified by flash column chromatography: 5-10% EtOAc in hexanes, 88.5 mg, 98% yield, 86% ee).

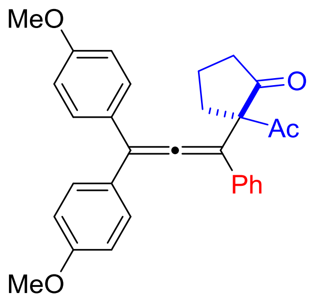

3f

$[\alpha]_D^{25}$: +179.2 (c = 1.0, $CHCl_3$). HPLC analysis of the product: Daicel CHIRALPAK AD-H column; 10% *i*-PrOH in hexanes; 1.0 mL/min; retention times: 9.3 min (major), 16.5 min (minor).

^{1}**H NMR** (400 MHz, $CDCl_3$) δ 7.40 (d, J = 8.8 Hz, 2H), 7.35-7.21 (m, 7H), 6.93 (d, J = 8.8 Hz, 2H), 6.89 (d, J = 8.8 Hz, 2H), 3.83 (s, 3H), 3.82 (s, 3H), 3.01-2.92 (m, 1H), 2.37 (s, 3H), 2.31-2.07 (m, 3H), 1.93-1.83 (m, 1H), 1.74-1.67 (m, 1H) ppm.

^{13}**C NMR** (100 MHz, $CDCl_3$) δ 212.8, 206.9 (2C), 159.4 (2C), 133.8, 129.6, 129.1, 128.9, 128.2, 127.7, 127.6, 126.6, 114.0, 113.9, 113.8, 107.5, 71.1, 55.3 (2C), 37.9, 31.3, 28.6, 18.8 ppm.

IR (thin film) 2956, 1702, 1605, 1507, 1244, 1172, 1029, 832, 696 cm^{-1}.

HRMS (CI+) Calcd for $C_{30}H_{28}O_4$ [M^+]: 452.1988, Found: 452.1989.

(*R*)-2-Acetyl-2-((S)-1-(4-chlorophenyl)-3-(4-methoxyphenyl)-3-(thiophen-2-yl)propa

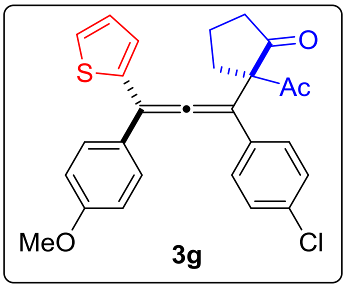

-1,2-dien-1-yl)cyclopentan-1-one (3g) was prepared as yellow oil from propargylic alcohol **1g** (0.2 mmol, 70.7 mg) **2a** (0.3 mmol, 36.3 μL) according to General Procedure **C** (3 h, purified by flash column chromatography: 5-10% EtOAc in hexanes, 78.6 mg, 85% yield, 5:1 d.r., 96% ee).

$[\alpha]_D^{25}$: +189.1 (*c* = 1.0, $CHCl_3$). HPLC analysis of the product: Daicel CHIRALPAK AD-H column; 5% *i*-PrOH in hexanes; 1.0 mL/min; retention times: 8.9 min (major), 15.4 min (minor).

^{1}H NMR (400 MHz, $CDCl_3$) δ 7.49 (d, *J* = 8.8 Hz, 2H), 7.31-7.24 (m, 5H), 7.11-6.91 (m, 4H), 3.85 (s, 3H), 2.98-2.90 (m, 1H), 2.42-2.18 (m, 3H), 2.33 (s, 3H), 2.01-1.89 (m, 2H) ppm.

^{13}C NMR (100 MHz, $CDCl_3$) δ 211.8, 207.3, 206.2, 159.7, 139.0, 133.8, 132.1, 129.5, 129.0, 128.3, 127.8, 126.9, 126.8, 125.5, 114.0 109.6, 107.2, 71.0, 55.3, 37.9, 31.3, 28.2, 18.9 ppm.

IR (thin film) 2958, 1702, 1605, 1508, 1247, 1030, 911, 828, 700 cm^{-1}.

HRMS (CI+) Calcd for $C_{27}H_{23}{}^{35}ClO_3S$ [M$^+$]: 462.1056, Found: 462.1052.

(*R*)-2-Acetyl-2-((*R*)-1-(4-chlorophenyl)-3-(4-methoxyphenyl)-3-phenylpropa-1,2-die

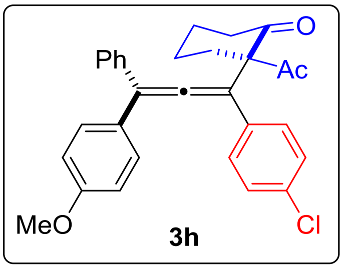

n-1-yl)cyclohexan-1-one (3h) was prepared as pale yellow foam from propargylic alcohol **1b** (0.2 mmol, 69.6 mg) and **2b** (0.3 mmol, 39.0 μL) with 200 mg anhydrous $MgSO_4$ according to General Procedure **C** (rt, 36 h, purified by flash column chromatography: 5-10% EtOAc in hexanes, 58.3 mg, 62% yield, 6:1 d.r., 90% ee).

$[\alpha]_D^{25}$: +41.9 (c = 1.0, $CHCl_3$). HPLC analysis of the product: Daicel CHIRALPAK AD-H column; 5% *i*-PrOH in hexanes; 1.0 mL/min; retention times: 8.6 min (major), 12.0 min (minor).

^{1}H NMR (400 MHz, $CDCl_3$) δ 7.48-7.21 (m, 11H), 6.89 (d, J = 8.8 Hz, 2H), 3.81 (s, 3H), 2.61-2.49 (m, 2H), 2.49-2.39 (m, 1H), 2.21-2.10 (m, 1H), 2.12 (s, 3H), 1.89-1.83 (m, 1H), 1.82-1.68 (m, 2H), 1.68-1.56 (m, 1H) ppm.

^{13}C NMR (100 MHz, $CDCl_3$) δ 209.0, 208.0, 207.8, 159.4, 135.5, 133.4, 133.1, 129.6, 129.1, 128.74, 128.71, 128.1, 128.0, 127.1, 114.1, 113.8, 108.4, 71.1, 55.3, 41.2, 34.2, 27.7, 27.1, 21.6 ppm.

IR (thin film) 2939, 1699, 1604, 1508, 1246, 1175, 1030, 905, 831, 729 cm^{-1}.

HRMS (CI+) Calcd for $C_{30}H_{27}{}^{35}ClO_3$ [M^+]: 470.1649, Found: 470.1651.

Ethyl (*S*)-1-((*R*)-1-(4-chlorophenyl)-3-(4-methoxyphenyl)-3-phenylpropa-1,2-dien-1-yl)-2-oxocyclopentane-1-carboxylate (3i) was prepared as pale yellow foam from propargylic alcohol **1b** (0.2 mmol, 69.6 mg) and **2c** (0.3 mmol, 44.5 μL) according to General Procedure **C** (-15 °C, 24 h, purified by flash column chromatography: 10% EtOAc and 20% CH_2Cl_2 in hexanes, 87.5 mg, 90% yield, 10:1 d.r., 82% ee).

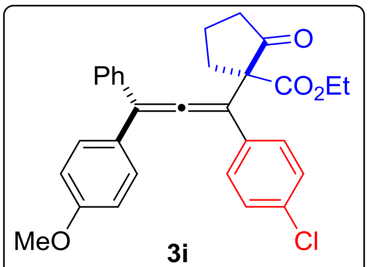

3i

$[\alpha]_D^{25}$: +156.1 (c = 1.0, $CHCl_3$). HPLC analysis of the product: Daicel CHIRALPAK IC column; 5% *i*-PrOH in hexanes; 1.0 mL/min; retention times: 8.5 min (minor), 10.8 min (major).

^{1}H NMR (400 MHz, $CDCl_3$) δ 7.48-7.26 (m, 11H), 6.89 (d, J = 8.8 Hz, 2H), 4.35-4.17 (m, 2H), 3.82 (s, 3H), 2.78-2.69 (m, 1H), 2.40-2.34 (m, 1H), 2.23-2.13 (m, 1H), 2.04-1.95 (m, 1H), 1.95-1.87 (m, 1H), 1.70-1.62 (m, 1H), 1.24 (t, J = 7.2 Hz, 3H) ppm.

^{13}**C NMR** (100 MHz, $CDCl_3$) δ 210.6, 206.8, 170.6, 159.4, 135.8, 133.2, 132.4, 129.7, 128.7, 128.6, 128.3, 128.1, 128.0, 127.1, 114.3, 113.9, 105.8, 64.6, 62.0, 55.3, 36.4, 33.0, 18.9, 14.0 ppm.

IR (thin film) 2977, 1718, 1605, 1509, 1246, 1176, 1029, 906, 831, 699 cm^{-1}.

HRMS (CI+) Calcd for $C_{30}H_{27}{}^{35}ClO_3$ [M^+]: 486.1598, Found: 486.1581.

(*R*)-3-(1-(4-Chlorophenyl)-3-(4-methoxyphenyl)-3-phenylpropa-1,2-dien-1-yl)-3-methylpentane-2,4-dione (3j) was prepared as pale yellow foam from propargylic alcohol **1b** (0.2 mmol, 69.6 mg) and **2d** (0.3 mmol, 34.9 μL) according to General Procedure **C** (0 °C, 10 h, purified by flash column chromatography: 5-10% EtOAc in hexanes, 72.1 mg, 81% yield, 78% ee).

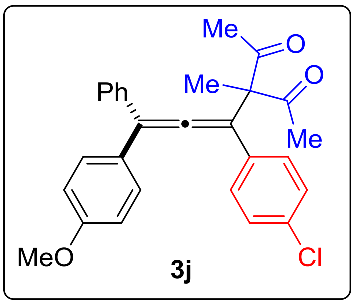

3j

$[\alpha]_D^{25}$: +74.5 (c = 1.0, $CHCl_3$). HPLC analysis of the product: Daicel CHIRALPAK AD-H column; 5% *i*-PrOH in hexanes; 1.0 mL/min; retention times: 8.4 min (major), 9.8 min (minor).

^{1}**H NMR** (400 MHz, $CDCl_3$) δ 7.41-7.37 (m, 4H), 7.37-7.21 (m, 7H), 6.92 (d, J = 8.8 Hz, 2H), 3.83 (s, 3H), 2.17 (s, 6H), 1.65 (m, 3H) ppm.

^{13}**C NMR** (100 MHz, $CDCl_3$) δ 207.2, 207.1 (2C), 159.5, 135.4, 133.5, 132.9, 129.4, 128.8, 128.76, 128.68, 128.2, 128.0, 127.1, 114.2, 114.0, 108.8, 68.7, 55.3, 27.8, 27.7, 20.3 ppm.

IR (thin film) 2933, 1701, 1604, 1508, 1247, 1090, 906, 831, 730 cm^{-1}.

HRMS (CI+) Calcd for $C_{28}H_{25}{}^{35}ClO_3$ [M^+]: 444.1492, Found: 444.1495.

(*R*)-2-Acetyl-2-((*S*)-3-(2-fluorophenyl)-3-(4-hydroxyphenyl)-1-phenylpropa-1,2-dien-1-yl)cyclopentan-1-one (3k) was prepared as pale yellow solid from propargylic alcohol **1h** (0.2 mmol, 63.8 mg) according to General Procedure **C** (purified by flash column chromatography: 10-20% EtOAc in hexanes, 65.3 mg, 75% yield, 21:1 d.r., 94% ee).

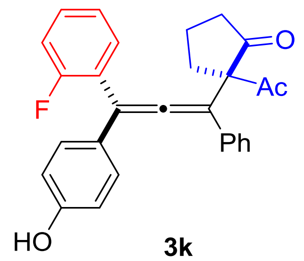

3k

$[\alpha]_D^{25}$: +103.7 (c = 1.0, $CHCl_3$). HPLC analysis of the product: Daicel CHIRALPAK IC column; 5% *i*-PrOH in hexanes; 1.0 mL/min; retention times: 20.7 min (major), 26.5 min (minor).

^{1}H NMR (400 MHz, CD_2Cl_2) δ 7.39-7.31 (m, 6H), δ 7.31-7.21 (m, 1H), δ 7.21-7.13 (m, 4H), 6.82 (d, J = 8.8 Hz, 2H), 6.23 (s br, 1H), 2.98-2.91 (m, 1H), 2.35 (s, 3H), 2.31-2.25 (m, 2H), 2.25-2.18 (m, 1H), 1.91-1.82 (m, 1H), 1.71-1.59 (m, 1H) ppm.

^{13}C NMR (100 MHz, CD_2Cl_2) δ 213.7, 207.3, 206.7, 160.6 (d, $J_{C\text{-}F}$ = 123.2 Hz), 156.5, 134.0, 131.6 (d, $J_{C\text{-}F}$ = 3 Hz), 130.2 (d, $J_{C\text{-}F}$ = 8 Hz), 129.2, 129.0, 128.2, 127.3, 127.1, 124.8 (d, $J_{C\text{-}F}$ = 3 Hz), 124.0 (d, $J_{C\text{-}F}$ = 15 Hz), 116.4 (d, $J_{C\text{-}F}$ = 22 Hz), 116.0, 108.7, 108.5, 71.7, 38.8, 32.6, 28.5, 19.4 ppm.

^{19}F NMR (376 MHz, CD_2Cl_2) δ -112.9 ppm.

IR (thin film) 3569, 2950, 1665, 1508, 1259, 1103, 832 cm^{-1}.

HRMS (CI+) Calcd for $C_{28}H_{23}FO_3$ [M^+]: 426.1631, Found: 426.1629.

(*R*)-2-Acetyl-2-((*R*)-3-(4-hydroxyphenyl)-1,3-diphenylpropa-1,2-dien-1-yl)cyclopentan-1-one (3l) was prepared as pale yellow solid from propargylic alcohol **1i** (0.2 mmol, 60.1 mg) according to General Procedure **C** (purified by flash column chromatography: 10-20% EtOAc in hexanes, 69.5 mg, 85% yield, 16:1 d.r., 94% ee).

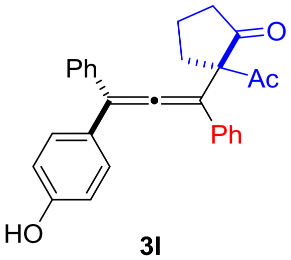

3l

$[\alpha]_D^{25}$: +217.9 (c = 1.0, $CHCl_3$). HPLC analysis of the product: Daicel CHIRALPAK AD-H column; 10% *i*-PrOH in hexanes; 0.5 mL/min; retention times: 20.7 min (minor), 21.6 min (major).

^{1}H NMR (400 MHz, CD_2Cl_2) δ 7.38-7.19 (m, 12H), 6.85 (d, J = 8.4 Hz, 2H), 6.02 (br s, 1H), 2.96-2.87 (m, 1H), 2.33 (s, 3H), 2.31-2.17 (m, 3H), 1.93-1.82 (m, 1H), 1.75-1.62 (m, 1H) ppm.

^{13}**C NMR** (100 MHz, CD_2Cl_2) δ 213.7, 207.6, 207.1, 156.4, 136.5, 134.2, 130.1, 129.3, 129.0, 128.5, 128.4, 128.1, 127.7, 127.2, 115.9, 114.7, 108.3, 71.6, 38.4, 32.1, 28.7, 19.2 ppm.

IR (thin film) 3435, 1703, 1610, 1511, 1216, 763, 696 cm^{-1}.

HRMS (CI+) Calcd for $C_{28}H_{24}O_3$ [M^+]: 408.1725, Found: 408.1720.

(*R*)-2-Acetyl-2-((*R*)-3-(4-hydroxyphenyl)-1-(4-methoxyphenyl)-3-phenylpropa-1,2-dien-1-yl)cyclopentan-1-one (3m) was prepared as pale yellow solid from propargylic alcohol **1j** (0.2 mmol, 66.1 mg) according to General Procedure **C** (purified by flash column chromatography: 10-20% EtOAc in hexanes, 71.0 mg, 81% yield, 10:1 d.r., 94% ee).

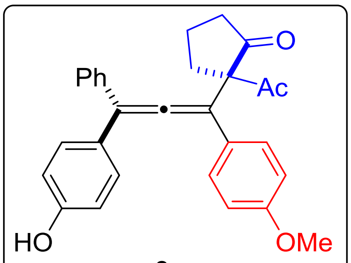

3m

$[\alpha]_D^{25}$: +170.7 (c = 1.0, $CHCl_3$). HPLC analysis of the product: Daicel CHIRALPAK IC column; 5% EtOH in hexanes; 0.5 mL/min; retention times: 23.4 min (major), 24.4 min (minor).

^{1}**H NMR** (400 MHz, CD_2Cl_2) δ 7.46-7.17 (m, 9H), 6.87-6.83 (m, 4H), 3.78 (s, 3H), 2.93-2.83 (m, 1H), 2.32 (s, 3H), 2.28-2.05 (m, 3H), 1.99-1.83 (m, 1H), 1.73-1.59 (m, 1H) ppm.

^{13}**C NMR** (100 MHz, CD_2Cl_2) δ 213.6, 207.0 (2C), 159.7, 156.2, 136.7, 130.1, 129.8, 129.0, 128.4, 128.2, 128.0, 126.1, 115.8, 114.7, 114.4, 108.0, 71.7, 55.7, 38.4, 32.0, 28.6, 19.2 ppm.

IR (thin film) 3362, 1695, 1607, 1508, 1355, 1252, 1178, 831, 754 cm^{-1}.

HRMS (CI+) Calcd for $C_{29}H_{26}O_4$ [M^+]: 438.1831, Found: 438.1815.

(*R*)-2-Acetyl-2-((*R*)-1-(4-chlorophenyl)-3-(4-hydroxyphenyl)-3-phenylpropa-1,2-dien-1-yl)cyclopentan-1-one (3n) was prepared as pale yellow solid from propargylic alcohol **1k** (0.2 mmol, 67.3 mg) according to General Procedure **C** (purified by flash column chromatography: 10-20% EtOAc in hexanes, 84.2 mg, 94% yield, 13:1 d.r., 97% ee).

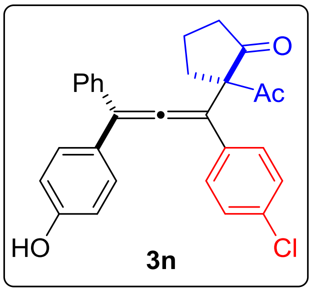

$[\alpha]_D^{25}$: +253.3 (*c* = 1.0, $CHCl_3$). HPLC analysis of the product: Daicel CHIRALPAK AD-H column; 10% *i*-PrOH in hexanes; 0.5 mL/min; retention times: 21.9 min (minor), 23.7 min (major).

^{1}H NMR (400 MHz, CD_2Cl_2) δ 7.41-7.21 (m, 11H), 6.85 (d, *J* = 8.4 Hz, 2H), 5.77 (br s, 1H), 2.93-2.85 (m, 1H), 2.29 (s, 3H), 2.26-2.09 (m, 3H), 1.92-1.79 (m, 1H), 1.70-1.59 (m, 1H) ppm.

^{13}C NMR (100 MHz, CD_2Cl_2) δ 213.4, 207.7, 206.7, 156.5, 136.2, 133.8, 133.0, 130.2, 129.4, 129.1, 128.6, 128.49, 128.47, 127.4, 115.9, 115.0, 114.4, 107.6, 71.5, 38.4, 32.2, 28.5, 19.3 ppm.

IR (thin film) 3584, 1704, 1610, 1511, 1353, 1215, 1172, 834, 697 cm^{-1}.

HRMS (CI+) Calcd for $C_{28}H_{23}{}^{35}ClO_3$ [M^+]: 442.1336, Found: 442.1322.

(*R*)-2-Acetyl-2-((*S*)-1-(furan-2-yl)-3-(4-hydroxyphenyl)-3-phenylpropa-1,2-dien-1-yl)

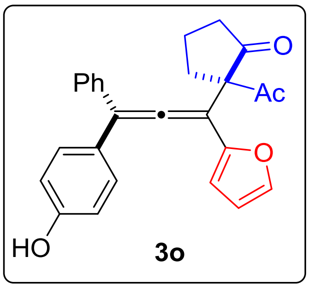

cyclopentan-1-one (3o) was prepared as white solid from propargylic alcohol **1l** (0.2 mmol, 58.1 mg) according to General Procedure **C** (purified by flash column chromatography: 10-20% EtOAc in hexanes, 71.7 mg, 90% yield, 12:1 d.r., 93% ee).

$[\alpha]_D^{25}$: +177.7 (*c* = 1.0, $CHCl_3$). HPLC analysis of the product: Daicel CHIRALPAK AD-H column; 10% *i*-PrOH in hexanes; 1.0 mL/min; retention times: 12.9 min (minor), 13.7 min (major).

^{1}H NMR (400 MHz, CD_2Cl_2) δ 7.46-7.16 (m, 8H), 6.86 (d, *J* = 8.0 Hz, 2H), 6.42 (s, 1H), 6.35-6.15 (m, 2H), 2.91-2.78 (m, 1H), 2.38 (s, 3H), 2.27-2.16 (m, 2H), 2.15-2.05 (m, 1H), 1.91-1.79 (m, 1H), 1.71-1.59 (m, 1H) ppm.

^{13}C NMR (100 MHz, CD_2Cl_2) δ 213.3, 206.0, 205.7, 156.6, 147.5, 143.1, 136.5, 130.3, 129.0, 128.8, 128.5, 127.6, 115.9, 112.0, 108.9, 101.4, 70.9, 38.4, 32.0, 28.1, 19.3 ppm.

IR (thin film) 3393, 3022, 2968, 1702, 1507, 1217, 1019, 830, 755 cm^{-1}.

HRMS (CI+) Calcd for $C_{26}H_{22}O_4$ [M^+]: 398.1518, Found: 398.1515.

(*R*)-2-Acetyl-2-((*S*)-3-(4-hydroxyphenyl)-3-phenyl-1-(thiophen-3-yl)propa-1,2-dien-1-yl)cyclopentan-1-one (3p) was prepared as pale yellow solid from propargylic alcohol **1m** (0.2 mmol, 61.3 mg) according to General Procedure **C** (purified by flash column chromatography: 10-20% EtOAc in hexanes, 71.1 mg, 86% yield, 13:1 dr, 94% ee).

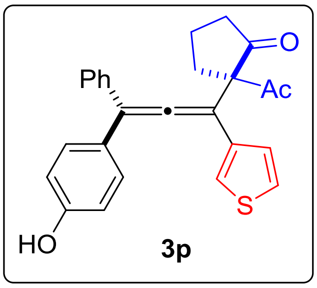

$[\alpha]_D^{25}$: +185.7 (*c* = 1.0, $CHCl_3$). HPLC analysis of the product: Daicel CHIRALPAK AD-H column; 10% *i*-PrOH in hexanes; 1.0 mL/min; retention times: 12.6 min (minor), 14.2 min (major).

^{1}H NMR (400 MHz, CD_2Cl_2) δ 7.52-7.28 (m, 8H), 7.14 (d, *J* = 5.2 Hz, 1H), 7.05-7.03 (m, 1H), 6.85 (d, *J* = 8.8 Hz, 2H), 5.85 (s br, 1H), 2.91-2.81 (m, 1H), 2.36-2.29 (m, 1H), 2.35 (s, 3H), 2.29-2.16 (m, 1H), 2.16-2.10 (m, 1H), 1.88-1.68 (m, 1H), 1.71-1.61 (m, 1H) ppm.

^{13}C NMR (100 MHz, CD_2Cl_2) δ 213.4, 207.2, 206.9, 156.3, 136.5, 135.0, 130.2, 130.0, 128.6, 128.3, 127.8, 127.6, 126.4, 121.6, 114.2, 104.7, 71.8, 38.4, 31.8, 28.3, 19.2 ppm.

IR (thin film) 3437, 1697, 1508, 1214, 757 cm^{-1}.

HRMS (CI+) Calcd for $C_{26}H_{22}O_3S$ [M$^+$]: 414.1290, Found: 414.1292.

General Procedure D

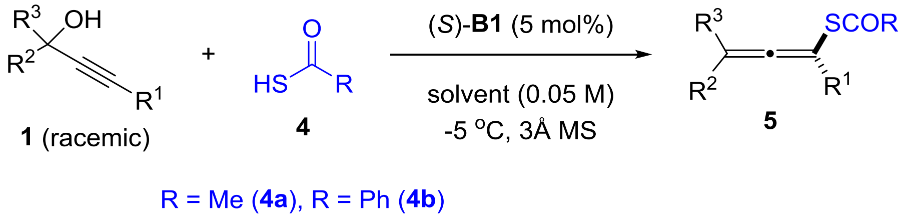

At room temperature, an oven-dried 10-mL vial was charged with a mixture of the alcohol substrate **1** (0.3 mmol), 3Å molecular sieves (90 mg), and the solvent (6.0 mL, toluene for **5a-g** and DCM for others, or noted otherwise). The reaction mixture was cooled to –5 °C, and a solution of the catalyst (*S*)-**B1**[6] (0.015 mmol, 5 mol%) in the same solvent (0.2 mL) and thioacetic acid **4a** (0.6 mmol, 42.9 uL) were added in one

portion. The reaction progress was monitored by thin layer chromatography. Upon completion, the reaction mixture was directly subjected to silica gel column flash chromatography (the column was pretreated with 10% AcOH in hexanes) to afford the desired product **5**.

Note: All the racemic products (used for HPLC reference in ee determination), were prepared using the same reaction with the simplest racemic phosphoric acid catalyst.

(*R*)-*S*-(3-(4-Hydroxyphenyl)-1,3-diphenylpropa-1,2-dien-1-yl) ethanethioate (5a)

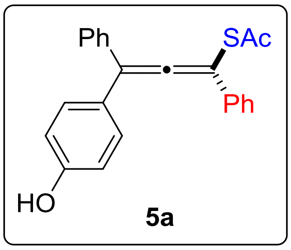

was prepared as brown oil from substrate **1i** (0.3 mmol, 90.2 mg) according to the General Procedure D (purified by flash column chromatography: 10% EtOAc and 1% AcOH in hexanes, 88.1 mg, 82% yield, 94% ee).

$[\alpha]_D^{25}$: +12.2 (c = 1.0, $CHCl_3$). HPLC analysis of the product: Daicel CHIRALPAK AD-H column; 15% *i*-PrOH in hexanes; 1.0 mL/min; retention times: 6.9 min (minor), 11.0 min (major).

^{1}H NMR (400 MHz, CD_2Cl_2) δ 7.61 (d, J = 7.6 Hz, 2H), 7.45-7.32 (m, 7H), 7.29 (d, J = 8.0 Hz, 3H), 6.83 (d, J = 8.8 Hz, 2H), 2.44 (s, 3H) ppm.

^{13}C NMR (100 MHz, CD_2Cl_2) δ 211.7, 194.6, 156.5, 135.4, 134.7, 130.6, 129.2, 129.1, 129.0, 128.6, 128.4, 127.0, 126.8, 116.0, 112.9, 99.8, 30.4 ppm.

IR (thin film) 3443, 3064, 2925, 1683, 1504, 1267, 1117, 834, 760 cm^{-1}.

HRMS (CI+) Calcd for $C_{23}H_{18}O_2S$ [M$^+$]: 358.1028, Found: 358.1036.

(*R*)-*S*-(3-(4-Hydroxyphenyl)-1-(4-methoxyphenyl)-3-phenylpropa-1,2-dien-1-yl)ethanethioate ethanethioate (5b)

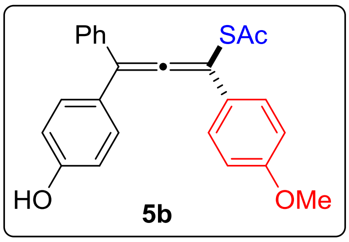

was prepared as brown oil from substrate **1j** (0.3 mmol, 99.2 mg) according to the General Procedure D (purified by flash column chromatography: 20% EtOAc and 1% AcOH in hexanes, 87.3 mg, 75% yield, 91% ee).

$[\alpha]_D^{25}$: –17.1 (c = 1.0, $CHCl_3$). HPLC analysis of the product: Daicel CHIRALPAK AD-H column; 20% *i*-PrOH in hexanes; 1.0 mL/min; retention times: 8.5 min (minor), 14.3 min (major).

^{1}H NMR (400 MHz, CD_2Cl_2) δ 7.55 (d, J = 8.8 Hz, 2H), 7.46-7.33 (m, 5H), 7.28 (d, J = 8.4 Hz, 2H), 6.90 (d, J = 8.8 Hz, 2H), 6.82 (d, J = 8.4 Hz, 2H), 3.80 (s, 3H), 2.44 (s, 3H) ppm.

^{13}C NMR (100 MHz, CD_2Cl_2) δ 211.0, 195.2, 160.0, 156.6, 135.7, 130.6, 129.2, 129.0, 128.5, 128.0, 127.0, 126.8, 116.0, 114.5, 112.8, 99.4, 55.7, 30.4 ppm.

IR (thin film) 3405, 3065, 1702, 1606, 1509, 1250, 1112, 833, 769 cm^{-1}.

HRMS (CI+) Calcd for $C_{24}H_{20}O_3S$ [M^+]: 388.1133, Found: 388.1145.

(*R*)-*S*-(1-(4-Fluorophenyl)-3-(4-hydroxyphenyl)-3-phenylpropa-1,2-dien-1-yl) ethanethioate (5c) was prepared as brown oil from substrate **1n** (0.3 mmol, 95.4 mg) according to the General Procedure D (purified by flash column chromatography: 10% EtOAc and 1% AcOH in hexanes, 93.6 mg, 83% yield, 92% ee).

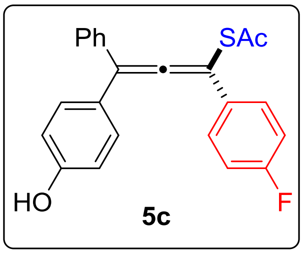

$[\alpha]_D^{25}$: +8.1 (c = 1.0, $CHCl_3$). HPLC analysis of the product: Daicel CHIRALPAK AD-H column; 15% *i*-PrOH in hexanes; 1.0 mL/min; retention times: 7.3 min (minor), 10.9 min (major).

^{1}H NMR (400 MHz, CD_2Cl_2) δ 7.61-7.56 (m, 2H), 7.44-7.34 (m, 5H), 7.28 (d, J = 8.4 Hz, 2H), 7.06 (d, J = 8.8 Hz, 2H), 6.83 (d, J = 8.8 Hz, 2H), 5.56 (br s, 1H), 2.44 (s, 3H) ppm.

^{13}C NMR (100 MHz, CD_2Cl_2) δ 211.5, 194.5, 162.9 (d, J_{C-F} = 246 Hz), 156.5, 135.3, 130.9, 130.6, 129.2, 129.0, 128.6 (d, J_{C-F} = 6.7 Hz), 128.5, 126.9, 115.9 (d, J_{C-F} = 21.9 Hz), 115.8, 113.0, 98.9, 30.5 ppm.

^{19}F NMR (376 MHz, CD_2Cl_2) δ -114.8 ppm.

IR (thin film) 3402, 3028, 2927, 1691, 1505, 1226, 1118, 835 cm^{-1}.

HRMS (CI+) Calcd for $C_{23}H_{17}FO_2S$ [M^+]: 376.0933, Found: 376.0934

(*R*)-*S*-(1-(4-Chlorophenyl)-3-(4-hydroxyphenyl)-3-phenylpropa-1,2-dien-1-yl) ethanethioate (5d) was prepared as brown oil from substrate **1k** (0.3 mmol, 100.2 mg) according to the General Procedure D (purified by flash column chromatography: 10% EtOAc and 1% AcOH in hexanes, 106.1 mg, 90% yield, 92% ee).

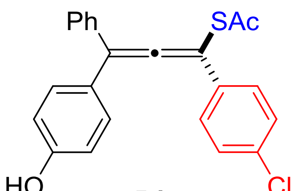

5d

$[\alpha]_D^{25}$: +5.3 (c = 1.0, $CHCl_3$). HPLC analysis of the product: Daicel CHIRALPAK AD-H column; 15% *i*-PrOH in hexanes; 1.0 mL/min; retention times: 8.9 min (minor), 16.5 min (major).

^{1}H NMR (400 MHz, CD_2Cl_2) δ 7.55 (d, J = 8.0 Hz, 2H), 7.43-7.31 (m, 7H), 7.28 (d, J = 8.0 Hz, 2H), 6.83 (d, J = 8.0 Hz, 2H), 5.63 (br s, 1H), 2.44 (s, 3H) ppm.

^{13}C NMR (100 MHz, CD_2Cl_2) δ 211.8, 194.5, 156.6, 135.1, 134.0, 133.5, 130.6, 129.2, 129.15, 129.05, 128.7, 128.1, 126.7, 116.0, 113.3, 98.9, 30.5 ppm.

IR (thin film) 3395, 1682, 1609, 1510, 1218, 1172, 1012, 830, 769 cm^{-1}.

HRMS (CI+) Calcd for $C_{23}H_{17}{}^{35}ClO_2S$ [M^+]: 392.0638, Found: 392.0649

(*R*)-*S*-(1-(3,5-Dimethylphenyl)-3-(4-hydroxyphenyl)-3-phenylpropa-1,2-dien-1-yl)ethanethioate (5e) was prepared as brown oil from substrate **1o** (0.3 mmol, 98.4 mg) according to the General Procedure D (purified by flash column chromatography: 10% EtOAc and 1% AcOH in hexanes, 107.3 mg, 93% yield, 92% ee).

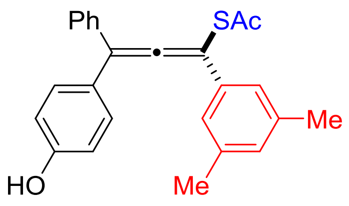

5e

$[\alpha]_D^{25}$: +13.8 (c = 1.0, $CHCl_3$). HPLC analysis of the product: Daicel CHIRALPAK AD-H column; 10% *i*-PrOH in hexanes; 1.0 mL/min; retention times: 5.1 min (minor), 5.9 min (major).

^{1}H NMR (400 MHz, CD_2Cl_2) δ 7.45-7.33 (m, 5H), 7.29 (d, J = 8.8 Hz, 2H), 7.23 (s, 2H), 6.93 (s, 1H), 6.83 (d, J = 8.8 Hz, 2H), 5.54 (br s, 1H), 2.43 (s, 3H), 2.30 (s, 6H) ppm.

^{13}C NMR (100 MHz, CD_2Cl_2) δ 211.6, 194.9, 156.4, 138.8, 135.5, 134.4, 130.6, 130.2, 129.2, 129.0, 128.5, 127.1, 124.5, 115.9, 112.5, 99.9, 30.4, 21.5 ppm.

IR (thin film) 3387, 3029, 2924, 1684, 1508, 1267, 1116, 839, 758 cm^{-1}.

HRMS (CI+) Calcd for $C_{25}H_{22}O_2S$ [M+]: 386.1341, Found: 386.1346.

(*R*)-*S*-(3-(4-Hydroxyphenyl)-3-phenyl-1-(thiophen-3-yl)propa-1,2-dien-1-yl)ethanethioate ethanethioate (5f) was prepared as brown oil from substrate **1l** (0.3 mmol, 87.1 mg) according to the General Procedure D (purified by flash column chromatography: 10% EtOAc and 1% AcOH in hexanes, 78.3 mg, 75% yield, 91% ee).

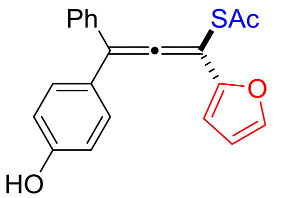

5f

$[\alpha]_D^{25}$: +9.2 (*c* = 1.0, $CHCl_3$). HPLC analysis of the product: Daicel CHIRALPAK AD-H column; 15% *i*-PrOH in hexanes; 1.0 mL/min; retention times: 7.9 min (major), 9.4 min (minor).

The pure **5f** is relatively unstable, but it can be stored as a solution in DCM or $CHCl_3$ in the refrigerator. However, its TBS-protected derivative **5f′** is stable. Therefore, the characterization of **5f′** is provided. At 0 °C, to a solution of **5f** (0.2 mmol, 69.6 mg) in CH_2Cl_2 (1 mL) were added 2,6-lutidine (83.2 uL, 0.72 mmol, 3.6 equiv) and a solution of TBSOTf (99.3 uL, 0.44 mmol, 2.2 equiv) in CH_2Cl_2 (0.2 mL) sequentially. The reaction was stirred at 0 °C for 2.5 h and then warmed to room temperature. The reaction mixture was directly subjected silica gel column chromatography (eluent: 3% Et_2O in hexanes, pale yellow oil, 0.62 g, quant. yield).

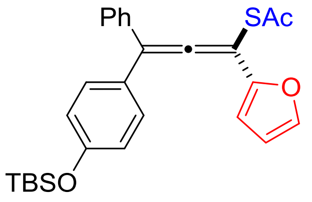

5f'

^{1}H NMR (400 MHz, $CDCl_3$) δ 7.46 (d, *J* = 8.4 Hz, 2H), 7.41-7.30 (m, 6H), 6.85 (d, *J* = 8.4 Hz, 2H), 6.46-6.40 (m, 2H), 2.42 (s, 3H), 1.00 (s, 9H), 0.22 (s, 6H) ppm.

^{13}C NMR (100 MHz, $CDCl_3$) δ 210.4, 193.4, 155.9, 148.3, 143.1, 135.1, 130.3, 129.1, 128.5, 128.2, 127.3, 120.2, 114.1, 111.7, 109.1, 90.9, 30.0, 25.7, 18.2, 4.4 ppm.

IR (thin film) 3292, 2930, 1705, 1603, 1507, 1262, 1119, 910, 838 cm^{-1}.

HRMS (CI+) Calcd for $C_{27}H_{30}O_3SSi$ [M+]: 462.1685, Found: 462.1692.

(*R*)-*S*-(3-(4-Hydroxyphenyl)-3-phenyl-1-(thiophen-3-yl)propa-1,2-dien-1-yl) ethanethioate ethanethioate (5g) was prepared as brown oil from substrate **1m** (0.3 mmol, 91.8 mg) according to the General Procedure D (purified by flash column chromatography: 10% EtOAc and 1% AcOH in hexanes, 65.7 mg, 88% yield, 90% ee).

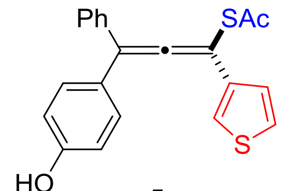

5g

$[\alpha]_D^{25}$: +8.0 (c = 1.0, $CHCl_3$). HPLC analysis of the product: Daicel CHIRALPAK AD-H column; 15% *i*-PrOH in hexanes; 1.0 mL/min; retention times: 8.3 min (minor), 16.0 min (major).

^{1}H NMR (400 MHz, CD_2Cl_2) δ 7.44-7.31 (m, 7H), 7.28 (d, J = 8.8 Hz, 2H), 7.22 (d, J = 5.2 Hz, 1H), 6.83 (d, J = 8.8 Hz, 2H), 5.60 (br s, 1H), 2.43 (s, 3H) ppm.

^{13}C NMR (100 MHz, CD_2Cl_2) δ 211.3, 195.1, 156.6, 137.0, 135.5, 130.7, 129.3, 129.0, 128.6, 127.1, 126.9, 126.5, 122.8, 116.0, 112.8, 95.2, 30.4 ppm.

IR (thin film) 3405, 3027, 1683, 1509, 1222, 1118, 841 cm^{-1}.

HRMS (CI+) Calcd for $C_{21}H_{16}O_2S_2$ [M$^+$]: 364.0592, Found: 364.0581

***S*-((*R*)-3-(4-Hydroxyphenyl)-1-((8*S*,9*S*,13*S*,14*S*)-13-methyl-17-oxo-7,8,9,11,12,13,14,15,16,17-decahydro-6H-cyclopenta[a]phenanthren-3-yl)-3-phenylpropa-1,2-dien-1-yl) ethanethioate (5h)** was prepared as red oil from substrate **1p** (0.2 mmol, 95.3 mg) according to the General Procedure D (purified by flash column chromatography: 20% EtOAc and 1% AcOH in hexanes, 81.3 mg, 76% yield, 89% de).

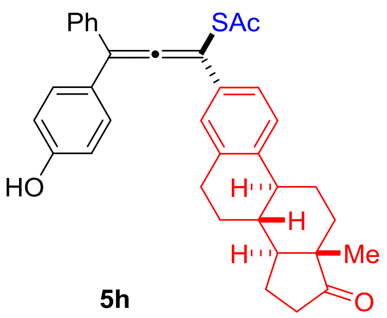

5h

$[\alpha]_D^{25}$: +86.6 (c = 1.0, $CHCl_3$). HPLC analysis of the product: Daicel CHIRALPAK AD-H column; 20% *i*-PrOH in hexanes; 1.0 mL/min; retention times: 8.7 min (minor), 9.9 min (major).

^{1}H NMR (400 MHz, CD_2Cl_2) δ 7.45-7.35 (m, 7H), 7.34-7.26 (m, 3H), 6.85 (d, J = 8.8 Hz, 2H), 5.61 (br s, 1H), 2.93-2.83 (m, 2H), 2.55-2.45 (m, 1H), 2.43 (s, 3H), 2.31-2.21 (m, 1H),

217-1.99 (m, 4H), 1.95-1.91 (m, 1H), 1.64-1.51 (m, 2H), 1.51-1.38 (m, 4H), 0.91 (m, 3H) ppm.

^{13}C NMR (100 MHz, CD_2Cl_2) δ 222.1, 211.5, 194.8, 156.8, 140.5, 137.6, 135.6, 131.9, 130.6, 129.2, 129.0, 128.5, 127.0, 126.8, 126.1, 124.1, 116.0, 112.7, 99.6, 50.8, 48.4, 44.8, 38.4, 36.3, 32.0, 30.4, 29.9, 26.8, 26.1, 21.9, 14.1 ppm.

IR (thin film) 3401, 2925, 1721, 1511, 1269, 1117, 838 cm^{-1}.

HRMS (CI+) Calcd for $C_{35}H_{35}O_3S$ [M+H$^+$]: 535.2307, Found: 535.2337.

(*R*)-*S*-(1-(Cyclohex-1-en-1-yl)-3-(4-hydroxyphenyl)-3-phenylpropa-1,2-dien-1-yl) ethanethioate (5i) was prepared as brown oil from substrate **1q** (0.3 mmol, 91.2 mg) according to the General Procedure D (purified by flash column chromatography: 10% EtOAc and 1% AcOH in hexanes, 76.1 mg, 70% yield, 91% ee). $[\alpha]_D^{25}$: +27.1 (c = 1.0, $CHCl_3$). HPLC analysis of the product: Daicel CHIRALPAK AD-H column; 10% *i*-PrOH in hexanes; 1.0 mL/min; retention times: 6.5 min (minor), 10.7 min (major).

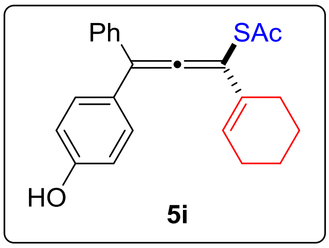

5i

^{1}H NMR (400 MHz, CD_2Cl_2) δ 7.38-7.28 (m, 5H), 7.23 (d, J = 8.8 Hz, 2H), 6.82 (d, J = 8.8 Hz, 2H), 6.18-6.14 (m, 1H), 2.37 (s, 3H), 2.24-2.21 (m, 2H), 2.21-2.16 (m, 2H), 1.71-1.61 (m, 2H), 1.61-1.56 (m, 2H) ppm.

^{13}C NMR (100 MHz, CD_2Cl_2) δ 210.3, 195.0, 156.2, 136.1, 131.5, 130.5, 129.1, 128.9, 128.2, 127.7, 127.6, 115.8, 111.9, 101.6, 30.3, 27.7, 26.3, 23.1, 22.5 ppm.

IR (thin film) 3446, 3027, 2930, 1681, 1507, 1268, 1119, 832, 757 cm^{-1}.

HRMS (CI+) Calcd for $C_{23}H_{22}O_2S$ [M$^+$]: 362.1341, Found: 362.1353.

(*R*)-*S*-(1-(4-Hydroxyphenyl)-4-methyl-1-phenylpenta-1,2,4-trien-3-yl) ethanethioate (5j) was prepared as brown oil from substrate **1r** (0.3 mmol, 79.2 mg) according to the General Procedure D (purified by flash column chromatography: 10% EtOAc and 1% AcOH in hexanes, 69.6 mg, 72% yield, 88% ee).

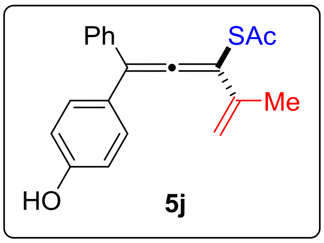

$[\alpha]_D^{25}$: –33.4 (c = 1.0, $CHCl_3$). HPLC analysis of the product: Daicel CHIRALPAK AD-H column; 10% *i*-PrOH in hexanes; 1.0 mL/min; retention times: 6.7 min (minor), 7.8 min (major).

^{1}H NMR (400 MHz, CD_2Cl_2) δ 7.39-7.33 (m, 4H), 7.33-7.31 (m, 1H), 7.25 (d, J = 8.8 Hz, 2H), 6.84 (d, J = 8.8 Hz, 2H), 5.74 (br s, 1H), 5.40 (s, 1H), 5.13 (s, 1H), 2.39 (s, 3H), 2.02 (s, 3H) ppm.

^{13}C NMR (100 MHz, CD_2Cl_2) δ 211.7, 194.9, 156.4, 138.8, 135.6, 130.6, 129.2, 128.9, 128.4, 127.2, 115.9, 115.1, 111.7, 101.3, 30.3, 21.5 ppm.

IR (thin film) 3403, 1681, 1510, 1269, 1122, 837 cm^{-1}.

HRMS (CI+) Calcd for $C_{20}H_{18}O_2S$ [M$^+$]: 322.1028, Found: 322.1016.

(*R*)-*S*-(3-(4-Hydroxyphenyl)-3-phenyl-1-(trimethylsilyl)propa-1,2-dien-1-yl) ethanethioate (5k) was prepared as brown oil from substrate **1s** (0.3 mmol, 89.1 mg) according to the General Procedure D (purified by flash column chromatography: 10% EtOAc and 1% AcOH in hexanes, 74.4 mg, 70% yield, 92% ee).

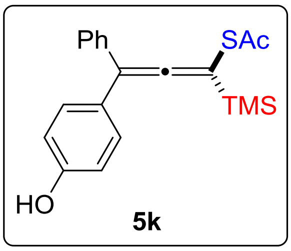

$[\alpha]_D^{25}$: –31.9 (c = 1.0, $CHCl_3$). HPLC analysis of the product: Daicel CHIRALCEL column; AD-H column; 5% *i*-PrOH in hexanes; 1.0 mL/min; retention times: 6.6 min (minor), 7.9 min (major).

^{1}H NMR (400 MHz, CD_2Cl_2) δ 7.37 (d, J = 8.0 Hz, 4H), 7.33-7.27 (m, 1H), 7.24 (d, J = 8.8 Hz, 3H), 6.84 (d, J = 8.8 Hz, 2H), 2.37 (s, 3H), 0.24 (s, 9H) ppm.

^{13}C NMR (100 MHz, CD_2Cl_2) δ 212.9, 195.6, 160.0, 136.1, 130.3, 128.9, 127.8 (2C), 127.5, 115.9, 107.2, 90.8, 30.3, –1.1 ppm.

IR (thin film) 3538, 1657, 1506, 1251, 1130, 834, 700 cm^{-1}.

HRMS (CI+) Calcd for $C_{20}H_{22}O_2SSi$ [M$^+$]: 354.1110, Found: 354.1107.

(*R*)-*S*-(3-(4-Hydroxyphenyl)-3-phenyl-1-(triethylsilyl)propa-1,2-dien-1-yl) ethanethioate (5l) was prepared as brown oil from substrate **1t** (0.3 mmol, 101.4 mg) according to the General Procedure D (purified by flash column chromatography: 10% EtOAc and 1% AcOH in hexanes, 107.4 mg, 90% yield, 90% ee).

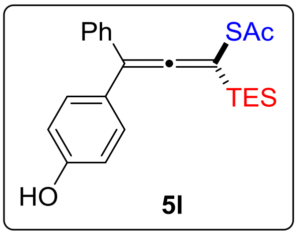

$[\alpha]_D^{25}$: –28.5 (*c* = 1.0, $CHCl_3$). HPLC analysis of the product: AD-H column; 3% *i*-PrOH in hexanes; 1.0 mL/min; retention times: 8.5 min (minor), 9.9 min (major).

^{1}H NMR (400 MHz, acetone-d_6) δ 8.55 (br s, 1H), 7.43-7.35 (m, 4H), 7.32-7.27 (m, 1H), 7.24 (d, *J* = 8.0 Hz, 2H), 6.88 (d, *J* = 8.4 Hz, 2H), 2.34 (s, 3H), 0.93 (t, *J* = 8.0 Hz, 9H), 0.73 (q, *J* = 8.0 Hz, 6H) ppm.

^{13}C NMR (100 MHz, acetone-d_6) δ 214.4, 193.5, 157.8, 136.5, 130.4, 129.1, 128.0 (2C), 126.6, 116.1, 107.0, 87.9, 30.0, 7.4, 3.9 ppm.

IR (thin film) 3447, 2955, 1662, 1507, 1227, 1119, 700 cm^{-1}.

HRMS (CI+) Calcd for $C_{23}H_{28}O_2SSi$ [M$^+$]: 396.1579, Found: 396.1574.

(*R*)-*S*-(1-(4-Hydroxyphenyl)-1-phenylhepta-1,2-dien-3-yl) ethanethioate (5m) was prepared as brown oil from substrate **1u** (0.3 mmol, 84.0 mg) according to the General Procedure D (purified by flash column chromatography: 10% EtOAc and 1% AcOH in hexanes, 96.2 mg, 95% yield, 92% ee).

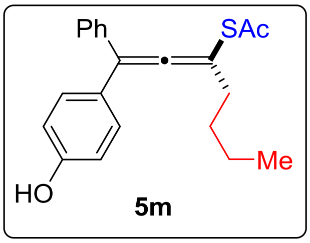

$[\alpha]_D^{25}$: –13.2 (*c* = 1.0, $CHCl_3$). HPLC analysis of the product: Daicel CHIRALPAK AD-H column; 5% *i*-PrOH in hexanes; 1.0 mL/min; retention times: 8.2 min (minor), 9.4 min (major).

^{1}H NMR (400 MHz, acetone-d_6) δ 8.56 (br s, 1H), 7.42-7.35 (m, 4H), 7.35-7.29 (m, 1H), 7.21 (d, *J* = 8.8 Hz, 2H), 6.88 (d, *J* = 8.8 Hz, 2H), 2.44 (t, *J* = 7.2 Hz, 2H), 2.35 (s, 3H), 1.61-1.52 (m, 2H), 1.39-1.28 (m, 2H), 0.85 (t, *J* = 7.2 Hz, 3H) ppm.

^{13}C NMR (100 MHz, acetone-d_6) δ 208.1, 194.0, 158.0, 136.7, 130.5, 129.3, 129.1, 128.4, 126.9, 116.1, 111.2, 98.9, 35.6, 30.5, 30.0, 22.5, 13.9 ppm.

IR (thin film) 3445, 3029, 2927, 1674, 1507, 1220, 1115, 832, 698 cm^{-1}.

HRMS (CI+) Calcd for $C_{21}H_{22}O_2S$ [M^+]: 338.1341, Found: 338.1338.

(*R*)-*S*-(1-(4-Hydroxyphenyl)-1,5-diphenylpenta-1,2-dien-3-yl) ethanethioate (5n) was prepared as brown oil from substrate **1v** (0.3 mmol, 98.4 mg) according to the General Procedure D (purified by flash column chromatography: 10% EtOAc and 1% AcOH in hexanes, 113.6 mg, 98% yield, 94% ee).

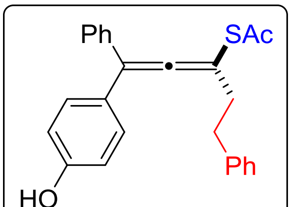

5n

$[\alpha]_D^{25}$: –20.1 (c = 1.0, $CHCl_3$). HPLC analysis of the product: Daicel CHIRALPAK AD-H column; 5% *i*-PrOH in hexanes; 1.0 mL/min; retention times: 17.9 min (minor), 19.1 min (major).

^{1}H NMR (400 MHz, CD_2Cl_2) δ 7.36-7.29 (m, 3H), 7.27-7.16 (m, 7H), 7.12 (d, J = 8.8 Hz, 2H), 6.80 (d, J = 8.8 Hz, 2H), 5.49 (br s, 1H), 2.91 (t, J = 7.6 Hz, 2H), 2.77 (t, J = 8.0 Hz, 2H), 2.38 (s, 3H) ppm.

^{13}C NMR (100 MHz, CD_2Cl_2) δ 208.3, 195.5, 156.1, 141.4, 136.1, 130.6, 129.2, 128.9, 128.8, 128.7, 128.1, 127.7, 126.4, 115.7, 111.2, 98.1, 37.3, 34.3, 30.6 ppm.

IR (thin film) 3405, 1674, 1510, 1267, 1115, 837 cm^{-1}.

HRMS (CI+) Calcd for $C_{25}H_{22}O_2S$ [M^+]: 386.1341, Found: 386.1331.

(*R*)-*S*-(1-(4-Hydroxyphenyl)-1-phenylhexa-1,2,5-trien-3-yl) ethanethioate (5o) was prepared as brown oil from propargylic substrate **1w** (0.3 mmol, 79.2 mg) according to the General Procedure D (purified by flash column chromatography: 10% EtOAc and 1% AcOH in hexanes, 90.2 mg, 92% yield, 93% ee).

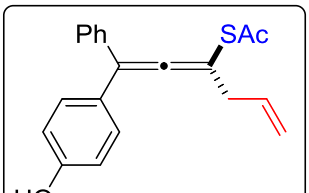

5o

$[\alpha]_D^{25}$: –17.7 (c = 1.0, $CHCl_3$). HPLC analysis of the product: Daicel CHIRALPAK AD-H column; 5% *i*-PrOH in hexanes; 1.0 mL/min; retention times: 10.7 min (minor), 12.6 min (major).

^{1}**H NMR** (400 MHz, CD_2Cl_2) δ 7.37-7.28 (m, 5H), 7.23 (d, J = 8.8 Hz, 2H), 6.83 (d, J = 8.8 Hz, 2H), 5.97-5.87 (m, 1H), 5.62 (br s, 1H), 5.20-5.09 (m, 2H), 3.19 (d, J = 6.8 Hz, 2H), 2.36 (s, 3H) ppm.

^{13}**C NMR** (100 MHz, CD_2Cl_2) δ 208.6, 195.0, 156.2, 136.1, 134.8, 130.6, 129.2, 128.8, 128.3, 127.8, 117.5, 115.8, 111.3, 97.1, 40.1, 30.5 ppm.

IR (thin film) 3315, 3078, 1675, 1510, 1269, 1116, 837 cm^{-1}.

HRMS (CI+) Calcd for $C_{20}H_{18}O_2S$ [M$^+$]: 322.1028, Found: 322.1017.

(*R*)-*S*-(1-(4-Hydroxyphenyl)-1-phenylundeca-1,2-dien-10-yn-3-yl) ethanethioate (5p)

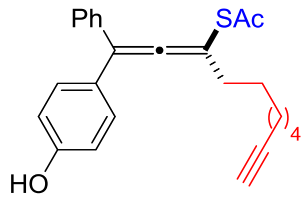

5p

was prepared as brown oil from substrate **1x** (0.3 mmol, 99.8 mg) according to the General Procedure D (purified by flash column chromatography: 10% EtOAc and 1% AcOH in hexanes, 75.5 mg, 64% yield, 91% ee). $[\alpha]_D^{25}$: –17.7 (c = 1.0, $CHCl_3$). HPLC analysis of the product: Daicel CHIRALPAK AD-H column; 5% *i*-PrOH in hexanes; 1.0 mL/min; retention times: 11.9 min (minor), 14.9 min (major).

^{1}**H NMR** (400 MHz, CD_2Cl_2) δ 7.42-7.34 (m, 4H), 7.34-7.27 (m, 1H), 7.24 (d, J = 8.8 Hz, 2H), 6.85 (d, J = 8.8 Hz, 2H), 2.45 (t, J = 7.2 Hz, 2H), 2.37 (s, 3H), 2.13-2.08 (3, 2H), 1.97-1.95 (m, 1H), 1.64-1.54 (m, 2H), 1.47-1.27 (m, 6H) ppm.

^{13}**C NMR** (100 MHz, CD_2Cl_2) δ 208.0, 195.7, 156.2, 136.3, 130.5, 129.1, 128.8, 128.2, 127.8, 115.8, 111.0, 98.6, 85.1, 68.4, 35.7, 30.5, 28.86, 28.85, 28.8, 28.1, 18.6 ppm.

IR (thin film) 3629, 3295, 2932, 1683, 1508, 1267, 1118, 833, 769 cm^{-1}.

HRMS (CI+) Calcd for $C_{25}H_{26}O_2S$ [M$^+$]: 390.1654, Found: 390.1671.

(*R*)-*S*-(5-((Tert-butyldimethylsilyl)oxy)-1-(4-hydroxyphenyl)-1-phenylpenta-1,2-die n-3-yl) ethanethioate (5q) was prepared as brown oil from substrate **1y** (0.3 mmol, 114.6 mg) according to the General Procedure D (purified by flash column chromatography: 10% EtOAc and 1% AcOH in hexanes, 108.6 mg, 82% yield, 93% ee).

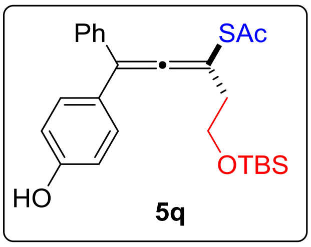

$[\alpha]_D^{25}$: –16.2 (c = 1.0, $CHCl_3$). HPLC analysis of the product: Daicel CHIRALCEL OD-H column; 5% *i*-PrOH in hexanes; 0.5 mL/min; retention times: 13.1 min (minor), 14.2 min (major).

^{1}H NMR (400 MHz, CD_2Cl_2) δ 7.35 (d, J = 8.0 Hz, 4H), 7.34-7.27 (m, 1H), 7.24 (d, J = 8.8 Hz, 2H), 6.82 (d, J = 8.8 Hz, 2H), 3.84 (t, J = 6.4 Hz, 2H), 2.66 (t, J = 6.4 Hz, 2H), 2.36 (s, 3H), 0.86 (s, 9H), 0.03 (s, 6H) ppm.

^{13}C NMR (100 MHz, CD_2Cl_2) δ 208.8, 195.2, 156.2, 136.1, 130.7, 129.3, 128.8, 128.2, 127.8, 115.8, 110.7, 95.8, 61.5, 39.0, 30.5, 26.1, 18.6, –5.2 ppm.

IR (thin film) 3340, 2939, 1698, 1508, 1259, 1103, 835 cm^{-1}.

HRMS (CI+) Calcd for $C_{25}H_{32}O_3SSi$ [M^+]: 440.1841, Found: 440.1831.

(*R*)-*S*-(6-Chloro-1-(4-hydroxyphenyl)-1-phenylhexa-1,2-dien-3-yl) ethanethioate (5r)

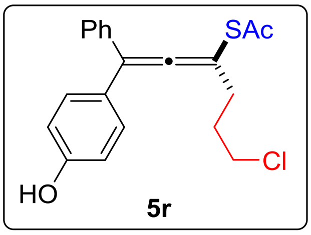

was prepared as brown oil from substrate **1z** (0.3 mmol, 90.3 mg) according to the General Procedure D (purified by flash column chromatography: 10% EtOAc and 1% AcOH in hexanes, 103.2 mg, 96% yield, 93% ee).

$[\alpha]_D^{25}$: –24.9 (c = 1.0, $CHCl_3$). HPLC analysis of the product: Daicel CHIRALPAK AD-H column; 10% *i*-PrOH in hexanes; 1.0 mL/min; retention times: 7.3 min (minor), 8.0 min (major).

^{1}H NMR (400 MHz, CD_2Cl_2) δ 7.41-7.30 (m, 5H), 7.23 (d, J = 8.8 Hz, 2H), 6.84 (d, J = 8.8 Hz, 2H), 5.78 (br s, 1H), 3.55 (t, J = 6.8 Hz, 2H), 2.60 (t, J = 6.8 Hz, 2H), 2.38 (s, 3H), 2.10-2.02 (m, 2H) ppm.

^{13}C NMR (100 MHz, CD_2Cl_2) δ 208.1, 195.5, 156.3, 136.0, 130.5, 129.1, 128.9, 128.3, 127.6, 115.8, 111.5, 97.3, 44.6, 33.1, 31.2, 30.6 ppm.

IR (thin film) 3421, 3029, 2926, 1675, 1511, 1269, 1118, 837 cm^{-1}.

HRMS (CI+) Calcd for $C_{20}H_{19}{}^{35}ClO_2S$ [M^+]: 358.0794, Found: 358.0779.

(*R*)-*S*-(6-Cyano-1-(4-hydroxyphenyl)-1-phenylhexa-1,2-dien-3-yl) ethanethioate (5s)

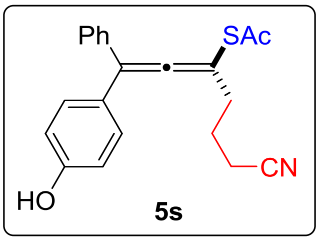

was prepared as brown oil from substrate **1aa** (0.3 mmol, 87.5 mg) according to the General Procedure D (purified by flash column chromatography: 10% EtOAc and 1% AcOH in hexanes, 81.6 mg, 78% yield, 92% ee).

$[\alpha]_D^{25}$: –10.7 (c = 1.0, $CHCl_3$). HPLC analysis of the product: Daicel CHIRALPAK AD-H column; 10% *i*-PrOH in hexanes; 1.0 mL/min; retention times: 13.3 min (minor), 15.3 min (major).

^{1}H NMR (400 MHz, CD_2Cl_2) δ 7.41-7.30 (m, 5H), 7.21 (d, J = 8.8 Hz, 2H), 6.84 (d, J = 8.8 Hz, 2H), 5.87 (br s, 1H), 2.57 (t, J = 7.2 Hz, 2H), 2.76 (s, 3H), 2.35 (t, J = 7.2 Hz, 2H), 1.97-1.89 (m, 2H) ppm.

^{13}C NMR (100 MHz, CD_2Cl_2) δ 208.2, 195.0, 156.5, 135.8, 130.5, 129.1, 128.9, 128.4, 127.3, 119.8, 115.9, 111.8, 96.8, 34.7, 30.5, 24.2, 16.7 ppm.

IR (thin film) 3227, 1610, 1511, 1269, 1114, 771 cm^{-1}.

HRMS (CI+) Calcd for $C_{21}H_{19}NO_2S$ [M$^+$]: 349.1136, Found: 349.1135.

(*R*)-*S*-(1-Cyclopropyl-3-(4-hydroxyphenyl)-3-phenylpropa-1,2-dien-1-yl) et

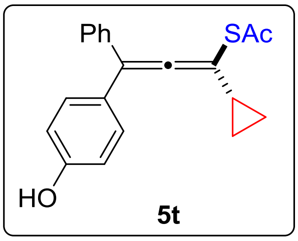

hanethioate (5t) was prepared as brown oil from substrate **1ab** (0.3 mmol, 79.2 mg) according to the General Procedure D (purified by flash column chromatography: 10% EtOAc and 1% AcOH in hexanes, 81.9 mg, 85% yield, 92% ee).

$[\alpha]_D^{25}$: –44.3 (c = 1.0, $CHCl_3$). HPLC analysis of the product: Daicel CHIRALPAK AD-H; 3% *i*-PrOH in hexanes; 1.0 mL/min; retention times: 24.8 min (minor), 26.1 min (major).

^{1}H NMR (400 MHz, acetone-d_6) δ 8.59 (br s, 1H), 7.42-7.30 (m, 5H), 7.18 (d, J = 8.4 Hz, 2H), 6.87 (d, J = 8.4 Hz, 2H), 2.37 (s, 3H), 1.73-1.65 (m, 1H), 0.82-0.78 (m, 2H), 0.59-0.49 (m, 2H) ppm.

^{13}C NMR (100 MHz, acetone-d_6) δ 207.9, 193.9, 158.2, 136.6, 130.6, 129.3, 129.2, 128.5, 126.8, 116.2, 112.1, 101.7, 30.0, 15.9, 7.48, 7.46 ppm.

IR (thin film) 3418, 3015, 2963, 1691, 1507, 1266, 1119, 832, 700 cm^{-1}.

HRMS (CI+) Calcd for $C_{20}H_{18}O_2S$ [M$^+$]: 322.1028, Found: 322.1016.

(*R*)-*S*-(1-(4-Hydroxyphenyl)-4-methyl-1-phenylpenta-1,2-dien-3-yl) ethanethioate

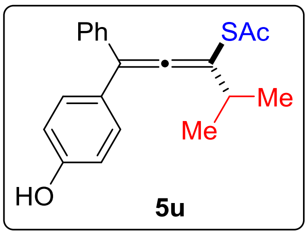

(5u) was prepared as brown oil from substrate **1ac** (0.3 mmol, 79.8 mg) according to the General Procedure D (purified by flash column chromatography: 10% EtOAc and 1% AcOH in hexanes, 87.8 mg, 90% yield, 92% ee).

$[\alpha]_D^{25}$: –12.1 (*c* = 1.0, $CHCl_3$). HPLC analysis of the product: Daicel CHIRALPAK AD-H column; 2% *i*-PrOH in hexanes; 1.0 mL/min; retention times: 19.9 min (minor), 21.8 min (major).

^{1}H NMR (400 MHz, acetone-d_6) δ 8.56 (br s, 1H), 7.48-7.38 (m, 4H), 7.38-7.28 (m, 1H), 7.22 (d, *J* = 8.8 Hz, 2H), 6.88 (d, *J* = 8.8 Hz, 2H), 2.98-2.51 (m, 1H), 2.36 (s, 3H), 1.18-1.15 (m, 6H) ppm.

^{13}C NMR (100 MHz, acetone-d_6) δ 207.4, 194.0, 158.0, 136.7, 130.5, 129.2, 129.1, 128.3, 126.9, 116.1, 111.9, 105.0, 35.2, 30.0, 21.7 ppm.

IR (thin film) 3415, 2965, 1671, 1508, 1222, 1115, 832, 700 cm^{-1}.

HRMS (CI+) Calcd for $C_{20}H_{20}O_2S$ [M$^+$]: 324.1184, Found: 324.1183.

(*R*)-*S*-(1-(4-Hydroxyphenyl)-4,4-dimethyl-1-phenylpenta-1,2-dien-3-yl) ethane-

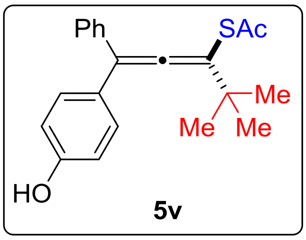

thioate (5v) was prepared as brown oil from substrate **1ad** (0.3 mmol, 84.5 mg) according to the General Procedure D (purified by flash column chromatography: 10% EtOAc and 1% AcOH in hexanes, 90.2 mg, 89% yield, 94% ee).

$[\alpha]_D^{25}$: –35.2 (*c* = 1.0, $CHCl_3$). HPLC analysis of the product: Daicel CHIRALPAK AD-H column; 5% *i*-PrOH in hexanes; 1.0 mL/min; retention times: 7.9 min (minor), 8.9 min (major).

^{1}H NMR (400 MHz, acetone-*d*$_6$) δ 8.55 (br s, 1H), 7.44-7.36 (m, 4H), 7.34-7.28 (m, 1H), 7.25 (d, *J* = 8.8 Hz, 2H), 6.88 (d, *J* = 8.8 Hz, 2H), 2.35 (s, 3H), 1.18 (s, 9H) ppm.

^{13}C NMR (100 MHz, acetone-*d*$_6$) δ 208.1, 193.3, 157.9, 136.8, 130.5, 129.2, 129.1, 128.2, 127.0, 116.1, 110.7, 107.2, 37.9, 29.8, 28.9 ppm.

IR (thin film) 3446, 2966, 1674, 1508, 1222, 1118, 759 cm^{-1}.

HRMS (CI+) Calcd for $C_{21}H_{22}O_2S$ [M$^+$]: 338.1341, Found: 338.1357.

(*S*)-S-(3-(4-Hydroxyphenyl)-1-phenyl-3-(4-(prop-2-yn-1-yloxy)phenyl)propa-1,2-dien-1-yl) ethanethioate (5w) was prepared as brown oil from substrate **1ae** (0.3 mmol, 106.2 mg) according to the General Procedure D (purified by flash column chromatography: 20% EtOAc and 1% AcOH in hexanes, 62.8 mg, 51% yield, 93% ee).

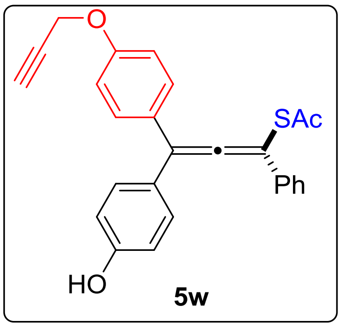

$[\alpha]_D^{25}$: –24.1 (*c* = 1.0, $CHCl_3$). HPLC analysis of the product: Daicel CHIRALPAK AD-H column; 20% *i*-PrOH in hexanes; 1.0 mL/min; retention times: 9.1 min (minor), 14.9 min (major).

The pure form of **5w** is relatively unstable, but it can be stored as a solution in DCM or $CHCl_3$ in the refrigerator. However, its TBS-protected derivative **5w′** is stable. Therefore, the characterization of **5w′** is provided.

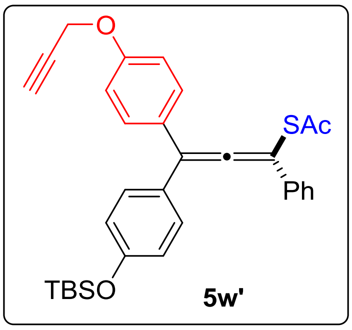

^{1}H NMR (400 MHz, $CDCl_3$) δ 7.61 (d, *J* = 8.0 Hz, 2H), 7.41-7.23 (m, 7H), 6.99 (d, *J* = 8.8 Hz, 2H), 6.85 (d, *J* = 8.8 Hz, 2H), 4.71 (s, 2H), 2.54 (s, 1H), 2.43 (s, 3H), 1.0 (s, 9H), 0.23 (s, 6H) ppm.

^{13}C NMR (100 MHz, $CDCl_3$) δ 211.4, 193.9, 157.5, 155.8, 134.5, 130.1, 130.0, 128.6, 128.0, 127.8, 127.3, 126.4, 120.2, 115.0, 112.0, 99.1, 78.4, 75.7, 55.8, 30.1, 25.6, 18.2, 4.4 ppm.

IR (thin film) 3443, 3294, 2930, 1705, 1603, 1507, 1261, 1029, 909, 837 cm^{-1}.

HRMS (CI+) Calcd for $C_{32}H_{34}O_3SSi$ [M$^+$]: 526.1998, Found: 526.2009.

(*S*)-*S*-(3-(3,5-Dimethoxyphenyl)-3-(4-hydroxyphenyl)-1-phenylpropa-1,2-dien-1-yl) ethanethioate (5x) was prepared as brown oil from substrate **1af** (0.3 mmol, 108.1 mg) according to the General Procedure D (purified by flash column chromatography: 20% EtOAc and 1% AcOH in hexanes, 80.3 mg, 64% yield, 92% ee).

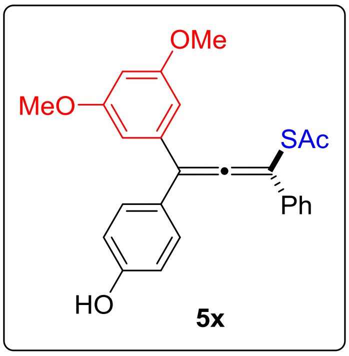

$[\alpha]_D^{25}$: –47.3 (*c* = 1.0, $CHCl_3$). HPLC analysis of the product: Daicel CHIRALPAK AD-H column; 15% *i*-PrOH in hexanes; 1.0 mL/min; retention times: 7.5 min (minor), 16.5 min (major).

^{1}H NMR (400 MHz, CD_2Cl_2) δ 7.61 (d, *J* = 7.2 Hz, 2H), 7.36 (t, *J* = 7.2 Hz, 2H), 7.31-7.27 (m, 3H), 6.82 (d, *J* = 8.4 Hz, 2H), 6.61-6.05 (m, 2H), 6.47-6.45 (m, 2H), 3.77 (s, 6H), 2.44 (s, 3H) ppm.

^{13}C NMR (100 MHz, CD_2Cl_2) δ 211.6, 194.7, 161.3, 156.5, 137.4, 134.6, 130.7, 129.1, 128.4 (2C), 126.7, 115.9, 112.8, 107.3, 100.6, 99.7, 55.8, 30.5 ppm.

IR (thin film) 3474, 2961, 1680, 1593, 1511, 1204, 1155, 837 cm^{-1}.

HRMS (CI+) Calcd for $C_{25}H_{22}O_4S$ [M$^+$]: 418.1239, Found: 418.1237.

(*R*)-*S*-(1-(4-Hydroxy-3-methylphenyl)-1-phenylhepta-1,2-dien-3-yl) ethanethioate (5y) was prepared as brown oil from substrate **1ag** (0.3 mmol, 88.4 mg) according to the General Procedure D (purified by flash column chromatography: 10% EtOAc and 1% AcOH in hexanes, 98.3 mg, 93% yield, 91% ee).

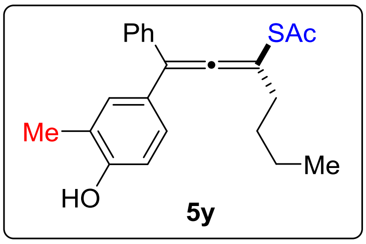

$[\alpha]_D^{25}$: –22.0 (*c* = 1.0, $CHCl_3$). HPLC analysis of the product: Daicel CHIRALPAK AD-H column; 5% *i*-PrOH in hexanes; 1.0 mL/min; retention times: 7.6 min (minor), 9.0 min (major).

^{1}H NMR (400 MHz, CD_2Cl_2) δ 7.37 (d, *J* = 8.4 Hz, 4H), 7.34-7.28 (m, 1H), 7.13-7.12 (m, 1H), 7.08-7.05 (m, 1H), 6.77 (d, *J* = 8.4 Hz, 1H), 2.44 (t, *J* = 7.6 Hz, 2H), 2.37 (s, 3H), 2.23 (s, 3H), 1.62-1.53 (m, 2H), 1.36 (q, *J* = 7.6 Hz, 2H), 0.89 (t, *J* = 7.6 Hz, 3H) ppm.

^{13}**C NMR** (100 MHz, CD_2Cl_2) δ 208.0, 195.3, 154.4, 136.5, 131.7, 129.2, 128.8, 128.1, 128.0, 127.8, 124.5, 115.3, 111.0, 98.7, 35.6, 30.52, 30.50, 22.6, 16.0, 14.0 ppm.

IR (thin film) 3400, 3023, 2928, 1685, 1503, 1278, 1116, 954, 824, 758 cm^{-1}.

HRMS (CI+) Calcd for $C_{22}H_{24}O_2S$ [M$^+$]: 352.1497, Found: 352.1501.

(*R*)-S-(1-(4-Hydroxyphenyl)-1-phenylhepta-1,2-dien-3-yl) benzothioate (5z) was

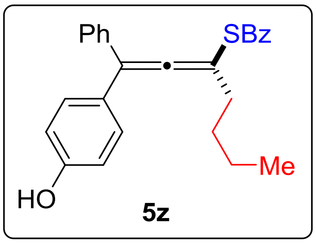

prepared as pale yellow solid from substrate **1u** (0.3 mmol, 84.5 mg) according to the General Procedure D (catalyst (*R*)-**A7** instead of catalyst (*S*)-**B1** and thiobenzoic acid instead of thioacetic acid, purified by flash column chromatography: 8% EtOAc and 2% AcOH in hexanes, 97.2 mg, 81% yield, 90% ee). $[\alpha]_D^{25}$: +27.5 (*c* = 1.0, $CHCl_3$). HPLC analysis of the product: Daicel CHIRALPAK AD-H column; 5% *i*-PrOH in hexanes; 1.0 mL/min; retention times: 11.3 min (major), 15.9 min (minor).

^{1}**H NMR** (400 MHz, acetone-d_6) δ 8.15 (br s, 1H), 7.98 (d, *J* = 8.4 Hz, 2H), 7.68 (t, *J* = 7.6 Hz, 1H), 7.55 (t, *J* = 7.6 Hz, 2H), 7.42-7.34 (m, 4H), 7.34-7.31 (m, 1H), 7.24 (d, *J* = 8.4 Hz, 2H), 6.89 (d, *J* = 8.8 Hz, 2H), 2.55 (t, *J* = 7.6 Hz, 2H), 1.68-1.59 (m, 2H), 1.44-1.33 (m, 2H), 0.88 (t, *J* = 7.6 Hz, 3H) ppm.

^{13}**C NMR** (100 MHz, acetone-d_6) δ 208.7, 190.4, 158.2, 137.5, 136.7, 134.5, 130.6, 129.7, 129.4, 129.2, 128.5, 127.9, 126.9, 116.2, 111.5, 98.2, 35.7, 30.7, 22.6, 14.0 ppm.

IR (thin film) 3416, 2929, 1669, 1510, 1206, 904, 767 cm^{-1}.

HRMS (CI+) Calcd for $C_{26}H_{24}O_2S$ [M$^+$]: 400.1497, Found: 400.1505.

Supplementary Note 1. Mechanistic Study

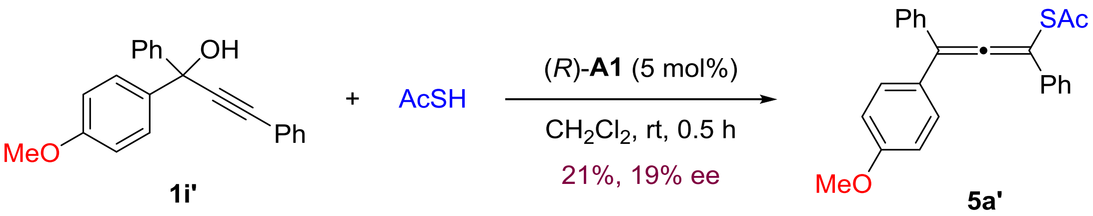

***S*-(3-(4-methoxyphenyl)-1,3-diphenylpropa-1,2-dien-1-yl) ethanethioate (5a′)** was prepared as red oil from propargylic alcohol **1i′** (94.3 mg, 0.3 mmol) according to the General Procedure D (with CH_2Cl_2 as solvent, and without 3Å MS, room temperature for 0.5 h (21% ^{1}H NMR yield) or 72 h (full conversion), purified by flash column chromatography: 10% EtOAc in hexanes, 74.8 mg, 67% yield, 19% ee).

$[\alpha]_D^{25}$: +4.2 (c = 1.0, $CHCl_3$). HPLC analysis of the product: Daicel CHIRALPAK AD-H column; 5% *i*-PrOH in hexanes; 1.0 mL/min; retention times: 8.5 min (major), 10.0 min (minor).

^{1}H NMR (400 MHz, $CDCl_3$) δ 7.62 (d, J = 8.0 Hz, 2H), 7.46 (d, J = 8.4 Hz, 2H), 7.41-7.33 (m, 7H), 7.29-7.24 (m, 1H), 6.93 (d, J = 8.8 Hz, 2H), 3.83 (s, 3H), 2.43 (s, 3H) ppm.

^{13}C NMR (100 MHz, $CDCl_3$) δ 211.4, 193.7, 159.6, 135.0, 134.3, 120.1, 128.9, 128.6, 128.1, 127.9, 126.7, 126.4, 114.1, 112.4, 99.3, 55.3, 30.1 ppm.

IR (thin film) 3057, 2932, 1605, 1509, 1444, 1250, 1178, 1034, 832, 698 cm^{-1}.

HRMS (CI+) Calcd for $C_{24}H_{20}O_2S$ [M$^+$]: 372.1184, Found: 372.1182.

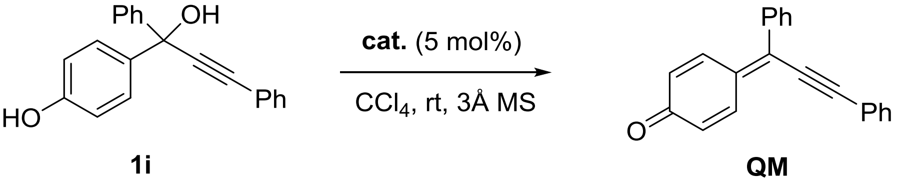

4-(1,3-Diphenylprop-2-yn-1-ylidene)cyclohexa-2,5-dienone (QM). To a solution of substrate **1i** (300.4 mg, 1.0 mmol) in CCl_4 (10 mL) were added 3Å MS (300 mg) and (*rac*)-**A7** (17.4 mg, 0.05 mmol) or (*S*)-**B1** (42.5 mg, 0.05 mmol). The reaction was stirred at room temperature for 1-2 h to give *para*-quinone methide **QM** as a red solid

(purified by flash column chromatography: 5% EtOAc in hexanes, 211.8 mg, 75% yield for **A7**; 104.5 mg, 37% yield for **B1**).

^{1}H NMR (400 MHz, acetone-*d*$_6$) δ 8.18 (dd, *J* = 2.4 Hz, *J* = 10 Hz, 1H), 7.68-7.62 (m, 4H), 7.58-7.52 (m, 3H), 7.51-7.44 (m, 4H), 6.48 (dd, *J* = 2.0 Hz, *J* = 10 Hz, 1H), 6.32 (dd, *J* = 2.0 Hz, *J* = 10 Hz, 1H) ppm.

^{13}C NMR (100 MHz, acetone-*d*$_6$) δ 186.7, 138.6, 138.1, 137.4, 136.5, 135.6, 132.7, 130.93, 130.89, 130.7, 130.0, 129.6, 129.5, 129.2, 122.6, 108.2, 90.4 ppm.

IR (thin film) 2976, 2180, 1625, 1501, 1444, 1166, 864, 698 cm^{-1}.

HRMS (CI+) Calcd for $C_{21}H_{14}O$ [M$^+$]: 282.1045, Found: 282.1058.

Kinetic Study

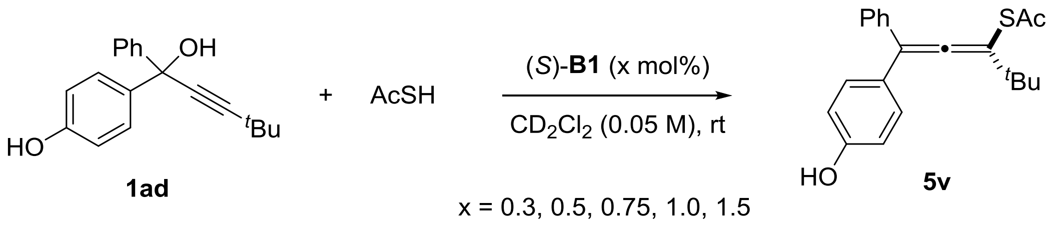

At room temperature, to an oven-dried 4-mL vial charged with a solution of propargylic alcohol **1ad** (14 mg, 0.05 mmol, 1.0 equiv.) in CD_2Cl_2 (1.0 mL) was added a solution of the catalyst (*S*)-**B1** (1st run: 0.13 mg, 0.15 μmol, 0.3 mol%; 2nd run: 0.21 mg, 0.25 μmol, 0.5 mol%; 3rd run: 0.32 mg, 0.38 μmol, 0.75 mol%; 4th run: 0.43 mg, 0.5 μmol, 1.0 mol%; 5th run: 0.64 mg, 0.75 μmol, 1.5 mol%) and thioacetic acid (7.2 uL, 0.1 mmol, 2.0 equiv.) in CD_2Cl_2 (50 uL). The homogeneous reaction mixture was transferred to an NMR tube. The progress of the five reactions was monitored by ^{1}H NMR every 2 min.

As shown in Supplementary Figure 219, a linear relationship between the initial rates and catalyst loading was observed, indicating 1st-order kinetics in catalyst.

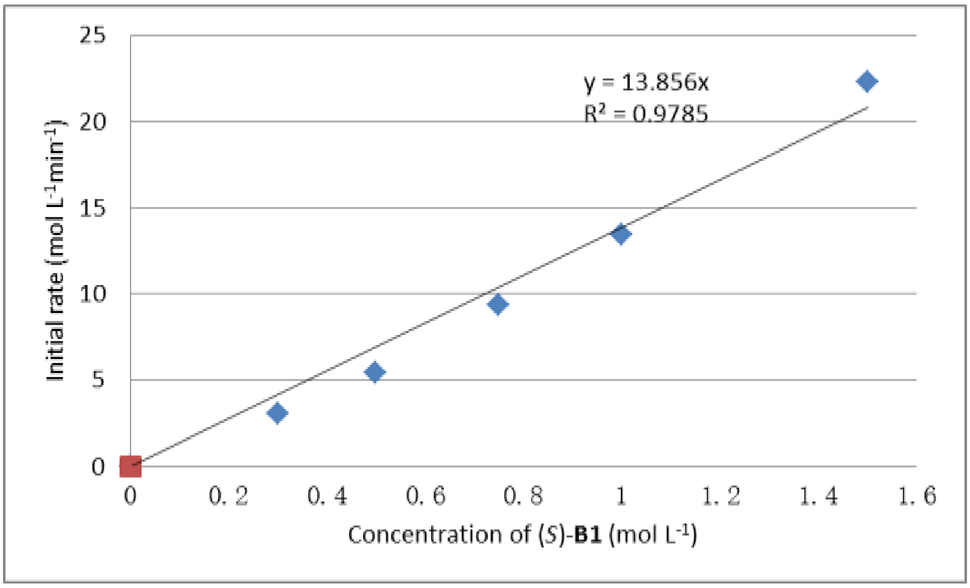

Supplementary Figure 219. First-order kinetics in catalyst.

Supplementary Note 2. Product Derivatizations

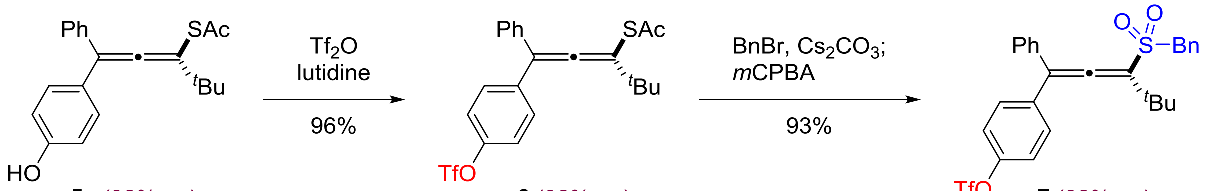

5v (92% ee) **6** (92% ee) **7** (82% ee)

(*R*)-*S*-(4,4-Dimethyl-1-phenyl-1-(4-(((trifluoromethyl)sulfonyl)oxy)phenyl)penta-1,2-dien-3-yl) ethanethioate (6). At 0 °C, to a solution of **5v** (338.2 mg, 1.0 mmol) in CH_2Cl_2 (10 mL) were added 2,6-lutidine (419.3 uL, 3.6 mmol, 3.6 equiv) and a solution of Tf_2O (370.1 uL, 2.2 mmol, 2.2 equiv) in CH_2Cl_2 (0.5 mL) sequentially. The reaction mixture was stirred at 0 °C for 10 min and then allowed to warm to room temperature. The mixture was concentrated, and the residue was purified by silica gel column chromatography (eluent: 3% Et_2O in hexanes, colorless oil, 451.7 mg, 96 yield, 92% ee).

$[\alpha]_D^{25}$: +22.8 (c = 1.0, $CHCl_3$). HPLC analysis of the product: Daicel CHIRALCEL OD-H column; 1% *i*-PrOH in hexanes; 0.5 mL/min; retention times: 7.8 min (major), 8.6 min (minor).

^{1}H NMR (400 MHz, $CDCl_3$) δ 7.53 (d, J = 8.8 Hz, 2H), 7.42-7.31 (m, 5H), 7.27 (d, J = 8.8 Hz, 2H), 2.37 (s, 3H), 1.19 (s, 9H) ppm.

^{13}C NMR (100 MHz, $CDCl_3$) δ 207.4, 192.9, 148.7, 136.4, 135.1, 130.3, 128.64, 128.62, 127.9, 121.3, 118.8 (d, $J_{C\text{-}F}$ = 319 Hz), 109.0, 107.8, 37.4, 30.1, 28.8 ppm.

^{19}F NMR (376 MHz, $CDCl_3$) δ –72.9 ppm.

IR (thin film) 3521, 2967, 2882, 1708, 1426, 1214, 1142, 887 cm^{-1}.

HRMS (CI+) Calcd for $C_{22}H_{25}F_3NO_4S_2$ [M+NH_4^+]: 488.1177, Found: 488.1164.

(*R*)-4-(3-(Benzylsulfonyl)-4,4-dimethyl-1-phenylpenta-1,2-dien-1-yl)phenyltrifluoromethanesulfonate (7). At room temperature, to a stirred solution of Cs_2CO_3 (117.6 mg, 0.36 mmol, 1.2 equiv) in MeOH (6.0 mL) was added a solution of **6** (141.2 mg, 0.3

mmol, 1.0 equiv) and benzyl bromide (71.3 uL, 0.6 mmol, 2.0 equiv) in MeOH (0.3 mL) dropwise. The mixture was stirred for 20 min. Next, Et_2O (10 mL) was added, and the mixture was filtered through a short pad of silica gel, which was washed with Et_2O (20 mL). The filtrate was concentrated to the crude product as a pale green oil, which was used for the next step without further purification.

At room temperature, the above oil was dissolved in CH_2Cl_2 (6 mL) and then treated with *m*CPBA (151.9 mg, 0.66 mmol, 75 wt%, 2.2 equiv). The reaction mixture was stirred for 5 min before a saturated aqueous $NaHCO_3$ solution (10 mL) was added. The layers were separated and the aqueous layer was extracted with CH_2Cl_2 (3×15 mL). The combined organic layers were washed with a saturated aqueous Na_2SO_3 solution, a saturated aqueous $NaHCO_3$ solution, and brine, dried over Na_2SO_4, and concentrated. The residue was purified by silica gel flash column chromatography (5% Et_2O in hexanes) to give pure sulfone **7** (153.6 mg, 93% yield, 82% ee).

$[\alpha]_D^{25}$: –44.8 (c = 1.0, $CHCl_3$). HPLC analysis of the product: Daicel CHIRALPAK AD-H column; 5% *i*-PrOH in hexanes; 1.0 mL/min; retention times: 15.9 min (major), 17.6 min (minor).

^{1}H NMR (400 MHz, CD_2Cl_2) δ 7.50-7.43 (m, 5H), 7.38-7.31 (m, 4H), 7.27-7.17 (m, 3H), 7.13-7.10 (m, 2H), 4.26 (s, 2H), 1.33 (s, 9H) ppm.

^{13}C NMR (100 MHz, CD_2Cl_2) δ 206.0, 149.9, 135.6, 134.2, 131.3, 130.9, 129.5, 129.1, 129.0, 128.8, 128.1, 124.1, 122.2, 119.2 (d, $J_{C\text{-}F}$ = 319 Hz), 117.1 (2C), 63.5, 37.5, 29.8 ppm.

^{19}F NMR (376 MHz, CD_2Cl_2) δ –73.2 ppm.

IR (thin film) 3444, 2970, 1934, 1498, 1425, 1313, 1214, 1141, 1016, 887, 698 cm^{-1}.

HRMS (CI+) Calcd for $C_{27}H_{29}F_3NO_5S_2$ [M+NH_4^+]: 568.1439, Found: 568.1470.

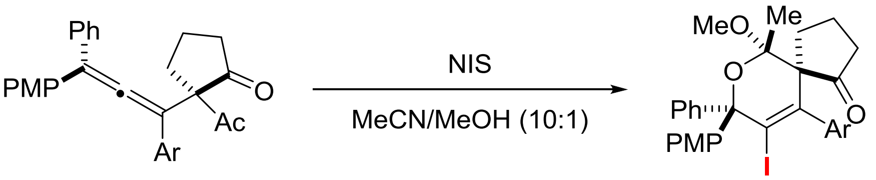

3b
(7:1 d.r., 94% *ee*)

PMP = *p*-(MeO)C_6H_4, Ar = *p*-ClC_6H_4

8
(7:1 d.r., 93% *ee*)

(5*R*,6*S*,8*R*)-10-(4-Chlorophenyl)-9-iodo-6-methoxy-8-(4-methoxyphenyl)-6-methyl-8-phenyl-7-oxaspiro[4.5]dec-9-en-1-one (8). Under N_2, the tetrasubstituted allene **3b** (91.2 mg, 0.2 mmol, 1 equiv) was dissolved in anhydrous MeCN/MeOH (10:1, 4 mL) and then *N*-iodosuccinimide (54.0 mg, 0.24mmol, 1.2 equiv) was added. After stirring at room temperature for 5 h, the reaction mixture was subjected to flash chromatography to give the desired product **8** as a white foam (eluent: 10% EtOAc in hexanes, 105.7 mg, 86% yield, 7:1 d.r., 93% ee).

$[\alpha]_D^{25}$: –26.1 (c = 1.0, $CHCl_3$). HPLC analysis of the product: Daicel CHIRALPAK AD-H; 5% *i*-PrOH in hexanes; 1.0 mL/min; retention times: 5.9 min (major), 8.2 min (minior). Daicel CHIRALPAK IC; 5% *i*-PrOH in hexanes; 0.5 mL/min; retention times: 8.9 (major), 11.9 min (minior).

^{1}H NMR (400 MHz, CD_2Cl_2) δ 7.90 (d, J = 7.6 Hz, 2H), 7.50 (d, J = 8.8 Hz, 2H), 7.45 (d, J = 8.4 Hz, 1H), 7.38-7.25 (m, 5H), 6.89 (d, J = 8.8 Hz, 2H), 6.70 (d, J = 8.8 Hz, 1H), 3.84 (s, 3H), 2.35-2.08 (m, 4H), 2.26 (s, 3H), 1.63-1.54 (m, 1H), 1.28 (s, 3H), 0.59-0.45 (m, 1H) ppm.

^{13}C NMR (100 MHz, CD_2Cl_2) δ 217.6, 159.2, 145.9, 143.7, 136.2, 134.1, 133.4, 132.1, 131.6, 130.3, 129.3, 128.0, 127.1, 112.8, 112.3, 102.4, 83.8, 63.1, 55.8, 48.4, 40.8, 32.9, 19.6, 19.0 ppm.

IR (thin film) 2963, 1736, 1608, 1509, 1376, 1249, 1140, 998, 829, 700 cm^{-1}.

HRMS (CI+) Calcd for $C_{30}H_{28}{}^{35}ClIO_4$ [M^+]: 614.0721, Found: 614.0725.

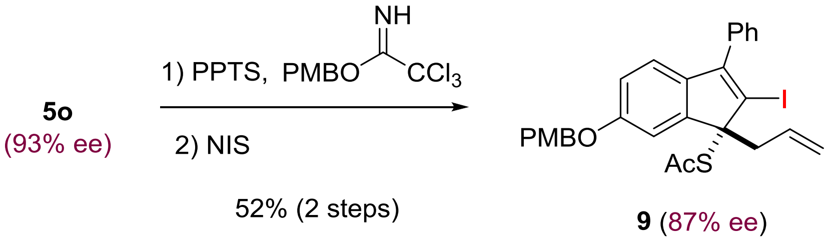

(*R*)-*S*-(1-Allyl-2-iodo-6-((4-methoxybenzyl)oxy)-3-phenyl-1H-inden-1-yl) ethanethioate (9). At room temperature, to a solution of **5o** (64.5 mg, 0.2 mmol) and *p*-methoxybenzyl trichloroacetimidate (PMBTCA)[7] in CCl_4 (2 mL) was added PPTS (25.1 mg, 0.1 mmol, 0.5 equiv). The mixture was stirred for 18 h, and then a saturated aqueous solution of $NaHCO_3$ (2 mL) was added. The layers were separated, and the aqueous layer was extracted with ethyl acetate (2 mL×3). The combined organic layers were concentrated. The residue was dissolved in DCM and then triturated with hexanes. The resulting mixture was filtered and the filtrate was concentrated. The residue was purified by silica gel column chromatography (eluent: 10% DCM and 1% Et_2O in hexanes) to give a pale yellow oil (75% yield based on ^1H NMR).

To a solution of *N*-iodosuccinimide (40.5 mg, 0.18 mmol, 1.2 equiv) in nitromethane (1 mL) was added a solution of the above oil (0.15 mmol) in nitromethane (0.5 mL). The mixture was stirred at room temperature for 30 min, and then a saturated solution of sodium thiosulfate (1 mL) was added to quench the reaction. The mixture was extracted with ethyl acetate (2×20 mL). The combined organics layers were washed with brine, dried over $MgSO_4$, filtered, and concentrated. The residue was purified by flash chromatography (eluent: 10% DCM and 1% Et_2O in hexanes) to get the iodide **9** as a colorless oil (59.2 mg, 52% yield, 87% ee).

$[\alpha]_D^{25}$: –31.1 (c = 1.0, $CHCl_3$). HPLC analysis of the product: Daicel CHIRALPAK AD-H column; 5% *i*-PrOH in hexanes; 1.0 mL/min; retention times: 8.0 min (minor), 12.6 min (major).

^{1}H NMR (400 MHz, $CDCl_3$) δ 7.48-7.37 (m, 8H), 7.06 (d, *J* = 8.4 Hz, 1H), 6.93 (d, *J* = 8.8 Hz, 2H), 6.84 (d, *J* = 8.8 Hz, 1H), 5.23-5.12 (m, 1H), 5.06 (s, 1H), 5.02 (s, 2H), 4.92 (d, *J* = 10 Hz, 1H), 3.82 (s, 3H), 3.12-3.06 (m, 1H), 2.74-2.67 (m, 1H), 2.21 (s, 3H) ppm.

^{13}C NMR (100 MHz, $CDCl_3$) δ 193.2, 159.5, 157.8, 150.0, 148.3, 137.1, 135.1, 130.1, 129.4, 128.8, 128.4, 128.3 (2C), 120.8, 119.3, 114.0, 113.7, 111.3, 103.7, 70.1, 66.3, 55.3, 40.0, 30.7 ppm.

IR (thin film) 3445, 1688, 1514, 1424, 1248, 1140, 1018, 824, 699 cm^{-1}.

HRMS (CI+) Calcd for $C_{28}H_{25}IO_3S$ [M^+]: 568.0569, Found: 568.0558.

Supplementary Note 3. Determination of the Absolute Stereochemistry

(1) The absolute stereochemistry of product **3n** was determined by X-ray diffraction (shown below). The X-ray data of **3n** have been deposited at the Cambridge Crystallographic Data Center (CCDC 1498348). The stereochemistry of other products was assumed by analogy.

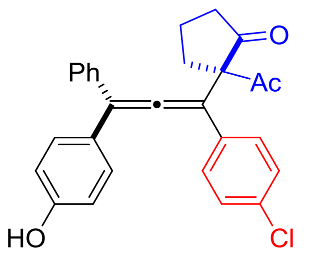

3n

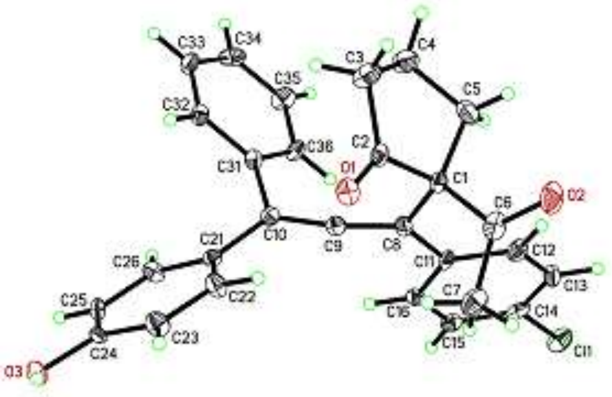

(2) The absolute stereochemistry of product **3b** was determined by comparing the optical rotation and HPLC spectra with that of **3n′** (the methyl-protected derivative of **3n**).

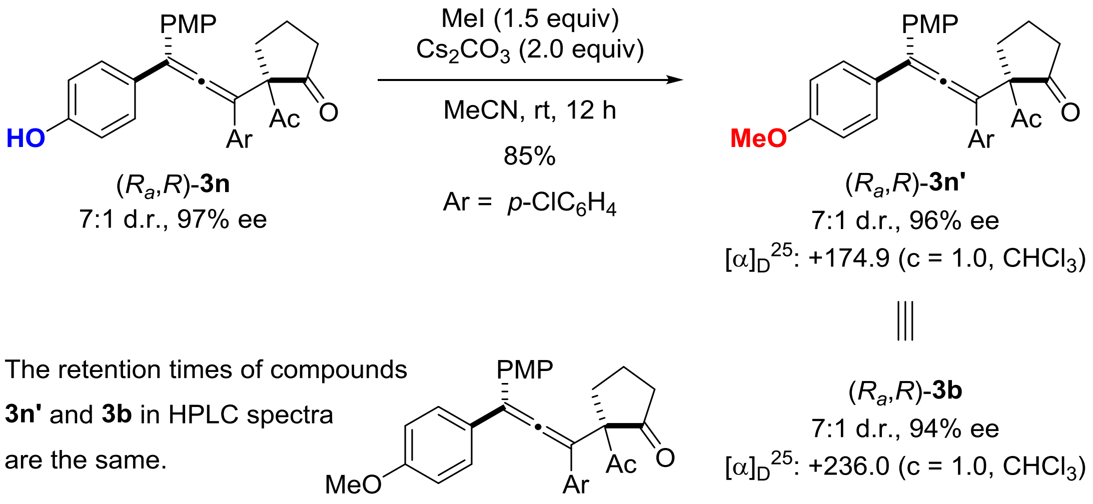

Supplementary Note 4. DFT Calculations

Using DFT calculations on a model system (Figure S1), we were able to locate the important transition state (**TS2**, as discussed in the main text) featuring a nucleophilic attack leading to the C-S bond formation. This transition state lies above the energy reference point (the substrate + a dimer formed between the catalyst and the nucleophile) by 11.4 kcal/mol in free energy, which is consistent with the experimental observations that the reaction can take place smoothly at room temperature. It is also consistent with the kinetic study and the proposed mechanism that only one catalyst molecule is sufficient to activate both of the reaction partners through the bifunctional model.

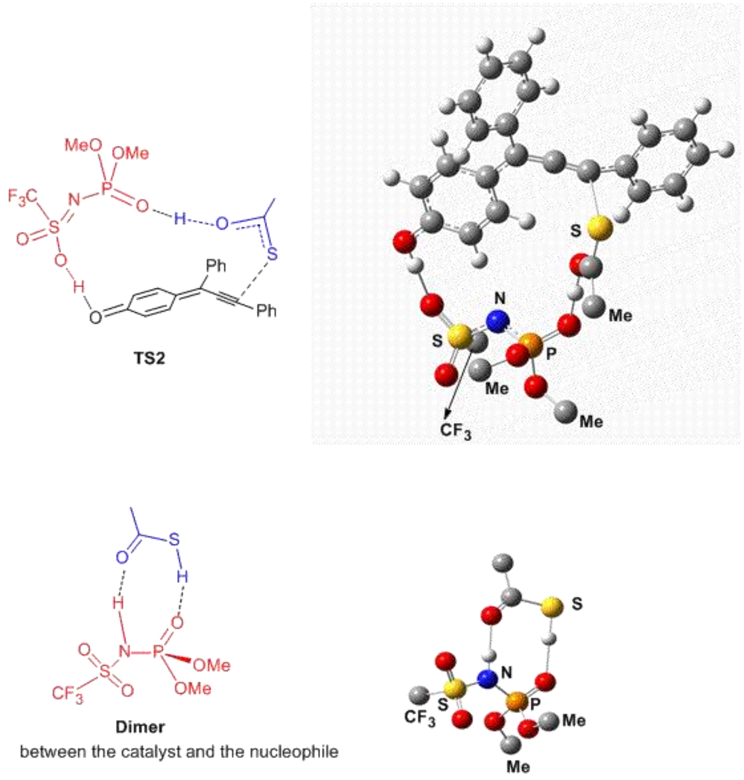

Supplementary Figure 220. The structures calculated for TS2 and the dimer formed between the catalyst and nucleophile.

In the DFT calculations, the Becke three-parameter hybrid functional (B3LYP) method was employed.[8-10] The effective core potentials (ECPs) of Lanl2dz were used to describe S and P atoms,[11] with polarization functions for P (ζ_d =0.387) and S (ζ_d

=0.503) being added.[12] The two H atoms, which were related to prototropic, were described by the 6-31G** basis set.[13] The 6-31g* basis set was used for all remaining atoms.[14,15] Frequency calculations were performed to ensure that a transition state has only one imaginary frequency and the local minimum has no imaginary frequency. Intrinsic reaction coordinates (IRC)[16,17] were run to ensure that transitions states connect the relevant minima. All the calculations were performed in the Gaussian 09 program.[18]

Cartesian Coordinates for **TS2**, the dimer, and the substrate:

TS2

S 2.593479 -2.070699 1.888858
S -4.259545 1.204648 -0.379173
P -3.127975 -1.107498 0.958979
O -1.480505 4.052451 -0.822059
C -0.300446 1.973829 -0.538138
C 0.909955 1.337247 -0.377785
C 2.134534 2.057259 -0.341916
C 2.073609 3.467074 -0.515740
C 0.866279 4.114674 -0.681041
C -0.342557 3.380109 -0.681789
H -1.222387 1.401401 -0.544359
H 0.924772 0.259383 -0.255531
H 2.991539 4.043691 -0.546892
H 0.818028 5.189280 -0.824347
C 3.381548 1.348362 -0.167519
C 4.642376 2.029028 0.218228
C 4.652894 3.030844 1.206997
C 5.863400 1.638847 -0.362142
C 5.851015 3.627893 1.595026
H 3.724295 3.314584 1.691108
C 7.056203 2.247654 0.018413
H 5.861557 0.868228 -1.127082
C 7.054145 3.243786 0.998775
H 5.844668 4.389712 2.369460
H 7.988611 1.946083 -0.450546
H 7.985892 3.715044 1.298950
C 3.436027 -0.004556 -0.348746
C 3.414341 -1.250568 -0.417756
C 3.646753 -2.497872 -1.091485
C 2.575356 -3.250187 -1.611592
C 4.967534 -2.952209 -1.273027
C 2.829329 -4.424625 -2.312313
H 1.560078 -2.906761 -1.445029
C 5.210577 -4.128549 -1.977736
H 5.789069 -2.374153 -0.861269
C 4.144005 -4.865674 -2.498656
H 2.000584 -4.999625 -2.715498
H 6.231685 -4.471391 -2.118898
H 4.335601 -5.784352 -3.046248
C 0.912002 -2.200349 1.719188
C 0.071542 -2.630026 2.898920
H 0.685415 -2.812536 3.780569
H -0.679663 -1.860978 3.112712
H -0.477191 -3.541855 2.634601
O 0.312698 -1.946097 0.607670
N -3.101790 0.151327 -0.057500
O -3.641031 2.453866 -0.911447
O -5.334501 1.333120 0.625692
C -5.082762 0.492416 -1.912002
O -2.813845 -0.660057 2.503306
O -4.622349 -1.735905 0.981974
O -2.095079 -2.195504 0.653037
F -6.054183 1.317420 -2.316378
F -4.187617 0.356726 -2.895452
F -5.612487 -0.704724 -1.638032
C -4.864851 -2.941072 1.725755

H -4.658491 -2.788208 2.790340
H -5.921333 -3.176315 1.586099
H -4.245211 -3.757544 1.344283
C -3.473382 0.478029 3.089560
H -3.211137 0.464680 4.149384
H -3.115693 1.404094 2.630500
H -4.556869 0.415987 2.965748
H -2.262212 3.444266 -0.818069
H -0.793463 -2.040695 0.637366

Dimer

S 1.427189 -0.975729 0.926824
S -4.151019 0.554918 -0.235269
P 0.147815 1.606739 -0.105825
N 0.155994 -0.019856 0.394482
O 0.842003 -2.097356 1.666482
O 2.496416 -0.119171 1.449527
C 2.080374 -1.720044 -0.677208
O 1.124027 2.441006 0.866520
O 1.025964 1.574703 -1.455523
O -1.262215 2.101171 -0.201140
F 2.952249 -2.680633 -0.365504
F 1.067148 -2.244591 -1.369642
F 2.688135 -0.788924 -1.409841
C 0.736064 2.699111 2.231087
H 1.333897 3.549548 2.562240
H -0.327843 2.945005 2.292621
H 0.967230 1.824415 2.844130
C 1.313066 2.807127 -2.147713
H 1.788301 2.519990 -3.086563
H 0.389886 3.357705 -2.351299
H 1.996155 3.421280 -1.554839
C -3.496564 -1.099881 -0.019684
O -2.313185 -1.357502 0.126018
C -4.546338 -2.190872 -0.065281
H -5.531121 -1.832317 0.245281
H -4.623629 -2.558803 -1.095813
H -4.224579 -3.019449 0.570672
H -0.750622 -0.515196 0.322998
H -2.937045 1.190579 -0.225691

Substrate

O -4.538811 -3.757969 -0.044859
C -2.261254 -3.114073 0.130015
C -1.348996 -2.115267 0.127202
C -1.732703 -0.721982 -0.029844
C -3.144839 -0.449905 -0.238920
C -4.067045 -1.441542 -0.250775
C -3.699778 -2.853196 -0.049351
H -1.971341 -4.151521 0.269321
H -0.294691 -2.333184 0.268727
H -3.450939 0.574114 -0.423235
H -5.118517 -1.237165 -0.430653
C -0.771871 0.284576 -0.013558
C -1.092774 1.739733 -0.011956
C -2.043368 2.275739 0.874641
C -0.403231 2.615049 -0.869497
C -2.313351 3.644116 0.883903
H -2.550112 1.619116 1.575027
C -0.683067 3.979605 -0.866303
H 0.345548 2.213476 -1.545753
C -1.639944 4.499203 0.009731
H -3.044734 4.042133 1.581921
H -0.151833 4.639372 -1.546897
H -1.852335 5.564738 0.016609
C 0.608411 -0.024600 0.006911
C 1.811607 -0.229220 0.018562
C 3.212019 -0.470750 0.036078
C 4.118157 0.598938 0.198438
C 3.717795 -1.780153 -0.107633
C 5.488971 0.360278 0.215326
H 3.731113 1.606842 0.311354
C 5.090464 -2.008089 -0.087925
H 3.023692 -2.605144 -0.234393
C 5.979340 -0.941170 0.072818
H 6.177981 1.190795 0.341177
H 5.469100 -3.020291 -0.198848
H 7.050289 -1.123259 0.087336

Supplementary References

1. Gabbutt, C. D., Heron, B. M., Kilner, C. & Kolla, S. B. The influence of a 1,1-diarylvinyl moiety on the photochromism of naphthopyrans. *Org. Biomol. Chem.* **8**, 4874–4883 (2010).
2. Wei, L.-M., Wei, L.-L., Pan, W.-B. & Wu, M.-J. Synthesis of tetraarylallenes via palladium-catalyzed addition-Elimination reactions of 1,1,3-triaryl-2-propyn-1-ols with aryl iodides. *Synlett* 2219–2223 (2005).
3. Zhu, S., Niljianskul, N. & Buchwald, S. L. A direct approach to amines with remote stereocentres by enantioselective CuH-catalysed reductive relay hydroamination . *Nat. Chem*. **8**, 144–150 (2016).
4. van der Born, D., Sewing, C., Herscheid, J. D. M., Windhorst, A. D., Orru, R. V. A. & Vugts, D. J. A universal procedure for the [^{18}F]trifluoromethylation of aryl iodides and aryl boronic acids with highly improved specific activity. *Angew. Chem. Int. Ed*. **53**, 11046–11050 (2014).
5. Buzas, A. & Gagosz, F. Gold(I) catalyzed isomerization of 5-en-2-yn-1-yl acetates: an efficient access to acetoxy bicyclo[3.1.0]hexenes and 2-cycloalken-1-ones. *J. Am. Chem. Soc.* **128**, 12614–12615 (2006).
6. Hong, X., Küçük, H. B., Maji, M. S., Yang, Y.-F., Rueping, M. & Houk, K. N. Mechanism and selectivity of *N*-triflylphosphoramide catalyzed (3+2) cycloaddition between hydrazones and alkenes. *J. Am. Chem. Soc.* **136**, 13769–13780 (2014).
7. Kato, T., Sato, T., Kashiwagi, Y. & Hosokawa, S. Synthetic studies on Aculeximycin: synthesis of C24–C40 segment by Kobayashi aldolization and epoxide rearrangements. *Org. Lett*. **17**, 2274–2277 (2015).
8. Becke, A. D. Density-functional thermochemistry. III. The role of exact exchange. *J. Chem. Phys.* **98**, 5648–5652 (1993).
9. Lee, C., Yang, W. & Parr, R. G. Development of the Colle-Salvetti correlation-energy formula into a functional of the electron density. *Phys. Rev. B*. **37**, 785-787 (1988).
10. Stephens, P. J., Devlin, F. J., Chabalowski, C. & Frisch, M. J. Ab initio calculation of vibrational absorption and circular dichroism spectra using density functional force fields. *J. Phys. Chem.* **98**, 11623-11627 (1994).
11. Wadt, W. R. & Hay, P. J. Ab initio effective core potentials for molecular calculations. Potentials for main group elements Na to Bi. *J. Chem. Phys. 82*, 284-298 (1985).
12. Höllwarth, A., Böhme, M., Dapprich, S., Ehlers, A. W., Gobbi, A., Jonas, V., Köhler, K., Stegmann, R. & Frenking, G. A set of d-polarization functions for pseudo-potential basis sets of the main group elements Al-Bi and f-type polarization functions for Zn, Cd, Hg. *Chem. Phys. Lett*. **208**, 237-240 (1993).

13. Hariharan, P. C. & Pople, J. A. The influence of polarization functions on molecular orbital hydrogenation energies. *Theor. Chim. Acta***.** **28**, 213-222 (1973).
14. Petersson, A., Bennett, A., Tensfeldt, T. G., Al-Laham, M. A., Shirley, W. A. & Mantzaris, J. A complete basis set model chemistry. I. The total energies of closed-shell atoms and hydrides of the first-row elements. *J. Chem. Phys.* **89**, 2193-2218 (1988).
15. Petersson, G. A. & Al-Laham, M. A. A complete basis set model chemistry. II. Open-shell systems and the total energies of the first-row atoms. *J. Chem. Phys.* **94**, 6081-6090 (1991).
16. Fukui, K. The path of chemical reactions-the IRC approach. *Acc. Chem. Res.* **14**, 363-368 (1981).
17. Fukui, K. Formulation of the reaction coordinate. *J. Phys. Chem.* **74**, 4161-4163 (1970).
18. Frisch, M. J., Trucks, G. W., Schlegel, H. B., Scuseria, G. E., Robb, M. A., Cheeseman, J. R., Scalmani, G., Barone, V., Mennucci, B., Petersson, G. A., Nakatsuji, H., Caricato, M., Li, X., Hratchian, H. P., Izmaylov, A. F., Bloino, J., Zheng, G., Sonnenberg, J. L., Hada, M., Ehara, M., Toyota, K., Fukuda, R., Hasegawa, J., Ishida, M., Nakajima, T., Honda, Y., Kitao, O., Nakai, H., Vreven, T., Montgomery, J. A., Jr., Peralta, J. E., Ogliaro, F., Bearpark, M., Heyd, J. J., Brothers, E., Kudin, K. N., Staroverov, V. N., Kobayashi, R., Normand, J., Raghavachari, K., Rendell, A., Burant, J. C., Iyengar, S. S., Tomasi, J., Cossi, M., Rega, N., Millam, M. J., Klene, M., Knox, J. E., Cross, J. B., Bakken, V., Adamo, C., Jaramillo, J., Gomperts, R., Stratmann, R. E., Yazyev, O., Austin, A. J., Cammi, R., Pomelli, C., Ochterski, J. W., Martin, R. L., Morokuma, K., Zakrzewski, V. G., Voth, G. A., Salvador, P., Dannenberg, J. J., Dapprich, S., Daniels, A. D., Farkas, Ö., Foresman, J. B., Ortiz, J. V., Cioslowski, J. & Fox, D. J. Gaussian 09, Revision D.01, Gaussian, Inc., Wallingford, CT, 2009.
